# Supplementary material for: How AI threat perception relates to post-adoptive AI agent engagement: the dual paths of self-efficacy and defensive motivation
Source: Front Psychol. 2026 Oct 22;17:1887571. doi: 10.3389/fpsyg.2026.1887571 (PMC13735560; doi:10.3389/fpsyg.2026.1887571)
Supplement: Supplementary file 1 [file Data_Sheet_1.zip › log+Questionnaire/001exploratory factor analysis.htm]

xml version="1.0" encoding="UTF-8"?


输出1


|  |  |  |
| --- | --- | --- |
| IBM SPSS Web 报告 - 输出1     ---   内容  上一个  下一个  帮助 |  | 系统已将控件禁用    --- |

- 日志

  - 日志
- 因子分析

  - 活动数据集
  - 公因子方差
  - 总方差解释
  - 成分矩阵
  - 旋转后的成分矩阵
  - 成分转换矩阵
  - 成分得分系数矩阵
  - 成分得分协方差矩阵

- 删除

日志  
日志 - 日志 - 九月 6, 2026

GET  
  FILE='D:\学术研究\066研究数据与论文撰写\01研究数据\AI智能体论文\信效度检验\AI智能体数据.sav'.  
DATASET NAME 数据集1 WINDOW=FRONT.  
FACTOR  
  /VARIABLES AA1 AA2 AA3 AA4 AA5 SE1 SE2 SE3 SE4 SE5 DM1 DM2 DM3 DM4 DM5 AB1 AB2 AB3 AB4 AB5  
  /MISSING LISTWISE  
  /ANALYSIS AA1 AA2 AA3 AA4 AA5 SE1 SE2 SE3 SE4 SE5 DM1 DM2 DM3 DM4 DM5 AB1 AB2 AB3 AB4 AB5  
  /PRINT INITIAL EXTRACTION ROTATION FSCORE  
  /FORMAT BLANK(.3)  
  /CRITERIA MINEIGEN(1) ITERATE(25)  
  /EXTRACTION PC  
  /CRITERIA ITERATE(25)  
  /ROTATION VARIMAX  
  /METHOD=CORRELATION.

因子分析

[数据集1] D:\学术研究\066研究数据与论文撰写\01研究数据\AI智能体论文\信效度检验\AI智能体数据.sav

因子分析

公因子方差公因子方差, 表, 1 个级别的列标题和 1 个级别的行标题, 包含 3 列 23 行的表

|  |  |  |
| --- | --- | --- |
|  | 初始 | 提取 |
| AA1 | 1.000 | .720 |
| AA2 | 1.000 | .686 |
| AA3 | 1.000 | .660 |
| AA4 | 1.000 | .635 |
| AA5 | 1.000 | .691 |
| SE1 | 1.000 | .525 |
| SE2 | 1.000 | .614 |
| SE3 | 1.000 | .620 |
| SE4 | 1.000 | .692 |
| SE5 | 1.000 | .671 |
| DM1 | 1.000 | .634 |
| DM2 | 1.000 | .646 |
| DM3 | 1.000 | .645 |
| DM4 | 1.000 | .596 |
| DM5 | 1.000 | .661 |
| AB1 | 1.000 | .681 |
| AB2 | 1.000 | .660 |
| AB3 | 1.000 | .711 |
| AB4 | 1.000 | .637 |
| AB5 | 1.000 | .649 |
|  |  |  |
| --- | --- | --- |
| 提取方法：主成分分析法。 | | |
|  |  |  |

因子分析

总方差解释总方差解释, 表, 2 个级别的列标题和 1 个级别的行标题, 包含 10 列 24 行的表

|  |  |  |  |  |  |  |  |  |  |
| --- | --- | --- | --- | --- | --- | --- | --- | --- | --- |
| 成分 | 初始特征值 | | | 提取载荷平方和 | | | 旋转载荷平方和 | | |
|  | 总计 | 方差百分比 | 累积 % | 总计 | 方差百分比 | 累积 % | 总计 | 方差百分比 | 累积 % |
| 1 | 7.136 | 35.678 | 35.678 | 7.136 | 35.678 | 35.678 | 3.733 | 18.666 | 18.666 |
| 2 | 2.494 | 12.471 | 48.149 | 2.494 | 12.471 | 48.149 | 3.455 | 17.276 | 35.942 |
| 3 | 2.142 | 10.709 | 58.858 | 2.142 | 10.709 | 58.858 | 3.001 | 15.004 | 50.945 |
| 4 | 1.264 | 6.319 | 65.177 | 1.264 | 6.319 | 65.177 | 2.846 | 14.232 | 65.177 |
| 5 | .624 | 3.121 | 68.298 |  |  |  |  |  |  |
| 6 | .572 | 2.862 | 71.160 |  |  |  |  |  |  |
| 7 | .545 | 2.726 | 73.886 |  |  |  |  |  |  |
| 8 | .542 | 2.710 | 76.597 |  |  |  |  |  |  |
| 9 | .498 | 2.488 | 79.085 |  |  |  |  |  |  |
| 10 | .464 | 2.322 | 81.407 |  |  |  |  |  |  |
| 11 | .442 | 2.209 | 83.616 |  |  |  |  |  |  |
| 12 | .432 | 2.159 | 85.775 |  |  |  |  |  |  |
| 13 | .426 | 2.128 | 87.903 |  |  |  |  |  |  |
| 14 | .416 | 2.082 | 89.986 |  |  |  |  |  |  |
| 15 | .397 | 1.984 | 91.970 |  |  |  |  |  |  |
| 16 | .360 | 1.802 | 93.771 |  |  |  |  |  |  |
| 17 | .343 | 1.715 | 95.487 |  |  |  |  |  |  |
| 18 | .327 | 1.636 | 97.122 |  |  |  |  |  |  |
| 19 | .300 | 1.499 | 98.621 |  |  |  |  |  |  |
| 20 | .276 | 1.379 | 100.000 |  |  |  |  |  |  |
|  |  |  |  |  |  |  |  |  |  |
| --- | --- | --- | --- | --- | --- | --- | --- | --- | --- |
| 提取方法：主成分分析法。 | | | | | | | | | |
|  |  |  |  |  |  |  |  |  |  |

因子分析

成分矩阵^a^成分矩阵, 表, 2 个级别的列标题和 1 个级别的行标题, 包含 5 列 25 行的表

|  |  |  |  |  |
| --- | --- | --- | --- | --- |
|  | 成分 | | | |
|  | 1 | 2 | 3 | 4 |
| AA1 | .649 | -.365 | -.343 |  |
| AA2 | .630 | -.352 | -.389 |  |
| AA3 | .627 |  | -.343 |  |
| AA4 | .652 | -.313 |  |  |
| AA5 | .640 | -.383 | -.322 |  |
| SE1 | .513 | .411 |  |  |
| SE2 | .548 | .430 |  |  |
| SE3 | .541 | .433 |  | .310 |
| SE4 | .643 | .393 |  |  |
| SE5 | .637 | .391 |  |  |
| DM1 | .503 | -.342 | .371 | .355 |
| DM2 | .576 |  | .423 |  |
| DM3 | .577 | -.330 | .335 | .302 |
| DM4 | .650 | -.415 |  |  |
| DM5 | .482 | -.384 | .374 | .377 |
| AB1 | .570 |  | .421 | -.320 |
| AB2 | .573 |  | .452 |  |
| AB3 | .598 |  | .423 | -.324 |
| AB4 | .702 | .369 |  |  |
| AB5 | .582 | .322 | .303 | -.339 |
|  |  |  |  |  |
| --- | --- | --- | --- | --- |
| 提取方法：主成分分析法。 | | | | |
| a. 提取了 4 个成分。 | | | | |
|  |  |  |  |  |

因子分析

旋转后的成分矩阵^a^旋转后的成分矩阵, 表, 2 个级别的列标题和 1 个级别的行标题, 包含 5 列 25 行的表

|  |  |  |  |  |
| --- | --- | --- | --- | --- |
|  | 成分 | | | |
|  | 1 | 2 | 3 | 4 |
| AA1 | .817 |  |  |  |
| AA2 | .786 |  |  |  |
| AA3 | .780 |  |  |  |
| AA4 | .746 |  |  |  |
| AA5 | .796 |  |  |  |
| SE1 |  | .699 |  |  |
| SE2 |  | .758 |  |  |
| SE3 |  | .769 |  |  |
| SE4 |  | .782 |  |  |
| SE5 |  | .762 |  |  |
| DM1 |  |  |  | .772 |
| DM2 |  |  |  | .736 |
| DM3 |  |  |  | .748 |
| DM4 | .567 |  |  | .490 |
| DM5 |  |  |  | .795 |
| AB1 |  |  | .789 |  |
| AB2 |  |  | .765 |  |
| AB3 |  |  | .801 |  |
| AB4 |  | .557 | .519 |  |
| AB5 |  |  | .756 |  |
|  |  |  |  |  |
| --- | --- | --- | --- | --- |
| 提取方法：主成分分析法。   旋转方法：凯撒正态化最大方差法。 | | | | |
| a. 旋转在 6 次迭代后已收敛。 | | | | |
|  |  |  |  |  |

因子分析

成分转换矩阵成分转换矩阵, 表, 1 个级别的列标题和 1 个级别的行标题, 包含 5 列 7 行的表

|  |  |  |  |  |
| --- | --- | --- | --- | --- |
| 成分 | 1 | 2 | 3 | 4 |
| 1 | .569 | .520 | .469 | .431 |
| 2 | -.526 | .620 | .366 | -.453 |
| 3 | -.511 | -.383 | .563 | .524 |
| 4 | -.372 | .446 | -.573 | .578 |
|  |  |  |  |  |
| --- | --- | --- | --- | --- |
| 提取方法：主成分分析法。   旋转方法：凯撒正态化最大方差法。 | | | | |
|  |  |  |  |  |

因子分析

成分得分系数矩阵成分得分系数矩阵, 表, 2 个级别的列标题和 1 个级别的行标题, 包含 5 列 24 行的表

|  |  |  |  |  |
| --- | --- | --- | --- | --- |
|  | 成分 | | | |
|  | 1 | 2 | 3 | 4 |
| AA1 | .274 | -.058 | -.003 | -.077 |
| AA2 | .252 | -.014 | -.059 | -.047 |
| AA3 | .267 | -.053 | .018 | -.103 |
| AA4 | .236 | -.036 | -.006 | -.050 |
| AA5 | .261 | -.053 | -.018 | -.052 |
| SE1 | -.052 | .252 | -.058 | -.006 |
| SE2 | -.041 | .274 | -.075 | -.017 |
| SE3 | -.089 | .294 | -.097 | .044 |
| SE4 | -.033 | .272 | -.073 | .003 |
| SE5 | -.015 | .257 | -.055 | -.022 |
| DM1 | -.081 | .010 | -.081 | .346 |
| DM2 | -.070 | -.014 | -.003 | .300 |
| DM3 | -.053 | .007 | -.059 | .315 |
| DM4 | .129 | -.062 | -.009 | .126 |
| DM5 | -.081 | .006 | -.097 | .363 |
| AB1 | -.019 | -.078 | .334 | -.060 |
| AB2 | -.026 | -.084 | .316 | -.023 |
| AB3 | -.014 | -.081 | .336 | -.057 |
| AB4 | -.044 | .125 | .126 | -.002 |
| AB5 | .006 | -.051 | .319 | -.104 |
|  |  |  |  |  |
| --- | --- | --- | --- | --- |
| 提取方法：主成分分析法。   旋转方法：凯撒正态化最大方差法。 | | | | |
|  |  |  |  |  |

因子分析

成分得分协方差矩阵成分得分协方差矩阵, 表, 1 个级别的列标题和 1 个级别的行标题, 包含 5 列 7 行的表

|  |  |  |  |  |
| --- | --- | --- | --- | --- |
| 成分 | 1 | 2 | 3 | 4 |
| 1 | 1.000 | .000 | .000 | .000 |
| 2 | .000 | 1.000 | .000 | .000 |
| 3 | .000 | .000 | 1.000 | .000 |
| 4 | .000 | .000 | .000 | 1.000 |
|  |  |  |  |  |
| --- | --- | --- | --- | --- |
| 提取方法：主成分分析法。   旋转方法：凯撒正态化最大方差法。 | | | | |
|  |  |  |  |  |

IBM SPSS Web 报告

X

关于

使用以下对象创建： IBM SPSS Statistics 26

创建日期： 九月 06, 2026

文档版本： 原始保存的副本

保存日期：  九月 06, 2026

导航控件

内容 - 打开并关闭 Web 报告中的图表和表的列表

下一个 & 上一个 - 显示 Web 报告中的下一个或上一个表或图表

帮助 - 打开帮助

工具栏按钮

|  |  |
| --- | --- |
|  | 撤销 - 撤销文档中的最近一次更改。 |
|  | 编辑 - 打开表和图表的编辑器工具。某些编辑选项仅当您连接到因特网服务器时才可用。 |
|  | 保存 - 创建 Web 报告的包含已保存更改的新副本。 |
|  | 打印 - 打印当前对象（对于对象视图）和所有对象（对于页面视图）。 |
|  | 页面视图 - 将 Web 报告切换为在单个页面上显示所有表和图表。 |
|  | 对象视图 - 切换 Web 报告，以便每次显示一个表或图表。 |

连接至服务器

:   Web 报告与因特网服务器的连接状态将显示在 Web 报告的右上角。
:   打开 Web 报告无需因特网连接。 借助 Web 报告的已保存副本，您可以查看所有图表和表，并在未连接到因特网时具有一些受限编辑能力。
:   使 Web 报告连接到因特网服务器将对表和图表启用更多编辑功能。

- 如果作者在创建 Web 报告时指定了因特网服务器，那么 Web 报告将在打开后尝试连接到该服务器。
- 如果 Web 报告未连接到服务器，请单击服务器“状态消息”以打开用于重试连接、尝试连接到其他服务器或输入新的服务器地址的工具。
- 有关向因特网服务器添加增强控件的信息，请转到https://developer.ibm.com/predictiveanalytics.
- 如果指定新服务器连接，那么首选格式为 http://xxx.xxx.xxx.xxx:xxxx。

编辑表

|  |  |
| --- | --- |
| 此功能的某些部分仅当在连接到因特网服务器时才可用。 | |
|  | 创建图表 - 根据表中的选定单元格创建图表。 |
|  | 旋转和排序 - 对表进行转置、排序和旋转。 |
|  | 背景色 - 选定单元格的背景色。 |
|  | 文本颜色和样式 - 字体颜色、样式和大小。 |
|  | 编号格式 - 字体颜色、样式和大小。 |

编辑图表

|  |  |
| --- | --- |
| 此功能 仅当在连接到因特网服务器时才可用。 | |
|  | 图表大小 - 更改图表的高度和宽度 |
|  | 背景色 - 选定对象的背景色。 |
|  | 边框和线条样式 - 线条或边框的颜色和厚度。 |
|  | 文本颜色和样式 - 字体颜色、样式和大小。 |
|  | 编号格式 - 字体颜色、样式和大小。 |
|  | 轴属性 - 更改标度并显示轴标题和刻度标记。 |

服务器连接

X
  
已保存的服务器连接  
   
  

  
  
状态

添加图表

旋转和排序

图表大小   
  

|  |  |  |
| --- | --- | --- |
|  |  |  |
|  |  |  |
| 锁定宽高比 | | |

背景   

|  |  |  |  |  |  |
| --- | --- | --- | --- | --- | --- |
|  | |  | |  | |
|  |  |  |  |  |  |
|  |  |  |  |  |  |
|  |  |  |  |  |  |

线条和边框   

|  |  |  |  |  |  |
| --- | --- | --- | --- | --- | --- |
|  | |  | |  | |
|  |  |  |  |  |  |
|  |  |  |  |  |  |
|  |  |  |  |  |  |

文本格式   

|  |  |  |  |  |  |
| --- | --- | --- | --- | --- | --- |
|  | |  | |  | |
|  |  |  |  |  |  |
|  |  |  |  |  |  |
|  |  |  |  |  |  |

  

|  |  |  |
| --- | --- | --- |
|  |  |  |

  

|  |  |  |  |
| --- | --- | --- | --- |
|  |  |  | 字体系列  Adobe Devanagari Adobe 宋体 Std L Adobe 黑体 Std R Agency FB Algerian Arial Arial Black Arial Narrow Arial Rounded MT Bold Arial Unicode MS Bahnschrift Baskerville Old Face Bauhaus 93 Bell MT Berlin Sans FB Berlin Sans FB Demi Bernard MT Condensed Blackadder ITC Bodoni MT Bodoni MT Black Bodoni MT Condensed Bodoni MT Poster Compressed Book Antiqua Bookman Old Style Bookshelf Symbol 7 Bradley Hand ITC Britannic Bold Broadway Brush Script MT Calibri Calibri Light Californian FB Calisto MT Cambria Cambria Math Candara Candara Light Castellar Centaur Century Century Gothic Century Schoolbook Chiller Colonna MT Comic Sans MS Consolas Constantia Cooper Black Copperplate Gothic Bold Copperplate Gothic Light Corbel Corbel Light Courier New Curlz MT Dialog DialogInput Dubai Dubai Light Dubai Medium Ebrima Edwardian Script ITC Elephant Engravers MT Eras Bold ITC Eras Demi ITC Eras Light ITC Eras Medium ITC Euclid Euclid Extra Euclid Fraktur Euclid Math One Euclid Math Two Euclid Symbol Felix Titling Fences Footlight MT Light Forte Franklin Gothic Book Franklin Gothic Demi Franklin Gothic Demi Cond Franklin Gothic Heavy Franklin Gothic Medium Franklin Gothic Medium Cond Freestyle Script French Script MT Gabriola Gadugi Garamond Georgia Gigi Gill Sans MT Gill Sans MT Condensed Gill Sans MT Ext Condensed Bold Gill Sans Ultra Bold Gill Sans Ultra Bold Condensed Gloucester MT Extra Condensed Goudy Old Style Goudy Stout Haettenschweiler Harlow Solid Italic Harrington HelvNeue Roman for IBM High Tower Text Impact Imprint MT Shadow Informal Roman Ink Free Javanese Text Jokerman Juice ITC Kristen ITC Kunstler Script Leelawadee Leelawadee UI Leelawadee UI Semilight Lucida Bright Lucida Calligraphy Lucida Console Lucida Fax Lucida Handwriting Lucida Sans Lucida Sans Typewriter Lucida Sans Unicode Magneto Maiandra GD Malgun Gothic Malgun Gothic Semilight Marlett Matura MT Script Capitals Microsoft Himalaya Microsoft JhengHei Microsoft JhengHei Light Microsoft JhengHei UI Microsoft JhengHei UI Light Microsoft New Tai Lue Microsoft PhagsPa Microsoft Sans Serif Microsoft Tai Le Microsoft Uighur Microsoft YaHei UI Microsoft YaHei UI Light Microsoft Yi Baiti MingLiU-ExtB MingLiU\_HKSCS-ExtB Mistral Modern No. 20 Mongolian Baiti Monospaced Monotype Corsiva MS Gothic MS Outlook MS PGothic MS Reference Sans Serif MS Reference Specialty MS UI Gothic MT Extra MT Extra Tiger MV Boli Myanmar Text Niagara Engraved Niagara Solid Nirmala Text Nirmala Text Semilight Nirmala UI Nirmala UI Semilight Noto Sans SC Noto Serif SC OCR A Extended Old English Text MT Onyx Palace Script MT Palatino Linotype Papyrus Parchment Perpetua Perpetua Titling MT Playbill PMingLiU-ExtB Poor Richard Pristina Rage Italic Ravie Rockwell Rockwell Condensed Rockwell Extra Bold Sans Serif Collection SansSerif Script MT Bold Segoe Fluent Icons Segoe MDL2 Assets Segoe Print Segoe Script Segoe UI Segoe UI Black Segoe UI Emoji Segoe UI Historic Segoe UI Light Segoe UI Semibold Segoe UI Semilight Segoe UI Symbol Segoe UI Variable Serif Showcard Gothic SimSun-ExtB SimSun-ExtG Sitka Text Snap ITC Stencil Sylfaen Symbol Symbol Tiger Symbol Tiger Expert Tahoma Tempus Sans ITC Tiger Tiger Expert Times New Roman Trebuchet MS Tw Cen MT Tw Cen MT Condensed Tw Cen MT Condensed Extra Bold Verdana Viner Hand ITC Vivaldi Vladimir Script Webdings Wide Latin Wingdings Wingdings 2 Wingdings 3 Yu Gothic Yu Gothic Light Yu Gothic Medium Yu Gothic UI Yu Gothic UI Light Yu Gothic UI Semibold Yu Gothic UI Semilight ZWAdobeF 仿宋 华文中宋 华文仿宋 华文宋体 华文彩云 华文新魏 华文楷体 华文琥珀 华文细黑 华文行楷 华文隶书 宋体 幼圆 微软雅黑 微软雅黑 Light 新宋体 方正姚体 方正舒体 楷体 汉仪中黑 197 等线 等线 Light 隶书 黑体 |

编号格式   
  

|  |  |  |
| --- | --- | --- |
| 0.00 |  |  |

轴选项   
  

|  |  |  |  |
| --- | --- | --- | --- |
|  |  |  |  |
|  |  |  |  |
| 显示轴标题 | | | |
| 显示刻度标记 | | | |
