# Supplementary material for: How AI threat perception relates to post-adoptive AI agent engagement: the dual paths of self-efficacy and defensive motivation
Source: Front Psychol. 2026 Oct 22;17:1887571. doi: 10.3389/fpsyg.2026.1887571 (PMC13735560; doi:10.3389/fpsyg.2026.1887571)
Supplement: Supplementary file 1 [file Data_Sheet_1.zip › log+Questionnaire/002confirmatory factor analysis.AmosOutput]

信效度检验V2.amw


#### D:\学术研究\066研究数据与论文撰写\01研究数据\AI智能体论文\信效度检验\信效度检验V2.amw

##### Analysis Summary

##### Date and Time

Date: 2026年2月21日

Time: 9:45:59

##### Title

信效度检验v2: 2026年2月21日 9:45

##### Groups

##### Group number 1 (Group number 1)

##### Notes for Group (Group number 1)

The model is recursive.

Sample size = 387

##### Variable Summary (Group number 1)

##### Your model contains the following variables (Group number 1)

Observed, endogenous variables

AA1

AA2

AA4

AA5

SE1

SE2

SE3

SE4

SE5

DM1

DM2

DM3

DM5

AB1

AB2

AB3

AB5

AA3

Unobserved, exogenous variables

AI焦虑

e1

e2

e4

e5

自我效能

e6

e7

e8

e9

e10

防御性动机

e11

e12

e13

e15

采纳行为

e16

e17

e18

e20

e3

##### Variable counts (Group number 1)

|  |  |
| --- | --- |
| Number of variables in your model: | 40 |
| Number of observed variables: | 18 |
| Number of unobserved variables: | 22 |
| Number of exogenous variables: | 22 |
| Number of endogenous variables: | 18 |

##### Parameter Summary (Group number 1)

|  | Weights | Covariances | Variances | Means | Intercepts | Total |
| --- | --- | --- | --- | --- | --- | --- |
| Fixed | 22 | 0 | 0 | 0 | 0 | 22 |
| Labeled | 0 | 0 | 0 | 0 | 0 | 0 |
| Unlabeled | 14 | 6 | 22 | 0 | 0 | 42 |
| Total | 36 | 6 | 22 | 0 | 0 | 64 |

##### Sample Moments (Group number 1)

##### Sample Covariances (Group number 1)

|  | AA3 | AB5 | AB3 | AB2 | AB1 | DM5 | DM3 | DM2 | DM1 | SE5 | SE4 | SE3 | SE2 | SE1 | AA5 | AA4 | AA2 | AA1 |
| --- | --- | --- | --- | --- | --- | --- | --- | --- | --- | --- | --- | --- | --- | --- | --- | --- | --- | --- |
| AA3 | 3.300 |  |  |  |  |  |  |  |  |  |  |  |  |  |  |  |  |  |
| AB5 | .704 | 3.347 |  |  |  |  |  |  |  |  |  |  |  |  |  |  |  |  |
| AB3 | .778 | 1.956 | 3.304 |  |  |  |  |  |  |  |  |  |  |  |  |  |  |  |
| AB2 | .784 | 1.743 | 1.972 | 3.325 |  |  |  |  |  |  |  |  |  |  |  |  |  |  |
| AB1 | .627 | 1.832 | 2.025 | 1.853 | 3.306 |  |  |  |  |  |  |  |  |  |  |  |  |  |
| DM5 | .639 | .481 | .855 | .817 | .736 | 3.273 |  |  |  |  |  |  |  |  |  |  |  |  |
| DM3 | .939 | .778 | 1.023 | .997 | .912 | 1.764 | 3.210 |  |  |  |  |  |  |  |  |  |  |  |
| DM2 | .826 | .948 | 1.131 | 1.225 | 1.078 | 1.707 | 1.754 | 3.238 |  |  |  |  |  |  |  |  |  |  |
| DM1 | .750 | .702 | .823 | .958 | .811 | 1.696 | 1.776 | 1.720 | 3.380 |  |  |  |  |  |  |  |  |  |
| SE5 | 1.061 | 1.098 | 1.034 | .910 | .951 | .486 | .570 | .579 | .458 | 3.157 |  |  |  |  |  |  |  |  |
| SE4 | 1.182 | 1.160 | .945 | .964 | 1.000 | .444 | .741 | .670 | .552 | 2.033 | 3.346 |  |  |  |  |  |  |  |
| SE3 | .730 | 1.106 | .875 | .739 | .719 | .377 | .506 | .569 | .580 | 1.793 | 1.845 | 3.274 |  |  |  |  |  |  |
| SE2 | .892 | 1.006 | .866 | .733 | .883 | .220 | .493 | .483 | .360 | 1.811 | 2.077 | 1.645 | 3.481 |  |  |  |  |  |
| SE1 | .675 | .803 | .880 | .805 | .870 | .304 | .514 | .477 | .233 | 1.631 | 1.598 | 1.557 | 1.521 | 3.248 |  |  |  |  |
| AA5 | 1.882 | .707 | .691 | .511 | .602 | .836 | 1.059 | .933 | .926 | .974 | .847 | .715 | .828 | .722 | 3.183 |  |  |  |
| AA4 | 1.695 | .781 | .737 | .719 | .769 | .897 | 1.057 | .970 | .873 | .967 | 1.099 | .753 | .949 | .860 | 1.996 | 3.273 |  |  |
| AA2 | 1.911 | .599 | .596 | .455 | .525 | .818 | 1.057 | .765 | .942 | 1.153 | 1.181 | .649 | .935 | .718 | 1.915 | 1.968 | 3.233 |  |
| AA1 | 2.127 | .761 | .703 | .701 | .583 | .889 | 1.009 | .973 | .804 | 1.110 | .978 | .771 | .716 | .757 | 2.053 | 1.951 | 2.066 | 3.264 |

Condition number = 21.885

Eigenvalues

20.597 7.383 6.997 4.168 2.055 1.901 1.783 1.639 1.594 1.490 1.419 1.376 1.312 1.224 1.175 1.092 .996 .941

Determinant of sample covariance matrix = 453293.145

##### Sample Correlations (Group number 1)

|  | AA3 | AB5 | AB3 | AB2 | AB1 | DM5 | DM3 | DM2 | DM1 | SE5 | SE4 | SE3 | SE2 | SE1 | AA5 | AA4 | AA2 | AA1 |
| --- | --- | --- | --- | --- | --- | --- | --- | --- | --- | --- | --- | --- | --- | --- | --- | --- | --- | --- |
| AA3 | 1.000 |  |  |  |  |  |  |  |  |  |  |  |  |  |  |  |  |  |
| AB5 | .212 | 1.000 |  |  |  |  |  |  |  |  |  |  |  |  |  |  |  |  |
| AB3 | .236 | .588 | 1.000 |  |  |  |  |  |  |  |  |  |  |  |  |  |  |  |
| AB2 | .237 | .523 | .595 | 1.000 |  |  |  |  |  |  |  |  |  |  |  |  |  |  |
| AB1 | .190 | .551 | .613 | .559 | 1.000 |  |  |  |  |  |  |  |  |  |  |  |  |  |
| DM5 | .194 | .145 | .260 | .248 | .224 | 1.000 |  |  |  |  |  |  |  |  |  |  |  |  |
| DM3 | .288 | .237 | .314 | .305 | .280 | .544 | 1.000 |  |  |  |  |  |  |  |  |  |  |  |
| DM2 | .253 | .288 | .346 | .373 | .330 | .524 | .544 | 1.000 |  |  |  |  |  |  |  |  |  |  |
| DM1 | .224 | .209 | .246 | .286 | .243 | .510 | .539 | .520 | 1.000 |  |  |  |  |  |  |  |  |  |
| SE5 | .329 | .338 | .320 | .281 | .294 | .151 | .179 | .181 | .140 | 1.000 |  |  |  |  |  |  |  |  |
| SE4 | .356 | .347 | .284 | .289 | .301 | .134 | .226 | .203 | .164 | .626 | 1.000 |  |  |  |  |  |  |  |
| SE3 | .222 | .334 | .266 | .224 | .218 | .115 | .156 | .175 | .174 | .558 | .557 | 1.000 |  |  |  |  |  |  |
| SE2 | .263 | .295 | .255 | .215 | .260 | .065 | .147 | .144 | .105 | .546 | .608 | .487 | 1.000 |  |  |  |  |  |
| SE1 | .206 | .244 | .269 | .245 | .266 | .093 | .159 | .147 | .070 | .509 | .485 | .477 | .452 | 1.000 |  |  |  |  |
| AA5 | .581 | .217 | .213 | .157 | .186 | .259 | .331 | .291 | .282 | .307 | .260 | .222 | .249 | .225 | 1.000 |  |  |  |
| AA4 | .516 | .236 | .224 | .218 | .234 | .274 | .326 | .298 | .262 | .301 | .332 | .230 | .281 | .264 | .618 | 1.000 |  |  |
| AA2 | .585 | .182 | .182 | .139 | .161 | .252 | .328 | .237 | .285 | .361 | .359 | .199 | .279 | .222 | .597 | .605 | 1.000 |  |
| AA1 | .648 | .230 | .214 | .213 | .178 | .272 | .312 | .299 | .242 | .346 | .296 | .236 | .213 | .233 | .637 | .597 | .636 | 1.000 |

Condition number = 21.908

Eigenvalues

6.277 2.245 2.130 1.264 .624 .570 .542 .501 .483 .457 .430 .419 .397 .376 .360 .332 .307 .287

##### Models

##### Default model (Default model)

##### Notes for Model (Default model)

##### Computation of degrees of freedom (Default model)

|  |  |
| --- | --- |
| Number of distinct sample moments: | 171 |
| Number of distinct parameters to be estimated: | 42 |
| Degrees of freedom (171 - 42): | 129 |

##### Result (Default model)

Minimum was achieved

Chi-square = 133.951

Degrees of freedom = 129

Probability level = .365

##### Group number 1 (Group number 1 - Default model)

##### Estimates (Group number 1 - Default model)

##### Scalar Estimates (Group number 1 - Default model)

##### Maximum Likelihood Estimates

##### Regression Weights: (Group number 1 - Default model)

|  |  |  | Estimate | S.E. | C.R. | P | Label |
| --- | --- | --- | --- | --- | --- | --- | --- |
| AA1 | <--- | AI焦虑 | 1.000 |  |  |  |  |
| AA2 | <--- | AI焦虑 | .952 | .057 | 16.762 | \*\*\* | par\_1 |
| AA4 | <--- | AI焦虑 | .921 | .058 | 15.935 | \*\*\* | par\_2 |
| AA5 | <--- | AI焦虑 | .945 | .056 | 16.766 | \*\*\* | par\_3 |
| SE1 | <--- | 自我效能 | 1.000 |  |  |  |  |
| SE2 | <--- | 自我效能 | 1.165 | .101 | 11.512 | \*\*\* | par\_4 |
| SE3 | <--- | 自我效能 | 1.099 | .097 | 11.277 | \*\*\* | par\_5 |
| SE4 | <--- | 自我效能 | 1.284 | .103 | 12.504 | \*\*\* | par\_6 |
| SE5 | <--- | 自我效能 | 1.222 | .099 | 12.338 | \*\*\* | par\_7 |
| DM1 | <--- | 防御性动机 | 1.000 |  |  |  |  |
| DM2 | <--- | 防御性动机 | 1.026 | .082 | 12.470 | \*\*\* | par\_8 |
| DM3 | <--- | 防御性动机 | 1.051 | .083 | 12.738 | \*\*\* | par\_9 |
| DM5 | <--- | 防御性动机 | .984 | .082 | 12.001 | \*\*\* | par\_10 |
| AB1 | <--- | 采纳行为 | 1.000 |  |  |  |  |
| AB2 | <--- | 采纳行为 | .975 | .071 | 13.751 | \*\*\* | par\_11 |
| AB3 | <--- | 采纳行为 | 1.064 | .071 | 14.925 | \*\*\* | par\_12 |
| AB5 | <--- | 采纳行为 | .964 | .071 | 13.543 | \*\*\* | par\_13 |
| AA3 | <--- | AI焦虑 | .920 | .058 | 15.833 | \*\*\* | par\_20 |

##### Standardized Regression Weights: (Group number 1 - Default model)

|  |  |  | Estimate |
| --- | --- | --- | --- |
| AA1 | <--- | AI焦虑 | .818 |
| AA2 | <--- | AI焦虑 | .782 |
| AA4 | <--- | AI焦虑 | .752 |
| AA5 | <--- | AI焦虑 | .782 |
| SE1 | <--- | 自我效能 | .636 |
| SE2 | <--- | 自我效能 | .716 |
| SE3 | <--- | 自我效能 | .696 |
| SE4 | <--- | 自我效能 | .805 |
| SE5 | <--- | 自我效能 | .788 |
| DM1 | <--- | 防御性动机 | .705 |
| DM2 | <--- | 防御性动机 | .739 |
| DM3 | <--- | 防御性动机 | .761 |
| DM5 | <--- | 防御性动机 | .705 |
| AB1 | <--- | 采纳行为 | .758 |
| AB2 | <--- | 采纳行为 | .737 |
| AB3 | <--- | 采纳行为 | .806 |
| AB5 | <--- | 采纳行为 | .725 |
| AA3 | <--- | AI焦虑 | .748 |

##### Covariances: (Group number 1 - Default model)

|  |  |  | Estimate | S.E. | C.R. | P | Label |
| --- | --- | --- | --- | --- | --- | --- | --- |
| AI焦虑 | <--> | 自我效能 | .823 | .123 | 6.711 | \*\*\* | par\_14 |
| AI焦虑 | <--> | 防御性动机 | .939 | .137 | 6.845 | \*\*\* | par\_15 |
| AI焦虑 | <--> | 采纳行为 | .693 | .130 | 5.315 | \*\*\* | par\_16 |
| 自我效能 | <--> | 防御性动机 | .424 | .099 | 4.281 | \*\*\* | par\_17 |
| 自我效能 | <--> | 采纳行为 | .787 | .119 | 6.611 | \*\*\* | par\_18 |
| 防御性动机 | <--> | 采纳行为 | .888 | .133 | 6.690 | \*\*\* | par\_19 |

##### Correlations: (Group number 1 - Default model)

|  |  |  | Estimate |
| --- | --- | --- | --- |
| AI焦虑 | <--> | 自我效能 | .486 |
| AI焦虑 | <--> | 防御性动机 | .490 |
| AI焦虑 | <--> | 采纳行为 | .341 |
| 自我效能 | <--> | 防御性动机 | .285 |
| 自我效能 | <--> | 采纳行为 | .498 |
| 防御性动机 | <--> | 采纳行为 | .497 |

##### Variances: (Group number 1 - Default model)

|  |  |  | Estimate | S.E. | C.R. | P | Label |
| --- | --- | --- | --- | --- | --- | --- | --- |
| AI焦虑 |  |  | 2.181 | .232 | 9.396 | \*\*\* | par\_21 |
| 自我效能 |  |  | 1.314 | .200 | 6.568 | \*\*\* | par\_22 |
| 防御性动机 |  |  | 1.682 | .230 | 7.319 | \*\*\* | par\_23 |
| 采纳行为 |  |  | 1.897 | .231 | 8.198 | \*\*\* | par\_24 |
| e1 |  |  | 1.082 | .104 | 10.394 | \*\*\* | par\_25 |
| e2 |  |  | 1.255 | .112 | 11.163 | \*\*\* | par\_26 |
| e4 |  |  | 1.424 | .122 | 11.651 | \*\*\* | par\_27 |
| e5 |  |  | 1.235 | .111 | 11.160 | \*\*\* | par\_28 |
| e6 |  |  | 1.933 | .155 | 12.448 | \*\*\* | par\_29 |
| e7 |  |  | 1.699 | .146 | 11.644 | \*\*\* | par\_30 |
| e8 |  |  | 1.686 | .142 | 11.880 | \*\*\* | par\_31 |
| e9 |  |  | 1.178 | .119 | 9.912 | \*\*\* | par\_32 |
| e10 |  |  | 1.194 | .116 | 10.337 | \*\*\* | par\_33 |
| e11 |  |  | 1.698 | .153 | 11.080 | \*\*\* | par\_34 |
| e12 |  |  | 1.468 | .140 | 10.452 | \*\*\* | par\_35 |
| e13 |  |  | 1.352 | .136 | 9.966 | \*\*\* | par\_36 |
| e15 |  |  | 1.646 | .148 | 11.086 | \*\*\* | par\_37 |
| e16 |  |  | 1.409 | .132 | 10.656 | \*\*\* | par\_38 |
| e17 |  |  | 1.521 | .138 | 11.053 | \*\*\* | par\_39 |
| e18 |  |  | 1.155 | .123 | 9.418 | \*\*\* | par\_40 |
| e20 |  |  | 1.586 | .141 | 11.240 | \*\*\* | par\_41 |
| e3 |  |  | 1.454 | .124 | 11.703 | \*\*\* | par\_42 |

##### Squared Multiple Correlations: (Group number 1 - Default model)

|  |  |  | Estimate |
| --- | --- | --- | --- |
| AA3 |  |  | .559 |
| AB5 |  |  | .526 |
| AB3 |  |  | .650 |
| AB2 |  |  | .543 |
| AB1 |  |  | .574 |
| DM5 |  |  | .497 |
| DM3 |  |  | .579 |
| DM2 |  |  | .547 |
| DM1 |  |  | .498 |
| SE5 |  |  | .622 |
| SE4 |  |  | .648 |
| SE3 |  |  | .485 |
| SE2 |  |  | .512 |
| SE1 |  |  | .405 |
| AA5 |  |  | .612 |
| AA4 |  |  | .565 |
| AA2 |  |  | .612 |
| AA1 |  |  | .668 |

##### Matrices (Group number 1 - Default model)

##### Implied (for all variables) Covariances (Group number 1 - Default model)

|  | 采纳行为 | 防御性动机 | 自我效能 | AI焦虑 | AA3 | AB5 | AB3 | AB2 | AB1 | DM5 | DM3 | DM2 | DM1 | SE5 | SE4 | SE3 | SE2 | SE1 | AA5 | AA4 | AA2 | AA1 |
| --- | --- | --- | --- | --- | --- | --- | --- | --- | --- | --- | --- | --- | --- | --- | --- | --- | --- | --- | --- | --- | --- | --- |
| 采纳行为 | 1.897 |  |  |  |  |  |  |  |  |  |  |  |  |  |  |  |  |  |  |  |  |  |
| 防御性动机 | .888 | 1.682 |  |  |  |  |  |  |  |  |  |  |  |  |  |  |  |  |  |  |  |  |
| 自我效能 | .787 | .424 | 1.314 |  |  |  |  |  |  |  |  |  |  |  |  |  |  |  |  |  |  |  |
| AI焦虑 | .693 | .939 | .823 | 2.181 |  |  |  |  |  |  |  |  |  |  |  |  |  |  |  |  |  |  |
| AA3 | .638 | .864 | .757 | 2.007 | 3.300 |  |  |  |  |  |  |  |  |  |  |  |  |  |  |  |  |  |
| AB5 | 1.828 | .856 | .758 | .668 | .615 | 3.347 |  |  |  |  |  |  |  |  |  |  |  |  |  |  |  |  |
| AB3 | 2.019 | .945 | .838 | .738 | .679 | 1.945 | 3.304 |  |  |  |  |  |  |  |  |  |  |  |  |  |  |  |
| AB2 | 1.850 | .866 | .768 | .676 | .622 | 1.782 | 1.969 | 3.325 |  |  |  |  |  |  |  |  |  |  |  |  |  |  |
| AB1 | 1.897 | .888 | .787 | .693 | .638 | 1.828 | 2.019 | 1.850 | 3.306 |  |  |  |  |  |  |  |  |  |  |  |  |  |
| DM5 | .873 | 1.654 | .417 | .924 | .850 | .841 | .930 | .852 | .873 | 3.273 |  |  |  |  |  |  |  |  |  |  |  |  |
| DM3 | .933 | 1.768 | .446 | .987 | .908 | .899 | .993 | .910 | .933 | 1.739 | 3.210 |  |  |  |  |  |  |  |  |  |  |  |
| DM2 | .911 | 1.725 | .435 | .963 | .886 | .878 | .970 | .888 | .911 | 1.697 | 1.814 | 3.238 |  |  |  |  |  |  |  |  |  |  |
| DM1 | .888 | 1.682 | .424 | .939 | .864 | .856 | .945 | .866 | .888 | 1.654 | 1.768 | 1.725 | 3.380 |  |  |  |  |  |  |  |  |  |
| SE5 | .962 | .518 | 1.606 | 1.006 | .925 | .927 | 1.024 | .938 | .962 | .509 | .544 | .531 | .518 | 3.157 |  |  |  |  |  |  |  |  |
| SE4 | 1.011 | .544 | 1.688 | 1.057 | .973 | .974 | 1.076 | .986 | 1.011 | .535 | .572 | .559 | .544 | 2.063 | 3.346 |  |  |  |  |  |  |  |
| SE3 | .865 | .466 | 1.444 | .905 | .832 | .833 | .921 | .843 | .865 | .458 | .490 | .478 | .466 | 1.765 | 1.855 | 3.274 |  |  |  |  |  |  |
| SE2 | .917 | .494 | 1.531 | .959 | .882 | .883 | .976 | .894 | .917 | .485 | .519 | .506 | .494 | 1.870 | 1.966 | 1.682 | 3.481 |  |  |  |  |  |
| SE1 | .787 | .424 | 1.314 | .823 | .757 | .758 | .838 | .768 | .787 | .417 | .446 | .435 | .424 | 1.606 | 1.688 | 1.444 | 1.531 | 3.248 |  |  |  |  |
| AA5 | .655 | .887 | .778 | 2.062 | 1.896 | .632 | .698 | .639 | .655 | .873 | .933 | .910 | .887 | .951 | .999 | .855 | .906 | .778 | 3.183 |  |  |  |
| AA4 | .639 | .864 | .758 | 2.009 | 1.848 | .615 | .680 | .623 | .639 | .850 | .909 | .887 | .864 | .926 | .974 | .833 | .883 | .758 | 1.898 | 3.273 |  |  |
| AA2 | .660 | .894 | .784 | 2.077 | 1.911 | .636 | .703 | .644 | .660 | .879 | .940 | .917 | .894 | .958 | 1.007 | .861 | .913 | .784 | 1.963 | 1.913 | 3.233 |  |
| AA1 | .693 | .939 | .823 | 2.181 | 2.007 | .668 | .738 | .676 | .693 | .924 | .987 | .963 | .939 | 1.006 | 1.057 | .905 | .959 | .823 | 2.062 | 2.009 | 2.077 | 3.264 |

##### Implied (for all variables) Correlations (Group number 1 - Default model)

|  | 采纳行为 | 防御性动机 | 自我效能 | AI焦虑 | AA3 | AB5 | AB3 | AB2 | AB1 | DM5 | DM3 | DM2 | DM1 | SE5 | SE4 | SE3 | SE2 | SE1 | AA5 | AA4 | AA2 | AA1 |
| --- | --- | --- | --- | --- | --- | --- | --- | --- | --- | --- | --- | --- | --- | --- | --- | --- | --- | --- | --- | --- | --- | --- |
| 采纳行为 | 1.000 |  |  |  |  |  |  |  |  |  |  |  |  |  |  |  |  |  |  |  |  |  |
| 防御性动机 | .497 | 1.000 |  |  |  |  |  |  |  |  |  |  |  |  |  |  |  |  |  |  |  |  |
| 自我效能 | .498 | .285 | 1.000 |  |  |  |  |  |  |  |  |  |  |  |  |  |  |  |  |  |  |  |
| AI焦虑 | .341 | .490 | .486 | 1.000 |  |  |  |  |  |  |  |  |  |  |  |  |  |  |  |  |  |  |
| AA3 | .255 | .367 | .364 | .748 | 1.000 |  |  |  |  |  |  |  |  |  |  |  |  |  |  |  |  |  |
| AB5 | .725 | .361 | .362 | .247 | .185 | 1.000 |  |  |  |  |  |  |  |  |  |  |  |  |  |  |  |  |
| AB3 | .806 | .401 | .402 | .275 | .206 | .585 | 1.000 |  |  |  |  |  |  |  |  |  |  |  |  |  |  |  |
| AB2 | .737 | .366 | .367 | .251 | .188 | .534 | .594 | 1.000 |  |  |  |  |  |  |  |  |  |  |  |  |  |  |
| AB1 | .758 | .377 | .378 | .258 | .193 | .549 | .611 | .558 | 1.000 |  |  |  |  |  |  |  |  |  |  |  |  |  |
| DM5 | .350 | .705 | .201 | .346 | .258 | .254 | .283 | .258 | .265 | 1.000 |  |  |  |  |  |  |  |  |  |  |  |  |
| DM3 | .378 | .761 | .217 | .373 | .279 | .274 | .305 | .279 | .287 | .536 | 1.000 |  |  |  |  |  |  |  |  |  |  |  |
| DM2 | .368 | .739 | .211 | .362 | .271 | .267 | .296 | .271 | .278 | .521 | .563 | 1.000 |  |  |  |  |  |  |  |  |  |  |
| DM1 | .351 | .705 | .201 | .346 | .259 | .254 | .283 | .258 | .266 | .497 | .537 | .522 | 1.000 |  |  |  |  |  |  |  |  |  |
| SE5 | .393 | .225 | .788 | .383 | .287 | .285 | .317 | .289 | .298 | .158 | .171 | .166 | .159 | 1.000 |  |  |  |  |  |  |  |  |
| SE4 | .401 | .229 | .805 | .391 | .293 | .291 | .324 | .296 | .304 | .162 | .175 | .170 | .162 | .635 | 1.000 |  |  |  |  |  |  |  |
| SE3 | .347 | .199 | .696 | .339 | .253 | .252 | .280 | .256 | .263 | .140 | .151 | .147 | .140 | .549 | .561 | 1.000 |  |  |  |  |  |  |
| SE2 | .357 | .204 | .716 | .348 | .260 | .259 | .288 | .263 | .270 | .144 | .155 | .151 | .144 | .564 | .576 | .498 | 1.000 |  |  |  |  |  |
| SE1 | .317 | .181 | .636 | .309 | .231 | .230 | .256 | .234 | .240 | .128 | .138 | .134 | .128 | .502 | .512 | .443 | .455 | 1.000 |  |  |  |  |
| AA5 | .267 | .384 | .380 | .782 | .585 | .193 | .215 | .196 | .202 | .270 | .292 | .284 | .271 | .300 | .306 | .265 | .272 | .242 | 1.000 |  |  |  |
| AA4 | .256 | .368 | .365 | .752 | .562 | .186 | .207 | .189 | .194 | .260 | .280 | .272 | .260 | .288 | .294 | .254 | .262 | .232 | .588 | 1.000 |  |  |
| AA2 | .267 | .383 | .380 | .782 | .585 | .193 | .215 | .196 | .202 | .270 | .292 | .283 | .270 | .300 | .306 | .265 | .272 | .242 | .612 | .588 | 1.000 |  |
| AA1 | .279 | .401 | .397 | .818 | .611 | .202 | .225 | .205 | .211 | .283 | .305 | .296 | .283 | .313 | .320 | .277 | .284 | .253 | .640 | .615 | .639 | 1.000 |

##### Modification Indices (Group number 1 - Default model)

##### Covariances: (Group number 1 - Default model)

|  |  |  | M.I. | Par Change |
| --- | --- | --- | --- | --- |
| e20 | <--> | 防御性动机 | 4.035 | -.171 |
| e15 | <--> | e20 | 5.071 | -.221 |
| e12 | <--> | 采纳行为 | 5.611 | .206 |
| e8 | <--> | e20 | 4.448 | .204 |
| e5 | <--> | e9 | 5.898 | -.188 |
| e4 | <--> | e3 | 5.727 | -.201 |
| e2 | <--> | 采纳行为 | 4.157 | -.162 |
| e2 | <--> | e12 | 4.644 | -.183 |
| e2 | <--> | e8 | 4.736 | -.189 |
| e1 | <--> | e3 | 5.035 | .171 |
| e1 | <--> | e7 | 5.385 | -.195 |

##### Variances: (Group number 1 - Default model)

|  |  |  | M.I. | Par Change |
| --- | --- | --- | --- | --- |

##### Regression Weights: (Group number 1 - Default model)

|  |  |  | M.I. | Par Change |
| --- | --- | --- | --- | --- |
| AB5 | <--- | DM5 | 5.427 | -.090 |
| AB5 | <--- | SE3 | 7.009 | .102 |
| DM5 | <--- | AB5 | 6.026 | -.096 |
| DM2 | <--- | 采纳行为 | 4.277 | .112 |
| DM2 | <--- | AB2 | 5.767 | .091 |
| SE3 | <--- | AA2 | 5.018 | -.088 |
| AA5 | <--- | SE4 | 4.070 | -.069 |
| AA1 | <--- | SE2 | 4.500 | -.068 |

##### Minimization History (Default model)

| Iteration |  | Negative eigenvalues | Condition # | Smallest eigenvalue | Diameter | F | NTries | Ratio |
| --- | --- | --- | --- | --- | --- | --- | --- | --- |
| 0 | e | 9 |  | -.650 | 9999.000 | 3215.822 | 0 | 9999.000 |
| 1 | e\* | 4 |  | -.198 | 3.383 | 1063.557 | 20 | .385 |
| 2 | e\* | 1 |  | -.030 | .991 | 548.442 | 5 | .696 |
| 3 | e | 0 | 966.888 |  | .894 | 230.324 | 5 | .822 |
| 4 | e | 0 | 102.823 |  | .912 | 174.046 | 3 | .000 |
| 5 | e | 0 | 138.833 |  | .342 | 136.470 | 1 | 1.118 |
| 6 | e | 0 | 162.696 |  | .147 | 134.008 | 1 | 1.085 |
| 7 | e | 0 | 167.348 |  | .026 | 133.951 | 1 | 1.021 |
| 8 | e | 0 | 172.134 |  | .001 | 133.951 | 1 | 1.001 |

##### Pairwise Parameter Comparisons (Default model)

##### Correlations of Estimates (Default model)

|  | par\_1 | par\_2 | par\_3 | par\_4 | par\_5 | par\_6 | par\_7 | par\_8 | par\_9 | par\_10 | par\_11 | par\_12 | par\_13 | par\_14 | par\_15 | par\_16 | par\_17 | par\_18 | par\_19 | par\_20 | par\_21 | par\_22 | par\_23 | par\_24 | par\_25 | par\_26 | par\_27 | par\_28 | par\_29 | par\_30 | par\_31 | par\_32 | par\_33 | par\_34 | par\_35 | par\_36 | par\_37 | par\_38 | par\_39 | par\_40 | par\_41 | par\_42 |
| --- | --- | --- | --- | --- | --- | --- | --- | --- | --- | --- | --- | --- | --- | --- | --- | --- | --- | --- | --- | --- | --- | --- | --- | --- | --- | --- | --- | --- | --- | --- | --- | --- | --- | --- | --- | --- | --- | --- | --- | --- | --- | --- |
| par\_1 | 1.000 |  |  |  |  |  |  |  |  |  |  |  |  |  |  |  |  |  |  |  |  |  |  |  |  |  |  |  |  |  |  |  |  |  |  |  |  |  |  |  |  |  |
| par\_2 | .425 | 1.000 |  |  |  |  |  |  |  |  |  |  |  |  |  |  |  |  |  |  |  |  |  |  |  |  |  |  |  |  |  |  |  |  |  |  |  |  |  |  |  |  |
| par\_3 | .447 | .425 | 1.000 |  |  |  |  |  |  |  |  |  |  |  |  |  |  |  |  |  |  |  |  |  |  |  |  |  |  |  |  |  |  |  |  |  |  |  |  |  |  |  |
| par\_4 | .000 | .000 | .000 | 1.000 |  |  |  |  |  |  |  |  |  |  |  |  |  |  |  |  |  |  |  |  |  |  |  |  |  |  |  |  |  |  |  |  |  |  |  |  |  |  |
| par\_5 | .000 | .000 | .000 | .588 | 1.000 |  |  |  |  |  |  |  |  |  |  |  |  |  |  |  |  |  |  |  |  |  |  |  |  |  |  |  |  |  |  |  |  |  |  |  |  |  |
| par\_6 | .000 | .000 | .000 | .652 | .639 | 1.000 |  |  |  |  |  |  |  |  |  |  |  |  |  |  |  |  |  |  |  |  |  |  |  |  |  |  |  |  |  |  |  |  |  |  |  |  |
| par\_7 | .000 | .000 | .000 | .643 | .630 | .697 | 1.000 |  |  |  |  |  |  |  |  |  |  |  |  |  |  |  |  |  |  |  |  |  |  |  |  |  |  |  |  |  |  |  |  |  |  |  |
| par\_8 | .000 | .000 | .000 | .000 | .000 | .000 | .000 | 1.000 |  |  |  |  |  |  |  |  |  |  |  |  |  |  |  |  |  |  |  |  |  |  |  |  |  |  |  |  |  |  |  |  |  |  |
| par\_9 | .000 | .000 | .000 | .000 | .000 | .000 | .000 | .550 | 1.000 |  |  |  |  |  |  |  |  |  |  |  |  |  |  |  |  |  |  |  |  |  |  |  |  |  |  |  |  |  |  |  |  |  |
| par\_10 | .000 | .000 | .000 | .000 | .000 | .000 | .000 | .519 | .530 | 1.000 |  |  |  |  |  |  |  |  |  |  |  |  |  |  |  |  |  |  |  |  |  |  |  |  |  |  |  |  |  |  |  |  |
| par\_11 | .000 | .000 | .000 | .000 | .000 | .000 | .000 | .000 | .000 | .000 | 1.000 |  |  |  |  |  |  |  |  |  |  |  |  |  |  |  |  |  |  |  |  |  |  |  |  |  |  |  |  |  |  |  |
| par\_12 | .000 | .000 | .000 | .000 | .000 | .000 | .000 | .000 | .000 | .000 | .515 | 1.000 |  |  |  |  |  |  |  |  |  |  |  |  |  |  |  |  |  |  |  |  |  |  |  |  |  |  |  |  |  |  |
| par\_13 | .000 | .000 | .000 | .000 | .000 | .000 | .000 | .000 | .000 | .000 | .466 | .507 | 1.000 |  |  |  |  |  |  |  |  |  |  |  |  |  |  |  |  |  |  |  |  |  |  |  |  |  |  |  |  |  |
| par\_14 | -.177 | -.168 | -.177 | -.351 | -.344 | -.385 | -.378 | .000 | .000 | .000 | .000 | .000 | .000 | 1.000 |  |  |  |  |  |  |  |  |  |  |  |  |  |  |  |  |  |  |  |  |  |  |  |  |  |  |  |  |
| par\_15 | -.181 | -.171 | -.181 | .000 | .000 | .000 | .000 | -.299 | -.308 | -.285 | .000 | .000 | .000 | .333 | 1.000 |  |  |  |  |  |  |  |  |  |  |  |  |  |  |  |  |  |  |  |  |  |  |  |  |  |  |  |
| par\_16 | -.141 | -.133 | -.141 | .000 | .000 | .000 | .000 | .000 | .000 | .000 | -.182 | -.203 | -.179 | .448 | .453 | 1.000 |  |  |  |  |  |  |  |  |  |  |  |  |  |  |  |  |  |  |  |  |  |  |  |  |  |  |
| par\_17 | .000 | .000 | .000 | -.224 | -.219 | -.245 | -.241 | -.187 | -.192 | -.178 | .000 | .000 | .000 | .497 | .478 | .295 | 1.000 |  |  |  |  |  |  |  |  |  |  |  |  |  |  |  |  |  |  |  |  |  |  |  |  |  |
| par\_18 | .000 | .000 | .000 | -.346 | -.338 | -.379 | -.373 | .000 | .000 | .000 | -.227 | -.253 | -.223 | .483 | .163 | .466 | .486 | 1.000 |  |  |  |  |  |  |  |  |  |  |  |  |  |  |  |  |  |  |  |  |  |  |  |  |
| par\_19 | .000 | .000 | .000 | .000 | .000 | .000 | .000 | -.292 | -.301 | -.279 | -.229 | -.256 | -.225 | .164 | .447 | .475 | .471 | .355 | 1.000 |  |  |  |  |  |  |  |  |  |  |  |  |  |  |  |  |  |  |  |  |  |  |  |
| par\_20 | .422 | .401 | .422 | .000 | .000 | .000 | .000 | .000 | .000 | .000 | .000 | .000 | .000 | -.167 | -.170 | -.132 | .000 | .000 | .000 | 1.000 |  |  |  |  |  |  |  |  |  |  |  |  |  |  |  |  |  |  |  |  |  |  |
| par\_21 | -.499 | -.473 | -.499 | .000 | .000 | .000 | .000 | .000 | .000 | .000 | .000 | .000 | .000 | .516 | .527 | .409 | .174 | .107 | .109 | -.469 | 1.000 |  |  |  |  |  |  |  |  |  |  |  |  |  |  |  |  |  |  |  |  |  |
| par\_22 | .000 | .000 | .000 | -.686 | -.672 | -.749 | -.738 | .000 | .000 | .000 | .000 | .000 | .000 | .622 | .066 | .129 | .397 | .613 | .065 | .000 | .076 | 1.000 |  |  |  |  |  |  |  |  |  |  |  |  |  |  |  |  |  |  |  |  |
| par\_23 | .000 | .000 | .000 | .000 | .000 | .000 | .000 | -.636 | -.653 | -.609 | .000 | .000 | .000 | .073 | .590 | .144 | .369 | .071 | .577 | .000 | .086 | .020 | 1.000 |  |  |  |  |  |  |  |  |  |  |  |  |  |  |  |  |  |  |  |
| par\_24 | .000 | .000 | .000 | .000 | .000 | .000 | .000 | .000 | .000 | .000 | -.563 | -.622 | -.554 | .100 | .101 | .432 | .158 | .538 | .544 | .000 | .046 | .069 | .077 | 1.000 |  |  |  |  |  |  |  |  |  |  |  |  |  |  |  |  |  |  |
| par\_25 | .209 | .195 | .209 | .000 | .000 | .000 | .000 | .000 | .000 | .000 | .000 | .000 | .000 | -.061 | -.063 | -.049 | .000 | .000 | .000 | .194 | -.197 | .000 | .000 | .000 | 1.000 |  |  |  |  |  |  |  |  |  |  |  |  |  |  |  |  |  |
| par\_26 | -.196 | -.007 | -.005 | .000 | .000 | .000 | .000 | .000 | .000 | .000 | .000 | .000 | .000 | .017 | .018 | .014 | .000 | .000 | .000 | -.007 | .031 | .000 | .000 | .000 | -.069 | 1.000 |  |  |  |  |  |  |  |  |  |  |  |  |  |  |  |  |
| par\_27 | -.004 | -.176 | -.004 | .000 | .000 | .000 | .000 | .000 | .000 | .000 | .000 | .000 | .000 | .013 | .014 | .011 | .000 | .000 | .000 | -.005 | .024 | .000 | .000 | .000 | -.053 | -.037 | 1.000 |  |  |  |  |  |  |  |  |  |  |  |  |  |  |  |
| par\_28 | -.005 | -.007 | -.196 | .000 | .000 | .000 | .000 | .000 | .000 | .000 | .000 | .000 | .000 | .017 | .018 | .014 | .000 | .000 | .000 | -.007 | .031 | .000 | .000 | .000 | -.069 | -.048 | -.037 | 1.000 |  |  |  |  |  |  |  |  |  |  |  |  |  |  |
| par\_29 | .000 | .000 | .000 | .142 | .139 | .158 | .155 | .000 | .000 | .000 | .000 | .000 | .000 | -.075 | .000 | .000 | -.048 | -.074 | .000 | .000 | .000 | -.153 | .000 | .000 | .000 | .000 | .000 | .000 | 1.000 |  |  |  |  |  |  |  |  |  |  |  |  |  |
| par\_30 | .000 | .000 | .000 | -.151 | .001 | .009 | .007 | .000 | .000 | .000 | .000 | .000 | .000 | .012 | .000 | .000 | .008 | .012 | .000 | .000 | .000 | .013 | .000 | .000 | .000 | .000 | .000 | .000 | -.016 | 1.000 |  |  |  |  |  |  |  |  |  |  |  |  |
| par\_31 | .000 | .000 | .000 | .002 | -.145 | .008 | .006 | .000 | .000 | .000 | .000 | .000 | .000 | .011 | .000 | .000 | .007 | .011 | .000 | .000 | .000 | .011 | .000 | .000 | .000 | .000 | .000 | .000 | -.014 | -.024 | 1.000 |  |  |  |  |  |  |  |  |  |  |  |
| par\_32 | .000 | .000 | .000 | .004 | .003 | -.196 | .016 | .000 | .000 | .000 | .000 | .000 | .000 | .028 | .000 | .000 | .018 | .028 | .000 | .000 | .000 | .029 | .000 | .000 | .000 | .000 | .000 | .000 | -.037 | -.062 | -.055 | 1.000 |  |  |  |  |  |  |  |  |  |  |
| par\_33 | .000 | .000 | .000 | .003 | .002 | .018 | -.184 | .000 | .000 | .000 | .000 | .000 | .000 | .023 | .000 | .000 | .015 | .023 | .000 | .000 | .000 | .024 | .000 | .000 | .000 | .000 | .000 | .000 | -.031 | -.051 | -.045 | -.118 | 1.000 |  |  |  |  |  |  |  |  |  |
| par\_34 | .000 | .000 | .000 | .000 | .000 | .000 | .000 | .238 | .248 | .225 | .000 | .000 | .000 | .000 | -.102 | .000 | -.064 | .000 | -.100 | .000 | .000 | .000 | -.243 | .000 | .000 | .000 | .000 | .000 | .000 | .000 | .000 | .000 | .000 | 1.000 |  |  |  |  |  |  |  |  |
| par\_35 | .000 | .000 | .000 | .000 | .000 | .000 | .000 | -.250 | .012 | .000 | .000 | .000 | .000 | .000 | .034 | .000 | .021 | .000 | .034 | .000 | .000 | .000 | .042 | .000 | .000 | .000 | .000 | .000 | .000 | .000 | .000 | .000 | .000 | -.063 | 1.000 |  |  |  |  |  |  |  |
| par\_36 | .000 | .000 | .000 | .000 | .000 | .000 | .000 | .007 | -.271 | .000 | .000 | .000 | .000 | .000 | .043 | .000 | .027 | .000 | .042 | .000 | .000 | .000 | .052 | .000 | .000 | .000 | .000 | .000 | .000 | .000 | .000 | .000 | .000 | -.078 | -.105 | 1.000 |  |  |  |  |  |  |
| par\_37 | .000 | .000 | .000 | .000 | .000 | .000 | .000 | .004 | .009 | -.225 | .000 | .000 | .000 | .000 | .026 | .000 | .016 | .000 | .025 | .000 | .000 | .000 | .031 | .000 | .000 | .000 | .000 | .000 | .000 | .000 | .000 | .000 | .000 | -.047 | -.063 | -.078 | 1.000 |  |  |  |  |  |
| par\_38 | .000 | .000 | .000 | .000 | .000 | .000 | .000 | .000 | .000 | .000 | .226 | .264 | .222 | .000 | .000 | -.068 | .000 | -.085 | -.086 | .000 | .000 | .000 | .000 | -.235 | .000 | .000 | .000 | .000 | .000 | .000 | .000 | .000 | .000 | .000 | .000 | .000 | .000 | 1.000 |  |  |  |  |
| par\_39 | .000 | .000 | .000 | .000 | .000 | .000 | .000 | .000 | .000 | .000 | -.219 | .013 | -.003 | .000 | .000 | .017 | .000 | .021 | .021 | .000 | .000 | .000 | .000 | .031 | .000 | .000 | .000 | .000 | .000 | .000 | .000 | .000 | .000 | .000 | .000 | .000 | .000 | -.055 | 1.000 |  |  |  |
| par\_40 | .000 | .000 | .000 | .000 | .000 | .000 | .000 | .000 | .000 | .000 | -.005 | -.291 | -.007 | .000 | .000 | .036 | .000 | .045 | .045 | .000 | .000 | .000 | .000 | .067 | .000 | .000 | .000 | .000 | .000 | .000 | .000 | .000 | .000 | .000 | .000 | .000 | .000 | -.117 | -.097 | 1.000 |  |  |
| par\_41 | .000 | .000 | .000 | .000 | .000 | .000 | .000 | .000 | .000 | .000 | -.002 | .012 | -.211 | .000 | .000 | .015 | .000 | .019 | .019 | .000 | .000 | .000 | .000 | .029 | .000 | .000 | .000 | .000 | .000 | .000 | .000 | .000 | .000 | .000 | .000 | .000 | .000 | -.050 | -.042 | -.089 | 1.000 |  |
| par\_42 | -.004 | -.005 | -.004 | .000 | .000 | .000 | .000 | .000 | .000 | .000 | .000 | .000 | .000 | .013 | .013 | .010 | .000 | .000 | .000 | -.174 | .023 | .000 | .000 | .000 | -.052 | -.036 | -.028 | -.036 | .000 | .000 | .000 | .000 | .000 | .000 | .000 | .000 | .000 | .000 | .000 | .000 | .000 | 1.000 |

##### Model Fit Summary

##### CMIN

| Model | NPAR | CMIN | DF | P | CMIN/DF |
| --- | --- | --- | --- | --- | --- |
| Default model | 42 | 133.951 | 129 | .365 | 1.038 |
| Saturated model | 171 | .000 | 0 |  |  |
| Independence model | 18 | 3236.025 | 153 | .000 | 21.150 |

##### RMR, GFI

| Model | RMR | GFI | AGFI | PGFI |
| --- | --- | --- | --- | --- |
| Default model | .102 | .963 | .952 | .727 |
| Saturated model | .000 | 1.000 |  |  |
| Independence model | 1.055 | .337 | .259 | .302 |

##### Baseline Comparisons

| Model | NFI Delta1 | RFI rho1 | IFI Delta2 | TLI rho2 | CFI |
| --- | --- | --- | --- | --- | --- |
| Default model | .959 | .951 | .998 | .998 | .998 |
| Saturated model | 1.000 |  | 1.000 |  | 1.000 |
| Independence model | .000 | .000 | .000 | .000 | .000 |

##### Parsimony-Adjusted Measures

| Model | PRATIO | PNFI | PCFI |
| --- | --- | --- | --- |
| Default model | .843 | .808 | .842 |
| Saturated model | .000 | .000 | .000 |
| Independence model | 1.000 | .000 | .000 |

##### NCP

| Model | NCP | LO 90 | HI 90 |
| --- | --- | --- | --- |
| Default model | 4.951 | .000 | 36.639 |
| Saturated model | .000 | .000 | .000 |
| Independence model | 3083.025 | 2901.704 | 3271.665 |

##### FMIN

| Model | FMIN | F0 | LO 90 | HI 90 |
| --- | --- | --- | --- | --- |
| Default model | .347 | .013 | .000 | .095 |
| Saturated model | .000 | .000 | .000 | .000 |
| Independence model | 8.383 | 7.987 | 7.517 | 8.476 |

##### RMSEA

| Model | RMSEA | LO 90 | HI 90 | PCLOSE |
| --- | --- | --- | --- | --- |
| Default model | .010 | .000 | .027 | 1.000 |
| Independence model | .228 | .222 | .235 | .000 |

##### AIC

| Model | AIC | BCC | BIC | CAIC |
| --- | --- | --- | --- | --- |
| Default model | 217.951 | 222.300 | 384.205 | 426.205 |
| Saturated model | 342.000 | 359.706 | 1018.891 | 1189.891 |
| Independence model | 3272.025 | 3273.889 | 3343.277 | 3361.277 |

##### ECVI

| Model | ECVI | LO 90 | HI 90 | MECVI |
| --- | --- | --- | --- | --- |
| Default model | .565 | .552 | .647 | .576 |
| Saturated model | .886 | .886 | .886 | .932 |
| Independence model | 8.477 | 8.007 | 8.965 | 8.482 |

##### HOELTER

| Model | HOELTER .05 | HOELTER .01 |
| --- | --- | --- |
| Default model | 451 | 488 |
| Independence model | 22 | 24 |

##### Execution time summary

|  |  |
| --- | --- |
| Minimization: | .018 |
| Miscellaneous: | .256 |
| Bootstrap: | .000 |
| Total: | .274 |
