# Supplementary material for: How AI threat perception relates to post-adoptive AI agent engagement: the dual paths of self-efficacy and defensive motivation
Source: Front Psychol. 2026 Oct 22;17:1887571. doi: 10.3389/fpsyg.2026.1887571 (PMC13735560; doi:10.3389/fpsyg.2026.1887571)
Supplement: Supplementary file 1 [file Data_Sheet_1.zip › log+Questionnaire/003structural mediation model.AmosOutput]

路径经验-FS.amw


#### D:\学术研究\066研究数据与论文撰写\01研究数据\AI智能体论文\路径检验\路径经验-FS.amw

##### Analysis Summary

##### Date and Time

Date: 2026年9月13日

Time: 9:36:32

##### Title

路径经验-fs: 2026年9月13日 9:36

##### Groups

##### Group number 1 (Group number 1)

##### Notes for Group (Group number 1)

The model is recursive.

Sample size = 387

##### Variable Summary (Group number 1)

##### Your model contains the following variables (Group number 1)

Observed, endogenous variables

AA1

AA2

AA4

AA5

DM1

DM2

DM3

DM5

SE1

SE2

SE3

SE4

SE5

AB1

AB2

AB3

AB5

AA3

Unobserved, endogenous variables

DM

SE

AB

Unobserved, exogenous variables

AA

e1

e2

e4

e5

e6

e8

e9

e10

e11

e12

e13

e14

e15

e16

e17

e18

e20

e21

e22

e23

e3

##### Variable counts (Group number 1)

|  |  |
| --- | --- |
| Number of variables in your model: | 43 |
| Number of observed variables: | 18 |
| Number of unobserved variables: | 25 |
| Number of exogenous variables: | 22 |
| Number of endogenous variables: | 21 |

##### Parameter Summary (Group number 1)

|  | Weights | Covariances | Variances | Means | Intercepts | Total |
| --- | --- | --- | --- | --- | --- | --- |
| Fixed | 25 | 0 | 0 | 0 | 0 | 25 |
| Labeled | 6 | 0 | 0 | 0 | 0 | 6 |
| Unlabeled | 14 | 0 | 22 | 0 | 0 | 36 |
| Total | 45 | 0 | 22 | 0 | 0 | 67 |

##### Assessment of normality (Group number 1)

| Variable | min | max | skew | c.r. | kurtosis | c.r. |
| --- | --- | --- | --- | --- | --- | --- |
| AA3 | 1.000 | 7.000 | .101 | .815 | -1.091 | -4.381 |
| AB5 | 1.000 | 7.000 | .037 | .300 | -1.113 | -4.468 |
| AB3 | 1.000 | 7.000 | .050 | .401 | -1.063 | -4.269 |
| AB2 | 1.000 | 7.000 | .022 | .176 | -1.121 | -4.502 |
| AB1 | 1.000 | 7.000 | .085 | .682 | -1.100 | -4.419 |
| SE5 | 1.000 | 7.000 | .039 | .316 | -1.060 | -4.255 |
| SE4 | 1.000 | 7.000 | .057 | .459 | -1.143 | -4.590 |
| SE3 | 1.000 | 7.000 | .052 | .414 | -1.103 | -4.430 |
| SE2 | 1.000 | 7.000 | .056 | .452 | -1.079 | -4.334 |
| SE1 | 1.000 | 7.000 | .095 | .766 | -1.050 | -4.218 |
| DM5 | 1.000 | 7.000 | .056 | .451 | -1.117 | -4.484 |
| DM3 | 1.000 | 7.000 | .108 | .865 | -1.045 | -4.198 |
| DM2 | 1.000 | 7.000 | .100 | .801 | -1.065 | -4.277 |
| DM1 | 1.000 | 7.000 | .057 | .460 | -1.121 | -4.501 |
| AA5 | 1.000 | 7.000 | .057 | .458 | -1.083 | -4.351 |
| AA4 | 1.000 | 7.000 | .037 | .297 | -1.126 | -4.520 |
| AA2 | 1.000 | 7.000 | .091 | .734 | -1.095 | -4.399 |
| AA1 | 1.000 | 7.000 | .089 | .715 | -1.104 | -4.433 |
| Multivariate |  |  |  |  | -3.427 | -1.256 |

##### Observations farthest from the centroid (Mahalanobis distance) (Group number 1)

| Observation number | Mahalanobis d-squared | p1 | p2 |
| --- | --- | --- | --- |
| 140 | 36.925 | .005 | .875 |
| 17 | 34.263 | .012 | .941 |
| 280 | 34.260 | .012 | .831 |
| 256 | 34.099 | .012 | .698 |
| 238 | 32.133 | .021 | .914 |
| 156 | 31.926 | .022 | .866 |
| 324 | 31.684 | .024 | .820 |
| 300 | 31.173 | .027 | .836 |
| 343 | 30.980 | .029 | .789 |
| 368 | 29.736 | .040 | .949 |
| 6 | 29.681 | .041 | .918 |
| 285 | 29.675 | .041 | .866 |
| 29 | 29.432 | .043 | .859 |
| 177 | 29.217 | .046 | .849 |
| 237 | 28.863 | .050 | .875 |
| 12 | 28.842 | .050 | .822 |
| 311 | 28.609 | .053 | .826 |
| 68 | 28.457 | .055 | .808 |
| 42 | 28.255 | .058 | .809 |
| 3 | 27.888 | .064 | .861 |
| 196 | 27.858 | .064 | .816 |
| 8 | 27.756 | .066 | .791 |
| 52 | 27.714 | .067 | .741 |
| 353 | 27.680 | .067 | .684 |
| 135 | 27.420 | .071 | .727 |
| 209 | 26.869 | .081 | .871 |
| 307 | 26.680 | .085 | .883 |
| 222 | 26.637 | .086 | .854 |
| 229 | 26.543 | .088 | .840 |
| 218 | 26.338 | .092 | .864 |
| 65 | 26.305 | .093 | .831 |
| 333 | 26.283 | .093 | .790 |
| 279 | 26.160 | .096 | .789 |
| 100 | 26.094 | .098 | .765 |
| 30 | 25.841 | .103 | .822 |
| 146 | 25.797 | .104 | .792 |
| 195 | 25.555 | .110 | .844 |
| 74 | 25.505 | .112 | .821 |
| 309 | 25.496 | .112 | .777 |
| 80 | 25.136 | .121 | .877 |
| 211 | 25.060 | .123 | .869 |
| 183 | 24.971 | .126 | .865 |
| 347 | 24.964 | .126 | .830 |
| 217 | 24.954 | .126 | .791 |
| 277 | 24.831 | .130 | .803 |
| 299 | 24.749 | .132 | .798 |
| 186 | 24.721 | .133 | .766 |
| 59 | 24.620 | .136 | .770 |
| 141 | 24.607 | .136 | .729 |
| 219 | 24.593 | .137 | .685 |
| 362 | 24.284 | .146 | .804 |
| 276 | 24.155 | .150 | .824 |
| 382 | 24.149 | .150 | .787 |
| 243 | 24.135 | .151 | .749 |
| 269 | 24.064 | .153 | .743 |
| 206 | 23.942 | .157 | .765 |
| 260 | 23.941 | .157 | .720 |
| 155 | 23.919 | .158 | .684 |
| 210 | 23.908 | .158 | .640 |
| 233 | 23.905 | .158 | .588 |
| 326 | 23.846 | .160 | .575 |
| 71 | 23.828 | .161 | .533 |
| 125 | 23.691 | .165 | .577 |
| 245 | 23.635 | .167 | .563 |
| 168 | 23.553 | .170 | .568 |
| 87 | 23.542 | .171 | .523 |
| 48 | 23.423 | .175 | .556 |
| 203 | 23.196 | .183 | .668 |
| 4 | 23.089 | .187 | .693 |
| 304 | 23.037 | .189 | .681 |
| 321 | 22.927 | .193 | .709 |
| 129 | 22.882 | .195 | .694 |
| 316 | 22.878 | .195 | .650 |
| 287 | 22.866 | .196 | .610 |
| 314 | 22.803 | .198 | .607 |
| 370 | 22.773 | .199 | .581 |
| 116 | 22.471 | .212 | .749 |
| 103 | 22.401 | .215 | .753 |
| 86 | 22.379 | .216 | .727 |
| 28 | 22.327 | .218 | .720 |
| 113 | 22.204 | .223 | .760 |
| 253 | 22.172 | .224 | .741 |
| 166 | 21.956 | .234 | .832 |
| 75 | 21.930 | .235 | .815 |
| 61 | 21.901 | .236 | .798 |
| 334 | 21.891 | .237 | .768 |
| 47 | 21.865 | .238 | .747 |
| 250 | 21.820 | .240 | .738 |
| 228 | 21.815 | .240 | .701 |
| 64 | 21.583 | .251 | .815 |
| 313 | 21.575 | .251 | .786 |
| 92 | 21.556 | .252 | .762 |
| 81 | 21.554 | .252 | .726 |
| 121 | 21.487 | .256 | .733 |
| 297 | 21.475 | .256 | .703 |
| 387 | 21.412 | .259 | .708 |
| 132 | 21.412 | .259 | .667 |
| 49 | 21.315 | .264 | .700 |
| 376 | 21.299 | .265 | .671 |
| 220 | 21.275 | .266 | .647 |

##### Models

##### Default model (Default model)

##### Notes for Model (Default model)

##### Computation of degrees of freedom (Default model)

|  |  |
| --- | --- |
| Number of distinct sample moments: | 171 |
| Number of distinct parameters to be estimated: | 42 |
| Degrees of freedom (171 - 42): | 129 |

##### Result (Default model)

Minimum was achieved

Chi-square = 133.951

Degrees of freedom = 129

Probability level = .365

##### Group number 1 (Group number 1 - Default model)

##### Estimates (Group number 1 - Default model)

##### Scalar Estimates (Group number 1 - Default model)

##### Maximum Likelihood Estimates

##### Regression Weights: (Group number 1 - Default model)

|  |  |  | Estimate | S.E. | C.R. | P | Label |
| --- | --- | --- | --- | --- | --- | --- | --- |
| SE | <--- | AA | .377 | .049 | 7.636 | \*\*\* | H1 |
| DM | <--- | AA | .404 | .062 | 6.505 | \*\*\* | H2 |
| DM | <--- | SE | .069 | .074 | .934 | .350 | H5 |
| AB | <--- | DM | .434 | .073 | 5.949 | \*\*\* | H4 |
| AB | <--- | SE | .493 | .082 | 6.003 | \*\*\* | H3 |
| AB | <--- | AA | -.055 | .063 | -.872 | .383 | c\_dir |
| AA1 | <--- | AA | 1.000 |  |  |  |  |
| AA2 | <--- | AA | .952 | .057 | 16.762 | \*\*\* |  |
| AA4 | <--- | AA | .921 | .058 | 15.935 | \*\*\* |  |
| AA5 | <--- | AA | .945 | .056 | 16.766 | \*\*\* |  |
| DM1 | <--- | DM | 1.000 |  |  |  |  |
| DM2 | <--- | DM | 1.026 | .082 | 12.470 | \*\*\* |  |
| DM3 | <--- | DM | 1.051 | .083 | 12.738 | \*\*\* |  |
| DM5 | <--- | DM | .984 | .082 | 12.001 | \*\*\* |  |
| SE1 | <--- | SE | 1.000 |  |  |  |  |
| SE2 | <--- | SE | 1.165 | .101 | 11.512 | \*\*\* |  |
| SE3 | <--- | SE | 1.099 | .097 | 11.277 | \*\*\* |  |
| SE4 | <--- | SE | 1.284 | .103 | 12.504 | \*\*\* |  |
| SE5 | <--- | SE | 1.222 | .099 | 12.338 | \*\*\* |  |
| AB1 | <--- | AB | 1.000 |  |  |  |  |
| AB2 | <--- | AB | .975 | .071 | 13.751 | \*\*\* |  |
| AB3 | <--- | AB | 1.064 | .071 | 14.925 | \*\*\* |  |
| AB5 | <--- | AB | .964 | .071 | 13.543 | \*\*\* |  |
| AA3 | <--- | AA | .920 | .058 | 15.833 | \*\*\* |  |

##### Standardized Regression Weights: (Group number 1 - Default model)

|  |  |  | Estimate |
| --- | --- | --- | --- |
| SE | <--- | AA | .486 |
| DM | <--- | AA | .460 |
| DM | <--- | SE | .061 |
| AB | <--- | DM | .409 |
| AB | <--- | SE | .411 |
| AB | <--- | AA | -.059 |
| AA1 | <--- | AA | .818 |
| AA2 | <--- | AA | .782 |
| AA4 | <--- | AA | .752 |
| AA5 | <--- | AA | .782 |
| DM1 | <--- | DM | .705 |
| DM2 | <--- | DM | .739 |
| DM3 | <--- | DM | .761 |
| DM5 | <--- | DM | .705 |
| SE1 | <--- | SE | .636 |
| SE2 | <--- | SE | .716 |
| SE3 | <--- | SE | .696 |
| SE4 | <--- | SE | .805 |
| SE5 | <--- | SE | .788 |
| AB1 | <--- | AB | .758 |
| AB2 | <--- | AB | .737 |
| AB3 | <--- | AB | .806 |
| AB5 | <--- | AB | .725 |
| AA3 | <--- | AA | .748 |

##### Variances: (Group number 1 - Default model)

|  |  |  | Estimate | S.E. | C.R. | P | Label |
| --- | --- | --- | --- | --- | --- | --- | --- |
| AA |  |  | 2.181 | .232 | 9.396 | \*\*\* |  |
| e21 |  |  | 1.004 | .156 | 6.414 | \*\*\* |  |
| e23 |  |  | 1.273 | .181 | 7.049 | \*\*\* |  |
| e22 |  |  | 1.161 | .155 | 7.470 | \*\*\* |  |
| e1 |  |  | 1.082 | .104 | 10.394 | \*\*\* |  |
| e2 |  |  | 1.255 | .112 | 11.163 | \*\*\* |  |
| e4 |  |  | 1.424 | .122 | 11.651 | \*\*\* |  |
| e5 |  |  | 1.235 | .111 | 11.160 | \*\*\* |  |
| e6 |  |  | 1.698 | .153 | 11.080 | \*\*\* |  |
| e8 |  |  | 1.468 | .140 | 10.452 | \*\*\* |  |
| e9 |  |  | 1.352 | .136 | 9.966 | \*\*\* |  |
| e10 |  |  | 1.646 | .148 | 11.086 | \*\*\* |  |
| e11 |  |  | 1.933 | .155 | 12.448 | \*\*\* |  |
| e12 |  |  | 1.699 | .146 | 11.644 | \*\*\* |  |
| e13 |  |  | 1.686 | .142 | 11.880 | \*\*\* |  |
| e14 |  |  | 1.178 | .119 | 9.912 | \*\*\* |  |
| e15 |  |  | 1.194 | .116 | 10.337 | \*\*\* |  |
| e16 |  |  | 1.409 | .132 | 10.656 | \*\*\* |  |
| e17 |  |  | 1.521 | .138 | 11.053 | \*\*\* |  |
| e18 |  |  | 1.155 | .123 | 9.418 | \*\*\* |  |
| e20 |  |  | 1.586 | .141 | 11.240 | \*\*\* |  |
| e3 |  |  | 1.454 | .124 | 11.703 | \*\*\* |  |

##### Squared Multiple Correlations: (Group number 1 - Default model)

|  |  |  | Estimate |
| --- | --- | --- | --- |
| SE |  |  | .236 |
| DM |  |  | .243 |
| AB |  |  | .388 |
| AA3 |  |  | .559 |
| AB5 |  |  | .526 |
| AB3 |  |  | .650 |
| AB2 |  |  | .543 |
| AB1 |  |  | .574 |
| SE5 |  |  | .622 |
| SE4 |  |  | .648 |
| SE3 |  |  | .485 |
| SE2 |  |  | .512 |
| SE1 |  |  | .405 |
| DM5 |  |  | .497 |
| DM3 |  |  | .579 |
| DM2 |  |  | .547 |
| DM1 |  |  | .498 |
| AA5 |  |  | .612 |
| AA4 |  |  | .565 |
| AA2 |  |  | .612 |
| AA1 |  |  | .668 |

##### User-defined estimands: (Group number 1 - Default model)

|  |  |  |  |
| --- | --- | --- | --- |
| ind\_SE |  |  | .186 |
| ind\_DM |  |  | .176 |
| ind\_Serial |  |  | .011 |
| c\_dir\_effect |  |  | -.055 |

##### Matrices (Group number 1 - Default model)

##### Total Effects (Group number 1 - Default model)

|  | AA | SE | DM | AB |
| --- | --- | --- | --- | --- |
| SE | .377 | .000 | .000 | .000 |
| DM | .430 | .069 | .000 | .000 |
| AB | .318 | .523 | .434 | .000 |
| AA3 | .920 | .000 | .000 | .000 |
| AB5 | .306 | .504 | .419 | .964 |
| AB3 | .338 | .557 | .462 | 1.064 |
| AB2 | .310 | .510 | .424 | .975 |
| AB1 | .318 | .523 | .434 | 1.000 |
| SE5 | .461 | 1.222 | .000 | .000 |
| SE4 | .485 | 1.284 | .000 | .000 |
| SE3 | .415 | 1.099 | .000 | .000 |
| SE2 | .439 | 1.165 | .000 | .000 |
| SE1 | .377 | 1.000 | .000 | .000 |
| DM5 | .423 | .068 | .984 | .000 |
| DM3 | .452 | .073 | 1.051 | .000 |
| DM2 | .442 | .071 | 1.026 | .000 |
| DM1 | .430 | .069 | 1.000 | .000 |
| AA5 | .945 | .000 | .000 | .000 |
| AA4 | .921 | .000 | .000 | .000 |
| AA2 | .952 | .000 | .000 | .000 |
| AA1 | 1.000 | .000 | .000 | .000 |

##### Standardized Total Effects (Group number 1 - Default model)

|  | AA | SE | DM | AB |
| --- | --- | --- | --- | --- |
| SE | .486 | .000 | .000 | .000 |
| DM | .490 | .061 | .000 | .000 |
| AB | .341 | .436 | .409 | .000 |
| AA3 | .748 | .000 | .000 | .000 |
| AB5 | .247 | .316 | .297 | .725 |
| AB3 | .275 | .351 | .330 | .806 |
| AB2 | .251 | .321 | .301 | .737 |
| AB1 | .258 | .330 | .310 | .758 |
| SE5 | .383 | .788 | .000 | .000 |
| SE4 | .391 | .805 | .000 | .000 |
| SE3 | .339 | .696 | .000 | .000 |
| SE2 | .348 | .716 | .000 | .000 |
| SE1 | .309 | .636 | .000 | .000 |
| DM5 | .346 | .043 | .705 | .000 |
| DM3 | .373 | .047 | .761 | .000 |
| DM2 | .362 | .045 | .739 | .000 |
| DM1 | .346 | .043 | .705 | .000 |
| AA5 | .782 | .000 | .000 | .000 |
| AA4 | .752 | .000 | .000 | .000 |
| AA2 | .782 | .000 | .000 | .000 |
| AA1 | .818 | .000 | .000 | .000 |

##### Direct Effects (Group number 1 - Default model)

|  | AA | SE | DM | AB |
| --- | --- | --- | --- | --- |
| SE | .377 | .000 | .000 | .000 |
| DM | .404 | .069 | .000 | .000 |
| AB | -.055 | .493 | .434 | .000 |
| AA3 | .920 | .000 | .000 | .000 |
| AB5 | .000 | .000 | .000 | .964 |
| AB3 | .000 | .000 | .000 | 1.064 |
| AB2 | .000 | .000 | .000 | .975 |
| AB1 | .000 | .000 | .000 | 1.000 |
| SE5 | .000 | 1.222 | .000 | .000 |
| SE4 | .000 | 1.284 | .000 | .000 |
| SE3 | .000 | 1.099 | .000 | .000 |
| SE2 | .000 | 1.165 | .000 | .000 |
| SE1 | .000 | 1.000 | .000 | .000 |
| DM5 | .000 | .000 | .984 | .000 |
| DM3 | .000 | .000 | 1.051 | .000 |
| DM2 | .000 | .000 | 1.026 | .000 |
| DM1 | .000 | .000 | 1.000 | .000 |
| AA5 | .945 | .000 | .000 | .000 |
| AA4 | .921 | .000 | .000 | .000 |
| AA2 | .952 | .000 | .000 | .000 |
| AA1 | 1.000 | .000 | .000 | .000 |

##### Standardized Direct Effects (Group number 1 - Default model)

|  | AA | SE | DM | AB |
| --- | --- | --- | --- | --- |
| SE | .486 | .000 | .000 | .000 |
| DM | .460 | .061 | .000 | .000 |
| AB | -.059 | .411 | .409 | .000 |
| AA3 | .748 | .000 | .000 | .000 |
| AB5 | .000 | .000 | .000 | .725 |
| AB3 | .000 | .000 | .000 | .806 |
| AB2 | .000 | .000 | .000 | .737 |
| AB1 | .000 | .000 | .000 | .758 |
| SE5 | .000 | .788 | .000 | .000 |
| SE4 | .000 | .805 | .000 | .000 |
| SE3 | .000 | .696 | .000 | .000 |
| SE2 | .000 | .716 | .000 | .000 |
| SE1 | .000 | .636 | .000 | .000 |
| DM5 | .000 | .000 | .705 | .000 |
| DM3 | .000 | .000 | .761 | .000 |
| DM2 | .000 | .000 | .739 | .000 |
| DM1 | .000 | .000 | .705 | .000 |
| AA5 | .782 | .000 | .000 | .000 |
| AA4 | .752 | .000 | .000 | .000 |
| AA2 | .782 | .000 | .000 | .000 |
| AA1 | .818 | .000 | .000 | .000 |

##### Indirect Effects (Group number 1 - Default model)

|  | AA | SE | DM | AB |
| --- | --- | --- | --- | --- |
| SE | .000 | .000 | .000 | .000 |
| DM | .026 | .000 | .000 | .000 |
| AB | .373 | .030 | .000 | .000 |
| AA3 | .000 | .000 | .000 | .000 |
| AB5 | .306 | .504 | .419 | .000 |
| AB3 | .338 | .557 | .462 | .000 |
| AB2 | .310 | .510 | .424 | .000 |
| AB1 | .318 | .523 | .434 | .000 |
| SE5 | .461 | .000 | .000 | .000 |
| SE4 | .485 | .000 | .000 | .000 |
| SE3 | .415 | .000 | .000 | .000 |
| SE2 | .439 | .000 | .000 | .000 |
| SE1 | .377 | .000 | .000 | .000 |
| DM5 | .423 | .068 | .000 | .000 |
| DM3 | .452 | .073 | .000 | .000 |
| DM2 | .442 | .071 | .000 | .000 |
| DM1 | .430 | .069 | .000 | .000 |
| AA5 | .000 | .000 | .000 | .000 |
| AA4 | .000 | .000 | .000 | .000 |
| AA2 | .000 | .000 | .000 | .000 |
| AA1 | .000 | .000 | .000 | .000 |

##### Standardized Indirect Effects (Group number 1 - Default model)

|  | AA | SE | DM | AB |
| --- | --- | --- | --- | --- |
| SE | .000 | .000 | .000 | .000 |
| DM | .030 | .000 | .000 | .000 |
| AB | .400 | .025 | .000 | .000 |
| AA3 | .000 | .000 | .000 | .000 |
| AB5 | .247 | .316 | .297 | .000 |
| AB3 | .275 | .351 | .330 | .000 |
| AB2 | .251 | .321 | .301 | .000 |
| AB1 | .258 | .330 | .310 | .000 |
| SE5 | .383 | .000 | .000 | .000 |
| SE4 | .391 | .000 | .000 | .000 |
| SE3 | .339 | .000 | .000 | .000 |
| SE2 | .348 | .000 | .000 | .000 |
| SE1 | .309 | .000 | .000 | .000 |
| DM5 | .346 | .043 | .000 | .000 |
| DM3 | .373 | .047 | .000 | .000 |
| DM2 | .362 | .045 | .000 | .000 |
| DM1 | .346 | .043 | .000 | .000 |
| AA5 | .000 | .000 | .000 | .000 |
| AA4 | .000 | .000 | .000 | .000 |
| AA2 | .000 | .000 | .000 | .000 |
| AA1 | .000 | .000 | .000 | .000 |

##### Modification Indices (Group number 1 - Default model)

##### Covariances: (Group number 1 - Default model)

|  |  |  | M.I. | Par Change |
| --- | --- | --- | --- | --- |
| e20 | <--> | e23 | 5.018 | -.202 |
| e13 | <--> | e20 | 4.448 | .204 |
| e10 | <--> | e20 | 5.071 | -.221 |
| e8 | <--> | e22 | 5.611 | .206 |
| e5 | <--> | e14 | 5.898 | -.188 |
| e4 | <--> | e3 | 5.727 | -.201 |
| e2 | <--> | e22 | 4.157 | -.162 |
| e2 | <--> | e13 | 4.736 | -.189 |
| e2 | <--> | e8 | 4.644 | -.183 |
| e1 | <--> | e3 | 5.035 | .171 |
| e1 | <--> | e12 | 5.385 | -.195 |

##### Variances: (Group number 1 - Default model)

|  |  |  | M.I. | Par Change |
| --- | --- | --- | --- | --- |

##### Regression Weights: (Group number 1 - Default model)

|  |  |  | M.I. | Par Change |
| --- | --- | --- | --- | --- |
| AB5 | <--- | SE3 | 7.009 | .102 |
| AB5 | <--- | DM5 | 5.427 | -.090 |
| SE3 | <--- | AA2 | 5.018 | -.088 |
| DM5 | <--- | AB5 | 6.026 | -.096 |
| DM2 | <--- | AB | 4.277 | .112 |
| DM2 | <--- | AB2 | 5.767 | .091 |
| AA5 | <--- | SE4 | 4.070 | -.069 |
| AA1 | <--- | SE2 | 4.500 | -.068 |

##### Bootstrap (Group number 1 - Default model)

##### Bootstrap standard errors (Group number 1 - Default model)

##### Scalar Estimates (Group number 1 - Default model)

##### Regression Weights: (Group number 1 - Default model)

| Parameter | | | SE | SE-SE | Mean | Bias | SE-Bias |
| --- | --- | --- | --- | --- | --- | --- | --- |
| SE | <--- | AA | .049 | .000 | .377 | -.001 | .001 |
| DM | <--- | AA | .062 | .001 | .406 | .002 | .001 |
| DM | <--- | SE | .076 | .001 | .068 | -.001 | .001 |
| AB | <--- | DM | .070 | .001 | .436 | .002 | .001 |
| AB | <--- | SE | .086 | .001 | .497 | .004 | .001 |
| AB | <--- | AA | .065 | .001 | -.057 | -.002 | .001 |
| AA1 | <--- | AA | .000 | .000 | 1.000 | .000 | .000 |
| AA2 | <--- | AA | .047 | .000 | .953 | .001 | .001 |
| AA4 | <--- | AA | .052 | .001 | .923 | .002 | .001 |
| AA5 | <--- | AA | .051 | .001 | .948 | .002 | .001 |
| DM1 | <--- | DM | .000 | .000 | 1.000 | .000 | .000 |
| DM2 | <--- | DM | .075 | .001 | 1.029 | .003 | .001 |
| DM3 | <--- | DM | .077 | .001 | 1.053 | .002 | .001 |
| DM5 | <--- | DM | .076 | .001 | .986 | .002 | .001 |
| SE1 | <--- | SE | .000 | .000 | 1.000 | .000 | .000 |
| SE2 | <--- | SE | .092 | .001 | 1.169 | .005 | .001 |
| SE3 | <--- | SE | .087 | .001 | 1.104 | .005 | .001 |
| SE4 | <--- | SE | .097 | .001 | 1.291 | .006 | .001 |
| SE5 | <--- | SE | .094 | .001 | 1.228 | .006 | .001 |
| AB1 | <--- | AB | .000 | .000 | 1.000 | .000 | .000 |
| AB2 | <--- | AB | .061 | .001 | .976 | .001 | .001 |
| AB3 | <--- | AB | .064 | .001 | 1.067 | .002 | .001 |
| AB5 | <--- | AB | .063 | .001 | .966 | .002 | .001 |
| AA3 | <--- | AA | .043 | .000 | .921 | .001 | .001 |

##### Standardized Regression Weights: (Group number 1 - Default model)

| Parameter | | | SE | SE-SE | Mean | Bias | SE-Bias |
| --- | --- | --- | --- | --- | --- | --- | --- |
| SE | <--- | AA | .047 | .000 | .485 | -.001 | .001 |
| DM | <--- | AA | .062 | .001 | .461 | .001 | .001 |
| DM | <--- | SE | .066 | .001 | .060 | -.001 | .001 |
| AB | <--- | DM | .059 | .001 | .410 | .001 | .001 |
| AB | <--- | SE | .063 | .001 | .412 | .001 | .001 |
| AB | <--- | AA | .070 | .001 | -.062 | -.002 | .001 |
| AA1 | <--- | AA | .022 | .000 | .817 | .000 | .000 |
| AA2 | <--- | AA | .023 | .000 | .782 | .000 | .000 |
| AA4 | <--- | AA | .027 | .000 | .752 | .000 | .000 |
| AA5 | <--- | AA | .025 | .000 | .783 | .000 | .000 |
| DM1 | <--- | DM | .034 | .000 | .705 | .000 | .000 |
| DM2 | <--- | DM | .031 | .000 | .739 | .000 | .000 |
| DM3 | <--- | DM | .029 | .000 | .760 | -.001 | .000 |
| DM5 | <--- | DM | .032 | .000 | .705 | .000 | .000 |
| SE1 | <--- | SE | .036 | .000 | .635 | -.001 | .001 |
| SE2 | <--- | SE | .032 | .000 | .715 | -.001 | .000 |
| SE3 | <--- | SE | .032 | .000 | .696 | .000 | .000 |
| SE4 | <--- | SE | .023 | .000 | .804 | -.001 | .000 |
| SE5 | <--- | SE | .025 | .000 | .788 | .000 | .000 |
| AB1 | <--- | AB | .028 | .000 | .757 | -.001 | .000 |
| AB2 | <--- | AB | .031 | .000 | .736 | -.001 | .000 |
| AB3 | <--- | AB | .024 | .000 | .806 | .000 | .000 |
| AB5 | <--- | AB | .030 | .000 | .725 | .000 | .000 |
| AA3 | <--- | AA | .025 | .000 | .748 | .000 | .000 |

##### Variances: (Group number 1 - Default model)

| Parameter | | | SE | SE-SE | Mean | Bias | SE-Bias |
| --- | --- | --- | --- | --- | --- | --- | --- |
| AA |  |  | .186 | .002 | 2.178 | -.004 | .003 |
| e21 |  |  | .144 | .001 | 1.001 | -.002 | .002 |
| e23 |  |  | .171 | .002 | 1.264 | -.009 | .002 |
| e22 |  |  | .143 | .001 | 1.145 | -.016 | .002 |
| e1 |  |  | .108 | .001 | 1.079 | -.004 | .002 |
| e2 |  |  | .109 | .001 | 1.250 | -.005 | .002 |
| e4 |  |  | .122 | .001 | 1.414 | -.010 | .002 |
| e5 |  |  | .122 | .001 | 1.227 | -.008 | .002 |
| e6 |  |  | .152 | .002 | 1.688 | -.010 | .002 |
| e8 |  |  | .142 | .001 | 1.459 | -.009 | .002 |
| e9 |  |  | .126 | .001 | 1.345 | -.007 | .002 |
| e10 |  |  | .138 | .001 | 1.637 | -.009 | .002 |
| e11 |  |  | .142 | .001 | 1.925 | -.008 | .002 |
| e12 |  |  | .159 | .002 | 1.694 | -.005 | .002 |
| e13 |  |  | .139 | .001 | 1.677 | -.009 | .002 |
| e14 |  |  | .114 | .001 | 1.174 | -.003 | .002 |
| e15 |  |  | .118 | .001 | 1.189 | -.005 | .002 |
| e16 |  |  | .127 | .001 | 1.403 | -.006 | .002 |
| e17 |  |  | .149 | .001 | 1.515 | -.006 | .002 |
| e18 |  |  | .123 | .001 | 1.151 | -.004 | .002 |
| e20 |  |  | .133 | .001 | 1.579 | -.007 | .002 |
| e3 |  |  | .113 | .001 | 1.446 | -.008 | .002 |

##### Squared Multiple Correlations: (Group number 1 - Default model)

| Parameter | | | SE | SE-SE | Mean | Bias | SE-Bias |
| --- | --- | --- | --- | --- | --- | --- | --- |
| SE |  |  | .045 | .000 | .237 | .001 | .001 |
| DM |  |  | .049 | .000 | .249 | .006 | .001 |
| AB |  |  | .052 | .001 | .395 | .007 | .001 |
| AA3 |  |  | .037 | .000 | .560 | .001 | .001 |
| AB5 |  |  | .043 | .000 | .527 | .000 | .001 |
| AB3 |  |  | .039 | .000 | .650 | .000 | .001 |
| AB2 |  |  | .046 | .000 | .542 | .000 | .001 |
| AB1 |  |  | .043 | .000 | .574 | .000 | .001 |
| SE5 |  |  | .040 | .000 | .622 | .000 | .001 |
| SE4 |  |  | .037 | .000 | .647 | -.001 | .001 |
| SE3 |  |  | .044 | .000 | .485 | .000 | .001 |
| SE2 |  |  | .046 | .000 | .512 | .000 | .001 |
| SE1 |  |  | .045 | .000 | .405 | .000 | .001 |
| DM5 |  |  | .045 | .000 | .498 | .000 | .001 |
| DM3 |  |  | .043 | .000 | .579 | .000 | .001 |
| DM2 |  |  | .045 | .000 | .548 | .001 | .001 |
| DM1 |  |  | .047 | .000 | .499 | .001 | .001 |
| AA5 |  |  | .040 | .000 | .613 | .001 | .001 |
| AA4 |  |  | .040 | .000 | .566 | .001 | .001 |
| AA2 |  |  | .036 | .000 | .612 | .000 | .001 |
| AA1 |  |  | .036 | .000 | .668 | .000 | .001 |

##### User-defined estimands: (Group number 1 - Default model)

| Parameter | | | SE | SE-SE | Mean | Bias | SE-Bias |
| --- | --- | --- | --- | --- | --- | --- | --- |
| ind\_SE |  |  | .037 | .000 | .187 | .001 | .001 |
| ind\_DM |  |  | .039 | .000 | .177 | .001 | .001 |
| ind\_Serial |  |  | .012 | .000 | .011 | .000 | .000 |
| c\_dir\_effect |  |  | .065 | .001 | -.057 | -.002 | .001 |

##### Matrices (Group number 1 - Default model)

##### Total Effects - Standard Errors (Group number 1 - Default model)

|  | AA | SE | DM | AB |
| --- | --- | --- | --- | --- |
| SE | .049 | .000 | .000 | .000 |
| DM | .053 | .076 | .000 | .000 |
| AB | .055 | .089 | .070 | .000 |
| AA3 | .043 | .000 | .000 | .000 |
| AB5 | .054 | .089 | .065 | .063 |
| AB3 | .059 | .091 | .075 | .064 |
| AB2 | .053 | .086 | .071 | .061 |
| AB1 | .055 | .089 | .070 | .000 |
| SE5 | .055 | .094 | .000 | .000 |
| SE4 | .058 | .097 | .000 | .000 |
| SE3 | .050 | .087 | .000 | .000 |
| SE2 | .054 | .092 | .000 | .000 |
| SE1 | .049 | .000 | .000 | .000 |
| DM5 | .052 | .074 | .076 | .000 |
| DM3 | .055 | .080 | .077 | .000 |
| DM2 | .053 | .078 | .075 | .000 |
| DM1 | .053 | .076 | .000 | .000 |
| AA5 | .051 | .000 | .000 | .000 |
| AA4 | .052 | .000 | .000 | .000 |
| AA2 | .047 | .000 | .000 | .000 |
| AA1 | .000 | .000 | .000 | .000 |

##### Standardized Total Effects - Standard Errors (Group number 1 - Default model)

|  | AA | SE | DM | AB |
| --- | --- | --- | --- | --- |
| SE | .047 | .000 | .000 | .000 |
| DM | .050 | .066 | .000 | .000 |
| AB | .054 | .065 | .059 | .000 |
| AA3 | .025 | .000 | .000 | .000 |
| AB5 | .041 | .051 | .042 | .030 |
| AB3 | .045 | .053 | .049 | .024 |
| AB2 | .041 | .050 | .047 | .031 |
| AB1 | .042 | .052 | .046 | .028 |
| SE5 | .041 | .025 | .000 | .000 |
| SE4 | .041 | .023 | .000 | .000 |
| SE3 | .037 | .032 | .000 | .000 |
| SE2 | .038 | .032 | .000 | .000 |
| SE1 | .036 | .036 | .000 | .000 |
| DM5 | .038 | .047 | .032 | .000 |
| DM3 | .041 | .050 | .029 | .000 |
| DM2 | .040 | .049 | .031 | .000 |
| DM1 | .039 | .047 | .034 | .000 |
| AA5 | .025 | .000 | .000 | .000 |
| AA4 | .027 | .000 | .000 | .000 |
| AA2 | .023 | .000 | .000 | .000 |
| AA1 | .022 | .000 | .000 | .000 |

##### Direct Effects - Standard Errors (Group number 1 - Default model)

|  | AA | SE | DM | AB |
| --- | --- | --- | --- | --- |
| SE | .049 | .000 | .000 | .000 |
| DM | .062 | .076 | .000 | .000 |
| AB | .065 | .086 | .070 | .000 |
| AA3 | .043 | .000 | .000 | .000 |
| AB5 | .000 | .000 | .000 | .063 |
| AB3 | .000 | .000 | .000 | .064 |
| AB2 | .000 | .000 | .000 | .061 |
| AB1 | .000 | .000 | .000 | .000 |
| SE5 | .000 | .094 | .000 | .000 |
| SE4 | .000 | .097 | .000 | .000 |
| SE3 | .000 | .087 | .000 | .000 |
| SE2 | .000 | .092 | .000 | .000 |
| SE1 | .000 | .000 | .000 | .000 |
| DM5 | .000 | .000 | .076 | .000 |
| DM3 | .000 | .000 | .077 | .000 |
| DM2 | .000 | .000 | .075 | .000 |
| DM1 | .000 | .000 | .000 | .000 |
| AA5 | .051 | .000 | .000 | .000 |
| AA4 | .052 | .000 | .000 | .000 |
| AA2 | .047 | .000 | .000 | .000 |
| AA1 | .000 | .000 | .000 | .000 |

##### Standardized Direct Effects - Standard Errors (Group number 1 - Default model)

|  | AA | SE | DM | AB |
| --- | --- | --- | --- | --- |
| SE | .047 | .000 | .000 | .000 |
| DM | .062 | .066 | .000 | .000 |
| AB | .070 | .063 | .059 | .000 |
| AA3 | .025 | .000 | .000 | .000 |
| AB5 | .000 | .000 | .000 | .030 |
| AB3 | .000 | .000 | .000 | .024 |
| AB2 | .000 | .000 | .000 | .031 |
| AB1 | .000 | .000 | .000 | .028 |
| SE5 | .000 | .025 | .000 | .000 |
| SE4 | .000 | .023 | .000 | .000 |
| SE3 | .000 | .032 | .000 | .000 |
| SE2 | .000 | .032 | .000 | .000 |
| SE1 | .000 | .036 | .000 | .000 |
| DM5 | .000 | .000 | .032 | .000 |
| DM3 | .000 | .000 | .029 | .000 |
| DM2 | .000 | .000 | .031 | .000 |
| DM1 | .000 | .000 | .034 | .000 |
| AA5 | .025 | .000 | .000 | .000 |
| AA4 | .027 | .000 | .000 | .000 |
| AA2 | .023 | .000 | .000 | .000 |
| AA1 | .022 | .000 | .000 | .000 |

##### Indirect Effects - Standard Errors (Group number 1 - Default model)

|  | AA | SE | DM | AB |
| --- | --- | --- | --- | --- |
| SE | .000 | .000 | .000 | .000 |
| DM | .029 | .000 | .000 | .000 |
| AB | .054 | .033 | .000 | .000 |
| AA3 | .000 | .000 | .000 | .000 |
| AB5 | .054 | .089 | .065 | .000 |
| AB3 | .059 | .091 | .075 | .000 |
| AB2 | .053 | .086 | .071 | .000 |
| AB1 | .055 | .089 | .070 | .000 |
| SE5 | .055 | .000 | .000 | .000 |
| SE4 | .058 | .000 | .000 | .000 |
| SE3 | .050 | .000 | .000 | .000 |
| SE2 | .054 | .000 | .000 | .000 |
| SE1 | .049 | .000 | .000 | .000 |
| DM5 | .052 | .074 | .000 | .000 |
| DM3 | .055 | .080 | .000 | .000 |
| DM2 | .053 | .078 | .000 | .000 |
| DM1 | .053 | .076 | .000 | .000 |
| AA5 | .000 | .000 | .000 | .000 |
| AA4 | .000 | .000 | .000 | .000 |
| AA2 | .000 | .000 | .000 | .000 |
| AA1 | .000 | .000 | .000 | .000 |

##### Standardized Indirect Effects - Standard Errors (Group number 1 - Default model)

|  | AA | SE | DM | AB |
| --- | --- | --- | --- | --- |
| SE | .000 | .000 | .000 | .000 |
| DM | .033 | .000 | .000 | .000 |
| AB | .051 | .027 | .000 | .000 |
| AA3 | .000 | .000 | .000 | .000 |
| AB5 | .041 | .051 | .042 | .000 |
| AB3 | .045 | .053 | .049 | .000 |
| AB2 | .041 | .050 | .047 | .000 |
| AB1 | .042 | .052 | .046 | .000 |
| SE5 | .041 | .000 | .000 | .000 |
| SE4 | .041 | .000 | .000 | .000 |
| SE3 | .037 | .000 | .000 | .000 |
| SE2 | .038 | .000 | .000 | .000 |
| SE1 | .036 | .000 | .000 | .000 |
| DM5 | .038 | .047 | .000 | .000 |
| DM3 | .041 | .050 | .000 | .000 |
| DM2 | .040 | .049 | .000 | .000 |
| DM1 | .039 | .047 | .000 | .000 |
| AA5 | .000 | .000 | .000 | .000 |
| AA4 | .000 | .000 | .000 | .000 |
| AA2 | .000 | .000 | .000 | .000 |
| AA1 | .000 | .000 | .000 | .000 |

##### Bootstrap Confidence (Group number 1 - Default model)

##### Percentile method (Group number 1 - Default model)

##### 95% confidence intervals (percentile method)

##### Scalar Estimates (Group number 1 - Default model)

##### Regression Weights: (Group number 1 - Default model)

| Parameter | | | Estimate | Lower | Upper | P |
| --- | --- | --- | --- | --- | --- | --- |
| SE | <--- | AA | .377 | .284 | .478 | .000 |
| DM | <--- | AA | .404 | .287 | .535 | .000 |
| DM | <--- | SE | .069 | -.083 | .220 | .358 |
| AB | <--- | DM | .434 | .306 | .582 | .000 |
| AB | <--- | SE | .493 | .330 | .673 | .000 |
| AB | <--- | AA | -.055 | -.187 | .072 | .379 |
| AA1 | <--- | AA | 1.000 | 1.000 | 1.000 | ... |
| AA2 | <--- | AA | .952 | .867 | 1.048 | .000 |
| AA4 | <--- | AA | .921 | .825 | 1.030 | .000 |
| AA5 | <--- | AA | .945 | .852 | 1.049 | .000 |
| DM1 | <--- | DM | 1.000 | 1.000 | 1.000 | ... |
| DM2 | <--- | DM | 1.026 | .889 | 1.186 | .000 |
| DM3 | <--- | DM | 1.051 | .911 | 1.216 | .000 |
| DM5 | <--- | DM | .984 | .847 | 1.138 | .000 |
| SE1 | <--- | SE | 1.000 | 1.000 | 1.000 | ... |
| SE2 | <--- | SE | 1.165 | .999 | 1.361 | .000 |
| SE3 | <--- | SE | 1.099 | .945 | 1.289 | .000 |
| SE4 | <--- | SE | 1.284 | 1.115 | 1.498 | .000 |
| SE5 | <--- | SE | 1.222 | 1.058 | 1.428 | .000 |
| AB1 | <--- | AB | 1.000 | 1.000 | 1.000 | ... |
| AB2 | <--- | AB | .975 | .861 | 1.103 | .000 |
| AB3 | <--- | AB | 1.064 | .950 | 1.200 | .000 |
| AB5 | <--- | AB | .964 | .846 | 1.098 | .000 |
| AA3 | <--- | AA | .920 | .838 | 1.009 | .000 |

##### Standardized Regression Weights: (Group number 1 - Default model)

| Parameter | | | Estimate | Lower | Upper | P |
| --- | --- | --- | --- | --- | --- | --- |
| SE | <--- | AA | .486 | .391 | .573 | .000 |
| DM | <--- | AA | .460 | .336 | .579 | .000 |
| DM | <--- | SE | .061 | -.071 | .191 | .358 |
| AB | <--- | DM | .409 | .292 | .524 | .000 |
| AB | <--- | SE | .411 | .284 | .535 | .000 |
| AB | <--- | AA | -.059 | -.198 | .078 | .379 |
| AA1 | <--- | AA | .818 | .770 | .859 | .000 |
| AA2 | <--- | AA | .782 | .736 | .824 | .000 |
| AA4 | <--- | AA | .752 | .698 | .801 | .000 |
| AA5 | <--- | AA | .782 | .729 | .829 | .000 |
| DM1 | <--- | DM | .705 | .635 | .769 | .000 |
| DM2 | <--- | DM | .739 | .678 | .796 | .000 |
| DM3 | <--- | DM | .761 | .702 | .814 | .000 |
| DM5 | <--- | DM | .705 | .639 | .767 | .000 |
| SE1 | <--- | SE | .636 | .562 | .703 | .000 |
| SE2 | <--- | SE | .716 | .649 | .775 | .000 |
| SE3 | <--- | SE | .696 | .629 | .755 | .000 |
| SE4 | <--- | SE | .805 | .757 | .847 | .000 |
| SE5 | <--- | SE | .788 | .736 | .834 | .000 |
| AB1 | <--- | AB | .758 | .698 | .811 | .000 |
| AB2 | <--- | AB | .737 | .671 | .794 | .000 |
| AB3 | <--- | AB | .806 | .755 | .851 | .000 |
| AB5 | <--- | AB | .725 | .663 | .780 | .000 |
| AA3 | <--- | AA | .748 | .697 | .794 | .000 |

##### Variances: (Group number 1 - Default model)

| Parameter | | | Estimate | Lower | Upper | P |
| --- | --- | --- | --- | --- | --- | --- |
| AA |  |  | 2.181 | 1.820 | 2.551 | .000 |
| e21 |  |  | 1.004 | .736 | 1.301 | .000 |
| e23 |  |  | 1.273 | .943 | 1.616 | .000 |
| e22 |  |  | 1.161 | .868 | 1.425 | .000 |
| e1 |  |  | 1.082 | .870 | 1.296 | .000 |
| e2 |  |  | 1.255 | 1.039 | 1.473 | .000 |
| e4 |  |  | 1.424 | 1.177 | 1.661 | .000 |
| e5 |  |  | 1.235 | .994 | 1.478 | .000 |
| e6 |  |  | 1.698 | 1.391 | 1.995 | .000 |
| e8 |  |  | 1.468 | 1.194 | 1.741 | .000 |
| e9 |  |  | 1.352 | 1.098 | 1.594 | .000 |
| e10 |  |  | 1.646 | 1.362 | 1.906 | .000 |
| e11 |  |  | 1.933 | 1.646 | 2.203 | .000 |
| e12 |  |  | 1.699 | 1.392 | 2.012 | .000 |
| e13 |  |  | 1.686 | 1.411 | 1.957 | .000 |
| e14 |  |  | 1.178 | .947 | 1.397 | .000 |
| e15 |  |  | 1.194 | .965 | 1.428 | .000 |
| e16 |  |  | 1.409 | 1.155 | 1.650 | .000 |
| e17 |  |  | 1.521 | 1.229 | 1.815 | .000 |
| e18 |  |  | 1.155 | .914 | 1.394 | .000 |
| e20 |  |  | 1.586 | 1.325 | 1.842 | .000 |
| e3 |  |  | 1.454 | 1.227 | 1.675 | .000 |

##### Squared Multiple Correlations: (Group number 1 - Default model)

| Parameter | | | Estimate | Lower | Upper | P |
| --- | --- | --- | --- | --- | --- | --- |
| SE |  |  | .236 | .153 | .329 | .000 |
| DM |  |  | .243 | .157 | .349 | .000 |
| AB |  |  | .388 | .295 | .500 | .000 |
| AA3 |  |  | .559 | .486 | .630 | .000 |
| AB5 |  |  | .526 | .440 | .608 | .000 |
| AB3 |  |  | .650 | .571 | .724 | .000 |
| AB2 |  |  | .543 | .450 | .631 | .000 |
| AB1 |  |  | .574 | .487 | .657 | .000 |
| SE5 |  |  | .622 | .542 | .696 | .000 |
| SE4 |  |  | .648 | .572 | .718 | .000 |
| SE3 |  |  | .485 | .395 | .569 | .000 |
| SE2 |  |  | .512 | .421 | .601 | .000 |
| SE1 |  |  | .405 | .315 | .494 | .000 |
| DM5 |  |  | .497 | .409 | .588 | .000 |
| DM3 |  |  | .579 | .492 | .663 | .000 |
| DM2 |  |  | .547 | .460 | .634 | .000 |
| DM1 |  |  | .498 | .404 | .591 | .000 |
| AA5 |  |  | .612 | .532 | .688 | .000 |
| AA4 |  |  | .565 | .488 | .642 | .000 |
| AA2 |  |  | .612 | .541 | .680 | .000 |
| AA1 |  |  | .668 | .593 | .738 | .000 |

##### User-defined estimands: (Group number 1 - Default model)

| Parameter | | | Estimate | Lower | Upper | P |
| --- | --- | --- | --- | --- | --- | --- |
| ind\_SE |  |  | .186 | .120 | .265 | .000 |
| ind\_DM |  |  | .176 | .108 | .259 | .000 |
| ind\_Serial |  |  | .011 | -.014 | .036 | .358 |
| c\_dir\_effect |  |  | -.055 | -.187 | .072 | .379 |

##### Matrices (Group number 1 - Default model)

##### Total Effects (Group number 1 - Default model)

##### Total Effects - Lower Bounds (PC) (Group number 1 - Default model)

|  | AA | SE | DM | AB |
| --- | --- | --- | --- | --- |
| SE | .284 | .000 | .000 | .000 |
| DM | .330 | -.083 | .000 | .000 |
| AB | .213 | .356 | .306 | .000 |
| AA3 | .838 | .000 | .000 | .000 |
| AB5 | .202 | .339 | .298 | .846 |
| AB3 | .225 | .385 | .323 | .950 |
| AB2 | .206 | .348 | .293 | .861 |
| AB1 | .213 | .356 | .306 | 1.000 |
| SE5 | .356 | 1.058 | .000 | .000 |
| SE4 | .372 | 1.115 | .000 | .000 |
| SE3 | .319 | .945 | .000 | .000 |
| SE2 | .337 | .999 | .000 | .000 |
| SE1 | .284 | 1.000 | .000 | .000 |
| DM5 | .325 | -.081 | .847 | .000 |
| DM3 | .344 | -.086 | .911 | .000 |
| DM2 | .343 | -.085 | .889 | .000 |
| DM1 | .330 | -.083 | 1.000 | .000 |
| AA5 | .852 | .000 | .000 | .000 |
| AA4 | .825 | .000 | .000 | .000 |
| AA2 | .867 | .000 | .000 | .000 |
| AA1 | 1.000 | .000 | .000 | .000 |

##### Total Effects - Upper Bounds (PC) (Group number 1 - Default model)

|  | AA | SE | DM | AB |
| --- | --- | --- | --- | --- |
| SE | .478 | .000 | .000 | .000 |
| DM | .539 | .220 | .000 | .000 |
| AB | .430 | .709 | .582 | .000 |
| AA3 | 1.009 | .000 | .000 | .000 |
| AB5 | .417 | .694 | .553 | 1.098 |
| AB3 | .458 | .740 | .621 | 1.200 |
| AB2 | .417 | .685 | .572 | 1.103 |
| AB1 | .430 | .709 | .582 | 1.000 |
| SE5 | .571 | 1.428 | .000 | .000 |
| SE4 | .603 | 1.498 | .000 | .000 |
| SE3 | .515 | 1.289 | .000 | .000 |
| SE2 | .550 | 1.361 | .000 | .000 |
| SE1 | .478 | 1.000 | .000 | .000 |
| DM5 | .528 | .214 | 1.138 | .000 |
| DM3 | .564 | .229 | 1.216 | .000 |
| DM2 | .549 | .227 | 1.186 | .000 |
| DM1 | .539 | .220 | 1.000 | .000 |
| AA5 | 1.049 | .000 | .000 | .000 |
| AA4 | 1.030 | .000 | .000 | .000 |
| AA2 | 1.048 | .000 | .000 | .000 |
| AA1 | 1.000 | .000 | .000 | .000 |

##### Total Effects - Two Tailed Significance (PC) (Group number 1 - Default model)

|  | AA | SE | DM | AB |
| --- | --- | --- | --- | --- |
| SE | .000 | ... | ... | ... |
| DM | .000 | .358 | ... | ... |
| AB | .000 | .000 | .000 | ... |
| AA3 | .000 | ... | ... | ... |
| AB5 | .000 | .000 | .000 | .000 |
| AB3 | .000 | .000 | .000 | .000 |
| AB2 | .000 | .000 | .000 | .000 |
| AB1 | .000 | .000 | .000 | ... |
| SE5 | .000 | .000 | ... | ... |
| SE4 | .000 | .000 | ... | ... |
| SE3 | .000 | .000 | ... | ... |
| SE2 | .000 | .000 | ... | ... |
| SE1 | .000 | ... | ... | ... |
| DM5 | .000 | .358 | .000 | ... |
| DM3 | .000 | .358 | .000 | ... |
| DM2 | .000 | .358 | .000 | ... |
| DM1 | .000 | .358 | ... | ... |
| AA5 | .000 | ... | ... | ... |
| AA4 | .000 | ... | ... | ... |
| AA2 | .000 | ... | ... | ... |
| AA1 | ... | ... | ... | ... |

##### Standardized Total Effects (Group number 1 - Default model)

##### Standardized Total Effects - Lower Bounds (PC) (Group number 1 - Default model)

|  | AA | SE | DM | AB |
| --- | --- | --- | --- | --- |
| SE | .391 | .000 | .000 | .000 |
| DM | .388 | -.071 | .000 | .000 |
| AB | .233 | .305 | .292 | .000 |
| AA3 | .697 | .000 | .000 | .000 |
| AB5 | .165 | .215 | .214 | .663 |
| AB3 | .185 | .245 | .234 | .755 |
| AB2 | .170 | .221 | .211 | .671 |
| AB1 | .175 | .227 | .219 | .698 |
| SE5 | .301 | .736 | .000 | .000 |
| SE4 | .309 | .757 | .000 | .000 |
| SE3 | .265 | .629 | .000 | .000 |
| SE2 | .272 | .649 | .000 | .000 |
| SE1 | .238 | .562 | .000 | .000 |
| DM5 | .269 | -.051 | .639 | .000 |
| DM3 | .290 | -.054 | .702 | .000 |
| DM2 | .283 | -.052 | .678 | .000 |
| DM1 | .268 | -.050 | .635 | .000 |
| AA5 | .729 | .000 | .000 | .000 |
| AA4 | .698 | .000 | .000 | .000 |
| AA2 | .736 | .000 | .000 | .000 |
| AA1 | .770 | .000 | .000 | .000 |

##### Standardized Total Effects - Upper Bounds (PC) (Group number 1 - Default model)

|  | AA | SE | DM | AB |
| --- | --- | --- | --- | --- |
| SE | .573 | .000 | .000 | .000 |
| DM | .586 | .191 | .000 | .000 |
| AB | .444 | .561 | .524 | .000 |
| AA3 | .794 | .000 | .000 | .000 |
| AB5 | .328 | .419 | .377 | .780 |
| AB3 | .363 | .455 | .427 | .851 |
| AB2 | .329 | .418 | .393 | .794 |
| AB1 | .342 | .430 | .401 | .811 |
| SE5 | .460 | .834 | .000 | .000 |
| SE4 | .468 | .847 | .000 | .000 |
| SE3 | .410 | .755 | .000 | .000 |
| SE2 | .420 | .775 | .000 | .000 |
| SE1 | .379 | .703 | .000 | .000 |
| DM5 | .419 | .134 | .767 | .000 |
| DM3 | .451 | .146 | .814 | .000 |
| DM2 | .442 | .142 | .796 | .000 |
| DM1 | .422 | .135 | .769 | .000 |
| AA5 | .829 | .000 | .000 | .000 |
| AA4 | .801 | .000 | .000 | .000 |
| AA2 | .824 | .000 | .000 | .000 |
| AA1 | .859 | .000 | .000 | .000 |

##### Standardized Total Effects - Two Tailed Significance (PC) (Group number 1 - Default model)

|  | AA | SE | DM | AB |
| --- | --- | --- | --- | --- |
| SE | .000 | ... | ... | ... |
| DM | .000 | .358 | ... | ... |
| AB | .000 | .000 | .000 | ... |
| AA3 | .000 | ... | ... | ... |
| AB5 | .000 | .000 | .000 | .000 |
| AB3 | .000 | .000 | .000 | .000 |
| AB2 | .000 | .000 | .000 | .000 |
| AB1 | .000 | .000 | .000 | .000 |
| SE5 | .000 | .000 | ... | ... |
| SE4 | .000 | .000 | ... | ... |
| SE3 | .000 | .000 | ... | ... |
| SE2 | .000 | .000 | ... | ... |
| SE1 | .000 | .000 | ... | ... |
| DM5 | .000 | .358 | .000 | ... |
| DM3 | .000 | .358 | .000 | ... |
| DM2 | .000 | .358 | .000 | ... |
| DM1 | .000 | .358 | .000 | ... |
| AA5 | .000 | ... | ... | ... |
| AA4 | .000 | ... | ... | ... |
| AA2 | .000 | ... | ... | ... |
| AA1 | .000 | ... | ... | ... |

##### Direct Effects (Group number 1 - Default model)

##### Direct Effects - Lower Bounds (PC) (Group number 1 - Default model)

|  | AA | SE | DM | AB |
| --- | --- | --- | --- | --- |
| SE | .284 | .000 | .000 | .000 |
| DM | .287 | -.083 | .000 | .000 |
| AB | -.187 | .330 | .306 | .000 |
| AA3 | .838 | .000 | .000 | .000 |
| AB5 | .000 | .000 | .000 | .846 |
| AB3 | .000 | .000 | .000 | .950 |
| AB2 | .000 | .000 | .000 | .861 |
| AB1 | .000 | .000 | .000 | 1.000 |
| SE5 | .000 | 1.058 | .000 | .000 |
| SE4 | .000 | 1.115 | .000 | .000 |
| SE3 | .000 | .945 | .000 | .000 |
| SE2 | .000 | .999 | .000 | .000 |
| SE1 | .000 | 1.000 | .000 | .000 |
| DM5 | .000 | .000 | .847 | .000 |
| DM3 | .000 | .000 | .911 | .000 |
| DM2 | .000 | .000 | .889 | .000 |
| DM1 | .000 | .000 | 1.000 | .000 |
| AA5 | .852 | .000 | .000 | .000 |
| AA4 | .825 | .000 | .000 | .000 |
| AA2 | .867 | .000 | .000 | .000 |
| AA1 | 1.000 | .000 | .000 | .000 |

##### Direct Effects - Upper Bounds (PC) (Group number 1 - Default model)

|  | AA | SE | DM | AB |
| --- | --- | --- | --- | --- |
| SE | .478 | .000 | .000 | .000 |
| DM | .535 | .220 | .000 | .000 |
| AB | .072 | .673 | .582 | .000 |
| AA3 | 1.009 | .000 | .000 | .000 |
| AB5 | .000 | .000 | .000 | 1.098 |
| AB3 | .000 | .000 | .000 | 1.200 |
| AB2 | .000 | .000 | .000 | 1.103 |
| AB1 | .000 | .000 | .000 | 1.000 |
| SE5 | .000 | 1.428 | .000 | .000 |
| SE4 | .000 | 1.498 | .000 | .000 |
| SE3 | .000 | 1.289 | .000 | .000 |
| SE2 | .000 | 1.361 | .000 | .000 |
| SE1 | .000 | 1.000 | .000 | .000 |
| DM5 | .000 | .000 | 1.138 | .000 |
| DM3 | .000 | .000 | 1.216 | .000 |
| DM2 | .000 | .000 | 1.186 | .000 |
| DM1 | .000 | .000 | 1.000 | .000 |
| AA5 | 1.049 | .000 | .000 | .000 |
| AA4 | 1.030 | .000 | .000 | .000 |
| AA2 | 1.048 | .000 | .000 | .000 |
| AA1 | 1.000 | .000 | .000 | .000 |

##### Direct Effects - Two Tailed Significance (PC) (Group number 1 - Default model)

|  | AA | SE | DM | AB |
| --- | --- | --- | --- | --- |
| SE | .000 | ... | ... | ... |
| DM | .000 | .358 | ... | ... |
| AB | .379 | .000 | .000 | ... |
| AA3 | .000 | ... | ... | ... |
| AB5 | ... | ... | ... | .000 |
| AB3 | ... | ... | ... | .000 |
| AB2 | ... | ... | ... | .000 |
| AB1 | ... | ... | ... | ... |
| SE5 | ... | .000 | ... | ... |
| SE4 | ... | .000 | ... | ... |
| SE3 | ... | .000 | ... | ... |
| SE2 | ... | .000 | ... | ... |
| SE1 | ... | ... | ... | ... |
| DM5 | ... | ... | .000 | ... |
| DM3 | ... | ... | .000 | ... |
| DM2 | ... | ... | .000 | ... |
| DM1 | ... | ... | ... | ... |
| AA5 | .000 | ... | ... | ... |
| AA4 | .000 | ... | ... | ... |
| AA2 | .000 | ... | ... | ... |
| AA1 | ... | ... | ... | ... |

##### Standardized Direct Effects (Group number 1 - Default model)

##### Standardized Direct Effects - Lower Bounds (PC) (Group number 1 - Default model)

|  | AA | SE | DM | AB |
| --- | --- | --- | --- | --- |
| SE | .391 | .000 | .000 | .000 |
| DM | .336 | -.071 | .000 | .000 |
| AB | -.198 | .284 | .292 | .000 |
| AA3 | .697 | .000 | .000 | .000 |
| AB5 | .000 | .000 | .000 | .663 |
| AB3 | .000 | .000 | .000 | .755 |
| AB2 | .000 | .000 | .000 | .671 |
| AB1 | .000 | .000 | .000 | .698 |
| SE5 | .000 | .736 | .000 | .000 |
| SE4 | .000 | .757 | .000 | .000 |
| SE3 | .000 | .629 | .000 | .000 |
| SE2 | .000 | .649 | .000 | .000 |
| SE1 | .000 | .562 | .000 | .000 |
| DM5 | .000 | .000 | .639 | .000 |
| DM3 | .000 | .000 | .702 | .000 |
| DM2 | .000 | .000 | .678 | .000 |
| DM1 | .000 | .000 | .635 | .000 |
| AA5 | .729 | .000 | .000 | .000 |
| AA4 | .698 | .000 | .000 | .000 |
| AA2 | .736 | .000 | .000 | .000 |
| AA1 | .770 | .000 | .000 | .000 |

##### Standardized Direct Effects - Upper Bounds (PC) (Group number 1 - Default model)

|  | AA | SE | DM | AB |
| --- | --- | --- | --- | --- |
| SE | .573 | .000 | .000 | .000 |
| DM | .579 | .191 | .000 | .000 |
| AB | .078 | .535 | .524 | .000 |
| AA3 | .794 | .000 | .000 | .000 |
| AB5 | .000 | .000 | .000 | .780 |
| AB3 | .000 | .000 | .000 | .851 |
| AB2 | .000 | .000 | .000 | .794 |
| AB1 | .000 | .000 | .000 | .811 |
| SE5 | .000 | .834 | .000 | .000 |
| SE4 | .000 | .847 | .000 | .000 |
| SE3 | .000 | .755 | .000 | .000 |
| SE2 | .000 | .775 | .000 | .000 |
| SE1 | .000 | .703 | .000 | .000 |
| DM5 | .000 | .000 | .767 | .000 |
| DM3 | .000 | .000 | .814 | .000 |
| DM2 | .000 | .000 | .796 | .000 |
| DM1 | .000 | .000 | .769 | .000 |
| AA5 | .829 | .000 | .000 | .000 |
| AA4 | .801 | .000 | .000 | .000 |
| AA2 | .824 | .000 | .000 | .000 |
| AA1 | .859 | .000 | .000 | .000 |

##### Standardized Direct Effects - Two Tailed Significance (PC) (Group number 1 - Default model)

|  | AA | SE | DM | AB |
| --- | --- | --- | --- | --- |
| SE | .000 | ... | ... | ... |
| DM | .000 | .358 | ... | ... |
| AB | .379 | .000 | .000 | ... |
| AA3 | .000 | ... | ... | ... |
| AB5 | ... | ... | ... | .000 |
| AB3 | ... | ... | ... | .000 |
| AB2 | ... | ... | ... | .000 |
| AB1 | ... | ... | ... | .000 |
| SE5 | ... | .000 | ... | ... |
| SE4 | ... | .000 | ... | ... |
| SE3 | ... | .000 | ... | ... |
| SE2 | ... | .000 | ... | ... |
| SE1 | ... | .000 | ... | ... |
| DM5 | ... | ... | .000 | ... |
| DM3 | ... | ... | .000 | ... |
| DM2 | ... | ... | .000 | ... |
| DM1 | ... | ... | .000 | ... |
| AA5 | .000 | ... | ... | ... |
| AA4 | .000 | ... | ... | ... |
| AA2 | .000 | ... | ... | ... |
| AA1 | .000 | ... | ... | ... |

##### Indirect Effects (Group number 1 - Default model)

##### Indirect Effects - Lower Bounds (PC) (Group number 1 - Default model)

|  | AA | SE | DM | AB |
| --- | --- | --- | --- | --- |
| SE | .000 | .000 | .000 | .000 |
| DM | -.031 | .000 | .000 | .000 |
| AB | .276 | -.038 | .000 | .000 |
| AA3 | .000 | .000 | .000 | .000 |
| AB5 | .202 | .339 | .298 | .000 |
| AB3 | .225 | .385 | .323 | .000 |
| AB2 | .206 | .348 | .293 | .000 |
| AB1 | .213 | .356 | .306 | .000 |
| SE5 | .356 | .000 | .000 | .000 |
| SE4 | .372 | .000 | .000 | .000 |
| SE3 | .319 | .000 | .000 | .000 |
| SE2 | .337 | .000 | .000 | .000 |
| SE1 | .284 | .000 | .000 | .000 |
| DM5 | .325 | -.081 | .000 | .000 |
| DM3 | .344 | -.086 | .000 | .000 |
| DM2 | .343 | -.085 | .000 | .000 |
| DM1 | .330 | -.083 | .000 | .000 |
| AA5 | .000 | .000 | .000 | .000 |
| AA4 | .000 | .000 | .000 | .000 |
| AA2 | .000 | .000 | .000 | .000 |
| AA1 | .000 | .000 | .000 | .000 |

##### Indirect Effects - Upper Bounds (PC) (Group number 1 - Default model)

|  | AA | SE | DM | AB |
| --- | --- | --- | --- | --- |
| SE | .000 | .000 | .000 | .000 |
| DM | .085 | .000 | .000 | .000 |
| AB | .486 | .094 | .000 | .000 |
| AA3 | .000 | .000 | .000 | .000 |
| AB5 | .417 | .694 | .553 | .000 |
| AB3 | .458 | .740 | .621 | .000 |
| AB2 | .417 | .685 | .572 | .000 |
| AB1 | .430 | .709 | .582 | .000 |
| SE5 | .571 | .000 | .000 | .000 |
| SE4 | .603 | .000 | .000 | .000 |
| SE3 | .515 | .000 | .000 | .000 |
| SE2 | .550 | .000 | .000 | .000 |
| SE1 | .478 | .000 | .000 | .000 |
| DM5 | .528 | .214 | .000 | .000 |
| DM3 | .564 | .229 | .000 | .000 |
| DM2 | .549 | .227 | .000 | .000 |
| DM1 | .539 | .220 | .000 | .000 |
| AA5 | .000 | .000 | .000 | .000 |
| AA4 | .000 | .000 | .000 | .000 |
| AA2 | .000 | .000 | .000 | .000 |
| AA1 | .000 | .000 | .000 | .000 |

##### Indirect Effects - Two Tailed Significance (PC) (Group number 1 - Default model)

|  | AA | SE | DM | AB |
| --- | --- | --- | --- | --- |
| SE | ... | ... | ... | ... |
| DM | .358 | ... | ... | ... |
| AB | .000 | .358 | ... | ... |
| AA3 | ... | ... | ... | ... |
| AB5 | .000 | .000 | .000 | ... |
| AB3 | .000 | .000 | .000 | ... |
| AB2 | .000 | .000 | .000 | ... |
| AB1 | .000 | .000 | .000 | ... |
| SE5 | .000 | ... | ... | ... |
| SE4 | .000 | ... | ... | ... |
| SE3 | .000 | ... | ... | ... |
| SE2 | .000 | ... | ... | ... |
| SE1 | .000 | ... | ... | ... |
| DM5 | .000 | .358 | ... | ... |
| DM3 | .000 | .358 | ... | ... |
| DM2 | .000 | .358 | ... | ... |
| DM1 | .000 | .358 | ... | ... |
| AA5 | ... | ... | ... | ... |
| AA4 | ... | ... | ... | ... |
| AA2 | ... | ... | ... | ... |
| AA1 | ... | ... | ... | ... |

##### Standardized Indirect Effects (Group number 1 - Default model)

##### Standardized Indirect Effects - Lower Bounds (PC) (Group number 1 - Default model)

|  | AA | SE | DM | AB |
| --- | --- | --- | --- | --- |
| SE | .000 | .000 | .000 | .000 |
| DM | -.036 | .000 | .000 | .000 |
| AB | .306 | -.031 | .000 | .000 |
| AA3 | .000 | .000 | .000 | .000 |
| AB5 | .165 | .215 | .214 | .000 |
| AB3 | .185 | .245 | .234 | .000 |
| AB2 | .170 | .221 | .211 | .000 |
| AB1 | .175 | .227 | .219 | .000 |
| SE5 | .301 | .000 | .000 | .000 |
| SE4 | .309 | .000 | .000 | .000 |
| SE3 | .265 | .000 | .000 | .000 |
| SE2 | .272 | .000 | .000 | .000 |
| SE1 | .238 | .000 | .000 | .000 |
| DM5 | .269 | -.051 | .000 | .000 |
| DM3 | .290 | -.054 | .000 | .000 |
| DM2 | .283 | -.052 | .000 | .000 |
| DM1 | .268 | -.050 | .000 | .000 |
| AA5 | .000 | .000 | .000 | .000 |
| AA4 | .000 | .000 | .000 | .000 |
| AA2 | .000 | .000 | .000 | .000 |
| AA1 | .000 | .000 | .000 | .000 |

##### Standardized Indirect Effects - Upper Bounds (PC) (Group number 1 - Default model)

|  | AA | SE | DM | AB |
| --- | --- | --- | --- | --- |
| SE | .000 | .000 | .000 | .000 |
| DM | .096 | .000 | .000 | .000 |
| AB | .504 | .077 | .000 | .000 |
| AA3 | .000 | .000 | .000 | .000 |
| AB5 | .328 | .419 | .377 | .000 |
| AB3 | .363 | .455 | .427 | .000 |
| AB2 | .329 | .418 | .393 | .000 |
| AB1 | .342 | .430 | .401 | .000 |
| SE5 | .460 | .000 | .000 | .000 |
| SE4 | .468 | .000 | .000 | .000 |
| SE3 | .410 | .000 | .000 | .000 |
| SE2 | .420 | .000 | .000 | .000 |
| SE1 | .379 | .000 | .000 | .000 |
| DM5 | .419 | .134 | .000 | .000 |
| DM3 | .451 | .146 | .000 | .000 |
| DM2 | .442 | .142 | .000 | .000 |
| DM1 | .422 | .135 | .000 | .000 |
| AA5 | .000 | .000 | .000 | .000 |
| AA4 | .000 | .000 | .000 | .000 |
| AA2 | .000 | .000 | .000 | .000 |
| AA1 | .000 | .000 | .000 | .000 |

##### Standardized Indirect Effects - Two Tailed Significance (PC) (Group number 1 - Default model)

|  | AA | SE | DM | AB |
| --- | --- | --- | --- | --- |
| SE | ... | ... | ... | ... |
| DM | .358 | ... | ... | ... |
| AB | .000 | .358 | ... | ... |
| AA3 | ... | ... | ... | ... |
| AB5 | .000 | .000 | .000 | ... |
| AB3 | .000 | .000 | .000 | ... |
| AB2 | .000 | .000 | .000 | ... |
| AB1 | .000 | .000 | .000 | ... |
| SE5 | .000 | ... | ... | ... |
| SE4 | .000 | ... | ... | ... |
| SE3 | .000 | ... | ... | ... |
| SE2 | .000 | ... | ... | ... |
| SE1 | .000 | ... | ... | ... |
| DM5 | .000 | .358 | ... | ... |
| DM3 | .000 | .358 | ... | ... |
| DM2 | .000 | .358 | ... | ... |
| DM1 | .000 | .358 | ... | ... |
| AA5 | ... | ... | ... | ... |
| AA4 | ... | ... | ... | ... |
| AA2 | ... | ... | ... | ... |
| AA1 | ... | ... | ... | ... |

##### Bias-corrected percentile method (Group number 1 - Default model)

##### 95% confidence intervals (bias-corrected percentile method)

##### Scalar Estimates (Group number 1 - Default model)

##### Regression Weights: (Group number 1 - Default model)

| Parameter | | | Estimate | Lower | Upper | P |
| --- | --- | --- | --- | --- | --- | --- |
| SE | <--- | AA | .377 | .288 | .483 | .000 |
| DM | <--- | AA | .404 | .285 | .533 | .000 |
| DM | <--- | SE | .069 | -.082 | .221 | .349 |
| AB | <--- | DM | .434 | .305 | .581 | .000 |
| AB | <--- | SE | .493 | .326 | .669 | .000 |
| AB | <--- | AA | -.055 | -.183 | .076 | .419 |
| AA1 | <--- | AA | 1.000 | 1.000 | 1.000 | ... |
| AA2 | <--- | AA | .952 | .869 | 1.049 | .000 |
| AA4 | <--- | AA | .921 | .823 | 1.028 | .000 |
| AA5 | <--- | AA | .945 | .847 | 1.047 | .001 |
| DM1 | <--- | DM | 1.000 | 1.000 | 1.000 | ... |
| DM2 | <--- | DM | 1.026 | .891 | 1.190 | .000 |
| DM3 | <--- | DM | 1.051 | .910 | 1.215 | .000 |
| DM5 | <--- | DM | .984 | .847 | 1.138 | .000 |
| SE1 | <--- | SE | 1.000 | 1.000 | 1.000 | ... |
| SE2 | <--- | SE | 1.165 | .997 | 1.360 | .000 |
| SE3 | <--- | SE | 1.099 | .944 | 1.286 | .000 |
| SE4 | <--- | SE | 1.284 | 1.110 | 1.492 | .000 |
| SE5 | <--- | SE | 1.222 | 1.056 | 1.427 | .000 |
| AB1 | <--- | AB | 1.000 | 1.000 | 1.000 | ... |
| AB2 | <--- | AB | .975 | .862 | 1.104 | .000 |
| AB3 | <--- | AB | 1.064 | .950 | 1.200 | .000 |
| AB5 | <--- | AB | .964 | .846 | 1.097 | .000 |
| AA3 | <--- | AA | .920 | .837 | 1.006 | .000 |

##### Standardized Regression Weights: (Group number 1 - Default model)

| Parameter | | | Estimate | Lower | Upper | P |
| --- | --- | --- | --- | --- | --- | --- |
| SE | <--- | AA | .486 | .394 | .576 | .000 |
| DM | <--- | AA | .460 | .332 | .576 | .001 |
| DM | <--- | SE | .061 | -.071 | .191 | .358 |
| AB | <--- | DM | .409 | .289 | .523 | .000 |
| AB | <--- | SE | .411 | .280 | .532 | .000 |
| AB | <--- | AA | -.059 | -.193 | .082 | .423 |
| AA1 | <--- | AA | .818 | .770 | .859 | .000 |
| AA2 | <--- | AA | .782 | .736 | .824 | .000 |
| AA4 | <--- | AA | .752 | .694 | .798 | .001 |
| AA5 | <--- | AA | .782 | .726 | .827 | .001 |
| DM1 | <--- | DM | .705 | .634 | .767 | .000 |
| DM2 | <--- | DM | .739 | .677 | .795 | .001 |
| DM3 | <--- | DM | .761 | .702 | .815 | .000 |
| DM5 | <--- | DM | .705 | .638 | .765 | .000 |
| SE1 | <--- | SE | .636 | .561 | .703 | .000 |
| SE2 | <--- | SE | .716 | .649 | .775 | .000 |
| SE3 | <--- | SE | .696 | .627 | .753 | .000 |
| SE4 | <--- | SE | .805 | .756 | .847 | .000 |
| SE5 | <--- | SE | .788 | .735 | .833 | .000 |
| AB1 | <--- | AB | .758 | .697 | .810 | .000 |
| AB2 | <--- | AB | .737 | .671 | .794 | .000 |
| AB3 | <--- | AB | .806 | .755 | .850 | .000 |
| AB5 | <--- | AB | .725 | .663 | .778 | .000 |
| AA3 | <--- | AA | .748 | .695 | .792 | .001 |

##### Variances: (Group number 1 - Default model)

| Parameter | | | Estimate | Lower | Upper | P |
| --- | --- | --- | --- | --- | --- | --- |
| AA |  |  | 2.181 | 1.842 | 2.568 | .000 |
| e21 |  |  | 1.004 | .749 | 1.322 | .000 |
| e23 |  |  | 1.273 | .970 | 1.652 | .000 |
| e22 |  |  | 1.161 | .898 | 1.469 | .000 |
| e1 |  |  | 1.082 | .881 | 1.308 | .000 |
| e2 |  |  | 1.255 | 1.050 | 1.482 | .000 |
| e4 |  |  | 1.424 | 1.202 | 1.680 | .000 |
| e5 |  |  | 1.235 | 1.013 | 1.507 | .000 |
| e6 |  |  | 1.698 | 1.417 | 2.021 | .000 |
| e8 |  |  | 1.468 | 1.209 | 1.760 | .000 |
| e9 |  |  | 1.352 | 1.112 | 1.605 | .000 |
| e10 |  |  | 1.646 | 1.383 | 1.924 | .000 |
| e11 |  |  | 1.933 | 1.663 | 2.220 | .000 |
| e12 |  |  | 1.699 | 1.415 | 2.041 | .000 |
| e13 |  |  | 1.686 | 1.430 | 1.981 | .000 |
| e14 |  |  | 1.178 | .953 | 1.405 | .000 |
| e15 |  |  | 1.194 | .980 | 1.444 | .000 |
| e16 |  |  | 1.409 | 1.164 | 1.660 | .000 |
| e17 |  |  | 1.521 | 1.247 | 1.837 | .000 |
| e18 |  |  | 1.155 | .928 | 1.414 | .000 |
| e20 |  |  | 1.586 | 1.342 | 1.856 | .000 |
| e3 |  |  | 1.454 | 1.247 | 1.700 | .000 |

##### Squared Multiple Correlations: (Group number 1 - Default model)

| Parameter | | | Estimate | Lower | Upper | P |
| --- | --- | --- | --- | --- | --- | --- |
| SE |  |  | .236 | .155 | .332 | .000 |
| DM |  |  | .243 | .149 | .338 | .001 |
| AB |  |  | .388 | .283 | .487 | .001 |
| AA3 |  |  | .559 | .484 | .627 | .001 |
| AB5 |  |  | .526 | .439 | .606 | .000 |
| AB3 |  |  | .650 | .570 | .723 | .000 |
| AB2 |  |  | .543 | .450 | .631 | .000 |
| AB1 |  |  | .574 | .486 | .656 | .000 |
| SE5 |  |  | .622 | .540 | .694 | .000 |
| SE4 |  |  | .648 | .572 | .718 | .000 |
| SE3 |  |  | .485 | .393 | .567 | .000 |
| SE2 |  |  | .512 | .421 | .601 | .000 |
| SE1 |  |  | .405 | .315 | .494 | .000 |
| DM5 |  |  | .497 | .407 | .585 | .000 |
| DM3 |  |  | .579 | .493 | .664 | .000 |
| DM2 |  |  | .547 | .458 | .632 | .001 |
| DM1 |  |  | .498 | .402 | .588 | .000 |
| AA5 |  |  | .612 | .527 | .684 | .001 |
| AA4 |  |  | .565 | .482 | .637 | .001 |
| AA2 |  |  | .612 | .541 | .680 | .000 |
| AA1 |  |  | .668 | .593 | .738 | .000 |

##### User-defined estimands: (Group number 1 - Default model)

| Parameter | | | Estimate | Lower | Upper | P |
| --- | --- | --- | --- | --- | --- | --- |
| ind\_SE |  |  | .186 | .122 | .271 | .000 |
| ind\_DM |  |  | .176 | .109 | .261 | .000 |
| ind\_Serial |  |  | .011 | -.013 | .037 | .318 |
| c\_dir\_effect |  |  | -.055 | -.183 | .076 | .419 |

##### Matrices (Group number 1 - Default model)

##### Total Effects (Group number 1 - Default model)

##### Total Effects - Lower Bounds (BC) (Group number 1 - Default model)

|  | AA | SE | DM | AB |
| --- | --- | --- | --- | --- |
| SE | .288 | .000 | .000 | .000 |
| DM | .330 | -.082 | .000 | .000 |
| AB | .216 | .353 | .305 | .000 |
| AA3 | .837 | .000 | .000 | .000 |
| AB5 | .205 | .334 | .299 | .846 |
| AB3 | .229 | .383 | .322 | .950 |
| AB2 | .210 | .347 | .293 | .862 |
| AB1 | .216 | .353 | .305 | 1.000 |
| SE5 | .360 | 1.056 | .000 | .000 |
| SE4 | .376 | 1.110 | .000 | .000 |
| SE3 | .323 | .944 | .000 | .000 |
| SE2 | .340 | .997 | .000 | .000 |
| SE1 | .288 | 1.000 | .000 | .000 |
| DM5 | .325 | -.081 | .847 | .000 |
| DM3 | .342 | -.086 | .910 | .000 |
| DM2 | .342 | -.084 | .891 | .000 |
| DM1 | .330 | -.082 | 1.000 | .000 |
| AA5 | .847 | .000 | .000 | .000 |
| AA4 | .823 | .000 | .000 | .000 |
| AA2 | .869 | .000 | .000 | .000 |
| AA1 | 1.000 | .000 | .000 | .000 |

##### Total Effects - Upper Bounds (BC) (Group number 1 - Default model)

|  | AA | SE | DM | AB |
| --- | --- | --- | --- | --- |
| SE | .483 | .000 | .000 | .000 |
| DM | .539 | .221 | .000 | .000 |
| AB | .435 | .706 | .581 | .000 |
| AA3 | 1.006 | .000 | .000 | .000 |
| AB5 | .420 | .687 | .554 | 1.097 |
| AB3 | .462 | .736 | .619 | 1.200 |
| AB2 | .420 | .685 | .572 | 1.104 |
| AB1 | .435 | .706 | .581 | 1.000 |
| SE5 | .575 | 1.427 | .000 | .000 |
| SE4 | .609 | 1.492 | .000 | .000 |
| SE3 | .517 | 1.286 | .000 | .000 |
| SE2 | .553 | 1.360 | .000 | .000 |
| SE1 | .483 | 1.000 | .000 | .000 |
| DM5 | .529 | .214 | 1.138 | .000 |
| DM3 | .563 | .230 | 1.215 | .000 |
| DM2 | .549 | .228 | 1.190 | .000 |
| DM1 | .539 | .221 | 1.000 | .000 |
| AA5 | 1.047 | .000 | .000 | .000 |
| AA4 | 1.028 | .000 | .000 | .000 |
| AA2 | 1.049 | .000 | .000 | .000 |
| AA1 | 1.000 | .000 | .000 | .000 |

##### Total Effects - Two Tailed Significance (BC) (Group number 1 - Default model)

|  | AA | SE | DM | AB |
| --- | --- | --- | --- | --- |
| SE | .000 | ... | ... | ... |
| DM | .000 | .349 | ... | ... |
| AB | .000 | .000 | .000 | ... |
| AA3 | .000 | ... | ... | ... |
| AB5 | .000 | .000 | .000 | .000 |
| AB3 | .000 | .000 | .000 | .000 |
| AB2 | .000 | .000 | .000 | .000 |
| AB1 | .000 | .000 | .000 | ... |
| SE5 | .000 | .000 | ... | ... |
| SE4 | .000 | .000 | ... | ... |
| SE3 | .000 | .000 | ... | ... |
| SE2 | .000 | .000 | ... | ... |
| SE1 | .000 | ... | ... | ... |
| DM5 | .000 | .353 | .000 | ... |
| DM3 | .000 | .356 | .000 | ... |
| DM2 | .000 | .353 | .000 | ... |
| DM1 | .000 | .349 | ... | ... |
| AA5 | .001 | ... | ... | ... |
| AA4 | .000 | ... | ... | ... |
| AA2 | .000 | ... | ... | ... |
| AA1 | ... | ... | ... | ... |

##### Standardized Total Effects (Group number 1 - Default model)

##### Standardized Total Effects - Lower Bounds (BC) (Group number 1 - Default model)

|  | AA | SE | DM | AB |
| --- | --- | --- | --- | --- |
| SE | .394 | .000 | .000 | .000 |
| DM | .386 | -.071 | .000 | .000 |
| AB | .236 | .304 | .289 | .000 |
| AA3 | .695 | .000 | .000 | .000 |
| AB5 | .166 | .215 | .213 | .663 |
| AB3 | .189 | .243 | .233 | .755 |
| AB2 | .173 | .219 | .211 | .671 |
| AB1 | .178 | .226 | .219 | .697 |
| SE5 | .303 | .735 | .000 | .000 |
| SE4 | .312 | .756 | .000 | .000 |
| SE3 | .267 | .627 | .000 | .000 |
| SE2 | .275 | .649 | .000 | .000 |
| SE1 | .243 | .561 | .000 | .000 |
| DM5 | .269 | -.050 | .638 | .000 |
| DM3 | .289 | -.053 | .702 | .000 |
| DM2 | .282 | -.051 | .677 | .000 |
| DM1 | .268 | -.049 | .634 | .000 |
| AA5 | .726 | .000 | .000 | .000 |
| AA4 | .694 | .000 | .000 | .000 |
| AA2 | .736 | .000 | .000 | .000 |
| AA1 | .770 | .000 | .000 | .000 |

##### Standardized Total Effects - Upper Bounds (BC) (Group number 1 - Default model)

|  | AA | SE | DM | AB |
| --- | --- | --- | --- | --- |
| SE | .576 | .000 | .000 | .000 |
| DM | .584 | .191 | .000 | .000 |
| AB | .448 | .559 | .523 | .000 |
| AA3 | .792 | .000 | .000 | .000 |
| AB5 | .329 | .419 | .376 | .778 |
| AB3 | .366 | .452 | .425 | .850 |
| AB2 | .331 | .417 | .393 | .794 |
| AB1 | .346 | .430 | .400 | .810 |
| SE5 | .462 | .833 | .000 | .000 |
| SE4 | .470 | .847 | .000 | .000 |
| SE3 | .412 | .753 | .000 | .000 |
| SE2 | .423 | .775 | .000 | .000 |
| SE1 | .384 | .703 | .000 | .000 |
| DM5 | .418 | .134 | .765 | .000 |
| DM3 | .450 | .146 | .815 | .000 |
| DM2 | .441 | .144 | .795 | .000 |
| DM1 | .422 | .136 | .767 | .000 |
| AA5 | .827 | .000 | .000 | .000 |
| AA4 | .798 | .000 | .000 | .000 |
| AA2 | .824 | .000 | .000 | .000 |
| AA1 | .859 | .000 | .000 | .000 |

##### Standardized Total Effects - Two Tailed Significance (BC) (Group number 1 - Default model)

|  | AA | SE | DM | AB |
| --- | --- | --- | --- | --- |
| SE | .000 | ... | ... | ... |
| DM | .000 | .358 | ... | ... |
| AB | .000 | .000 | .000 | ... |
| AA3 | .001 | ... | ... | ... |
| AB5 | .000 | .000 | .000 | .000 |
| AB3 | .000 | .000 | .000 | .000 |
| AB2 | .000 | .000 | .000 | .000 |
| AB1 | .000 | .000 | .000 | .000 |
| SE5 | .000 | .000 | ... | ... |
| SE4 | .000 | .000 | ... | ... |
| SE3 | .000 | .000 | ... | ... |
| SE2 | .000 | .000 | ... | ... |
| SE1 | .000 | .000 | ... | ... |
| DM5 | .000 | .356 | .000 | ... |
| DM3 | .000 | .353 | .000 | ... |
| DM2 | .000 | .350 | .001 | ... |
| DM1 | .000 | .350 | .000 | ... |
| AA5 | .001 | ... | ... | ... |
| AA4 | .001 | ... | ... | ... |
| AA2 | .000 | ... | ... | ... |
| AA1 | .000 | ... | ... | ... |

##### Direct Effects (Group number 1 - Default model)

##### Direct Effects - Lower Bounds (BC) (Group number 1 - Default model)

|  | AA | SE | DM | AB |
| --- | --- | --- | --- | --- |
| SE | .288 | .000 | .000 | .000 |
| DM | .285 | -.082 | .000 | .000 |
| AB | -.183 | .326 | .305 | .000 |
| AA3 | .837 | .000 | .000 | .000 |
| AB5 | .000 | .000 | .000 | .846 |
| AB3 | .000 | .000 | .000 | .950 |
| AB2 | .000 | .000 | .000 | .862 |
| AB1 | .000 | .000 | .000 | 1.000 |
| SE5 | .000 | 1.056 | .000 | .000 |
| SE4 | .000 | 1.110 | .000 | .000 |
| SE3 | .000 | .944 | .000 | .000 |
| SE2 | .000 | .997 | .000 | .000 |
| SE1 | .000 | 1.000 | .000 | .000 |
| DM5 | .000 | .000 | .847 | .000 |
| DM3 | .000 | .000 | .910 | .000 |
| DM2 | .000 | .000 | .891 | .000 |
| DM1 | .000 | .000 | 1.000 | .000 |
| AA5 | .847 | .000 | .000 | .000 |
| AA4 | .823 | .000 | .000 | .000 |
| AA2 | .869 | .000 | .000 | .000 |
| AA1 | 1.000 | .000 | .000 | .000 |

##### Direct Effects - Upper Bounds (BC) (Group number 1 - Default model)

|  | AA | SE | DM | AB |
| --- | --- | --- | --- | --- |
| SE | .483 | .000 | .000 | .000 |
| DM | .533 | .221 | .000 | .000 |
| AB | .076 | .669 | .581 | .000 |
| AA3 | 1.006 | .000 | .000 | .000 |
| AB5 | .000 | .000 | .000 | 1.097 |
| AB3 | .000 | .000 | .000 | 1.200 |
| AB2 | .000 | .000 | .000 | 1.104 |
| AB1 | .000 | .000 | .000 | 1.000 |
| SE5 | .000 | 1.427 | .000 | .000 |
| SE4 | .000 | 1.492 | .000 | .000 |
| SE3 | .000 | 1.286 | .000 | .000 |
| SE2 | .000 | 1.360 | .000 | .000 |
| SE1 | .000 | 1.000 | .000 | .000 |
| DM5 | .000 | .000 | 1.138 | .000 |
| DM3 | .000 | .000 | 1.215 | .000 |
| DM2 | .000 | .000 | 1.190 | .000 |
| DM1 | .000 | .000 | 1.000 | .000 |
| AA5 | 1.047 | .000 | .000 | .000 |
| AA4 | 1.028 | .000 | .000 | .000 |
| AA2 | 1.049 | .000 | .000 | .000 |
| AA1 | 1.000 | .000 | .000 | .000 |

##### Direct Effects - Two Tailed Significance (BC) (Group number 1 - Default model)

|  | AA | SE | DM | AB |
| --- | --- | --- | --- | --- |
| SE | .000 | ... | ... | ... |
| DM | .000 | .349 | ... | ... |
| AB | .419 | .000 | .000 | ... |
| AA3 | .000 | ... | ... | ... |
| AB5 | ... | ... | ... | .000 |
| AB3 | ... | ... | ... | .000 |
| AB2 | ... | ... | ... | .000 |
| AB1 | ... | ... | ... | ... |
| SE5 | ... | .000 | ... | ... |
| SE4 | ... | .000 | ... | ... |
| SE3 | ... | .000 | ... | ... |
| SE2 | ... | .000 | ... | ... |
| SE1 | ... | ... | ... | ... |
| DM5 | ... | ... | .000 | ... |
| DM3 | ... | ... | .000 | ... |
| DM2 | ... | ... | .000 | ... |
| DM1 | ... | ... | ... | ... |
| AA5 | .001 | ... | ... | ... |
| AA4 | .000 | ... | ... | ... |
| AA2 | .000 | ... | ... | ... |
| AA1 | ... | ... | ... | ... |

##### Standardized Direct Effects (Group number 1 - Default model)

##### Standardized Direct Effects - Lower Bounds (BC) (Group number 1 - Default model)

|  | AA | SE | DM | AB |
| --- | --- | --- | --- | --- |
| SE | .394 | .000 | .000 | .000 |
| DM | .332 | -.071 | .000 | .000 |
| AB | -.193 | .280 | .289 | .000 |
| AA3 | .695 | .000 | .000 | .000 |
| AB5 | .000 | .000 | .000 | .663 |
| AB3 | .000 | .000 | .000 | .755 |
| AB2 | .000 | .000 | .000 | .671 |
| AB1 | .000 | .000 | .000 | .697 |
| SE5 | .000 | .735 | .000 | .000 |
| SE4 | .000 | .756 | .000 | .000 |
| SE3 | .000 | .627 | .000 | .000 |
| SE2 | .000 | .649 | .000 | .000 |
| SE1 | .000 | .561 | .000 | .000 |
| DM5 | .000 | .000 | .638 | .000 |
| DM3 | .000 | .000 | .702 | .000 |
| DM2 | .000 | .000 | .677 | .000 |
| DM1 | .000 | .000 | .634 | .000 |
| AA5 | .726 | .000 | .000 | .000 |
| AA4 | .694 | .000 | .000 | .000 |
| AA2 | .736 | .000 | .000 | .000 |
| AA1 | .770 | .000 | .000 | .000 |

##### Standardized Direct Effects - Upper Bounds (BC) (Group number 1 - Default model)

|  | AA | SE | DM | AB |
| --- | --- | --- | --- | --- |
| SE | .576 | .000 | .000 | .000 |
| DM | .576 | .191 | .000 | .000 |
| AB | .082 | .532 | .523 | .000 |
| AA3 | .792 | .000 | .000 | .000 |
| AB5 | .000 | .000 | .000 | .778 |
| AB3 | .000 | .000 | .000 | .850 |
| AB2 | .000 | .000 | .000 | .794 |
| AB1 | .000 | .000 | .000 | .810 |
| SE5 | .000 | .833 | .000 | .000 |
| SE4 | .000 | .847 | .000 | .000 |
| SE3 | .000 | .753 | .000 | .000 |
| SE2 | .000 | .775 | .000 | .000 |
| SE1 | .000 | .703 | .000 | .000 |
| DM5 | .000 | .000 | .765 | .000 |
| DM3 | .000 | .000 | .815 | .000 |
| DM2 | .000 | .000 | .795 | .000 |
| DM1 | .000 | .000 | .767 | .000 |
| AA5 | .827 | .000 | .000 | .000 |
| AA4 | .798 | .000 | .000 | .000 |
| AA2 | .824 | .000 | .000 | .000 |
| AA1 | .859 | .000 | .000 | .000 |

##### Standardized Direct Effects - Two Tailed Significance (BC) (Group number 1 - Default model)

|  | AA | SE | DM | AB |
| --- | --- | --- | --- | --- |
| SE | .000 | ... | ... | ... |
| DM | .001 | .358 | ... | ... |
| AB | .423 | .000 | .000 | ... |
| AA3 | .001 | ... | ... | ... |
| AB5 | ... | ... | ... | .000 |
| AB3 | ... | ... | ... | .000 |
| AB2 | ... | ... | ... | .000 |
| AB1 | ... | ... | ... | .000 |
| SE5 | ... | .000 | ... | ... |
| SE4 | ... | .000 | ... | ... |
| SE3 | ... | .000 | ... | ... |
| SE2 | ... | .000 | ... | ... |
| SE1 | ... | .000 | ... | ... |
| DM5 | ... | ... | .000 | ... |
| DM3 | ... | ... | .000 | ... |
| DM2 | ... | ... | .001 | ... |
| DM1 | ... | ... | .000 | ... |
| AA5 | .001 | ... | ... | ... |
| AA4 | .001 | ... | ... | ... |
| AA2 | .000 | ... | ... | ... |
| AA1 | .000 | ... | ... | ... |

##### Indirect Effects (Group number 1 - Default model)

##### Indirect Effects - Lower Bounds (BC) (Group number 1 - Default model)

|  | AA | SE | DM | AB |
| --- | --- | --- | --- | --- |
| SE | .000 | .000 | .000 | .000 |
| DM | -.030 | .000 | .000 | .000 |
| AB | .276 | -.035 | .000 | .000 |
| AA3 | .000 | .000 | .000 | .000 |
| AB5 | .205 | .334 | .299 | .000 |
| AB3 | .229 | .383 | .322 | .000 |
| AB2 | .210 | .347 | .293 | .000 |
| AB1 | .216 | .353 | .305 | .000 |
| SE5 | .360 | .000 | .000 | .000 |
| SE4 | .376 | .000 | .000 | .000 |
| SE3 | .323 | .000 | .000 | .000 |
| SE2 | .340 | .000 | .000 | .000 |
| SE1 | .288 | .000 | .000 | .000 |
| DM5 | .325 | -.081 | .000 | .000 |
| DM3 | .342 | -.086 | .000 | .000 |
| DM2 | .342 | -.084 | .000 | .000 |
| DM1 | .330 | -.082 | .000 | .000 |
| AA5 | .000 | .000 | .000 | .000 |
| AA4 | .000 | .000 | .000 | .000 |
| AA2 | .000 | .000 | .000 | .000 |
| AA1 | .000 | .000 | .000 | .000 |

##### Indirect Effects - Upper Bounds (BC) (Group number 1 - Default model)

|  | AA | SE | DM | AB |
| --- | --- | --- | --- | --- |
| SE | .000 | .000 | .000 | .000 |
| DM | .087 | .000 | .000 | .000 |
| AB | .486 | .096 | .000 | .000 |
| AA3 | .000 | .000 | .000 | .000 |
| AB5 | .420 | .687 | .554 | .000 |
| AB3 | .462 | .736 | .619 | .000 |
| AB2 | .420 | .685 | .572 | .000 |
| AB1 | .435 | .706 | .581 | .000 |
| SE5 | .575 | .000 | .000 | .000 |
| SE4 | .609 | .000 | .000 | .000 |
| SE3 | .517 | .000 | .000 | .000 |
| SE2 | .553 | .000 | .000 | .000 |
| SE1 | .483 | .000 | .000 | .000 |
| DM5 | .529 | .214 | .000 | .000 |
| DM3 | .563 | .230 | .000 | .000 |
| DM2 | .549 | .228 | .000 | .000 |
| DM1 | .539 | .221 | .000 | .000 |
| AA5 | .000 | .000 | .000 | .000 |
| AA4 | .000 | .000 | .000 | .000 |
| AA2 | .000 | .000 | .000 | .000 |
| AA1 | .000 | .000 | .000 | .000 |

##### Indirect Effects - Two Tailed Significance (BC) (Group number 1 - Default model)

|  | AA | SE | DM | AB |
| --- | --- | --- | --- | --- |
| SE | ... | ... | ... | ... |
| DM | .331 | ... | ... | ... |
| AB | .000 | .329 | ... | ... |
| AA3 | ... | ... | ... | ... |
| AB5 | .000 | .000 | .000 | ... |
| AB3 | .000 | .000 | .000 | ... |
| AB2 | .000 | .000 | .000 | ... |
| AB1 | .000 | .000 | .000 | ... |
| SE5 | .000 | ... | ... | ... |
| SE4 | .000 | ... | ... | ... |
| SE3 | .000 | ... | ... | ... |
| SE2 | .000 | ... | ... | ... |
| SE1 | .000 | ... | ... | ... |
| DM5 | .000 | .353 | ... | ... |
| DM3 | .000 | .356 | ... | ... |
| DM2 | .000 | .353 | ... | ... |
| DM1 | .000 | .349 | ... | ... |
| AA5 | ... | ... | ... | ... |
| AA4 | ... | ... | ... | ... |
| AA2 | ... | ... | ... | ... |
| AA1 | ... | ... | ... | ... |

##### Standardized Indirect Effects (Group number 1 - Default model)

##### Standardized Indirect Effects - Lower Bounds (BC) (Group number 1 - Default model)

|  | AA | SE | DM | AB |
| --- | --- | --- | --- | --- |
| SE | .000 | .000 | .000 | .000 |
| DM | -.034 | .000 | .000 | .000 |
| AB | .309 | -.029 | .000 | .000 |
| AA3 | .000 | .000 | .000 | .000 |
| AB5 | .166 | .215 | .213 | .000 |
| AB3 | .189 | .243 | .233 | .000 |
| AB2 | .173 | .219 | .211 | .000 |
| AB1 | .178 | .226 | .219 | .000 |
| SE5 | .303 | .000 | .000 | .000 |
| SE4 | .312 | .000 | .000 | .000 |
| SE3 | .267 | .000 | .000 | .000 |
| SE2 | .275 | .000 | .000 | .000 |
| SE1 | .243 | .000 | .000 | .000 |
| DM5 | .269 | -.050 | .000 | .000 |
| DM3 | .289 | -.053 | .000 | .000 |
| DM2 | .282 | -.051 | .000 | .000 |
| DM1 | .268 | -.049 | .000 | .000 |
| AA5 | .000 | .000 | .000 | .000 |
| AA4 | .000 | .000 | .000 | .000 |
| AA2 | .000 | .000 | .000 | .000 |
| AA1 | .000 | .000 | .000 | .000 |

##### Standardized Indirect Effects - Upper Bounds (BC) (Group number 1 - Default model)

|  | AA | SE | DM | AB |
| --- | --- | --- | --- | --- |
| SE | .000 | .000 | .000 | .000 |
| DM | .097 | .000 | .000 | .000 |
| AB | .507 | .078 | .000 | .000 |
| AA3 | .000 | .000 | .000 | .000 |
| AB5 | .329 | .419 | .376 | .000 |
| AB3 | .366 | .452 | .425 | .000 |
| AB2 | .331 | .417 | .393 | .000 |
| AB1 | .346 | .430 | .400 | .000 |
| SE5 | .462 | .000 | .000 | .000 |
| SE4 | .470 | .000 | .000 | .000 |
| SE3 | .412 | .000 | .000 | .000 |
| SE2 | .423 | .000 | .000 | .000 |
| SE1 | .384 | .000 | .000 | .000 |
| DM5 | .418 | .134 | .000 | .000 |
| DM3 | .450 | .146 | .000 | .000 |
| DM2 | .441 | .144 | .000 | .000 |
| DM1 | .422 | .136 | .000 | .000 |
| AA5 | .000 | .000 | .000 | .000 |
| AA4 | .000 | .000 | .000 | .000 |
| AA2 | .000 | .000 | .000 | .000 |
| AA1 | .000 | .000 | .000 | .000 |

##### Standardized Indirect Effects - Two Tailed Significance (BC) (Group number 1 - Default model)

|  | AA | SE | DM | AB |
| --- | --- | --- | --- | --- |
| SE | ... | ... | ... | ... |
| DM | .341 | ... | ... | ... |
| AB | .000 | .327 | ... | ... |
| AA3 | ... | ... | ... | ... |
| AB5 | .000 | .000 | .000 | ... |
| AB3 | .000 | .000 | .000 | ... |
| AB2 | .000 | .000 | .000 | ... |
| AB1 | .000 | .000 | .000 | ... |
| SE5 | .000 | ... | ... | ... |
| SE4 | .000 | ... | ... | ... |
| SE3 | .000 | ... | ... | ... |
| SE2 | .000 | ... | ... | ... |
| SE1 | .000 | ... | ... | ... |
| DM5 | .000 | .356 | ... | ... |
| DM3 | .000 | .353 | ... | ... |
| DM2 | .000 | .350 | ... | ... |
| DM1 | .000 | .350 | ... | ... |
| AA5 | ... | ... | ... | ... |
| AA4 | ... | ... | ... | ... |
| AA2 | ... | ... | ... | ... |
| AA1 | ... | ... | ... | ... |

##### Minimization History (Default model)

| Iteration |  | Negative eigenvalues | Condition # | Smallest eigenvalue | Diameter | F | NTries | Ratio |
| --- | --- | --- | --- | --- | --- | --- | --- | --- |
| 0 | e | 8 |  | -.634 | 9999.000 | 3177.379 | 0 | 9999.000 |
| 1 | e\* | 3 |  | -.089 | 3.627 | 1111.234 | 20 | .329 |
| 2 | e | 1 |  | -.032 | 1.414 | 382.157 | 4 | .779 |
| 3 | e | 0 | 16.121 |  | .986 | 181.892 | 5 | .804 |
| 4 | e | 0 | 21.039 |  | .481 | 138.490 | 1 | 1.108 |
| 5 | e | 0 | 36.457 |  | .224 | 134.146 | 1 | 1.103 |
| 6 | e | 0 | 46.004 |  | .062 | 133.952 | 1 | 1.048 |
| 7 | e | 0 | 47.415 |  | .006 | 133.951 | 1 | 1.006 |
| 8 | e | 0 | 47.418 |  | .000 | 133.951 | 1 | 1.000 |

##### Bootstrap (Default model)

##### Summary of Bootstrap Iterations (Default model)

##### (Default model)

| Iterations | Method 0 | Method 1 | Method 2 |
| --- | --- | --- | --- |
| 1 | 0 | 0 | 0 |
| 2 | 0 | 0 | 0 |
| 3 | 0 | 0 | 0 |
| 4 | 0 | 5 | 0 |
| 5 | 0 | 568 | 0 |
| 6 | 0 | 2014 | 0 |
| 7 | 0 | 1481 | 0 |
| 8 | 0 | 665 | 0 |
| 9 | 0 | 203 | 0 |
| 10 | 0 | 47 | 0 |
| 11 | 0 | 16 | 0 |
| 12 | 0 | 1 | 0 |
| 13 | 0 | 0 | 0 |
| 14 | 0 | 0 | 0 |
| 15 | 0 | 0 | 0 |
| 16 | 0 | 0 | 0 |
| 17 | 0 | 0 | 0 |
| 18 | 0 | 0 | 0 |
| 19 | 0 | 0 | 0 |
| Total | 0 | 5000 | 0 |

0 bootstrap samples were unused because of a singular covariance matrix.

0 bootstrap samples were unused because a solution was not found.

5000 usable bootstrap samples were obtained.

##### Bootstrap Distributions (Default model)

##### ML discrepancy (implied vs sample) (Default model)

|  |  |  |
| --- | --- | --- |
|  |  | \|-------------------- |
|  | 181.309 | \|\* |
|  | 196.210 | \|\* |
|  | 211.110 | \|\*\*\* |
|  | 226.010 | \|\*\*\*\*\*\*\*\* |
|  | 240.911 | \|\*\*\*\*\*\*\*\*\*\*\*\*\*\*\* |
|  | 255.811 | \|\*\*\*\*\*\*\*\*\*\*\*\*\*\*\*\*\*\*\* |
|  | 270.711 | \|\*\*\*\*\*\*\*\*\*\*\*\*\*\*\*\*\*\*\*\* |
| N = 5000 | 285.612 | \|\*\*\*\*\*\*\*\*\*\*\*\*\*\*\*\*\* |
| Mean = 269.972 | 300.512 | \|\*\*\*\*\*\*\*\*\*\*\* |
| S. e. = .429 | 315.412 | \|\*\*\*\*\*\*\* |
|  | 330.313 | \|\*\*\*\* |
|  | 345.213 | \|\* |
|  | 360.113 | \|\* |
|  | 375.014 | \|\* |
|  | 389.914 | \|\* |
|  |  | \|-------------------- |

##### ML discrepancy (implied vs pop) (Default model)

|  |  |  |
| --- | --- | --- |
|  |  | \|-------------------- |
|  | 156.858 | \|\* |
|  | 164.200 | \|\*\* |
|  | 171.542 | \|\*\*\*\*\*\*\*\* |
|  | 178.885 | \|\*\*\*\*\*\*\*\*\*\*\*\*\*\*\*\*\* |
|  | 186.227 | \|\*\*\*\*\*\*\*\*\*\*\*\*\*\*\*\*\*\*\*\* |
|  | 193.569 | \|\*\*\*\*\*\*\*\*\*\*\*\*\*\*\*\* |
|  | 200.911 | \|\*\*\*\*\*\*\*\*\* |
| N = 5000 | 208.253 | \|\*\*\*\*\* |
| Mean = 188.178 | 215.595 | \|\*\* |
| S. e. = .163 | 222.937 | \|\* |
|  | 230.280 | \|\* |
|  | 237.622 | \|\* |
|  | 244.964 | \|\* |
|  | 252.306 | \| |
|  | 259.648 | \|\* |
|  |  | \|-------------------- |

##### K-L overoptimism (unstabilized) (Default model)

|  |  |  |
| --- | --- | --- |
|  |  | \|-------------------- |
|  | -273.765 | \|\* |
|  | -211.586 | \|\* |
|  | -149.407 | \|\*\* |
|  | -87.228 | \|\*\*\*\*\* |
|  | -25.048 | \|\*\*\*\*\*\*\*\*\*\*\*\* |
|  | 37.131 | \|\*\*\*\*\*\*\*\*\*\*\*\*\*\*\*\*\*\* |
|  | 99.310 | \|\*\*\*\*\*\*\*\*\*\*\*\*\*\*\*\*\*\*\*\* |
| N = 5000 | 161.489 | \|\*\*\*\*\*\*\*\*\*\*\*\*\*\*\*\*\* |
| Mean = 105.315 | 223.668 | \|\*\*\*\*\*\*\*\*\*\*\* |
| S. e. = 1.631 | 285.848 | \|\*\*\*\*\*\* |
|  | 348.027 | \|\*\*\* |
|  | 410.206 | \|\* |
|  | 472.385 | \|\* |
|  | 534.564 | \|\* |
|  | 596.744 | \|\* |
|  |  | \|-------------------- |

##### K-L overoptimism (stabilized) (Default model)

|  |  |  |
| --- | --- | --- |
|  |  | \|-------------------- |
|  | 2.933 | \|\* |
|  | 19.375 | \|\* |
|  | 35.817 | \|\* |
|  | 52.259 | \|\*\*\*\* |
|  | 68.701 | \|\*\*\*\*\*\*\*\*\* |
|  | 85.143 | \|\*\*\*\*\*\*\*\*\*\*\*\*\*\*\*\* |
|  | 101.585 | \|\*\*\*\*\*\*\*\*\*\*\*\*\*\*\*\*\*\*\*\* |
| N = 5000 | 118.028 | \|\*\*\*\*\*\*\*\*\*\*\*\*\*\*\*\*\*\*\*\* |
| Mean = 107.766 | 134.470 | \|\*\*\*\*\*\*\*\*\*\*\*\*\*\* |
| S. e. = .407 | 150.912 | \|\*\*\*\*\*\* |
|  | 167.354 | \|\*\*\* |
|  | 183.796 | \|\* |
|  | 200.238 | \|\* |
|  | 216.680 | \|\* |
|  | 233.122 | \|\* |
|  |  | \|-------------------- |

##### Model Fit Summary

##### CMIN

| Model | NPAR | CMIN | DF | P | CMIN/DF |
| --- | --- | --- | --- | --- | --- |
| Default model | 42 | 133.951 | 129 | .365 | 1.038 |
| Saturated model | 171 | .000 | 0 |  |  |
| Independence model | 18 | 3236.025 | 153 | .000 | 21.150 |

##### RMR, GFI

| Model | RMR | GFI | AGFI | PGFI |
| --- | --- | --- | --- | --- |
| Default model | .102 | .963 | .952 | .727 |
| Saturated model | .000 | 1.000 |  |  |
| Independence model | 1.055 | .337 | .259 | .302 |

##### Baseline Comparisons

| Model | NFI Delta1 | RFI rho1 | IFI Delta2 | TLI rho2 | CFI |
| --- | --- | --- | --- | --- | --- |
| Default model | .959 | .951 | .998 | .998 | .998 |
| Saturated model | 1.000 |  | 1.000 |  | 1.000 |
| Independence model | .000 | .000 | .000 | .000 | .000 |

##### Parsimony-Adjusted Measures

| Model | PRATIO | PNFI | PCFI |
| --- | --- | --- | --- |
| Default model | .843 | .808 | .842 |
| Saturated model | .000 | .000 | .000 |
| Independence model | 1.000 | .000 | .000 |

##### NCP

| Model | NCP | LO 90 | HI 90 |
| --- | --- | --- | --- |
| Default model | 4.951 | .000 | 36.639 |
| Saturated model | .000 | .000 | .000 |
| Independence model | 3083.025 | 2901.704 | 3271.665 |

##### FMIN

| Model | FMIN | F0 | LO 90 | HI 90 |
| --- | --- | --- | --- | --- |
| Default model | .347 | .013 | .000 | .095 |
| Saturated model | .000 | .000 | .000 | .000 |
| Independence model | 8.383 | 7.987 | 7.517 | 8.476 |

##### RMSEA

| Model | RMSEA | LO 90 | HI 90 | PCLOSE |
| --- | --- | --- | --- | --- |
| Default model | .010 | .000 | .027 | 1.000 |
| Independence model | .228 | .222 | .235 | .000 |

##### AIC

| Model | AIC | BCC | BIC | CAIC |
| --- | --- | --- | --- | --- |
| Default model | 217.951 | 222.300 | 384.205 | 426.205 |
| Saturated model | 342.000 | 359.706 | 1018.891 | 1189.891 |
| Independence model | 3272.025 | 3273.889 | 3343.277 | 3361.277 |

##### ECVI

| Model | ECVI | LO 90 | HI 90 | MECVI |
| --- | --- | --- | --- | --- |
| Default model | .565 | .552 | .647 | .576 |
| Saturated model | .886 | .886 | .886 | .932 |
| Independence model | 8.477 | 8.007 | 8.965 | 8.482 |

##### HOELTER

| Model | HOELTER .05 | HOELTER .01 |
| --- | --- | --- |
| Default model | 451 | 488 |
| Independence model | 22 | 24 |

##### Execution time summary

|  |  |
| --- | --- |
| Minimization: | .020 |
| Miscellaneous: | .300 |
| Bootstrap: | 3.179 |
| Total: | 3.499 |
