# Supplementary material for: How AI threat perception relates to post-adoptive AI agent engagement: the dual paths of self-efficacy and defensive motivation
Source: Front Psychol. 2026 Oct 22;17:1887571. doi: 10.3389/fpsyg.2026.1887571 (PMC13735560; doi:10.3389/fpsyg.2026.1887571)
Supplement: Supplementary file 1 [file Data_Sheet_1.zip › log+Questionnaire/004_MGA_Moderation_Age.AmosOutput]

多群组比较模型-年龄.amw


#### D:\学术研究\066研究数据与论文撰写\01研究数据\AI智能体论文\多群组比较\多群组比较模型-年龄.amw

##### Analysis Summary

##### Date and Time

Date: 2026年9月13日

Time: 17:45:21

##### Title

多群组比较模型-年龄: 2026年9月13日 17:45

##### Groups

##### Group number 1 (Group number 1)

##### Notes for Group (Group number 1)

The model is recursive.

Sample size = 282

##### Variable Summary (Middle)

##### Your model contains the following variables (Middle)

Observed, endogenous variables

AA1

AA2

AA3

AA4

AA5

DM1

DM2

DM3

DM5

SE1

SE2

SE3

SE4

SE5

AB1

AB2

AB3

AB5

Unobserved, endogenous variables

防御性动机

AI自我效能

使用行为

Unobserved, exogenous variables

AI威胁感知

e1

e2

e3

e4

e5

e6

e8

e9

e10

e11

e12

e13

e14

e15

e16

e17

e18

e20

e21

e22

e23

##### Variable counts (Middle)

|  |  |
| --- | --- |
| Number of variables in your model: | 43 |
| Number of observed variables: | 18 |
| Number of unobserved variables: | 25 |
| Number of exogenous variables: | 22 |
| Number of endogenous variables: | 21 |

##### Parameter Summary (Middle)

|  | Weights | Covariances | Variances | Means | Intercepts | Total |
| --- | --- | --- | --- | --- | --- | --- |
| Fixed | 25 | 0 | 0 | 0 | 0 | 25 |
| Labeled | 5 | 0 | 0 | 0 | 0 | 5 |
| Unlabeled | 14 | 0 | 22 | 0 | 0 | 36 |
| Total | 44 | 0 | 22 | 0 | 0 | 66 |

##### Assessment of normality (Middle)

| Variable | min | max | skew | c.r. | kurtosis | c.r. |
| --- | --- | --- | --- | --- | --- | --- |
| AB5 | 1.000 | 7.000 | .091 | .625 | -1.083 | -3.713 |
| AB3 | 1.000 | 7.000 | .037 | .252 | -1.084 | -3.714 |
| AB2 | 1.000 | 7.000 | .016 | .109 | -1.137 | -3.899 |
| AB1 | 1.000 | 7.000 | .102 | .699 | -1.153 | -3.954 |
| SE5 | 1.000 | 7.000 | .062 | .428 | -1.022 | -3.503 |
| SE4 | 1.000 | 7.000 | .097 | .668 | -1.095 | -3.754 |
| SE3 | 1.000 | 7.000 | .016 | .109 | -1.122 | -3.845 |
| SE2 | 1.000 | 7.000 | .100 | .684 | -1.088 | -3.728 |
| SE1 | 1.000 | 7.000 | .084 | .578 | -1.093 | -3.745 |
| DM5 | 1.000 | 7.000 | .059 | .404 | -1.131 | -3.877 |
| DM3 | 1.000 | 7.000 | .102 | .697 | -1.056 | -3.619 |
| DM2 | 1.000 | 7.000 | .081 | .556 | -1.106 | -3.792 |
| DM1 | 1.000 | 7.000 | .043 | .294 | -1.121 | -3.843 |
| AA5 | 1.000 | 7.000 | .031 | .210 | -1.118 | -3.833 |
| AA4 | 1.000 | 7.000 | .033 | .227 | -1.121 | -3.844 |
| AA3 | 1.000 | 7.000 | .202 | 1.382 | -1.097 | -3.760 |
| AA2 | 1.000 | 7.000 | .012 | .080 | -1.101 | -3.775 |
| AA1 | 1.000 | 7.000 | .112 | .770 | -1.114 | -3.820 |
| Multivariate |  |  |  |  | -1.385 | -.433 |

##### Observations farthest from the centroid (Mahalanobis distance) (Middle)

| Observation number | Mahalanobis d-squared | p1 | p2 |
| --- | --- | --- | --- |
| 11 | 34.470 | .011 | .956 |
| 241 | 31.256 | .027 | .996 |
| 185 | 31.108 | .028 | .986 |
| 251 | 30.337 | .034 | .988 |
| 142 | 30.218 | .035 | .972 |
| 21 | 30.182 | .036 | .939 |
| 112 | 30.010 | .037 | .904 |
| 219 | 29.959 | .038 | .840 |
| 152 | 29.746 | .040 | .797 |
| 6 | 29.558 | .042 | .749 |
| 230 | 29.224 | .046 | .745 |
| 128 | 29.208 | .046 | .646 |
| 97 | 28.629 | .053 | .737 |
| 206 | 28.542 | .054 | .671 |
| 260 | 28.258 | .058 | .677 |
| 104 | 28.071 | .061 | .652 |
| 226 | 27.987 | .062 | .589 |
| 46 | 27.732 | .066 | .599 |
| 2 | 27.692 | .067 | .521 |
| 29 | 27.481 | .070 | .520 |
| 37 | 27.390 | .072 | .468 |
| 228 | 27.239 | .075 | .446 |
| 51 | 27.208 | .075 | .373 |
| 3 | 27.187 | .076 | .302 |
| 168 | 27.121 | .077 | .255 |
| 188 | 26.289 | .093 | .556 |
| 255 | 26.276 | .094 | .481 |
| 69 | 25.942 | .101 | .569 |
| 160 | 25.799 | .104 | .564 |
| 141 | 25.593 | .109 | .593 |
| 159 | 25.477 | .112 | .579 |
| 154 | 25.308 | .117 | .594 |
| 56 | 25.278 | .117 | .536 |
| 171 | 25.266 | .118 | .469 |
| 88 | 25.036 | .124 | .523 |
| 199 | 24.934 | .127 | .509 |
| 94 | 24.930 | .127 | .440 |
| 111 | 24.825 | .130 | .429 |
| 274 | 24.599 | .136 | .489 |
| 149 | 24.564 | .137 | .440 |
| 223 | 24.512 | .139 | .403 |
| 101 | 24.501 | .139 | .345 |
| 238 | 24.328 | .145 | .378 |
| 178 | 24.137 | .151 | .424 |
| 176 | 24.087 | .152 | .390 |
| 72 | 23.977 | .156 | .392 |
| 183 | 23.971 | .156 | .334 |
| 242 | 23.770 | .163 | .391 |
| 269 | 23.756 | .163 | .339 |
| 200 | 23.684 | .166 | .323 |
| 120 | 23.640 | .167 | .292 |
| 164 | 23.513 | .172 | .308 |
| 218 | 23.482 | .173 | .272 |
| 153 | 23.114 | .186 | .434 |
| 35 | 23.111 | .186 | .378 |
| 161 | 22.960 | .192 | .415 |
| 217 | 22.864 | .196 | .419 |
| 25 | 22.819 | .198 | .390 |
| 193 | 22.587 | .207 | .486 |
| 233 | 22.500 | .211 | .487 |
| 99 | 22.488 | .211 | .437 |
| 49 | 22.484 | .211 | .384 |
| 208 | 22.215 | .223 | .510 |
| 81 | 22.145 | .226 | .502 |
| 63 | 22.132 | .226 | .454 |
| 55 | 22.074 | .229 | .439 |
| 78 | 21.968 | .233 | .457 |
| 34 | 21.923 | .235 | .433 |
| 61 | 21.772 | .242 | .485 |
| 62 | 21.719 | .245 | .468 |
| 39 | 21.637 | .248 | .472 |
| 52 | 21.608 | .250 | .439 |
| 232 | 21.532 | .253 | .439 |
| 167 | 21.486 | .256 | .419 |
| 276 | 21.424 | .259 | .410 |
| 162 | 21.297 | .265 | .450 |
| 85 | 21.252 | .267 | .430 |
| 7 | 21.113 | .274 | .480 |
| 31 | 21.085 | .275 | .448 |
| 15 | 20.992 | .280 | .465 |
| 125 | 20.989 | .280 | .415 |
| 163 | 20.905 | .284 | .426 |
| 64 | 20.901 | .284 | .378 |
| 20 | 20.876 | .286 | .347 |
| 54 | 20.782 | .291 | .366 |
| 196 | 20.678 | .296 | .392 |
| 282 | 20.614 | .299 | .390 |
| 250 | 20.547 | .303 | .390 |
| 267 | 20.546 | .303 | .342 |
| 50 | 20.544 | .303 | .298 |
| 76 | 20.544 | .303 | .255 |
| 47 | 20.442 | .309 | .279 |
| 60 | 20.406 | .310 | .260 |
| 132 | 20.308 | .316 | .282 |
| 237 | 20.031 | .331 | .440 |
| 268 | 19.906 | .338 | .490 |
| 216 | 19.801 | .344 | .525 |
| 148 | 19.761 | .346 | .507 |
| 131 | 19.748 | .347 | .468 |
| 91 | 19.727 | .348 | .435 |

##### Group number 2 (Group number 2)

##### Notes for Group (Group number 2)

The model is recursive.

Sample size = 105

##### Variable Summary (others)

##### Your model contains the following variables (others)

Observed, endogenous variables

AA1

AA2

AA3

AA4

AA5

DM1

DM2

DM3

DM5

SE1

SE2

SE3

SE4

SE5

AB1

AB2

AB3

AB5

Unobserved, endogenous variables

防御性动机

AI自我效能

使用行为

Unobserved, exogenous variables

AI威胁感知

e1

e2

e3

e4

e5

e6

e8

e9

e10

e11

e12

e13

e14

e15

e16

e17

e18

e20

e21

e22

e23

##### Variable counts (others)

|  |  |
| --- | --- |
| Number of variables in your model: | 43 |
| Number of observed variables: | 18 |
| Number of unobserved variables: | 25 |
| Number of exogenous variables: | 22 |
| Number of endogenous variables: | 21 |

##### Parameter Summary (others)

|  | Weights | Covariances | Variances | Means | Intercepts | Total |
| --- | --- | --- | --- | --- | --- | --- |
| Fixed | 25 | 0 | 0 | 0 | 0 | 25 |
| Labeled | 5 | 0 | 0 | 0 | 0 | 5 |
| Unlabeled | 14 | 0 | 22 | 0 | 0 | 36 |
| Total | 44 | 0 | 22 | 0 | 0 | 66 |

##### Assessment of normality (others)

| Variable | min | max | skew | c.r. | kurtosis | c.r. |
| --- | --- | --- | --- | --- | --- | --- |
| AB5 | 1.000 | 7.000 | -.116 | -.486 | -1.191 | -2.490 |
| AB3 | 1.000 | 7.000 | .085 | .355 | -1.010 | -2.112 |
| AB2 | 1.000 | 7.000 | .033 | .139 | -1.100 | -2.302 |
| AB1 | 1.000 | 7.000 | .020 | .085 | -.965 | -2.018 |
| SE5 | 1.000 | 7.000 | -.024 | -.100 | -1.155 | -2.416 |
| SE4 | 1.000 | 7.000 | -.065 | -.271 | -1.235 | -2.583 |
| SE3 | 1.000 | 7.000 | .148 | .621 | -1.036 | -2.167 |
| SE2 | 1.000 | 7.000 | -.054 | -.227 | -1.027 | -2.148 |
| SE1 | 1.000 | 7.000 | .127 | .532 | -.927 | -1.939 |
| DM5 | 1.000 | 7.000 | .051 | .213 | -1.078 | -2.254 |
| DM3 | 1.000 | 7.000 | .123 | .516 | -1.017 | -2.127 |
| DM2 | 1.000 | 7.000 | .151 | .632 | -.948 | -1.983 |
| DM1 | 1.000 | 7.000 | .096 | .401 | -1.119 | -2.341 |
| AA5 | 1.000 | 7.000 | .133 | .557 | -1.007 | -2.106 |
| AA4 | 1.000 | 7.000 | .048 | .201 | -1.139 | -2.383 |
| AA3 | 1.000 | 7.000 | -.172 | -.718 | -.932 | -1.949 |
| AA2 | 1.000 | 7.000 | .306 | 1.279 | -1.004 | -2.099 |
| AA1 | 1.000 | 7.000 | .026 | .109 | -1.067 | -2.232 |
| Multivariate |  |  |  |  | -9.559 | -1.825 |

##### Observations farthest from the centroid (Mahalanobis distance) (others)

| Observation number | Mahalanobis d-squared | p1 | p2 |
| --- | --- | --- | --- |
| 40 | 29.705 | .040 | .987 |
| 79 | 28.534 | .054 | .980 |
| 22 | 28.352 | .057 | .942 |
| 66 | 27.847 | .064 | .912 |
| 57 | 27.071 | .078 | .918 |
| 53 | 25.719 | .106 | .973 |
| 51 | 24.714 | .133 | .990 |
| 88 | 24.668 | .134 | .978 |
| 96 | 24.460 | .141 | .968 |
| 2 | 24.091 | .152 | .967 |
| 60 | 23.936 | .157 | .953 |
| 65 | 23.779 | .162 | .935 |
| 35 | 23.734 | .164 | .897 |
| 78 | 23.627 | .168 | .859 |
| 38 | 23.406 | .175 | .843 |
| 4 | 23.153 | .185 | .837 |
| 47 | 22.915 | .194 | .829 |
| 14 | 22.869 | .196 | .770 |
| 25 | 22.204 | .223 | .877 |
| 20 | 22.131 | .226 | .839 |
| 98 | 22.101 | .228 | .783 |
| 102 | 21.721 | .245 | .829 |
| 9 | 21.549 | .253 | .816 |
| 48 | 21.331 | .263 | .819 |
| 73 | 21.056 | .277 | .839 |
| 54 | 20.992 | .280 | .799 |
| 28 | 20.936 | .283 | .752 |
| 100 | 20.308 | .316 | .884 |
| 11 | 20.283 | .317 | .843 |
| 21 | 20.264 | .318 | .792 |
| 18 | 20.139 | .325 | .774 |
| 84 | 19.965 | .335 | .773 |
| 16 | 19.838 | .342 | .757 |
| 92 | 19.604 | .356 | .781 |
| 34 | 19.530 | .360 | .746 |
| 19 | 19.475 | .363 | .701 |
| 89 | 19.447 | .365 | .640 |
| 70 | 19.338 | .371 | .615 |
| 90 | 19.219 | .378 | .595 |
| 56 | 19.048 | .389 | .602 |
| 80 | 18.925 | .396 | .586 |
| 41 | 18.707 | .410 | .619 |
| 50 | 18.634 | .415 | .580 |
| 82 | 18.632 | .415 | .502 |
| 105 | 18.316 | .435 | .590 |
| 61 | 18.231 | .441 | .558 |
| 46 | 18.188 | .443 | .502 |
| 23 | 18.087 | .450 | .479 |
| 6 | 17.926 | .461 | .488 |
| 26 | 17.846 | .466 | .454 |
| 7 | 17.837 | .466 | .382 |
| 10 | 17.739 | .473 | .359 |
| 85 | 17.719 | .474 | .299 |
| 17 | 17.718 | .474 | .235 |
| 64 | 17.608 | .482 | .222 |
| 93 | 17.505 | .489 | .207 |
| 69 | 17.351 | .499 | .212 |
| 42 | 17.166 | .512 | .231 |
| 24 | 17.137 | .514 | .187 |
| 101 | 17.130 | .514 | .141 |
| 12 | 17.086 | .517 | .113 |
| 15 | 17.062 | .519 | .085 |
| 104 | 16.943 | .527 | .080 |
| 103 | 16.615 | .550 | .128 |
| 39 | 16.440 | .562 | .139 |
| 37 | 16.393 | .565 | .112 |
| 43 | 16.368 | .567 | .084 |
| 33 | 15.861 | .602 | .198 |
| 58 | 15.819 | .605 | .161 |
| 81 | 15.762 | .609 | .134 |
| 13 | 15.598 | .621 | .141 |
| 44 | 15.357 | .637 | .177 |
| 30 | 15.058 | .658 | .243 |
| 94 | 15.018 | .661 | .198 |
| 3 | 14.963 | .665 | .164 |
| 55 | 14.928 | .667 | .128 |
| 95 | 14.737 | .680 | .142 |
| 27 | 14.729 | .680 | .101 |
| 52 | 14.565 | .692 | .105 |
| 76 | 14.405 | .702 | .108 |
| 49 | 14.281 | .711 | .101 |
| 63 | 13.970 | .731 | .148 |
| 99 | 13.834 | .740 | .141 |
| 1 | 13.791 | .743 | .107 |
| 29 | 13.759 | .745 | .076 |
| 67 | 13.578 | .756 | .079 |
| 32 | 13.450 | .764 | .072 |
| 68 | 13.396 | .768 | .051 |
| 62 | 13.393 | .768 | .030 |
| 72 | 13.358 | .770 | .018 |
| 8 | 13.223 | .778 | .016 |
| 75 | 13.127 | .784 | .011 |
| 71 | 13.100 | .786 | .006 |
| 45 | 13.086 | .786 | .003 |
| 74 | 13.010 | .791 | .002 |
| 86 | 12.668 | .811 | .003 |
| 59 | 12.649 | .812 | .001 |
| 83 | 12.219 | .836 | .003 |
| 36 | 11.382 | .877 | .021 |
| 87 | 11.339 | .879 | .010 |

##### Models

##### Default model (Default model)

##### Notes for Model (Default model)

##### Computation of degrees of freedom (Default model)

|  |  |
| --- | --- |
| Number of distinct sample moments: | 342 |
| Number of distinct parameters to be estimated: | 82 |
| Degrees of freedom (342 - 82): | 260 |

##### Result (Default model)

Minimum was achieved

Chi-square = 289.057

Degrees of freedom = 260

Probability level = .104

##### Middle (Middle - Default model)

##### Estimates (Middle - Default model)

##### Scalar Estimates (Middle - Default model)

##### Maximum Likelihood Estimates

##### Regression Weights: (Middle - Default model)

|  |  |  | Estimate | S.E. | C.R. | P | Label |
| --- | --- | --- | --- | --- | --- | --- | --- |
| AI自我效能 | <--- | AI威胁感知 | .481 | .061 | 7.821 | \*\*\* | H1a |
| 防御性动机 | <--- | AI威胁感知 | .378 | .074 | 5.085 | \*\*\* | H2a |
| 防御性动机 | <--- | AI自我效能 | .141 | .085 | 1.660 | .097 | H5a |
| 使用行为 | <--- | 防御性动机 | .384 | .081 | 4.767 | \*\*\* | H4a |
| 使用行为 | <--- | AI自我效能 | .515 | .086 | 6.017 | \*\*\* | H3a |
| AA1 | <--- | AI威胁感知 | 1.000 |  |  |  |  |
| AA2 | <--- | AI威胁感知 | .950 | .065 | 14.701 | \*\*\* |  |
| AA3 | <--- | AI威胁感知 | .926 | .068 | 13.705 | \*\*\* |  |
| AA4 | <--- | AI威胁感知 | .936 | .066 | 14.149 | \*\*\* |  |
| AA5 | <--- | AI威胁感知 | .926 | .064 | 14.543 | \*\*\* |  |
| DM1 | <--- | 防御性动机 | 1.000 |  |  |  |  |
| DM2 | <--- | 防御性动机 | 1.068 | .102 | 10.462 | \*\*\* |  |
| DM3 | <--- | 防御性动机 | 1.082 | .102 | 10.604 | \*\*\* |  |
| DM5 | <--- | 防御性动机 | 1.013 | .101 | 9.985 | \*\*\* |  |
| SE1 | <--- | AI自我效能 | 1.000 |  |  |  |  |
| SE2 | <--- | AI自我效能 | 1.093 | .105 | 10.434 | \*\*\* |  |
| SE3 | <--- | AI自我效能 | .992 | .100 | 9.906 | \*\*\* |  |
| SE4 | <--- | AI自我效能 | 1.152 | .102 | 11.310 | \*\*\* |  |
| SE5 | <--- | AI自我效能 | 1.152 | .101 | 11.438 | \*\*\* |  |
| AB1 | <--- | 使用行为 | 1.000 |  |  |  |  |
| AB2 | <--- | 使用行为 | .923 | .078 | 11.788 | \*\*\* |  |
| AB3 | <--- | 使用行为 | 1.036 | .078 | 13.300 | \*\*\* |  |
| AB5 | <--- | 使用行为 | .962 | .079 | 12.192 | \*\*\* |  |

##### Standardized Regression Weights: (Middle - Default model)

|  |  |  | Estimate |
| --- | --- | --- | --- |
| AI自我效能 | <--- | AI威胁感知 | .580 |
| 防御性动机 | <--- | AI威胁感知 | .445 |
| 防御性动机 | <--- | AI自我效能 | .138 |
| 使用行为 | <--- | 防御性动机 | .341 |
| 使用行为 | <--- | AI自我效能 | .446 |
| AA1 | <--- | AI威胁感知 | .824 |
| AA2 | <--- | AI威胁感知 | .789 |
| AA3 | <--- | AI威胁感知 | .748 |
| AA4 | <--- | AI威胁感知 | .766 |
| AA5 | <--- | AI威胁感知 | .783 |
| DM1 | <--- | 防御性动机 | .688 |
| DM2 | <--- | 防御性动机 | .747 |
| DM3 | <--- | 防御性动机 | .761 |
| DM5 | <--- | 防御性动机 | .703 |
| SE1 | <--- | AI自我效能 | .675 |
| SE2 | <--- | AI自我效能 | .715 |
| SE3 | <--- | AI自我效能 | .674 |
| SE4 | <--- | AI自我效能 | .790 |
| SE5 | <--- | AI自我效能 | .802 |
| AB1 | <--- | 使用行为 | .774 |
| AB2 | <--- | 使用行为 | .719 |
| AB3 | <--- | 使用行为 | .812 |
| AB5 | <--- | 使用行为 | .743 |

##### Variances: (Middle - Default model)

|  |  |  | Estimate | S.E. | C.R. | P | Label |
| --- | --- | --- | --- | --- | --- | --- | --- |
| AI威胁感知 |  |  | 2.208 | .272 | 8.134 | \*\*\* |  |
| e21 |  |  | 1.010 | .174 | 5.797 | \*\*\* |  |
| e23 |  |  | 1.135 | .196 | 5.793 | \*\*\* |  |
| e22 |  |  | 1.143 | .175 | 6.531 | \*\*\* |  |
| e1 |  |  | 1.045 | .118 | 8.886 | \*\*\* |  |
| e2 |  |  | 1.208 | .127 | 9.541 | \*\*\* |  |
| e3 |  |  | 1.491 | .148 | 10.078 | \*\*\* |  |
| e4 |  |  | 1.360 | .138 | 9.861 | \*\*\* |  |
| e5 |  |  | 1.197 | .124 | 9.639 | \*\*\* |  |
| e6 |  |  | 1.774 | .184 | 9.667 | \*\*\* |  |
| e8 |  |  | 1.444 | .165 | 8.769 | \*\*\* |  |
| e9 |  |  | 1.355 | .160 | 8.481 | \*\*\* |  |
| e10 |  |  | 1.676 | .177 | 9.476 | \*\*\* |  |
| e11 |  |  | 1.819 | .175 | 10.387 | \*\*\* |  |
| e12 |  |  | 1.733 | .173 | 10.009 | \*\*\* |  |
| e13 |  |  | 1.804 | .174 | 10.397 | \*\*\* |  |
| e14 |  |  | 1.217 | .136 | 8.921 | \*\*\* |  |
| e15 |  |  | 1.122 | .129 | 8.673 | \*\*\* |  |
| e16 |  |  | 1.357 | .151 | 8.958 | \*\*\* |  |
| e17 |  |  | 1.607 | .164 | 9.789 | \*\*\* |  |
| e18 |  |  | 1.120 | .138 | 8.092 | \*\*\* |  |
| e20 |  |  | 1.521 | .161 | 9.477 | \*\*\* |  |

##### Squared Multiple Correlations: (Middle - Default model)

|  |  |  | Estimate |
| --- | --- | --- | --- |
| AI自我效能 |  |  | .336 |
| 防御性动机 |  |  | .288 |
| 使用行为 |  |  | .435 |
| AB5 |  |  | .552 |
| AB3 |  |  | .660 |
| AB2 |  |  | .518 |
| AB1 |  |  | .599 |
| SE5 |  |  | .643 |
| SE4 |  |  | .624 |
| SE3 |  |  | .454 |
| SE2 |  |  | .512 |
| SE1 |  |  | .455 |
| DM5 |  |  | .494 |
| DM3 |  |  | .579 |
| DM2 |  |  | .557 |
| DM1 |  |  | .473 |
| AA5 |  |  | .612 |
| AA4 |  |  | .587 |
| AA3 |  |  | .559 |
| AA2 |  |  | .622 |
| AA1 |  |  | .679 |

##### User-defined estimands: (Middle - Default model)

|  |  |  |  |
| --- | --- | --- | --- |
| diff1 |  |  | .335 |
| diff2 |  |  | .232 |
| diff3 |  |  | .369 |
| diff4 |  |  | .238 |
| diff5 |  |  | -.005 |

##### Modification Indices (Middle - Default model)

##### Covariances: (Middle - Default model)

|  |  |  | M.I. | Par Change |
| --- | --- | --- | --- | --- |
| e8 | <--> | e22 | 12.054 | .352 |
| e8 | <--> | e17 | 4.233 | .228 |
| e5 | <--> | e17 | 5.158 | -.222 |
| e4 | <--> | e15 | 4.341 | -.189 |
| e3 | <--> | e17 | 5.616 | .254 |
| e3 | <--> | e4 | 4.325 | -.203 |
| e2 | <--> | e22 | 9.029 | -.273 |
| e1 | <--> | e15 | 4.136 | .169 |
| e1 | <--> | e12 | 8.114 | -.278 |

##### Variances: (Middle - Default model)

|  |  |  | M.I. | Par Change |
| --- | --- | --- | --- | --- |

##### Regression Weights: (Middle - Default model)

|  |  |  | M.I. | Par Change |
| --- | --- | --- | --- | --- |
| DM2 | <--- | 使用行为 | 7.680 | .169 |
| DM2 | <--- | AB3 | 6.540 | .114 |
| DM2 | <--- | AB2 | 10.081 | .140 |
| DM2 | <--- | AB1 | 7.037 | .116 |
| AA2 | <--- | AB2 | 6.185 | -.098 |
| AA1 | <--- | SE2 | 4.861 | -.081 |

##### Bootstrap (Middle - Default model)

##### Bootstrap standard errors (Middle - Default model)

##### Scalar Estimates (Middle - Default model)

##### Regression Weights: (Middle - Default model)

| Parameter | | | SE | SE-SE | Mean | Bias | SE-Bias |
| --- | --- | --- | --- | --- | --- | --- | --- |
| AI自我效能 | <--- | AI威胁感知 | .058 | .001 | .482 | .001 | .002 |
| 防御性动机 | <--- | AI威胁感知 | .076 | .002 | .381 | .003 | .002 |
| 防御性动机 | <--- | AI自我效能 | .088 | .002 | .141 | .000 | .003 |
| 使用行为 | <--- | 防御性动机 | .080 | .002 | .384 | .000 | .003 |
| 使用行为 | <--- | AI自我效能 | .085 | .002 | .513 | -.002 | .003 |
| AA1 | <--- | AI威胁感知 | .000 | .000 | 1.000 | .000 | .000 |
| AA2 | <--- | AI威胁感知 | .052 | .001 | .953 | .003 | .002 |
| AA3 | <--- | AI威胁感知 | .055 | .001 | .925 | -.001 | .002 |
| AA4 | <--- | AI威胁感知 | .058 | .001 | .934 | -.002 | .002 |
| AA5 | <--- | AI威胁感知 | .058 | .001 | .928 | .002 | .002 |
| DM1 | <--- | 防御性动机 | .000 | .000 | 1.000 | .000 | .000 |
| DM2 | <--- | 防御性动机 | .100 | .002 | 1.072 | .004 | .003 |
| DM3 | <--- | 防御性动机 | .096 | .002 | 1.087 | .005 | .003 |
| DM5 | <--- | 防御性动机 | .103 | .002 | 1.018 | .005 | .003 |
| SE1 | <--- | AI自我效能 | .000 | .000 | 1.000 | .000 | .000 |
| SE2 | <--- | AI自我效能 | .094 | .002 | 1.092 | -.001 | .003 |
| SE3 | <--- | AI自我效能 | .088 | .002 | .995 | .002 | .003 |
| SE4 | <--- | AI自我效能 | .096 | .002 | 1.154 | .003 | .003 |
| SE5 | <--- | AI自我效能 | .096 | .002 | 1.151 | -.001 | .003 |
| AB1 | <--- | 使用行为 | .000 | .000 | 1.000 | .000 | .000 |
| AB2 | <--- | 使用行为 | .066 | .001 | .925 | .003 | .002 |
| AB3 | <--- | 使用行为 | .069 | .002 | 1.042 | .006 | .002 |
| AB5 | <--- | 使用行为 | .072 | .002 | .963 | .001 | .002 |

##### Standardized Regression Weights: (Middle - Default model)

| Parameter | | | SE | SE-SE | Mean | Bias | SE-Bias |
| --- | --- | --- | --- | --- | --- | --- | --- |
| AI自我效能 | <--- | AI威胁感知 | .046 | .001 | .577 | -.003 | .001 |
| 防御性动机 | <--- | AI威胁感知 | .079 | .002 | .447 | .002 | .003 |
| 防御性动机 | <--- | AI自我效能 | .083 | .002 | .137 | .000 | .003 |
| 使用行为 | <--- | 防御性动机 | .065 | .001 | .340 | -.001 | .002 |
| 使用行为 | <--- | AI自我效能 | .063 | .001 | .445 | -.001 | .002 |
| AA1 | <--- | AI威胁感知 | .026 | .001 | .823 | -.001 | .001 |
| AA2 | <--- | AI威胁感知 | .024 | .001 | .790 | .001 | .001 |
| AA3 | <--- | AI威胁感知 | .029 | .001 | .747 | -.001 | .001 |
| AA4 | <--- | AI威胁感知 | .029 | .001 | .764 | -.002 | .001 |
| AA5 | <--- | AI威胁感知 | .032 | .001 | .784 | .002 | .001 |
| DM1 | <--- | 防御性动机 | .043 | .001 | .687 | -.001 | .001 |
| DM2 | <--- | 防御性动机 | .035 | .001 | .745 | -.002 | .001 |
| DM3 | <--- | 防御性动机 | .034 | .001 | .761 | -.001 | .001 |
| DM5 | <--- | 防御性动机 | .038 | .001 | .701 | -.001 | .001 |
| SE1 | <--- | AI自我效能 | .038 | .001 | .676 | .001 | .001 |
| SE2 | <--- | AI自我效能 | .037 | .001 | .714 | -.001 | .001 |
| SE3 | <--- | AI自我效能 | .039 | .001 | .673 | .000 | .001 |
| SE4 | <--- | AI自我效能 | .027 | .001 | .790 | .000 | .001 |
| SE5 | <--- | AI自我效能 | .027 | .001 | .799 | -.003 | .001 |
| AB1 | <--- | 使用行为 | .032 | .001 | .772 | -.001 | .001 |
| AB2 | <--- | 使用行为 | .036 | .001 | .718 | -.001 | .001 |
| AB3 | <--- | 使用行为 | .027 | .001 | .813 | .001 | .001 |
| AB5 | <--- | 使用行为 | .034 | .001 | .741 | -.001 | .001 |

##### Variances: (Middle - Default model)

| Parameter | | | SE | SE-SE | Mean | Bias | SE-Bias |
| --- | --- | --- | --- | --- | --- | --- | --- |
| AI威胁感知 |  |  | .214 | .005 | 2.203 | -.005 | .007 |
| e21 |  |  | .160 | .004 | 1.017 | .007 | .005 |
| e23 |  |  | .191 | .004 | 1.119 | -.016 | .006 |
| e22 |  |  | .158 | .004 | 1.128 | -.015 | .005 |
| e1 |  |  | .125 | .003 | 1.042 | -.003 | .004 |
| e2 |  |  | .116 | .003 | 1.196 | -.011 | .004 |
| e3 |  |  | .129 | .003 | 1.477 | -.014 | .004 |
| e4 |  |  | .133 | .003 | 1.356 | -.004 | .004 |
| e5 |  |  | .146 | .003 | 1.175 | -.022 | .005 |
| e6 |  |  | .189 | .004 | 1.764 | -.010 | .006 |
| e8 |  |  | .159 | .004 | 1.438 | -.006 | .005 |
| e9 |  |  | .153 | .003 | 1.344 | -.012 | .005 |
| e10 |  |  | .165 | .004 | 1.667 | -.009 | .005 |
| e11 |  |  | .153 | .003 | 1.802 | -.017 | .005 |
| e12 |  |  | .198 | .004 | 1.723 | -.011 | .006 |
| e13 |  |  | .162 | .004 | 1.794 | -.010 | .005 |
| e14 |  |  | .128 | .003 | 1.207 | -.010 | .004 |
| e15 |  |  | .128 | .003 | 1.128 | .006 | .004 |
| e16 |  |  | .154 | .003 | 1.351 | -.006 | .005 |
| e17 |  |  | .167 | .004 | 1.599 | -.008 | .005 |
| e18 |  |  | .133 | .003 | 1.108 | -.012 | .004 |
| e20 |  |  | .152 | .003 | 1.508 | -.014 | .005 |

##### Squared Multiple Correlations: (Middle - Default model)

| Parameter | | | SE | SE-SE | Mean | Bias | SE-Bias |
| --- | --- | --- | --- | --- | --- | --- | --- |
| AI自我效能 |  |  | .053 | .001 | .335 | -.001 | .002 |
| 防御性动机 |  |  | .058 | .001 | .298 | .010 | .002 |
| 使用行为 |  |  | .058 | .001 | .439 | .003 | .002 |
| AB5 |  |  | .050 | .001 | .551 | -.001 | .002 |
| AB3 |  |  | .044 | .001 | .662 | .002 | .001 |
| AB2 |  |  | .052 | .001 | .517 | .000 | .002 |
| AB1 |  |  | .049 | .001 | .598 | -.001 | .002 |
| SE5 |  |  | .042 | .001 | .639 | -.003 | .001 |
| SE4 |  |  | .042 | .001 | .625 | .001 | .001 |
| SE3 |  |  | .052 | .001 | .455 | .001 | .002 |
| SE2 |  |  | .053 | .001 | .512 | .000 | .002 |
| SE1 |  |  | .051 | .001 | .458 | .003 | .002 |
| DM5 |  |  | .053 | .001 | .493 | .000 | .002 |
| DM3 |  |  | .052 | .001 | .580 | .000 | .002 |
| DM2 |  |  | .052 | .001 | .556 | -.001 | .002 |
| DM1 |  |  | .058 | .001 | .474 | .000 | .002 |
| AA5 |  |  | .049 | .001 | .616 | .004 | .002 |
| AA4 |  |  | .044 | .001 | .585 | -.003 | .001 |
| AA3 |  |  | .044 | .001 | .559 | .000 | .001 |
| AA2 |  |  | .038 | .001 | .624 | .002 | .001 |
| AA1 |  |  | .042 | .001 | .678 | -.001 | .001 |

##### User-defined estimands: (Middle - Default model)

| Parameter | | | SE | SE-SE | Mean | Bias | SE-Bias |
| --- | --- | --- | --- | --- | --- | --- | --- |
| diff1 |  |  | .100 | .002 | .333 | -.002 | .003 |
| diff2 |  |  | .108 | .002 | .233 | .000 | .003 |
| diff3 |  |  | .118 | .003 | .365 | -.004 | .004 |
| diff4 |  |  | .115 | .003 | .236 | -.003 | .004 |
| diff5 |  |  | .123 | .003 | -.008 | -.003 | .004 |

##### Bootstrap Confidence (Middle - Default model)

##### Percentile method (Middle - Default model)

##### 95% confidence intervals (percentile method)

##### Scalar Estimates (Middle - Default model)

##### Regression Weights: (Middle - Default model)

| Parameter | | | Estimate | Lower | Upper | P |
| --- | --- | --- | --- | --- | --- | --- |
| AI自我效能 | <--- | AI威胁感知 | .481 | .371 | .594 | .002 |
| 防御性动机 | <--- | AI威胁感知 | .378 | .240 | .534 | .002 |
| 防御性动机 | <--- | AI自我效能 | .141 | -.029 | .311 | .102 |
| 使用行为 | <--- | 防御性动机 | .384 | .230 | .539 | .002 |
| 使用行为 | <--- | AI自我效能 | .515 | .351 | .682 | .002 |
| AA1 | <--- | AI威胁感知 | 1.000 | 1.000 | 1.000 | ... |
| AA2 | <--- | AI威胁感知 | .950 | .853 | 1.058 | .002 |
| AA3 | <--- | AI威胁感知 | .926 | .819 | 1.037 | .002 |
| AA4 | <--- | AI威胁感知 | .936 | .821 | 1.055 | .002 |
| AA5 | <--- | AI威胁感知 | .926 | .815 | 1.053 | .002 |
| DM1 | <--- | 防御性动机 | 1.000 | 1.000 | 1.000 | ... |
| DM2 | <--- | 防御性动机 | 1.068 | .897 | 1.301 | .002 |
| DM3 | <--- | 防御性动机 | 1.082 | .919 | 1.305 | .002 |
| DM5 | <--- | 防御性动机 | 1.013 | .834 | 1.252 | .002 |
| SE1 | <--- | AI自我效能 | 1.000 | 1.000 | 1.000 | ... |
| SE2 | <--- | AI自我效能 | 1.093 | .912 | 1.281 | .002 |
| SE3 | <--- | AI自我效能 | .992 | .835 | 1.173 | .002 |
| SE4 | <--- | AI自我效能 | 1.152 | .985 | 1.350 | .002 |
| SE5 | <--- | AI自我效能 | 1.152 | .975 | 1.345 | .002 |
| AB1 | <--- | 使用行为 | 1.000 | 1.000 | 1.000 | ... |
| AB2 | <--- | 使用行为 | .923 | .804 | 1.065 | .002 |
| AB3 | <--- | 使用行为 | 1.036 | .923 | 1.198 | .002 |
| AB5 | <--- | 使用行为 | .962 | .830 | 1.114 | .002 |

##### Standardized Regression Weights: (Middle - Default model)

| Parameter | | | Estimate | Lower | Upper | P |
| --- | --- | --- | --- | --- | --- | --- |
| AI自我效能 | <--- | AI威胁感知 | .580 | .484 | .660 | .002 |
| 防御性动机 | <--- | AI威胁感知 | .445 | .286 | .602 | .002 |
| 防御性动机 | <--- | AI自我效能 | .138 | -.029 | .298 | .102 |
| 使用行为 | <--- | 防御性动机 | .341 | .200 | .459 | .002 |
| 使用行为 | <--- | AI自我效能 | .446 | .310 | .566 | .002 |
| AA1 | <--- | AI威胁感知 | .824 | .767 | .872 | .002 |
| AA2 | <--- | AI威胁感知 | .789 | .737 | .836 | .002 |
| AA3 | <--- | AI威胁感知 | .748 | .685 | .800 | .002 |
| AA4 | <--- | AI威胁感知 | .766 | .704 | .819 | .002 |
| AA5 | <--- | AI威胁感知 | .783 | .722 | .843 | .002 |
| DM1 | <--- | 防御性动机 | .688 | .596 | .764 | .002 |
| DM2 | <--- | 防御性动机 | .747 | .675 | .806 | .002 |
| DM3 | <--- | 防御性动机 | .761 | .693 | .825 | .002 |
| DM5 | <--- | 防御性动机 | .703 | .623 | .771 | .002 |
| SE1 | <--- | AI自我效能 | .675 | .602 | .746 | .002 |
| SE2 | <--- | AI自我效能 | .715 | .633 | .787 | .002 |
| SE3 | <--- | AI自我效能 | .674 | .591 | .746 | .002 |
| SE4 | <--- | AI自我效能 | .790 | .733 | .841 | .002 |
| SE5 | <--- | AI自我效能 | .802 | .742 | .845 | .002 |
| AB1 | <--- | 使用行为 | .774 | .700 | .827 | .002 |
| AB2 | <--- | 使用行为 | .719 | .646 | .783 | .002 |
| AB3 | <--- | 使用行为 | .812 | .755 | .860 | .002 |
| AB5 | <--- | 使用行为 | .743 | .667 | .804 | .002 |

##### Variances: (Middle - Default model)

| Parameter | | | Estimate | Lower | Upper | P |
| --- | --- | --- | --- | --- | --- | --- |
| AI威胁感知 |  |  | 2.208 | 1.769 | 2.633 | .002 |
| e21 |  |  | 1.010 | .740 | 1.334 | .002 |
| e23 |  |  | 1.135 | .773 | 1.521 | .002 |
| e22 |  |  | 1.143 | .833 | 1.442 | .002 |
| e1 |  |  | 1.045 | .802 | 1.298 | .002 |
| e2 |  |  | 1.208 | .982 | 1.435 | .002 |
| e3 |  |  | 1.491 | 1.236 | 1.735 | .002 |
| e4 |  |  | 1.360 | 1.107 | 1.635 | .002 |
| e5 |  |  | 1.197 | .908 | 1.476 | .002 |
| e6 |  |  | 1.774 | 1.407 | 2.148 | .002 |
| e8 |  |  | 1.444 | 1.136 | 1.759 | .002 |
| e9 |  |  | 1.355 | 1.039 | 1.630 | .002 |
| e10 |  |  | 1.676 | 1.336 | 1.981 | .002 |
| e11 |  |  | 1.819 | 1.499 | 2.118 | .002 |
| e12 |  |  | 1.733 | 1.312 | 2.122 | .002 |
| e13 |  |  | 1.804 | 1.484 | 2.124 | .002 |
| e14 |  |  | 1.217 | .947 | 1.461 | .002 |
| e15 |  |  | 1.122 | .899 | 1.399 | .002 |
| e16 |  |  | 1.357 | 1.066 | 1.650 | .002 |
| e17 |  |  | 1.607 | 1.294 | 1.951 | .002 |
| e18 |  |  | 1.120 | .859 | 1.363 | .002 |
| e20 |  |  | 1.521 | 1.198 | 1.839 | .002 |

##### Squared Multiple Correlations: (Middle - Default model)

| Parameter | | | Estimate | Lower | Upper | P |
| --- | --- | --- | --- | --- | --- | --- |
| AI自我效能 |  |  | .336 | .234 | .436 | .002 |
| 防御性动机 |  |  | .288 | .182 | .417 | .002 |
| 使用行为 |  |  | .435 | .318 | .557 | .002 |
| AB5 |  |  | .552 | .445 | .646 | .002 |
| AB3 |  |  | .660 | .571 | .740 | .002 |
| AB2 |  |  | .518 | .418 | .613 | .002 |
| AB1 |  |  | .599 | .489 | .684 | .002 |
| SE5 |  |  | .643 | .550 | .714 | .002 |
| SE4 |  |  | .624 | .537 | .708 | .002 |
| SE3 |  |  | .454 | .349 | .557 | .002 |
| SE2 |  |  | .512 | .401 | .620 | .002 |
| SE1 |  |  | .455 | .363 | .557 | .002 |
| DM5 |  |  | .494 | .388 | .595 | .002 |
| DM3 |  |  | .579 | .480 | .680 | .002 |
| DM2 |  |  | .557 | .455 | .650 | .002 |
| DM1 |  |  | .473 | .355 | .584 | .002 |
| AA5 |  |  | .612 | .521 | .711 | .002 |
| AA4 |  |  | .587 | .495 | .670 | .002 |
| AA3 |  |  | .559 | .469 | .641 | .002 |
| AA2 |  |  | .622 | .543 | .698 | .002 |
| AA1 |  |  | .679 | .589 | .761 | .002 |

##### User-defined estimands: (Middle - Default model)

| Parameter | | | Estimate | Lower | Upper | P |
| --- | --- | --- | --- | --- | --- | --- |
| diff1 |  |  | .335 | .130 | .527 | .002 |
| diff2 |  |  | .232 | .020 | .450 | .029 |
| diff3 |  |  | .369 | .124 | .600 | .003 |
| diff4 |  |  | .238 | -.001 | .453 | .051 |
| diff5 |  |  | -.005 | -.263 | .229 | .958 |

##### Bias-corrected percentile method (Middle - Default model)

##### 95% confidence intervals (bias-corrected percentile method)

##### Scalar Estimates (Middle - Default model)

##### Regression Weights: (Middle - Default model)

| Parameter | | | Estimate | Lower | Upper | P |
| --- | --- | --- | --- | --- | --- | --- |
| AI自我效能 | <--- | AI威胁感知 | .481 | .377 | .602 | .001 |
| 防御性动机 | <--- | AI威胁感知 | .378 | .236 | .526 | .002 |
| 防御性动机 | <--- | AI自我效能 | .141 | -.035 | .310 | .104 |
| 使用行为 | <--- | 防御性动机 | .384 | .224 | .538 | .002 |
| 使用行为 | <--- | AI自我效能 | .515 | .368 | .703 | .001 |
| AA1 | <--- | AI威胁感知 | 1.000 | 1.000 | 1.000 | ... |
| AA2 | <--- | AI威胁感知 | .950 | .848 | 1.054 | .003 |
| AA3 | <--- | AI威胁感知 | .926 | .824 | 1.046 | .001 |
| AA4 | <--- | AI威胁感知 | .936 | .826 | 1.060 | .001 |
| AA5 | <--- | AI威胁感知 | .926 | .811 | 1.048 | .002 |
| DM1 | <--- | 防御性动机 | 1.000 | 1.000 | 1.000 | ... |
| DM2 | <--- | 防御性动机 | 1.068 | .893 | 1.299 | .002 |
| DM3 | <--- | 防御性动机 | 1.082 | .916 | 1.299 | .002 |
| DM5 | <--- | 防御性动机 | 1.013 | .834 | 1.253 | .002 |
| SE1 | <--- | AI自我效能 | 1.000 | 1.000 | 1.000 | ... |
| SE2 | <--- | AI自我效能 | 1.093 | .932 | 1.298 | .001 |
| SE3 | <--- | AI自我效能 | .992 | .835 | 1.173 | .002 |
| SE4 | <--- | AI自我效能 | 1.152 | .995 | 1.363 | .001 |
| SE5 | <--- | AI自我效能 | 1.152 | .981 | 1.347 | .002 |
| AB1 | <--- | 使用行为 | 1.000 | 1.000 | 1.000 | ... |
| AB2 | <--- | 使用行为 | .923 | .801 | 1.060 | .002 |
| AB3 | <--- | 使用行为 | 1.036 | .924 | 1.202 | .002 |
| AB5 | <--- | 使用行为 | .962 | .835 | 1.134 | .001 |

##### Standardized Regression Weights: (Middle - Default model)

| Parameter | | | Estimate | Lower | Upper | P |
| --- | --- | --- | --- | --- | --- | --- |
| AI自我效能 | <--- | AI威胁感知 | .580 | .487 | .664 | .002 |
| 防御性动机 | <--- | AI威胁感知 | .445 | .268 | .583 | .004 |
| 防御性动机 | <--- | AI自我效能 | .138 | -.040 | .294 | .120 |
| 使用行为 | <--- | 防御性动机 | .341 | .200 | .459 | .002 |
| 使用行为 | <--- | AI自我效能 | .446 | .304 | .561 | .003 |
| AA1 | <--- | AI威胁感知 | .824 | .767 | .870 | .003 |
| AA2 | <--- | AI威胁感知 | .789 | .735 | .831 | .003 |
| AA3 | <--- | AI威胁感知 | .748 | .685 | .803 | .002 |
| AA4 | <--- | AI威胁感知 | .766 | .709 | .820 | .001 |
| AA5 | <--- | AI威胁感知 | .783 | .716 | .839 | .003 |
| DM1 | <--- | 防御性动机 | .688 | .596 | .764 | .002 |
| DM2 | <--- | 防御性动机 | .747 | .676 | .807 | .002 |
| DM3 | <--- | 防御性动机 | .761 | .693 | .825 | .002 |
| DM5 | <--- | 防御性动机 | .703 | .627 | .775 | .002 |
| SE1 | <--- | AI自我效能 | .675 | .597 | .745 | .003 |
| SE2 | <--- | AI自我效能 | .715 | .631 | .785 | .002 |
| SE3 | <--- | AI自我效能 | .674 | .584 | .742 | .003 |
| SE4 | <--- | AI自我效能 | .790 | .731 | .840 | .002 |
| SE5 | <--- | AI自我效能 | .802 | .744 | .847 | .001 |
| AB1 | <--- | 使用行为 | .774 | .700 | .827 | .002 |
| AB2 | <--- | 使用行为 | .719 | .648 | .784 | .002 |
| AB3 | <--- | 使用行为 | .812 | .749 | .857 | .003 |
| AB5 | <--- | 使用行为 | .743 | .665 | .802 | .002 |

##### Variances: (Middle - Default model)

| Parameter | | | Estimate | Lower | Upper | P |
| --- | --- | --- | --- | --- | --- | --- |
| AI威胁感知 |  |  | 2.208 | 1.780 | 2.665 | .002 |
| e21 |  |  | 1.010 | .741 | 1.355 | .002 |
| e23 |  |  | 1.135 | .813 | 1.571 | .001 |
| e22 |  |  | 1.143 | .866 | 1.468 | .001 |
| e1 |  |  | 1.045 | .819 | 1.305 | .001 |
| e2 |  |  | 1.208 | 1.013 | 1.507 | .001 |
| e3 |  |  | 1.491 | 1.266 | 1.771 | .001 |
| e4 |  |  | 1.360 | 1.129 | 1.654 | .001 |
| e5 |  |  | 1.197 | .951 | 1.530 | .001 |
| e6 |  |  | 1.774 | 1.431 | 2.177 | .001 |
| e8 |  |  | 1.444 | 1.168 | 1.784 | .001 |
| e9 |  |  | 1.355 | 1.056 | 1.642 | .001 |
| e10 |  |  | 1.676 | 1.337 | 1.984 | .002 |
| e11 |  |  | 1.819 | 1.537 | 2.146 | .001 |
| e12 |  |  | 1.733 | 1.367 | 2.168 | .001 |
| e13 |  |  | 1.804 | 1.503 | 2.166 | .001 |
| e14 |  |  | 1.217 | .974 | 1.495 | .001 |
| e15 |  |  | 1.122 | .907 | 1.409 | .002 |
| e16 |  |  | 1.357 | 1.072 | 1.673 | .002 |
| e17 |  |  | 1.607 | 1.304 | 1.968 | .001 |
| e18 |  |  | 1.120 | .889 | 1.388 | .001 |
| e20 |  |  | 1.521 | 1.256 | 1.892 | .001 |

##### Squared Multiple Correlations: (Middle - Default model)

| Parameter | | | Estimate | Lower | Upper | P |
| --- | --- | --- | --- | --- | --- | --- |
| AI自我效能 |  |  | .336 | .237 | .442 | .002 |
| 防御性动机 |  |  | .288 | .162 | .391 | .006 |
| 使用行为 |  |  | .435 | .311 | .551 | .003 |
| AB5 |  |  | .552 | .442 | .643 | .002 |
| AB3 |  |  | .660 | .561 | .735 | .003 |
| AB2 |  |  | .518 | .419 | .615 | .002 |
| AB1 |  |  | .599 | .491 | .685 | .002 |
| SE5 |  |  | .643 | .554 | .717 | .001 |
| SE4 |  |  | .624 | .535 | .706 | .002 |
| SE3 |  |  | .454 | .341 | .551 | .003 |
| SE2 |  |  | .512 | .399 | .616 | .002 |
| SE1 |  |  | .455 | .356 | .555 | .003 |
| DM5 |  |  | .494 | .393 | .601 | .002 |
| DM3 |  |  | .579 | .480 | .680 | .002 |
| DM2 |  |  | .557 | .457 | .651 | .002 |
| DM1 |  |  | .473 | .355 | .584 | .002 |
| AA5 |  |  | .612 | .512 | .703 | .003 |
| AA4 |  |  | .587 | .502 | .672 | .001 |
| AA3 |  |  | .559 | .470 | .645 | .002 |
| AA2 |  |  | .622 | .540 | .691 | .003 |
| AA1 |  |  | .679 | .588 | .756 | .003 |

##### User-defined estimands: (Middle - Default model)

| Parameter | | | Estimate | Lower | Upper | P |
| --- | --- | --- | --- | --- | --- | --- |
| diff1 |  |  | .335 | .130 | .527 | .002 |
| diff2 |  |  | .232 | .023 | .456 | .027 |
| diff3 |  |  | .369 | .126 | .605 | .002 |
| diff4 |  |  | .238 | .006 | .459 | .044 |
| diff5 |  |  | -.005 | -.262 | .229 | .978 |

##### others (others - Default model)

##### Estimates (others - Default model)

##### Scalar Estimates (others - Default model)

##### Maximum Likelihood Estimates

##### Regression Weights: (others - Default model)

|  |  |  | Estimate | S.E. | C.R. | P | Label |
| --- | --- | --- | --- | --- | --- | --- | --- |
| AI自我效能 | <--- | AI威胁感知 | .146 | .074 | 1.967 | .049 | H1 |
| 防御性动机 | <--- | AI威胁感知 | .378 | .116 | 3.250 | .001 | H2 |
| 防御性动机 | <--- | AI自我效能 | -.163 | .176 | -.927 | .354 | H5 |
| 使用行为 | <--- | 防御性动机 | .399 | .108 | 3.706 | \*\*\* | H4 |
| 使用行为 | <--- | AI自我效能 | .340 | .156 | 2.182 | .029 | H3 |
| AA1 | <--- | AI威胁感知 | 1.000 |  |  |  |  |
| AA2 | <--- | AI威胁感知 | .952 | .115 | 8.265 | \*\*\* |  |
| AA3 | <--- | AI威胁感知 | .893 | .110 | 8.081 | \*\*\* |  |
| AA4 | <--- | AI威胁感知 | .871 | .116 | 7.537 | \*\*\* |  |
| AA5 | <--- | AI威胁感知 | .995 | .117 | 8.516 | \*\*\* |  |
| DM1 | <--- | 防御性动机 | 1.000 |  |  |  |  |
| DM2 | <--- | 防御性动机 | .896 | .131 | 6.824 | \*\*\* |  |
| DM3 | <--- | 防御性动机 | .928 | .132 | 7.038 | \*\*\* |  |
| DM5 | <--- | 防御性动机 | .920 | .132 | 6.992 | \*\*\* |  |
| SE1 | <--- | AI自我效能 | 1.000 |  |  |  |  |
| SE2 | <--- | AI自我效能 | 1.368 | .273 | 5.007 | \*\*\* |  |
| SE3 | <--- | AI自我效能 | 1.493 | .283 | 5.271 | \*\*\* |  |
| SE4 | <--- | AI自我效能 | 1.709 | .314 | 5.441 | \*\*\* |  |
| SE5 | <--- | AI自我效能 | 1.479 | .281 | 5.259 | \*\*\* |  |
| AB1 | <--- | 使用行为 | 1.000 |  |  |  |  |
| AB2 | <--- | 使用行为 | 1.156 | .160 | 7.222 | \*\*\* |  |
| AB3 | <--- | 使用行为 | 1.124 | .160 | 7.043 | \*\*\* |  |
| AB5 | <--- | 使用行为 | .932 | .154 | 6.059 | \*\*\* |  |

##### Standardized Regression Weights: (others - Default model)

|  |  |  | Estimate |
| --- | --- | --- | --- |
| AI自我效能 | <--- | AI威胁感知 | .230 |
| 防御性动机 | <--- | AI威胁感知 | .389 |
| 防御性动机 | <--- | AI自我效能 | -.106 |
| 使用行为 | <--- | 防御性动机 | .448 |
| 使用行为 | <--- | AI自我效能 | .249 |
| AA1 | <--- | AI威胁感知 | .807 |
| AA2 | <--- | AI威胁感知 | .767 |
| AA3 | <--- | AI威胁感知 | .753 |
| AA4 | <--- | AI威胁感知 | .710 |
| AA5 | <--- | AI威胁感知 | .787 |
| DM1 | <--- | 防御性动机 | .769 |
| DM2 | <--- | 防御性动机 | .715 |
| DM3 | <--- | 防御性动机 | .739 |
| DM5 | <--- | 防御性动机 | .734 |
| SE1 | <--- | AI自我效能 | .535 |
| SE2 | <--- | AI自我效能 | .701 |
| SE3 | <--- | AI自我效能 | .774 |
| SE4 | <--- | AI自我效能 | .834 |
| SE5 | <--- | AI自我效能 | .771 |
| AB1 | <--- | 使用行为 | .720 |
| AB2 | <--- | 使用行为 | .810 |
| AB3 | <--- | 使用行为 | .780 |
| AB5 | <--- | 使用行为 | .657 |

##### Variances: (others - Default model)

|  |  |  | Estimate | S.E. | C.R. | P | Label |
| --- | --- | --- | --- | --- | --- | --- | --- |
| AI威胁感知 |  |  | 2.140 | .449 | 4.763 | \*\*\* |  |
| e21 |  |  | .813 | .294 | 2.761 | .006 |  |
| e23 |  |  | 1.728 | .414 | 4.175 | \*\*\* |  |
| e22 |  |  | 1.186 | .315 | 3.763 | \*\*\* |  |
| e1 |  |  | 1.143 | .213 | 5.354 | \*\*\* |  |
| e2 |  |  | 1.355 | .234 | 5.802 | \*\*\* |  |
| e3 |  |  | 1.304 | .220 | 5.925 | \*\*\* |  |
| e4 |  |  | 1.592 | .256 | 6.219 | \*\*\* |  |
| e5 |  |  | 1.304 | .233 | 5.604 | \*\*\* |  |
| e6 |  |  | 1.391 | .271 | 5.140 | \*\*\* |  |
| e8 |  |  | 1.549 | .270 | 5.730 | \*\*\* |  |
| e9 |  |  | 1.440 | .262 | 5.496 | \*\*\* |  |
| e10 |  |  | 1.461 | .263 | 5.551 | \*\*\* |  |
| e11 |  |  | 2.142 | .315 | 6.792 | \*\*\* |  |
| e12 |  |  | 1.661 | .270 | 6.160 | \*\*\* |  |
| e13 |  |  | 1.278 | .230 | 5.546 | \*\*\* |  |
| e14 |  |  | 1.094 | .235 | 4.660 | \*\*\* |  |
| e15 |  |  | 1.283 | .230 | 5.585 | \*\*\* |  |
| e16 |  |  | 1.490 | .258 | 5.773 | \*\*\* |  |
| e17 |  |  | 1.121 | .243 | 4.614 | \*\*\* |  |
| e18 |  |  | 1.306 | .256 | 5.092 | \*\*\* |  |
| e20 |  |  | 1.831 | .295 | 6.213 | \*\*\* |  |

##### Squared Multiple Correlations: (others - Default model)

|  |  |  | Estimate |
| --- | --- | --- | --- |
| AI自我效能 |  |  | .053 |
| 防御性动机 |  |  | .144 |
| 使用行为 |  |  | .259 |
| AB5 |  |  | .431 |
| AB3 |  |  | .608 |
| AB2 |  |  | .656 |
| AB1 |  |  | .518 |
| SE5 |  |  | .594 |
| SE4 |  |  | .696 |
| SE3 |  |  | .600 |
| SE2 |  |  | .491 |
| SE1 |  |  | .286 |
| DM5 |  |  | .539 |
| DM3 |  |  | .547 |
| DM2 |  |  | .511 |
| DM1 |  |  | .592 |
| AA5 |  |  | .619 |
| AA4 |  |  | .505 |
| AA3 |  |  | .567 |
| AA2 |  |  | .588 |
| AA1 |  |  | .652 |

##### User-defined estimands: (others - Default model)

|  |  |  |  |
| --- | --- | --- | --- |
| diff1 |  |  | .335 |
| diff2 |  |  | .232 |
| diff3 |  |  | .369 |
| diff4 |  |  | .238 |
| diff5 |  |  | -.005 |

##### Modification Indices (others - Default model)

##### Covariances: (others - Default model)

|  |  |  | M.I. | Par Change |
| --- | --- | --- | --- | --- |
| e20 | <--> | e21 | 5.872 | .332 |
| e17 | <--> | e23 | 5.180 | .411 |
| e14 | <--> | AI威胁感知 | 4.781 | .415 |
| e13 | <--> | e20 | 4.054 | .357 |
| e11 | <--> | e20 | 6.999 | -.560 |
| e10 | <--> | e20 | 8.003 | -.534 |
| e9 | <--> | e13 | 5.223 | -.376 |
| e5 | <--> | e16 | 4.297 | -.344 |
| e5 | <--> | e8 | 5.436 | .396 |
| e3 | <--> | e21 | 4.839 | .260 |

##### Variances: (others - Default model)

|  |  |  | M.I. | Par Change |
| --- | --- | --- | --- | --- |

##### Regression Weights: (others - Default model)

|  |  |  | M.I. | Par Change |
| --- | --- | --- | --- | --- |
| AB5 | <--- | AI自我效能 | 6.270 | .408 |
| AB5 | <--- | SE4 | 5.931 | .181 |
| AB5 | <--- | SE3 | 8.724 | .233 |
| AB5 | <--- | SE2 | 4.452 | .164 |
| AB5 | <--- | DM5 | 5.548 | -.186 |
| AB2 | <--- | 防御性动机 | 4.047 | .190 |
| AB2 | <--- | SE3 | 4.269 | -.142 |
| AB2 | <--- | DM5 | 4.553 | .147 |
| AB1 | <--- | AA5 | 5.438 | -.165 |
| SE4 | <--- | AI威胁感知 | 4.781 | .194 |
| SE4 | <--- | DM3 | 4.677 | .148 |
| SE4 | <--- | AA4 | 5.158 | .155 |
| SE4 | <--- | AA3 | 6.277 | .176 |
| SE4 | <--- | AA2 | 5.192 | .153 |
| SE3 | <--- | AA2 | 4.095 | -.137 |
| SE1 | <--- | AB5 | 4.118 | -.166 |
| DM5 | <--- | AB5 | 4.550 | -.156 |
| DM3 | <--- | SE4 | 5.261 | .158 |
| AA5 | <--- | AB1 | 4.012 | -.142 |
| AA3 | <--- | AI自我效能 | 4.525 | .299 |
| AA3 | <--- | SE4 | 5.711 | .153 |

##### Bootstrap (others - Default model)

##### Bootstrap standard errors (others - Default model)

##### Scalar Estimates (others - Default model)

##### Regression Weights: (others - Default model)

| Parameter | | | SE | SE-SE | Mean | Bias | SE-Bias |
| --- | --- | --- | --- | --- | --- | --- | --- |
| AI自我效能 | <--- | AI威胁感知 | .081 | .002 | .148 | .002 | .003 |
| 防御性动机 | <--- | AI威胁感知 | .112 | .003 | .372 | -.006 | .004 |
| 防御性动机 | <--- | AI自我效能 | .198 | .004 | -.171 | -.008 | .006 |
| 使用行为 | <--- | 防御性动机 | .099 | .002 | .396 | -.003 | .003 |
| 使用行为 | <--- | AI自我效能 | .195 | .004 | .362 | .022 | .006 |
| AA1 | <--- | AI威胁感知 | .000 | .000 | 1.000 | .000 | .000 |
| AA2 | <--- | AI威胁感知 | .104 | .002 | .951 | -.001 | .003 |
| AA3 | <--- | AI威胁感知 | .076 | .002 | .895 | .003 | .002 |
| AA4 | <--- | AI威胁感知 | .116 | .003 | .866 | -.005 | .004 |
| AA5 | <--- | AI威胁感知 | .114 | .003 | 1.000 | .005 | .004 |
| DM1 | <--- | 防御性动机 | .000 | .000 | 1.000 | .000 | .000 |
| DM2 | <--- | 防御性动机 | .135 | .003 | .902 | .006 | .004 |
| DM3 | <--- | 防御性动机 | .145 | .003 | .929 | .002 | .005 |
| DM5 | <--- | 防御性动机 | .108 | .002 | .925 | .005 | .003 |
| SE1 | <--- | AI自我效能 | .000 | .000 | 1.000 | .000 | .000 |
| SE2 | <--- | AI自我效能 | .287 | .006 | 1.407 | .039 | .009 |
| SE3 | <--- | AI自我效能 | .304 | .007 | 1.545 | .052 | .010 |
| SE4 | <--- | AI自我效能 | .339 | .008 | 1.764 | .056 | .011 |
| SE5 | <--- | AI自我效能 | .298 | .007 | 1.532 | .052 | .009 |
| AB1 | <--- | 使用行为 | .000 | .000 | 1.000 | .000 | .000 |
| AB2 | <--- | 使用行为 | .161 | .004 | 1.171 | .015 | .005 |
| AB3 | <--- | 使用行为 | .159 | .004 | 1.137 | .012 | .005 |
| AB5 | <--- | 使用行为 | .146 | .003 | .942 | .011 | .005 |

##### Standardized Regression Weights: (others - Default model)

| Parameter | | | SE | SE-SE | Mean | Bias | SE-Bias |
| --- | --- | --- | --- | --- | --- | --- | --- |
| AI自我效能 | <--- | AI威胁感知 | .117 | .003 | .232 | .001 | .004 |
| 防御性动机 | <--- | AI威胁感知 | .104 | .002 | .380 | -.009 | .003 |
| 防御性动机 | <--- | AI自我效能 | .117 | .003 | -.105 | .001 | .004 |
| 使用行为 | <--- | 防御性动机 | .099 | .002 | .444 | -.004 | .003 |
| 使用行为 | <--- | AI自我效能 | .111 | .002 | .251 | .002 | .004 |
| AA1 | <--- | AI威胁感知 | .048 | .001 | .806 | -.001 | .002 |
| AA2 | <--- | AI威胁感知 | .058 | .001 | .762 | -.005 | .002 |
| AA3 | <--- | AI威胁感知 | .053 | .001 | .753 | .000 | .002 |
| AA4 | <--- | AI威胁感知 | .068 | .002 | .702 | -.009 | .002 |
| AA5 | <--- | AI威胁感知 | .049 | .001 | .785 | -.002 | .002 |
| DM1 | <--- | 防御性动机 | .058 | .001 | .770 | .000 | .002 |
| DM2 | <--- | 防御性动机 | .067 | .002 | .713 | -.002 | .002 |
| DM3 | <--- | 防御性动机 | .061 | .001 | .734 | -.005 | .002 |
| DM5 | <--- | 防御性动机 | .059 | .001 | .733 | -.001 | .002 |
| SE1 | <--- | AI自我效能 | .079 | .002 | .532 | -.003 | .002 |
| SE2 | <--- | AI自我效能 | .063 | .001 | .697 | -.004 | .002 |
| SE3 | <--- | AI自我效能 | .045 | .001 | .775 | .000 | .001 |
| SE4 | <--- | AI自我效能 | .052 | .001 | .832 | -.002 | .002 |
| SE5 | <--- | AI自我效能 | .061 | .001 | .770 | -.001 | .002 |
| AB1 | <--- | 使用行为 | .061 | .001 | .717 | -.003 | .002 |
| AB2 | <--- | 使用行为 | .060 | .001 | .810 | .000 | .002 |
| AB3 | <--- | 使用行为 | .059 | .001 | .780 | .000 | .002 |
| AB5 | <--- | 使用行为 | .068 | .002 | .656 | -.001 | .002 |

##### Variances: (others - Default model)

| Parameter | | | SE | SE-SE | Mean | Bias | SE-Bias |
| --- | --- | --- | --- | --- | --- | --- | --- |
| AI威胁感知 |  |  | .385 | .009 | 2.128 | -.012 | .012 |
| e21 |  |  | .272 | .006 | .806 | -.006 | .009 |
| e23 |  |  | .393 | .009 | 1.706 | -.022 | .012 |
| e22 |  |  | .314 | .007 | 1.162 | -.024 | .010 |
| e1 |  |  | .222 | .005 | 1.118 | -.025 | .007 |
| e2 |  |  | .256 | .006 | 1.338 | -.017 | .008 |
| e3 |  |  | .233 | .005 | 1.269 | -.035 | .007 |
| e4 |  |  | .271 | .006 | 1.585 | -.006 | .009 |
| e5 |  |  | .238 | .005 | 1.280 | -.025 | .008 |
| e6 |  |  | .273 | .006 | 1.357 | -.034 | .009 |
| e8 |  |  | .314 | .007 | 1.528 | -.021 | .010 |
| e9 |  |  | .235 | .005 | 1.422 | -.019 | .007 |
| e10 |  |  | .261 | .006 | 1.437 | -.024 | .008 |
| e11 |  |  | .277 | .006 | 2.099 | -.044 | .009 |
| e12 |  |  | .254 | .006 | 1.639 | -.022 | .008 |
| e13 |  |  | .217 | .005 | 1.250 | -.028 | .007 |
| e14 |  |  | .269 | .006 | 1.075 | -.019 | .009 |
| e15 |  |  | .255 | .006 | 1.255 | -.027 | .008 |
| e16 |  |  | .231 | .005 | 1.472 | -.019 | .007 |
| e17 |  |  | .309 | .007 | 1.098 | -.023 | .010 |
| e18 |  |  | .297 | .007 | 1.272 | -.034 | .009 |
| e20 |  |  | .289 | .006 | 1.804 | -.027 | .009 |

##### Squared Multiple Correlations: (others - Default model)

| Parameter | | | SE | SE-SE | Mean | Bias | SE-Bias |
| --- | --- | --- | --- | --- | --- | --- | --- |
| AI自我效能 |  |  | .054 | .001 | .067 | .014 | .002 |
| 防御性动机 |  |  | .075 | .002 | .161 | .017 | .002 |
| 使用行为 |  |  | .092 | .002 | .277 | .018 | .003 |
| AB5 |  |  | .088 | .002 | .434 | .003 | .003 |
| AB3 |  |  | .090 | .002 | .611 | .004 | .003 |
| AB2 |  |  | .096 | .002 | .659 | .003 | .003 |
| AB1 |  |  | .086 | .002 | .517 | -.001 | .003 |
| SE5 |  |  | .092 | .002 | .597 | .003 | .003 |
| SE4 |  |  | .085 | .002 | .696 | -.001 | .003 |
| SE3 |  |  | .070 | .002 | .602 | .003 | .002 |
| SE2 |  |  | .086 | .002 | .490 | -.001 | .003 |
| SE1 |  |  | .082 | .002 | .289 | .003 | .003 |
| DM5 |  |  | .085 | .002 | .541 | .003 | .003 |
| DM3 |  |  | .088 | .002 | .543 | -.004 | .003 |
| DM2 |  |  | .095 | .002 | .514 | .002 | .003 |
| DM1 |  |  | .089 | .002 | .596 | .004 | .003 |
| AA5 |  |  | .076 | .002 | .619 | .000 | .002 |
| AA4 |  |  | .094 | .002 | .497 | -.008 | .003 |
| AA3 |  |  | .079 | .002 | .570 | .003 | .003 |
| AA2 |  |  | .087 | .002 | .585 | -.004 | .003 |
| AA1 |  |  | .077 | .002 | .652 | .001 | .002 |

##### User-defined estimands: (others - Default model)

| Parameter | | | SE | SE-SE | Mean | Bias | SE-Bias |
| --- | --- | --- | --- | --- | --- | --- | --- |
| diff1 |  |  | .100 | .002 | .333 | -.002 | .003 |
| diff2 |  |  | .108 | .002 | .233 | .000 | .003 |
| diff3 |  |  | .118 | .003 | .365 | -.004 | .004 |
| diff4 |  |  | .115 | .003 | .236 | -.003 | .004 |
| diff5 |  |  | .123 | .003 | -.008 | -.003 | .004 |

##### Bootstrap Confidence (others - Default model)

##### Percentile method (others - Default model)

##### 95% confidence intervals (percentile method)

##### Scalar Estimates (others - Default model)

##### Regression Weights: (others - Default model)

| Parameter | | | Estimate | Lower | Upper | P |
| --- | --- | --- | --- | --- | --- | --- |
| AI自我效能 | <--- | AI威胁感知 | .146 | -.009 | .319 | .064 |
| 防御性动机 | <--- | AI威胁感知 | .378 | .156 | .600 | .003 |
| 防御性动机 | <--- | AI自我效能 | -.163 | -.597 | .221 | .363 |
| 使用行为 | <--- | 防御性动机 | .399 | .205 | .598 | .002 |
| 使用行为 | <--- | AI自我效能 | .340 | .035 | .799 | .029 |
| AA1 | <--- | AI威胁感知 | 1.000 | 1.000 | 1.000 | ... |
| AA2 | <--- | AI威胁感知 | .952 | .746 | 1.161 | .002 |
| AA3 | <--- | AI威胁感知 | .893 | .747 | 1.051 | .002 |
| AA4 | <--- | AI威胁感知 | .871 | .652 | 1.104 | .002 |
| AA5 | <--- | AI威胁感知 | .995 | .800 | 1.234 | .002 |
| DM1 | <--- | 防御性动机 | 1.000 | 1.000 | 1.000 | ... |
| DM2 | <--- | 防御性动机 | .896 | .677 | 1.193 | .002 |
| DM3 | <--- | 防御性动机 | .928 | .661 | 1.223 | .002 |
| DM5 | <--- | 防御性动机 | .920 | .729 | 1.140 | .002 |
| SE1 | <--- | AI自我效能 | 1.000 | 1.000 | 1.000 | ... |
| SE2 | <--- | AI自我效能 | 1.368 | .946 | 2.070 | .002 |
| SE3 | <--- | AI自我效能 | 1.493 | 1.123 | 2.222 | .002 |
| SE4 | <--- | AI自我效能 | 1.709 | 1.278 | 2.544 | .002 |
| SE5 | <--- | AI自我效能 | 1.479 | 1.060 | 2.229 | .002 |
| AB1 | <--- | 使用行为 | 1.000 | 1.000 | 1.000 | ... |
| AB2 | <--- | 使用行为 | 1.156 | .913 | 1.522 | .002 |
| AB3 | <--- | 使用行为 | 1.124 | .880 | 1.493 | .002 |
| AB5 | <--- | 使用行为 | .932 | .684 | 1.238 | .002 |

##### Standardized Regression Weights: (others - Default model)

| Parameter | | | Estimate | Lower | Upper | P |
| --- | --- | --- | --- | --- | --- | --- |
| AI自我效能 | <--- | AI威胁感知 | .230 | -.017 | .446 | .064 |
| 防御性动机 | <--- | AI威胁感知 | .389 | .161 | .572 | .003 |
| 防御性动机 | <--- | AI自我效能 | -.106 | -.318 | .133 | .363 |
| 使用行为 | <--- | 防御性动机 | .448 | .238 | .613 | .002 |
| 使用行为 | <--- | AI自我效能 | .249 | .027 | .459 | .029 |
| AA1 | <--- | AI威胁感知 | .807 | .703 | .891 | .002 |
| AA2 | <--- | AI威胁感知 | .767 | .629 | .861 | .002 |
| AA3 | <--- | AI威胁感知 | .753 | .645 | .845 | .002 |
| AA4 | <--- | AI威胁感知 | .710 | .548 | .821 | .002 |
| AA5 | <--- | AI威胁感知 | .787 | .677 | .868 | .002 |
| DM1 | <--- | 防御性动机 | .769 | .653 | .874 | .002 |
| DM2 | <--- | 防御性动机 | .715 | .575 | .832 | .002 |
| DM3 | <--- | 防御性动机 | .739 | .602 | .841 | .002 |
| DM5 | <--- | 防御性动机 | .734 | .602 | .839 | .002 |
| SE1 | <--- | AI自我效能 | .535 | .369 | .671 | .002 |
| SE2 | <--- | AI自我效能 | .701 | .557 | .812 | .002 |
| SE3 | <--- | AI自我效能 | .774 | .680 | .857 | .002 |
| SE4 | <--- | AI自我效能 | .834 | .722 | .921 | .002 |
| SE5 | <--- | AI自我效能 | .771 | .634 | .879 | .002 |
| AB1 | <--- | 使用行为 | .720 | .585 | .829 | .002 |
| AB2 | <--- | 使用行为 | .810 | .683 | .920 | .002 |
| AB3 | <--- | 使用行为 | .780 | .652 | .879 | .002 |
| AB5 | <--- | 使用行为 | .657 | .509 | .776 | .002 |

##### Variances: (others - Default model)

| Parameter | | | Estimate | Lower | Upper | P |
| --- | --- | --- | --- | --- | --- | --- |
| AI威胁感知 |  |  | 2.140 | 1.410 | 2.910 | .002 |
| e21 |  |  | .813 | .342 | 1.372 | .002 |
| e23 |  |  | 1.728 | 1.000 | 2.536 | .002 |
| e22 |  |  | 1.186 | .625 | 1.904 | .002 |
| e1 |  |  | 1.143 | .698 | 1.587 | .002 |
| e2 |  |  | 1.355 | .877 | 1.892 | .002 |
| e3 |  |  | 1.304 | .843 | 1.768 | .002 |
| e4 |  |  | 1.592 | 1.081 | 2.147 | .002 |
| e5 |  |  | 1.304 | .829 | 1.785 | .002 |
| e6 |  |  | 1.391 | .813 | 1.892 | .002 |
| e8 |  |  | 1.549 | .946 | 2.195 | .002 |
| e9 |  |  | 1.440 | .971 | 1.875 | .002 |
| e10 |  |  | 1.461 | .966 | 1.981 | .002 |
| e11 |  |  | 2.142 | 1.582 | 2.631 | .002 |
| e12 |  |  | 1.661 | 1.180 | 2.158 | .002 |
| e13 |  |  | 1.278 | .844 | 1.690 | .002 |
| e14 |  |  | 1.094 | .544 | 1.587 | .002 |
| e15 |  |  | 1.283 | .755 | 1.776 | .002 |
| e16 |  |  | 1.490 | 1.006 | 1.943 | .002 |
| e17 |  |  | 1.121 | .482 | 1.691 | .002 |
| e18 |  |  | 1.306 | .737 | 1.878 | .002 |
| e20 |  |  | 1.831 | 1.261 | 2.384 | .002 |

##### Squared Multiple Correlations: (others - Default model)

| Parameter | | | Estimate | Lower | Upper | P |
| --- | --- | --- | --- | --- | --- | --- |
| AI自我效能 |  |  | .053 | .001 | .199 | .002 |
| 防御性动机 |  |  | .144 | .038 | .326 | .002 |
| 使用行为 |  |  | .259 | .107 | .462 | .002 |
| AB5 |  |  | .431 | .260 | .603 | .002 |
| AB3 |  |  | .608 | .426 | .773 | .002 |
| AB2 |  |  | .656 | .467 | .846 | .002 |
| AB1 |  |  | .518 | .343 | .687 | .002 |
| SE5 |  |  | .594 | .402 | .772 | .002 |
| SE4 |  |  | .696 | .522 | .848 | .002 |
| SE3 |  |  | .600 | .463 | .734 | .002 |
| SE2 |  |  | .491 | .310 | .660 | .002 |
| SE1 |  |  | .286 | .136 | .450 | .002 |
| DM5 |  |  | .539 | .362 | .704 | .002 |
| DM3 |  |  | .547 | .363 | .707 | .002 |
| DM2 |  |  | .511 | .331 | .692 | .002 |
| DM1 |  |  | .592 | .427 | .764 | .002 |
| AA5 |  |  | .619 | .458 | .753 | .002 |
| AA4 |  |  | .505 | .301 | .674 | .002 |
| AA3 |  |  | .567 | .416 | .714 | .002 |
| AA2 |  |  | .588 | .396 | .741 | .002 |
| AA1 |  |  | .652 | .494 | .794 | .002 |

##### User-defined estimands: (others - Default model)

| Parameter | | | Estimate | Lower | Upper | P |
| --- | --- | --- | --- | --- | --- | --- |
| diff1 |  |  | .335 | .130 | .527 | .002 |
| diff2 |  |  | .232 | .020 | .450 | .029 |
| diff3 |  |  | .369 | .124 | .600 | .003 |
| diff4 |  |  | .238 | -.001 | .453 | .051 |
| diff5 |  |  | -.005 | -.263 | .229 | .958 |

##### Bias-corrected percentile method (others - Default model)

##### 95% confidence intervals (bias-corrected percentile method)

##### Scalar Estimates (others - Default model)

##### Regression Weights: (others - Default model)

| Parameter | | | Estimate | Lower | Upper | P |
| --- | --- | --- | --- | --- | --- | --- |
| AI自我效能 | <--- | AI威胁感知 | .146 | -.010 | .316 | .069 |
| 防御性动机 | <--- | AI威胁感知 | .378 | .185 | .635 | .001 |
| 防御性动机 | <--- | AI自我效能 | -.163 | -.597 | .221 | .363 |
| 使用行为 | <--- | 防御性动机 | .399 | .218 | .627 | .001 |
| 使用行为 | <--- | AI自我效能 | .340 | .045 | .811 | .027 |
| AA1 | <--- | AI威胁感知 | 1.000 | 1.000 | 1.000 | ... |
| AA2 | <--- | AI威胁感知 | .952 | .768 | 1.190 | .001 |
| AA3 | <--- | AI威胁感知 | .893 | .739 | 1.045 | .003 |
| AA4 | <--- | AI威胁感知 | .871 | .675 | 1.125 | .001 |
| AA5 | <--- | AI威胁感知 | .995 | .803 | 1.235 | .002 |
| DM1 | <--- | 防御性动机 | 1.000 | 1.000 | 1.000 | ... |
| DM2 | <--- | 防御性动机 | .896 | .685 | 1.211 | .001 |
| DM3 | <--- | 防御性动机 | .928 | .668 | 1.248 | .001 |
| DM5 | <--- | 防御性动机 | .920 | .730 | 1.140 | .002 |
| SE1 | <--- | AI自我效能 | 1.000 | 1.000 | 1.000 | ... |
| SE2 | <--- | AI自我效能 | 1.368 | .951 | 2.086 | .002 |
| SE3 | <--- | AI自我效能 | 1.493 | 1.121 | 2.203 | .002 |
| SE4 | <--- | AI自我效能 | 1.709 | 1.279 | 2.565 | .002 |
| SE5 | <--- | AI自我效能 | 1.479 | 1.038 | 2.140 | .003 |
| AB1 | <--- | 使用行为 | 1.000 | 1.000 | 1.000 | ... |
| AB2 | <--- | 使用行为 | 1.156 | .925 | 1.560 | .001 |
| AB3 | <--- | 使用行为 | 1.124 | .880 | 1.495 | .002 |
| AB5 | <--- | 使用行为 | .932 | .678 | 1.238 | .002 |

##### Standardized Regression Weights: (others - Default model)

| Parameter | | | Estimate | Lower | Upper | P |
| --- | --- | --- | --- | --- | --- | --- |
| AI自我效能 | <--- | AI威胁感知 | .230 | -.021 | .435 | .077 |
| 防御性动机 | <--- | AI威胁感知 | .389 | .187 | .581 | .001 |
| 防御性动机 | <--- | AI自我效能 | -.106 | -.319 | .130 | .352 |
| 使用行为 | <--- | 防御性动机 | .448 | .233 | .610 | .002 |
| 使用行为 | <--- | AI自我效能 | .249 | .020 | .453 | .033 |
| AA1 | <--- | AI威胁感知 | .807 | .691 | .886 | .003 |
| AA2 | <--- | AI威胁感知 | .767 | .630 | .861 | .002 |
| AA3 | <--- | AI威胁感知 | .753 | .627 | .840 | .003 |
| AA4 | <--- | AI威胁感知 | .710 | .563 | .829 | .001 |
| AA5 | <--- | AI威胁感知 | .787 | .668 | .864 | .003 |
| DM1 | <--- | 防御性动机 | .769 | .647 | .870 | .003 |
| DM2 | <--- | 防御性动机 | .715 | .574 | .831 | .002 |
| DM3 | <--- | 防御性动机 | .739 | .600 | .839 | .002 |
| DM5 | <--- | 防御性动机 | .734 | .586 | .832 | .003 |
| SE1 | <--- | AI自我效能 | .535 | .368 | .669 | .002 |
| SE2 | <--- | AI自我效能 | .701 | .553 | .810 | .002 |
| SE3 | <--- | AI自我效能 | .774 | .677 | .854 | .003 |
| SE4 | <--- | AI自我效能 | .834 | .711 | .917 | .003 |
| SE5 | <--- | AI自我效能 | .771 | .628 | .876 | .003 |
| AB1 | <--- | 使用行为 | .720 | .586 | .830 | .002 |
| AB2 | <--- | 使用行为 | .810 | .677 | .913 | .003 |
| AB3 | <--- | 使用行为 | .780 | .633 | .871 | .004 |
| AB5 | <--- | 使用行为 | .657 | .501 | .770 | .003 |

##### Variances: (others - Default model)

| Parameter | | | Estimate | Lower | Upper | P |
| --- | --- | --- | --- | --- | --- | --- |
| AI威胁感知 |  |  | 2.140 | 1.424 | 2.921 | .002 |
| e21 |  |  | .813 | .374 | 1.456 | .001 |
| e23 |  |  | 1.728 | 1.058 | 2.629 | .001 |
| e22 |  |  | 1.186 | .729 | 2.049 | .001 |
| e1 |  |  | 1.143 | .754 | 1.646 | .001 |
| e2 |  |  | 1.355 | .907 | 1.929 | .001 |
| e3 |  |  | 1.304 | .912 | 1.836 | .001 |
| e4 |  |  | 1.592 | 1.110 | 2.178 | .001 |
| e5 |  |  | 1.304 | .890 | 1.855 | .001 |
| e6 |  |  | 1.391 | .904 | 1.956 | .001 |
| e8 |  |  | 1.549 | .999 | 2.233 | .001 |
| e9 |  |  | 1.440 | .989 | 1.918 | .001 |
| e10 |  |  | 1.461 | 1.038 | 2.085 | .001 |
| e11 |  |  | 2.142 | 1.651 | 2.722 | .001 |
| e12 |  |  | 1.661 | 1.223 | 2.218 | .001 |
| e13 |  |  | 1.278 | .874 | 1.779 | .001 |
| e14 |  |  | 1.094 | .566 | 1.612 | .002 |
| e15 |  |  | 1.283 | .823 | 1.829 | .001 |
| e16 |  |  | 1.490 | 1.048 | 1.986 | .001 |
| e17 |  |  | 1.121 | .517 | 1.740 | .001 |
| e18 |  |  | 1.306 | .813 | 1.923 | .001 |
| e20 |  |  | 1.831 | 1.305 | 2.446 | .001 |

##### Squared Multiple Correlations: (others - Default model)

| Parameter | | | Estimate | Lower | Upper | P |
| --- | --- | --- | --- | --- | --- | --- |
| AI自我效能 |  |  | .053 | .000 | .189 | .003 |
| 防御性动机 |  |  | .144 | .028 | .304 | .004 |
| 使用行为 |  |  | .259 | .100 | .438 | .005 |
| AB5 |  |  | .431 | .251 | .593 | .003 |
| AB3 |  |  | .608 | .401 | .758 | .004 |
| AB2 |  |  | .656 | .459 | .834 | .003 |
| AB1 |  |  | .518 | .344 | .690 | .002 |
| SE5 |  |  | .594 | .394 | .768 | .003 |
| SE4 |  |  | .696 | .506 | .841 | .003 |
| SE3 |  |  | .600 | .458 | .730 | .003 |
| SE2 |  |  | .491 | .306 | .657 | .002 |
| SE1 |  |  | .286 | .136 | .447 | .002 |
| DM5 |  |  | .539 | .343 | .692 | .003 |
| DM3 |  |  | .547 | .361 | .704 | .002 |
| DM2 |  |  | .511 | .329 | .691 | .002 |
| DM1 |  |  | .592 | .419 | .758 | .003 |
| AA5 |  |  | .619 | .446 | .747 | .003 |
| AA4 |  |  | .505 | .316 | .687 | .001 |
| AA3 |  |  | .567 | .393 | .706 | .003 |
| AA2 |  |  | .588 | .397 | .742 | .002 |
| AA1 |  |  | .652 | .477 | .786 | .003 |

##### User-defined estimands: (others - Default model)

| Parameter | | | Estimate | Lower | Upper | P |
| --- | --- | --- | --- | --- | --- | --- |
| diff1 |  |  | .335 | .130 | .527 | .002 |
| diff2 |  |  | .232 | .023 | .456 | .027 |
| diff3 |  |  | .369 | .126 | .605 | .002 |
| diff4 |  |  | .238 | .006 | .459 | .044 |
| diff5 |  |  | -.005 | -.262 | .229 | .978 |

##### Minimization History (Default model)

| Iteration |  | Negative eigenvalues | Condition # | Smallest eigenvalue | Diameter | F | NTries | Ratio |
| --- | --- | --- | --- | --- | --- | --- | --- | --- |
| 0 | e | 11 |  | -.651 | 9999.000 | 3579.686 | 0 | 9999.000 |
| 1 | e | 5 |  | -.076 | 3.394 | 1330.305 | 20 | .393 |
| 2 | e | 0 | 223.423 |  | 2.506 | 496.579 | 5 | .666 |
| 3 | e | 0 | 91.635 |  | 1.525 | 346.043 | 1 | .917 |
| 4 | e | 0 | 99.231 |  | .577 | 293.172 | 1 | 1.126 |
| 5 | e | 0 | 137.758 |  | .368 | 289.277 | 1 | 1.094 |
| 6 | e | 0 | 201.474 |  | .142 | 289.064 | 1 | 1.087 |
| 7 | e | 0 | 214.619 |  | .042 | 289.057 | 1 | 1.033 |
| 8 | e | 0 | 216.072 |  | .002 | 289.057 | 1 | 1.002 |
| 9 | e | 0 | 222.348 |  | .000 | 289.057 | 1 | 1.000 |

##### Bootstrap (Default model)

##### Summary of Bootstrap Iterations (Default model)

##### (Default model)

| Iterations | Method 0 | Method 1 | Method 2 |
| --- | --- | --- | --- |
| 1 | 0 | 0 | 0 |
| 2 | 0 | 0 | 0 |
| 3 | 0 | 0 | 0 |
| 4 | 0 | 0 | 0 |
| 5 | 0 | 0 | 0 |
| 6 | 0 | 0 | 0 |
| 7 | 0 | 16 | 0 |
| 8 | 0 | 76 | 0 |
| 9 | 0 | 173 | 0 |
| 10 | 0 | 141 | 0 |
| 11 | 0 | 164 | 0 |
| 12 | 0 | 142 | 0 |
| 13 | 0 | 111 | 0 |
| 14 | 0 | 67 | 0 |
| 15 | 0 | 40 | 0 |
| 16 | 0 | 28 | 0 |
| 17 | 0 | 16 | 0 |
| 18 | 0 | 7 | 0 |
| 19 | 0 | 19 | 0 |
| Total | 0 | 1000 | 0 |

0 bootstrap samples were unused because of a singular covariance matrix.

0 bootstrap samples were unused because a solution was not found.

1000 usable bootstrap samples were obtained.

##### Bootstrap Distributions (Default model)

##### ML discrepancy (implied vs sample) (Default model)

|  |  |  |
| --- | --- | --- |
|  |  | \|-------------------- |
|  | 437.858 | \|\* |
|  | 458.647 | \|\* |
|  | 479.436 | \|\*\*\* |
|  | 500.226 | \|\*\*\*\*\*\* |
|  | 521.015 | \|\*\*\*\*\*\*\*\*\*\*\*\* |
|  | 541.804 | \|\*\*\*\*\*\*\*\*\*\*\*\*\*\*\*\*\* |
|  | 562.594 | \|\*\*\*\*\*\*\*\*\*\*\*\*\*\*\*\*\*\*\* |
| N = 1000 | 583.383 | \|\*\*\*\*\*\*\*\*\*\*\*\*\*\*\*\*\*\*\* |
| Mean = 573.569 | 604.172 | \|\*\*\*\*\*\*\*\*\*\*\*\*\*\*\*\* |
| S. e. = 1.453 | 624.961 | \|\*\*\*\*\*\*\*\*\*\*\*\* |
|  | 645.751 | \|\*\*\*\*\* |
|  | 666.540 | \|\*\*\*\* |
|  | 687.329 | \|\*\* |
|  | 708.119 | \|\* |
|  | 728.908 | \|\* |
|  |  | \|-------------------- |

##### ML discrepancy (implied vs pop) (Default model)

|  |  |  |
| --- | --- | --- |
|  |  | \|-------------------- |
|  | 352.155 | \|\* |
|  | 360.199 | \|\*\* |
|  | 368.243 | \|\*\*\*\* |
|  | 376.287 | \|\*\*\*\*\*\*\*\*\*\*\* |
|  | 384.330 | \|\*\*\*\*\*\*\*\*\*\*\*\*\*\*\*\*\* |
|  | 392.374 | \|\*\*\*\*\*\*\*\*\*\*\*\*\*\*\*\*\*\*\* |
|  | 400.418 | \|\*\*\*\*\*\*\*\*\*\*\*\*\*\*\*\*\*\*\*\* |
| N = 1000 | 408.462 | \|\*\*\*\*\*\*\*\*\*\*\*\*\*\*\* |
| Mean = 399.727 | 416.506 | \|\*\*\*\*\*\*\*\*\*\* |
| S. e. = .601 | 424.549 | \|\*\*\*\*\*\*\*\* |
|  | 432.593 | \|\*\*\*\*\* |
|  | 440.637 | \|\*\*\* |
|  | 448.681 | \|\* |
|  | 456.724 | \|\* |
|  | 464.768 | \|\* |
|  |  | \|-------------------- |

##### K-L overoptimism (unstabilized) (Default model)

|  |  |  |
| --- | --- | --- |
|  |  | \|-------------------- |
|  | -143.668 | \|\* |
|  | -93.174 | \| |
|  | -42.680 | \|\*\* |
|  | 7.814 | \|\*\*\*\* |
|  | 58.308 | \|\*\*\*\*\*\*\*\*\* |
|  | 108.802 | \|\*\*\*\*\*\*\*\*\*\*\*\*\*\* |
|  | 159.296 | \|\*\*\*\*\*\*\*\*\*\*\*\*\*\*\*\*\*\* |
| N = 1000 | 209.789 | \|\*\*\*\*\*\*\*\*\*\*\*\*\*\*\*\*\*\*\* |
| Mean = 213.716 | 260.283 | \|\*\*\*\*\*\*\*\*\*\*\*\*\*\*\*\*\*\* |
| S. e. = 3.694 | 310.777 | \|\*\*\*\*\*\*\*\*\*\*\*\* |
|  | 361.271 | \|\*\*\*\*\*\*\*\* |
|  | 411.765 | \|\*\*\*\*\* |
|  | 462.259 | \|\*\*\* |
|  | 512.753 | \|\*\* |
|  | 563.247 | \|\* |
|  |  | \|-------------------- |

##### K-L overoptimism (stabilized) (Default model)

|  |  |  |
| --- | --- | --- |
|  |  | \|-------------------- |
|  | 91.941 | \|\* |
|  | 110.623 | \|\* |
|  | 129.304 | \|\* |
|  | 147.986 | \|\*\*\*\*\*\* |
|  | 166.668 | \|\*\*\*\*\*\*\*\*\*\* |
|  | 185.349 | \|\*\*\*\*\*\*\*\*\*\*\*\*\*\*\* |
|  | 204.031 | \|\*\*\*\*\*\*\*\*\*\*\*\*\*\*\*\*\*\*\* |
| N = 1000 | 222.713 | \|\*\*\*\*\*\*\*\*\*\*\*\*\*\*\*\*\* |
| Mean = 215.113 | 241.394 | \|\*\*\*\*\*\*\*\*\*\*\*\*\* |
| S. e. = 1.353 | 260.076 | \|\*\*\*\*\*\*\*\*\*\* |
|  | 278.757 | \|\*\*\*\*\*\* |
|  | 297.439 | \|\*\*\* |
|  | 316.121 | \|\*\* |
|  | 334.802 | \|\* |
|  | 353.484 | \|\* |
|  |  | \|-------------------- |

##### H1a vs H1 (H1a vs H1)

##### Notes for Model (H1a vs H1)

##### Computation of degrees of freedom (H1a vs H1)

|  |  |
| --- | --- |
| Number of distinct sample moments: | 342 |
| Number of distinct parameters to be estimated: | 81 |
| Degrees of freedom (342 - 81): | 261 |

##### Result (H1a vs H1)

Minimum was achieved

Chi-square = 298.753

Degrees of freedom = 261

Probability level = .054

##### Middle (Middle - H1a vs H1)

##### Estimates (Middle - H1a vs H1)

##### Scalar Estimates (Middle - H1a vs H1)

##### Maximum Likelihood Estimates

##### Regression Weights: (Middle - H1a vs H1)

|  |  |  | Estimate | S.E. | C.R. | P | Label |
| --- | --- | --- | --- | --- | --- | --- | --- |
| AI自我效能 | <--- | AI威胁感知 | .398 | .050 | 7.949 | \*\*\* | H1a |
| 防御性动机 | <--- | AI威胁感知 | .369 | .070 | 5.306 | \*\*\* | H2a |
| 防御性动机 | <--- | AI自我效能 | .154 | .088 | 1.753 | .080 | H5a |
| 使用行为 | <--- | 防御性动机 | .387 | .080 | 4.819 | \*\*\* | H4a |
| 使用行为 | <--- | AI自我效能 | .543 | .093 | 5.865 | \*\*\* | H3a |
| AA1 | <--- | AI威胁感知 | 1.000 |  |  |  |  |
| AA2 | <--- | AI威胁感知 | .930 | .062 | 14.900 | \*\*\* |  |
| AA3 | <--- | AI威胁感知 | .909 | .065 | 13.896 | \*\*\* |  |
| AA4 | <--- | AI威胁感知 | .917 | .064 | 14.329 | \*\*\* |  |
| AA5 | <--- | AI威胁感知 | .907 | .062 | 14.754 | \*\*\* |  |
| DM1 | <--- | 防御性动机 | 1.000 |  |  |  |  |
| DM2 | <--- | 防御性动机 | 1.068 | .103 | 10.414 | \*\*\* |  |
| DM3 | <--- | 防御性动机 | 1.082 | .103 | 10.553 | \*\*\* |  |
| DM5 | <--- | 防御性动机 | 1.013 | .102 | 9.938 | \*\*\* |  |
| SE1 | <--- | AI自我效能 | 1.000 |  |  |  |  |
| SE2 | <--- | AI自我效能 | 1.157 | .116 | 9.980 | \*\*\* |  |
| SE3 | <--- | AI自我效能 | 1.049 | .111 | 9.485 | \*\*\* |  |
| SE4 | <--- | AI自我效能 | 1.219 | .113 | 10.789 | \*\*\* |  |
| SE5 | <--- | AI自我效能 | 1.217 | .112 | 10.888 | \*\*\* |  |
| AB1 | <--- | 使用行为 | 1.000 |  |  |  |  |
| AB2 | <--- | 使用行为 | .923 | .079 | 11.648 | \*\*\* |  |
| AB3 | <--- | 使用行为 | 1.036 | .079 | 13.131 | \*\*\* |  |
| AB5 | <--- | 使用行为 | .962 | .080 | 12.044 | \*\*\* |  |

##### Standardized Regression Weights: (Middle - H1a vs H1)

|  |  |  | Estimate |
| --- | --- | --- | --- |
| AI自我效能 | <--- | AI威胁感知 | .530 |
| 防御性动机 | <--- | AI威胁感知 | .445 |
| 防御性动机 | <--- | AI自我效能 | .139 |
| 使用行为 | <--- | 防御性动机 | .345 |
| 使用行为 | <--- | AI自我效能 | .439 |
| AA1 | <--- | AI威胁感知 | .831 |
| AA2 | <--- | AI威胁感知 | .788 |
| AA3 | <--- | AI威胁感知 | .749 |
| AA4 | <--- | AI威胁感知 | .766 |
| AA5 | <--- | AI威胁感知 | .783 |
| DM1 | <--- | 防御性动机 | .687 |
| DM2 | <--- | 防御性动机 | .746 |
| DM3 | <--- | 防御性动机 | .760 |
| DM5 | <--- | 防御性动机 | .701 |
| SE1 | <--- | AI自我效能 | .642 |
| SE2 | <--- | AI自我效能 | .708 |
| SE3 | <--- | AI自我效能 | .665 |
| SE4 | <--- | AI自我效能 | .784 |
| SE5 | <--- | AI自我效能 | .794 |
| AB1 | <--- | 使用行为 | .771 |
| AB2 | <--- | 使用行为 | .716 |
| AB3 | <--- | 使用行为 | .810 |
| AB5 | <--- | 使用行为 | .740 |

##### Variances: (Middle - H1a vs H1)

|  |  |  | Estimate | S.E. | C.R. | P | Label |
| --- | --- | --- | --- | --- | --- | --- | --- |
| AI威胁感知 |  |  | 2.297 | .277 | 8.286 | \*\*\* |  |
| e21 |  |  | .933 | .167 | 5.568 | \*\*\* |  |
| e23 |  |  | 1.135 | .196 | 5.778 | \*\*\* |  |
| e22 |  |  | 1.142 | .176 | 6.493 | \*\*\* |  |
| e1 |  |  | 1.029 | .118 | 8.728 | \*\*\* |  |
| e2 |  |  | 1.212 | .127 | 9.554 | \*\*\* |  |
| e3 |  |  | 1.488 | .148 | 10.074 | \*\*\* |  |
| e4 |  |  | 1.363 | .138 | 9.870 | \*\*\* |  |
| e5 |  |  | 1.198 | .124 | 9.641 | \*\*\* |  |
| e6 |  |  | 1.775 | .184 | 9.666 | \*\*\* |  |
| e8 |  |  | 1.444 | .165 | 8.763 | \*\*\* |  |
| e9 |  |  | 1.356 | .160 | 8.479 | \*\*\* |  |
| e10 |  |  | 1.677 | .177 | 9.475 | \*\*\* |  |
| e11 |  |  | 1.847 | .175 | 10.542 | \*\*\* |  |
| e12 |  |  | 1.727 | .173 | 9.955 | \*\*\* |  |
| e13 |  |  | 1.801 | .174 | 10.360 | \*\*\* |  |
| e14 |  |  | 1.207 | .137 | 8.814 | \*\*\* |  |
| e15 |  |  | 1.122 | .130 | 8.601 | \*\*\* |  |
| e16 |  |  | 1.357 | .152 | 8.949 | \*\*\* |  |
| e17 |  |  | 1.607 | .164 | 9.782 | \*\*\* |  |
| e18 |  |  | 1.120 | .139 | 8.079 | \*\*\* |  |
| e20 |  |  | 1.521 | .161 | 9.470 | \*\*\* |  |

##### Squared Multiple Correlations: (Middle - H1a vs H1)

|  |  |  | Estimate |
| --- | --- | --- | --- |
| AI自我效能 |  |  | .281 |
| 防御性动机 |  |  | .283 |
| 使用行为 |  |  | .425 |
| AB5 |  |  | .547 |
| AB3 |  |  | .656 |
| AB2 |  |  | .513 |
| AB1 |  |  | .594 |
| SE5 |  |  | .631 |
| SE4 |  |  | .615 |
| SE3 |  |  | .442 |
| SE2 |  |  | .501 |
| SE1 |  |  | .413 |
| DM5 |  |  | .492 |
| DM3 |  |  | .578 |
| DM2 |  |  | .556 |
| DM1 |  |  | .471 |
| AA5 |  |  | .612 |
| AA4 |  |  | .586 |
| AA3 |  |  | .560 |
| AA2 |  |  | .621 |
| AA1 |  |  | .691 |

##### User-defined estimands: (Middle - H1a vs H1)

|  |  |  |  |
| --- | --- | --- | --- |
| diff1 |  |  | .000 |
| diff2 |  |  | -.029 |
| diff3 |  |  | .145 |
| diff4 |  |  | -.012 |
| diff5 |  |  | -.244 |

##### Modification Indices (Middle - H1a vs H1)

##### Covariances: (Middle - H1a vs H1)

|  |  |  | M.I. | Par Change |
| --- | --- | --- | --- | --- |
| e8 | <--> | e22 | 11.956 | .351 |
| e8 | <--> | e17 | 4.217 | .227 |
| e5 | <--> | e17 | 5.163 | -.223 |
| e4 | <--> | e15 | 4.226 | -.187 |
| e3 | <--> | e17 | 5.619 | .254 |
| e3 | <--> | e4 | 4.317 | -.203 |
| e2 | <--> | e22 | 8.883 | -.271 |
| e1 | <--> | e15 | 4.280 | .172 |
| e1 | <--> | e12 | 7.936 | -.275 |

##### Variances: (Middle - H1a vs H1)

|  |  |  | M.I. | Par Change |
| --- | --- | --- | --- | --- |

##### Regression Weights: (Middle - H1a vs H1)

|  |  |  | M.I. | Par Change |
| --- | --- | --- | --- | --- |
| SE5 | <--- | AA1 | 4.431 | .083 |
| DM2 | <--- | 使用行为 | 7.810 | .172 |
| DM2 | <--- | AB3 | 6.583 | .115 |
| DM2 | <--- | AB2 | 10.133 | .141 |
| DM2 | <--- | AB1 | 7.081 | .117 |
| AA4 | <--- | AB1 | 4.290 | .085 |
| AA2 | <--- | AB2 | 5.850 | -.096 |
| AA1 | <--- | SE2 | 5.346 | -.086 |

##### Bootstrap (Middle - H1a vs H1)

##### Bootstrap standard errors (Middle - H1a vs H1)

##### Scalar Estimates (Middle - H1a vs H1)

##### Regression Weights: (Middle - H1a vs H1)

| Parameter | | | SE | SE-SE | Mean | Bias | SE-Bias |
| --- | --- | --- | --- | --- | --- | --- | --- |
| AI自我效能 | <--- | AI威胁感知 | .050 | .001 | .399 | .000 | .002 |
| 防御性动机 | <--- | AI威胁感知 | .073 | .002 | .372 | .002 | .002 |
| 防御性动机 | <--- | AI自我效能 | .092 | .002 | .154 | .000 | .003 |
| 使用行为 | <--- | 防御性动机 | .080 | .002 | .386 | .000 | .003 |
| 使用行为 | <--- | AI自我效能 | .091 | .002 | .541 | -.002 | .003 |
| AA1 | <--- | AI威胁感知 | .000 | .000 | 1.000 | .000 | .000 |
| AA2 | <--- | AI威胁感知 | .049 | .001 | .933 | .003 | .002 |
| AA3 | <--- | AI威胁感知 | .053 | .001 | .908 | -.001 | .002 |
| AA4 | <--- | AI威胁感知 | .055 | .001 | .915 | -.002 | .002 |
| AA5 | <--- | AI威胁感知 | .056 | .001 | .910 | .002 | .002 |
| DM1 | <--- | 防御性动机 | .000 | .000 | 1.000 | .000 | .000 |
| DM2 | <--- | 防御性动机 | .100 | .002 | 1.072 | .004 | .003 |
| DM3 | <--- | 防御性动机 | .096 | .002 | 1.087 | .005 | .003 |
| DM5 | <--- | 防御性动机 | .103 | .002 | 1.018 | .005 | .003 |
| SE1 | <--- | AI自我效能 | .000 | .000 | 1.000 | .000 | .000 |
| SE2 | <--- | AI自我效能 | .105 | .002 | 1.155 | -.001 | .003 |
| SE3 | <--- | AI自我效能 | .098 | .002 | 1.051 | .002 | .003 |
| SE4 | <--- | AI自我效能 | .109 | .002 | 1.222 | .002 | .003 |
| SE5 | <--- | AI自我效能 | .109 | .002 | 1.215 | -.001 | .003 |
| AB1 | <--- | 使用行为 | .000 | .000 | 1.000 | .000 | .000 |
| AB2 | <--- | 使用行为 | .066 | .001 | .925 | .003 | .002 |
| AB3 | <--- | 使用行为 | .069 | .002 | 1.042 | .006 | .002 |
| AB5 | <--- | 使用行为 | .072 | .002 | .963 | .001 | .002 |

##### Standardized Regression Weights: (Middle - H1a vs H1)

| Parameter | | | SE | SE-SE | Mean | Bias | SE-Bias |
| --- | --- | --- | --- | --- | --- | --- | --- |
| AI自我效能 | <--- | AI威胁感知 | .052 | .001 | .527 | -.003 | .002 |
| 防御性动机 | <--- | AI威胁感知 | .078 | .002 | .447 | .002 | .002 |
| 防御性动机 | <--- | AI自我效能 | .081 | .002 | .139 | .000 | .003 |
| 使用行为 | <--- | 防御性动机 | .066 | .001 | .344 | -.001 | .002 |
| 使用行为 | <--- | AI自我效能 | .063 | .001 | .438 | -.001 | .002 |
| AA1 | <--- | AI威胁感知 | .024 | .001 | .830 | -.001 | .001 |
| AA2 | <--- | AI威胁感知 | .024 | .001 | .789 | .001 | .001 |
| AA3 | <--- | AI威胁感知 | .029 | .001 | .748 | -.001 | .001 |
| AA4 | <--- | AI威胁感知 | .029 | .001 | .764 | -.002 | .001 |
| AA5 | <--- | AI威胁感知 | .032 | .001 | .784 | .002 | .001 |
| DM1 | <--- | 防御性动机 | .043 | .001 | .685 | -.001 | .001 |
| DM2 | <--- | 防御性动机 | .035 | .001 | .744 | -.002 | .001 |
| DM3 | <--- | 防御性动机 | .034 | .001 | .759 | -.001 | .001 |
| DM5 | <--- | 防御性动机 | .038 | .001 | .700 | -.001 | .001 |
| SE1 | <--- | AI自我效能 | .041 | .001 | .644 | .002 | .001 |
| SE2 | <--- | AI自我效能 | .038 | .001 | .707 | -.001 | .001 |
| SE3 | <--- | AI自我效能 | .039 | .001 | .665 | .000 | .001 |
| SE4 | <--- | AI自我效能 | .027 | .001 | .785 | .000 | .001 |
| SE5 | <--- | AI自我效能 | .027 | .001 | .792 | -.002 | .001 |
| AB1 | <--- | 使用行为 | .032 | .001 | .769 | -.001 | .001 |
| AB2 | <--- | 使用行为 | .036 | .001 | .715 | -.001 | .001 |
| AB3 | <--- | 使用行为 | .027 | .001 | .810 | .001 | .001 |
| AB5 | <--- | 使用行为 | .034 | .001 | .738 | -.001 | .001 |

##### Variances: (Middle - H1a vs H1)

| Parameter | | | SE | SE-SE | Mean | Bias | SE-Bias |
| --- | --- | --- | --- | --- | --- | --- | --- |
| AI威胁感知 |  |  | .209 | .005 | 2.291 | -.006 | .007 |
| e21 |  |  | .166 | .004 | .944 | .012 | .005 |
| e23 |  |  | .191 | .004 | 1.119 | -.016 | .006 |
| e22 |  |  | .159 | .004 | 1.127 | -.015 | .005 |
| e1 |  |  | .123 | .003 | 1.026 | -.003 | .004 |
| e2 |  |  | .117 | .003 | 1.200 | -.012 | .004 |
| e3 |  |  | .129 | .003 | 1.474 | -.014 | .004 |
| e4 |  |  | .133 | .003 | 1.359 | -.004 | .004 |
| e5 |  |  | .146 | .003 | 1.175 | -.022 | .005 |
| e6 |  |  | .189 | .004 | 1.765 | -.010 | .006 |
| e8 |  |  | .158 | .004 | 1.437 | -.006 | .005 |
| e9 |  |  | .153 | .003 | 1.344 | -.012 | .005 |
| e10 |  |  | .165 | .004 | 1.667 | -.009 | .005 |
| e11 |  |  | .154 | .003 | 1.829 | -.018 | .005 |
| e12 |  |  | .198 | .004 | 1.716 | -.011 | .006 |
| e13 |  |  | .163 | .004 | 1.791 | -.010 | .005 |
| e14 |  |  | .128 | .003 | 1.197 | -.010 | .004 |
| e15 |  |  | .130 | .003 | 1.128 | .006 | .004 |
| e16 |  |  | .154 | .003 | 1.351 | -.006 | .005 |
| e17 |  |  | .167 | .004 | 1.599 | -.008 | .005 |
| e18 |  |  | .133 | .003 | 1.108 | -.012 | .004 |
| e20 |  |  | .152 | .003 | 1.508 | -.014 | .005 |

##### Squared Multiple Correlations: (Middle - H1a vs H1)

| Parameter | | | SE | SE-SE | Mean | Bias | SE-Bias |
| --- | --- | --- | --- | --- | --- | --- | --- |
| AI自我效能 |  |  | .055 | .001 | .281 | .000 | .002 |
| 防御性动机 |  |  | .057 | .001 | .293 | .010 | .002 |
| 使用行为 |  |  | .058 | .001 | .429 | .004 | .002 |
| AB5 |  |  | .050 | .001 | .546 | -.001 | .002 |
| AB3 |  |  | .044 | .001 | .657 | .002 | .001 |
| AB2 |  |  | .052 | .001 | .513 | .000 | .002 |
| AB1 |  |  | .049 | .001 | .593 | -.001 | .002 |
| SE5 |  |  | .043 | .001 | .628 | -.003 | .001 |
| SE4 |  |  | .043 | .001 | .616 | .001 | .001 |
| SE3 |  |  | .051 | .001 | .444 | .002 | .002 |
| SE2 |  |  | .053 | .001 | .502 | .001 | .002 |
| SE1 |  |  | .052 | .001 | .417 | .004 | .002 |
| DM5 |  |  | .053 | .001 | .492 | .000 | .002 |
| DM3 |  |  | .052 | .001 | .578 | .000 | .002 |
| DM2 |  |  | .052 | .001 | .555 | -.001 | .002 |
| DM1 |  |  | .058 | .001 | .472 | .000 | .002 |
| AA5 |  |  | .049 | .001 | .616 | .004 | .002 |
| AA4 |  |  | .044 | .001 | .584 | -.003 | .001 |
| AA3 |  |  | .044 | .001 | .560 | .000 | .001 |
| AA2 |  |  | .038 | .001 | .623 | .002 | .001 |
| AA1 |  |  | .039 | .001 | .690 | -.001 | .001 |

##### User-defined estimands: (Middle - H1a vs H1)

| Parameter | | | SE | SE-SE | Mean | Bias | SE-Bias |
| --- | --- | --- | --- | --- | --- | --- | --- |
| diff1 |  |  | .000 | .000 | .000 | .000 | .000 |
| diff2 |  |  | .085 | .002 | -.027 | .002 | .003 |
| diff3 |  |  | .112 | .003 | .142 | -.003 | .004 |
| diff4 |  |  | .094 | .002 | -.012 | .000 | .003 |
| diff5 |  |  | .109 | .002 | -.245 | -.001 | .003 |

##### Bootstrap Confidence (Middle - H1a vs H1)

##### Percentile method (Middle - H1a vs H1)

##### 95% confidence intervals (percentile method)

##### Scalar Estimates (Middle - H1a vs H1)

##### Regression Weights: (Middle - H1a vs H1)

| Parameter | | | Estimate | Lower | Upper | P |
| --- | --- | --- | --- | --- | --- | --- |
| AI自我效能 | <--- | AI威胁感知 | .398 | .301 | .504 | .002 |
| 防御性动机 | <--- | AI威胁感知 | .369 | .235 | .515 | .002 |
| 防御性动机 | <--- | AI自我效能 | .154 | -.022 | .337 | .082 |
| 使用行为 | <--- | 防御性动机 | .387 | .233 | .542 | .002 |
| 使用行为 | <--- | AI自我效能 | .543 | .373 | .724 | .002 |
| AA1 | <--- | AI威胁感知 | 1.000 | 1.000 | 1.000 | ... |
| AA2 | <--- | AI威胁感知 | .930 | .839 | 1.030 | .002 |
| AA3 | <--- | AI威胁感知 | .909 | .808 | 1.009 | .002 |
| AA4 | <--- | AI威胁感知 | .917 | .808 | 1.030 | .002 |
| AA5 | <--- | AI威胁感知 | .907 | .801 | 1.027 | .002 |
| DM1 | <--- | 防御性动机 | 1.000 | 1.000 | 1.000 | ... |
| DM2 | <--- | 防御性动机 | 1.068 | .897 | 1.302 | .002 |
| DM3 | <--- | 防御性动机 | 1.082 | .919 | 1.305 | .002 |
| DM5 | <--- | 防御性动机 | 1.013 | .834 | 1.252 | .002 |
| SE1 | <--- | AI自我效能 | 1.000 | 1.000 | 1.000 | ... |
| SE2 | <--- | AI自我效能 | 1.157 | .952 | 1.373 | .002 |
| SE3 | <--- | AI自我效能 | 1.049 | .873 | 1.257 | .002 |
| SE4 | <--- | AI自我效能 | 1.219 | 1.030 | 1.445 | .002 |
| SE5 | <--- | AI自我效能 | 1.217 | 1.011 | 1.447 | .002 |
| AB1 | <--- | 使用行为 | 1.000 | 1.000 | 1.000 | ... |
| AB2 | <--- | 使用行为 | .923 | .804 | 1.065 | .002 |
| AB3 | <--- | 使用行为 | 1.036 | .923 | 1.198 | .002 |
| AB5 | <--- | 使用行为 | .962 | .830 | 1.115 | .002 |

##### Standardized Regression Weights: (Middle - H1a vs H1)

| Parameter | | | Estimate | Lower | Upper | P |
| --- | --- | --- | --- | --- | --- | --- |
| AI自我效能 | <--- | AI威胁感知 | .530 | .421 | .623 | .002 |
| 防御性动机 | <--- | AI威胁感知 | .445 | .287 | .599 | .002 |
| 防御性动机 | <--- | AI自我效能 | .139 | -.020 | .297 | .082 |
| 使用行为 | <--- | 防御性动机 | .345 | .205 | .464 | .002 |
| 使用行为 | <--- | AI自我效能 | .439 | .307 | .558 | .002 |
| AA1 | <--- | AI威胁感知 | .831 | .779 | .876 | .002 |
| AA2 | <--- | AI威胁感知 | .788 | .737 | .835 | .002 |
| AA3 | <--- | AI威胁感知 | .749 | .685 | .802 | .002 |
| AA4 | <--- | AI威胁感知 | .766 | .703 | .818 | .002 |
| AA5 | <--- | AI威胁感知 | .783 | .721 | .843 | .002 |
| DM1 | <--- | 防御性动机 | .687 | .595 | .761 | .002 |
| DM2 | <--- | 防御性动机 | .746 | .674 | .805 | .002 |
| DM3 | <--- | 防御性动机 | .760 | .691 | .824 | .002 |
| DM5 | <--- | 防御性动机 | .701 | .620 | .770 | .002 |
| SE1 | <--- | AI自我效能 | .642 | .561 | .720 | .002 |
| SE2 | <--- | AI自我效能 | .708 | .628 | .782 | .002 |
| SE3 | <--- | AI自我效能 | .665 | .580 | .740 | .002 |
| SE4 | <--- | AI自我效能 | .784 | .726 | .838 | .002 |
| SE5 | <--- | AI自我效能 | .794 | .733 | .840 | .002 |
| AB1 | <--- | 使用行为 | .771 | .697 | .824 | .002 |
| AB2 | <--- | 使用行为 | .716 | .644 | .780 | .002 |
| AB3 | <--- | 使用行为 | .810 | .753 | .859 | .002 |
| AB5 | <--- | 使用行为 | .740 | .665 | .800 | .002 |

##### Variances: (Middle - H1a vs H1)

| Parameter | | | Estimate | Lower | Upper | P |
| --- | --- | --- | --- | --- | --- | --- |
| AI威胁感知 |  |  | 2.297 | 1.879 | 2.722 | .002 |
| e21 |  |  | .933 | .660 | 1.270 | .002 |
| e23 |  |  | 1.135 | .771 | 1.520 | .002 |
| e22 |  |  | 1.142 | .832 | 1.441 | .002 |
| e1 |  |  | 1.029 | .789 | 1.274 | .002 |
| e2 |  |  | 1.212 | .987 | 1.440 | .002 |
| e3 |  |  | 1.488 | 1.234 | 1.725 | .002 |
| e4 |  |  | 1.363 | 1.107 | 1.638 | .002 |
| e5 |  |  | 1.198 | .909 | 1.477 | .002 |
| e6 |  |  | 1.775 | 1.408 | 2.148 | .002 |
| e8 |  |  | 1.444 | 1.135 | 1.759 | .002 |
| e9 |  |  | 1.356 | 1.041 | 1.630 | .002 |
| e10 |  |  | 1.677 | 1.336 | 1.983 | .002 |
| e11 |  |  | 1.847 | 1.522 | 2.148 | .002 |
| e12 |  |  | 1.727 | 1.308 | 2.120 | .002 |
| e13 |  |  | 1.801 | 1.480 | 2.119 | .002 |
| e14 |  |  | 1.207 | .938 | 1.451 | .002 |
| e15 |  |  | 1.122 | .897 | 1.407 | .002 |
| e16 |  |  | 1.357 | 1.066 | 1.650 | .002 |
| e17 |  |  | 1.607 | 1.293 | 1.950 | .002 |
| e18 |  |  | 1.120 | .858 | 1.363 | .002 |
| e20 |  |  | 1.521 | 1.198 | 1.838 | .002 |

##### Squared Multiple Correlations: (Middle - H1a vs H1)

| Parameter | | | Estimate | Lower | Upper | P |
| --- | --- | --- | --- | --- | --- | --- |
| AI自我效能 |  |  | .281 | .178 | .388 | .002 |
| 防御性动机 |  |  | .283 | .180 | .409 | .002 |
| 使用行为 |  |  | .425 | .309 | .546 | .002 |
| AB5 |  |  | .547 | .442 | .640 | .002 |
| AB3 |  |  | .656 | .567 | .737 | .002 |
| AB2 |  |  | .513 | .414 | .609 | .002 |
| AB1 |  |  | .594 | .486 | .680 | .002 |
| SE5 |  |  | .631 | .538 | .705 | .002 |
| SE4 |  |  | .615 | .527 | .703 | .002 |
| SE3 |  |  | .442 | .336 | .547 | .002 |
| SE2 |  |  | .501 | .395 | .612 | .002 |
| SE1 |  |  | .413 | .314 | .519 | .002 |
| DM5 |  |  | .492 | .385 | .594 | .002 |
| DM3 |  |  | .578 | .478 | .679 | .002 |
| DM2 |  |  | .556 | .454 | .649 | .002 |
| DM1 |  |  | .471 | .354 | .579 | .002 |
| AA5 |  |  | .612 | .520 | .710 | .002 |
| AA4 |  |  | .586 | .494 | .670 | .002 |
| AA3 |  |  | .560 | .469 | .642 | .002 |
| AA2 |  |  | .621 | .543 | .698 | .002 |
| AA1 |  |  | .691 | .606 | .767 | .002 |

##### User-defined estimands: (Middle - H1a vs H1)

| Parameter | | | Estimate | Lower | Upper | P |
| --- | --- | --- | --- | --- | --- | --- |
| diff1 |  |  | .000 | .000 | .000 | ... |
| diff2 |  |  | -.029 | -.201 | .149 | .732 |
| diff3 |  |  | .145 | -.068 | .382 | .201 |
| diff4 |  |  | -.012 | -.200 | .180 | .886 |
| diff5 |  |  | -.244 | -.456 | -.015 | .032 |

##### Bias-corrected percentile method (Middle - H1a vs H1)

##### 95% confidence intervals (bias-corrected percentile method)

##### Scalar Estimates (Middle - H1a vs H1)

##### Regression Weights: (Middle - H1a vs H1)

| Parameter | | | Estimate | Lower | Upper | P |
| --- | --- | --- | --- | --- | --- | --- |
| AI自我效能 | <--- | AI威胁感知 | .398 | .303 | .505 | .002 |
| 防御性动机 | <--- | AI威胁感知 | .369 | .233 | .511 | .002 |
| 防御性动机 | <--- | AI自我效能 | .154 | -.029 | .337 | .084 |
| 使用行为 | <--- | 防御性动机 | .387 | .228 | .541 | .002 |
| 使用行为 | <--- | AI自我效能 | .543 | .386 | .740 | .001 |
| AA1 | <--- | AI威胁感知 | 1.000 | 1.000 | 1.000 | ... |
| AA2 | <--- | AI威胁感知 | .930 | .830 | 1.025 | .003 |
| AA3 | <--- | AI威胁感知 | .909 | .808 | 1.016 | .002 |
| AA4 | <--- | AI威胁感知 | .917 | .811 | 1.037 | .001 |
| AA5 | <--- | AI威胁感知 | .907 | .798 | 1.021 | .003 |
| DM1 | <--- | 防御性动机 | 1.000 | 1.000 | 1.000 | ... |
| DM2 | <--- | 防御性动机 | 1.068 | .893 | 1.297 | .002 |
| DM3 | <--- | 防御性动机 | 1.082 | .917 | 1.299 | .002 |
| DM5 | <--- | 防御性动机 | 1.013 | .835 | 1.254 | .002 |
| SE1 | <--- | AI自我效能 | 1.000 | 1.000 | 1.000 | ... |
| SE2 | <--- | AI自我效能 | 1.157 | .985 | 1.390 | .001 |
| SE3 | <--- | AI自我效能 | 1.049 | .874 | 1.262 | .002 |
| SE4 | <--- | AI自我效能 | 1.219 | 1.038 | 1.463 | .001 |
| SE5 | <--- | AI自我效能 | 1.217 | 1.029 | 1.454 | .001 |
| AB1 | <--- | 使用行为 | 1.000 | 1.000 | 1.000 | ... |
| AB2 | <--- | 使用行为 | .923 | .801 | 1.059 | .002 |
| AB3 | <--- | 使用行为 | 1.036 | .924 | 1.203 | .002 |
| AB5 | <--- | 使用行为 | .962 | .835 | 1.134 | .001 |

##### Standardized Regression Weights: (Middle - H1a vs H1)

| Parameter | | | Estimate | Lower | Upper | P |
| --- | --- | --- | --- | --- | --- | --- |
| AI自我效能 | <--- | AI威胁感知 | .530 | .422 | .624 | .002 |
| 防御性动机 | <--- | AI威胁感知 | .445 | .273 | .582 | .004 |
| 防御性动机 | <--- | AI自我效能 | .139 | -.035 | .291 | .099 |
| 使用行为 | <--- | 防御性动机 | .345 | .204 | .462 | .002 |
| 使用行为 | <--- | AI自我效能 | .439 | .297 | .553 | .003 |
| AA1 | <--- | AI威胁感知 | .831 | .778 | .875 | .002 |
| AA2 | <--- | AI威胁感知 | .788 | .733 | .831 | .003 |
| AA3 | <--- | AI威胁感知 | .749 | .687 | .804 | .002 |
| AA4 | <--- | AI威胁感知 | .766 | .708 | .820 | .001 |
| AA5 | <--- | AI威胁感知 | .783 | .716 | .838 | .003 |
| DM1 | <--- | 防御性动机 | .687 | .593 | .760 | .002 |
| DM2 | <--- | 防御性动机 | .746 | .674 | .806 | .002 |
| DM3 | <--- | 防御性动机 | .760 | .691 | .823 | .002 |
| DM5 | <--- | 防御性动机 | .701 | .625 | .774 | .002 |
| SE1 | <--- | AI自我效能 | .642 | .555 | .715 | .003 |
| SE2 | <--- | AI自我效能 | .708 | .627 | .782 | .002 |
| SE3 | <--- | AI自我效能 | .665 | .576 | .735 | .003 |
| SE4 | <--- | AI自我效能 | .784 | .725 | .834 | .002 |
| SE5 | <--- | AI自我效能 | .794 | .734 | .840 | .002 |
| AB1 | <--- | 使用行为 | .771 | .698 | .824 | .002 |
| AB2 | <--- | 使用行为 | .716 | .644 | .781 | .002 |
| AB3 | <--- | 使用行为 | .810 | .747 | .856 | .003 |
| AB5 | <--- | 使用行为 | .740 | .662 | .800 | .002 |

##### Variances: (Middle - H1a vs H1)

| Parameter | | | Estimate | Lower | Upper | P |
| --- | --- | --- | --- | --- | --- | --- |
| AI威胁感知 |  |  | 2.297 | 1.894 | 2.734 | .002 |
| e21 |  |  | .933 | .660 | 1.270 | .002 |
| e23 |  |  | 1.135 | .810 | 1.571 | .001 |
| e22 |  |  | 1.142 | .864 | 1.467 | .001 |
| e1 |  |  | 1.029 | .796 | 1.285 | .002 |
| e2 |  |  | 1.212 | 1.018 | 1.514 | .001 |
| e3 |  |  | 1.488 | 1.257 | 1.765 | .001 |
| e4 |  |  | 1.363 | 1.132 | 1.659 | .001 |
| e5 |  |  | 1.198 | .947 | 1.528 | .001 |
| e6 |  |  | 1.775 | 1.432 | 2.178 | .001 |
| e8 |  |  | 1.444 | 1.167 | 1.782 | .001 |
| e9 |  |  | 1.356 | 1.056 | 1.643 | .001 |
| e10 |  |  | 1.677 | 1.337 | 1.984 | .002 |
| e11 |  |  | 1.847 | 1.564 | 2.169 | .001 |
| e12 |  |  | 1.727 | 1.353 | 2.161 | .001 |
| e13 |  |  | 1.801 | 1.493 | 2.137 | .001 |
| e14 |  |  | 1.207 | .958 | 1.477 | .001 |
| e15 |  |  | 1.122 | .905 | 1.411 | .002 |
| e16 |  |  | 1.357 | 1.072 | 1.674 | .002 |
| e17 |  |  | 1.607 | 1.303 | 1.967 | .001 |
| e18 |  |  | 1.120 | .890 | 1.388 | .001 |
| e20 |  |  | 1.521 | 1.257 | 1.891 | .001 |

##### Squared Multiple Correlations: (Middle - H1a vs H1)

| Parameter | | | Estimate | Lower | Upper | P |
| --- | --- | --- | --- | --- | --- | --- |
| AI自我效能 |  |  | .281 | .178 | .389 | .002 |
| 防御性动机 |  |  | .283 | .160 | .387 | .006 |
| 使用行为 |  |  | .425 | .305 | .541 | .003 |
| AB5 |  |  | .547 | .438 | .639 | .002 |
| AB3 |  |  | .656 | .559 | .732 | .003 |
| AB2 |  |  | .513 | .415 | .609 | .002 |
| AB1 |  |  | .594 | .487 | .680 | .002 |
| SE5 |  |  | .631 | .538 | .706 | .002 |
| SE4 |  |  | .615 | .525 | .696 | .002 |
| SE3 |  |  | .442 | .332 | .540 | .003 |
| SE2 |  |  | .501 | .393 | .612 | .002 |
| SE1 |  |  | .413 | .308 | .511 | .003 |
| DM5 |  |  | .492 | .391 | .599 | .002 |
| DM3 |  |  | .578 | .478 | .678 | .002 |
| DM2 |  |  | .556 | .455 | .649 | .002 |
| DM1 |  |  | .471 | .351 | .578 | .002 |
| AA5 |  |  | .612 | .512 | .702 | .003 |
| AA4 |  |  | .586 | .501 | .672 | .001 |
| AA3 |  |  | .560 | .472 | .646 | .002 |
| AA2 |  |  | .621 | .538 | .690 | .003 |
| AA1 |  |  | .691 | .605 | .765 | .002 |

##### User-defined estimands: (Middle - H1a vs H1)

| Parameter | | | Estimate | Lower | Upper | P |
| --- | --- | --- | --- | --- | --- | --- |
| diff1 |  |  | .000 | .000 | .000 | ... |
| diff2 |  |  | -.029 | -.202 | .149 | .728 |
| diff3 |  |  | .145 | -.061 | .402 | .176 |
| diff4 |  |  | -.012 | -.200 | .171 | .855 |
| diff5 |  |  | -.244 | -.446 | -.011 | .035 |

##### others (others - H1a vs H1)

##### Estimates (others - H1a vs H1)

##### Scalar Estimates (others - H1a vs H1)

##### Maximum Likelihood Estimates

##### Regression Weights: (others - H1a vs H1)

|  |  |  | Estimate | S.E. | C.R. | P | Label |
| --- | --- | --- | --- | --- | --- | --- | --- |
| AI自我效能 | <--- | AI威胁感知 | .398 | .050 | 7.949 | \*\*\* | H1a |
| 防御性动机 | <--- | AI威胁感知 | .419 | .139 | 3.015 | .003 | H2 |
| 防御性动机 | <--- | AI自我效能 | -.152 | .145 | -1.045 | .296 | H5 |
| 使用行为 | <--- | 防御性动机 | .396 | .108 | 3.654 | \*\*\* | H4 |
| 使用行为 | <--- | AI自我效能 | .275 | .114 | 2.423 | .015 | H3 |
| AA1 | <--- | AI威胁感知 | 1.000 |  |  |  |  |
| AA2 | <--- | AI威胁感知 | 1.032 | .129 | 8.028 | \*\*\* |  |
| AA3 | <--- | AI威胁感知 | .973 | .123 | 7.898 | \*\*\* |  |
| AA4 | <--- | AI威胁感知 | .951 | .128 | 7.415 | \*\*\* |  |
| AA5 | <--- | AI威胁感知 | 1.076 | .131 | 8.230 | \*\*\* |  |
| DM1 | <--- | 防御性动机 | 1.000 |  |  |  |  |
| DM2 | <--- | 防御性动机 | .896 | .133 | 6.755 | \*\*\* |  |
| DM3 | <--- | 防御性动机 | .927 | .133 | 6.963 | \*\*\* |  |
| DM5 | <--- | 防御性动机 | .919 | .133 | 6.918 | \*\*\* |  |
| SE1 | <--- | AI自我效能 | 1.000 |  |  |  |  |
| SE2 | <--- | AI自我效能 | 1.123 | .170 | 6.593 | \*\*\* |  |
| SE3 | <--- | AI自我效能 | 1.216 | .172 | 7.079 | \*\*\* |  |
| SE4 | <--- | AI自我效能 | 1.416 | .188 | 7.549 | \*\*\* |  |
| SE5 | <--- | AI自我效能 | 1.215 | .171 | 7.099 | \*\*\* |  |
| AB1 | <--- | 使用行为 | 1.000 |  |  |  |  |
| AB2 | <--- | 使用行为 | 1.157 | .158 | 7.318 | \*\*\* |  |
| AB3 | <--- | 使用行为 | 1.125 | .158 | 7.133 | \*\*\* |  |
| AB5 | <--- | 使用行为 | .931 | .152 | 6.128 | \*\*\* |  |

##### Standardized Regression Weights: (others - H1a vs H1)

|  |  |  | Estimate |
| --- | --- | --- | --- |
| AI自我效能 | <--- | AI威胁感知 | .440 |
| 防御性动机 | <--- | AI威胁感知 | .401 |
| 防御性动机 | <--- | AI自我效能 | -.132 |
| 使用行为 | <--- | 防御性动机 | .437 |
| 使用行为 | <--- | AI自我效能 | .264 |
| AA1 | <--- | AI威胁感知 | .772 |
| AA2 | <--- | AI威胁感知 | .767 |
| AA3 | <--- | AI威胁感知 | .756 |
| AA4 | <--- | AI威胁感知 | .716 |
| AA5 | <--- | AI威胁感知 | .785 |
| DM1 | <--- | 防御性动机 | .767 |
| DM2 | <--- | 防御性动机 | .712 |
| DM3 | <--- | 防御性动机 | .737 |
| DM5 | <--- | 防御性动机 | .731 |
| SE1 | <--- | AI自我效能 | .641 |
| SE2 | <--- | AI自我效能 | .728 |
| SE3 | <--- | AI自我效能 | .791 |
| SE4 | <--- | AI自我效能 | .859 |
| SE5 | <--- | AI自我效能 | .794 |
| AB1 | <--- | 使用行为 | .723 |
| AB2 | <--- | 使用行为 | .813 |
| AB3 | <--- | 使用行为 | .783 |
| AB5 | <--- | 使用行为 | .660 |

##### Variances: (others - H1a vs H1)

|  |  |  | Estimate | S.E. | C.R. | P | Label |
| --- | --- | --- | --- | --- | --- | --- | --- |
| AI威胁感知 |  |  | 1.820 | .397 | 4.582 | \*\*\* |  |
| e21 |  |  | 1.205 | .336 | 3.587 | \*\*\* |  |
| e23 |  |  | 1.728 | .416 | 4.150 | \*\*\* |  |
| e22 |  |  | 1.190 | .314 | 3.791 | \*\*\* |  |
| e1 |  |  | 1.230 | .214 | 5.734 | \*\*\* |  |
| e2 |  |  | 1.354 | .235 | 5.766 | \*\*\* |  |
| e3 |  |  | 1.287 | .220 | 5.864 | \*\*\* |  |
| e4 |  |  | 1.567 | .254 | 6.161 | \*\*\* |  |
| e5 |  |  | 1.316 | .235 | 5.591 | \*\*\* |  |
| e6 |  |  | 1.389 | .271 | 5.127 | \*\*\* |  |
| e8 |  |  | 1.548 | .271 | 5.724 | \*\*\* |  |
| e9 |  |  | 1.441 | .262 | 5.490 | \*\*\* |  |
| e10 |  |  | 1.462 | .264 | 5.547 | \*\*\* |  |
| e11 |  |  | 2.141 | .323 | 6.621 | \*\*\* |  |
| e12 |  |  | 1.671 | .269 | 6.221 | \*\*\* |  |
| e13 |  |  | 1.319 | .231 | 5.712 | \*\*\* |  |
| e14 |  |  | 1.061 | .227 | 4.679 | \*\*\* |  |
| e15 |  |  | 1.293 | .227 | 5.683 | \*\*\* |  |
| e16 |  |  | 1.491 | .258 | 5.779 | \*\*\* |  |
| e17 |  |  | 1.119 | .242 | 4.618 | \*\*\* |  |
| e18 |  |  | 1.305 | .256 | 5.099 | \*\*\* |  |
| e20 |  |  | 1.833 | .295 | 6.219 | \*\*\* |  |

##### Squared Multiple Correlations: (others - H1a vs H1)

|  |  |  | Estimate |
| --- | --- | --- | --- |
| AI自我效能 |  |  | .193 |
| 防御性动机 |  |  | .131 |
| 使用行为 |  |  | .271 |
| AB5 |  |  | .436 |
| AB3 |  |  | .613 |
| AB2 |  |  | .661 |
| AB1 |  |  | .522 |
| SE5 |  |  | .630 |
| SE4 |  |  | .738 |
| SE3 |  |  | .626 |
| SE2 |  |  | .530 |
| SE1 |  |  | .411 |
| DM5 |  |  | .535 |
| DM3 |  |  | .543 |
| DM2 |  |  | .508 |
| DM1 |  |  | .589 |
| AA5 |  |  | .616 |
| AA4 |  |  | .512 |
| AA3 |  |  | .572 |
| AA2 |  |  | .589 |
| AA1 |  |  | .597 |

##### User-defined estimands: (others - H1a vs H1)

|  |  |  |  |
| --- | --- | --- | --- |
| diff1 |  |  | .000 |
| diff2 |  |  | -.029 |
| diff3 |  |  | .145 |
| diff4 |  |  | -.012 |
| diff5 |  |  | -.244 |

##### Modification Indices (others - H1a vs H1)

##### Covariances: (others - H1a vs H1)

|  |  |  | M.I. | Par Change |
| --- | --- | --- | --- | --- |
| e21 | <--> | AI威胁感知 | 5.250 | -.384 |
| e20 | <--> | e21 | 4.699 | .364 |
| e17 | <--> | e23 | 5.225 | .413 |
| e13 | <--> | e20 | 4.288 | .370 |
| e13 | <--> | e15 | 4.029 | .306 |
| e11 | <--> | e20 | 8.854 | -.637 |
| e10 | <--> | e20 | 8.010 | -.535 |
| e9 | <--> | e13 | 5.327 | -.383 |
| e5 | <--> | e16 | 4.143 | -.340 |
| e5 | <--> | e8 | 5.445 | .398 |

##### Variances: (others - H1a vs H1)

|  |  |  | M.I. | Par Change |
| --- | --- | --- | --- | --- |

##### Regression Weights: (others - H1a vs H1)

|  |  |  | M.I. | Par Change |
| --- | --- | --- | --- | --- |
| AI自我效能 | <--- | AI威胁感知 | 5.250 | -.211 |
| AB5 | <--- | AI自我效能 | 4.996 | .273 |
| AB5 | <--- | SE4 | 5.281 | .161 |
| AB5 | <--- | SE3 | 7.939 | .211 |
| AB5 | <--- | SE2 | 4.118 | .152 |
| AB5 | <--- | DM5 | 5.577 | -.188 |
| AB2 | <--- | 防御性动机 | 4.114 | .193 |
| AB2 | <--- | DM5 | 4.594 | .149 |
| AB1 | <--- | AA5 | 5.482 | -.165 |
| SE4 | <--- | AA3 | 4.464 | .147 |
| SE3 | <--- | AA2 | 5.111 | -.155 |
| SE1 | <--- | AB5 | 6.194 | -.206 |
| SE1 | <--- | AA1 | 4.139 | -.173 |
| DM5 | <--- | AB5 | 4.498 | -.154 |
| DM3 | <--- | SE4 | 4.648 | .140 |
| AA5 | <--- | AB1 | 4.101 | -.143 |

##### Bootstrap (others - H1a vs H1)

##### Bootstrap standard errors (others - H1a vs H1)

##### Scalar Estimates (others - H1a vs H1)

##### Regression Weights: (others - H1a vs H1)

| Parameter | | | SE | SE-SE | Mean | Bias | SE-Bias |
| --- | --- | --- | --- | --- | --- | --- | --- |
| AI自我效能 | <--- | AI威胁感知 | .050 | .001 | .399 | .000 | .002 |
| 防御性动机 | <--- | AI威胁感知 | .126 | .003 | .413 | -.006 | .004 |
| 防御性动机 | <--- | AI自我效能 | .157 | .004 | -.155 | -.003 | .005 |
| 使用行为 | <--- | 防御性动机 | .099 | .002 | .393 | -.003 | .003 |
| 使用行为 | <--- | AI自我效能 | .140 | .003 | .284 | .008 | .004 |
| AA1 | <--- | AI威胁感知 | .000 | .000 | 1.000 | .000 | .000 |
| AA2 | <--- | AI威胁感知 | .121 | .003 | 1.032 | .000 | .004 |
| AA3 | <--- | AI威胁感知 | .090 | .002 | .977 | .004 | .003 |
| AA4 | <--- | AI威胁感知 | .133 | .003 | .948 | -.003 | .004 |
| AA5 | <--- | AI威胁感知 | .136 | .003 | 1.084 | .008 | .004 |
| DM1 | <--- | 防御性动机 | .000 | .000 | 1.000 | .000 | .000 |
| DM2 | <--- | 防御性动机 | .135 | .003 | .902 | .006 | .004 |
| DM3 | <--- | 防御性动机 | .145 | .003 | .929 | .002 | .005 |
| DM5 | <--- | 防御性动机 | .108 | .002 | .924 | .005 | .003 |
| SE1 | <--- | AI自我效能 | .000 | .000 | 1.000 | .000 | .000 |
| SE2 | <--- | AI自我效能 | .164 | .004 | 1.132 | .010 | .005 |
| SE3 | <--- | AI自我效能 | .141 | .003 | 1.231 | .015 | .004 |
| SE4 | <--- | AI自我效能 | .178 | .004 | 1.433 | .017 | .006 |
| SE5 | <--- | AI自我效能 | .163 | .004 | 1.233 | .018 | .005 |
| AB1 | <--- | 使用行为 | .000 | .000 | 1.000 | .000 | .000 |
| AB2 | <--- | 使用行为 | .161 | .004 | 1.172 | .015 | .005 |
| AB3 | <--- | 使用行为 | .159 | .004 | 1.137 | .012 | .005 |
| AB5 | <--- | 使用行为 | .146 | .003 | .942 | .011 | .005 |

##### Standardized Regression Weights: (others - H1a vs H1)

| Parameter | | | SE | SE-SE | Mean | Bias | SE-Bias |
| --- | --- | --- | --- | --- | --- | --- | --- |
| AI自我效能 | <--- | AI威胁感知 | .052 | .001 | .441 | .002 | .002 |
| 防御性动机 | <--- | AI威胁感知 | .108 | .002 | .391 | -.010 | .003 |
| 防御性动机 | <--- | AI自我效能 | .130 | .003 | -.131 | .001 | .004 |
| 使用行为 | <--- | 防御性动机 | .098 | .002 | .433 | -.004 | .003 |
| 使用行为 | <--- | AI自我效能 | .118 | .003 | .265 | .001 | .004 |
| AA1 | <--- | AI威胁感知 | .058 | .001 | .771 | -.002 | .002 |
| AA2 | <--- | AI威胁感知 | .059 | .001 | .762 | -.005 | .002 |
| AA3 | <--- | AI威胁感知 | .053 | .001 | .757 | .000 | .002 |
| AA4 | <--- | AI威胁感知 | .067 | .002 | .707 | -.009 | .002 |
| AA5 | <--- | AI威胁感知 | .049 | .001 | .783 | -.001 | .002 |
| DM1 | <--- | 防御性动机 | .058 | .001 | .768 | .001 | .002 |
| DM2 | <--- | 防御性动机 | .068 | .002 | .711 | -.001 | .002 |
| DM3 | <--- | 防御性动机 | .061 | .001 | .732 | -.005 | .002 |
| DM5 | <--- | 防御性动机 | .060 | .001 | .731 | .000 | .002 |
| SE1 | <--- | AI自我效能 | .052 | .001 | .639 | -.002 | .002 |
| SE2 | <--- | AI自我效能 | .060 | .001 | .723 | -.004 | .002 |
| SE3 | <--- | AI自我效能 | .043 | .001 | .791 | .000 | .001 |
| SE4 | <--- | AI自我效能 | .043 | .001 | .857 | -.002 | .001 |
| SE5 | <--- | AI自我效能 | .058 | .001 | .793 | -.001 | .002 |
| AB1 | <--- | 使用行为 | .061 | .001 | .720 | -.003 | .002 |
| AB2 | <--- | 使用行为 | .059 | .001 | .813 | .000 | .002 |
| AB3 | <--- | 使用行为 | .059 | .001 | .783 | .000 | .002 |
| AB5 | <--- | 使用行为 | .069 | .002 | .659 | -.001 | .002 |

##### Variances: (others - H1a vs H1)

| Parameter | | | SE | SE-SE | Mean | Bias | SE-Bias |
| --- | --- | --- | --- | --- | --- | --- | --- |
| AI威胁感知 |  |  | .389 | .009 | 1.819 | .000 | .012 |
| e21 |  |  | .274 | .006 | 1.192 | -.013 | .009 |
| e23 |  |  | .392 | .009 | 1.705 | -.022 | .012 |
| e22 |  |  | .315 | .007 | 1.166 | -.024 | .010 |
| e1 |  |  | .231 | .005 | 1.204 | -.025 | .007 |
| e2 |  |  | .260 | .006 | 1.338 | -.015 | .008 |
| e3 |  |  | .235 | .005 | 1.252 | -.035 | .007 |
| e4 |  |  | .272 | .006 | 1.561 | -.006 | .009 |
| e5 |  |  | .239 | .005 | 1.290 | -.026 | .008 |
| e6 |  |  | .273 | .006 | 1.355 | -.034 | .009 |
| e8 |  |  | .314 | .007 | 1.528 | -.021 | .010 |
| e9 |  |  | .236 | .005 | 1.422 | -.019 | .007 |
| e10 |  |  | .261 | .006 | 1.438 | -.024 | .008 |
| e11 |  |  | .287 | .006 | 2.104 | -.037 | .009 |
| e12 |  |  | .250 | .006 | 1.648 | -.023 | .008 |
| e13 |  |  | .212 | .005 | 1.291 | -.028 | .007 |
| e14 |  |  | .259 | .006 | 1.045 | -.017 | .008 |
| e15 |  |  | .253 | .006 | 1.263 | -.029 | .008 |
| e16 |  |  | .232 | .005 | 1.472 | -.019 | .007 |
| e17 |  |  | .309 | .007 | 1.096 | -.023 | .010 |
| e18 |  |  | .297 | .007 | 1.271 | -.034 | .009 |
| e20 |  |  | .289 | .006 | 1.806 | -.027 | .009 |

##### Squared Multiple Correlations: (others - H1a vs H1)

| Parameter | | | SE | SE-SE | Mean | Bias | SE-Bias |
| --- | --- | --- | --- | --- | --- | --- | --- |
| AI自我效能 |  |  | .046 | .001 | .197 | .004 | .001 |
| 防御性动机 |  |  | .071 | .002 | .150 | .019 | .002 |
| 使用行为 |  |  | .095 | .002 | .288 | .018 | .003 |
| AB5 |  |  | .089 | .002 | .439 | .003 | .003 |
| AB3 |  |  | .091 | .002 | .617 | .004 | .003 |
| AB2 |  |  | .095 | .002 | .664 | .003 | .003 |
| AB1 |  |  | .086 | .002 | .522 | .000 | .003 |
| SE5 |  |  | .089 | .002 | .632 | .001 | .003 |
| SE4 |  |  | .073 | .002 | .737 | -.002 | .002 |
| SE3 |  |  | .067 | .002 | .627 | .001 | .002 |
| SE2 |  |  | .085 | .002 | .527 | -.003 | .003 |
| SE1 |  |  | .066 | .001 | .412 | .001 | .002 |
| DM5 |  |  | .086 | .002 | .538 | .003 | .003 |
| DM3 |  |  | .089 | .002 | .539 | -.003 | .003 |
| DM2 |  |  | .096 | .002 | .510 | .003 | .003 |
| DM1 |  |  | .089 | .002 | .593 | .004 | .003 |
| AA5 |  |  | .076 | .002 | .616 | .000 | .002 |
| AA4 |  |  | .094 | .002 | .504 | -.008 | .003 |
| AA3 |  |  | .080 | .002 | .576 | .004 | .003 |
| AA2 |  |  | .088 | .002 | .584 | -.004 | .003 |
| AA1 |  |  | .088 | .002 | .597 | .001 | .003 |

##### User-defined estimands: (others - H1a vs H1)

| Parameter | | | SE | SE-SE | Mean | Bias | SE-Bias |
| --- | --- | --- | --- | --- | --- | --- | --- |
| diff1 |  |  | .000 | .000 | .000 | .000 | .000 |
| diff2 |  |  | .085 | .002 | -.027 | .002 | .003 |
| diff3 |  |  | .112 | .003 | .142 | -.003 | .004 |
| diff4 |  |  | .094 | .002 | -.012 | .000 | .003 |
| diff5 |  |  | .109 | .002 | -.245 | -.001 | .003 |

##### Bootstrap Confidence (others - H1a vs H1)

##### Percentile method (others - H1a vs H1)

##### 95% confidence intervals (percentile method)

##### Scalar Estimates (others - H1a vs H1)

##### Regression Weights: (others - H1a vs H1)

| Parameter | | | Estimate | Lower | Upper | P |
| --- | --- | --- | --- | --- | --- | --- |
| AI自我效能 | <--- | AI威胁感知 | .398 | .301 | .504 | .002 |
| 防御性动机 | <--- | AI威胁感知 | .419 | .167 | .667 | .002 |
| 防御性动机 | <--- | AI自我效能 | -.152 | -.477 | .158 | .317 |
| 使用行为 | <--- | 防御性动机 | .396 | .202 | .588 | .002 |
| 使用行为 | <--- | AI自我效能 | .275 | .029 | .574 | .034 |
| AA1 | <--- | AI威胁感知 | 1.000 | 1.000 | 1.000 | ... |
| AA2 | <--- | AI威胁感知 | 1.032 | .814 | 1.289 | .002 |
| AA3 | <--- | AI威胁感知 | .973 | .815 | 1.158 | .002 |
| AA4 | <--- | AI威胁感知 | .951 | .715 | 1.234 | .002 |
| AA5 | <--- | AI威胁感知 | 1.076 | .847 | 1.372 | .002 |
| DM1 | <--- | 防御性动机 | 1.000 | 1.000 | 1.000 | ... |
| DM2 | <--- | 防御性动机 | .896 | .676 | 1.193 | .002 |
| DM3 | <--- | 防御性动机 | .927 | .661 | 1.222 | .002 |
| DM5 | <--- | 防御性动机 | .919 | .729 | 1.139 | .002 |
| SE1 | <--- | AI自我效能 | 1.000 | 1.000 | 1.000 | ... |
| SE2 | <--- | AI自我效能 | 1.123 | .824 | 1.499 | .002 |
| SE3 | <--- | AI自我效能 | 1.216 | .973 | 1.536 | .002 |
| SE4 | <--- | AI自我效能 | 1.416 | 1.125 | 1.836 | .002 |
| SE5 | <--- | AI自我效能 | 1.215 | .916 | 1.560 | .002 |
| AB1 | <--- | 使用行为 | 1.000 | 1.000 | 1.000 | ... |
| AB2 | <--- | 使用行为 | 1.157 | .915 | 1.522 | .002 |
| AB3 | <--- | 使用行为 | 1.125 | .880 | 1.494 | .002 |
| AB5 | <--- | 使用行为 | .931 | .684 | 1.238 | .002 |

##### Standardized Regression Weights: (others - H1a vs H1)

| Parameter | | | Estimate | Lower | Upper | P |
| --- | --- | --- | --- | --- | --- | --- |
| AI自我效能 | <--- | AI威胁感知 | .440 | .339 | .547 | .002 |
| 防御性动机 | <--- | AI威胁感知 | .401 | .168 | .597 | .002 |
| 防御性动机 | <--- | AI自我效能 | -.132 | -.377 | .127 | .317 |
| 使用行为 | <--- | 防御性动机 | .437 | .230 | .599 | .002 |
| 使用行为 | <--- | AI自我效能 | .264 | .024 | .489 | .034 |
| AA1 | <--- | AI威胁感知 | .772 | .635 | .874 | .002 |
| AA2 | <--- | AI威胁感知 | .767 | .627 | .863 | .002 |
| AA3 | <--- | AI威胁感知 | .756 | .648 | .849 | .002 |
| AA4 | <--- | AI威胁感知 | .716 | .558 | .828 | .002 |
| AA5 | <--- | AI威胁感知 | .785 | .677 | .867 | .002 |
| DM1 | <--- | 防御性动机 | .767 | .648 | .872 | .002 |
| DM2 | <--- | 防御性动机 | .712 | .574 | .831 | .002 |
| DM3 | <--- | 防御性动机 | .737 | .597 | .842 | .002 |
| DM5 | <--- | 防御性动机 | .731 | .596 | .835 | .002 |
| SE1 | <--- | AI自我效能 | .641 | .537 | .740 | .002 |
| SE2 | <--- | AI自我效能 | .728 | .593 | .832 | .002 |
| SE3 | <--- | AI自我效能 | .791 | .699 | .867 | .002 |
| SE4 | <--- | AI自我效能 | .859 | .764 | .931 | .002 |
| SE5 | <--- | AI自我效能 | .794 | .661 | .891 | .002 |
| AB1 | <--- | 使用行为 | .723 | .587 | .832 | .002 |
| AB2 | <--- | 使用行为 | .813 | .692 | .923 | .002 |
| AB3 | <--- | 使用行为 | .783 | .656 | .883 | .002 |
| AB5 | <--- | 使用行为 | .660 | .513 | .779 | .002 |

##### Variances: (others - H1a vs H1)

| Parameter | | | Estimate | Lower | Upper | P |
| --- | --- | --- | --- | --- | --- | --- |
| AI威胁感知 |  |  | 1.820 | 1.103 | 2.631 | .002 |
| e21 |  |  | 1.205 | .710 | 1.811 | .002 |
| e23 |  |  | 1.728 | .999 | 2.539 | .002 |
| e22 |  |  | 1.190 | .622 | 1.905 | .002 |
| e1 |  |  | 1.230 | .767 | 1.670 | .002 |
| e2 |  |  | 1.354 | .862 | 1.880 | .002 |
| e3 |  |  | 1.287 | .825 | 1.744 | .002 |
| e4 |  |  | 1.567 | 1.049 | 2.113 | .002 |
| e5 |  |  | 1.316 | .838 | 1.789 | .002 |
| e6 |  |  | 1.389 | .811 | 1.888 | .002 |
| e8 |  |  | 1.548 | .946 | 2.196 | .002 |
| e9 |  |  | 1.441 | .969 | 1.874 | .002 |
| e10 |  |  | 1.462 | .967 | 1.982 | .002 |
| e11 |  |  | 2.141 | 1.579 | 2.669 | .002 |
| e12 |  |  | 1.671 | 1.185 | 2.172 | .002 |
| e13 |  |  | 1.319 | .891 | 1.714 | .002 |
| e14 |  |  | 1.061 | .550 | 1.548 | .002 |
| e15 |  |  | 1.293 | .765 | 1.777 | .002 |
| e16 |  |  | 1.491 | 1.005 | 1.944 | .002 |
| e17 |  |  | 1.119 | .483 | 1.685 | .002 |
| e18 |  |  | 1.305 | .734 | 1.878 | .002 |
| e20 |  |  | 1.833 | 1.261 | 2.389 | .002 |

##### Squared Multiple Correlations: (others - H1a vs H1)

| Parameter | | | Estimate | Lower | Upper | P |
| --- | --- | --- | --- | --- | --- | --- |
| AI自我效能 |  |  | .193 | .115 | .299 | .002 |
| 防御性动机 |  |  | .131 | .033 | .310 | .002 |
| 使用行为 |  |  | .271 | .116 | .484 | .002 |
| AB5 |  |  | .436 | .263 | .608 | .002 |
| AB3 |  |  | .613 | .430 | .779 | .002 |
| AB2 |  |  | .661 | .478 | .852 | .002 |
| AB1 |  |  | .522 | .344 | .692 | .002 |
| SE5 |  |  | .630 | .437 | .794 | .002 |
| SE4 |  |  | .738 | .584 | .867 | .002 |
| SE3 |  |  | .626 | .489 | .752 | .002 |
| SE2 |  |  | .530 | .352 | .691 | .002 |
| SE1 |  |  | .411 | .288 | .547 | .002 |
| DM5 |  |  | .535 | .355 | .698 | .002 |
| DM3 |  |  | .543 | .356 | .709 | .002 |
| DM2 |  |  | .508 | .329 | .690 | .002 |
| DM1 |  |  | .589 | .420 | .761 | .002 |
| AA5 |  |  | .616 | .459 | .752 | .002 |
| AA4 |  |  | .512 | .311 | .685 | .002 |
| AA3 |  |  | .572 | .420 | .720 | .002 |
| AA2 |  |  | .589 | .394 | .745 | .002 |
| AA1 |  |  | .597 | .404 | .764 | .002 |

##### User-defined estimands: (others - H1a vs H1)

| Parameter | | | Estimate | Lower | Upper | P |
| --- | --- | --- | --- | --- | --- | --- |
| diff1 |  |  | .000 | .000 | .000 | ... |
| diff2 |  |  | -.029 | -.201 | .149 | .732 |
| diff3 |  |  | .145 | -.068 | .382 | .201 |
| diff4 |  |  | -.012 | -.200 | .180 | .886 |
| diff5 |  |  | -.244 | -.456 | -.015 | .032 |

##### Bias-corrected percentile method (others - H1a vs H1)

##### 95% confidence intervals (bias-corrected percentile method)

##### Scalar Estimates (others - H1a vs H1)

##### Regression Weights: (others - H1a vs H1)

| Parameter | | | Estimate | Lower | Upper | P |
| --- | --- | --- | --- | --- | --- | --- |
| AI自我效能 | <--- | AI威胁感知 | .398 | .303 | .505 | .002 |
| 防御性动机 | <--- | AI威胁感知 | .419 | .206 | .715 | .001 |
| 防御性动机 | <--- | AI自我效能 | -.152 | -.478 | .153 | .303 |
| 使用行为 | <--- | 防御性动机 | .396 | .220 | .624 | .001 |
| 使用行为 | <--- | AI自我效能 | .275 | .019 | .563 | .037 |
| AA1 | <--- | AI威胁感知 | 1.000 | 1.000 | 1.000 | ... |
| AA2 | <--- | AI威胁感知 | 1.032 | .840 | 1.324 | .001 |
| AA3 | <--- | AI威胁感知 | .973 | .816 | 1.159 | .002 |
| AA4 | <--- | AI威胁感知 | .951 | .729 | 1.258 | .001 |
| AA5 | <--- | AI威胁感知 | 1.076 | .856 | 1.379 | .001 |
| DM1 | <--- | 防御性动机 | 1.000 | 1.000 | 1.000 | ... |
| DM2 | <--- | 防御性动机 | .896 | .684 | 1.210 | .001 |
| DM3 | <--- | 防御性动机 | .927 | .668 | 1.248 | .001 |
| DM5 | <--- | 防御性动机 | .919 | .729 | 1.140 | .002 |
| SE1 | <--- | AI自我效能 | 1.000 | 1.000 | 1.000 | ... |
| SE2 | <--- | AI自我效能 | 1.123 | .816 | 1.466 | .003 |
| SE3 | <--- | AI自我效能 | 1.216 | .968 | 1.519 | .002 |
| SE4 | <--- | AI自我效能 | 1.416 | 1.117 | 1.823 | .002 |
| SE5 | <--- | AI自我效能 | 1.215 | .902 | 1.547 | .003 |
| AB1 | <--- | 使用行为 | 1.000 | 1.000 | 1.000 | ... |
| AB2 | <--- | 使用行为 | 1.157 | .927 | 1.569 | .001 |
| AB3 | <--- | 使用行为 | 1.125 | .880 | 1.495 | .002 |
| AB5 | <--- | 使用行为 | .931 | .678 | 1.236 | .002 |

##### Standardized Regression Weights: (others - H1a vs H1)

| Parameter | | | Estimate | Lower | Upper | P |
| --- | --- | --- | --- | --- | --- | --- |
| AI自我效能 | <--- | AI威胁感知 | .440 | .339 | .544 | .002 |
| 防御性动机 | <--- | AI威胁感知 | .401 | .196 | .600 | .001 |
| 防御性动机 | <--- | AI自我效能 | -.132 | -.380 | .126 | .303 |
| 使用行为 | <--- | 防御性动机 | .437 | .221 | .599 | .002 |
| 使用行为 | <--- | AI自我效能 | .264 | .023 | .484 | .036 |
| AA1 | <--- | AI威胁感知 | .772 | .627 | .864 | .003 |
| AA2 | <--- | AI威胁感知 | .767 | .637 | .866 | .002 |
| AA3 | <--- | AI威胁感知 | .756 | .629 | .840 | .004 |
| AA4 | <--- | AI威胁感知 | .716 | .570 | .833 | .001 |
| AA5 | <--- | AI威胁感知 | .785 | .665 | .860 | .004 |
| DM1 | <--- | 防御性动机 | .767 | .644 | .869 | .003 |
| DM2 | <--- | 防御性动机 | .712 | .572 | .830 | .002 |
| DM3 | <--- | 防御性动机 | .737 | .596 | .839 | .002 |
| DM5 | <--- | 防御性动机 | .731 | .584 | .830 | .003 |
| SE1 | <--- | AI自我效能 | .641 | .538 | .740 | .002 |
| SE2 | <--- | AI自我效能 | .728 | .590 | .831 | .002 |
| SE3 | <--- | AI自我效能 | .791 | .686 | .862 | .003 |
| SE4 | <--- | AI自我效能 | .859 | .758 | .930 | .003 |
| SE5 | <--- | AI自我效能 | .794 | .649 | .886 | .003 |
| AB1 | <--- | 使用行为 | .723 | .590 | .832 | .002 |
| AB2 | <--- | 使用行为 | .813 | .681 | .915 | .003 |
| AB3 | <--- | 使用行为 | .783 | .633 | .873 | .004 |
| AB5 | <--- | 使用行为 | .660 | .502 | .774 | .003 |

##### Variances: (others - H1a vs H1)

| Parameter | | | Estimate | Lower | Upper | P |
| --- | --- | --- | --- | --- | --- | --- |
| AI威胁感知 |  |  | 1.820 | 1.110 | 2.631 | .002 |
| e21 |  |  | 1.205 | .742 | 1.876 | .001 |
| e23 |  |  | 1.728 | 1.056 | 2.630 | .001 |
| e22 |  |  | 1.190 | .725 | 2.052 | .001 |
| e1 |  |  | 1.230 | .836 | 1.739 | .001 |
| e2 |  |  | 1.354 | .895 | 1.935 | .001 |
| e3 |  |  | 1.287 | .878 | 1.827 | .001 |
| e4 |  |  | 1.567 | 1.068 | 2.129 | .002 |
| e5 |  |  | 1.316 | .902 | 1.858 | .001 |
| e6 |  |  | 1.389 | .904 | 1.961 | .001 |
| e8 |  |  | 1.548 | .999 | 2.232 | .001 |
| e9 |  |  | 1.441 | .990 | 1.920 | .001 |
| e10 |  |  | 1.462 | 1.039 | 2.086 | .001 |
| e11 |  |  | 2.141 | 1.638 | 2.762 | .001 |
| e12 |  |  | 1.671 | 1.242 | 2.236 | .001 |
| e13 |  |  | 1.319 | .943 | 1.830 | .001 |
| e14 |  |  | 1.061 | .581 | 1.580 | .001 |
| e15 |  |  | 1.293 | .830 | 1.837 | .001 |
| e16 |  |  | 1.491 | 1.048 | 1.988 | .001 |
| e17 |  |  | 1.119 | .519 | 1.739 | .001 |
| e18 |  |  | 1.305 | .811 | 1.924 | .001 |
| e20 |  |  | 1.833 | 1.305 | 2.443 | .001 |

##### Squared Multiple Correlations: (others - H1a vs H1)

| Parameter | | | Estimate | Lower | Upper | P |
| --- | --- | --- | --- | --- | --- | --- |
| AI自我效能 |  |  | .193 | .115 | .296 | .002 |
| 防御性动机 |  |  | .131 | .025 | .284 | .004 |
| 使用行为 |  |  | .271 | .104 | .459 | .005 |
| AB5 |  |  | .436 | .252 | .599 | .003 |
| AB3 |  |  | .613 | .400 | .762 | .004 |
| AB2 |  |  | .661 | .464 | .838 | .003 |
| AB1 |  |  | .522 | .348 | .693 | .002 |
| SE5 |  |  | .630 | .421 | .785 | .003 |
| SE4 |  |  | .738 | .575 | .865 | .003 |
| SE3 |  |  | .626 | .471 | .742 | .003 |
| SE2 |  |  | .530 | .348 | .690 | .002 |
| SE1 |  |  | .411 | .289 | .548 | .002 |
| DM5 |  |  | .535 | .341 | .688 | .003 |
| DM3 |  |  | .543 | .355 | .703 | .002 |
| DM2 |  |  | .508 | .328 | .689 | .002 |
| DM1 |  |  | .589 | .415 | .756 | .003 |
| AA5 |  |  | .616 | .442 | .739 | .004 |
| AA4 |  |  | .512 | .325 | .695 | .001 |
| AA3 |  |  | .572 | .396 | .705 | .004 |
| AA2 |  |  | .589 | .406 | .750 | .002 |
| AA1 |  |  | .597 | .393 | .747 | .003 |

##### User-defined estimands: (others - H1a vs H1)

| Parameter | | | Estimate | Lower | Upper | P |
| --- | --- | --- | --- | --- | --- | --- |
| diff1 |  |  | .000 | .000 | .000 | ... |
| diff2 |  |  | -.029 | -.202 | .149 | .728 |
| diff3 |  |  | .145 | -.061 | .402 | .176 |
| diff4 |  |  | -.012 | -.200 | .171 | .855 |
| diff5 |  |  | -.244 | -.446 | -.011 | .035 |

##### Minimization History (H1a vs H1)

| Iteration |  | Negative eigenvalues | Condition # | Smallest eigenvalue | Diameter | F | NTries | Ratio |
| --- | --- | --- | --- | --- | --- | --- | --- | --- |
| 0 | e | 11 |  | -.647 | 9999.000 | 3579.805 | 0 | 9999.000 |
| 1 | e | 5 |  | -.045 | 3.417 | 1331.908 | 20 | .389 |
| 2 | e | 0 | 86.220 |  | 2.168 | 497.373 | 5 | .756 |
| 3 | e | 0 | 77.467 |  | .993 | 364.345 | 2 | .000 |
| 4 | e | 0 | 70.235 |  | .807 | 306.016 | 1 | 1.158 |
| 5 | e | 0 | 88.664 |  | .488 | 299.201 | 1 | 1.120 |
| 6 | e | 0 | 107.668 |  | .159 | 298.761 | 1 | 1.078 |
| 7 | e | 0 | 111.835 |  | .030 | 298.753 | 1 | 1.019 |
| 8 | e | 0 | 112.239 |  | .001 | 298.753 | 1 | 1.001 |

##### Bootstrap (H1a vs H1)

##### Summary of Bootstrap Iterations (H1a vs H1)

##### (H1a vs H1)

| Iterations | Method 0 | Method 1 | Method 2 |
| --- | --- | --- | --- |
| 1 | 0 | 0 | 0 |
| 2 | 0 | 0 | 0 |
| 3 | 0 | 0 | 0 |
| 4 | 0 | 0 | 0 |
| 5 | 0 | 0 | 0 |
| 6 | 0 | 0 | 0 |
| 7 | 0 | 19 | 0 |
| 8 | 0 | 90 | 0 |
| 9 | 0 | 232 | 0 |
| 10 | 0 | 196 | 0 |
| 11 | 0 | 229 | 0 |
| 12 | 0 | 99 | 0 |
| 13 | 0 | 66 | 0 |
| 14 | 0 | 37 | 0 |
| 15 | 0 | 16 | 0 |
| 16 | 0 | 8 | 0 |
| 17 | 0 | 3 | 0 |
| 18 | 0 | 1 | 0 |
| 19 | 0 | 4 | 0 |
| Total | 0 | 1000 | 0 |

0 bootstrap samples were unused because of a singular covariance matrix.

0 bootstrap samples were unused because a solution was not found.

1000 usable bootstrap samples were obtained.

##### Bootstrap Distributions (H1a vs H1)

##### ML discrepancy (implied vs sample) (H1a vs H1)

|  |  |  |
| --- | --- | --- |
|  |  | \|-------------------- |
|  | 454.984 | \|\* |
|  | 474.901 | \|\* |
|  | 494.817 | \|\*\*\*\* |
|  | 514.734 | \|\*\*\*\*\*\*\*\* |
|  | 534.651 | \|\*\*\*\*\*\*\*\*\*\*\* |
|  | 554.568 | \|\*\*\*\*\*\*\*\*\*\*\*\*\*\*\*\*\*\*\* |
|  | 574.485 | \|\*\*\*\*\*\*\*\*\*\*\*\*\*\*\*\* |
| N = 1000 | 594.402 | \|\*\*\*\*\*\*\*\*\*\*\*\*\*\*\*\*\*\* |
| Mean = 584.311 | 614.318 | \|\*\*\*\*\*\*\*\*\*\*\*\*\*\*\*\* |
| S. e. = 1.466 | 634.235 | \|\*\*\*\*\*\*\*\*\*\*\* |
|  | 654.152 | \|\*\*\*\*\*\* |
|  | 674.069 | \|\*\*\*\*\* |
|  | 693.986 | \|\*\* |
|  | 713.903 | \|\* |
|  | 733.819 | \|\* |
|  |  | \|-------------------- |

##### ML discrepancy (implied vs pop) (H1a vs H1)

|  |  |  |
| --- | --- | --- |
|  |  | \|-------------------- |
|  | 361.284 | \|\* |
|  | 369.319 | \|\*\* |
|  | 377.355 | \|\*\*\*\* |
|  | 385.390 | \|\*\*\*\*\*\*\*\*\*\* |
|  | 393.425 | \|\*\*\*\*\*\*\*\*\*\*\*\*\*\*\* |
|  | 401.460 | \|\*\*\*\*\*\*\*\*\*\*\*\*\*\*\*\*\* |
|  | 409.495 | \|\*\*\*\*\*\*\*\*\*\*\*\*\*\*\*\*\*\*\* |
| N = 1000 | 417.530 | \|\*\*\*\*\*\*\*\*\*\*\*\*\* |
| Mean = 408.263 | 425.565 | \|\*\*\*\*\*\*\*\*\* |
| S. e. = .597 | 433.600 | \|\*\*\*\*\*\*\* |
|  | 441.635 | \|\*\*\*\* |
|  | 449.670 | \|\*\* |
|  | 457.705 | \|\*\* |
|  | 465.740 | \|\* |
|  | 473.775 | \|\* |
|  |  | \|-------------------- |

##### K-L overoptimism (unstabilized) (H1a vs H1)

|  |  |  |
| --- | --- | --- |
|  |  | \|-------------------- |
|  | -148.775 | \|\* |
|  | -97.509 | \|\* |
|  | -46.243 | \|\*\* |
|  | 5.023 | \|\*\*\*\* |
|  | 56.289 | \|\*\*\*\*\*\*\*\*\*\* |
|  | 107.554 | \|\*\*\*\*\*\*\*\*\*\*\*\*\*\*\* |
|  | 158.820 | \|\*\*\*\*\*\*\*\*\*\*\*\*\*\*\*\*\*\*\* |
| N = 1000 | 210.086 | \|\*\*\*\*\*\*\*\*\*\*\*\*\*\*\*\*\*\*\* |
| Mean = 211.510 | 261.352 | \|\*\*\*\*\*\*\*\*\*\*\*\*\*\*\*\*\*\* |
| S. e. = 3.684 | 312.618 | \|\*\*\*\*\*\*\*\*\*\*\*\*\* |
|  | 363.883 | \|\*\*\*\*\*\*\*\* |
|  | 415.149 | \|\*\*\*\*\* |
|  | 466.415 | \|\*\*\* |
|  | 517.681 | \|\*\* |
|  | 568.947 | \|\* |
|  |  | \|-------------------- |

##### K-L overoptimism (stabilized) (H1a vs H1)

|  |  |  |
| --- | --- | --- |
|  |  | \|-------------------- |
|  | 85.777 | \|\* |
|  | 104.537 | \|\* |
|  | 123.298 | \|\*\* |
|  | 142.058 | \|\*\*\*\*\* |
|  | 160.818 | \|\*\*\*\*\*\*\*\* |
|  | 179.578 | \|\*\*\*\*\*\*\*\*\*\*\*\*\* |
|  | 198.339 | \|\*\*\*\*\*\*\*\*\*\*\*\*\*\*\*\*\*\*\* |
| N = 1000 | 217.099 | \|\*\*\*\*\*\*\*\*\*\*\*\*\* |
| Mean = 212.907 | 235.859 | \|\*\*\*\*\*\*\*\*\*\*\*\*\*\* |
| S. e. = 1.349 | 254.620 | \|\*\*\*\*\*\*\*\* |
|  | 273.380 | \|\*\*\*\*\*\*\* |
|  | 292.140 | \|\*\*\* |
|  | 310.901 | \|\*\* |
|  | 329.661 | \|\* |
|  | 348.421 | \|\* |
|  |  | \|-------------------- |

##### H2a vs H2 (H2a vs H2)

##### Notes for Model (H2a vs H2)

##### Computation of degrees of freedom (H2a vs H2)

|  |  |
| --- | --- |
| Number of distinct sample moments: | 342 |
| Number of distinct parameters to be estimated: | 81 |
| Degrees of freedom (342 - 81): | 261 |

##### Result (H2a vs H2)

Minimum was achieved

Chi-square = 289.057

Degrees of freedom = 261

Probability level = .112

##### Middle (Middle - H2a vs H2)

##### Estimates (Middle - H2a vs H2)

##### Scalar Estimates (Middle - H2a vs H2)

##### Maximum Likelihood Estimates

##### Regression Weights: (Middle - H2a vs H2)

|  |  |  | Estimate | S.E. | C.R. | P | Label |
| --- | --- | --- | --- | --- | --- | --- | --- |
| AI自我效能 | <--- | AI威胁感知 | .481 | .061 | 7.830 | \*\*\* | H1a |
| 防御性动机 | <--- | AI威胁感知 | .378 | .063 | 6.035 | \*\*\* | H2a |
| 防御性动机 | <--- | AI自我效能 | .141 | .081 | 1.740 | .082 | H5a |
| 使用行为 | <--- | 防御性动机 | .384 | .081 | 4.772 | \*\*\* | H4a |
| 使用行为 | <--- | AI自我效能 | .515 | .086 | 6.017 | \*\*\* | H3a |
| AA1 | <--- | AI威胁感知 | 1.000 |  |  |  |  |
| AA2 | <--- | AI威胁感知 | .950 | .064 | 14.753 | \*\*\* |  |
| AA3 | <--- | AI威胁感知 | .926 | .067 | 13.747 | \*\*\* |  |
| AA4 | <--- | AI威胁感知 | .936 | .066 | 14.195 | \*\*\* |  |
| AA5 | <--- | AI威胁感知 | .925 | .063 | 14.593 | \*\*\* |  |
| DM1 | <--- | 防御性动机 | 1.000 |  |  |  |  |
| DM2 | <--- | 防御性动机 | 1.068 | .101 | 10.582 | \*\*\* |  |
| DM3 | <--- | 防御性动机 | 1.082 | .101 | 10.730 | \*\*\* |  |
| DM5 | <--- | 防御性动机 | 1.013 | .100 | 10.087 | \*\*\* |  |
| SE1 | <--- | AI自我效能 | 1.000 |  |  |  |  |
| SE2 | <--- | AI自我效能 | 1.093 | .105 | 10.434 | \*\*\* |  |
| SE3 | <--- | AI自我效能 | .992 | .100 | 9.906 | \*\*\* |  |
| SE4 | <--- | AI自我效能 | 1.152 | .102 | 11.310 | \*\*\* |  |
| SE5 | <--- | AI自我效能 | 1.152 | .101 | 11.438 | \*\*\* |  |
| AB1 | <--- | 使用行为 | 1.000 |  |  |  |  |
| AB2 | <--- | 使用行为 | .923 | .078 | 11.788 | \*\*\* |  |
| AB3 | <--- | 使用行为 | 1.036 | .078 | 13.300 | \*\*\* |  |
| AB5 | <--- | 使用行为 | .962 | .079 | 12.192 | \*\*\* |  |

##### Standardized Regression Weights: (Middle - H2a vs H2)

|  |  |  | Estimate |
| --- | --- | --- | --- |
| AI自我效能 | <--- | AI威胁感知 | .580 |
| 防御性动机 | <--- | AI威胁感知 | .445 |
| 防御性动机 | <--- | AI自我效能 | .138 |
| 使用行为 | <--- | 防御性动机 | .341 |
| 使用行为 | <--- | AI自我效能 | .446 |
| AA1 | <--- | AI威胁感知 | .824 |
| AA2 | <--- | AI威胁感知 | .789 |
| AA3 | <--- | AI威胁感知 | .748 |
| AA4 | <--- | AI威胁感知 | .766 |
| AA5 | <--- | AI威胁感知 | .783 |
| DM1 | <--- | 防御性动机 | .688 |
| DM2 | <--- | 防御性动机 | .747 |
| DM3 | <--- | 防御性动机 | .761 |
| DM5 | <--- | 防御性动机 | .703 |
| SE1 | <--- | AI自我效能 | .675 |
| SE2 | <--- | AI自我效能 | .715 |
| SE3 | <--- | AI自我效能 | .674 |
| SE4 | <--- | AI自我效能 | .790 |
| SE5 | <--- | AI自我效能 | .802 |
| AB1 | <--- | 使用行为 | .774 |
| AB2 | <--- | 使用行为 | .719 |
| AB3 | <--- | 使用行为 | .812 |
| AB5 | <--- | 使用行为 | .743 |

##### Variances: (Middle - H2a vs H2)

|  |  |  | Estimate | S.E. | C.R. | P | Label |
| --- | --- | --- | --- | --- | --- | --- | --- |
| AI威胁感知 |  |  | 2.209 | .270 | 8.169 | \*\*\* |  |
| e21 |  |  | 1.010 | .174 | 5.797 | \*\*\* |  |
| e23 |  |  | 1.135 | .195 | 5.829 | \*\*\* |  |
| e22 |  |  | 1.143 | .175 | 6.531 | \*\*\* |  |
| e1 |  |  | 1.045 | .118 | 8.893 | \*\*\* |  |
| e2 |  |  | 1.208 | .127 | 9.541 | \*\*\* |  |
| e3 |  |  | 1.491 | .148 | 10.078 | \*\*\* |  |
| e4 |  |  | 1.360 | .138 | 9.861 | \*\*\* |  |
| e5 |  |  | 1.197 | .124 | 9.639 | \*\*\* |  |
| e6 |  |  | 1.774 | .183 | 9.679 | \*\*\* |  |
| e8 |  |  | 1.444 | .165 | 8.770 | \*\*\* |  |
| e9 |  |  | 1.355 | .160 | 8.482 | \*\*\* |  |
| e10 |  |  | 1.676 | .177 | 9.477 | \*\*\* |  |
| e11 |  |  | 1.819 | .175 | 10.387 | \*\*\* |  |
| e12 |  |  | 1.733 | .173 | 10.009 | \*\*\* |  |
| e13 |  |  | 1.804 | .174 | 10.397 | \*\*\* |  |
| e14 |  |  | 1.217 | .136 | 8.921 | \*\*\* |  |
| e15 |  |  | 1.122 | .129 | 8.673 | \*\*\* |  |
| e16 |  |  | 1.357 | .151 | 8.958 | \*\*\* |  |
| e17 |  |  | 1.607 | .164 | 9.789 | \*\*\* |  |
| e18 |  |  | 1.120 | .138 | 8.092 | \*\*\* |  |
| e20 |  |  | 1.521 | .161 | 9.477 | \*\*\* |  |

##### Squared Multiple Correlations: (Middle - H2a vs H2)

|  |  |  | Estimate |
| --- | --- | --- | --- |
| AI自我效能 |  |  | .336 |
| 防御性动机 |  |  | .288 |
| 使用行为 |  |  | .435 |
| AB5 |  |  | .552 |
| AB3 |  |  | .660 |
| AB2 |  |  | .518 |
| AB1 |  |  | .599 |
| SE5 |  |  | .643 |
| SE4 |  |  | .624 |
| SE3 |  |  | .454 |
| SE2 |  |  | .512 |
| SE1 |  |  | .455 |
| DM5 |  |  | .494 |
| DM3 |  |  | .579 |
| DM2 |  |  | .557 |
| DM1 |  |  | .473 |
| AA5 |  |  | .612 |
| AA4 |  |  | .587 |
| AA3 |  |  | .559 |
| AA2 |  |  | .622 |
| AA1 |  |  | .679 |

##### User-defined estimands: (Middle - H2a vs H2)

|  |  |  |  |
| --- | --- | --- | --- |
| diff1 |  |  | .335 |
| diff2 |  |  | .232 |
| diff3 |  |  | .369 |
| diff4 |  |  | .238 |
| diff5 |  |  | -.005 |

##### Modification Indices (Middle - H2a vs H2)

##### Covariances: (Middle - H2a vs H2)

|  |  |  | M.I. | Par Change |
| --- | --- | --- | --- | --- |
| e8 | <--> | e22 | 12.054 | .352 |
| e8 | <--> | e17 | 4.233 | .228 |
| e5 | <--> | e17 | 5.158 | -.222 |
| e4 | <--> | e15 | 4.341 | -.189 |
| e3 | <--> | e17 | 5.616 | .254 |
| e3 | <--> | e4 | 4.325 | -.203 |
| e2 | <--> | e22 | 9.029 | -.273 |
| e1 | <--> | e15 | 4.136 | .169 |
| e1 | <--> | e12 | 8.115 | -.278 |

##### Variances: (Middle - H2a vs H2)

|  |  |  | M.I. | Par Change |
| --- | --- | --- | --- | --- |

##### Regression Weights: (Middle - H2a vs H2)

|  |  |  | M.I. | Par Change |
| --- | --- | --- | --- | --- |
| DM2 | <--- | 使用行为 | 7.679 | .169 |
| DM2 | <--- | AB3 | 6.539 | .114 |
| DM2 | <--- | AB2 | 10.081 | .140 |
| DM2 | <--- | AB1 | 7.036 | .116 |
| AA2 | <--- | AB2 | 6.184 | -.098 |
| AA1 | <--- | SE2 | 4.862 | -.081 |

##### Bootstrap (Middle - H2a vs H2)

##### Bootstrap standard errors (Middle - H2a vs H2)

##### Scalar Estimates (Middle - H2a vs H2)

##### Regression Weights: (Middle - H2a vs H2)

| Parameter | | | SE | SE-SE | Mean | Bias | SE-Bias |
| --- | --- | --- | --- | --- | --- | --- | --- |
| AI自我效能 | <--- | AI威胁感知 | .058 | .001 | .481 | .001 | .002 |
| 防御性动机 | <--- | AI威胁感知 | .065 | .001 | .378 | .000 | .002 |
| 防御性动机 | <--- | AI自我效能 | .083 | .002 | .142 | .001 | .003 |
| 使用行为 | <--- | 防御性动机 | .079 | .002 | .384 | .000 | .003 |
| 使用行为 | <--- | AI自我效能 | .085 | .002 | .513 | -.002 | .003 |
| AA1 | <--- | AI威胁感知 | .000 | .000 | 1.000 | .000 | .000 |
| AA2 | <--- | AI威胁感知 | .052 | .001 | .953 | .003 | .002 |
| AA3 | <--- | AI威胁感知 | .055 | .001 | .925 | -.001 | .002 |
| AA4 | <--- | AI威胁感知 | .058 | .001 | .934 | -.003 | .002 |
| AA5 | <--- | AI威胁感知 | .058 | .001 | .927 | .002 | .002 |
| DM1 | <--- | 防御性动机 | .000 | .000 | 1.000 | .000 | .000 |
| DM2 | <--- | 防御性动机 | .097 | .002 | 1.073 | .004 | .003 |
| DM3 | <--- | 防御性动机 | .095 | .002 | 1.088 | .006 | .003 |
| DM5 | <--- | 防御性动机 | .103 | .002 | 1.018 | .006 | .003 |
| SE1 | <--- | AI自我效能 | .000 | .000 | 1.000 | .000 | .000 |
| SE2 | <--- | AI自我效能 | .093 | .002 | 1.092 | -.001 | .003 |
| SE3 | <--- | AI自我效能 | .088 | .002 | .995 | .002 | .003 |
| SE4 | <--- | AI自我效能 | .096 | .002 | 1.154 | .003 | .003 |
| SE5 | <--- | AI自我效能 | .096 | .002 | 1.151 | -.001 | .003 |
| AB1 | <--- | 使用行为 | .000 | .000 | 1.000 | .000 | .000 |
| AB2 | <--- | 使用行为 | .066 | .001 | .925 | .003 | .002 |
| AB3 | <--- | 使用行为 | .069 | .002 | 1.042 | .006 | .002 |
| AB5 | <--- | 使用行为 | .072 | .002 | .963 | .001 | .002 |

##### Standardized Regression Weights: (Middle - H2a vs H2)

| Parameter | | | SE | SE-SE | Mean | Bias | SE-Bias |
| --- | --- | --- | --- | --- | --- | --- | --- |
| AI自我效能 | <--- | AI威胁感知 | .046 | .001 | .577 | -.003 | .001 |
| 防御性动机 | <--- | AI威胁感知 | .070 | .002 | .445 | .000 | .002 |
| 防御性动机 | <--- | AI自我效能 | .079 | .002 | .139 | .001 | .002 |
| 使用行为 | <--- | 防御性动机 | .066 | .001 | .340 | -.001 | .002 |
| 使用行为 | <--- | AI自我效能 | .063 | .001 | .445 | -.001 | .002 |
| AA1 | <--- | AI威胁感知 | .026 | .001 | .823 | -.001 | .001 |
| AA2 | <--- | AI威胁感知 | .024 | .001 | .790 | .001 | .001 |
| AA3 | <--- | AI威胁感知 | .029 | .001 | .747 | -.001 | .001 |
| AA4 | <--- | AI威胁感知 | .029 | .001 | .764 | -.002 | .001 |
| AA5 | <--- | AI威胁感知 | .032 | .001 | .784 | .002 | .001 |
| DM1 | <--- | 防御性动机 | .042 | .001 | .686 | -.002 | .001 |
| DM2 | <--- | 防御性动机 | .035 | .001 | .745 | -.002 | .001 |
| DM3 | <--- | 防御性动机 | .034 | .001 | .761 | -.001 | .001 |
| DM5 | <--- | 防御性动机 | .038 | .001 | .701 | -.001 | .001 |
| SE1 | <--- | AI自我效能 | .038 | .001 | .676 | .001 | .001 |
| SE2 | <--- | AI自我效能 | .037 | .001 | .714 | -.001 | .001 |
| SE3 | <--- | AI自我效能 | .039 | .001 | .673 | .000 | .001 |
| SE4 | <--- | AI自我效能 | .027 | .001 | .790 | .000 | .001 |
| SE5 | <--- | AI自我效能 | .027 | .001 | .799 | -.003 | .001 |
| AB1 | <--- | 使用行为 | .032 | .001 | .772 | -.001 | .001 |
| AB2 | <--- | 使用行为 | .036 | .001 | .718 | -.001 | .001 |
| AB3 | <--- | 使用行为 | .027 | .001 | .813 | .001 | .001 |
| AB5 | <--- | 使用行为 | .034 | .001 | .741 | -.001 | .001 |

##### Variances: (Middle - H2a vs H2)

| Parameter | | | SE | SE-SE | Mean | Bias | SE-Bias |
| --- | --- | --- | --- | --- | --- | --- | --- |
| AI威胁感知 |  |  | .215 | .005 | 2.205 | -.003 | .007 |
| e21 |  |  | .160 | .004 | 1.017 | .007 | .005 |
| e23 |  |  | .191 | .004 | 1.120 | -.016 | .006 |
| e22 |  |  | .158 | .004 | 1.128 | -.015 | .005 |
| e1 |  |  | .125 | .003 | 1.041 | -.004 | .004 |
| e2 |  |  | .116 | .003 | 1.196 | -.012 | .004 |
| e3 |  |  | .129 | .003 | 1.477 | -.014 | .004 |
| e4 |  |  | .132 | .003 | 1.356 | -.004 | .004 |
| e5 |  |  | .146 | .003 | 1.175 | -.022 | .005 |
| e6 |  |  | .188 | .004 | 1.765 | -.009 | .006 |
| e8 |  |  | .158 | .004 | 1.438 | -.007 | .005 |
| e9 |  |  | .153 | .003 | 1.343 | -.012 | .005 |
| e10 |  |  | .165 | .004 | 1.667 | -.009 | .005 |
| e11 |  |  | .153 | .003 | 1.802 | -.017 | .005 |
| e12 |  |  | .198 | .004 | 1.723 | -.010 | .006 |
| e13 |  |  | .162 | .004 | 1.794 | -.010 | .005 |
| e14 |  |  | .128 | .003 | 1.207 | -.010 | .004 |
| e15 |  |  | .128 | .003 | 1.128 | .006 | .004 |
| e16 |  |  | .154 | .003 | 1.351 | -.006 | .005 |
| e17 |  |  | .167 | .004 | 1.599 | -.008 | .005 |
| e18 |  |  | .133 | .003 | 1.108 | -.012 | .004 |
| e20 |  |  | .152 | .003 | 1.508 | -.014 | .005 |

##### Squared Multiple Correlations: (Middle - H2a vs H2)

| Parameter | | | SE | SE-SE | Mean | Bias | SE-Bias |
| --- | --- | --- | --- | --- | --- | --- | --- |
| AI自我效能 |  |  | .053 | .001 | .335 | -.001 | .002 |
| 防御性动机 |  |  | .055 | .001 | .296 | .008 | .002 |
| 使用行为 |  |  | .058 | .001 | .439 | .003 | .002 |
| AB5 |  |  | .050 | .001 | .551 | -.001 | .002 |
| AB3 |  |  | .044 | .001 | .662 | .002 | .001 |
| AB2 |  |  | .052 | .001 | .517 | .000 | .002 |
| AB1 |  |  | .049 | .001 | .598 | -.001 | .002 |
| SE5 |  |  | .042 | .001 | .639 | -.003 | .001 |
| SE4 |  |  | .042 | .001 | .625 | .001 | .001 |
| SE3 |  |  | .052 | .001 | .455 | .001 | .002 |
| SE2 |  |  | .053 | .001 | .512 | .000 | .002 |
| SE1 |  |  | .051 | .001 | .458 | .003 | .002 |
| DM5 |  |  | .053 | .001 | .493 | -.001 | .002 |
| DM3 |  |  | .051 | .001 | .580 | .000 | .002 |
| DM2 |  |  | .052 | .001 | .556 | -.001 | .002 |
| DM1 |  |  | .057 | .001 | .473 | .000 | .002 |
| AA5 |  |  | .049 | .001 | .616 | .004 | .002 |
| AA4 |  |  | .044 | .001 | .585 | -.003 | .001 |
| AA3 |  |  | .044 | .001 | .559 | .000 | .001 |
| AA2 |  |  | .038 | .001 | .624 | .002 | .001 |
| AA1 |  |  | .042 | .001 | .678 | .000 | .001 |

##### User-defined estimands: (Middle - H2a vs H2)

| Parameter | | | SE | SE-SE | Mean | Bias | SE-Bias |
| --- | --- | --- | --- | --- | --- | --- | --- |
| diff1 |  |  | .100 | .002 | .333 | -.002 | .003 |
| diff2 |  |  | .098 | .002 | .229 | -.003 | .003 |
| diff3 |  |  | .118 | .003 | .365 | -.004 | .004 |
| diff4 |  |  | .115 | .003 | .236 | -.003 | .004 |
| diff5 |  |  | .121 | .003 | -.006 | -.001 | .004 |

##### Bootstrap Confidence (Middle - H2a vs H2)

##### Percentile method (Middle - H2a vs H2)

##### 95% confidence intervals (percentile method)

##### Scalar Estimates (Middle - H2a vs H2)

##### Regression Weights: (Middle - H2a vs H2)

| Parameter | | | Estimate | Lower | Upper | P |
| --- | --- | --- | --- | --- | --- | --- |
| AI自我效能 | <--- | AI威胁感知 | .481 | .371 | .592 | .002 |
| 防御性动机 | <--- | AI威胁感知 | .378 | .252 | .502 | .002 |
| 防御性动机 | <--- | AI自我效能 | .141 | -.020 | .308 | .076 |
| 使用行为 | <--- | 防御性动机 | .384 | .232 | .537 | .002 |
| 使用行为 | <--- | AI自我效能 | .515 | .351 | .682 | .002 |
| AA1 | <--- | AI威胁感知 | 1.000 | 1.000 | 1.000 | ... |
| AA2 | <--- | AI威胁感知 | .950 | .852 | 1.059 | .002 |
| AA3 | <--- | AI威胁感知 | .926 | .819 | 1.038 | .002 |
| AA4 | <--- | AI威胁感知 | .936 | .825 | 1.052 | .002 |
| AA5 | <--- | AI威胁感知 | .925 | .815 | 1.053 | .002 |
| DM1 | <--- | 防御性动机 | 1.000 | 1.000 | 1.000 | ... |
| DM2 | <--- | 防御性动机 | 1.068 | .902 | 1.283 | .002 |
| DM3 | <--- | 防御性动机 | 1.082 | .925 | 1.305 | .002 |
| DM5 | <--- | 防御性动机 | 1.013 | .835 | 1.243 | .002 |
| SE1 | <--- | AI自我效能 | 1.000 | 1.000 | 1.000 | ... |
| SE2 | <--- | AI自我效能 | 1.093 | .912 | 1.281 | .002 |
| SE3 | <--- | AI自我效能 | .992 | .835 | 1.173 | .002 |
| SE4 | <--- | AI自我效能 | 1.152 | .985 | 1.351 | .002 |
| SE5 | <--- | AI自我效能 | 1.152 | .974 | 1.345 | .002 |
| AB1 | <--- | 使用行为 | 1.000 | 1.000 | 1.000 | ... |
| AB2 | <--- | 使用行为 | .923 | .804 | 1.065 | .002 |
| AB3 | <--- | 使用行为 | 1.036 | .923 | 1.198 | .002 |
| AB5 | <--- | 使用行为 | .962 | .830 | 1.114 | .002 |

##### Standardized Regression Weights: (Middle - H2a vs H2)

| Parameter | | | Estimate | Lower | Upper | P |
| --- | --- | --- | --- | --- | --- | --- |
| AI自我效能 | <--- | AI威胁感知 | .580 | .484 | .660 | .002 |
| 防御性动机 | <--- | AI威胁感知 | .445 | .307 | .582 | .002 |
| 防御性动机 | <--- | AI自我效能 | .138 | -.021 | .287 | .076 |
| 使用行为 | <--- | 防御性动机 | .341 | .199 | .461 | .002 |
| 使用行为 | <--- | AI自我效能 | .446 | .309 | .566 | .002 |
| AA1 | <--- | AI威胁感知 | .824 | .770 | .872 | .002 |
| AA2 | <--- | AI威胁感知 | .789 | .737 | .836 | .002 |
| AA3 | <--- | AI威胁感知 | .748 | .684 | .802 | .002 |
| AA4 | <--- | AI威胁感知 | .766 | .704 | .819 | .002 |
| AA5 | <--- | AI威胁感知 | .783 | .722 | .843 | .002 |
| DM1 | <--- | 防御性动机 | .688 | .600 | .762 | .002 |
| DM2 | <--- | 防御性动机 | .747 | .675 | .807 | .002 |
| DM3 | <--- | 防御性动机 | .761 | .692 | .823 | .002 |
| DM5 | <--- | 防御性动机 | .703 | .624 | .771 | .002 |
| SE1 | <--- | AI自我效能 | .675 | .603 | .746 | .002 |
| SE2 | <--- | AI自我效能 | .715 | .633 | .788 | .002 |
| SE3 | <--- | AI自我效能 | .674 | .590 | .746 | .002 |
| SE4 | <--- | AI自我效能 | .790 | .733 | .841 | .002 |
| SE5 | <--- | AI自我效能 | .802 | .742 | .845 | .002 |
| AB1 | <--- | 使用行为 | .774 | .700 | .827 | .002 |
| AB2 | <--- | 使用行为 | .719 | .647 | .783 | .002 |
| AB3 | <--- | 使用行为 | .812 | .755 | .860 | .002 |
| AB5 | <--- | 使用行为 | .743 | .667 | .803 | .002 |

##### Variances: (Middle - H2a vs H2)

| Parameter | | | Estimate | Lower | Upper | P |
| --- | --- | --- | --- | --- | --- | --- |
| AI威胁感知 |  |  | 2.209 | 1.789 | 2.647 | .002 |
| e21 |  |  | 1.010 | .739 | 1.334 | .002 |
| e23 |  |  | 1.135 | .778 | 1.519 | .002 |
| e22 |  |  | 1.143 | .834 | 1.441 | .002 |
| e1 |  |  | 1.045 | .805 | 1.294 | .002 |
| e2 |  |  | 1.208 | .981 | 1.436 | .002 |
| e3 |  |  | 1.491 | 1.233 | 1.735 | .002 |
| e4 |  |  | 1.360 | 1.106 | 1.635 | .002 |
| e5 |  |  | 1.197 | .908 | 1.476 | .002 |
| e6 |  |  | 1.774 | 1.413 | 2.147 | .002 |
| e8 |  |  | 1.444 | 1.141 | 1.763 | .002 |
| e9 |  |  | 1.355 | 1.040 | 1.629 | .002 |
| e10 |  |  | 1.676 | 1.334 | 1.982 | .002 |
| e11 |  |  | 1.819 | 1.499 | 2.118 | .002 |
| e12 |  |  | 1.733 | 1.312 | 2.122 | .002 |
| e13 |  |  | 1.804 | 1.484 | 2.123 | .002 |
| e14 |  |  | 1.217 | .947 | 1.460 | .002 |
| e15 |  |  | 1.122 | .900 | 1.399 | .002 |
| e16 |  |  | 1.357 | 1.066 | 1.650 | .002 |
| e17 |  |  | 1.607 | 1.293 | 1.951 | .002 |
| e18 |  |  | 1.120 | .859 | 1.363 | .002 |
| e20 |  |  | 1.521 | 1.198 | 1.838 | .002 |

##### Squared Multiple Correlations: (Middle - H2a vs H2)

| Parameter | | | Estimate | Lower | Upper | P |
| --- | --- | --- | --- | --- | --- | --- |
| AI自我效能 |  |  | .336 | .234 | .436 | .002 |
| 防御性动机 |  |  | .288 | .185 | .407 | .002 |
| 使用行为 |  |  | .435 | .319 | .558 | .002 |
| AB5 |  |  | .552 | .445 | .645 | .002 |
| AB3 |  |  | .660 | .571 | .740 | .002 |
| AB2 |  |  | .518 | .418 | .613 | .002 |
| AB1 |  |  | .599 | .490 | .684 | .002 |
| SE5 |  |  | .643 | .550 | .714 | .002 |
| SE4 |  |  | .624 | .537 | .708 | .002 |
| SE3 |  |  | .454 | .349 | .556 | .002 |
| SE2 |  |  | .512 | .401 | .620 | .002 |
| SE1 |  |  | .455 | .363 | .557 | .002 |
| DM5 |  |  | .494 | .390 | .595 | .002 |
| DM3 |  |  | .579 | .479 | .678 | .002 |
| DM2 |  |  | .557 | .456 | .651 | .002 |
| DM1 |  |  | .473 | .360 | .581 | .002 |
| AA5 |  |  | .612 | .521 | .711 | .002 |
| AA4 |  |  | .587 | .496 | .670 | .002 |
| AA3 |  |  | .559 | .468 | .643 | .002 |
| AA2 |  |  | .622 | .543 | .698 | .002 |
| AA1 |  |  | .679 | .593 | .761 | .002 |

##### User-defined estimands: (Middle - H2a vs H2)

| Parameter | | | Estimate | Lower | Upper | P |
| --- | --- | --- | --- | --- | --- | --- |
| diff1 |  |  | .335 | .129 | .526 | .002 |
| diff2 |  |  | .232 | .046 | .431 | .014 |
| diff3 |  |  | .369 | .124 | .600 | .003 |
| diff4 |  |  | .238 | -.001 | .456 | .051 |
| diff5 |  |  | -.005 | -.259 | .219 | .989 |

##### Bias-corrected percentile method (Middle - H2a vs H2)

##### 95% confidence intervals (bias-corrected percentile method)

##### Scalar Estimates (Middle - H2a vs H2)

##### Regression Weights: (Middle - H2a vs H2)

| Parameter | | | Estimate | Lower | Upper | P |
| --- | --- | --- | --- | --- | --- | --- |
| AI自我效能 | <--- | AI威胁感知 | .481 | .373 | .597 | .002 |
| 防御性动机 | <--- | AI威胁感知 | .378 | .254 | .504 | .002 |
| 防御性动机 | <--- | AI自我效能 | .141 | -.021 | .308 | .077 |
| 使用行为 | <--- | 防御性动机 | .384 | .231 | .536 | .002 |
| 使用行为 | <--- | AI自我效能 | .515 | .369 | .703 | .001 |
| AA1 | <--- | AI威胁感知 | 1.000 | 1.000 | 1.000 | ... |
| AA2 | <--- | AI威胁感知 | .950 | .845 | 1.051 | .003 |
| AA3 | <--- | AI威胁感知 | .926 | .825 | 1.045 | .001 |
| AA4 | <--- | AI威胁感知 | .936 | .829 | 1.060 | .001 |
| AA5 | <--- | AI威胁感知 | .925 | .809 | 1.048 | .003 |
| DM1 | <--- | 防御性动机 | 1.000 | 1.000 | 1.000 | ... |
| DM2 | <--- | 防御性动机 | 1.068 | .902 | 1.283 | .002 |
| DM3 | <--- | 防御性动机 | 1.082 | .922 | 1.294 | .002 |
| DM5 | <--- | 防御性动机 | 1.013 | .835 | 1.242 | .002 |
| SE1 | <--- | AI自我效能 | 1.000 | 1.000 | 1.000 | ... |
| SE2 | <--- | AI自我效能 | 1.093 | .930 | 1.296 | .001 |
| SE3 | <--- | AI自我效能 | .992 | .835 | 1.173 | .002 |
| SE4 | <--- | AI自我效能 | 1.152 | .994 | 1.362 | .001 |
| SE5 | <--- | AI自我效能 | 1.152 | .981 | 1.347 | .002 |
| AB1 | <--- | 使用行为 | 1.000 | 1.000 | 1.000 | ... |
| AB2 | <--- | 使用行为 | .923 | .801 | 1.059 | .002 |
| AB3 | <--- | 使用行为 | 1.036 | .924 | 1.201 | .002 |
| AB5 | <--- | 使用行为 | .962 | .835 | 1.134 | .001 |

##### Standardized Regression Weights: (Middle - H2a vs H2)

| Parameter | | | Estimate | Lower | Upper | P |
| --- | --- | --- | --- | --- | --- | --- |
| AI自我效能 | <--- | AI威胁感知 | .580 | .487 | .664 | .002 |
| 防御性动机 | <--- | AI威胁感知 | .445 | .305 | .580 | .002 |
| 防御性动机 | <--- | AI自我效能 | .138 | -.024 | .281 | .086 |
| 使用行为 | <--- | 防御性动机 | .341 | .200 | .462 | .002 |
| 使用行为 | <--- | AI自我效能 | .446 | .305 | .562 | .003 |
| AA1 | <--- | AI威胁感知 | .824 | .767 | .868 | .003 |
| AA2 | <--- | AI威胁感知 | .789 | .735 | .832 | .003 |
| AA3 | <--- | AI威胁感知 | .748 | .685 | .803 | .002 |
| AA4 | <--- | AI威胁感知 | .766 | .710 | .821 | .001 |
| AA5 | <--- | AI威胁感知 | .783 | .716 | .839 | .003 |
| DM1 | <--- | 防御性动机 | .688 | .600 | .763 | .002 |
| DM2 | <--- | 防御性动机 | .747 | .676 | .807 | .002 |
| DM3 | <--- | 防御性动机 | .761 | .692 | .823 | .002 |
| DM5 | <--- | 防御性动机 | .703 | .629 | .775 | .001 |
| SE1 | <--- | AI自我效能 | .675 | .597 | .744 | .003 |
| SE2 | <--- | AI自我效能 | .715 | .630 | .784 | .002 |
| SE3 | <--- | AI自我效能 | .674 | .585 | .742 | .003 |
| SE4 | <--- | AI自我效能 | .790 | .731 | .841 | .002 |
| SE5 | <--- | AI自我效能 | .802 | .744 | .847 | .002 |
| AB1 | <--- | 使用行为 | .774 | .701 | .827 | .002 |
| AB2 | <--- | 使用行为 | .719 | .648 | .784 | .002 |
| AB3 | <--- | 使用行为 | .812 | .749 | .857 | .003 |
| AB5 | <--- | 使用行为 | .743 | .664 | .802 | .002 |

##### Variances: (Middle - H2a vs H2)

| Parameter | | | Estimate | Lower | Upper | P |
| --- | --- | --- | --- | --- | --- | --- |
| AI威胁感知 |  |  | 2.209 | 1.795 | 2.652 | .002 |
| e21 |  |  | 1.010 | .742 | 1.355 | .002 |
| e23 |  |  | 1.135 | .825 | 1.563 | .001 |
| e22 |  |  | 1.143 | .867 | 1.470 | .001 |
| e1 |  |  | 1.045 | .822 | 1.301 | .001 |
| e2 |  |  | 1.208 | 1.016 | 1.508 | .001 |
| e3 |  |  | 1.491 | 1.267 | 1.771 | .001 |
| e4 |  |  | 1.360 | 1.129 | 1.654 | .001 |
| e5 |  |  | 1.197 | .951 | 1.529 | .001 |
| e6 |  |  | 1.774 | 1.431 | 2.179 | .001 |
| e8 |  |  | 1.444 | 1.176 | 1.806 | .001 |
| e9 |  |  | 1.355 | 1.057 | 1.643 | .001 |
| e10 |  |  | 1.676 | 1.337 | 1.985 | .002 |
| e11 |  |  | 1.819 | 1.538 | 2.148 | .001 |
| e12 |  |  | 1.733 | 1.366 | 2.164 | .001 |
| e13 |  |  | 1.804 | 1.502 | 2.163 | .001 |
| e14 |  |  | 1.217 | .976 | 1.497 | .001 |
| e15 |  |  | 1.122 | .907 | 1.410 | .002 |
| e16 |  |  | 1.357 | 1.072 | 1.673 | .002 |
| e17 |  |  | 1.607 | 1.304 | 1.968 | .001 |
| e18 |  |  | 1.120 | .889 | 1.387 | .001 |
| e20 |  |  | 1.521 | 1.257 | 1.892 | .001 |

##### Squared Multiple Correlations: (Middle - H2a vs H2)

| Parameter | | | Estimate | Lower | Upper | P |
| --- | --- | --- | --- | --- | --- | --- |
| AI自我效能 |  |  | .336 | .237 | .441 | .002 |
| 防御性动机 |  |  | .288 | .180 | .394 | .004 |
| 使用行为 |  |  | .435 | .312 | .551 | .003 |
| AB5 |  |  | .552 | .441 | .643 | .002 |
| AB3 |  |  | .660 | .561 | .735 | .003 |
| AB2 |  |  | .518 | .419 | .615 | .002 |
| AB1 |  |  | .599 | .491 | .684 | .002 |
| SE5 |  |  | .643 | .553 | .717 | .002 |
| SE4 |  |  | .624 | .535 | .707 | .002 |
| SE3 |  |  | .454 | .342 | .551 | .003 |
| SE2 |  |  | .512 | .396 | .614 | .002 |
| SE1 |  |  | .455 | .356 | .554 | .003 |
| DM5 |  |  | .494 | .395 | .601 | .001 |
| DM3 |  |  | .579 | .479 | .678 | .002 |
| DM2 |  |  | .557 | .458 | .652 | .002 |
| DM1 |  |  | .473 | .360 | .582 | .002 |
| AA5 |  |  | .612 | .512 | .704 | .003 |
| AA4 |  |  | .587 | .504 | .674 | .001 |
| AA3 |  |  | .559 | .469 | .644 | .002 |
| AA2 |  |  | .622 | .540 | .691 | .003 |
| AA1 |  |  | .679 | .588 | .753 | .003 |

##### User-defined estimands: (Middle - H2a vs H2)

| Parameter | | | Estimate | Lower | Upper | P |
| --- | --- | --- | --- | --- | --- | --- |
| diff1 |  |  | .335 | .127 | .526 | .002 |
| diff2 |  |  | .232 | .057 | .445 | .010 |
| diff3 |  |  | .369 | .125 | .605 | .003 |
| diff4 |  |  | .238 | .011 | .462 | .044 |
| diff5 |  |  | -.005 | -.262 | .218 | .973 |

##### others (others - H2a vs H2)

##### Estimates (others - H2a vs H2)

##### Scalar Estimates (others - H2a vs H2)

##### Maximum Likelihood Estimates

##### Regression Weights: (others - H2a vs H2)

|  |  |  | Estimate | S.E. | C.R. | P | Label |
| --- | --- | --- | --- | --- | --- | --- | --- |
| AI自我效能 | <--- | AI威胁感知 | .146 | .074 | 1.970 | .049 | H1 |
| 防御性动机 | <--- | AI威胁感知 | .378 | .063 | 6.035 | \*\*\* | H2a |
| 防御性动机 | <--- | AI自我效能 | -.163 | .172 | -.950 | .342 | H5 |
| 使用行为 | <--- | 防御性动机 | .399 | .107 | 3.720 | \*\*\* | H4 |
| 使用行为 | <--- | AI自我效能 | .340 | .156 | 2.182 | .029 | H3 |
| AA1 | <--- | AI威胁感知 | 1.000 |  |  |  |  |
| AA2 | <--- | AI威胁感知 | .952 | .114 | 8.355 | \*\*\* |  |
| AA3 | <--- | AI威胁感知 | .893 | .109 | 8.165 | \*\*\* |  |
| AA4 | <--- | AI威胁感知 | .871 | .114 | 7.605 | \*\*\* |  |
| AA5 | <--- | AI威胁感知 | .995 | .116 | 8.615 | \*\*\* |  |
| DM1 | <--- | 防御性动机 | 1.000 |  |  |  |  |
| DM2 | <--- | 防御性动机 | .896 | .129 | 6.929 | \*\*\* |  |
| DM3 | <--- | 防御性动机 | .928 | .130 | 7.154 | \*\*\* |  |
| DM5 | <--- | 防御性动机 | .920 | .129 | 7.106 | \*\*\* |  |
| SE1 | <--- | AI自我效能 | 1.000 |  |  |  |  |
| SE2 | <--- | AI自我效能 | 1.368 | .273 | 5.007 | \*\*\* |  |
| SE3 | <--- | AI自我效能 | 1.493 | .283 | 5.271 | \*\*\* |  |
| SE4 | <--- | AI自我效能 | 1.709 | .314 | 5.441 | \*\*\* |  |
| SE5 | <--- | AI自我效能 | 1.479 | .281 | 5.259 | \*\*\* |  |
| AB1 | <--- | 使用行为 | 1.000 |  |  |  |  |
| AB2 | <--- | 使用行为 | 1.156 | .160 | 7.223 | \*\*\* |  |
| AB3 | <--- | 使用行为 | 1.124 | .160 | 7.043 | \*\*\* |  |
| AB5 | <--- | 使用行为 | .932 | .154 | 6.059 | \*\*\* |  |

##### Standardized Regression Weights: (others - H2a vs H2)

|  |  |  | Estimate |
| --- | --- | --- | --- |
| AI自我效能 | <--- | AI威胁感知 | .230 |
| 防御性动机 | <--- | AI威胁感知 | .389 |
| 防御性动机 | <--- | AI自我效能 | -.106 |
| 使用行为 | <--- | 防御性动机 | .448 |
| 使用行为 | <--- | AI自我效能 | .249 |
| AA1 | <--- | AI威胁感知 | .807 |
| AA2 | <--- | AI威胁感知 | .767 |
| AA3 | <--- | AI威胁感知 | .753 |
| AA4 | <--- | AI威胁感知 | .710 |
| AA5 | <--- | AI威胁感知 | .787 |
| DM1 | <--- | 防御性动机 | .769 |
| DM2 | <--- | 防御性动机 | .715 |
| DM3 | <--- | 防御性动机 | .739 |
| DM5 | <--- | 防御性动机 | .734 |
| SE1 | <--- | AI自我效能 | .535 |
| SE2 | <--- | AI自我效能 | .701 |
| SE3 | <--- | AI自我效能 | .774 |
| SE4 | <--- | AI自我效能 | .834 |
| SE5 | <--- | AI自我效能 | .771 |
| AB1 | <--- | 使用行为 | .720 |
| AB2 | <--- | 使用行为 | .810 |
| AB3 | <--- | 使用行为 | .780 |
| AB5 | <--- | 使用行为 | .657 |

##### Variances: (others - H2a vs H2)

|  |  |  | Estimate | S.E. | C.R. | P | Label |
| --- | --- | --- | --- | --- | --- | --- | --- |
| AI威胁感知 |  |  | 2.139 | .443 | 4.830 | \*\*\* |  |
| e21 |  |  | .813 | .294 | 2.761 | .006 |  |
| e23 |  |  | 1.728 | .411 | 4.201 | \*\*\* |  |
| e22 |  |  | 1.186 | .315 | 3.763 | \*\*\* |  |
| e1 |  |  | 1.143 | .213 | 5.367 | \*\*\* |  |
| e2 |  |  | 1.355 | .234 | 5.802 | \*\*\* |  |
| e3 |  |  | 1.304 | .220 | 5.925 | \*\*\* |  |
| e4 |  |  | 1.592 | .256 | 6.219 | \*\*\* |  |
| e5 |  |  | 1.304 | .233 | 5.604 | \*\*\* |  |
| e6 |  |  | 1.391 | .270 | 5.156 | \*\*\* |  |
| e8 |  |  | 1.549 | .270 | 5.731 | \*\*\* |  |
| e9 |  |  | 1.440 | .262 | 5.498 | \*\*\* |  |
| e10 |  |  | 1.461 | .263 | 5.554 | \*\*\* |  |
| e11 |  |  | 2.142 | .315 | 6.792 | \*\*\* |  |
| e12 |  |  | 1.661 | .270 | 6.160 | \*\*\* |  |
| e13 |  |  | 1.278 | .230 | 5.546 | \*\*\* |  |
| e14 |  |  | 1.094 | .235 | 4.660 | \*\*\* |  |
| e15 |  |  | 1.283 | .230 | 5.585 | \*\*\* |  |
| e16 |  |  | 1.490 | .258 | 5.773 | \*\*\* |  |
| e17 |  |  | 1.121 | .243 | 4.614 | \*\*\* |  |
| e18 |  |  | 1.306 | .256 | 5.092 | \*\*\* |  |
| e20 |  |  | 1.831 | .295 | 6.213 | \*\*\* |  |

##### Squared Multiple Correlations: (others - H2a vs H2)

|  |  |  | Estimate |
| --- | --- | --- | --- |
| AI自我效能 |  |  | .053 |
| 防御性动机 |  |  | .144 |
| 使用行为 |  |  | .259 |
| AB5 |  |  | .431 |
| AB3 |  |  | .608 |
| AB2 |  |  | .656 |
| AB1 |  |  | .518 |
| SE5 |  |  | .594 |
| SE4 |  |  | .696 |
| SE3 |  |  | .600 |
| SE2 |  |  | .491 |
| SE1 |  |  | .286 |
| DM5 |  |  | .539 |
| DM3 |  |  | .547 |
| DM2 |  |  | .511 |
| DM1 |  |  | .592 |
| AA5 |  |  | .619 |
| AA4 |  |  | .505 |
| AA3 |  |  | .567 |
| AA2 |  |  | .588 |
| AA1 |  |  | .652 |

##### User-defined estimands: (others - H2a vs H2)

|  |  |  |  |
| --- | --- | --- | --- |
| diff1 |  |  | .335 |
| diff2 |  |  | .232 |
| diff3 |  |  | .369 |
| diff4 |  |  | .238 |
| diff5 |  |  | -.005 |

##### Modification Indices (others - H2a vs H2)

##### Covariances: (others - H2a vs H2)

|  |  |  | M.I. | Par Change |
| --- | --- | --- | --- | --- |
| e20 | <--> | e21 | 5.872 | .332 |
| e17 | <--> | e23 | 5.181 | .411 |
| e14 | <--> | AI威胁感知 | 4.780 | .415 |
| e13 | <--> | e20 | 4.054 | .357 |
| e11 | <--> | e20 | 6.999 | -.560 |
| e10 | <--> | e20 | 8.003 | -.534 |
| e9 | <--> | e13 | 5.222 | -.376 |
| e5 | <--> | e16 | 4.297 | -.344 |
| e5 | <--> | e8 | 5.435 | .396 |
| e3 | <--> | e21 | 4.839 | .260 |

##### Variances: (others - H2a vs H2)

|  |  |  | M.I. | Par Change |
| --- | --- | --- | --- | --- |

##### Regression Weights: (others - H2a vs H2)

|  |  |  | M.I. | Par Change |
| --- | --- | --- | --- | --- |
| AB5 | <--- | AI自我效能 | 6.270 | .408 |
| AB5 | <--- | SE4 | 5.931 | .181 |
| AB5 | <--- | SE3 | 8.724 | .233 |
| AB5 | <--- | SE2 | 4.452 | .164 |
| AB5 | <--- | DM5 | 5.547 | -.186 |
| AB2 | <--- | 防御性动机 | 4.046 | .190 |
| AB2 | <--- | SE3 | 4.269 | -.142 |
| AB2 | <--- | DM5 | 4.552 | .147 |
| AB1 | <--- | AA5 | 5.438 | -.165 |
| SE4 | <--- | AI威胁感知 | 4.780 | .194 |
| SE4 | <--- | DM3 | 4.677 | .148 |
| SE4 | <--- | AA4 | 5.158 | .155 |
| SE4 | <--- | AA3 | 6.276 | .176 |
| SE4 | <--- | AA2 | 5.192 | .153 |
| SE3 | <--- | AA2 | 4.095 | -.137 |
| SE1 | <--- | AB5 | 4.118 | -.166 |
| DM5 | <--- | AB5 | 4.549 | -.156 |
| DM3 | <--- | SE4 | 5.261 | .158 |
| AA5 | <--- | AB1 | 4.013 | -.142 |
| AA3 | <--- | AI自我效能 | 4.524 | .299 |
| AA3 | <--- | SE4 | 5.710 | .153 |

##### Bootstrap (others - H2a vs H2)

##### Bootstrap standard errors (others - H2a vs H2)

##### Scalar Estimates (others - H2a vs H2)

##### Regression Weights: (others - H2a vs H2)

| Parameter | | | SE | SE-SE | Mean | Bias | SE-Bias |
| --- | --- | --- | --- | --- | --- | --- | --- |
| AI自我效能 | <--- | AI威胁感知 | .081 | .002 | .148 | .003 | .003 |
| 防御性动机 | <--- | AI威胁感知 | .065 | .001 | .378 | .000 | .002 |
| 防御性动机 | <--- | AI自我效能 | .197 | .004 | -.176 | -.013 | .006 |
| 使用行为 | <--- | 防御性动机 | .099 | .002 | .395 | -.003 | .003 |
| 使用行为 | <--- | AI自我效能 | .195 | .004 | .362 | .022 | .006 |
| AA1 | <--- | AI威胁感知 | .000 | .000 | 1.000 | .000 | .000 |
| AA2 | <--- | AI威胁感知 | .102 | .002 | .951 | -.001 | .003 |
| AA3 | <--- | AI威胁感知 | .074 | .002 | .896 | .003 | .002 |
| AA4 | <--- | AI威胁感知 | .114 | .003 | .866 | -.004 | .004 |
| AA5 | <--- | AI威胁感知 | .111 | .002 | 1.000 | .005 | .003 |
| DM1 | <--- | 防御性动机 | .000 | .000 | 1.000 | .000 | .000 |
| DM2 | <--- | 防御性动机 | .134 | .003 | .901 | .005 | .004 |
| DM3 | <--- | 防御性动机 | .145 | .003 | .929 | .001 | .005 |
| DM5 | <--- | 防御性动机 | .105 | .002 | .923 | .004 | .003 |
| SE1 | <--- | AI自我效能 | .000 | .000 | 1.000 | .000 | .000 |
| SE2 | <--- | AI自我效能 | .287 | .006 | 1.407 | .039 | .009 |
| SE3 | <--- | AI自我效能 | .304 | .007 | 1.545 | .052 | .010 |
| SE4 | <--- | AI自我效能 | .339 | .008 | 1.764 | .056 | .011 |
| SE5 | <--- | AI自我效能 | .298 | .007 | 1.532 | .052 | .009 |
| AB1 | <--- | 使用行为 | .000 | .000 | 1.000 | .000 | .000 |
| AB2 | <--- | 使用行为 | .160 | .004 | 1.171 | .015 | .005 |
| AB3 | <--- | 使用行为 | .159 | .004 | 1.137 | .012 | .005 |
| AB5 | <--- | 使用行为 | .146 | .003 | .943 | .011 | .005 |

##### Standardized Regression Weights: (others - H2a vs H2)

| Parameter | | | SE | SE-SE | Mean | Bias | SE-Bias |
| --- | --- | --- | --- | --- | --- | --- | --- |
| AI自我效能 | <--- | AI威胁感知 | .117 | .003 | .232 | .001 | .004 |
| 防御性动机 | <--- | AI威胁感知 | .072 | .002 | .387 | -.002 | .002 |
| 防御性动机 | <--- | AI自我效能 | .114 | .003 | -.107 | -.001 | .004 |
| 使用行为 | <--- | 防御性动机 | .100 | .002 | .445 | -.003 | .003 |
| 使用行为 | <--- | AI自我效能 | .111 | .002 | .251 | .002 | .004 |
| AA1 | <--- | AI威胁感知 | .047 | .001 | .806 | -.001 | .001 |
| AA2 | <--- | AI威胁感知 | .058 | .001 | .762 | -.005 | .002 |
| AA3 | <--- | AI威胁感知 | .053 | .001 | .753 | .000 | .002 |
| AA4 | <--- | AI威胁感知 | .068 | .002 | .702 | -.009 | .002 |
| AA5 | <--- | AI威胁感知 | .049 | .001 | .785 | -.002 | .002 |
| DM1 | <--- | 防御性动机 | .057 | .001 | .771 | .001 | .002 |
| DM2 | <--- | 防御性动机 | .067 | .001 | .714 | -.001 | .002 |
| DM3 | <--- | 防御性动机 | .060 | .001 | .736 | -.004 | .002 |
| DM5 | <--- | 防御性动机 | .060 | .001 | .734 | .000 | .002 |
| SE1 | <--- | AI自我效能 | .079 | .002 | .532 | -.003 | .002 |
| SE2 | <--- | AI自我效能 | .063 | .001 | .697 | -.004 | .002 |
| SE3 | <--- | AI自我效能 | .045 | .001 | .775 | .000 | .001 |
| SE4 | <--- | AI自我效能 | .052 | .001 | .832 | -.002 | .002 |
| SE5 | <--- | AI自我效能 | .061 | .001 | .770 | -.001 | .002 |
| AB1 | <--- | 使用行为 | .060 | .001 | .717 | -.003 | .002 |
| AB2 | <--- | 使用行为 | .060 | .001 | .810 | .000 | .002 |
| AB3 | <--- | 使用行为 | .059 | .001 | .780 | .000 | .002 |
| AB5 | <--- | 使用行为 | .068 | .002 | .656 | -.001 | .002 |

##### Variances: (others - H2a vs H2)

| Parameter | | | SE | SE-SE | Mean | Bias | SE-Bias |
| --- | --- | --- | --- | --- | --- | --- | --- |
| AI威胁感知 |  |  | .375 | .008 | 2.123 | -.016 | .012 |
| e21 |  |  | .272 | .006 | .806 | -.006 | .009 |
| e23 |  |  | .398 | .009 | 1.722 | -.006 | .013 |
| e22 |  |  | .314 | .007 | 1.162 | -.024 | .010 |
| e1 |  |  | .220 | .005 | 1.119 | -.024 | .007 |
| e2 |  |  | .256 | .006 | 1.338 | -.017 | .008 |
| e3 |  |  | .234 | .005 | 1.269 | -.035 | .007 |
| e4 |  |  | .270 | .006 | 1.585 | -.007 | .009 |
| e5 |  |  | .239 | .005 | 1.280 | -.025 | .008 |
| e6 |  |  | .273 | .006 | 1.359 | -.032 | .009 |
| e8 |  |  | .314 | .007 | 1.528 | -.020 | .010 |
| e9 |  |  | .235 | .005 | 1.420 | -.021 | .007 |
| e10 |  |  | .260 | .006 | 1.438 | -.024 | .008 |
| e11 |  |  | .277 | .006 | 2.099 | -.044 | .009 |
| e12 |  |  | .253 | .006 | 1.639 | -.022 | .008 |
| e13 |  |  | .217 | .005 | 1.250 | -.028 | .007 |
| e14 |  |  | .269 | .006 | 1.075 | -.019 | .009 |
| e15 |  |  | .254 | .006 | 1.255 | -.028 | .008 |
| e16 |  |  | .231 | .005 | 1.472 | -.019 | .007 |
| e17 |  |  | .309 | .007 | 1.098 | -.023 | .010 |
| e18 |  |  | .297 | .007 | 1.272 | -.034 | .009 |
| e20 |  |  | .289 | .006 | 1.803 | -.027 | .009 |

##### Squared Multiple Correlations: (others - H2a vs H2)

| Parameter | | | SE | SE-SE | Mean | Bias | SE-Bias |
| --- | --- | --- | --- | --- | --- | --- | --- |
| AI自我效能 |  |  | .054 | .001 | .067 | .014 | .002 |
| 防御性动机 |  |  | .056 | .001 | .160 | .017 | .002 |
| 使用行为 |  |  | .092 | .002 | .277 | .018 | .003 |
| AB5 |  |  | .087 | .002 | .435 | .003 | .003 |
| AB3 |  |  | .090 | .002 | .612 | .004 | .003 |
| AB2 |  |  | .096 | .002 | .659 | .003 | .003 |
| AB1 |  |  | .086 | .002 | .518 | .000 | .003 |
| SE5 |  |  | .092 | .002 | .597 | .003 | .003 |
| SE4 |  |  | .085 | .002 | .696 | -.001 | .003 |
| SE3 |  |  | .070 | .002 | .602 | .003 | .002 |
| SE2 |  |  | .086 | .002 | .490 | -.002 | .003 |
| SE1 |  |  | .082 | .002 | .289 | .003 | .003 |
| DM5 |  |  | .086 | .002 | .542 | .003 | .003 |
| DM3 |  |  | .087 | .002 | .545 | -.002 | .003 |
| DM2 |  |  | .095 | .002 | .514 | .003 | .003 |
| DM1 |  |  | .088 | .002 | .597 | .005 | .003 |
| AA5 |  |  | .076 | .002 | .619 | .000 | .002 |
| AA4 |  |  | .094 | .002 | .497 | -.008 | .003 |
| AA3 |  |  | .079 | .002 | .570 | .003 | .003 |
| AA2 |  |  | .087 | .002 | .584 | -.004 | .003 |
| AA1 |  |  | .075 | .002 | .652 | .000 | .002 |

##### User-defined estimands: (others - H2a vs H2)

| Parameter | | | SE | SE-SE | Mean | Bias | SE-Bias |
| --- | --- | --- | --- | --- | --- | --- | --- |
| diff1 |  |  | .100 | .002 | .333 | -.002 | .003 |
| diff2 |  |  | .098 | .002 | .229 | -.003 | .003 |
| diff3 |  |  | .118 | .003 | .365 | -.004 | .004 |
| diff4 |  |  | .115 | .003 | .236 | -.003 | .004 |
| diff5 |  |  | .121 | .003 | -.006 | -.001 | .004 |

##### Bootstrap Confidence (others - H2a vs H2)

##### Percentile method (others - H2a vs H2)

##### 95% confidence intervals (percentile method)

##### Scalar Estimates (others - H2a vs H2)

##### Regression Weights: (others - H2a vs H2)

| Parameter | | | Estimate | Lower | Upper | P |
| --- | --- | --- | --- | --- | --- | --- |
| AI自我效能 | <--- | AI威胁感知 | .146 | -.009 | .315 | .062 |
| 防御性动机 | <--- | AI威胁感知 | .378 | .252 | .502 | .002 |
| 防御性动机 | <--- | AI自我效能 | -.163 | -.608 | .190 | .356 |
| 使用行为 | <--- | 防御性动机 | .399 | .207 | .595 | .002 |
| 使用行为 | <--- | AI自我效能 | .340 | .036 | .800 | .030 |
| AA1 | <--- | AI威胁感知 | 1.000 | 1.000 | 1.000 | ... |
| AA2 | <--- | AI威胁感知 | .952 | .747 | 1.162 | .002 |
| AA3 | <--- | AI威胁感知 | .893 | .752 | 1.044 | .002 |
| AA4 | <--- | AI威胁感知 | .871 | .658 | 1.097 | .002 |
| AA5 | <--- | AI威胁感知 | .995 | .806 | 1.234 | .002 |
| DM1 | <--- | 防御性动机 | 1.000 | 1.000 | 1.000 | ... |
| DM2 | <--- | 防御性动机 | .896 | .679 | 1.193 | .002 |
| DM3 | <--- | 防御性动机 | .928 | .657 | 1.233 | .002 |
| DM5 | <--- | 防御性动机 | .920 | .734 | 1.145 | .002 |
| SE1 | <--- | AI自我效能 | 1.000 | 1.000 | 1.000 | ... |
| SE2 | <--- | AI自我效能 | 1.368 | .946 | 2.068 | .002 |
| SE3 | <--- | AI自我效能 | 1.493 | 1.123 | 2.225 | .002 |
| SE4 | <--- | AI自我效能 | 1.709 | 1.277 | 2.549 | .002 |
| SE5 | <--- | AI自我效能 | 1.479 | 1.062 | 2.228 | .002 |
| AB1 | <--- | 使用行为 | 1.000 | 1.000 | 1.000 | ... |
| AB2 | <--- | 使用行为 | 1.156 | .913 | 1.519 | .002 |
| AB3 | <--- | 使用行为 | 1.124 | .880 | 1.493 | .002 |
| AB5 | <--- | 使用行为 | .932 | .684 | 1.238 | .002 |

##### Standardized Regression Weights: (others - H2a vs H2)

| Parameter | | | Estimate | Lower | Upper | P |
| --- | --- | --- | --- | --- | --- | --- |
| AI自我效能 | <--- | AI威胁感知 | .230 | -.015 | .445 | .062 |
| 防御性动机 | <--- | AI威胁感知 | .389 | .251 | .533 | .002 |
| 防御性动机 | <--- | AI自我效能 | -.106 | -.318 | .125 | .356 |
| 使用行为 | <--- | 防御性动机 | .448 | .241 | .617 | .002 |
| 使用行为 | <--- | AI自我效能 | .249 | .027 | .460 | .030 |
| AA1 | <--- | AI威胁感知 | .807 | .704 | .891 | .002 |
| AA2 | <--- | AI威胁感知 | .767 | .631 | .861 | .002 |
| AA3 | <--- | AI威胁感知 | .753 | .645 | .844 | .002 |
| AA4 | <--- | AI威胁感知 | .710 | .552 | .821 | .002 |
| AA5 | <--- | AI威胁感知 | .787 | .677 | .869 | .002 |
| DM1 | <--- | 防御性动机 | .769 | .657 | .878 | .002 |
| DM2 | <--- | 防御性动机 | .715 | .579 | .831 | .002 |
| DM3 | <--- | 防御性动机 | .739 | .601 | .840 | .002 |
| DM5 | <--- | 防御性动机 | .734 | .599 | .838 | .002 |
| SE1 | <--- | AI自我效能 | .535 | .368 | .671 | .002 |
| SE2 | <--- | AI自我效能 | .701 | .557 | .811 | .002 |
| SE3 | <--- | AI自我效能 | .774 | .680 | .857 | .002 |
| SE4 | <--- | AI自我效能 | .834 | .723 | .921 | .002 |
| SE5 | <--- | AI自我效能 | .771 | .632 | .879 | .002 |
| AB1 | <--- | 使用行为 | .720 | .585 | .829 | .002 |
| AB2 | <--- | 使用行为 | .810 | .682 | .921 | .002 |
| AB3 | <--- | 使用行为 | .780 | .654 | .878 | .002 |
| AB5 | <--- | 使用行为 | .657 | .511 | .777 | .002 |

##### Variances: (others - H2a vs H2)

| Parameter | | | Estimate | Lower | Upper | P |
| --- | --- | --- | --- | --- | --- | --- |
| AI威胁感知 |  |  | 2.139 | 1.431 | 2.899 | .002 |
| e21 |  |  | .813 | .344 | 1.372 | .002 |
| e23 |  |  | 1.728 | .991 | 2.574 | .002 |
| e22 |  |  | 1.186 | .625 | 1.898 | .002 |
| e1 |  |  | 1.143 | .707 | 1.586 | .002 |
| e2 |  |  | 1.355 | .871 | 1.888 | .002 |
| e3 |  |  | 1.304 | .849 | 1.763 | .002 |
| e4 |  |  | 1.592 | 1.086 | 2.123 | .002 |
| e5 |  |  | 1.304 | .824 | 1.785 | .002 |
| e6 |  |  | 1.391 | .822 | 1.887 | .002 |
| e8 |  |  | 1.549 | .945 | 2.189 | .002 |
| e9 |  |  | 1.440 | .975 | 1.874 | .002 |
| e10 |  |  | 1.461 | .967 | 1.992 | .002 |
| e11 |  |  | 2.142 | 1.582 | 2.631 | .002 |
| e12 |  |  | 1.661 | 1.180 | 2.156 | .002 |
| e13 |  |  | 1.278 | .844 | 1.699 | .002 |
| e14 |  |  | 1.094 | .544 | 1.587 | .002 |
| e15 |  |  | 1.283 | .758 | 1.777 | .002 |
| e16 |  |  | 1.490 | 1.006 | 1.942 | .002 |
| e17 |  |  | 1.121 | .488 | 1.691 | .002 |
| e18 |  |  | 1.306 | .735 | 1.879 | .002 |
| e20 |  |  | 1.831 | 1.260 | 2.384 | .002 |

##### Squared Multiple Correlations: (others - H2a vs H2)

| Parameter | | | Estimate | Lower | Upper | P |
| --- | --- | --- | --- | --- | --- | --- |
| AI自我效能 |  |  | .053 | .001 | .198 | .002 |
| 防御性动机 |  |  | .144 | .068 | .286 | .002 |
| 使用行为 |  |  | .259 | .108 | .465 | .002 |
| AB5 |  |  | .431 | .261 | .604 | .002 |
| AB3 |  |  | .608 | .428 | .771 | .002 |
| AB2 |  |  | .656 | .466 | .848 | .002 |
| AB1 |  |  | .518 | .342 | .688 | .002 |
| SE5 |  |  | .594 | .399 | .772 | .002 |
| SE4 |  |  | .696 | .523 | .848 | .002 |
| SE3 |  |  | .600 | .463 | .734 | .002 |
| SE2 |  |  | .491 | .310 | .657 | .002 |
| SE1 |  |  | .286 | .136 | .450 | .002 |
| DM5 |  |  | .539 | .359 | .703 | .002 |
| DM3 |  |  | .547 | .361 | .706 | .002 |
| DM2 |  |  | .511 | .335 | .690 | .002 |
| DM1 |  |  | .592 | .431 | .770 | .002 |
| AA5 |  |  | .619 | .458 | .755 | .002 |
| AA4 |  |  | .505 | .305 | .675 | .002 |
| AA3 |  |  | .567 | .416 | .713 | .002 |
| AA2 |  |  | .588 | .398 | .741 | .002 |
| AA1 |  |  | .652 | .495 | .795 | .002 |

##### User-defined estimands: (others - H2a vs H2)

| Parameter | | | Estimate | Lower | Upper | P |
| --- | --- | --- | --- | --- | --- | --- |
| diff1 |  |  | .335 | .129 | .526 | .002 |
| diff2 |  |  | .232 | .046 | .431 | .014 |
| diff3 |  |  | .369 | .124 | .600 | .003 |
| diff4 |  |  | .238 | -.001 | .456 | .051 |
| diff5 |  |  | -.005 | -.259 | .219 | .989 |

##### Bias-corrected percentile method (others - H2a vs H2)

##### 95% confidence intervals (bias-corrected percentile method)

##### Scalar Estimates (others - H2a vs H2)

##### Regression Weights: (others - H2a vs H2)

| Parameter | | | Estimate | Lower | Upper | P |
| --- | --- | --- | --- | --- | --- | --- |
| AI自我效能 | <--- | AI威胁感知 | .146 | -.011 | .311 | .073 |
| 防御性动机 | <--- | AI威胁感知 | .378 | .254 | .504 | .002 |
| 防御性动机 | <--- | AI自我效能 | -.163 | -.591 | .214 | .388 |
| 使用行为 | <--- | 防御性动机 | .399 | .218 | .623 | .001 |
| 使用行为 | <--- | AI自我效能 | .340 | .045 | .808 | .027 |
| AA1 | <--- | AI威胁感知 | 1.000 | 1.000 | 1.000 | ... |
| AA2 | <--- | AI威胁感知 | .952 | .765 | 1.178 | .001 |
| AA3 | <--- | AI威胁感知 | .893 | .746 | 1.038 | .003 |
| AA4 | <--- | AI威胁感知 | .871 | .672 | 1.122 | .001 |
| AA5 | <--- | AI威胁感知 | .995 | .804 | 1.228 | .002 |
| DM1 | <--- | 防御性动机 | 1.000 | 1.000 | 1.000 | ... |
| DM2 | <--- | 防御性动机 | .896 | .683 | 1.201 | .001 |
| DM3 | <--- | 防御性动机 | .928 | .666 | 1.247 | .001 |
| DM5 | <--- | 防御性动机 | .920 | .740 | 1.157 | .002 |
| SE1 | <--- | AI自我效能 | 1.000 | 1.000 | 1.000 | ... |
| SE2 | <--- | AI自我效能 | 1.368 | .951 | 2.087 | .002 |
| SE3 | <--- | AI自我效能 | 1.493 | 1.123 | 2.214 | .002 |
| SE4 | <--- | AI自我效能 | 1.709 | 1.278 | 2.568 | .002 |
| SE5 | <--- | AI自我效能 | 1.479 | 1.041 | 2.158 | .003 |
| AB1 | <--- | 使用行为 | 1.000 | 1.000 | 1.000 | ... |
| AB2 | <--- | 使用行为 | 1.156 | .926 | 1.562 | .001 |
| AB3 | <--- | 使用行为 | 1.124 | .880 | 1.493 | .002 |
| AB5 | <--- | 使用行为 | .932 | .680 | 1.238 | .002 |

##### Standardized Regression Weights: (others - H2a vs H2)

| Parameter | | | Estimate | Lower | Upper | P |
| --- | --- | --- | --- | --- | --- | --- |
| AI自我效能 | <--- | AI威胁感知 | .230 | -.020 | .433 | .075 |
| 防御性动机 | <--- | AI威胁感知 | .389 | .265 | .548 | .001 |
| 防御性动机 | <--- | AI自我效能 | -.106 | -.318 | .129 | .361 |
| 使用行为 | <--- | 防御性动机 | .448 | .229 | .610 | .002 |
| 使用行为 | <--- | AI自我效能 | .249 | .017 | .452 | .035 |
| AA1 | <--- | AI威胁感知 | .807 | .700 | .885 | .003 |
| AA2 | <--- | AI威胁感知 | .767 | .631 | .861 | .002 |
| AA3 | <--- | AI威胁感知 | .753 | .627 | .840 | .003 |
| AA4 | <--- | AI威胁感知 | .710 | .567 | .828 | .001 |
| AA5 | <--- | AI威胁感知 | .787 | .669 | .864 | .003 |
| DM1 | <--- | 防御性动机 | .769 | .650 | .869 | .003 |
| DM2 | <--- | 防御性动机 | .715 | .576 | .831 | .002 |
| DM3 | <--- | 防御性动机 | .739 | .601 | .840 | .002 |
| DM5 | <--- | 防御性动机 | .734 | .593 | .832 | .003 |
| SE1 | <--- | AI自我效能 | .535 | .368 | .669 | .002 |
| SE2 | <--- | AI自我效能 | .701 | .553 | .810 | .002 |
| SE3 | <--- | AI自我效能 | .774 | .673 | .853 | .003 |
| SE4 | <--- | AI自我效能 | .834 | .711 | .917 | .003 |
| SE5 | <--- | AI自我效能 | .771 | .628 | .876 | .003 |
| AB1 | <--- | 使用行为 | .720 | .585 | .826 | .002 |
| AB2 | <--- | 使用行为 | .810 | .677 | .914 | .003 |
| AB3 | <--- | 使用行为 | .780 | .633 | .871 | .004 |
| AB5 | <--- | 使用行为 | .657 | .504 | .774 | .003 |

##### Variances: (others - H2a vs H2)

| Parameter | | | Estimate | Lower | Upper | P |
| --- | --- | --- | --- | --- | --- | --- |
| AI威胁感知 |  |  | 2.139 | 1.443 | 2.927 | .002 |
| e21 |  |  | .813 | .376 | 1.458 | .001 |
| e23 |  |  | 1.728 | 1.051 | 2.628 | .001 |
| e22 |  |  | 1.186 | .728 | 2.033 | .001 |
| e1 |  |  | 1.143 | .764 | 1.639 | .001 |
| e2 |  |  | 1.355 | .905 | 1.933 | .001 |
| e3 |  |  | 1.304 | .913 | 1.831 | .001 |
| e4 |  |  | 1.592 | 1.106 | 2.159 | .001 |
| e5 |  |  | 1.304 | .886 | 1.842 | .001 |
| e6 |  |  | 1.391 | .907 | 1.975 | .001 |
| e8 |  |  | 1.549 | .996 | 2.223 | .001 |
| e9 |  |  | 1.440 | .990 | 1.917 | .001 |
| e10 |  |  | 1.461 | 1.040 | 2.089 | .001 |
| e11 |  |  | 2.142 | 1.653 | 2.725 | .001 |
| e12 |  |  | 1.661 | 1.219 | 2.215 | .001 |
| e13 |  |  | 1.278 | .875 | 1.777 | .001 |
| e14 |  |  | 1.094 | .565 | 1.607 | .002 |
| e15 |  |  | 1.283 | .825 | 1.828 | .001 |
| e16 |  |  | 1.490 | 1.047 | 1.990 | .001 |
| e17 |  |  | 1.121 | .517 | 1.742 | .001 |
| e18 |  |  | 1.306 | .810 | 1.921 | .001 |
| e20 |  |  | 1.831 | 1.305 | 2.446 | .001 |

##### Squared Multiple Correlations: (others - H2a vs H2)

| Parameter | | | Estimate | Lower | Upper | P |
| --- | --- | --- | --- | --- | --- | --- |
| AI自我效能 |  |  | .053 | .000 | .187 | .003 |
| 防御性动机 |  |  | .144 | .055 | .258 | .006 |
| 使用行为 |  |  | .259 | .098 | .433 | .005 |
| AB5 |  |  | .431 | .254 | .599 | .003 |
| AB3 |  |  | .608 | .401 | .759 | .004 |
| AB2 |  |  | .656 | .459 | .836 | .003 |
| AB1 |  |  | .518 | .342 | .682 | .002 |
| SE5 |  |  | .594 | .394 | .767 | .003 |
| SE4 |  |  | .696 | .506 | .841 | .003 |
| SE3 |  |  | .600 | .453 | .728 | .003 |
| SE2 |  |  | .491 | .306 | .657 | .002 |
| SE1 |  |  | .286 | .135 | .447 | .002 |
| DM5 |  |  | .539 | .352 | .692 | .003 |
| DM3 |  |  | .547 | .361 | .706 | .002 |
| DM2 |  |  | .511 | .332 | .690 | .002 |
| DM1 |  |  | .592 | .423 | .755 | .003 |
| AA5 |  |  | .619 | .448 | .747 | .003 |
| AA4 |  |  | .505 | .322 | .686 | .001 |
| AA3 |  |  | .567 | .393 | .706 | .003 |
| AA2 |  |  | .588 | .398 | .741 | .002 |
| AA1 |  |  | .652 | .490 | .784 | .003 |

##### User-defined estimands: (others - H2a vs H2)

| Parameter | | | Estimate | Lower | Upper | P |
| --- | --- | --- | --- | --- | --- | --- |
| diff1 |  |  | .335 | .127 | .526 | .002 |
| diff2 |  |  | .232 | .057 | .445 | .010 |
| diff3 |  |  | .369 | .125 | .605 | .003 |
| diff4 |  |  | .238 | .011 | .462 | .044 |
| diff5 |  |  | -.005 | -.262 | .218 | .973 |

##### Minimization History (H2a vs H2)

| Iteration |  | Negative eigenvalues | Condition # | Smallest eigenvalue | Diameter | F | NTries | Ratio |
| --- | --- | --- | --- | --- | --- | --- | --- | --- |
| 0 | e | 11 |  | -.651 | 9999.000 | 3579.618 | 0 | 9999.000 |
| 1 | e | 4 |  | -.080 | 3.434 | 1312.948 | 20 | .388 |
| 2 | e | 0 | 209.165 |  | 2.441 | 494.852 | 5 | .678 |
| 3 | e | 0 | 107.519 |  | 1.568 | 347.919 | 1 | .884 |
| 4 | e | 0 | 109.530 |  | .597 | 293.336 | 1 | 1.125 |
| 5 | e | 0 | 139.214 |  | .366 | 289.288 | 1 | 1.097 |
| 6 | e | 0 | 204.439 |  | .139 | 289.063 | 1 | 1.082 |
| 7 | e | 0 | 218.422 |  | .040 | 289.057 | 1 | 1.031 |
| 8 | e | 0 | 218.424 |  | .002 | 289.057 | 1 | 1.002 |
| 9 | e | 0 | 218.424 |  | .000 | 289.057 | 1 | 1.000 |

##### Bootstrap (H2a vs H2)

##### Summary of Bootstrap Iterations (H2a vs H2)

##### (H2a vs H2)

| Iterations | Method 0 | Method 1 | Method 2 |
| --- | --- | --- | --- |
| 1 | 0 | 0 | 0 |
| 2 | 0 | 0 | 0 |
| 3 | 0 | 0 | 0 |
| 4 | 0 | 0 | 0 |
| 5 | 0 | 0 | 0 |
| 6 | 0 | 0 | 0 |
| 7 | 0 | 23 | 0 |
| 8 | 0 | 77 | 0 |
| 9 | 0 | 171 | 0 |
| 10 | 0 | 167 | 0 |
| 11 | 0 | 157 | 0 |
| 12 | 0 | 133 | 0 |
| 13 | 0 | 106 | 0 |
| 14 | 0 | 59 | 0 |
| 15 | 0 | 43 | 0 |
| 16 | 0 | 25 | 0 |
| 17 | 0 | 9 | 0 |
| 18 | 0 | 10 | 0 |
| 19 | 0 | 20 | 0 |
| Total | 0 | 1000 | 0 |

0 bootstrap samples were unused because of a singular covariance matrix.

0 bootstrap samples were unused because a solution was not found.

1000 usable bootstrap samples were obtained.

##### Bootstrap Distributions (H2a vs H2)

##### ML discrepancy (implied vs sample) (H2a vs H2)

|  |  |  |
| --- | --- | --- |
|  |  | \|-------------------- |
|  | 437.897 | \|\* |
|  | 458.751 | \|\* |
|  | 479.606 | \|\*\*\* |
|  | 500.461 | \|\*\*\*\*\*\* |
|  | 521.316 | \|\*\*\*\*\*\*\*\*\*\*\*\* |
|  | 542.170 | \|\*\*\*\*\*\*\*\*\*\*\*\*\*\*\*\*\* |
|  | 563.025 | \|\*\*\*\*\*\*\*\*\*\*\*\*\*\*\*\*\*\*\* |
| N = 1000 | 583.880 | \|\*\*\*\*\*\*\*\*\*\*\*\*\*\*\*\*\*\*\* |
| Mean = 574.440 | 604.735 | \|\*\*\*\*\*\*\*\*\*\*\*\*\*\*\*\* |
| S. e. = 1.453 | 625.590 | \|\*\*\*\*\*\*\*\*\*\*\* |
|  | 646.444 | \|\*\*\*\*\*\* |
|  | 667.299 | \|\*\*\*\* |
|  | 688.154 | \|\*\* |
|  | 709.009 | \|\* |
|  | 729.864 | \|\* |
|  |  | \|-------------------- |

##### ML discrepancy (implied vs pop) (H2a vs H2)

|  |  |  |
| --- | --- | --- |
|  |  | \|-------------------- |
|  | 352.175 | \|\* |
|  | 360.119 | \|\*\* |
|  | 368.064 | \|\*\*\*\*\* |
|  | 376.008 | \|\*\*\*\*\*\*\*\*\*\*\*\* |
|  | 383.952 | \|\*\*\*\*\*\*\*\*\*\*\*\*\*\*\*\* |
|  | 391.896 | \|\*\*\*\*\*\*\*\*\*\*\*\*\*\*\*\*\*\*\*\* |
|  | 399.841 | \|\*\*\*\*\*\*\*\*\*\*\*\*\*\*\*\*\*\*\*\* |
| N = 1000 | 407.785 | \|\*\*\*\*\*\*\*\*\*\*\*\*\*\*\* |
| Mean = 398.740 | 415.729 | \|\*\*\*\*\*\*\*\*\* |
| S. e. = .597 | 423.674 | \|\*\*\*\*\*\*\* |
|  | 431.618 | \|\*\*\*\*\* |
|  | 439.562 | \|\*\* |
|  | 447.506 | \|\*\* |
|  | 455.451 | \|\* |
|  | 463.395 | \|\* |
|  |  | \|-------------------- |

##### K-L overoptimism (unstabilized) (H2a vs H2)

|  |  |  |
| --- | --- | --- |
|  |  | \|-------------------- |
|  | -146.617 | \|\* |
|  | -96.120 | \| |
|  | -45.622 | \|\*\* |
|  | 4.876 | \|\*\*\*\* |
|  | 55.373 | \|\*\*\*\*\*\*\*\*\* |
|  | 105.871 | \|\*\*\*\*\*\*\*\*\*\*\*\*\* |
|  | 156.368 | \|\*\*\*\*\*\*\*\*\*\*\*\*\*\*\*\*\*\*\*\* |
| N = 1000 | 206.866 | \|\*\*\*\*\*\*\*\*\*\*\*\*\*\*\*\*\*\* |
| Mean = 211.858 | 257.364 | \|\*\*\*\*\*\*\*\*\*\*\*\*\*\*\*\*\*\* |
| S. e. = 3.690 | 307.861 | \|\*\*\*\*\*\*\*\*\*\*\*\* |
|  | 358.359 | \|\*\*\*\*\*\*\*\* |
|  | 408.857 | \|\*\*\*\*\* |
|  | 459.354 | \|\*\*\* |
|  | 509.852 | \|\*\* |
|  | 560.349 | \|\* |
|  |  | \|-------------------- |

##### K-L overoptimism (stabilized) (H2a vs H2)

|  |  |  |
| --- | --- | --- |
|  |  | \|-------------------- |
|  | 91.932 | \|\* |
|  | 110.528 | \|\* |
|  | 129.124 | \|\* |
|  | 147.720 | \|\*\*\*\*\*\*\* |
|  | 166.316 | \|\*\*\*\*\*\*\*\*\*\*\* |
|  | 184.912 | \|\*\*\*\*\*\*\*\*\*\*\*\*\*\*\* |
|  | 203.508 | \|\*\*\*\*\*\*\*\*\*\*\*\*\*\*\*\*\*\*\*\* |
| N = 1000 | 222.104 | \|\*\*\*\*\*\*\*\*\*\*\*\*\*\*\*\* |
| Mean = 213.255 | 240.700 | \|\*\*\*\*\*\*\*\*\*\*\*\* |
| S. e. = 1.347 | 259.296 | \|\*\*\*\*\*\*\*\*\*\* |
|  | 277.892 | \|\*\*\*\*\*\* |
|  | 296.488 | \|\*\*\* |
|  | 315.084 | \|\*\* |
|  | 333.680 | \|\* |
|  | 352.276 | \|\* |
|  |  | \|-------------------- |

##### H3a vs H3 (H3a vs H3)

##### Notes for Model (H3a vs H3)

##### Computation of degrees of freedom (H3a vs H3)

|  |  |
| --- | --- |
| Number of distinct sample moments: | 342 |
| Number of distinct parameters to be estimated: | 81 |
| Degrees of freedom (342 - 81): | 261 |

##### Result (H3a vs H3)

Minimum was achieved

Chi-square = 289.909

Degrees of freedom = 261

Probability level = .106

##### Middle (Middle - H3a vs H3)

##### Estimates (Middle - H3a vs H3)

##### Scalar Estimates (Middle - H3a vs H3)

##### Maximum Likelihood Estimates

##### Regression Weights: (Middle - H3a vs H3)

|  |  |  | Estimate | S.E. | C.R. | P | Label |
| --- | --- | --- | --- | --- | --- | --- | --- |
| AI自我效能 | <--- | AI威胁感知 | .486 | .062 | 7.879 | \*\*\* | H1a |
| 防御性动机 | <--- | AI威胁感知 | .378 | .074 | 5.089 | \*\*\* | H2a |
| 防御性动机 | <--- | AI自我效能 | .140 | .084 | 1.674 | .094 | H5a |
| 使用行为 | <--- | 防御性动机 | .394 | .080 | 4.921 | \*\*\* | H4a |
| 使用行为 | <--- | AI自我效能 | .482 | .076 | 6.373 | \*\*\* | H3a |
| AA1 | <--- | AI威胁感知 | 1.000 |  |  |  |  |
| AA2 | <--- | AI威胁感知 | .950 | .065 | 14.702 | \*\*\* |  |
| AA3 | <--- | AI威胁感知 | .926 | .068 | 13.705 | \*\*\* |  |
| AA4 | <--- | AI威胁感知 | .936 | .066 | 14.149 | \*\*\* |  |
| AA5 | <--- | AI威胁感知 | .925 | .064 | 14.543 | \*\*\* |  |
| DM1 | <--- | 防御性动机 | 1.000 |  |  |  |  |
| DM2 | <--- | 防御性动机 | 1.069 | .102 | 10.467 | \*\*\* |  |
| DM3 | <--- | 防御性动机 | 1.082 | .102 | 10.600 | \*\*\* |  |
| DM5 | <--- | 防御性动机 | 1.012 | .101 | 9.979 | \*\*\* |  |
| SE1 | <--- | AI自我效能 | 1.000 |  |  |  |  |
| SE2 | <--- | AI自我效能 | 1.082 | .102 | 10.586 | \*\*\* |  |
| SE3 | <--- | AI自我效能 | .982 | .098 | 10.037 | \*\*\* |  |
| SE4 | <--- | AI自我效能 | 1.139 | .099 | 11.497 | \*\*\* |  |
| SE5 | <--- | AI自我效能 | 1.139 | .098 | 11.633 | \*\*\* |  |
| AB1 | <--- | 使用行为 | 1.000 |  |  |  |  |
| AB2 | <--- | 使用行为 | .929 | .079 | 11.729 | \*\*\* |  |
| AB3 | <--- | 使用行为 | 1.044 | .079 | 13.244 | \*\*\* |  |
| AB5 | <--- | 使用行为 | .968 | .080 | 12.126 | \*\*\* |  |

##### Standardized Regression Weights: (Middle - H3a vs H3)

|  |  |  | Estimate |
| --- | --- | --- | --- |
| AI自我效能 | <--- | AI威胁感知 | .579 |
| 防御性动机 | <--- | AI威胁感知 | .445 |
| 防御性动机 | <--- | AI自我效能 | .139 |
| 使用行为 | <--- | 防御性动机 | .353 |
| 使用行为 | <--- | AI自我效能 | .427 |
| AA1 | <--- | AI威胁感知 | .824 |
| AA2 | <--- | AI威胁感知 | .789 |
| AA3 | <--- | AI威胁感知 | .748 |
| AA4 | <--- | AI威胁感知 | .766 |
| AA5 | <--- | AI威胁感知 | .783 |
| DM1 | <--- | 防御性动机 | .688 |
| DM2 | <--- | 防御性动机 | .747 |
| DM3 | <--- | 防御性动机 | .761 |
| DM5 | <--- | 防御性动机 | .702 |
| SE1 | <--- | AI自我效能 | .679 |
| SE2 | <--- | AI自我效能 | .716 |
| SE3 | <--- | AI自我效能 | .674 |
| SE4 | <--- | AI自我效能 | .790 |
| SE5 | <--- | AI自我效能 | .801 |
| AB1 | <--- | 使用行为 | .770 |
| AB2 | <--- | 使用行为 | .718 |
| AB3 | <--- | 使用行为 | .812 |
| AB5 | <--- | 使用行为 | .741 |

##### Variances: (Middle - H3a vs H3)

|  |  |  | Estimate | S.E. | C.R. | P | Label |
| --- | --- | --- | --- | --- | --- | --- | --- |
| AI威胁感知 |  |  | 2.209 | .272 | 8.134 | \*\*\* |  |
| e21 |  |  | 1.032 | .175 | 5.903 | \*\*\* |  |
| e23 |  |  | 1.133 | .196 | 5.792 | \*\*\* |  |
| e22 |  |  | 1.134 | .174 | 6.512 | \*\*\* |  |
| e1 |  |  | 1.045 | .118 | 8.886 | \*\*\* |  |
| e2 |  |  | 1.208 | .127 | 9.541 | \*\*\* |  |
| e3 |  |  | 1.491 | .148 | 10.079 | \*\*\* |  |
| e4 |  |  | 1.360 | .138 | 9.861 | \*\*\* |  |
| e5 |  |  | 1.197 | .124 | 9.639 | \*\*\* |  |
| e6 |  |  | 1.775 | .184 | 9.674 | \*\*\* |  |
| e8 |  |  | 1.442 | .165 | 8.764 | \*\*\* |  |
| e9 |  |  | 1.358 | .160 | 8.496 | \*\*\* |  |
| e10 |  |  | 1.678 | .177 | 9.487 | \*\*\* |  |
| e11 |  |  | 1.814 | .175 | 10.355 | \*\*\* |  |
| e12 |  |  | 1.732 | .173 | 10.005 | \*\*\* |  |
| e13 |  |  | 1.803 | .173 | 10.393 | \*\*\* |  |
| e14 |  |  | 1.218 | .137 | 8.921 | \*\*\* |  |
| e15 |  |  | 1.123 | .129 | 8.673 | \*\*\* |  |
| e16 |  |  | 1.362 | .151 | 9.001 | \*\*\* |  |
| e17 |  |  | 1.606 | .164 | 9.778 | \*\*\* |  |
| e18 |  |  | 1.115 | .139 | 8.051 | \*\*\* |  |
| e20 |  |  | 1.522 | .161 | 9.469 | \*\*\* |  |

##### Squared Multiple Correlations: (Middle - H3a vs H3)

|  |  |  | Estimate |
| --- | --- | --- | --- |
| AI自我效能 |  |  | .336 |
| 防御性动机 |  |  | .289 |
| 使用行为 |  |  | .427 |
| AB5 |  |  | .549 |
| AB3 |  |  | .660 |
| AB2 |  |  | .516 |
| AB1 |  |  | .592 |
| SE5 |  |  | .642 |
| SE4 |  |  | .623 |
| SE3 |  |  | .454 |
| SE2 |  |  | .512 |
| SE1 |  |  | .461 |
| DM5 |  |  | .493 |
| DM3 |  |  | .579 |
| DM2 |  |  | .558 |
| DM1 |  |  | .473 |
| AA5 |  |  | .612 |
| AA4 |  |  | .587 |
| AA3 |  |  | .559 |
| AA2 |  |  | .623 |
| AA1 |  |  | .679 |

##### User-defined estimands: (Middle - H3a vs H3)

|  |  |  |  |
| --- | --- | --- | --- |
| diff1 |  |  | .350 |
| diff2 |  |  | .242 |
| diff3 |  |  | .346 |
| diff4 |  |  | .258 |
| diff5 |  |  | .004 |

##### Modification Indices (Middle - H3a vs H3)

##### Covariances: (Middle - H3a vs H3)

|  |  |  | M.I. | Par Change |
| --- | --- | --- | --- | --- |
| e8 | <--> | e22 | 11.856 | .347 |
| e8 | <--> | e17 | 4.194 | .226 |
| e5 | <--> | e17 | 5.163 | -.223 |
| e4 | <--> | e15 | 4.311 | -.189 |
| e3 | <--> | e17 | 5.607 | .254 |
| e3 | <--> | e4 | 4.322 | -.203 |
| e2 | <--> | e22 | 8.872 | -.269 |
| e1 | <--> | e15 | 4.116 | .169 |
| e1 | <--> | e12 | 8.145 | -.278 |

##### Variances: (Middle - H3a vs H3)

|  |  |  | M.I. | Par Change |
| --- | --- | --- | --- | --- |

##### Regression Weights: (Middle - H3a vs H3)

|  |  |  | M.I. | Par Change |
| --- | --- | --- | --- | --- |
| DM2 | <--- | 使用行为 | 7.488 | .169 |
| DM2 | <--- | AB3 | 6.350 | .112 |
| DM2 | <--- | AB2 | 9.878 | .139 |
| DM2 | <--- | AB1 | 6.904 | .116 |
| AA2 | <--- | AB2 | 6.206 | -.099 |
| AA1 | <--- | SE2 | 4.866 | -.081 |

##### Bootstrap (Middle - H3a vs H3)

##### Bootstrap standard errors (Middle - H3a vs H3)

##### Scalar Estimates (Middle - H3a vs H3)

##### Regression Weights: (Middle - H3a vs H3)

| Parameter | | | SE | SE-SE | Mean | Bias | SE-Bias |
| --- | --- | --- | --- | --- | --- | --- | --- |
| AI自我效能 | <--- | AI威胁感知 | .058 | .001 | .486 | .000 | .002 |
| 防御性动机 | <--- | AI威胁感知 | .076 | .002 | .381 | .003 | .002 |
| 防御性动机 | <--- | AI自我效能 | .087 | .002 | .140 | .000 | .003 |
| 使用行为 | <--- | 防御性动机 | .079 | .002 | .393 | -.001 | .002 |
| 使用行为 | <--- | AI自我效能 | .079 | .002 | .483 | .000 | .003 |
| AA1 | <--- | AI威胁感知 | .000 | .000 | 1.000 | .000 | .000 |
| AA2 | <--- | AI威胁感知 | .052 | .001 | .953 | .003 | .002 |
| AA3 | <--- | AI威胁感知 | .055 | .001 | .925 | -.001 | .002 |
| AA4 | <--- | AI威胁感知 | .058 | .001 | .934 | -.002 | .002 |
| AA5 | <--- | AI威胁感知 | .058 | .001 | .928 | .002 | .002 |
| DM1 | <--- | 防御性动机 | .000 | .000 | 1.000 | .000 | .000 |
| DM2 | <--- | 防御性动机 | .100 | .002 | 1.073 | .004 | .003 |
| DM3 | <--- | 防御性动机 | .096 | .002 | 1.087 | .005 | .003 |
| DM5 | <--- | 防御性动机 | .103 | .002 | 1.017 | .005 | .003 |
| SE1 | <--- | AI自我效能 | .000 | .000 | 1.000 | .000 | .000 |
| SE2 | <--- | AI自我效能 | .091 | .002 | 1.082 | .000 | .003 |
| SE3 | <--- | AI自我效能 | .086 | .002 | .985 | .003 | .003 |
| SE4 | <--- | AI自我效能 | .093 | .002 | 1.143 | .004 | .003 |
| SE5 | <--- | AI自我效能 | .093 | .002 | 1.139 | .000 | .003 |
| AB1 | <--- | 使用行为 | .000 | .000 | 1.000 | .000 | .000 |
| AB2 | <--- | 使用行为 | .067 | .002 | .931 | .002 | .002 |
| AB3 | <--- | 使用行为 | .070 | .002 | 1.050 | .005 | .002 |
| AB5 | <--- | 使用行为 | .073 | .002 | .969 | .001 | .002 |

##### Standardized Regression Weights: (Middle - H3a vs H3)

| Parameter | | | SE | SE-SE | Mean | Bias | SE-Bias |
| --- | --- | --- | --- | --- | --- | --- | --- |
| AI自我效能 | <--- | AI威胁感知 | .046 | .001 | .577 | -.002 | .001 |
| 防御性动机 | <--- | AI威胁感知 | .079 | .002 | .447 | .002 | .003 |
| 防御性动机 | <--- | AI自我效能 | .083 | .002 | .138 | .000 | .003 |
| 使用行为 | <--- | 防御性动机 | .065 | .001 | .351 | -.002 | .002 |
| 使用行为 | <--- | AI自我效能 | .063 | .001 | .427 | .000 | .002 |
| AA1 | <--- | AI威胁感知 | .026 | .001 | .823 | -.001 | .001 |
| AA2 | <--- | AI威胁感知 | .024 | .001 | .790 | .001 | .001 |
| AA3 | <--- | AI威胁感知 | .029 | .001 | .747 | -.001 | .001 |
| AA4 | <--- | AI威胁感知 | .029 | .001 | .764 | -.002 | .001 |
| AA5 | <--- | AI威胁感知 | .032 | .001 | .784 | .002 | .001 |
| DM1 | <--- | 防御性动机 | .043 | .001 | .687 | -.001 | .001 |
| DM2 | <--- | 防御性动机 | .035 | .001 | .745 | -.002 | .001 |
| DM3 | <--- | 防御性动机 | .034 | .001 | .760 | -.001 | .001 |
| DM5 | <--- | 防御性动机 | .038 | .001 | .701 | -.001 | .001 |
| SE1 | <--- | AI自我效能 | .037 | .001 | .680 | .001 | .001 |
| SE2 | <--- | AI自我效能 | .037 | .001 | .715 | -.001 | .001 |
| SE3 | <--- | AI自我效能 | .039 | .001 | .674 | .000 | .001 |
| SE4 | <--- | AI自我效能 | .027 | .001 | .790 | .000 | .001 |
| SE5 | <--- | AI自我效能 | .027 | .001 | .799 | -.003 | .001 |
| AB1 | <--- | 使用行为 | .033 | .001 | .769 | -.001 | .001 |
| AB2 | <--- | 使用行为 | .036 | .001 | .717 | -.001 | .001 |
| AB3 | <--- | 使用行为 | .027 | .001 | .813 | .001 | .001 |
| AB5 | <--- | 使用行为 | .034 | .001 | .740 | -.001 | .001 |

##### Variances: (Middle - H3a vs H3)

| Parameter | | | SE | SE-SE | Mean | Bias | SE-Bias |
| --- | --- | --- | --- | --- | --- | --- | --- |
| AI威胁感知 |  |  | .214 | .005 | 2.203 | -.005 | .007 |
| e21 |  |  | .160 | .004 | 1.037 | .005 | .005 |
| e23 |  |  | .190 | .004 | 1.117 | -.016 | .006 |
| e22 |  |  | .160 | .004 | 1.121 | -.013 | .005 |
| e1 |  |  | .125 | .003 | 1.042 | -.003 | .004 |
| e2 |  |  | .116 | .003 | 1.196 | -.011 | .004 |
| e3 |  |  | .129 | .003 | 1.477 | -.014 | .004 |
| e4 |  |  | .133 | .003 | 1.356 | -.004 | .004 |
| e5 |  |  | .146 | .003 | 1.175 | -.022 | .005 |
| e6 |  |  | .189 | .004 | 1.765 | -.010 | .006 |
| e8 |  |  | .158 | .004 | 1.436 | -.006 | .005 |
| e9 |  |  | .153 | .003 | 1.346 | -.012 | .005 |
| e10 |  |  | .165 | .004 | 1.669 | -.009 | .005 |
| e11 |  |  | .153 | .003 | 1.798 | -.016 | .005 |
| e12 |  |  | .198 | .004 | 1.721 | -.010 | .006 |
| e13 |  |  | .162 | .004 | 1.793 | -.010 | .005 |
| e14 |  |  | .128 | .003 | 1.207 | -.011 | .004 |
| e15 |  |  | .128 | .003 | 1.128 | .006 | .004 |
| e16 |  |  | .155 | .003 | 1.356 | -.006 | .005 |
| e17 |  |  | .167 | .004 | 1.598 | -.008 | .005 |
| e18 |  |  | .133 | .003 | 1.103 | -.012 | .004 |
| e20 |  |  | .153 | .003 | 1.508 | -.014 | .005 |

##### Squared Multiple Correlations: (Middle - H3a vs H3)

| Parameter | | | SE | SE-SE | Mean | Bias | SE-Bias |
| --- | --- | --- | --- | --- | --- | --- | --- |
| AI自我效能 |  |  | .053 | .001 | .335 | -.001 | .002 |
| 防御性动机 |  |  | .058 | .001 | .298 | .010 | .002 |
| 使用行为 |  |  | .059 | .001 | .431 | .004 | .002 |
| AB5 |  |  | .050 | .001 | .549 | -.001 | .002 |
| AB3 |  |  | .044 | .001 | .661 | .002 | .001 |
| AB2 |  |  | .052 | .001 | .515 | .000 | .002 |
| AB1 |  |  | .050 | .001 | .592 | -.001 | .002 |
| SE5 |  |  | .042 | .001 | .639 | -.003 | .001 |
| SE4 |  |  | .042 | .001 | .625 | .001 | .001 |
| SE3 |  |  | .052 | .001 | .455 | .001 | .002 |
| SE2 |  |  | .053 | .001 | .512 | .000 | .002 |
| SE1 |  |  | .050 | .001 | .464 | .002 | .002 |
| DM5 |  |  | .053 | .001 | .493 | .000 | .002 |
| DM3 |  |  | .052 | .001 | .579 | .000 | .002 |
| DM2 |  |  | .052 | .001 | .557 | -.001 | .002 |
| DM1 |  |  | .058 | .001 | .473 | .000 | .002 |
| AA5 |  |  | .049 | .001 | .616 | .004 | .002 |
| AA4 |  |  | .044 | .001 | .585 | -.003 | .001 |
| AA3 |  |  | .044 | .001 | .559 | .000 | .001 |
| AA2 |  |  | .038 | .001 | .624 | .002 | .001 |
| AA1 |  |  | .042 | .001 | .678 | -.001 | .001 |

##### User-defined estimands: (Middle - H3a vs H3)

| Parameter | | | SE | SE-SE | Mean | Bias | SE-Bias |
| --- | --- | --- | --- | --- | --- | --- | --- |
| diff1 |  |  | .095 | .002 | .347 | -.003 | .003 |
| diff2 |  |  | .105 | .002 | .242 | -.001 | .003 |
| diff3 |  |  | .116 | .003 | .343 | -.003 | .004 |
| diff4 |  |  | .109 | .002 | .254 | -.004 | .003 |
| diff5 |  |  | .118 | .003 | .001 | -.003 | .004 |

##### Bootstrap Confidence (Middle - H3a vs H3)

##### Percentile method (Middle - H3a vs H3)

##### 95% confidence intervals (percentile method)

##### Scalar Estimates (Middle - H3a vs H3)

##### Regression Weights: (Middle - H3a vs H3)

| Parameter | | | Estimate | Lower | Upper | P |
| --- | --- | --- | --- | --- | --- | --- |
| AI自我效能 | <--- | AI威胁感知 | .486 | .375 | .600 | .002 |
| 防御性动机 | <--- | AI威胁感知 | .378 | .240 | .534 | .002 |
| 防御性动机 | <--- | AI自我效能 | .140 | -.028 | .314 | .100 |
| 使用行为 | <--- | 防御性动机 | .394 | .239 | .547 | .002 |
| 使用行为 | <--- | AI自我效能 | .482 | .329 | .646 | .002 |
| AA1 | <--- | AI威胁感知 | 1.000 | 1.000 | 1.000 | ... |
| AA2 | <--- | AI威胁感知 | .950 | .853 | 1.058 | .002 |
| AA3 | <--- | AI威胁感知 | .926 | .819 | 1.037 | .002 |
| AA4 | <--- | AI威胁感知 | .936 | .821 | 1.055 | .002 |
| AA5 | <--- | AI威胁感知 | .925 | .815 | 1.053 | .002 |
| DM1 | <--- | 防御性动机 | 1.000 | 1.000 | 1.000 | ... |
| DM2 | <--- | 防御性动机 | 1.069 | .897 | 1.301 | .002 |
| DM3 | <--- | 防御性动机 | 1.082 | .919 | 1.305 | .002 |
| DM5 | <--- | 防御性动机 | 1.012 | .833 | 1.252 | .002 |
| SE1 | <--- | AI自我效能 | 1.000 | 1.000 | 1.000 | ... |
| SE2 | <--- | AI自我效能 | 1.082 | .899 | 1.272 | .002 |
| SE3 | <--- | AI自我效能 | .982 | .828 | 1.160 | .002 |
| SE4 | <--- | AI自我效能 | 1.139 | .979 | 1.334 | .002 |
| SE5 | <--- | AI自我效能 | 1.139 | .964 | 1.323 | .002 |
| AB1 | <--- | 使用行为 | 1.000 | 1.000 | 1.000 | ... |
| AB2 | <--- | 使用行为 | .929 | .805 | 1.072 | .002 |
| AB3 | <--- | 使用行为 | 1.044 | .928 | 1.208 | .002 |
| AB5 | <--- | 使用行为 | .968 | .833 | 1.127 | .002 |

##### Standardized Regression Weights: (Middle - H3a vs H3)

| Parameter | | | Estimate | Lower | Upper | P |
| --- | --- | --- | --- | --- | --- | --- |
| AI自我效能 | <--- | AI威胁感知 | .579 | .484 | .660 | .002 |
| 防御性动机 | <--- | AI威胁感知 | .445 | .286 | .602 | .002 |
| 防御性动机 | <--- | AI自我效能 | .139 | -.029 | .298 | .100 |
| 使用行为 | <--- | 防御性动机 | .353 | .217 | .475 | .002 |
| 使用行为 | <--- | AI自我效能 | .427 | .297 | .545 | .002 |
| AA1 | <--- | AI威胁感知 | .824 | .767 | .872 | .002 |
| AA2 | <--- | AI威胁感知 | .789 | .737 | .836 | .002 |
| AA3 | <--- | AI威胁感知 | .748 | .685 | .800 | .002 |
| AA4 | <--- | AI威胁感知 | .766 | .704 | .819 | .002 |
| AA5 | <--- | AI威胁感知 | .783 | .721 | .843 | .002 |
| DM1 | <--- | 防御性动机 | .688 | .595 | .764 | .002 |
| DM2 | <--- | 防御性动机 | .747 | .676 | .807 | .002 |
| DM3 | <--- | 防御性动机 | .761 | .692 | .824 | .002 |
| DM5 | <--- | 防御性动机 | .702 | .623 | .771 | .002 |
| SE1 | <--- | AI自我效能 | .679 | .603 | .749 | .002 |
| SE2 | <--- | AI自我效能 | .716 | .634 | .788 | .002 |
| SE3 | <--- | AI自我效能 | .674 | .591 | .746 | .002 |
| SE4 | <--- | AI自我效能 | .790 | .733 | .841 | .002 |
| SE5 | <--- | AI自我效能 | .801 | .742 | .845 | .002 |
| AB1 | <--- | 使用行为 | .770 | .696 | .824 | .002 |
| AB2 | <--- | 使用行为 | .718 | .643 | .780 | .002 |
| AB3 | <--- | 使用行为 | .812 | .754 | .860 | .002 |
| AB5 | <--- | 使用行为 | .741 | .668 | .803 | .002 |

##### Variances: (Middle - H3a vs H3)

| Parameter | | | Estimate | Lower | Upper | P |
| --- | --- | --- | --- | --- | --- | --- |
| AI威胁感知 |  |  | 2.209 | 1.769 | 2.633 | .002 |
| e21 |  |  | 1.032 | .761 | 1.363 | .002 |
| e23 |  |  | 1.133 | .771 | 1.518 | .002 |
| e22 |  |  | 1.134 | .824 | 1.438 | .002 |
| e1 |  |  | 1.045 | .802 | 1.298 | .002 |
| e2 |  |  | 1.208 | .982 | 1.434 | .002 |
| e3 |  |  | 1.491 | 1.236 | 1.735 | .002 |
| e4 |  |  | 1.360 | 1.107 | 1.635 | .002 |
| e5 |  |  | 1.197 | .908 | 1.476 | .002 |
| e6 |  |  | 1.775 | 1.409 | 2.147 | .002 |
| e8 |  |  | 1.442 | 1.135 | 1.753 | .002 |
| e9 |  |  | 1.358 | 1.043 | 1.627 | .002 |
| e10 |  |  | 1.678 | 1.338 | 1.982 | .002 |
| e11 |  |  | 1.814 | 1.498 | 2.112 | .002 |
| e12 |  |  | 1.732 | 1.310 | 2.121 | .002 |
| e13 |  |  | 1.803 | 1.482 | 2.120 | .002 |
| e14 |  |  | 1.218 | .948 | 1.460 | .002 |
| e15 |  |  | 1.123 | .901 | 1.399 | .002 |
| e16 |  |  | 1.362 | 1.069 | 1.665 | .002 |
| e17 |  |  | 1.606 | 1.290 | 1.950 | .002 |
| e18 |  |  | 1.115 | .856 | 1.359 | .002 |
| e20 |  |  | 1.522 | 1.200 | 1.838 | .002 |

##### Squared Multiple Correlations: (Middle - H3a vs H3)

| Parameter | | | Estimate | Lower | Upper | P |
| --- | --- | --- | --- | --- | --- | --- |
| AI自我效能 |  |  | .336 | .234 | .435 | .002 |
| 防御性动机 |  |  | .289 | .183 | .417 | .002 |
| 使用行为 |  |  | .427 | .313 | .549 | .002 |
| AB5 |  |  | .549 | .446 | .645 | .002 |
| AB3 |  |  | .660 | .569 | .739 | .002 |
| AB2 |  |  | .516 | .414 | .608 | .002 |
| AB1 |  |  | .592 | .484 | .680 | .002 |
| SE5 |  |  | .642 | .550 | .714 | .002 |
| SE4 |  |  | .623 | .537 | .708 | .002 |
| SE3 |  |  | .454 | .349 | .556 | .002 |
| SE2 |  |  | .512 | .402 | .620 | .002 |
| SE1 |  |  | .461 | .364 | .560 | .002 |
| DM5 |  |  | .493 | .388 | .594 | .002 |
| DM3 |  |  | .579 | .479 | .679 | .002 |
| DM2 |  |  | .558 | .457 | .651 | .002 |
| DM1 |  |  | .473 | .354 | .584 | .002 |
| AA5 |  |  | .612 | .521 | .711 | .002 |
| AA4 |  |  | .587 | .495 | .670 | .002 |
| AA3 |  |  | .559 | .469 | .641 | .002 |
| AA2 |  |  | .623 | .543 | .698 | .002 |
| AA1 |  |  | .679 | .589 | .761 | .002 |

##### User-defined estimands: (Middle - H3a vs H3)

| Parameter | | | Estimate | Lower | Upper | P |
| --- | --- | --- | --- | --- | --- | --- |
| diff1 |  |  | .350 | .155 | .535 | .002 |
| diff2 |  |  | .242 | .034 | .453 | .022 |
| diff3 |  |  | .346 | .108 | .572 | .003 |
| diff4 |  |  | .258 | .042 | .466 | .014 |
| diff5 |  |  | .004 | -.244 | .229 | .992 |

##### Bias-corrected percentile method (Middle - H3a vs H3)

##### 95% confidence intervals (bias-corrected percentile method)

##### Scalar Estimates (Middle - H3a vs H3)

##### Regression Weights: (Middle - H3a vs H3)

| Parameter | | | Estimate | Lower | Upper | P |
| --- | --- | --- | --- | --- | --- | --- |
| AI自我效能 | <--- | AI威胁感知 | .486 | .386 | .608 | .001 |
| 防御性动机 | <--- | AI威胁感知 | .378 | .237 | .528 | .002 |
| 防御性动机 | <--- | AI自我效能 | .140 | -.036 | .307 | .103 |
| 使用行为 | <--- | 防御性动机 | .394 | .240 | .549 | .002 |
| 使用行为 | <--- | AI自我效能 | .482 | .334 | .648 | .002 |
| AA1 | <--- | AI威胁感知 | 1.000 | 1.000 | 1.000 | ... |
| AA2 | <--- | AI威胁感知 | .950 | .848 | 1.053 | .003 |
| AA3 | <--- | AI威胁感知 | .926 | .824 | 1.046 | .001 |
| AA4 | <--- | AI威胁感知 | .936 | .826 | 1.060 | .001 |
| AA5 | <--- | AI威胁感知 | .925 | .811 | 1.048 | .003 |
| DM1 | <--- | 防御性动机 | 1.000 | 1.000 | 1.000 | ... |
| DM2 | <--- | 防御性动机 | 1.069 | .893 | 1.297 | .002 |
| DM3 | <--- | 防御性动机 | 1.082 | .916 | 1.295 | .002 |
| DM5 | <--- | 防御性动机 | 1.012 | .833 | 1.252 | .002 |
| SE1 | <--- | AI自我效能 | 1.000 | 1.000 | 1.000 | ... |
| SE2 | <--- | AI自我效能 | 1.082 | .925 | 1.286 | .001 |
| SE3 | <--- | AI自我效能 | .982 | .826 | 1.156 | .002 |
| SE4 | <--- | AI自我效能 | 1.139 | .987 | 1.347 | .001 |
| SE5 | <--- | AI自我效能 | 1.139 | .965 | 1.323 | .002 |
| AB1 | <--- | 使用行为 | 1.000 | 1.000 | 1.000 | ... |
| AB2 | <--- | 使用行为 | .929 | .802 | 1.071 | .002 |
| AB3 | <--- | 使用行为 | 1.044 | .928 | 1.208 | .002 |
| AB5 | <--- | 使用行为 | .968 | .838 | 1.136 | .001 |

##### Standardized Regression Weights: (Middle - H3a vs H3)

| Parameter | | | Estimate | Lower | Upper | P |
| --- | --- | --- | --- | --- | --- | --- |
| AI自我效能 | <--- | AI威胁感知 | .579 | .487 | .664 | .001 |
| 防御性动机 | <--- | AI威胁感知 | .445 | .268 | .583 | .004 |
| 防御性动机 | <--- | AI自我效能 | .139 | -.041 | .293 | .121 |
| 使用行为 | <--- | 防御性动机 | .353 | .214 | .474 | .002 |
| 使用行为 | <--- | AI自我效能 | .427 | .290 | .540 | .003 |
| AA1 | <--- | AI威胁感知 | .824 | .766 | .869 | .003 |
| AA2 | <--- | AI威胁感知 | .789 | .735 | .832 | .003 |
| AA3 | <--- | AI威胁感知 | .748 | .685 | .803 | .002 |
| AA4 | <--- | AI威胁感知 | .766 | .709 | .820 | .001 |
| AA5 | <--- | AI威胁感知 | .783 | .716 | .839 | .003 |
| DM1 | <--- | 防御性动机 | .688 | .595 | .764 | .002 |
| DM2 | <--- | 防御性动机 | .747 | .677 | .807 | .002 |
| DM3 | <--- | 防御性动机 | .761 | .692 | .824 | .002 |
| DM5 | <--- | 防御性动机 | .702 | .627 | .776 | .002 |
| SE1 | <--- | AI自我效能 | .679 | .602 | .747 | .003 |
| SE2 | <--- | AI自我效能 | .716 | .632 | .785 | .002 |
| SE3 | <--- | AI自我效能 | .674 | .586 | .743 | .003 |
| SE4 | <--- | AI自我效能 | .790 | .731 | .840 | .002 |
| SE5 | <--- | AI自我效能 | .801 | .744 | .847 | .002 |
| AB1 | <--- | 使用行为 | .770 | .698 | .825 | .002 |
| AB2 | <--- | 使用行为 | .718 | .647 | .784 | .002 |
| AB3 | <--- | 使用行为 | .812 | .750 | .858 | .003 |
| AB5 | <--- | 使用行为 | .741 | .667 | .800 | .002 |

##### Variances: (Middle - H3a vs H3)

| Parameter | | | Estimate | Lower | Upper | P |
| --- | --- | --- | --- | --- | --- | --- |
| AI威胁感知 |  |  | 2.209 | 1.780 | 2.665 | .002 |
| e21 |  |  | 1.032 | .765 | 1.366 | .002 |
| e23 |  |  | 1.133 | .815 | 1.573 | .001 |
| e22 |  |  | 1.134 | .849 | 1.463 | .001 |
| e1 |  |  | 1.045 | .818 | 1.305 | .001 |
| e2 |  |  | 1.208 | 1.014 | 1.508 | .001 |
| e3 |  |  | 1.491 | 1.266 | 1.771 | .001 |
| e4 |  |  | 1.360 | 1.129 | 1.654 | .001 |
| e5 |  |  | 1.197 | .951 | 1.530 | .001 |
| e6 |  |  | 1.775 | 1.436 | 2.180 | .001 |
| e8 |  |  | 1.442 | 1.172 | 1.795 | .001 |
| e9 |  |  | 1.358 | 1.051 | 1.641 | .002 |
| e10 |  |  | 1.678 | 1.343 | 1.985 | .002 |
| e11 |  |  | 1.814 | 1.532 | 2.140 | .001 |
| e12 |  |  | 1.732 | 1.362 | 2.160 | .001 |
| e13 |  |  | 1.803 | 1.507 | 2.172 | .001 |
| e14 |  |  | 1.218 | .975 | 1.500 | .001 |
| e15 |  |  | 1.123 | .906 | 1.411 | .002 |
| e16 |  |  | 1.362 | 1.077 | 1.677 | .002 |
| e17 |  |  | 1.606 | 1.302 | 1.965 | .001 |
| e18 |  |  | 1.115 | .885 | 1.389 | .001 |
| e20 |  |  | 1.522 | 1.255 | 1.891 | .001 |

##### Squared Multiple Correlations: (Middle - H3a vs H3)

| Parameter | | | Estimate | Lower | Upper | P |
| --- | --- | --- | --- | --- | --- | --- |
| AI自我效能 |  |  | .336 | .238 | .441 | .001 |
| 防御性动机 |  |  | .289 | .163 | .392 | .006 |
| 使用行为 |  |  | .427 | .304 | .542 | .003 |
| AB5 |  |  | .549 | .444 | .640 | .002 |
| AB3 |  |  | .660 | .562 | .735 | .003 |
| AB2 |  |  | .516 | .418 | .614 | .002 |
| AB1 |  |  | .592 | .487 | .680 | .002 |
| SE5 |  |  | .642 | .553 | .717 | .002 |
| SE4 |  |  | .623 | .534 | .706 | .002 |
| SE3 |  |  | .454 | .344 | .551 | .003 |
| SE2 |  |  | .512 | .400 | .616 | .002 |
| SE1 |  |  | .461 | .362 | .558 | .003 |
| DM5 |  |  | .493 | .393 | .602 | .002 |
| DM3 |  |  | .579 | .479 | .679 | .002 |
| DM2 |  |  | .558 | .459 | .652 | .002 |
| DM1 |  |  | .473 | .354 | .584 | .002 |
| AA5 |  |  | .612 | .512 | .703 | .003 |
| AA4 |  |  | .587 | .502 | .672 | .001 |
| AA3 |  |  | .559 | .470 | .645 | .002 |
| AA2 |  |  | .623 | .540 | .691 | .003 |
| AA1 |  |  | .679 | .587 | .755 | .003 |

##### User-defined estimands: (Middle - H3a vs H3)

| Parameter | | | Estimate | Lower | Upper | P |
| --- | --- | --- | --- | --- | --- | --- |
| diff1 |  |  | .350 | .156 | .536 | .002 |
| diff2 |  |  | .242 | .044 | .468 | .017 |
| diff3 |  |  | .346 | .109 | .573 | .003 |
| diff4 |  |  | .258 | .059 | .480 | .008 |
| diff5 |  |  | .004 | -.237 | .232 | .952 |

##### others (others - H3a vs H3)

##### Estimates (others - H3a vs H3)

##### Scalar Estimates (others - H3a vs H3)

##### Maximum Likelihood Estimates

##### Regression Weights: (others - H3a vs H3)

|  |  |  | Estimate | S.E. | C.R. | P | Label |
| --- | --- | --- | --- | --- | --- | --- | --- |
| AI自我效能 | <--- | AI威胁感知 | .136 | .069 | 1.971 | .049 | H1 |
| 防御性动机 | <--- | AI威胁感知 | .378 | .116 | 3.249 | .001 | H2 |
| 防御性动机 | <--- | AI自我效能 | -.187 | .188 | -.994 | .320 | H5 |
| 使用行为 | <--- | 防御性动机 | .405 | .109 | 3.730 | \*\*\* | H4 |
| 使用行为 | <--- | AI自我效能 | .482 | .076 | 6.373 | \*\*\* | H3a |
| AA1 | <--- | AI威胁感知 | 1.000 |  |  |  |  |
| AA2 | <--- | AI威胁感知 | .951 | .115 | 8.266 | \*\*\* |  |
| AA3 | <--- | AI威胁感知 | .893 | .110 | 8.083 | \*\*\* |  |
| AA4 | <--- | AI威胁感知 | .870 | .115 | 7.536 | \*\*\* |  |
| AA5 | <--- | AI威胁感知 | .995 | .117 | 8.516 | \*\*\* |  |
| DM1 | <--- | 防御性动机 | 1.000 |  |  |  |  |
| DM2 | <--- | 防御性动机 | .895 | .131 | 6.828 | \*\*\* |  |
| DM3 | <--- | 防御性动机 | .924 | .131 | 7.029 | \*\*\* |  |
| DM5 | <--- | 防御性动机 | .920 | .131 | 7.008 | \*\*\* |  |
| SE1 | <--- | AI自我效能 | 1.000 |  |  |  |  |
| SE2 | <--- | AI自我效能 | 1.468 | .287 | 5.120 | \*\*\* |  |
| SE3 | <--- | AI自我效能 | 1.601 | .296 | 5.399 | \*\*\* |  |
| SE4 | <--- | AI自我效能 | 1.829 | .328 | 5.579 | \*\*\* |  |
| SE5 | <--- | AI自我效能 | 1.585 | .294 | 5.385 | \*\*\* |  |
| AB1 | <--- | 使用行为 | 1.000 |  |  |  |  |
| AB2 | <--- | 使用行为 | 1.124 | .148 | 7.585 | \*\*\* |  |
| AB3 | <--- | 使用行为 | 1.102 | .149 | 7.416 | \*\*\* |  |
| AB5 | <--- | 使用行为 | .919 | .145 | 6.356 | \*\*\* |  |

##### Standardized Regression Weights: (others - H3a vs H3)

|  |  |  | Estimate |
| --- | --- | --- | --- |
| AI自我效能 | <--- | AI威胁感知 | .230 |
| 防御性动机 | <--- | AI威胁感知 | .388 |
| 防御性动机 | <--- | AI自我效能 | -.113 |
| 使用行为 | <--- | 防御性动机 | .439 |
| 使用行为 | <--- | AI自我效能 | .318 |
| AA1 | <--- | AI威胁感知 | .807 |
| AA2 | <--- | AI威胁感知 | .767 |
| AA3 | <--- | AI威胁感知 | .753 |
| AA4 | <--- | AI威胁感知 | .710 |
| AA5 | <--- | AI威胁感知 | .787 |
| DM1 | <--- | 防御性动机 | .770 |
| DM2 | <--- | 防御性动机 | .715 |
| DM3 | <--- | 防御性动机 | .738 |
| DM5 | <--- | 防御性动机 | .735 |
| SE1 | <--- | AI自我效能 | .507 |
| SE2 | <--- | AI自我效能 | .702 |
| SE3 | <--- | AI自我效能 | .775 |
| SE4 | <--- | AI自我效能 | .833 |
| SE5 | <--- | AI自我效能 | .771 |
| AB1 | <--- | 使用行为 | .733 |
| AB2 | <--- | 使用行为 | .809 |
| AB3 | <--- | 使用行为 | .785 |
| AB5 | <--- | 使用行为 | .667 |

##### Variances: (others - H3a vs H3)

|  |  |  | Estimate | S.E. | C.R. | P | Label |
| --- | --- | --- | --- | --- | --- | --- | --- |
| AI威胁感知 |  |  | 2.140 | .449 | 4.764 | \*\*\* |  |
| e21 |  |  | .708 | .250 | 2.834 | .005 |  |
| e23 |  |  | 1.733 | .414 | 4.181 | \*\*\* |  |
| e22 |  |  | 1.228 | .318 | 3.867 | \*\*\* |  |
| e1 |  |  | 1.142 | .213 | 5.352 | \*\*\* |  |
| e2 |  |  | 1.355 | .234 | 5.802 | \*\*\* |  |
| e3 |  |  | 1.303 | .220 | 5.925 | \*\*\* |  |
| e4 |  |  | 1.592 | .256 | 6.219 | \*\*\* |  |
| e5 |  |  | 1.305 | .233 | 5.605 | \*\*\* |  |
| e6 |  |  | 1.386 | .270 | 5.126 | \*\*\* |  |
| e8 |  |  | 1.550 | .270 | 5.732 | \*\*\* |  |
| e9 |  |  | 1.449 | .263 | 5.514 | \*\*\* |  |
| e10 |  |  | 1.456 | .263 | 5.539 | \*\*\* |  |
| e11 |  |  | 2.162 | .315 | 6.861 | \*\*\* |  |
| e12 |  |  | 1.656 | .269 | 6.150 | \*\*\* |  |
| e13 |  |  | 1.275 | .230 | 5.537 | \*\*\* |  |
| e14 |  |  | 1.099 | .235 | 4.672 | \*\*\* |  |
| e15 |  |  | 1.283 | .230 | 5.582 | \*\*\* |  |
| e16 |  |  | 1.483 | .259 | 5.727 | \*\*\* |  |
| e17 |  |  | 1.149 | .242 | 4.739 | \*\*\* |  |
| e18 |  |  | 1.299 | .255 | 5.104 | \*\*\* |  |
| e20 |  |  | 1.811 | .292 | 6.196 | \*\*\* |  |

##### Squared Multiple Correlations: (others - H3a vs H3)

|  |  |  | Estimate |
| --- | --- | --- | --- |
| AI自我效能 |  |  | .053 |
| 防御性动机 |  |  | .144 |
| 使用行为 |  |  | .287 |
| AB5 |  |  | .445 |
| AB3 |  |  | .617 |
| AB2 |  |  | .655 |
| AB1 |  |  | .537 |
| SE5 |  |  | .594 |
| SE4 |  |  | .695 |
| SE3 |  |  | .600 |
| SE2 |  |  | .493 |
| SE1 |  |  | .257 |
| DM5 |  |  | .540 |
| DM3 |  |  | .544 |
| DM2 |  |  | .511 |
| DM1 |  |  | .593 |
| AA5 |  |  | .619 |
| AA4 |  |  | .505 |
| AA3 |  |  | .567 |
| AA2 |  |  | .588 |
| AA1 |  |  | .652 |

##### User-defined estimands: (others - H3a vs H3)

|  |  |  |  |
| --- | --- | --- | --- |
| diff1 |  |  | .350 |
| diff2 |  |  | .242 |
| diff3 |  |  | .346 |
| diff4 |  |  | .258 |
| diff5 |  |  | .004 |

##### Modification Indices (others - H3a vs H3)

##### Covariances: (others - H3a vs H3)

|  |  |  | M.I. | Par Change |
| --- | --- | --- | --- | --- |
| e20 | <--> | e21 | 5.343 | .294 |
| e17 | <--> | e23 | 5.440 | .424 |
| e14 | <--> | AI威胁感知 | 4.784 | .416 |
| e11 | <--> | e20 | 6.509 | -.538 |
| e10 | <--> | e20 | 7.875 | -.527 |
| e9 | <--> | e13 | 5.203 | -.376 |
| e5 | <--> | e16 | 4.158 | -.339 |
| e5 | <--> | e8 | 5.411 | .395 |
| e3 | <--> | e21 | 4.871 | .243 |

##### Variances: (others - H3a vs H3)

|  |  |  | M.I. | Par Change |
| --- | --- | --- | --- | --- |

##### Regression Weights: (others - H3a vs H3)

|  |  |  | M.I. | Par Change |
| --- | --- | --- | --- | --- |
| AB5 | <--- | AI自我效能 | 5.704 | .415 |
| AB5 | <--- | SE4 | 5.399 | .172 |
| AB5 | <--- | SE3 | 8.095 | .223 |
| AB5 | <--- | SE2 | 4.024 | .156 |
| AB5 | <--- | DM5 | 5.540 | -.185 |
| AB2 | <--- | AI自我效能 | 4.024 | -.307 |
| AB2 | <--- | 防御性动机 | 4.376 | .198 |
| AB2 | <--- | SE5 | 4.103 | -.141 |
| AB2 | <--- | SE3 | 4.847 | -.152 |
| AB2 | <--- | DM5 | 4.808 | .152 |
| AB1 | <--- | AA5 | 5.664 | -.168 |
| SE4 | <--- | AI威胁感知 | 4.784 | .195 |
| SE4 | <--- | DM3 | 4.671 | .148 |
| SE4 | <--- | AA4 | 5.244 | .156 |
| SE4 | <--- | AA3 | 6.246 | .176 |
| SE4 | <--- | AA2 | 5.157 | .153 |
| SE3 | <--- | AA2 | 4.124 | -.138 |
| DM5 | <--- | AB5 | 4.381 | -.152 |
| DM3 | <--- | SE4 | 5.585 | .163 |
| AA3 | <--- | AI自我效能 | 4.555 | .322 |
| AA3 | <--- | SE4 | 5.715 | .153 |

##### Bootstrap (others - H3a vs H3)

##### Bootstrap standard errors (others - H3a vs H3)

##### Scalar Estimates (others - H3a vs H3)

##### Regression Weights: (others - H3a vs H3)

| Parameter | | | SE | SE-SE | Mean | Bias | SE-Bias |
| --- | --- | --- | --- | --- | --- | --- | --- |
| AI自我效能 | <--- | AI威胁感知 | .076 | .002 | .139 | .003 | .002 |
| 防御性动机 | <--- | AI威胁感知 | .112 | .003 | .372 | -.005 | .004 |
| 防御性动机 | <--- | AI自我效能 | .210 | .005 | -.191 | -.004 | .007 |
| 使用行为 | <--- | 防御性动机 | .101 | .002 | .401 | -.004 | .003 |
| 使用行为 | <--- | AI自我效能 | .079 | .002 | .483 | .000 | .003 |
| AA1 | <--- | AI威胁感知 | .000 | .000 | 1.000 | .000 | .000 |
| AA2 | <--- | AI威胁感知 | .104 | .002 | .950 | -.001 | .003 |
| AA3 | <--- | AI威胁感知 | .076 | .002 | .895 | .003 | .002 |
| AA4 | <--- | AI威胁感知 | .116 | .003 | .866 | -.005 | .004 |
| AA5 | <--- | AI威胁感知 | .113 | .003 | 1.000 | .005 | .004 |
| DM1 | <--- | 防御性动机 | .000 | .000 | 1.000 | .000 | .000 |
| DM2 | <--- | 防御性动机 | .135 | .003 | .901 | .006 | .004 |
| DM3 | <--- | 防御性动机 | .143 | .003 | .926 | .002 | .005 |
| DM5 | <--- | 防御性动机 | .108 | .002 | .925 | .005 | .003 |
| SE1 | <--- | AI自我效能 | .000 | .000 | 1.000 | .000 | .000 |
| SE2 | <--- | AI自我效能 | .268 | .006 | 1.492 | .025 | .008 |
| SE3 | <--- | AI自我效能 | .300 | .007 | 1.640 | .039 | .009 |
| SE4 | <--- | AI自我效能 | .353 | .008 | 1.874 | .045 | .011 |
| SE5 | <--- | AI自我效能 | .311 | .007 | 1.626 | .041 | .010 |
| AB1 | <--- | 使用行为 | .000 | .000 | 1.000 | .000 | .000 |
| AB2 | <--- | 使用行为 | .137 | .003 | 1.135 | .011 | .004 |
| AB3 | <--- | 使用行为 | .146 | .003 | 1.113 | .011 | .005 |
| AB5 | <--- | 使用行为 | .139 | .003 | .929 | .010 | .004 |

##### Standardized Regression Weights: (others - H3a vs H3)

| Parameter | | | SE | SE-SE | Mean | Bias | SE-Bias |
| --- | --- | --- | --- | --- | --- | --- | --- |
| AI自我效能 | <--- | AI威胁感知 | .117 | .003 | .231 | .001 | .004 |
| 防御性动机 | <--- | AI威胁感知 | .104 | .002 | .379 | -.009 | .003 |
| 防御性动机 | <--- | AI自我效能 | .117 | .003 | -.112 | .002 | .004 |
| 使用行为 | <--- | 防御性动机 | .098 | .002 | .435 | -.004 | .003 |
| 使用行为 | <--- | AI自我效能 | .060 | .001 | .318 | .000 | .002 |
| AA1 | <--- | AI威胁感知 | .048 | .001 | .806 | -.001 | .002 |
| AA2 | <--- | AI威胁感知 | .058 | .001 | .762 | -.005 | .002 |
| AA3 | <--- | AI威胁感知 | .053 | .001 | .753 | .000 | .002 |
| AA4 | <--- | AI威胁感知 | .068 | .002 | .701 | -.009 | .002 |
| AA5 | <--- | AI威胁感知 | .049 | .001 | .785 | -.002 | .002 |
| DM1 | <--- | 防御性动机 | .058 | .001 | .771 | .000 | .002 |
| DM2 | <--- | 防御性动机 | .068 | .002 | .713 | -.002 | .002 |
| DM3 | <--- | 防御性动机 | .060 | .001 | .733 | -.005 | .002 |
| DM5 | <--- | 防御性动机 | .059 | .001 | .735 | -.001 | .002 |
| SE1 | <--- | AI自我效能 | .076 | .002 | .507 | .001 | .002 |
| SE2 | <--- | AI自我效能 | .063 | .001 | .698 | -.004 | .002 |
| SE3 | <--- | AI自我效能 | .046 | .001 | .775 | .000 | .001 |
| SE4 | <--- | AI自我效能 | .052 | .001 | .832 | -.001 | .002 |
| SE5 | <--- | AI自我效能 | .061 | .001 | .770 | -.001 | .002 |
| AB1 | <--- | 使用行为 | .054 | .001 | .730 | -.003 | .002 |
| AB2 | <--- | 使用行为 | .058 | .001 | .808 | -.001 | .002 |
| AB3 | <--- | 使用行为 | .056 | .001 | .786 | .000 | .002 |
| AB5 | <--- | 使用行为 | .065 | .001 | .666 | -.002 | .002 |

##### Variances: (others - H3a vs H3)

| Parameter | | | SE | SE-SE | Mean | Bias | SE-Bias |
| --- | --- | --- | --- | --- | --- | --- | --- |
| AI威胁感知 |  |  | .385 | .009 | 2.128 | -.012 | .012 |
| e21 |  |  | .234 | .005 | .711 | .003 | .007 |
| e23 |  |  | .392 | .009 | 1.710 | -.023 | .012 |
| e22 |  |  | .313 | .007 | 1.215 | -.012 | .010 |
| e1 |  |  | .222 | .005 | 1.117 | -.025 | .007 |
| e2 |  |  | .256 | .006 | 1.338 | -.017 | .008 |
| e3 |  |  | .233 | .005 | 1.269 | -.035 | .007 |
| e4 |  |  | .271 | .006 | 1.586 | -.006 | .009 |
| e5 |  |  | .238 | .005 | 1.280 | -.025 | .008 |
| e6 |  |  | .272 | .006 | 1.352 | -.034 | .009 |
| e8 |  |  | .314 | .007 | 1.529 | -.021 | .010 |
| e9 |  |  | .233 | .005 | 1.430 | -.019 | .007 |
| e10 |  |  | .259 | .006 | 1.432 | -.024 | .008 |
| e11 |  |  | .277 | .006 | 2.121 | -.041 | .009 |
| e12 |  |  | .253 | .006 | 1.634 | -.022 | .008 |
| e13 |  |  | .217 | .005 | 1.249 | -.027 | .007 |
| e14 |  |  | .271 | .006 | 1.077 | -.022 | .009 |
| e15 |  |  | .254 | .006 | 1.257 | -.026 | .008 |
| e16 |  |  | .227 | .005 | 1.464 | -.019 | .007 |
| e17 |  |  | .299 | .007 | 1.128 | -.021 | .009 |
| e18 |  |  | .290 | .006 | 1.264 | -.035 | .009 |
| e20 |  |  | .284 | .006 | 1.783 | -.027 | .009 |

##### Squared Multiple Correlations: (others - H3a vs H3)

| Parameter | | | SE | SE-SE | Mean | Bias | SE-Bias |
| --- | --- | --- | --- | --- | --- | --- | --- |
| AI自我效能 |  |  | .054 | .001 | .067 | .014 | .002 |
| 防御性动机 |  |  | .075 | .002 | .161 | .017 | .002 |
| 使用行为 |  |  | .085 | .002 | .294 | .007 | .003 |
| AB5 |  |  | .085 | .002 | .447 | .002 | .003 |
| AB3 |  |  | .087 | .002 | .620 | .003 | .003 |
| AB2 |  |  | .093 | .002 | .656 | .001 | .003 |
| AB1 |  |  | .078 | .002 | .536 | -.001 | .002 |
| SE5 |  |  | .092 | .002 | .596 | .002 | .003 |
| SE4 |  |  | .085 | .002 | .695 | .000 | .003 |
| SE3 |  |  | .070 | .002 | .603 | .002 | .002 |
| SE2 |  |  | .086 | .002 | .492 | -.001 | .003 |
| SE1 |  |  | .077 | .002 | .263 | .006 | .002 |
| DM5 |  |  | .085 | .002 | .543 | .003 | .003 |
| DM3 |  |  | .087 | .002 | .540 | -.004 | .003 |
| DM2 |  |  | .095 | .002 | .513 | .002 | .003 |
| DM1 |  |  | .088 | .002 | .597 | .004 | .003 |
| AA5 |  |  | .076 | .002 | .619 | .000 | .002 |
| AA4 |  |  | .094 | .002 | .497 | -.008 | .003 |
| AA3 |  |  | .079 | .002 | .570 | .003 | .003 |
| AA2 |  |  | .087 | .002 | .585 | -.004 | .003 |
| AA1 |  |  | .077 | .002 | .653 | .001 | .002 |

##### User-defined estimands: (others - H3a vs H3)

| Parameter | | | SE | SE-SE | Mean | Bias | SE-Bias |
| --- | --- | --- | --- | --- | --- | --- | --- |
| diff1 |  |  | .095 | .002 | .347 | -.003 | .003 |
| diff2 |  |  | .105 | .002 | .242 | -.001 | .003 |
| diff3 |  |  | .116 | .003 | .343 | -.003 | .004 |
| diff4 |  |  | .109 | .002 | .254 | -.004 | .003 |
| diff5 |  |  | .118 | .003 | .001 | -.003 | .004 |

##### Bootstrap Confidence (others - H3a vs H3)

##### Percentile method (others - H3a vs H3)

##### 95% confidence intervals (percentile method)

##### Scalar Estimates (others - H3a vs H3)

##### Regression Weights: (others - H3a vs H3)

| Parameter | | | Estimate | Lower | Upper | P |
| --- | --- | --- | --- | --- | --- | --- |
| AI自我效能 | <--- | AI威胁感知 | .136 | -.008 | .298 | .064 |
| 防御性动机 | <--- | AI威胁感知 | .378 | .154 | .600 | .003 |
| 防御性动机 | <--- | AI自我效能 | -.187 | -.604 | .238 | .326 |
| 使用行为 | <--- | 防御性动机 | .405 | .205 | .602 | .002 |
| 使用行为 | <--- | AI自我效能 | .482 | .329 | .646 | .002 |
| AA1 | <--- | AI威胁感知 | 1.000 | 1.000 | 1.000 | ... |
| AA2 | <--- | AI威胁感知 | .951 | .746 | 1.161 | .002 |
| AA3 | <--- | AI威胁感知 | .893 | .747 | 1.051 | .002 |
| AA4 | <--- | AI威胁感知 | .870 | .652 | 1.103 | .002 |
| AA5 | <--- | AI威胁感知 | .995 | .800 | 1.234 | .002 |
| DM1 | <--- | 防御性动机 | 1.000 | 1.000 | 1.000 | ... |
| DM2 | <--- | 防御性动机 | .895 | .677 | 1.191 | .002 |
| DM3 | <--- | 防御性动机 | .924 | .660 | 1.218 | .002 |
| DM5 | <--- | 防御性动机 | .920 | .729 | 1.145 | .002 |
| SE1 | <--- | AI自我效能 | 1.000 | 1.000 | 1.000 | ... |
| SE2 | <--- | AI自我效能 | 1.468 | 1.055 | 2.104 | .002 |
| SE3 | <--- | AI自我效能 | 1.601 | 1.180 | 2.327 | .002 |
| SE4 | <--- | AI自我效能 | 1.829 | 1.346 | 2.728 | .002 |
| SE5 | <--- | AI自我效能 | 1.585 | 1.131 | 2.353 | .002 |
| AB1 | <--- | 使用行为 | 1.000 | 1.000 | 1.000 | ... |
| AB2 | <--- | 使用行为 | 1.124 | .902 | 1.425 | .002 |
| AB3 | <--- | 使用行为 | 1.102 | .867 | 1.443 | .002 |
| AB5 | <--- | 使用行为 | .919 | .675 | 1.219 | .002 |

##### Standardized Regression Weights: (others - H3a vs H3)

| Parameter | | | Estimate | Lower | Upper | P |
| --- | --- | --- | --- | --- | --- | --- |
| AI自我效能 | <--- | AI威胁感知 | .230 | -.016 | .449 | .064 |
| 防御性动机 | <--- | AI威胁感知 | .388 | .161 | .569 | .003 |
| 防御性动机 | <--- | AI自我效能 | -.113 | -.327 | .125 | .326 |
| 使用行为 | <--- | 防御性动机 | .439 | .232 | .609 | .002 |
| 使用行为 | <--- | AI自我效能 | .318 | .204 | .440 | .002 |
| AA1 | <--- | AI威胁感知 | .807 | .703 | .891 | .002 |
| AA2 | <--- | AI威胁感知 | .767 | .630 | .861 | .002 |
| AA3 | <--- | AI威胁感知 | .753 | .645 | .845 | .002 |
| AA4 | <--- | AI威胁感知 | .710 | .549 | .821 | .002 |
| AA5 | <--- | AI威胁感知 | .787 | .677 | .868 | .002 |
| DM1 | <--- | 防御性动机 | .770 | .654 | .874 | .002 |
| DM2 | <--- | 防御性动机 | .715 | .577 | .832 | .002 |
| DM3 | <--- | 防御性动机 | .738 | .600 | .838 | .002 |
| DM5 | <--- | 防御性动机 | .735 | .603 | .839 | .002 |
| SE1 | <--- | AI自我效能 | .507 | .359 | .647 | .002 |
| SE2 | <--- | AI自我效能 | .702 | .559 | .812 | .002 |
| SE3 | <--- | AI自我效能 | .775 | .680 | .857 | .002 |
| SE4 | <--- | AI自我效能 | .833 | .721 | .921 | .002 |
| SE5 | <--- | AI自我效能 | .771 | .633 | .878 | .002 |
| AB1 | <--- | 使用行为 | .733 | .612 | .830 | .002 |
| AB2 | <--- | 使用行为 | .809 | .683 | .914 | .002 |
| AB3 | <--- | 使用行为 | .785 | .666 | .880 | .002 |
| AB5 | <--- | 使用行为 | .667 | .529 | .780 | .002 |

##### Variances: (others - H3a vs H3)

| Parameter | | | Estimate | Lower | Upper | P |
| --- | --- | --- | --- | --- | --- | --- |
| AI威胁感知 |  |  | 2.140 | 1.412 | 2.911 | .002 |
| e21 |  |  | .708 | .309 | 1.208 | .002 |
| e23 |  |  | 1.733 | 1.003 | 2.539 | .002 |
| e22 |  |  | 1.228 | .697 | 1.946 | .002 |
| e1 |  |  | 1.142 | .699 | 1.585 | .002 |
| e2 |  |  | 1.355 | .878 | 1.891 | .002 |
| e3 |  |  | 1.303 | .843 | 1.767 | .002 |
| e4 |  |  | 1.592 | 1.080 | 2.147 | .002 |
| e5 |  |  | 1.305 | .830 | 1.785 | .002 |
| e6 |  |  | 1.386 | .812 | 1.885 | .002 |
| e8 |  |  | 1.550 | .945 | 2.196 | .002 |
| e9 |  |  | 1.449 | .972 | 1.875 | .002 |
| e10 |  |  | 1.456 | .961 | 1.969 | .002 |
| e11 |  |  | 2.162 | 1.598 | 2.667 | .002 |
| e12 |  |  | 1.656 | 1.178 | 2.156 | .002 |
| e13 |  |  | 1.275 | .840 | 1.682 | .002 |
| e14 |  |  | 1.099 | .542 | 1.589 | .002 |
| e15 |  |  | 1.283 | .752 | 1.783 | .002 |
| e16 |  |  | 1.483 | 1.001 | 1.922 | .002 |
| e17 |  |  | 1.149 | .540 | 1.722 | .002 |
| e18 |  |  | 1.299 | .740 | 1.831 | .002 |
| e20 |  |  | 1.811 | 1.258 | 2.353 | .002 |

##### Squared Multiple Correlations: (others - H3a vs H3)

| Parameter | | | Estimate | Lower | Upper | P |
| --- | --- | --- | --- | --- | --- | --- |
| AI自我效能 |  |  | .053 | .001 | .201 | .002 |
| 防御性动机 |  |  | .144 | .039 | .325 | .002 |
| 使用行为 |  |  | .287 | .141 | .472 | .002 |
| AB5 |  |  | .445 | .280 | .608 | .002 |
| AB3 |  |  | .617 | .443 | .774 | .002 |
| AB2 |  |  | .655 | .466 | .835 | .002 |
| AB1 |  |  | .537 | .374 | .688 | .002 |
| SE5 |  |  | .594 | .401 | .770 | .002 |
| SE4 |  |  | .695 | .519 | .849 | .002 |
| SE3 |  |  | .600 | .463 | .734 | .002 |
| SE2 |  |  | .493 | .312 | .659 | .002 |
| SE1 |  |  | .257 | .129 | .419 | .002 |
| DM5 |  |  | .540 | .364 | .704 | .002 |
| DM3 |  |  | .544 | .360 | .702 | .002 |
| DM2 |  |  | .511 | .332 | .693 | .002 |
| DM1 |  |  | .593 | .428 | .764 | .002 |
| AA5 |  |  | .619 | .458 | .753 | .002 |
| AA4 |  |  | .505 | .301 | .674 | .002 |
| AA3 |  |  | .567 | .416 | .713 | .002 |
| AA2 |  |  | .588 | .396 | .741 | .002 |
| AA1 |  |  | .652 | .494 | .794 | .002 |

##### User-defined estimands: (others - H3a vs H3)

| Parameter | | | Estimate | Lower | Upper | P |
| --- | --- | --- | --- | --- | --- | --- |
| diff1 |  |  | .350 | .155 | .535 | .002 |
| diff2 |  |  | .242 | .034 | .453 | .022 |
| diff3 |  |  | .346 | .108 | .572 | .003 |
| diff4 |  |  | .258 | .042 | .466 | .014 |
| diff5 |  |  | .004 | -.244 | .229 | .992 |

##### Bias-corrected percentile method (others - H3a vs H3)

##### 95% confidence intervals (bias-corrected percentile method)

##### Scalar Estimates (others - H3a vs H3)

##### Regression Weights: (others - H3a vs H3)

| Parameter | | | Estimate | Lower | Upper | P |
| --- | --- | --- | --- | --- | --- | --- |
| AI自我效能 | <--- | AI威胁感知 | .136 | -.011 | .294 | .073 |
| 防御性动机 | <--- | AI威胁感知 | .378 | .184 | .635 | .001 |
| 防御性动机 | <--- | AI自我效能 | -.187 | -.606 | .227 | .309 |
| 使用行为 | <--- | 防御性动机 | .405 | .224 | .631 | .001 |
| 使用行为 | <--- | AI自我效能 | .482 | .334 | .648 | .002 |
| AA1 | <--- | AI威胁感知 | 1.000 | 1.000 | 1.000 | ... |
| AA2 | <--- | AI威胁感知 | .951 | .768 | 1.189 | .001 |
| AA3 | <--- | AI威胁感知 | .893 | .739 | 1.045 | .003 |
| AA4 | <--- | AI威胁感知 | .870 | .675 | 1.124 | .001 |
| AA5 | <--- | AI威胁感知 | .995 | .802 | 1.235 | .002 |
| DM1 | <--- | 防御性动机 | 1.000 | 1.000 | 1.000 | ... |
| DM2 | <--- | 防御性动机 | .895 | .685 | 1.210 | .001 |
| DM3 | <--- | 防御性动机 | .924 | .668 | 1.226 | .002 |
| DM5 | <--- | 防御性动机 | .920 | .730 | 1.149 | .002 |
| SE1 | <--- | AI自我效能 | 1.000 | 1.000 | 1.000 | ... |
| SE2 | <--- | AI自我效能 | 1.468 | 1.060 | 2.132 | .002 |
| SE3 | <--- | AI自我效能 | 1.601 | 1.189 | 2.375 | .002 |
| SE4 | <--- | AI自我效能 | 1.829 | 1.367 | 2.745 | .002 |
| SE5 | <--- | AI自我效能 | 1.585 | 1.126 | 2.349 | .002 |
| AB1 | <--- | 使用行为 | 1.000 | 1.000 | 1.000 | ... |
| AB2 | <--- | 使用行为 | 1.124 | .910 | 1.431 | .002 |
| AB3 | <--- | 使用行为 | 1.102 | .868 | 1.454 | .002 |
| AB5 | <--- | 使用行为 | .919 | .672 | 1.203 | .002 |

##### Standardized Regression Weights: (others - H3a vs H3)

| Parameter | | | Estimate | Lower | Upper | P |
| --- | --- | --- | --- | --- | --- | --- |
| AI自我效能 | <--- | AI威胁感知 | .230 | -.022 | .432 | .075 |
| 防御性动机 | <--- | AI威胁感知 | .388 | .186 | .581 | .001 |
| 防御性动机 | <--- | AI自我效能 | -.113 | -.329 | .125 | .319 |
| 使用行为 | <--- | 防御性动机 | .439 | .227 | .606 | .002 |
| 使用行为 | <--- | AI自我效能 | .318 | .207 | .455 | .001 |
| AA1 | <--- | AI威胁感知 | .807 | .690 | .886 | .003 |
| AA2 | <--- | AI威胁感知 | .767 | .631 | .861 | .002 |
| AA3 | <--- | AI威胁感知 | .753 | .627 | .840 | .003 |
| AA4 | <--- | AI威胁感知 | .710 | .562 | .829 | .001 |
| AA5 | <--- | AI威胁感知 | .787 | .669 | .864 | .003 |
| DM1 | <--- | 防御性动机 | .770 | .647 | .872 | .003 |
| DM2 | <--- | 防御性动机 | .715 | .574 | .832 | .002 |
| DM3 | <--- | 防御性动机 | .738 | .601 | .838 | .002 |
| DM5 | <--- | 防御性动机 | .735 | .589 | .832 | .003 |
| SE1 | <--- | AI自我效能 | .507 | .345 | .646 | .002 |
| SE2 | <--- | AI自我效能 | .702 | .559 | .811 | .002 |
| SE3 | <--- | AI自我效能 | .775 | .675 | .854 | .003 |
| SE4 | <--- | AI自我效能 | .833 | .711 | .916 | .003 |
| SE5 | <--- | AI自我效能 | .771 | .629 | .877 | .002 |
| AB1 | <--- | 使用行为 | .733 | .612 | .830 | .002 |
| AB2 | <--- | 使用行为 | .809 | .673 | .907 | .003 |
| AB3 | <--- | 使用行为 | .785 | .654 | .874 | .003 |
| AB5 | <--- | 使用行为 | .667 | .527 | .779 | .002 |

##### Variances: (others - H3a vs H3)

| Parameter | | | Estimate | Lower | Upper | P |
| --- | --- | --- | --- | --- | --- | --- |
| AI威胁感知 |  |  | 2.140 | 1.425 | 2.921 | .002 |
| e21 |  |  | .708 | .337 | 1.250 | .001 |
| e23 |  |  | 1.733 | 1.074 | 2.635 | .001 |
| e22 |  |  | 1.228 | .757 | 2.067 | .001 |
| e1 |  |  | 1.142 | .754 | 1.645 | .001 |
| e2 |  |  | 1.355 | .906 | 1.928 | .001 |
| e3 |  |  | 1.303 | .912 | 1.836 | .001 |
| e4 |  |  | 1.592 | 1.110 | 2.177 | .001 |
| e5 |  |  | 1.305 | .889 | 1.851 | .001 |
| e6 |  |  | 1.386 | .882 | 1.936 | .001 |
| e8 |  |  | 1.550 | .996 | 2.226 | .001 |
| e9 |  |  | 1.449 | .996 | 1.923 | .001 |
| e10 |  |  | 1.456 | 1.034 | 2.084 | .001 |
| e11 |  |  | 2.162 | 1.663 | 2.743 | .001 |
| e12 |  |  | 1.656 | 1.221 | 2.222 | .001 |
| e13 |  |  | 1.275 | .876 | 1.783 | .001 |
| e14 |  |  | 1.099 | .578 | 1.617 | .001 |
| e15 |  |  | 1.283 | .818 | 1.819 | .001 |
| e16 |  |  | 1.483 | 1.053 | 1.978 | .001 |
| e17 |  |  | 1.149 | .599 | 1.757 | .001 |
| e18 |  |  | 1.299 | .822 | 1.934 | .001 |
| e20 |  |  | 1.811 | 1.295 | 2.430 | .001 |

##### Squared Multiple Correlations: (others - H3a vs H3)

| Parameter | | | Estimate | Lower | Upper | P |
| --- | --- | --- | --- | --- | --- | --- |
| AI自我效能 |  |  | .053 | .000 | .187 | .003 |
| 防御性动机 |  |  | .144 | .028 | .304 | .004 |
| 使用行为 |  |  | .287 | .137 | .466 | .003 |
| AB5 |  |  | .445 | .278 | .607 | .002 |
| AB3 |  |  | .617 | .428 | .764 | .003 |
| AB2 |  |  | .655 | .452 | .823 | .003 |
| AB1 |  |  | .537 | .374 | .688 | .002 |
| SE5 |  |  | .594 | .396 | .770 | .002 |
| SE4 |  |  | .695 | .505 | .839 | .003 |
| SE3 |  |  | .600 | .456 | .729 | .003 |
| SE2 |  |  | .493 | .312 | .657 | .002 |
| SE1 |  |  | .257 | .119 | .417 | .002 |
| DM5 |  |  | .540 | .347 | .693 | .003 |
| DM3 |  |  | .544 | .361 | .702 | .002 |
| DM2 |  |  | .511 | .330 | .692 | .002 |
| DM1 |  |  | .593 | .419 | .760 | .003 |
| AA5 |  |  | .619 | .447 | .747 | .003 |
| AA4 |  |  | .505 | .316 | .686 | .001 |
| AA3 |  |  | .567 | .393 | .706 | .003 |
| AA2 |  |  | .588 | .398 | .742 | .002 |
| AA1 |  |  | .652 | .476 | .785 | .003 |

##### User-defined estimands: (others - H3a vs H3)

| Parameter | | | Estimate | Lower | Upper | P |
| --- | --- | --- | --- | --- | --- | --- |
| diff1 |  |  | .350 | .156 | .536 | .002 |
| diff2 |  |  | .242 | .044 | .468 | .017 |
| diff3 |  |  | .346 | .109 | .573 | .003 |
| diff4 |  |  | .258 | .059 | .480 | .008 |
| diff5 |  |  | .004 | -.237 | .232 | .952 |

##### Minimization History (H3a vs H3)

| Iteration |  | Negative eigenvalues | Condition # | Smallest eigenvalue | Diameter | F | NTries | Ratio |
| --- | --- | --- | --- | --- | --- | --- | --- | --- |
| 0 | e | 11 |  | -.651 | 9999.000 | 3579.647 | 0 | 9999.000 |
| 1 | e | 4 |  | -.066 | 3.408 | 1322.449 | 20 | .391 |
| 2 | e | 0 | 315.579 |  | 2.540 | 530.509 | 5 | .635 |
| 3 | e | 0 | 110.577 |  | 1.520 | 346.239 | 1 | 1.031 |
| 4 | e | 0 | 99.623 |  | .600 | 294.705 | 1 | 1.137 |
| 5 | e | 0 | 134.335 |  | .390 | 290.206 | 1 | 1.109 |
| 6 | e | 0 | 205.751 |  | .182 | 289.921 | 1 | 1.107 |
| 7 | e | 0 | 225.527 |  | .057 | 289.909 | 1 | 1.045 |
| 8 | e | 0 | 231.389 |  | .004 | 289.909 | 1 | 1.004 |
| 9 | e | 0 | 231.376 |  | .000 | 289.909 | 1 | 1.000 |

##### Bootstrap (H3a vs H3)

##### Summary of Bootstrap Iterations (H3a vs H3)

##### (H3a vs H3)

| Iterations | Method 0 | Method 1 | Method 2 |
| --- | --- | --- | --- |
| 1 | 0 | 0 | 0 |
| 2 | 0 | 0 | 0 |
| 3 | 0 | 0 | 0 |
| 4 | 0 | 0 | 0 |
| 5 | 0 | 0 | 0 |
| 6 | 0 | 1 | 0 |
| 7 | 0 | 23 | 0 |
| 8 | 0 | 98 | 0 |
| 9 | 0 | 177 | 0 |
| 10 | 0 | 159 | 0 |
| 11 | 0 | 184 | 0 |
| 12 | 0 | 132 | 0 |
| 13 | 0 | 97 | 0 |
| 14 | 0 | 48 | 0 |
| 15 | 0 | 35 | 0 |
| 16 | 0 | 13 | 0 |
| 17 | 0 | 13 | 0 |
| 18 | 0 | 7 | 0 |
| 19 | 0 | 13 | 0 |
| Total | 0 | 1000 | 0 |

0 bootstrap samples were unused because of a singular covariance matrix.

0 bootstrap samples were unused because a solution was not found.

1000 usable bootstrap samples were obtained.

##### Bootstrap Distributions (H3a vs H3)

##### ML discrepancy (implied vs sample) (H3a vs H3)

|  |  |  |
| --- | --- | --- |
|  |  | \|-------------------- |
|  | 438.811 | \|\* |
|  | 459.784 | \|\* |
|  | 480.758 | \|\*\*\* |
|  | 501.732 | \|\*\*\*\*\*\* |
|  | 522.706 | \|\*\*\*\*\*\*\*\*\*\*\*\*\* |
|  | 543.679 | \|\*\*\*\*\*\*\*\*\*\*\*\*\*\*\*\* |
|  | 564.653 | \|\*\*\*\*\*\*\*\*\*\*\*\*\*\*\*\*\*\*\*\* |
| N = 1000 | 585.627 | \|\*\*\*\*\*\*\*\*\*\*\*\*\*\*\*\*\*\*\* |
| Mean = 575.499 | 606.601 | \|\*\*\*\*\*\*\*\*\*\*\*\*\*\*\*\*\* |
| S. e. = 1.462 | 627.574 | \|\*\*\*\*\*\*\*\*\*\*\* |
|  | 648.548 | \|\*\*\*\*\* |
|  | 669.522 | \|\*\*\*\* |
|  | 690.495 | \|\*\* |
|  | 711.469 | \|\* |
|  | 732.443 | \|\* |
|  |  | \|-------------------- |

##### ML discrepancy (implied vs pop) (H3a vs H3)

|  |  |  |
| --- | --- | --- |
|  |  | \|-------------------- |
|  | 351.318 | \|\* |
|  | 359.392 | \|\* |
|  | 367.466 | \|\*\*\*\* |
|  | 375.540 | \|\*\*\*\*\*\*\*\*\*\* |
|  | 383.613 | \|\*\*\*\*\*\*\*\*\*\*\*\*\* |
|  | 391.687 | \|\*\*\*\*\*\*\*\*\*\*\*\*\*\*\*\*\*\* |
|  | 399.761 | \|\*\*\*\*\*\*\*\*\*\*\*\*\*\*\*\*\*\*\* |
| N = 1000 | 407.835 | \|\*\*\*\*\*\*\*\*\*\*\*\*\*\* |
| Mean = 399.243 | 415.908 | \|\*\*\*\*\*\*\*\*\*\* |
| S. e. = .590 | 423.982 | \|\*\*\*\*\*\*\* |
|  | 432.056 | \|\*\*\*\* |
|  | 440.130 | \|\*\*\* |
|  | 448.203 | \|\*\* |
|  | 456.277 | \|\* |
|  | 464.351 | \|\* |
|  |  | \|-------------------- |

##### K-L overoptimism (unstabilized) (H3a vs H3)

|  |  |  |
| --- | --- | --- |
|  |  | \|-------------------- |
|  | -143.627 | \|\* |
|  | -93.144 | \| |
|  | -42.660 | \|\*\* |
|  | 7.824 | \|\*\*\*\* |
|  | 58.307 | \|\*\*\*\*\*\*\*\*\*\* |
|  | 108.791 | \|\*\*\*\*\*\*\*\*\*\*\*\*\*\* |
|  | 159.275 | \|\*\*\*\*\*\*\*\*\*\*\*\*\*\*\*\*\*\*\* |
| N = 1000 | 209.758 | \|\*\*\*\*\*\*\*\*\*\*\*\*\*\*\*\*\*\*\* |
| Mean = 211.301 | 260.242 | \|\*\*\*\*\*\*\*\*\*\*\*\*\*\*\*\*\*\* |
| S. e. = 3.686 | 310.726 | \|\*\*\*\*\*\*\*\*\*\*\*\*\* |
|  | 361.209 | \|\*\*\*\*\*\*\* |
|  | 411.693 | \|\*\*\*\*\* |
|  | 462.177 | \|\*\*\* |
|  | 512.660 | \|\*\* |
|  | 563.144 | \|\* |
|  |  | \|-------------------- |

##### K-L overoptimism (stabilized) (H3a vs H3)

|  |  |  |
| --- | --- | --- |
|  |  | \|-------------------- |
|  | 88.283 | \|\* |
|  | 106.947 | \|\* |
|  | 125.612 | \|\*\* |
|  | 144.277 | \|\*\*\*\*\*\* |
|  | 162.941 | \|\*\*\*\*\*\*\*\*\*\* |
|  | 181.606 | \|\*\*\*\*\*\*\*\*\*\*\*\*\*\* |
|  | 200.270 | \|\*\*\*\*\*\*\*\*\*\*\*\*\*\*\*\*\*\*\* |
| N = 1000 | 218.935 | \|\*\*\*\*\*\*\*\*\*\*\*\*\*\*\*\*\* |
| Mean = 212.699 | 237.599 | \|\*\*\*\*\*\*\*\*\*\*\*\*\* |
| S. e. = 1.347 | 256.264 | \|\*\*\*\*\*\*\*\*\*\* |
|  | 274.928 | \|\*\*\*\*\*\*\* |
|  | 293.593 | \|\*\*\* |
|  | 312.257 | \|\*\* |
|  | 330.922 | \|\* |
|  | 349.586 | \|\* |
|  |  | \|-------------------- |

##### H4a vs H4 (H4a vs H4)

##### Notes for Model (H4a vs H4)

##### Computation of degrees of freedom (H4a vs H4)

|  |  |
| --- | --- |
| Number of distinct sample moments: | 342 |
| Number of distinct parameters to be estimated: | 81 |
| Degrees of freedom (342 - 81): | 261 |

##### Result (H4a vs H4)

Minimum was achieved

Chi-square = 289.068

Degrees of freedom = 261

Probability level = .112

##### Middle (Middle - H4a vs H4)

##### Estimates (Middle - H4a vs H4)

##### Scalar Estimates (Middle - H4a vs H4)

##### Maximum Likelihood Estimates

##### Regression Weights: (Middle - H4a vs H4)

|  |  |  | Estimate | S.E. | C.R. | P | Label |
| --- | --- | --- | --- | --- | --- | --- | --- |
| AI自我效能 | <--- | AI威胁感知 | .481 | .061 | 7.821 | \*\*\* | H1a |
| 防御性动机 | <--- | AI威胁感知 | .378 | .074 | 5.092 | \*\*\* | H2a |
| 防御性动机 | <--- | AI自我效能 | .141 | .085 | 1.661 | .097 | H5a |
| 使用行为 | <--- | 防御性动机 | .390 | .064 | 6.043 | \*\*\* | H4a |
| 使用行为 | <--- | AI自我效能 | .513 | .084 | 6.103 | \*\*\* | H3a |
| AA1 | <--- | AI威胁感知 | 1.000 |  |  |  |  |
| AA2 | <--- | AI威胁感知 | .950 | .065 | 14.701 | \*\*\* |  |
| AA3 | <--- | AI威胁感知 | .926 | .068 | 13.705 | \*\*\* |  |
| AA4 | <--- | AI威胁感知 | .936 | .066 | 14.149 | \*\*\* |  |
| AA5 | <--- | AI威胁感知 | .926 | .064 | 14.543 | \*\*\* |  |
| DM1 | <--- | 防御性动机 | 1.000 |  |  |  |  |
| DM2 | <--- | 防御性动机 | 1.070 | .101 | 10.587 | \*\*\* |  |
| DM3 | <--- | 防御性动机 | 1.084 | .101 | 10.731 | \*\*\* |  |
| DM5 | <--- | 防御性动机 | 1.014 | .101 | 10.091 | \*\*\* |  |
| SE1 | <--- | AI自我效能 | 1.000 |  |  |  |  |
| SE2 | <--- | AI自我效能 | 1.093 | .105 | 10.435 | \*\*\* |  |
| SE3 | <--- | AI自我效能 | .992 | .100 | 9.906 | \*\*\* |  |
| SE4 | <--- | AI自我效能 | 1.152 | .102 | 11.310 | \*\*\* |  |
| SE5 | <--- | AI自我效能 | 1.152 | .101 | 11.438 | \*\*\* |  |
| AB1 | <--- | 使用行为 | 1.000 |  |  |  |  |
| AB2 | <--- | 使用行为 | .922 | .078 | 11.868 | \*\*\* |  |
| AB3 | <--- | 使用行为 | 1.035 | .077 | 13.419 | \*\*\* |  |
| AB5 | <--- | 使用行为 | .961 | .078 | 12.278 | \*\*\* |  |

##### Standardized Regression Weights: (Middle - H4a vs H4)

|  |  |  | Estimate |
| --- | --- | --- | --- |
| AI自我效能 | <--- | AI威胁感知 | .579 |
| 防御性动机 | <--- | AI威胁感知 | .445 |
| 防御性动机 | <--- | AI自我效能 | .137 |
| 使用行为 | <--- | 防御性动机 | .345 |
| 使用行为 | <--- | AI自我效能 | .444 |
| AA1 | <--- | AI威胁感知 | .824 |
| AA2 | <--- | AI威胁感知 | .789 |
| AA3 | <--- | AI威胁感知 | .748 |
| AA4 | <--- | AI威胁感知 | .766 |
| AA5 | <--- | AI威胁感知 | .783 |
| DM1 | <--- | 防御性动机 | .687 |
| DM2 | <--- | 防御性动机 | .747 |
| DM3 | <--- | 防御性动机 | .761 |
| DM5 | <--- | 防御性动机 | .703 |
| SE1 | <--- | AI自我效能 | .675 |
| SE2 | <--- | AI自我效能 | .715 |
| SE3 | <--- | AI自我效能 | .674 |
| SE4 | <--- | AI自我效能 | .790 |
| SE5 | <--- | AI自我效能 | .802 |
| AB1 | <--- | 使用行为 | .774 |
| AB2 | <--- | 使用行为 | .720 |
| AB3 | <--- | 使用行为 | .813 |
| AB5 | <--- | 使用行为 | .743 |

##### Variances: (Middle - H4a vs H4)

|  |  |  | Estimate | S.E. | C.R. | P | Label |
| --- | --- | --- | --- | --- | --- | --- | --- |
| AI威胁感知 |  |  | 2.208 | .272 | 8.134 | \*\*\* |  |
| e21 |  |  | 1.010 | .174 | 5.798 | \*\*\* |  |
| e23 |  |  | 1.132 | .192 | 5.880 | \*\*\* |  |
| e22 |  |  | 1.144 | .175 | 6.545 | \*\*\* |  |
| e1 |  |  | 1.045 | .118 | 8.886 | \*\*\* |  |
| e2 |  |  | 1.208 | .127 | 9.541 | \*\*\* |  |
| e3 |  |  | 1.491 | .148 | 10.078 | \*\*\* |  |
| e4 |  |  | 1.360 | .138 | 9.861 | \*\*\* |  |
| e5 |  |  | 1.197 | .124 | 9.639 | \*\*\* |  |
| e6 |  |  | 1.776 | .183 | 9.696 | \*\*\* |  |
| e8 |  |  | 1.443 | .165 | 8.764 | \*\*\* |  |
| e9 |  |  | 1.356 | .160 | 8.484 | \*\*\* |  |
| e10 |  |  | 1.677 | .177 | 9.478 | \*\*\* |  |
| e11 |  |  | 1.819 | .175 | 10.386 | \*\*\* |  |
| e12 |  |  | 1.733 | .173 | 10.008 | \*\*\* |  |
| e13 |  |  | 1.804 | .174 | 10.396 | \*\*\* |  |
| e14 |  |  | 1.217 | .136 | 8.920 | \*\*\* |  |
| e15 |  |  | 1.122 | .129 | 8.673 | \*\*\* |  |
| e16 |  |  | 1.356 | .151 | 8.961 | \*\*\* |  |
| e17 |  |  | 1.607 | .164 | 9.792 | \*\*\* |  |
| e18 |  |  | 1.120 | .138 | 8.096 | \*\*\* |  |
| e20 |  |  | 1.522 | .161 | 9.482 | \*\*\* |  |

##### Squared Multiple Correlations: (Middle - H4a vs H4)

|  |  |  | Estimate |
| --- | --- | --- | --- |
| AI自我效能 |  |  | .336 |
| 防御性动机 |  |  | .288 |
| 使用行为 |  |  | .437 |
| AB5 |  |  | .552 |
| AB3 |  |  | .660 |
| AB2 |  |  | .518 |
| AB1 |  |  | .600 |
| SE5 |  |  | .643 |
| SE4 |  |  | .624 |
| SE3 |  |  | .454 |
| SE2 |  |  | .512 |
| SE1 |  |  | .455 |
| DM5 |  |  | .494 |
| DM3 |  |  | .579 |
| DM2 |  |  | .558 |
| DM1 |  |  | .472 |
| AA5 |  |  | .612 |
| AA4 |  |  | .587 |
| AA3 |  |  | .559 |
| AA2 |  |  | .622 |
| AA1 |  |  | .679 |

##### User-defined estimands: (Middle - H4a vs H4)

|  |  |  |  |
| --- | --- | --- | --- |
| diff1 |  |  | .335 |
| diff2 |  |  | .232 |
| diff3 |  |  | .367 |
| diff4 |  |  | .244 |
| diff5 |  |  | -.005 |

##### Modification Indices (Middle - H4a vs H4)

##### Covariances: (Middle - H4a vs H4)

|  |  |  | M.I. | Par Change |
| --- | --- | --- | --- | --- |
| e8 | <--> | e22 | 11.948 | .351 |
| e8 | <--> | e17 | 4.224 | .227 |
| e5 | <--> | e17 | 5.161 | -.223 |
| e4 | <--> | e15 | 4.338 | -.189 |
| e3 | <--> | e17 | 5.618 | .254 |
| e3 | <--> | e4 | 4.326 | -.203 |
| e2 | <--> | e22 | 9.018 | -.273 |
| e1 | <--> | e15 | 4.137 | .169 |
| e1 | <--> | e12 | 8.116 | -.278 |

##### Variances: (Middle - H4a vs H4)

|  |  |  | M.I. | Par Change |
| --- | --- | --- | --- | --- |

##### Regression Weights: (Middle - H4a vs H4)

|  |  |  | M.I. | Par Change |
| --- | --- | --- | --- | --- |
| DM2 | <--- | 使用行为 | 7.578 | .167 |
| DM2 | <--- | AB3 | 6.459 | .113 |
| DM2 | <--- | AB2 | 9.997 | .140 |
| DM2 | <--- | AB1 | 6.951 | .116 |
| AA2 | <--- | AB2 | 6.183 | -.098 |
| AA1 | <--- | SE2 | 4.861 | -.081 |

##### Bootstrap (Middle - H4a vs H4)

##### Bootstrap standard errors (Middle - H4a vs H4)

##### Scalar Estimates (Middle - H4a vs H4)

##### Regression Weights: (Middle - H4a vs H4)

| Parameter | | | SE | SE-SE | Mean | Bias | SE-Bias |
| --- | --- | --- | --- | --- | --- | --- | --- |
| AI自我效能 | <--- | AI威胁感知 | .058 | .001 | .482 | .001 | .002 |
| 防御性动机 | <--- | AI威胁感知 | .076 | .002 | .380 | .003 | .002 |
| 防御性动机 | <--- | AI自我效能 | .087 | .002 | .140 | .000 | .003 |
| 使用行为 | <--- | 防御性动机 | .061 | .001 | .388 | -.001 | .002 |
| 使用行为 | <--- | AI自我效能 | .082 | .002 | .511 | -.002 | .003 |
| AA1 | <--- | AI威胁感知 | .000 | .000 | 1.000 | .000 | .000 |
| AA2 | <--- | AI威胁感知 | .052 | .001 | .953 | .003 | .002 |
| AA3 | <--- | AI威胁感知 | .055 | .001 | .925 | -.001 | .002 |
| AA4 | <--- | AI威胁感知 | .058 | .001 | .934 | -.002 | .002 |
| AA5 | <--- | AI威胁感知 | .058 | .001 | .928 | .002 | .002 |
| DM1 | <--- | 防御性动机 | .000 | .000 | 1.000 | .000 | .000 |
| DM2 | <--- | 防御性动机 | .097 | .002 | 1.073 | .003 | .003 |
| DM3 | <--- | 防御性动机 | .095 | .002 | 1.088 | .005 | .003 |
| DM5 | <--- | 防御性动机 | .103 | .002 | 1.019 | .005 | .003 |
| SE1 | <--- | AI自我效能 | .000 | .000 | 1.000 | .000 | .000 |
| SE2 | <--- | AI自我效能 | .094 | .002 | 1.092 | -.001 | .003 |
| SE3 | <--- | AI自我效能 | .088 | .002 | .995 | .002 | .003 |
| SE4 | <--- | AI自我效能 | .096 | .002 | 1.154 | .003 | .003 |
| SE5 | <--- | AI自我效能 | .096 | .002 | 1.151 | -.001 | .003 |
| AB1 | <--- | 使用行为 | .000 | .000 | 1.000 | .000 | .000 |
| AB2 | <--- | 使用行为 | .066 | .001 | .925 | .003 | .002 |
| AB3 | <--- | 使用行为 | .069 | .002 | 1.041 | .006 | .002 |
| AB5 | <--- | 使用行为 | .071 | .002 | .962 | .001 | .002 |

##### Standardized Regression Weights: (Middle - H4a vs H4)

| Parameter | | | SE | SE-SE | Mean | Bias | SE-Bias |
| --- | --- | --- | --- | --- | --- | --- | --- |
| AI自我效能 | <--- | AI威胁感知 | .046 | .001 | .577 | -.003 | .001 |
| 防御性动机 | <--- | AI威胁感知 | .079 | .002 | .448 | .002 | .003 |
| 防御性动机 | <--- | AI自我效能 | .083 | .002 | .137 | .000 | .003 |
| 使用行为 | <--- | 防御性动机 | .053 | .001 | .344 | -.001 | .002 |
| 使用行为 | <--- | AI自我效能 | .060 | .001 | .442 | -.001 | .002 |
| AA1 | <--- | AI威胁感知 | .026 | .001 | .823 | -.001 | .001 |
| AA2 | <--- | AI威胁感知 | .024 | .001 | .790 | .001 | .001 |
| AA3 | <--- | AI威胁感知 | .029 | .001 | .747 | -.001 | .001 |
| AA4 | <--- | AI威胁感知 | .029 | .001 | .764 | -.002 | .001 |
| AA5 | <--- | AI威胁感知 | .032 | .001 | .784 | .002 | .001 |
| DM1 | <--- | 防御性动机 | .042 | .001 | .686 | -.001 | .001 |
| DM2 | <--- | 防御性动机 | .034 | .001 | .745 | -.002 | .001 |
| DM3 | <--- | 防御性动机 | .034 | .001 | .760 | -.001 | .001 |
| DM5 | <--- | 防御性动机 | .038 | .001 | .701 | -.001 | .001 |
| SE1 | <--- | AI自我效能 | .038 | .001 | .676 | .001 | .001 |
| SE2 | <--- | AI自我效能 | .037 | .001 | .715 | -.001 | .001 |
| SE3 | <--- | AI自我效能 | .039 | .001 | .673 | .000 | .001 |
| SE4 | <--- | AI自我效能 | .027 | .001 | .790 | .000 | .001 |
| SE5 | <--- | AI自我效能 | .027 | .001 | .799 | -.003 | .001 |
| AB1 | <--- | 使用行为 | .032 | .001 | .773 | -.002 | .001 |
| AB2 | <--- | 使用行为 | .036 | .001 | .718 | -.001 | .001 |
| AB3 | <--- | 使用行为 | .027 | .001 | .813 | .001 | .001 |
| AB5 | <--- | 使用行为 | .034 | .001 | .742 | -.001 | .001 |

##### Variances: (Middle - H4a vs H4)

| Parameter | | | SE | SE-SE | Mean | Bias | SE-Bias |
| --- | --- | --- | --- | --- | --- | --- | --- |
| AI威胁感知 |  |  | .214 | .005 | 2.203 | -.005 | .007 |
| e21 |  |  | .160 | .004 | 1.017 | .007 | .005 |
| e23 |  |  | .189 | .004 | 1.116 | -.015 | .006 |
| e22 |  |  | .160 | .004 | 1.131 | -.013 | .005 |
| e1 |  |  | .125 | .003 | 1.042 | -.003 | .004 |
| e2 |  |  | .116 | .003 | 1.196 | -.011 | .004 |
| e3 |  |  | .129 | .003 | 1.477 | -.014 | .004 |
| e4 |  |  | .133 | .003 | 1.356 | -.004 | .004 |
| e5 |  |  | .146 | .003 | 1.175 | -.022 | .005 |
| e6 |  |  | .187 | .004 | 1.766 | -.010 | .006 |
| e8 |  |  | .156 | .003 | 1.437 | -.006 | .005 |
| e9 |  |  | .153 | .003 | 1.344 | -.012 | .005 |
| e10 |  |  | .165 | .004 | 1.667 | -.009 | .005 |
| e11 |  |  | .153 | .003 | 1.802 | -.017 | .005 |
| e12 |  |  | .198 | .004 | 1.722 | -.011 | .006 |
| e13 |  |  | .162 | .004 | 1.794 | -.010 | .005 |
| e14 |  |  | .128 | .003 | 1.207 | -.010 | .004 |
| e15 |  |  | .128 | .003 | 1.128 | .006 | .004 |
| e16 |  |  | .154 | .003 | 1.351 | -.006 | .005 |
| e17 |  |  | .167 | .004 | 1.599 | -.008 | .005 |
| e18 |  |  | .132 | .003 | 1.108 | -.013 | .004 |
| e20 |  |  | .151 | .003 | 1.508 | -.014 | .005 |

##### Squared Multiple Correlations: (Middle - H4a vs H4)

| Parameter | | | SE | SE-SE | Mean | Bias | SE-Bias |
| --- | --- | --- | --- | --- | --- | --- | --- |
| AI自我效能 |  |  | .053 | .001 | .335 | -.001 | .002 |
| 防御性动机 |  |  | .058 | .001 | .298 | .010 | .002 |
| 使用行为 |  |  | .057 | .001 | .439 | .002 | .002 |
| AB5 |  |  | .051 | .001 | .551 | -.001 | .002 |
| AB3 |  |  | .043 | .001 | .662 | .002 | .001 |
| AB2 |  |  | .052 | .001 | .517 | .000 | .002 |
| AB1 |  |  | .049 | .001 | .598 | -.001 | .002 |
| SE5 |  |  | .042 | .001 | .639 | -.003 | .001 |
| SE4 |  |  | .042 | .001 | .625 | .001 | .001 |
| SE3 |  |  | .052 | .001 | .455 | .001 | .002 |
| SE2 |  |  | .053 | .001 | .512 | .000 | .002 |
| SE1 |  |  | .051 | .001 | .458 | .003 | .002 |
| DM5 |  |  | .053 | .001 | .493 | .000 | .002 |
| DM3 |  |  | .052 | .001 | .580 | .000 | .002 |
| DM2 |  |  | .051 | .001 | .556 | -.001 | .002 |
| DM1 |  |  | .057 | .001 | .473 | .000 | .002 |
| AA5 |  |  | .049 | .001 | .616 | .004 | .002 |
| AA4 |  |  | .044 | .001 | .585 | -.003 | .001 |
| AA3 |  |  | .044 | .001 | .559 | .000 | .001 |
| AA2 |  |  | .038 | .001 | .624 | .002 | .001 |
| AA1 |  |  | .042 | .001 | .678 | -.001 | .001 |

##### User-defined estimands: (Middle - H4a vs H4)

| Parameter | | | SE | SE-SE | Mean | Bias | SE-Bias |
| --- | --- | --- | --- | --- | --- | --- | --- |
| diff1 |  |  | .100 | .002 | .333 | -.002 | .003 |
| diff2 |  |  | .108 | .002 | .232 | .000 | .003 |
| diff3 |  |  | .116 | .003 | .363 | -.004 | .004 |
| diff4 |  |  | .102 | .002 | .240 | -.004 | .003 |
| diff5 |  |  | .123 | .003 | -.008 | -.003 | .004 |

##### Bootstrap Confidence (Middle - H4a vs H4)

##### Percentile method (Middle - H4a vs H4)

##### 95% confidence intervals (percentile method)

##### Scalar Estimates (Middle - H4a vs H4)

##### Regression Weights: (Middle - H4a vs H4)

| Parameter | | | Estimate | Lower | Upper | P |
| --- | --- | --- | --- | --- | --- | --- |
| AI自我效能 | <--- | AI威胁感知 | .481 | .371 | .594 | .002 |
| 防御性动机 | <--- | AI威胁感知 | .378 | .240 | .533 | .002 |
| 防御性动机 | <--- | AI自我效能 | .141 | -.029 | .309 | .102 |
| 使用行为 | <--- | 防御性动机 | .390 | .270 | .515 | .002 |
| 使用行为 | <--- | AI自我效能 | .513 | .354 | .676 | .002 |
| AA1 | <--- | AI威胁感知 | 1.000 | 1.000 | 1.000 | ... |
| AA2 | <--- | AI威胁感知 | .950 | .853 | 1.058 | .002 |
| AA3 | <--- | AI威胁感知 | .926 | .819 | 1.037 | .002 |
| AA4 | <--- | AI威胁感知 | .936 | .821 | 1.055 | .002 |
| AA5 | <--- | AI威胁感知 | .926 | .815 | 1.053 | .002 |
| DM1 | <--- | 防御性动机 | 1.000 | 1.000 | 1.000 | ... |
| DM2 | <--- | 防御性动机 | 1.070 | .902 | 1.279 | .002 |
| DM3 | <--- | 防御性动机 | 1.084 | .922 | 1.292 | .002 |
| DM5 | <--- | 防御性动机 | 1.014 | .838 | 1.255 | .002 |
| SE1 | <--- | AI自我效能 | 1.000 | 1.000 | 1.000 | ... |
| SE2 | <--- | AI自我效能 | 1.093 | .912 | 1.282 | .002 |
| SE3 | <--- | AI自我效能 | .992 | .835 | 1.173 | .002 |
| SE4 | <--- | AI自我效能 | 1.152 | .985 | 1.351 | .002 |
| SE5 | <--- | AI自我效能 | 1.152 | .976 | 1.344 | .002 |
| AB1 | <--- | 使用行为 | 1.000 | 1.000 | 1.000 | ... |
| AB2 | <--- | 使用行为 | .922 | .805 | 1.061 | .002 |
| AB3 | <--- | 使用行为 | 1.035 | .920 | 1.193 | .002 |
| AB5 | <--- | 使用行为 | .961 | .833 | 1.118 | .002 |

##### Standardized Regression Weights: (Middle - H4a vs H4)

| Parameter | | | Estimate | Lower | Upper | P |
| --- | --- | --- | --- | --- | --- | --- |
| AI自我效能 | <--- | AI威胁感知 | .579 | .484 | .660 | .002 |
| 防御性动机 | <--- | AI威胁感知 | .445 | .287 | .602 | .002 |
| 防御性动机 | <--- | AI自我效能 | .137 | -.029 | .299 | .102 |
| 使用行为 | <--- | 防御性动机 | .345 | .238 | .449 | .002 |
| 使用行为 | <--- | AI自我效能 | .444 | .316 | .553 | .002 |
| AA1 | <--- | AI威胁感知 | .824 | .767 | .872 | .002 |
| AA2 | <--- | AI威胁感知 | .789 | .737 | .836 | .002 |
| AA3 | <--- | AI威胁感知 | .748 | .685 | .801 | .002 |
| AA4 | <--- | AI威胁感知 | .766 | .704 | .819 | .002 |
| AA5 | <--- | AI威胁感知 | .783 | .722 | .843 | .002 |
| DM1 | <--- | 防御性动机 | .687 | .595 | .762 | .002 |
| DM2 | <--- | 防御性动机 | .747 | .675 | .806 | .002 |
| DM3 | <--- | 防御性动机 | .761 | .693 | .825 | .002 |
| DM5 | <--- | 防御性动机 | .703 | .623 | .771 | .002 |
| SE1 | <--- | AI自我效能 | .675 | .602 | .746 | .002 |
| SE2 | <--- | AI自我效能 | .715 | .633 | .787 | .002 |
| SE3 | <--- | AI自我效能 | .674 | .591 | .746 | .002 |
| SE4 | <--- | AI自我效能 | .790 | .733 | .842 | .002 |
| SE5 | <--- | AI自我效能 | .802 | .742 | .845 | .002 |
| AB1 | <--- | 使用行为 | .774 | .702 | .829 | .002 |
| AB2 | <--- | 使用行为 | .720 | .647 | .783 | .002 |
| AB3 | <--- | 使用行为 | .813 | .758 | .860 | .002 |
| AB5 | <--- | 使用行为 | .743 | .666 | .804 | .002 |

##### Variances: (Middle - H4a vs H4)

| Parameter | | | Estimate | Lower | Upper | P |
| --- | --- | --- | --- | --- | --- | --- |
| AI威胁感知 |  |  | 2.208 | 1.769 | 2.632 | .002 |
| e21 |  |  | 1.010 | .738 | 1.337 | .002 |
| e23 |  |  | 1.132 | .768 | 1.519 | .002 |
| e22 |  |  | 1.144 | .836 | 1.445 | .002 |
| e1 |  |  | 1.045 | .802 | 1.297 | .002 |
| e2 |  |  | 1.208 | .982 | 1.435 | .002 |
| e3 |  |  | 1.491 | 1.235 | 1.735 | .002 |
| e4 |  |  | 1.360 | 1.106 | 1.635 | .002 |
| e5 |  |  | 1.197 | .908 | 1.476 | .002 |
| e6 |  |  | 1.776 | 1.428 | 2.149 | .002 |
| e8 |  |  | 1.443 | 1.135 | 1.761 | .002 |
| e9 |  |  | 1.356 | 1.042 | 1.628 | .002 |
| e10 |  |  | 1.677 | 1.336 | 1.984 | .002 |
| e11 |  |  | 1.819 | 1.500 | 2.117 | .002 |
| e12 |  |  | 1.733 | 1.315 | 2.121 | .002 |
| e13 |  |  | 1.804 | 1.485 | 2.123 | .002 |
| e14 |  |  | 1.217 | .947 | 1.461 | .002 |
| e15 |  |  | 1.122 | .899 | 1.399 | .002 |
| e16 |  |  | 1.356 | 1.059 | 1.646 | .002 |
| e17 |  |  | 1.607 | 1.293 | 1.952 | .002 |
| e18 |  |  | 1.120 | .862 | 1.363 | .002 |
| e20 |  |  | 1.522 | 1.209 | 1.838 | .002 |

##### Squared Multiple Correlations: (Middle - H4a vs H4)

| Parameter | | | Estimate | Lower | Upper | P |
| --- | --- | --- | --- | --- | --- | --- |
| AI自我效能 |  |  | .336 | .234 | .436 | .002 |
| 防御性动机 |  |  | .288 | .182 | .416 | .002 |
| 使用行为 |  |  | .437 | .328 | .552 | .002 |
| AB5 |  |  | .552 | .443 | .647 | .002 |
| AB3 |  |  | .660 | .574 | .740 | .002 |
| AB2 |  |  | .518 | .418 | .614 | .002 |
| AB1 |  |  | .600 | .493 | .687 | .002 |
| SE5 |  |  | .643 | .550 | .714 | .002 |
| SE4 |  |  | .624 | .537 | .708 | .002 |
| SE3 |  |  | .454 | .349 | .557 | .002 |
| SE2 |  |  | .512 | .400 | .620 | .002 |
| SE1 |  |  | .455 | .363 | .557 | .002 |
| DM5 |  |  | .494 | .388 | .594 | .002 |
| DM3 |  |  | .579 | .480 | .680 | .002 |
| DM2 |  |  | .558 | .455 | .649 | .002 |
| DM1 |  |  | .472 | .354 | .580 | .002 |
| AA5 |  |  | .612 | .521 | .711 | .002 |
| AA4 |  |  | .587 | .495 | .670 | .002 |
| AA3 |  |  | .559 | .469 | .641 | .002 |
| AA2 |  |  | .622 | .543 | .698 | .002 |
| AA1 |  |  | .679 | .589 | .761 | .002 |

##### User-defined estimands: (Middle - H4a vs H4)

| Parameter | | | Estimate | Lower | Upper | P |
| --- | --- | --- | --- | --- | --- | --- |
| diff1 |  |  | .335 | .131 | .526 | .002 |
| diff2 |  |  | .232 | .017 | .448 | .029 |
| diff3 |  |  | .367 | .127 | .590 | .002 |
| diff4 |  |  | .244 | .030 | .432 | .018 |
| diff5 |  |  | -.005 | -.262 | .227 | .963 |

##### Bias-corrected percentile method (Middle - H4a vs H4)

##### 95% confidence intervals (bias-corrected percentile method)

##### Scalar Estimates (Middle - H4a vs H4)

##### Regression Weights: (Middle - H4a vs H4)

| Parameter | | | Estimate | Lower | Upper | P |
| --- | --- | --- | --- | --- | --- | --- |
| AI自我效能 | <--- | AI威胁感知 | .481 | .376 | .600 | .001 |
| 防御性动机 | <--- | AI威胁感知 | .378 | .238 | .529 | .002 |
| 防御性动机 | <--- | AI自我效能 | .141 | -.040 | .306 | .109 |
| 使用行为 | <--- | 防御性动机 | .390 | .272 | .516 | .002 |
| 使用行为 | <--- | AI自我效能 | .513 | .363 | .687 | .001 |
| AA1 | <--- | AI威胁感知 | 1.000 | 1.000 | 1.000 | ... |
| AA2 | <--- | AI威胁感知 | .950 | .848 | 1.054 | .003 |
| AA3 | <--- | AI威胁感知 | .926 | .824 | 1.046 | .001 |
| AA4 | <--- | AI威胁感知 | .936 | .826 | 1.060 | .001 |
| AA5 | <--- | AI威胁感知 | .926 | .811 | 1.048 | .002 |
| DM1 | <--- | 防御性动机 | 1.000 | 1.000 | 1.000 | ... |
| DM2 | <--- | 防御性动机 | 1.070 | .902 | 1.279 | .002 |
| DM3 | <--- | 防御性动机 | 1.084 | .923 | 1.296 | .002 |
| DM5 | <--- | 防御性动机 | 1.014 | .835 | 1.255 | .002 |
| SE1 | <--- | AI自我效能 | 1.000 | 1.000 | 1.000 | ... |
| SE2 | <--- | AI自我效能 | 1.093 | .933 | 1.297 | .001 |
| SE3 | <--- | AI自我效能 | .992 | .835 | 1.173 | .002 |
| SE4 | <--- | AI自我效能 | 1.152 | .994 | 1.362 | .001 |
| SE5 | <--- | AI自我效能 | 1.152 | .980 | 1.346 | .002 |
| AB1 | <--- | 使用行为 | 1.000 | 1.000 | 1.000 | ... |
| AB2 | <--- | 使用行为 | .922 | .802 | 1.059 | .002 |
| AB3 | <--- | 使用行为 | 1.035 | .918 | 1.185 | .002 |
| AB5 | <--- | 使用行为 | .961 | .835 | 1.134 | .001 |

##### Standardized Regression Weights: (Middle - H4a vs H4)

| Parameter | | | Estimate | Lower | Upper | P |
| --- | --- | --- | --- | --- | --- | --- |
| AI自我效能 | <--- | AI威胁感知 | .579 | .486 | .664 | .001 |
| 防御性动机 | <--- | AI威胁感知 | .445 | .268 | .582 | .004 |
| 防御性动机 | <--- | AI自我效能 | .137 | -.040 | .294 | .120 |
| 使用行为 | <--- | 防御性动机 | .345 | .241 | .452 | .002 |
| 使用行为 | <--- | AI自我效能 | .444 | .311 | .546 | .003 |
| AA1 | <--- | AI威胁感知 | .824 | .767 | .870 | .003 |
| AA2 | <--- | AI威胁感知 | .789 | .734 | .831 | .003 |
| AA3 | <--- | AI威胁感知 | .748 | .685 | .803 | .002 |
| AA4 | <--- | AI威胁感知 | .766 | .709 | .820 | .001 |
| AA5 | <--- | AI威胁感知 | .783 | .716 | .839 | .003 |
| DM1 | <--- | 防御性动机 | .687 | .595 | .761 | .002 |
| DM2 | <--- | 防御性动机 | .747 | .678 | .806 | .002 |
| DM3 | <--- | 防御性动机 | .761 | .693 | .824 | .002 |
| DM5 | <--- | 防御性动机 | .703 | .625 | .775 | .002 |
| SE1 | <--- | AI自我效能 | .675 | .596 | .744 | .003 |
| SE2 | <--- | AI自我效能 | .715 | .631 | .784 | .002 |
| SE3 | <--- | AI自我效能 | .674 | .584 | .742 | .003 |
| SE4 | <--- | AI自我效能 | .790 | .732 | .841 | .002 |
| SE5 | <--- | AI自我效能 | .802 | .744 | .847 | .001 |
| AB1 | <--- | 使用行为 | .774 | .704 | .830 | .002 |
| AB2 | <--- | 使用行为 | .720 | .649 | .785 | .002 |
| AB3 | <--- | 使用行为 | .813 | .753 | .858 | .003 |
| AB5 | <--- | 使用行为 | .743 | .665 | .803 | .002 |

##### Variances: (Middle - H4a vs H4)

| Parameter | | | Estimate | Lower | Upper | P |
| --- | --- | --- | --- | --- | --- | --- |
| AI威胁感知 |  |  | 2.208 | 1.780 | 2.665 | .002 |
| e21 |  |  | 1.010 | .741 | 1.355 | .002 |
| e23 |  |  | 1.132 | .815 | 1.561 | .001 |
| e22 |  |  | 1.144 | .866 | 1.470 | .001 |
| e1 |  |  | 1.045 | .819 | 1.305 | .001 |
| e2 |  |  | 1.208 | 1.015 | 1.508 | .001 |
| e3 |  |  | 1.491 | 1.265 | 1.771 | .001 |
| e4 |  |  | 1.360 | 1.129 | 1.654 | .001 |
| e5 |  |  | 1.197 | .950 | 1.528 | .001 |
| e6 |  |  | 1.776 | 1.434 | 2.169 | .001 |
| e8 |  |  | 1.443 | 1.168 | 1.778 | .001 |
| e9 |  |  | 1.356 | 1.051 | 1.645 | .002 |
| e10 |  |  | 1.677 | 1.346 | 1.985 | .002 |
| e11 |  |  | 1.819 | 1.537 | 2.148 | .001 |
| e12 |  |  | 1.733 | 1.363 | 2.163 | .001 |
| e13 |  |  | 1.804 | 1.500 | 2.159 | .001 |
| e14 |  |  | 1.217 | .976 | 1.497 | .001 |
| e15 |  |  | 1.122 | .907 | 1.408 | .002 |
| e16 |  |  | 1.356 | 1.069 | 1.668 | .002 |
| e17 |  |  | 1.607 | 1.305 | 1.968 | .001 |
| e18 |  |  | 1.120 | .888 | 1.384 | .001 |
| e20 |  |  | 1.522 | 1.252 | 1.893 | .001 |

##### Squared Multiple Correlations: (Middle - H4a vs H4)

| Parameter | | | Estimate | Lower | Upper | P |
| --- | --- | --- | --- | --- | --- | --- |
| AI自我效能 |  |  | .336 | .236 | .441 | .001 |
| 防御性动机 |  |  | .288 | .161 | .390 | .006 |
| 使用行为 |  |  | .437 | .316 | .549 | .003 |
| AB5 |  |  | .552 | .443 | .645 | .002 |
| AB3 |  |  | .660 | .567 | .736 | .003 |
| AB2 |  |  | .518 | .422 | .616 | .002 |
| AB1 |  |  | .600 | .496 | .689 | .002 |
| SE5 |  |  | .643 | .554 | .718 | .001 |
| SE4 |  |  | .624 | .535 | .707 | .002 |
| SE3 |  |  | .454 | .341 | .550 | .003 |
| SE2 |  |  | .512 | .398 | .615 | .002 |
| SE1 |  |  | .455 | .355 | .554 | .003 |
| DM5 |  |  | .494 | .391 | .601 | .002 |
| DM3 |  |  | .579 | .480 | .680 | .002 |
| DM2 |  |  | .558 | .460 | .650 | .002 |
| DM1 |  |  | .472 | .354 | .580 | .002 |
| AA5 |  |  | .612 | .512 | .703 | .003 |
| AA4 |  |  | .587 | .502 | .672 | .001 |
| AA3 |  |  | .559 | .470 | .644 | .002 |
| AA2 |  |  | .622 | .539 | .691 | .003 |
| AA1 |  |  | .679 | .588 | .756 | .003 |

##### User-defined estimands: (Middle - H4a vs H4)

| Parameter | | | Estimate | Lower | Upper | P |
| --- | --- | --- | --- | --- | --- | --- |
| diff1 |  |  | .335 | .125 | .526 | .002 |
| diff2 |  |  | .232 | .025 | .452 | .026 |
| diff3 |  |  | .367 | .130 | .600 | .002 |
| diff4 |  |  | .244 | .042 | .448 | .013 |
| diff5 |  |  | -.005 | -.259 | .231 | .991 |

##### others (others - H4a vs H4)

##### Estimates (others - H4a vs H4)

##### Scalar Estimates (others - H4a vs H4)

##### Maximum Likelihood Estimates

##### Regression Weights: (others - H4a vs H4)

|  |  |  | Estimate | S.E. | C.R. | P | Label |
| --- | --- | --- | --- | --- | --- | --- | --- |
| AI自我效能 | <--- | AI威胁感知 | .146 | .074 | 1.969 | .049 | H1 |
| 防御性动机 | <--- | AI威胁感知 | .379 | .116 | 3.261 | .001 | H2 |
| 防御性动机 | <--- | AI自我效能 | -.163 | .176 | -.926 | .354 | H5 |
| 使用行为 | <--- | 防御性动机 | .390 | .064 | 6.043 | \*\*\* | H4a |
| 使用行为 | <--- | AI自我效能 | .339 | .155 | 2.189 | .029 | H3 |
| AA1 | <--- | AI威胁感知 | 1.000 |  |  |  |  |
| AA2 | <--- | AI威胁感知 | .952 | .115 | 8.264 | \*\*\* |  |
| AA3 | <--- | AI威胁感知 | .893 | .110 | 8.081 | \*\*\* |  |
| AA4 | <--- | AI威胁感知 | .871 | .116 | 7.537 | \*\*\* |  |
| AA5 | <--- | AI威胁感知 | .995 | .117 | 8.516 | \*\*\* |  |
| DM1 | <--- | 防御性动机 | 1.000 |  |  |  |  |
| DM2 | <--- | 防御性动机 | .894 | .128 | 6.955 | \*\*\* |  |
| DM3 | <--- | 防御性动机 | .924 | .129 | 7.177 | \*\*\* |  |
| DM5 | <--- | 防御性动机 | .917 | .129 | 7.133 | \*\*\* |  |
| SE1 | <--- | AI自我效能 | 1.000 |  |  |  |  |
| SE2 | <--- | AI自我效能 | 1.368 | .273 | 5.007 | \*\*\* |  |
| SE3 | <--- | AI自我效能 | 1.493 | .283 | 5.270 | \*\*\* |  |
| SE4 | <--- | AI自我效能 | 1.709 | .314 | 5.441 | \*\*\* |  |
| SE5 | <--- | AI自我效能 | 1.479 | .281 | 5.259 | \*\*\* |  |
| AB1 | <--- | 使用行为 | 1.000 |  |  |  |  |
| AB2 | <--- | 使用行为 | 1.159 | .156 | 7.422 | \*\*\* |  |
| AB3 | <--- | 使用行为 | 1.129 | .156 | 7.223 | \*\*\* |  |
| AB5 | <--- | 使用行为 | .935 | .152 | 6.164 | \*\*\* |  |

##### Standardized Regression Weights: (others - H4a vs H4)

|  |  |  | Estimate |
| --- | --- | --- | --- |
| AI自我效能 | <--- | AI威胁感知 | .230 |
| 防御性动机 | <--- | AI威胁感知 | .389 |
| 防御性动机 | <--- | AI自我效能 | -.106 |
| 使用行为 | <--- | 防御性动机 | .441 |
| 使用行为 | <--- | AI自我效能 | .250 |
| AA1 | <--- | AI威胁感知 | .807 |
| AA2 | <--- | AI威胁感知 | .767 |
| AA3 | <--- | AI威胁感知 | .753 |
| AA4 | <--- | AI威胁感知 | .710 |
| AA5 | <--- | AI威胁感知 | .787 |
| DM1 | <--- | 防御性动机 | .771 |
| DM2 | <--- | 防御性动机 | .715 |
| DM3 | <--- | 防御性动机 | .739 |
| DM5 | <--- | 防御性动机 | .734 |
| SE1 | <--- | AI自我效能 | .535 |
| SE2 | <--- | AI自我效能 | .701 |
| SE3 | <--- | AI自我效能 | .774 |
| SE4 | <--- | AI自我效能 | .834 |
| SE5 | <--- | AI自我效能 | .771 |
| AB1 | <--- | 使用行为 | .717 |
| AB2 | <--- | 使用行为 | .809 |
| AB3 | <--- | 使用行为 | .779 |
| AB5 | <--- | 使用行为 | .656 |

##### Variances: (others - H4a vs H4)

|  |  |  | Estimate | S.E. | C.R. | P | Label |
| --- | --- | --- | --- | --- | --- | --- | --- |
| AI威胁感知 |  |  | 2.140 | .449 | 4.763 | \*\*\* |  |
| e21 |  |  | .813 | .294 | 2.761 | .006 |  |
| e23 |  |  | 1.739 | .405 | 4.294 | \*\*\* |  |
| e22 |  |  | 1.182 | .311 | 3.795 | \*\*\* |  |
| e1 |  |  | 1.143 | .213 | 5.354 | \*\*\* |  |
| e2 |  |  | 1.355 | .234 | 5.802 | \*\*\* |  |
| e3 |  |  | 1.303 | .220 | 5.925 | \*\*\* |  |
| e4 |  |  | 1.592 | .256 | 6.219 | \*\*\* |  |
| e5 |  |  | 1.304 | .233 | 5.604 | \*\*\* |  |
| e6 |  |  | 1.387 | .269 | 5.153 | \*\*\* |  |
| e8 |  |  | 1.548 | .270 | 5.730 | \*\*\* |  |
| e9 |  |  | 1.443 | .262 | 5.502 | \*\*\* |  |
| e10 |  |  | 1.461 | .263 | 5.551 | \*\*\* |  |
| e11 |  |  | 2.142 | .315 | 6.792 | \*\*\* |  |
| e12 |  |  | 1.661 | .270 | 6.160 | \*\*\* |  |
| e13 |  |  | 1.278 | .230 | 5.547 | \*\*\* |  |
| e14 |  |  | 1.093 | .235 | 4.659 | \*\*\* |  |
| e15 |  |  | 1.283 | .230 | 5.585 | \*\*\* |  |
| e16 |  |  | 1.492 | .257 | 5.799 | \*\*\* |  |
| e17 |  |  | 1.122 | .243 | 4.617 | \*\*\* |  |
| e18 |  |  | 1.303 | .256 | 5.082 | \*\*\* |  |
| e20 |  |  | 1.830 | .295 | 6.209 | \*\*\* |  |

##### Squared Multiple Correlations: (others - H4a vs H4)

|  |  |  | Estimate |
| --- | --- | --- | --- |
| AI自我效能 |  |  | .053 |
| 防御性动机 |  |  | .143 |
| 使用行为 |  |  | .253 |
| AB5 |  |  | .431 |
| AB3 |  |  | .607 |
| AB2 |  |  | .655 |
| AB1 |  |  | .515 |
| SE5 |  |  | .594 |
| SE4 |  |  | .696 |
| SE3 |  |  | .599 |
| SE2 |  |  | .491 |
| SE1 |  |  | .286 |
| DM5 |  |  | .539 |
| DM3 |  |  | .546 |
| DM2 |  |  | .512 |
| DM1 |  |  | .594 |
| AA5 |  |  | .619 |
| AA4 |  |  | .505 |
| AA3 |  |  | .567 |
| AA2 |  |  | .588 |
| AA1 |  |  | .652 |

##### User-defined estimands: (others - H4a vs H4)

|  |  |  |  |
| --- | --- | --- | --- |
| diff1 |  |  | .335 |
| diff2 |  |  | .232 |
| diff3 |  |  | .367 |
| diff4 |  |  | .244 |
| diff5 |  |  | -.005 |

##### Modification Indices (others - H4a vs H4)

##### Covariances: (others - H4a vs H4)

|  |  |  | M.I. | Par Change |
| --- | --- | --- | --- | --- |
| e20 | <--> | e21 | 5.850 | .331 |
| e17 | <--> | e23 | 5.283 | .417 |
| e14 | <--> | AI威胁感知 | 4.770 | .415 |
| e13 | <--> | e20 | 4.050 | .357 |
| e11 | <--> | e20 | 7.006 | -.560 |
| e10 | <--> | e20 | 7.991 | -.534 |
| e9 | <--> | e13 | 5.224 | -.376 |
| e5 | <--> | e16 | 4.302 | -.344 |
| e5 | <--> | e8 | 5.436 | .396 |
| e3 | <--> | e21 | 4.839 | .260 |

##### Variances: (others - H4a vs H4)

|  |  |  | M.I. | Par Change |
| --- | --- | --- | --- | --- |

##### Regression Weights: (others - H4a vs H4)

|  |  |  | M.I. | Par Change |
| --- | --- | --- | --- | --- |
| AB5 | <--- | AI自我效能 | 6.252 | .407 |
| AB5 | <--- | SE4 | 5.924 | .181 |
| AB5 | <--- | SE3 | 8.705 | .232 |
| AB5 | <--- | SE2 | 4.439 | .164 |
| AB5 | <--- | DM5 | 5.512 | -.186 |
| AB2 | <--- | 防御性动机 | 4.142 | .192 |
| AB2 | <--- | SE3 | 4.284 | -.143 |
| AB2 | <--- | DM5 | 4.631 | .149 |
| AB1 | <--- | AA5 | 5.396 | -.164 |
| SE4 | <--- | AI威胁感知 | 4.770 | .194 |
| SE4 | <--- | DM3 | 4.659 | .148 |
| SE4 | <--- | AA4 | 5.150 | .154 |
| SE4 | <--- | AA3 | 6.268 | .176 |
| SE4 | <--- | AA2 | 5.184 | .153 |
| SE3 | <--- | AA2 | 4.100 | -.137 |
| SE1 | <--- | AB5 | 4.128 | -.167 |
| DM5 | <--- | AB5 | 4.509 | -.155 |
| DM3 | <--- | SE4 | 5.252 | .158 |
| AA5 | <--- | AB1 | 4.029 | -.142 |
| AA3 | <--- | AI自我效能 | 4.524 | .299 |
| AA3 | <--- | SE4 | 5.710 | .153 |

##### Bootstrap (others - H4a vs H4)

##### Bootstrap standard errors (others - H4a vs H4)

##### Scalar Estimates (others - H4a vs H4)

##### Regression Weights: (others - H4a vs H4)

| Parameter | | | SE | SE-SE | Mean | Bias | SE-Bias |
| --- | --- | --- | --- | --- | --- | --- | --- |
| AI自我效能 | <--- | AI威胁感知 | .081 | .002 | .148 | .002 | .003 |
| 防御性动机 | <--- | AI威胁感知 | .112 | .003 | .373 | -.006 | .004 |
| 防御性动机 | <--- | AI自我效能 | .197 | .004 | -.171 | -.008 | .006 |
| 使用行为 | <--- | 防御性动机 | .061 | .001 | .388 | -.001 | .002 |
| 使用行为 | <--- | AI自我效能 | .194 | .004 | .360 | .021 | .006 |
| AA1 | <--- | AI威胁感知 | .000 | .000 | 1.000 | .000 | .000 |
| AA2 | <--- | AI威胁感知 | .104 | .002 | .951 | -.001 | .003 |
| AA3 | <--- | AI威胁感知 | .076 | .002 | .895 | .003 | .002 |
| AA4 | <--- | AI威胁感知 | .116 | .003 | .866 | -.005 | .004 |
| AA5 | <--- | AI威胁感知 | .113 | .003 | 1.000 | .005 | .004 |
| DM1 | <--- | 防御性动机 | .000 | .000 | 1.000 | .000 | .000 |
| DM2 | <--- | 防御性动机 | .135 | .003 | .901 | .007 | .004 |
| DM3 | <--- | 防御性动机 | .138 | .003 | .926 | .002 | .004 |
| DM5 | <--- | 防御性动机 | .105 | .002 | .923 | .006 | .003 |
| SE1 | <--- | AI自我效能 | .000 | .000 | 1.000 | .000 | .000 |
| SE2 | <--- | AI自我效能 | .287 | .006 | 1.407 | .039 | .009 |
| SE3 | <--- | AI自我效能 | .304 | .007 | 1.545 | .052 | .010 |
| SE4 | <--- | AI自我效能 | .339 | .008 | 1.765 | .056 | .011 |
| SE5 | <--- | AI自我效能 | .298 | .007 | 1.532 | .052 | .009 |
| AB1 | <--- | 使用行为 | .000 | .000 | 1.000 | .000 | .000 |
| AB2 | <--- | 使用行为 | .163 | .004 | 1.174 | .015 | .005 |
| AB3 | <--- | 使用行为 | .158 | .004 | 1.139 | .010 | .005 |
| AB5 | <--- | 使用行为 | .143 | .003 | .944 | .009 | .005 |

##### Standardized Regression Weights: (others - H4a vs H4)

| Parameter | | | SE | SE-SE | Mean | Bias | SE-Bias |
| --- | --- | --- | --- | --- | --- | --- | --- |
| AI自我效能 | <--- | AI威胁感知 | .117 | .003 | .232 | .001 | .004 |
| 防御性动机 | <--- | AI威胁感知 | .104 | .002 | .380 | -.009 | .003 |
| 防御性动机 | <--- | AI自我效能 | .117 | .003 | -.105 | .001 | .004 |
| 使用行为 | <--- | 防御性动机 | .077 | .002 | .442 | .001 | .002 |
| 使用行为 | <--- | AI自我效能 | .110 | .002 | .251 | .001 | .003 |
| AA1 | <--- | AI威胁感知 | .048 | .001 | .806 | -.001 | .002 |
| AA2 | <--- | AI威胁感知 | .058 | .001 | .762 | -.005 | .002 |
| AA3 | <--- | AI威胁感知 | .053 | .001 | .753 | .000 | .002 |
| AA4 | <--- | AI威胁感知 | .068 | .002 | .702 | -.009 | .002 |
| AA5 | <--- | AI威胁感知 | .049 | .001 | .785 | -.002 | .002 |
| DM1 | <--- | 防御性动机 | .057 | .001 | .770 | .000 | .002 |
| DM2 | <--- | 防御性动机 | .067 | .002 | .714 | -.001 | .002 |
| DM3 | <--- | 防御性动机 | .060 | .001 | .734 | -.005 | .002 |
| DM5 | <--- | 防御性动机 | .059 | .001 | .733 | -.001 | .002 |
| SE1 | <--- | AI自我效能 | .079 | .002 | .532 | -.003 | .002 |
| SE2 | <--- | AI自我效能 | .063 | .001 | .697 | -.004 | .002 |
| SE3 | <--- | AI自我效能 | .045 | .001 | .775 | .000 | .001 |
| SE4 | <--- | AI自我效能 | .052 | .001 | .832 | -.002 | .002 |
| SE5 | <--- | AI自我效能 | .061 | .001 | .770 | -.001 | .002 |
| AB1 | <--- | 使用行为 | .060 | .001 | .715 | -.002 | .002 |
| AB2 | <--- | 使用行为 | .059 | .001 | .810 | .001 | .002 |
| AB3 | <--- | 使用行为 | .059 | .001 | .779 | .000 | .002 |
| AB5 | <--- | 使用行为 | .068 | .002 | .655 | -.001 | .002 |

##### Variances: (others - H4a vs H4)

| Parameter | | | SE | SE-SE | Mean | Bias | SE-Bias |
| --- | --- | --- | --- | --- | --- | --- | --- |
| AI威胁感知 |  |  | .385 | .009 | 2.128 | -.012 | .012 |
| e21 |  |  | .272 | .006 | .806 | -.006 | .009 |
| e23 |  |  | .388 | .009 | 1.712 | -.027 | .012 |
| e22 |  |  | .320 | .007 | 1.167 | -.014 | .010 |
| e1 |  |  | .222 | .005 | 1.118 | -.025 | .007 |
| e2 |  |  | .256 | .006 | 1.338 | -.017 | .008 |
| e3 |  |  | .233 | .005 | 1.269 | -.035 | .007 |
| e4 |  |  | .271 | .006 | 1.585 | -.006 | .009 |
| e5 |  |  | .238 | .005 | 1.280 | -.025 | .008 |
| e6 |  |  | .268 | .006 | 1.357 | -.030 | .008 |
| e8 |  |  | .313 | .007 | 1.526 | -.023 | .010 |
| e9 |  |  | .231 | .005 | 1.423 | -.020 | .007 |
| e10 |  |  | .260 | .006 | 1.437 | -.024 | .008 |
| e11 |  |  | .277 | .006 | 2.099 | -.044 | .009 |
| e12 |  |  | .254 | .006 | 1.639 | -.022 | .008 |
| e13 |  |  | .217 | .005 | 1.250 | -.028 | .007 |
| e14 |  |  | .269 | .006 | 1.074 | -.019 | .009 |
| e15 |  |  | .255 | .006 | 1.255 | -.027 | .008 |
| e16 |  |  | .232 | .005 | 1.474 | -.018 | .007 |
| e17 |  |  | .307 | .007 | 1.097 | -.025 | .010 |
| e18 |  |  | .296 | .007 | 1.271 | -.032 | .009 |
| e20 |  |  | .289 | .006 | 1.803 | -.026 | .009 |

##### Squared Multiple Correlations: (others - H4a vs H4)

| Parameter | | | SE | SE-SE | Mean | Bias | SE-Bias |
| --- | --- | --- | --- | --- | --- | --- | --- |
| AI自我效能 |  |  | .054 | .001 | .067 | .014 | .002 |
| 防御性动机 |  |  | .075 | .002 | .160 | .017 | .002 |
| 使用行为 |  |  | .080 | .002 | .270 | .017 | .003 |
| AB5 |  |  | .087 | .002 | .434 | .003 | .003 |
| AB3 |  |  | .091 | .002 | .611 | .004 | .003 |
| AB2 |  |  | .095 | .002 | .659 | .005 | .003 |
| AB1 |  |  | .085 | .002 | .515 | .000 | .003 |
| SE5 |  |  | .092 | .002 | .597 | .003 | .003 |
| SE4 |  |  | .085 | .002 | .696 | -.001 | .003 |
| SE3 |  |  | .070 | .002 | .602 | .003 | .002 |
| SE2 |  |  | .086 | .002 | .490 | -.001 | .003 |
| SE1 |  |  | .082 | .002 | .289 | .003 | .003 |
| DM5 |  |  | .085 | .002 | .541 | .003 | .003 |
| DM3 |  |  | .086 | .002 | .543 | -.003 | .003 |
| DM2 |  |  | .095 | .002 | .514 | .003 | .003 |
| DM1 |  |  | .087 | .002 | .597 | .003 | .003 |
| AA5 |  |  | .076 | .002 | .619 | .000 | .002 |
| AA4 |  |  | .094 | .002 | .497 | -.008 | .003 |
| AA3 |  |  | .079 | .002 | .570 | .003 | .003 |
| AA2 |  |  | .087 | .002 | .584 | -.004 | .003 |
| AA1 |  |  | .077 | .002 | .652 | .001 | .002 |

##### User-defined estimands: (others - H4a vs H4)

| Parameter | | | SE | SE-SE | Mean | Bias | SE-Bias |
| --- | --- | --- | --- | --- | --- | --- | --- |
| diff1 |  |  | .100 | .002 | .333 | -.002 | .003 |
| diff2 |  |  | .108 | .002 | .232 | .000 | .003 |
| diff3 |  |  | .116 | .003 | .363 | -.004 | .004 |
| diff4 |  |  | .102 | .002 | .240 | -.004 | .003 |
| diff5 |  |  | .123 | .003 | -.008 | -.003 | .004 |

##### Bootstrap Confidence (others - H4a vs H4)

##### Percentile method (others - H4a vs H4)

##### 95% confidence intervals (percentile method)

##### Scalar Estimates (others - H4a vs H4)

##### Regression Weights: (others - H4a vs H4)

| Parameter | | | Estimate | Lower | Upper | P |
| --- | --- | --- | --- | --- | --- | --- |
| AI自我效能 | <--- | AI威胁感知 | .146 | -.010 | .319 | .063 |
| 防御性动机 | <--- | AI威胁感知 | .379 | .157 | .601 | .003 |
| 防御性动机 | <--- | AI自我效能 | -.163 | -.599 | .222 | .368 |
| 使用行为 | <--- | 防御性动机 | .390 | .270 | .515 | .002 |
| 使用行为 | <--- | AI自我效能 | .339 | .037 | .811 | .030 |
| AA1 | <--- | AI威胁感知 | 1.000 | 1.000 | 1.000 | ... |
| AA2 | <--- | AI威胁感知 | .952 | .745 | 1.161 | .002 |
| AA3 | <--- | AI威胁感知 | .893 | .747 | 1.051 | .002 |
| AA4 | <--- | AI威胁感知 | .871 | .652 | 1.104 | .002 |
| AA5 | <--- | AI威胁感知 | .995 | .801 | 1.234 | .002 |
| DM1 | <--- | 防御性动机 | 1.000 | 1.000 | 1.000 | ... |
| DM2 | <--- | 防御性动机 | .894 | .669 | 1.189 | .002 |
| DM3 | <--- | 防御性动机 | .924 | .662 | 1.217 | .002 |
| DM5 | <--- | 防御性动机 | .917 | .733 | 1.131 | .002 |
| SE1 | <--- | AI自我效能 | 1.000 | 1.000 | 1.000 | ... |
| SE2 | <--- | AI自我效能 | 1.368 | .946 | 2.065 | .002 |
| SE3 | <--- | AI自我效能 | 1.493 | 1.122 | 2.222 | .002 |
| SE4 | <--- | AI自我效能 | 1.709 | 1.277 | 2.547 | .002 |
| SE5 | <--- | AI自我效能 | 1.479 | 1.060 | 2.221 | .002 |
| AB1 | <--- | 使用行为 | 1.000 | 1.000 | 1.000 | ... |
| AB2 | <--- | 使用行为 | 1.159 | .910 | 1.535 | .002 |
| AB3 | <--- | 使用行为 | 1.129 | .881 | 1.493 | .002 |
| AB5 | <--- | 使用行为 | .935 | .685 | 1.234 | .002 |

##### Standardized Regression Weights: (others - H4a vs H4)

| Parameter | | | Estimate | Lower | Upper | P |
| --- | --- | --- | --- | --- | --- | --- |
| AI自我效能 | <--- | AI威胁感知 | .230 | -.015 | .446 | .063 |
| 防御性动机 | <--- | AI威胁感知 | .389 | .162 | .571 | .003 |
| 防御性动机 | <--- | AI自我效能 | -.106 | -.318 | .134 | .368 |
| 使用行为 | <--- | 防御性动机 | .441 | .295 | .595 | .002 |
| 使用行为 | <--- | AI自我效能 | .250 | .031 | .462 | .030 |
| AA1 | <--- | AI威胁感知 | .807 | .703 | .891 | .002 |
| AA2 | <--- | AI威胁感知 | .767 | .629 | .861 | .002 |
| AA3 | <--- | AI威胁感知 | .753 | .645 | .845 | .002 |
| AA4 | <--- | AI威胁感知 | .710 | .549 | .821 | .002 |
| AA5 | <--- | AI威胁感知 | .787 | .677 | .868 | .002 |
| DM1 | <--- | 防御性动机 | .771 | .655 | .872 | .002 |
| DM2 | <--- | 防御性动机 | .715 | .577 | .833 | .002 |
| DM3 | <--- | 防御性动机 | .739 | .606 | .839 | .002 |
| DM5 | <--- | 防御性动机 | .734 | .602 | .836 | .002 |
| SE1 | <--- | AI自我效能 | .535 | .369 | .671 | .002 |
| SE2 | <--- | AI自我效能 | .701 | .557 | .812 | .002 |
| SE3 | <--- | AI自我效能 | .774 | .680 | .857 | .002 |
| SE4 | <--- | AI自我效能 | .834 | .723 | .921 | .002 |
| SE5 | <--- | AI自我效能 | .771 | .633 | .879 | .002 |
| AB1 | <--- | 使用行为 | .717 | .590 | .827 | .002 |
| AB2 | <--- | 使用行为 | .809 | .687 | .918 | .002 |
| AB3 | <--- | 使用行为 | .779 | .651 | .879 | .002 |
| AB5 | <--- | 使用行为 | .656 | .513 | .777 | .002 |

##### Variances: (others - H4a vs H4)

| Parameter | | | Estimate | Lower | Upper | P |
| --- | --- | --- | --- | --- | --- | --- |
| AI威胁感知 |  |  | 2.140 | 1.410 | 2.908 | .002 |
| e21 |  |  | .813 | .342 | 1.372 | .002 |
| e23 |  |  | 1.739 | 1.027 | 2.535 | .002 |
| e22 |  |  | 1.182 | .633 | 1.906 | .002 |
| e1 |  |  | 1.143 | .698 | 1.587 | .002 |
| e2 |  |  | 1.355 | .877 | 1.893 | .002 |
| e3 |  |  | 1.303 | .843 | 1.768 | .002 |
| e4 |  |  | 1.592 | 1.081 | 2.147 | .002 |
| e5 |  |  | 1.304 | .829 | 1.785 | .002 |
| e6 |  |  | 1.387 | .833 | 1.869 | .002 |
| e8 |  |  | 1.548 | .951 | 2.187 | .002 |
| e9 |  |  | 1.443 | .956 | 1.876 | .002 |
| e10 |  |  | 1.461 | .980 | 1.978 | .002 |
| e11 |  |  | 2.142 | 1.581 | 2.631 | .002 |
| e12 |  |  | 1.661 | 1.178 | 2.155 | .002 |
| e13 |  |  | 1.278 | .842 | 1.693 | .002 |
| e14 |  |  | 1.093 | .545 | 1.587 | .002 |
| e15 |  |  | 1.283 | .750 | 1.776 | .002 |
| e16 |  |  | 1.492 | 1.008 | 1.938 | .002 |
| e17 |  |  | 1.122 | .489 | 1.683 | .002 |
| e18 |  |  | 1.303 | .733 | 1.849 | .002 |
| e20 |  |  | 1.830 | 1.262 | 2.385 | .002 |

##### Squared Multiple Correlations: (others - H4a vs H4)

| Parameter | | | Estimate | Lower | Upper | P |
| --- | --- | --- | --- | --- | --- | --- |
| AI自我效能 |  |  | .053 | .001 | .199 | .002 |
| 防御性动机 |  |  | .143 | .037 | .324 | .002 |
| 使用行为 |  |  | .253 | .130 | .438 | .002 |
| AB5 |  |  | .431 | .263 | .603 | .002 |
| AB3 |  |  | .607 | .424 | .773 | .002 |
| AB2 |  |  | .655 | .472 | .843 | .002 |
| AB1 |  |  | .515 | .348 | .684 | .002 |
| SE5 |  |  | .594 | .401 | .773 | .002 |
| SE4 |  |  | .696 | .522 | .847 | .002 |
| SE3 |  |  | .599 | .462 | .735 | .002 |
| SE2 |  |  | .491 | .310 | .660 | .002 |
| SE1 |  |  | .286 | .136 | .450 | .002 |
| DM5 |  |  | .539 | .362 | .699 | .002 |
| DM3 |  |  | .546 | .368 | .703 | .002 |
| DM2 |  |  | .512 | .333 | .694 | .002 |
| DM1 |  |  | .594 | .429 | .760 | .002 |
| AA5 |  |  | .619 | .458 | .753 | .002 |
| AA4 |  |  | .505 | .301 | .674 | .002 |
| AA3 |  |  | .567 | .416 | .714 | .002 |
| AA2 |  |  | .588 | .396 | .741 | .002 |
| AA1 |  |  | .652 | .494 | .794 | .002 |

##### User-defined estimands: (others - H4a vs H4)

| Parameter | | | Estimate | Lower | Upper | P |
| --- | --- | --- | --- | --- | --- | --- |
| diff1 |  |  | .335 | .131 | .526 | .002 |
| diff2 |  |  | .232 | .017 | .448 | .029 |
| diff3 |  |  | .367 | .127 | .590 | .002 |
| diff4 |  |  | .244 | .030 | .432 | .018 |
| diff5 |  |  | -.005 | -.262 | .227 | .963 |

##### Bias-corrected percentile method (others - H4a vs H4)

##### 95% confidence intervals (bias-corrected percentile method)

##### Scalar Estimates (others - H4a vs H4)

##### Regression Weights: (others - H4a vs H4)

| Parameter | | | Estimate | Lower | Upper | P |
| --- | --- | --- | --- | --- | --- | --- |
| AI自我效能 | <--- | AI威胁感知 | .146 | -.010 | .318 | .068 |
| 防御性动机 | <--- | AI威胁感知 | .379 | .182 | .628 | .001 |
| 防御性动机 | <--- | AI自我效能 | -.163 | -.600 | .221 | .365 |
| 使用行为 | <--- | 防御性动机 | .390 | .272 | .516 | .002 |
| 使用行为 | <--- | AI自我效能 | .339 | .044 | .832 | .026 |
| AA1 | <--- | AI威胁感知 | 1.000 | 1.000 | 1.000 | ... |
| AA2 | <--- | AI威胁感知 | .952 | .770 | 1.190 | .001 |
| AA3 | <--- | AI威胁感知 | .893 | .739 | 1.045 | .003 |
| AA4 | <--- | AI威胁感知 | .871 | .674 | 1.123 | .001 |
| AA5 | <--- | AI威胁感知 | .995 | .804 | 1.235 | .002 |
| DM1 | <--- | 防御性动机 | 1.000 | 1.000 | 1.000 | ... |
| DM2 | <--- | 防御性动机 | .894 | .678 | 1.211 | .002 |
| DM3 | <--- | 防御性动机 | .924 | .671 | 1.226 | .002 |
| DM5 | <--- | 防御性动机 | .917 | .735 | 1.132 | .002 |
| SE1 | <--- | AI自我效能 | 1.000 | 1.000 | 1.000 | ... |
| SE2 | <--- | AI自我效能 | 1.368 | .953 | 2.091 | .002 |
| SE3 | <--- | AI自我效能 | 1.493 | 1.121 | 2.203 | .002 |
| SE4 | <--- | AI自我效能 | 1.709 | 1.276 | 2.542 | .002 |
| SE5 | <--- | AI自我效能 | 1.479 | 1.038 | 2.142 | .003 |
| AB1 | <--- | 使用行为 | 1.000 | 1.000 | 1.000 | ... |
| AB2 | <--- | 使用行为 | 1.159 | .916 | 1.538 | .002 |
| AB3 | <--- | 使用行为 | 1.129 | .883 | 1.496 | .002 |
| AB5 | <--- | 使用行为 | .935 | .680 | 1.218 | .002 |

##### Standardized Regression Weights: (others - H4a vs H4)

| Parameter | | | Estimate | Lower | Upper | P |
| --- | --- | --- | --- | --- | --- | --- |
| AI自我效能 | <--- | AI威胁感知 | .230 | -.020 | .434 | .076 |
| 防御性动机 | <--- | AI威胁感知 | .389 | .183 | .580 | .002 |
| 防御性动机 | <--- | AI自我效能 | -.106 | -.319 | .130 | .347 |
| 使用行为 | <--- | 防御性动机 | .441 | .294 | .592 | .002 |
| 使用行为 | <--- | AI自我效能 | .250 | .016 | .453 | .036 |
| AA1 | <--- | AI威胁感知 | .807 | .691 | .886 | .003 |
| AA2 | <--- | AI威胁感知 | .767 | .630 | .861 | .002 |
| AA3 | <--- | AI威胁感知 | .753 | .627 | .840 | .003 |
| AA4 | <--- | AI威胁感知 | .710 | .563 | .828 | .001 |
| AA5 | <--- | AI威胁感知 | .787 | .669 | .864 | .003 |
| DM1 | <--- | 防御性动机 | .771 | .653 | .870 | .002 |
| DM2 | <--- | 防御性动机 | .715 | .574 | .832 | .002 |
| DM3 | <--- | 防御性动机 | .739 | .606 | .838 | .002 |
| DM5 | <--- | 防御性动机 | .734 | .587 | .831 | .003 |
| SE1 | <--- | AI自我效能 | .535 | .368 | .668 | .002 |
| SE2 | <--- | AI自我效能 | .701 | .554 | .811 | .002 |
| SE3 | <--- | AI自我效能 | .774 | .672 | .853 | .003 |
| SE4 | <--- | AI自我效能 | .834 | .711 | .918 | .003 |
| SE5 | <--- | AI自我效能 | .771 | .628 | .876 | .003 |
| AB1 | <--- | 使用行为 | .717 | .592 | .827 | .002 |
| AB2 | <--- | 使用行为 | .809 | .681 | .913 | .003 |
| AB3 | <--- | 使用行为 | .779 | .631 | .872 | .004 |
| AB5 | <--- | 使用行为 | .656 | .502 | .769 | .003 |

##### Variances: (others - H4a vs H4)

| Parameter | | | Estimate | Lower | Upper | P |
| --- | --- | --- | --- | --- | --- | --- |
| AI威胁感知 |  |  | 2.140 | 1.426 | 2.923 | .002 |
| e21 |  |  | .813 | .374 | 1.459 | .001 |
| e23 |  |  | 1.739 | 1.093 | 2.649 | .001 |
| e22 |  |  | 1.182 | .700 | 2.050 | .001 |
| e1 |  |  | 1.143 | .754 | 1.646 | .001 |
| e2 |  |  | 1.355 | .905 | 1.929 | .001 |
| e3 |  |  | 1.303 | .910 | 1.835 | .001 |
| e4 |  |  | 1.592 | 1.109 | 2.175 | .001 |
| e5 |  |  | 1.304 | .889 | 1.850 | .001 |
| e6 |  |  | 1.387 | .925 | 1.967 | .001 |
| e8 |  |  | 1.548 | .998 | 2.225 | .001 |
| e9 |  |  | 1.443 | 1.001 | 1.915 | .001 |
| e10 |  |  | 1.461 | 1.032 | 2.083 | .001 |
| e11 |  |  | 2.142 | 1.651 | 2.723 | .001 |
| e12 |  |  | 1.661 | 1.224 | 2.220 | .001 |
| e13 |  |  | 1.278 | .877 | 1.782 | .001 |
| e14 |  |  | 1.093 | .563 | 1.611 | .002 |
| e15 |  |  | 1.283 | .823 | 1.825 | .001 |
| e16 |  |  | 1.492 | 1.047 | 1.988 | .001 |
| e17 |  |  | 1.122 | .531 | 1.735 | .001 |
| e18 |  |  | 1.303 | .809 | 1.926 | .001 |
| e20 |  |  | 1.830 | 1.288 | 2.431 | .001 |

##### Squared Multiple Correlations: (others - H4a vs H4)

| Parameter | | | Estimate | Lower | Upper | P |
| --- | --- | --- | --- | --- | --- | --- |
| AI自我效能 |  |  | .053 | .000 | .188 | .003 |
| 防御性动机 |  |  | .143 | .027 | .304 | .004 |
| 使用行为 |  |  | .253 | .115 | .408 | .005 |
| AB5 |  |  | .431 | .252 | .592 | .003 |
| AB3 |  |  | .607 | .398 | .760 | .004 |
| AB2 |  |  | .655 | .464 | .834 | .003 |
| AB1 |  |  | .515 | .350 | .684 | .002 |
| SE5 |  |  | .594 | .394 | .767 | .003 |
| SE4 |  |  | .696 | .505 | .842 | .003 |
| SE3 |  |  | .599 | .452 | .728 | .003 |
| SE2 |  |  | .491 | .306 | .657 | .002 |
| SE1 |  |  | .286 | .136 | .446 | .002 |
| DM5 |  |  | .539 | .345 | .691 | .003 |
| DM3 |  |  | .546 | .368 | .702 | .002 |
| DM2 |  |  | .512 | .330 | .693 | .002 |
| DM1 |  |  | .594 | .426 | .756 | .002 |
| AA5 |  |  | .619 | .447 | .746 | .003 |
| AA4 |  |  | .505 | .316 | .686 | .001 |
| AA3 |  |  | .567 | .393 | .706 | .003 |
| AA2 |  |  | .588 | .397 | .742 | .002 |
| AA1 |  |  | .652 | .477 | .786 | .003 |

##### User-defined estimands: (others - H4a vs H4)

| Parameter | | | Estimate | Lower | Upper | P |
| --- | --- | --- | --- | --- | --- | --- |
| diff1 |  |  | .335 | .125 | .526 | .002 |
| diff2 |  |  | .232 | .025 | .452 | .026 |
| diff3 |  |  | .367 | .130 | .600 | .002 |
| diff4 |  |  | .244 | .042 | .448 | .013 |
| diff5 |  |  | -.005 | -.259 | .231 | .991 |

##### Minimization History (H4a vs H4)

| Iteration |  | Negative eigenvalues | Condition # | Smallest eigenvalue | Diameter | F | NTries | Ratio |
| --- | --- | --- | --- | --- | --- | --- | --- | --- |
| 0 | e | 11 |  | -.651 | 9999.000 | 3579.763 | 0 | 9999.000 |
| 1 | e | 4 |  | -.076 | 3.413 | 1313.729 | 20 | .391 |
| 2 | e | 0 | 220.191 |  | 2.433 | 502.679 | 5 | .677 |
| 3 | e | 0 | 89.246 |  | 1.519 | 346.179 | 1 | .926 |
| 4 | e | 0 | 97.257 |  | .627 | 293.249 | 1 | 1.126 |
| 5 | e | 0 | 138.446 |  | .369 | 289.290 | 1 | 1.093 |
| 6 | e | 0 | 199.575 |  | .143 | 289.075 | 1 | 1.088 |
| 7 | e | 0 | 220.081 |  | .042 | 289.068 | 1 | 1.033 |
| 8 | e | 0 | 220.081 |  | .002 | 289.068 | 1 | 1.002 |
| 9 | e | 0 | 220.081 |  | .000 | 289.068 | 1 | 1.000 |

##### Bootstrap (H4a vs H4)

##### Summary of Bootstrap Iterations (H4a vs H4)

##### (H4a vs H4)

| Iterations | Method 0 | Method 1 | Method 2 |
| --- | --- | --- | --- |
| 1 | 0 | 0 | 0 |
| 2 | 0 | 0 | 0 |
| 3 | 0 | 0 | 0 |
| 4 | 0 | 0 | 0 |
| 5 | 0 | 0 | 0 |
| 6 | 0 | 0 | 0 |
| 7 | 0 | 21 | 0 |
| 8 | 0 | 76 | 0 |
| 9 | 0 | 174 | 0 |
| 10 | 0 | 162 | 0 |
| 11 | 0 | 169 | 0 |
| 12 | 0 | 133 | 0 |
| 13 | 0 | 112 | 0 |
| 14 | 0 | 54 | 0 |
| 15 | 0 | 37 | 0 |
| 16 | 0 | 20 | 0 |
| 17 | 0 | 14 | 0 |
| 18 | 0 | 8 | 0 |
| 19 | 0 | 20 | 0 |
| Total | 0 | 1000 | 0 |

0 bootstrap samples were unused because of a singular covariance matrix.

0 bootstrap samples were unused because a solution was not found.

1000 usable bootstrap samples were obtained.

##### Bootstrap Distributions (H4a vs H4)

##### ML discrepancy (implied vs sample) (H4a vs H4)

|  |  |  |
| --- | --- | --- |
|  |  | \|-------------------- |
|  | 441.193 | \|\* |
|  | 461.770 | \|\* |
|  | 482.346 | \|\*\*\*\* |
|  | 502.923 | \|\*\*\*\*\*\*\*\* |
|  | 523.499 | \|\*\*\*\*\*\*\*\*\*\*\*\*\*\* |
|  | 544.076 | \|\*\*\*\*\*\*\*\*\*\*\*\*\*\*\*\*\*\*\*\* |
|  | 564.653 | \|\*\*\*\*\*\*\*\*\*\*\*\*\*\*\*\*\*\*\*\* |
| N = 1000 | 585.229 | \|\*\*\*\*\*\*\*\*\*\*\*\*\*\*\*\*\*\*\*\* |
| Mean = 574.426 | 605.806 | \|\*\*\*\*\*\*\*\*\*\*\*\*\*\*\*\*\*\*\* |
| S. e. = 1.455 | 626.382 | \|\*\*\*\*\*\*\*\*\*\*\*\*\* |
|  | 646.959 | \|\*\*\*\*\*\* |
|  | 667.535 | \|\*\*\*\* |
|  | 688.112 | \|\*\* |
|  | 708.688 | \|\* |
|  | 729.265 | \|\* |
|  |  | \|-------------------- |

##### ML discrepancy (implied vs pop) (H4a vs H4)

|  |  |  |
| --- | --- | --- |
|  |  | \|-------------------- |
|  | 351.483 | \|\* |
|  | 359.518 | \|\*\* |
|  | 367.552 | \|\*\*\*\* |
|  | 375.587 | \|\*\*\*\*\*\*\*\*\*\*\*\* |
|  | 383.622 | \|\*\*\*\*\*\*\*\*\*\*\*\*\*\*\*\* |
|  | 391.656 | \|\*\*\*\*\*\*\*\*\*\*\*\*\*\*\*\*\*\*\* |
|  | 399.691 | \|\*\*\*\*\*\*\*\*\*\*\*\*\*\*\*\*\*\*\*\* |
| N = 1000 | 407.726 | \|\*\*\*\*\*\*\*\*\*\*\*\*\*\*\*\* |
| Mean = 398.759 | 415.760 | \|\*\*\*\*\*\*\*\*\* |
| S. e. = .595 | 423.795 | \|\*\*\*\*\*\*\*\* |
|  | 431.830 | \|\*\*\*\*\* |
|  | 439.864 | \|\*\*\* |
|  | 447.899 | \|\* |
|  | 455.934 | \|\* |
|  | 463.968 | \|\* |
|  |  | \|-------------------- |

##### K-L overoptimism (unstabilized) (H4a vs H4)

|  |  |  |
| --- | --- | --- |
|  |  | \|-------------------- |
|  | -143.598 | \|\* |
|  | -93.354 | \| |
|  | -43.109 | \|\*\* |
|  | 7.135 | \|\*\*\*\* |
|  | 57.380 | \|\*\*\*\*\*\*\*\*\*\* |
|  | 107.624 | \|\*\*\*\*\*\*\*\*\*\*\*\*\*\* |
|  | 157.868 | \|\*\*\*\*\*\*\*\*\*\*\*\*\*\*\*\*\*\* |
| N = 1000 | 208.113 | \|\*\*\*\*\*\*\*\*\*\*\*\*\*\*\*\*\*\*\* |
| Mean = 211.891 | 258.357 | \|\*\*\*\*\*\*\*\*\*\*\*\*\*\*\*\*\*\* |
| S. e. = 3.689 | 308.602 | \|\*\*\*\*\*\*\*\*\*\*\*\* |
|  | 358.846 | \|\*\*\*\*\*\*\*\* |
|  | 409.091 | \|\*\*\*\*\* |
|  | 459.335 | \|\*\*\* |
|  | 509.579 | \|\*\* |
|  | 559.824 | \|\* |
|  |  | \|-------------------- |

##### K-L overoptimism (stabilized) (H4a vs H4)

|  |  |  |
| --- | --- | --- |
|  |  | \|-------------------- |
|  | 88.881 | \|\* |
|  | 107.098 | \|\* |
|  | 125.315 | \|\* |
|  | 143.532 | \|\*\*\*\*\*\* |
|  | 161.750 | \|\*\*\*\*\*\*\*\*\* |
|  | 179.967 | \|\*\*\*\*\*\*\*\*\*\*\*\*\*\* |
|  | 198.184 | \|\*\*\*\*\*\*\*\*\*\*\*\*\*\*\*\*\*\*\* |
| N = 1000 | 216.402 | \|\*\*\*\*\*\*\*\*\*\*\*\*\*\*\*\*\* |
| Mean = 213.288 | 234.619 | \|\*\*\*\*\*\*\*\*\*\*\*\*\* |
| S. e. = 1.345 | 252.836 | \|\*\*\*\*\*\*\*\*\*\* |
|  | 271.054 | \|\*\*\*\*\*\*\*\* |
|  | 289.271 | \|\*\*\*\* |
|  | 307.488 | \|\*\*\* |
|  | 325.705 | \|\* |
|  | 343.923 | \|\* |
|  |  | \|-------------------- |

##### Model Fit Summary

##### CMIN

| Model | NPAR | CMIN | DF | P | CMIN/DF |
| --- | --- | --- | --- | --- | --- |
| Default model | 82 | 289.057 | 260 | .104 | 1.112 |
| H1a vs H1 | 81 | 298.753 | 261 | .054 | 1.145 |
| H2a vs H2 | 81 | 289.057 | 261 | .112 | 1.107 |
| H3a vs H3 | 81 | 289.909 | 261 | .106 | 1.111 |
| H4a vs H4 | 81 | 289.068 | 261 | .112 | 1.108 |
| Saturated model | 342 | .000 | 0 |  |  |
| Independence model | 36 | 3428.105 | 306 | .000 | 11.203 |

##### RMR, GFI

| Model | RMR | GFI | AGFI | PGFI |
| --- | --- | --- | --- | --- |
| Default model | .179 | .926 | .902 | .704 |
| H1a vs H1 | .252 | .924 | .900 | .705 |
| H2a vs H2 | .179 | .926 | .903 | .707 |
| H3a vs H3 | .182 | .926 | .903 | .707 |
| H4a vs H4 | .179 | .926 | .903 | .707 |
| Saturated model | .000 | 1.000 |  |  |
| Independence model | 1.031 | .326 | .247 | .292 |

##### Baseline Comparisons

| Model | NFI Delta1 | RFI rho1 | IFI Delta2 | TLI rho2 | CFI |
| --- | --- | --- | --- | --- | --- |
| Default model | .916 | .901 | .991 | .989 | .991 |
| H1a vs H1 | .913 | .898 | .988 | .986 | .988 |
| H2a vs H2 | .916 | .901 | .991 | .989 | .991 |
| H3a vs H3 | .915 | .901 | .991 | .989 | .991 |
| H4a vs H4 | .916 | .901 | .991 | .989 | .991 |
| Saturated model | 1.000 |  | 1.000 |  | 1.000 |
| Independence model | .000 | .000 | .000 | .000 | .000 |

##### Parsimony-Adjusted Measures

| Model | PRATIO | PNFI | PCFI |
| --- | --- | --- | --- |
| Default model | .850 | .778 | .842 |
| H1a vs H1 | .853 | .779 | .843 |
| H2a vs H2 | .853 | .781 | .845 |
| H3a vs H3 | .853 | .781 | .845 |
| H4a vs H4 | .853 | .781 | .845 |
| Saturated model | .000 | .000 | .000 |
| Independence model | 1.000 | .000 | .000 |

##### NCP

| Model | NCP | LO 90 | HI 90 |
| --- | --- | --- | --- |
| Default model | 29.057 | .000 | 74.747 |
| H1a vs H1 | 37.753 | .000 | 84.602 |
| H2a vs H2 | 28.057 | .000 | 73.685 |
| H3a vs H3 | 28.909 | .000 | 74.645 |
| H4a vs H4 | 28.068 | .000 | 73.698 |
| Saturated model | .000 | .000 | .000 |
| Independence model | 3122.105 | 2937.440 | 3314.108 |

##### FMIN

| Model | FMIN | F0 | LO 90 | HI 90 |
| --- | --- | --- | --- | --- |
| Default model | .751 | .075 | .000 | .194 |
| H1a vs H1 | .776 | .098 | .000 | .220 |
| H2a vs H2 | .751 | .073 | .000 | .191 |
| H3a vs H3 | .753 | .075 | .000 | .194 |
| H4a vs H4 | .751 | .073 | .000 | .191 |
| Saturated model | .000 | .000 | .000 | .000 |
| Independence model | 8.904 | 8.109 | 7.630 | 8.608 |

##### RMSEA

| Model | RMSEA | LO 90 | HI 90 | PCLOSE |
| --- | --- | --- | --- | --- |
| Default model | .017 | .000 | .027 | 1.000 |
| H1a vs H1 | .019 | .000 | .029 | 1.000 |
| H2a vs H2 | .017 | .000 | .027 | 1.000 |
| H3a vs H3 | .017 | .000 | .027 | 1.000 |
| H4a vs H4 | .017 | .000 | .027 | 1.000 |
| Independence model | .163 | .158 | .168 | .000 |

##### AIC

| Model | AIC | BCC | BIC | CAIC |
| --- | --- | --- | --- | --- |
| Default model | 453.057 | 477.631 |  |  |
| H1a vs H1 | 460.753 | 485.027 |  |  |
| H2a vs H2 | 451.057 | 475.331 |  |  |
| H3a vs H3 | 451.909 | 476.183 |  |  |
| H4a vs H4 | 451.068 | 475.343 |  |  |
| Saturated model | 684.000 | 786.492 |  |  |
| Independence model | 3500.105 | 3510.893 |  |  |

##### ECVI

| Model | ECVI | LO 90 | HI 90 | MECVI |
| --- | --- | --- | --- | --- |
| Default model | 1.177 | 1.101 | 1.295 | 1.241 |
| H1a vs H1 | 1.197 | 1.099 | 1.318 | 1.260 |
| H2a vs H2 | 1.172 | 1.099 | 1.290 | 1.235 |
| H3a vs H3 | 1.174 | 1.099 | 1.293 | 1.237 |
| H4a vs H4 | 1.172 | 1.099 | 1.290 | 1.235 |
| Saturated model | 1.777 | 1.777 | 1.777 | 2.043 |
| Independence model | 9.091 | 8.612 | 9.590 | 9.119 |

##### HOELTER

| Model | HOELTER .05 | HOELTER .01 |
| --- | --- | --- |
| Default model | 399 | 422 |
| H1a vs H1 | 388 | 410 |
| H2a vs H2 | 401 | 424 |
| H3a vs H3 | 399 | 423 |
| H4a vs H4 | 401 | 424 |
| Independence model | 41 | 43 |

##### Nested Model Comparisons

##### Assuming model Default model to be correct:

|  |  |  |  |  |  |  |  |
| --- | --- | --- | --- | --- | --- | --- | --- |
| Model | DF | CMIN | P | NFI Delta-1 | IFI Delta-2 | RFI rho-1 | TLI rho2 |
| H1a vs H1 | 1 | 9.696 | .002 | .003 | .003 | .003 | .003 |
| H2a vs H2 | 1 | .000 | .997 | .000 | .000 | .000 | .000 |
| H3a vs H3 | 1 | .852 | .356 | .000 | .000 | .000 | .000 |
| H4a vs H4 | 1 | .012 | .914 | .000 | .000 | .000 | .000 |

##### Execution time summary

|  |  |
| --- | --- |
| Minimization: | .018 |
| Miscellaneous: | 4.990 |
| Bootstrap: | 1.165 |
| Total: | 6.173 |
