# Supplementary material for: How AI threat perception relates to post-adoptive AI agent engagement: the dual paths of self-efficacy and defensive motivation
Source: Front Psychol. 2026 Oct 22;17:1887571. doi: 10.3389/fpsyg.2026.1887571 (PMC13735560; doi:10.3389/fpsyg.2026.1887571)
Supplement: Supplementary file 1 [file Data_Sheet_1.zip › log+Questionnaire/006_Measurement_Invariance_Region.AmosOutput]

多群组比较模型-地区.amw


#### D:\学术研究\066研究数据与论文撰写\01研究数据\AI智能体论文\多群组比较\多群组比较模型-地区.amw

##### Analysis Summary

##### Date and Time

Date: 2026年9月13日

Time: 12:36:20

##### Title

多群组比较模型-地区: 2026年9月13日 12:36

##### Groups

##### Group number 1 (Group number 1)

##### Notes for Group (Group number 1)

The model is recursive.

Sample size = 157

##### Variable Summary (east)

##### Your model contains the following variables (east)

Observed, endogenous variables

AA1

AA2

AA3

AA4

AA5

DM1

DM2

DM3

DM5

SE1

SE2

SE3

SE4

SE5

AB1

AB2

AB3

AB5

Unobserved, endogenous variables

防御性动机

AI自我效能

使用行为

Unobserved, exogenous variables

AI威胁感知

e1

e2

e3

e4

e5

e6

e8

e9

e10

e11

e12

e13

e14

e15

e16

e17

e18

e20

e21

e22

e23

##### Variable counts (east)

|  |  |
| --- | --- |
| Number of variables in your model: | 43 |
| Number of observed variables: | 18 |
| Number of unobserved variables: | 25 |
| Number of exogenous variables: | 22 |
| Number of endogenous variables: | 21 |

##### Parameter Summary (east)

|  | Weights | Covariances | Variances | Means | Intercepts | Total |
| --- | --- | --- | --- | --- | --- | --- |
| Fixed | 25 | 0 | 0 | 0 | 0 | 25 |
| Labeled | 19 | 0 | 22 | 0 | 0 | 41 |
| Unlabeled | 0 | 0 | 0 | 0 | 0 | 0 |
| Total | 44 | 0 | 22 | 0 | 0 | 66 |

##### Assessment of normality (east)

| Variable | min | max | skew | c.r. | kurtosis | c.r. |
| --- | --- | --- | --- | --- | --- | --- |
| AB5 | 1.000 | 7.000 | .016 | .084 | -1.081 | -2.765 |
| AB3 | 1.000 | 7.000 | .072 | .370 | -1.028 | -2.629 |
| AB2 | 1.000 | 7.000 | .025 | .126 | -1.079 | -2.760 |
| AB1 | 1.000 | 7.000 | .064 | .328 | -1.218 | -3.114 |
| SE5 | 1.000 | 7.000 | .031 | .161 | -1.113 | -2.847 |
| SE4 | 1.000 | 7.000 | .001 | .003 | -1.205 | -3.081 |
| SE3 | 1.000 | 7.000 | -.003 | -.014 | -1.146 | -2.932 |
| SE2 | 1.000 | 7.000 | .004 | .019 | -1.211 | -3.098 |
| SE1 | 1.000 | 7.000 | .222 | 1.135 | -1.016 | -2.599 |
| DM5 | 1.000 | 7.000 | .140 | .718 | -1.039 | -2.658 |
| DM3 | 1.000 | 7.000 | .062 | .316 | -1.100 | -2.814 |
| DM2 | 1.000 | 7.000 | .028 | .146 | -1.149 | -2.938 |
| DM1 | 1.000 | 7.000 | .030 | .152 | -1.056 | -2.701 |
| AA5 | 1.000 | 7.000 | -.001 | -.006 | -.967 | -2.473 |
| AA4 | 1.000 | 7.000 | -.010 | -.051 | -1.025 | -2.622 |
| AA3 | 1.000 | 7.000 | -.067 | -.343 | -1.112 | -2.845 |
| AA2 | 1.000 | 7.000 | .058 | .296 | -1.136 | -2.907 |
| AA1 | 1.000 | 7.000 | .034 | .173 | -1.246 | -3.186 |
| Multivariate |  |  |  |  | .972 | .227 |

##### Observations farthest from the centroid (Mahalanobis distance) (east)

| Observation number | Mahalanobis d-squared | p1 | p2 |
| --- | --- | --- | --- |
| 7 | 35.531 | .008 | .721 |
| 105 | 35.012 | .009 | .436 |
| 1 | 32.372 | .020 | .605 |
| 26 | 29.780 | .040 | .873 |
| 96 | 29.765 | .040 | .752 |
| 52 | 29.538 | .042 | .654 |
| 62 | 28.992 | .048 | .642 |
| 73 | 27.406 | .072 | .882 |
| 82 | 26.683 | .085 | .925 |
| 155 | 25.805 | .104 | .971 |
| 137 | 25.681 | .107 | .956 |
| 145 | 25.239 | .118 | .967 |
| 78 | 25.132 | .121 | .952 |
| 16 | 25.129 | .121 | .918 |
| 128 | 25.027 | .124 | .890 |
| 93 | 24.758 | .132 | .893 |
| 91 | 24.673 | .134 | .859 |
| 38 | 24.644 | .135 | .804 |
| 66 | 24.405 | .142 | .807 |
| 115 | 24.400 | .142 | .737 |
| 118 | 24.378 | .143 | .664 |
| 80 | 24.375 | .143 | .577 |
| 56 | 24.339 | .144 | .502 |
| 88 | 24.092 | .152 | .523 |
| 45 | 23.997 | .155 | .477 |
| 54 | 23.866 | .159 | .450 |
| 36 | 23.859 | .160 | .370 |
| 24 | 23.574 | .169 | .416 |
| 113 | 23.449 | .174 | .393 |
| 126 | 23.414 | .175 | .331 |
| 2 | 23.176 | .184 | .362 |
| 44 | 22.954 | .192 | .389 |
| 29 | 22.812 | .198 | .380 |
| 83 | 22.801 | .198 | .313 |
| 103 | 22.607 | .206 | .331 |
| 37 | 22.549 | .209 | .289 |
| 11 | 22.517 | .210 | .240 |
| 25 | 22.168 | .225 | .329 |
| 109 | 21.685 | .246 | .506 |
| 12 | 21.683 | .246 | .434 |
| 104 | 21.524 | .254 | .447 |
| 121 | 21.515 | .254 | .380 |
| 27 | 21.445 | .258 | .348 |
| 47 | 21.403 | .260 | .305 |
| 102 | 21.374 | .261 | .258 |
| 34 | 21.233 | .268 | .264 |
| 42 | 21.213 | .269 | .218 |
| 77 | 21.193 | .270 | .177 |
| 147 | 20.879 | .286 | .256 |
| 86 | 20.867 | .286 | .209 |
| 129 | 20.812 | .289 | .183 |
| 40 | 20.798 | .290 | .145 |
| 90 | 20.743 | .293 | .126 |
| 17 | 20.677 | .296 | .111 |
| 43 | 20.444 | .308 | .147 |
| 22 | 20.392 | .311 | .127 |
| 60 | 20.246 | .319 | .137 |
| 21 | 19.844 | .342 | .256 |
| 28 | 19.831 | .342 | .212 |
| 68 | 19.805 | .344 | .177 |
| 72 | 19.795 | .344 | .141 |
| 136 | 19.728 | .348 | .128 |
| 49 | 19.591 | .356 | .138 |
| 61 | 19.513 | .361 | .128 |
| 150 | 19.479 | .363 | .107 |
| 9 | 19.469 | .364 | .082 |
| 157 | 19.389 | .368 | .076 |
| 138 | 19.308 | .373 | .071 |
| 39 | 19.286 | .374 | .056 |
| 69 | 18.955 | .395 | .109 |
| 97 | 18.890 | .399 | .099 |
| 8 | 18.640 | .414 | .148 |
| 92 | 18.256 | .439 | .281 |
| 153 | 18.155 | .445 | .283 |
| 14 | 17.951 | .459 | .347 |
| 142 | 17.938 | .460 | .297 |
| 120 | 17.910 | .462 | .259 |
| 71 | 17.876 | .464 | .227 |
| 152 | 17.539 | .486 | .366 |
| 144 | 17.420 | .494 | .382 |
| 112 | 17.340 | .500 | .373 |
| 67 | 17.169 | .511 | .424 |
| 107 | 17.145 | .513 | .379 |
| 143 | 17.123 | .515 | .334 |
| 122 | 17.100 | .516 | .291 |
| 65 | 16.813 | .536 | .416 |
| 75 | 16.657 | .547 | .459 |
| 74 | 16.653 | .547 | .399 |
| 15 | 16.590 | .551 | .380 |
| 5 | 16.374 | .566 | .465 |
| 133 | 16.170 | .581 | .544 |
| 148 | 16.143 | .583 | .499 |
| 151 | 15.995 | .593 | .539 |
| 154 | 15.890 | .600 | .550 |
| 100 | 15.753 | .610 | .582 |
| 19 | 15.670 | .616 | .577 |
| 156 | 15.540 | .625 | .604 |
| 35 | 15.522 | .626 | .553 |
| 20 | 15.436 | .632 | .549 |
| 13 | 15.403 | .634 | .507 |

##### Group number 2 (Group number 2)

##### Notes for Group (Group number 2)

The model is recursive.

Sample size = 230

##### Variable Summary (other)

##### Your model contains the following variables (other)

Observed, endogenous variables

AA1

AA2

AA3

AA4

AA5

DM1

DM2

DM3

DM5

SE1

SE2

SE3

SE4

SE5

AB1

AB2

AB3

AB5

Unobserved, endogenous variables

防御性动机

AI自我效能

使用行为

Unobserved, exogenous variables

AI威胁感知

e1

e2

e3

e4

e5

e6

e8

e9

e10

e11

e12

e13

e14

e15

e16

e17

e18

e20

e21

e22

e23

##### Variable counts (other)

|  |  |
| --- | --- |
| Number of variables in your model: | 43 |
| Number of observed variables: | 18 |
| Number of unobserved variables: | 25 |
| Number of exogenous variables: | 22 |
| Number of endogenous variables: | 21 |

##### Parameter Summary (other)

|  | Weights | Covariances | Variances | Means | Intercepts | Total |
| --- | --- | --- | --- | --- | --- | --- |
| Fixed | 25 | 0 | 0 | 0 | 0 | 25 |
| Labeled | 19 | 0 | 22 | 0 | 0 | 41 |
| Unlabeled | 0 | 0 | 0 | 0 | 0 | 0 |
| Total | 44 | 0 | 22 | 0 | 0 | 66 |

##### Assessment of normality (other)

| Variable | min | max | skew | c.r. | kurtosis | c.r. |
| --- | --- | --- | --- | --- | --- | --- |
| AB5 | 1.000 | 7.000 | .052 | .324 | -1.133 | -3.507 |
| AB3 | 1.000 | 7.000 | .036 | .222 | -1.088 | -3.369 |
| AB2 | 1.000 | 7.000 | .022 | .139 | -1.151 | -3.564 |
| AB1 | 1.000 | 7.000 | .093 | .573 | -1.013 | -3.137 |
| SE5 | 1.000 | 7.000 | .070 | .436 | -1.059 | -3.280 |
| SE4 | 1.000 | 7.000 | .093 | .575 | -1.095 | -3.388 |
| SE3 | 1.000 | 7.000 | .070 | .430 | -1.087 | -3.366 |
| SE2 | 1.000 | 7.000 | .094 | .585 | -.974 | -3.016 |
| SE1 | 1.000 | 7.000 | .004 | .026 | -1.063 | -3.292 |
| DM5 | 1.000 | 7.000 | .021 | .127 | -1.182 | -3.659 |
| DM3 | 1.000 | 7.000 | .132 | .820 | -1.007 | -3.118 |
| DM2 | 1.000 | 7.000 | .146 | .904 | -.994 | -3.078 |
| DM1 | 1.000 | 7.000 | .076 | .472 | -1.173 | -3.630 |
| AA5 | 1.000 | 7.000 | .097 | .599 | -1.150 | -3.561 |
| AA4 | 1.000 | 7.000 | .070 | .436 | -1.184 | -3.666 |
| AA3 | 1.000 | 7.000 | .217 | 1.342 | -1.037 | -3.209 |
| AA2 | 1.000 | 7.000 | .114 | .705 | -1.072 | -3.317 |
| AA1 | 1.000 | 7.000 | .126 | .778 | -1.014 | -3.139 |
| Multivariate |  |  |  |  | -6.038 | -1.706 |

##### Observations farthest from the centroid (Mahalanobis distance) (other)

| Observation number | Mahalanobis d-squared | p1 | p2 |
| --- | --- | --- | --- |
| 82 | 33.188 | .016 | .975 |
| 165 | 33.122 | .016 | .887 |
| 179 | 31.603 | .024 | .922 |
| 207 | 29.425 | .043 | .991 |
| 107 | 29.152 | .047 | .983 |
| 222 | 29.144 | .047 | .959 |
| 133 | 29.017 | .048 | .929 |
| 168 | 28.803 | .051 | .902 |
| 184 | 28.306 | .058 | .917 |
| 191 | 27.849 | .064 | .930 |
| 5 | 27.820 | .065 | .885 |
| 142 | 27.802 | .065 | .823 |
| 118 | 27.749 | .066 | .755 |
| 50 | 27.570 | .069 | .721 |
| 87 | 27.545 | .069 | .634 |
| 128 | 27.439 | .071 | .573 |
| 33 | 27.132 | .077 | .595 |
| 9 | 26.035 | .099 | .880 |
| 213 | 25.722 | .106 | .902 |
| 19 | 25.701 | .107 | .862 |
| 40 | 25.537 | .111 | .854 |
| 178 | 25.366 | .115 | .849 |
| 7 | 25.144 | .121 | .861 |
| 79 | 25.126 | .121 | .814 |
| 182 | 25.094 | .122 | .764 |
| 3 | 24.934 | .127 | .762 |
| 28 | 24.395 | .143 | .884 |
| 200 | 24.332 | .144 | .859 |
| 45 | 24.287 | .146 | .826 |
| 83 | 24.022 | .154 | .864 |
| 132 | 23.885 | .159 | .863 |
| 32 | 23.834 | .161 | .835 |
| 111 | 23.767 | .163 | .810 |
| 145 | 23.711 | .165 | .780 |
| 65 | 23.506 | .172 | .809 |
| 164 | 23.432 | .175 | .788 |
| 181 | 23.401 | .176 | .747 |
| 190 | 23.168 | .184 | .795 |
| 53 | 23.133 | .186 | .758 |
| 193 | 23.086 | .187 | .723 |
| 155 | 22.838 | .197 | .785 |
| 144 | 22.832 | .197 | .734 |
| 94 | 22.758 | .200 | .715 |
| 125 | 22.290 | .219 | .867 |
| 46 | 22.049 | .230 | .907 |
| 102 | 21.789 | .242 | .942 |
| 138 | 21.646 | .248 | .949 |
| 205 | 21.604 | .250 | .939 |
| 201 | 21.485 | .256 | .943 |
| 71 | 21.361 | .262 | .948 |
| 39 | 21.314 | .264 | .938 |
| 29 | 21.285 | .265 | .924 |
| 177 | 21.250 | .267 | .910 |
| 36 | 21.219 | .269 | .892 |
| 49 | 21.158 | .272 | .882 |
| 224 | 21.117 | .274 | .864 |
| 84 | 21.105 | .274 | .833 |
| 81 | 21.061 | .276 | .813 |
| 98 | 21.007 | .279 | .797 |
| 230 | 20.985 | .280 | .764 |
| 157 | 20.982 | .280 | .717 |
| 189 | 20.869 | .286 | .733 |
| 56 | 20.631 | .298 | .811 |
| 126 | 20.578 | .301 | .796 |
| 186 | 20.532 | .304 | .777 |
| 66 | 20.498 | .305 | .751 |
| 192 | 20.402 | .311 | .758 |
| 134 | 20.292 | .317 | .774 |
| 169 | 20.285 | .317 | .732 |
| 22 | 20.276 | .318 | .689 |
| 55 | 20.274 | .318 | .638 |
| 166 | 20.241 | .319 | .606 |
| 124 | 20.177 | .323 | .596 |
| 51 | 20.118 | .326 | .582 |
| 176 | 20.106 | .327 | .535 |
| 143 | 20.096 | .327 | .486 |
| 202 | 20.047 | .330 | .465 |
| 68 | 19.861 | .341 | .545 |
| 214 | 19.793 | .345 | .539 |
| 226 | 19.774 | .346 | .498 |
| 18 | 19.694 | .350 | .501 |
| 140 | 19.540 | .359 | .560 |
| 135 | 19.527 | .360 | .515 |
| 212 | 19.504 | .361 | .477 |
| 24 | 19.400 | .368 | .500 |
| 34 | 19.291 | .374 | .528 |
| 47 | 19.164 | .382 | .569 |
| 162 | 19.068 | .388 | .587 |
| 80 | 19.025 | .390 | .566 |
| 2 | 19.013 | .391 | .521 |
| 211 | 18.986 | .393 | .488 |
| 58 | 18.974 | .393 | .444 |
| 221 | 18.901 | .398 | .446 |
| 141 | 18.838 | .402 | .441 |
| 21 | 18.788 | .405 | .426 |
| 72 | 18.781 | .405 | .380 |
| 136 | 18.714 | .410 | .378 |
| 154 | 18.541 | .421 | .458 |
| 44 | 18.528 | .421 | .415 |
| 110 | 18.425 | .428 | .442 |

##### Models

##### Unconstrained (Unconstrained)

##### Notes for Model (Unconstrained)

##### Computation of degrees of freedom (Unconstrained)

|  |  |
| --- | --- |
| Number of distinct sample moments: | 342 |
| Number of distinct parameters to be estimated: | 82 |
| Degrees of freedom (342 - 82): | 260 |

##### Result (Unconstrained)

Minimum was achieved

Chi-square = 257.968

Degrees of freedom = 260

Probability level = .524

##### east (east - Unconstrained)

##### Estimates (east - Unconstrained)

##### Scalar Estimates (east - Unconstrained)

##### Maximum Likelihood Estimates

##### Regression Weights: (east - Unconstrained)

|  |  |  | Estimate | S.E. | C.R. | P | Label |
| --- | --- | --- | --- | --- | --- | --- | --- |
| AI自我效能 | <--- | AI威胁感知 | .506 | .083 | 6.068 | \*\*\* | H1a |
| 防御性动机 | <--- | AI威胁感知 | .362 | .107 | 3.398 | \*\*\* | H2a |
| 防御性动机 | <--- | AI自我效能 | .201 | .123 | 1.633 | .103 | H5a |
| 使用行为 | <--- | 防御性动机 | .335 | .101 | 3.328 | \*\*\* | H4a |
| 使用行为 | <--- | AI自我效能 | .570 | .119 | 4.799 | \*\*\* | H3a |
| AA1 | <--- | AI威胁感知 | 1.000 |  |  |  |  |
| AA2 | <--- | AI威胁感知 | .906 | .087 | 10.447 | \*\*\* | a1\_1 |
| AA3 | <--- | AI威胁感知 | .880 | .090 | 9.758 | \*\*\* | a2\_1 |
| AA4 | <--- | AI威胁感知 | .917 | .086 | 10.608 | \*\*\* | a3\_1 |
| AA5 | <--- | AI威胁感知 | .912 | .083 | 10.943 | \*\*\* | a4\_1 |
| DM1 | <--- | 防御性动机 | 1.000 |  |  |  |  |
| DM2 | <--- | 防御性动机 | 1.072 | .122 | 8.789 | \*\*\* | a5\_1 |
| DM3 | <--- | 防御性动机 | .981 | .121 | 8.122 | \*\*\* | a6\_1 |
| DM5 | <--- | 防御性动机 | .916 | .112 | 8.187 | \*\*\* | a7\_1 |
| SE1 | <--- | AI自我效能 | 1.000 |  |  |  |  |
| SE2 | <--- | AI自我效能 | 1.160 | .143 | 8.094 | \*\*\* | a8\_1 |
| SE3 | <--- | AI自我效能 | 1.116 | .138 | 8.083 | \*\*\* | a9\_1 |
| SE4 | <--- | AI自我效能 | 1.240 | .141 | 8.779 | \*\*\* | a10\_1 |
| SE5 | <--- | AI自我效能 | 1.230 | .141 | 8.700 | \*\*\* | a11\_1 |
| AB1 | <--- | 使用行为 | 1.000 |  |  |  |  |
| AB2 | <--- | 使用行为 | .853 | .098 | 8.710 | \*\*\* | a12\_1 |
| AB3 | <--- | 使用行为 | 1.009 | .098 | 10.260 | \*\*\* | a13\_1 |
| AB5 | <--- | 使用行为 | .963 | .097 | 9.906 | \*\*\* | a14\_1 |

##### Standardized Regression Weights: (east - Unconstrained)

|  |  |  | Estimate |
| --- | --- | --- | --- |
| AI自我效能 | <--- | AI威胁感知 | .603 |
| 防御性动机 | <--- | AI威胁感知 | .395 |
| 防御性动机 | <--- | AI自我效能 | .184 |
| 使用行为 | <--- | 防御性动机 | .307 |
| 使用行为 | <--- | AI自我效能 | .479 |
| AA1 | <--- | AI威胁感知 | .831 |
| AA2 | <--- | AI威胁感知 | .758 |
| AA3 | <--- | AI威胁感知 | .720 |
| AA4 | <--- | AI威胁感知 | .767 |
| AA5 | <--- | AI威胁感知 | .785 |
| DM1 | <--- | 防御性动机 | .725 |
| DM2 | <--- | 防御性动机 | .796 |
| DM3 | <--- | 防御性动机 | .722 |
| DM5 | <--- | 防御性动机 | .728 |
| SE1 | <--- | AI自我效能 | .671 |
| SE2 | <--- | AI自我效能 | .741 |
| SE3 | <--- | AI自我效能 | .740 |
| SE4 | <--- | AI自我效能 | .819 |
| SE5 | <--- | AI自我效能 | .809 |
| AB1 | <--- | 使用行为 | .784 |
| AB2 | <--- | 使用行为 | .699 |
| AB3 | <--- | 使用行为 | .814 |
| AB5 | <--- | 使用行为 | .786 |

##### Variances: (east - Unconstrained)

|  |  |  | Estimate | S.E. | C.R. | P | Label |
| --- | --- | --- | --- | --- | --- | --- | --- |
| AI威胁感知 |  |  | 2.188 | .357 | 6.129 | \*\*\* | vvv1\_1 |
| e21 |  |  | .982 | .227 | 4.323 | \*\*\* | vv1\_1 |
| e23 |  |  | 1.328 | .284 | 4.675 | \*\*\* | vv3\_1 |
| e22 |  |  | 1.206 | .241 | 5.002 | \*\*\* | vv2\_1 |
| e1 |  |  | .982 | .153 | 6.420 | \*\*\* | v1\_1 |
| e2 |  |  | 1.325 | .180 | 7.375 | \*\*\* | v2\_1 |
| e3 |  |  | 1.575 | .205 | 7.681 | \*\*\* | v3\_1 |
| e4 |  |  | 1.285 | .176 | 7.290 | \*\*\* | v4\_1 |
| e5 |  |  | 1.129 | .159 | 7.092 | \*\*\* | v5\_1 |
| e6 |  |  | 1.662 | .237 | 7.008 | \*\*\* | v6\_1 |
| e8 |  |  | 1.224 | .205 | 5.972 | \*\*\* | v7\_1 |
| e9 |  |  | 1.624 | .231 | 7.037 | \*\*\* | v8\_1 |
| e10 |  |  | 1.363 | .196 | 6.966 | \*\*\* | v9\_1 |
| e11 |  |  | 1.888 | .238 | 7.926 | \*\*\* | v10\_1 |
| e12 |  |  | 1.706 | .228 | 7.473 | \*\*\* | v11\_1 |
| e13 |  |  | 1.592 | .213 | 7.483 | \*\*\* | v12\_1 |
| e14 |  |  | 1.165 | .178 | 6.536 | \*\*\* | v13\_1 |
| e15 |  |  | 1.228 | .184 | 6.691 | \*\*\* | v14\_1 |
| e16 |  |  | 1.366 | .205 | 6.680 | \*\*\* | v15\_1 |
| e17 |  |  | 1.665 | .220 | 7.569 | \*\*\* | v16\_1 |
| e18 |  |  | 1.135 | .183 | 6.188 | \*\*\* | v17\_1 |
| e20 |  |  | 1.256 | .189 | 6.661 | \*\*\* | v18\_1 |

##### Squared Multiple Correlations: (east - Unconstrained)

|  |  |  | Estimate |
| --- | --- | --- | --- |
| AI自我效能 |  |  | .363 |
| 防御性动机 |  |  | .278 |
| 使用行为 |  |  | .448 |
| AB5 |  |  | .617 |
| AB3 |  |  | .662 |
| AB2 |  |  | .488 |
| AB1 |  |  | .615 |
| SE5 |  |  | .655 |
| SE4 |  |  | .671 |
| SE3 |  |  | .547 |
| SE2 |  |  | .549 |
| SE1 |  |  | .450 |
| DM5 |  |  | .531 |
| DM3 |  |  | .521 |
| DM2 |  |  | .633 |
| DM1 |  |  | .525 |
| AA5 |  |  | .617 |
| AA4 |  |  | .589 |
| AA3 |  |  | .518 |
| AA2 |  |  | .575 |
| AA1 |  |  | .690 |

##### User-defined estimands: (east - Unconstrained)

|  |  |  |  |
| --- | --- | --- | --- |
| diff1 |  |  | .214 |
| diff2 |  |  | .070 |
| diff3 |  |  | .278 |
| diff4 |  |  | .043 |
| diff5 |  |  | -.091 |

##### Modification Indices (east - Unconstrained)

##### Covariances: (east - Unconstrained)

|  |  |  | M.I. | Par Change |
| --- | --- | --- | --- | --- |
| e13 | <--> | e16 | 7.621 | -.392 |
| e4 | <--> | e17 | 4.773 | .296 |
| e4 | <--> | e11 | 4.978 | .317 |
| e2 | <--> | e8 | 4.218 | -.264 |
| e1 | <--> | e15 | 5.044 | .254 |

##### Variances: (east - Unconstrained)

|  |  |  | M.I. | Par Change |
| --- | --- | --- | --- | --- |

##### Regression Weights: (east - Unconstrained)

|  |  |  | M.I. | Par Change |
| --- | --- | --- | --- | --- |
| AB1 | <--- | SE3 | 4.767 | -.122 |
| SE2 | <--- | DM5 | 5.346 | -.152 |
| DM5 | <--- | SE2 | 4.092 | -.107 |

##### Bootstrap (east - Unconstrained)

##### Bootstrap standard errors (east - Unconstrained)

##### Scalar Estimates (east - Unconstrained)

##### Regression Weights: (east - Unconstrained)

| Parameter | | | SE | SE-SE | Mean | Bias | SE-Bias |
| --- | --- | --- | --- | --- | --- | --- | --- |
| AI自我效能 | <--- | AI威胁感知 | .079 | .002 | .512 | .006 | .003 |
| 防御性动机 | <--- | AI威胁感知 | .115 | .003 | .364 | .002 | .004 |
| 防御性动机 | <--- | AI自我效能 | .129 | .003 | .208 | .007 | .004 |
| 使用行为 | <--- | 防御性动机 | .098 | .002 | .333 | -.002 | .003 |
| 使用行为 | <--- | AI自我效能 | .121 | .003 | .570 | .001 | .004 |
| AA1 | <--- | AI威胁感知 | .000 | .000 | 1.000 | .000 | .000 |
| AA2 | <--- | AI威胁感知 | .072 | .002 | .908 | .003 | .002 |
| AA3 | <--- | AI威胁感知 | .069 | .002 | .884 | .005 | .002 |
| AA4 | <--- | AI威胁感知 | .072 | .002 | .919 | .002 | .002 |
| AA5 | <--- | AI威胁感知 | .079 | .002 | .919 | .007 | .002 |
| DM1 | <--- | 防御性动机 | .000 | .000 | 1.000 | .000 | .000 |
| DM2 | <--- | 防御性动机 | .124 | .003 | 1.085 | .013 | .004 |
| DM3 | <--- | 防御性动机 | .119 | .003 | .992 | .012 | .004 |
| DM5 | <--- | 防御性动机 | .111 | .002 | .922 | .006 | .004 |
| SE1 | <--- | AI自我效能 | .000 | .000 | 1.000 | .000 | .000 |
| SE2 | <--- | AI自我效能 | .132 | .003 | 1.160 | .000 | .004 |
| SE3 | <--- | AI自我效能 | .134 | .003 | 1.124 | .008 | .004 |
| SE4 | <--- | AI自我效能 | .140 | .003 | 1.244 | .004 | .004 |
| SE5 | <--- | AI自我效能 | .137 | .003 | 1.236 | .007 | .004 |
| AB1 | <--- | 使用行为 | .000 | .000 | 1.000 | .000 | .000 |
| AB2 | <--- | 使用行为 | .087 | .002 | .856 | .003 | .003 |
| AB3 | <--- | 使用行为 | .076 | .002 | 1.015 | .006 | .002 |
| AB5 | <--- | 使用行为 | .082 | .002 | .966 | .003 | .003 |

##### Standardized Regression Weights: (east - Unconstrained)

| Parameter | | | SE | SE-SE | Mean | Bias | SE-Bias |
| --- | --- | --- | --- | --- | --- | --- | --- |
| AI自我效能 | <--- | AI威胁感知 | .061 | .001 | .603 | .000 | .002 |
| 防御性动机 | <--- | AI威胁感知 | .114 | .003 | .394 | -.001 | .004 |
| 防御性动机 | <--- | AI自我效能 | .116 | .003 | .191 | .007 | .004 |
| 使用行为 | <--- | 防御性动机 | .089 | .002 | .305 | -.002 | .003 |
| 使用行为 | <--- | AI自我效能 | .083 | .002 | .477 | -.002 | .003 |
| AA1 | <--- | AI威胁感知 | .034 | .001 | .828 | -.002 | .001 |
| AA2 | <--- | AI威胁感知 | .038 | .001 | .757 | -.001 | .001 |
| AA3 | <--- | AI威胁感知 | .044 | .001 | .720 | .000 | .001 |
| AA4 | <--- | AI威胁感知 | .037 | .001 | .767 | -.001 | .001 |
| AA5 | <--- | AI威胁感知 | .036 | .001 | .788 | .002 | .001 |
| DM1 | <--- | 防御性动机 | .053 | .001 | .724 | -.001 | .002 |
| DM2 | <--- | 防御性动机 | .040 | .001 | .797 | .001 | .001 |
| DM3 | <--- | 防御性动机 | .048 | .001 | .724 | .001 | .002 |
| DM5 | <--- | 防御性动机 | .047 | .001 | .727 | -.001 | .001 |
| SE1 | <--- | AI自我效能 | .053 | .001 | .672 | .001 | .002 |
| SE2 | <--- | AI自我效能 | .050 | .001 | .738 | -.002 | .002 |
| SE3 | <--- | AI自我效能 | .043 | .001 | .741 | .001 | .001 |
| SE4 | <--- | AI自我效能 | .034 | .001 | .818 | -.001 | .001 |
| SE5 | <--- | AI自我效能 | .036 | .001 | .811 | .001 | .001 |
| AB1 | <--- | 使用行为 | .040 | .001 | .784 | .000 | .001 |
| AB2 | <--- | 使用行为 | .056 | .001 | .699 | .000 | .002 |
| AB3 | <--- | 使用行为 | .034 | .001 | .815 | .001 | .001 |
| AB5 | <--- | 使用行为 | .040 | .001 | .785 | .000 | .001 |

##### Variances: (east - Unconstrained)

| Parameter | | | SE | SE-SE | Mean | Bias | SE-Bias |
| --- | --- | --- | --- | --- | --- | --- | --- |
| AI威胁感知 |  |  | .260 | .006 | 2.173 | -.014 | .008 |
| e21 |  |  | .221 | .005 | .987 | .005 | .007 |
| e23 |  |  | .281 | .006 | 1.294 | -.034 | .009 |
| e22 |  |  | .215 | .005 | 1.185 | -.021 | .007 |
| e1 |  |  | .174 | .004 | .985 | .002 | .006 |
| e2 |  |  | .160 | .004 | 1.312 | -.013 | .005 |
| e3 |  |  | .196 | .004 | 1.555 | -.020 | .006 |
| e4 |  |  | .170 | .004 | 1.269 | -.016 | .005 |
| e5 |  |  | .144 | .003 | 1.104 | -.026 | .005 |
| e6 |  |  | .246 | .005 | 1.644 | -.018 | .008 |
| e8 |  |  | .200 | .004 | 1.204 | -.020 | .006 |
| e9 |  |  | .224 | .005 | 1.598 | -.026 | .007 |
| e10 |  |  | .179 | .004 | 1.348 | -.014 | .006 |
| e11 |  |  | .221 | .005 | 1.863 | -.025 | .007 |
| e12 |  |  | .281 | .006 | 1.696 | -.010 | .009 |
| e13 |  |  | .192 | .004 | 1.567 | -.026 | .006 |
| e14 |  |  | .181 | .004 | 1.153 | -.012 | .006 |
| e15 |  |  | .198 | .004 | 1.204 | -.024 | .006 |
| e16 |  |  | .203 | .005 | 1.347 | -.019 | .006 |
| e17 |  |  | .242 | .005 | 1.636 | -.029 | .008 |
| e18 |  |  | .169 | .004 | 1.116 | -.019 | .005 |
| e20 |  |  | .189 | .004 | 1.239 | -.018 | .006 |

##### Squared Multiple Correlations: (east - Unconstrained)

| Parameter | | | SE | SE-SE | Mean | Bias | SE-Bias |
| --- | --- | --- | --- | --- | --- | --- | --- |
| AI自我效能 |  |  | .073 | .002 | .367 | .004 | .002 |
| 防御性动机 |  |  | .079 | .002 | .298 | .020 | .003 |
| 使用行为 |  |  | .078 | .002 | .454 | .006 | .002 |
| AB5 |  |  | .062 | .001 | .618 | .001 | .002 |
| AB3 |  |  | .054 | .001 | .665 | .003 | .002 |
| AB2 |  |  | .077 | .002 | .492 | .004 | .002 |
| AB1 |  |  | .062 | .001 | .616 | .001 | .002 |
| SE5 |  |  | .058 | .001 | .658 | .003 | .002 |
| SE4 |  |  | .055 | .001 | .670 | .000 | .002 |
| SE3 |  |  | .064 | .001 | .550 | .003 | .002 |
| SE2 |  |  | .073 | .002 | .548 | -.001 | .002 |
| SE1 |  |  | .070 | .002 | .454 | .004 | .002 |
| DM5 |  |  | .067 | .002 | .531 | .000 | .002 |
| DM3 |  |  | .069 | .002 | .526 | .004 | .002 |
| DM2 |  |  | .064 | .001 | .637 | .004 | .002 |
| DM1 |  |  | .075 | .002 | .526 | .001 | .002 |
| AA5 |  |  | .057 | .001 | .621 | .005 | .002 |
| AA4 |  |  | .057 | .001 | .589 | .000 | .002 |
| AA3 |  |  | .062 | .001 | .521 | .003 | .002 |
| AA2 |  |  | .057 | .001 | .575 | .000 | .002 |
| AA1 |  |  | .056 | .001 | .687 | -.003 | .002 |

##### User-defined estimands: (east - Unconstrained)

| Parameter | | | SE | SE-SE | Mean | Bias | SE-Bias |
| --- | --- | --- | --- | --- | --- | --- | --- |
| diff1 |  |  | .105 | .002 | .217 | .003 | .003 |
| diff2 |  |  | .132 | .003 | .069 | -.001 | .004 |
| diff3 |  |  | .135 | .003 | .276 | -.002 | .004 |
| diff4 |  |  | .117 | .003 | .038 | -.005 | .004 |
| diff5 |  |  | .144 | .003 | -.087 | .004 | .005 |

##### Bootstrap Confidence (east - Unconstrained)

##### Percentile method (east - Unconstrained)

##### 95% confidence intervals (percentile method)

##### Scalar Estimates (east - Unconstrained)

##### Regression Weights: (east - Unconstrained)

| Parameter | | | Estimate | Lower | Upper | P |
| --- | --- | --- | --- | --- | --- | --- |
| AI自我效能 | <--- | AI威胁感知 | .506 | .358 | .671 | .002 |
| 防御性动机 | <--- | AI威胁感知 | .362 | .139 | .597 | .006 |
| 防御性动机 | <--- | AI自我效能 | .201 | -.035 | .475 | .097 |
| 使用行为 | <--- | 防御性动机 | .335 | .143 | .525 | .004 |
| 使用行为 | <--- | AI自我效能 | .570 | .323 | .821 | .002 |
| AA1 | <--- | AI威胁感知 | 1.000 | 1.000 | 1.000 | ... |
| AA2 | <--- | AI威胁感知 | .906 | .773 | 1.054 | .002 |
| AA3 | <--- | AI威胁感知 | .880 | .754 | 1.031 | .002 |
| AA4 | <--- | AI威胁感知 | .917 | .782 | 1.069 | .002 |
| AA5 | <--- | AI威胁感知 | .912 | .770 | 1.079 | .002 |
| DM1 | <--- | 防御性动机 | 1.000 | 1.000 | 1.000 | ... |
| DM2 | <--- | 防御性动机 | 1.072 | .876 | 1.354 | .002 |
| DM3 | <--- | 防御性动机 | .981 | .789 | 1.258 | .002 |
| DM5 | <--- | 防御性动机 | .916 | .720 | 1.156 | .002 |
| SE1 | <--- | AI自我效能 | 1.000 | 1.000 | 1.000 | ... |
| SE2 | <--- | AI自我效能 | 1.160 | .924 | 1.457 | .002 |
| SE3 | <--- | AI自我效能 | 1.116 | .894 | 1.412 | .002 |
| SE4 | <--- | AI自我效能 | 1.240 | .996 | 1.554 | .002 |
| SE5 | <--- | AI自我效能 | 1.230 | .990 | 1.541 | .002 |
| AB1 | <--- | 使用行为 | 1.000 | 1.000 | 1.000 | ... |
| AB2 | <--- | 使用行为 | .853 | .683 | 1.032 | .002 |
| AB3 | <--- | 使用行为 | 1.009 | .885 | 1.184 | .002 |
| AB5 | <--- | 使用行为 | .963 | .808 | 1.131 | .002 |

##### Standardized Regression Weights: (east - Unconstrained)

| Parameter | | | Estimate | Lower | Upper | P |
| --- | --- | --- | --- | --- | --- | --- |
| AI自我效能 | <--- | AI威胁感知 | .603 | .474 | .713 | .002 |
| 防御性动机 | <--- | AI威胁感知 | .395 | .160 | .600 | .006 |
| 防御性动机 | <--- | AI自我效能 | .184 | -.025 | .423 | .097 |
| 使用行为 | <--- | 防御性动机 | .307 | .125 | .466 | .004 |
| 使用行为 | <--- | AI自我效能 | .479 | .288 | .633 | .002 |
| AA1 | <--- | AI威胁感知 | .831 | .756 | .887 | .002 |
| AA2 | <--- | AI威胁感知 | .758 | .679 | .828 | .002 |
| AA3 | <--- | AI威胁感知 | .720 | .630 | .797 | .002 |
| AA4 | <--- | AI威胁感知 | .767 | .691 | .837 | .002 |
| AA5 | <--- | AI威胁感知 | .785 | .712 | .858 | .002 |
| DM1 | <--- | 防御性动机 | .725 | .613 | .815 | .002 |
| DM2 | <--- | 防御性动机 | .796 | .715 | .872 | .002 |
| DM3 | <--- | 防御性动机 | .722 | .618 | .811 | .002 |
| DM5 | <--- | 防御性动机 | .728 | .633 | .812 | .002 |
| SE1 | <--- | AI自我效能 | .671 | .565 | .769 | .002 |
| SE2 | <--- | AI自我效能 | .741 | .632 | .829 | .002 |
| SE3 | <--- | AI自我效能 | .740 | .654 | .821 | .002 |
| SE4 | <--- | AI自我效能 | .819 | .741 | .878 | .002 |
| SE5 | <--- | AI自我效能 | .809 | .735 | .872 | .002 |
| AB1 | <--- | 使用行为 | .784 | .701 | .853 | .002 |
| AB2 | <--- | 使用行为 | .699 | .574 | .804 | .002 |
| AB3 | <--- | 使用行为 | .814 | .747 | .873 | .002 |
| AB5 | <--- | 使用行为 | .786 | .704 | .857 | .002 |

##### Variances: (east - Unconstrained)

| Parameter | | | Estimate | Lower | Upper | P |
| --- | --- | --- | --- | --- | --- | --- |
| AI威胁感知 |  |  | 2.188 | 1.667 | 2.702 | .002 |
| e21 |  |  | .982 | .593 | 1.461 | .002 |
| e23 |  |  | 1.328 | .778 | 1.882 | .002 |
| e22 |  |  | 1.206 | .780 | 1.625 | .002 |
| e1 |  |  | .982 | .666 | 1.345 | .002 |
| e2 |  |  | 1.325 | 1.010 | 1.638 | .002 |
| e3 |  |  | 1.575 | 1.194 | 1.968 | .002 |
| e4 |  |  | 1.285 | .947 | 1.605 | .002 |
| e5 |  |  | 1.129 | .821 | 1.366 | .002 |
| e6 |  |  | 1.662 | 1.185 | 2.127 | .002 |
| e8 |  |  | 1.224 | .809 | 1.588 | .002 |
| e9 |  |  | 1.624 | 1.160 | 2.066 | .002 |
| e10 |  |  | 1.363 | 1.004 | 1.726 | .002 |
| e11 |  |  | 1.888 | 1.439 | 2.326 | .002 |
| e12 |  |  | 1.706 | 1.156 | 2.267 | .002 |
| e13 |  |  | 1.592 | 1.193 | 1.946 | .002 |
| e14 |  |  | 1.165 | .827 | 1.535 | .002 |
| e15 |  |  | 1.228 | .849 | 1.598 | .002 |
| e16 |  |  | 1.366 | .973 | 1.770 | .002 |
| e17 |  |  | 1.665 | 1.153 | 2.143 | .002 |
| e18 |  |  | 1.135 | .792 | 1.444 | .002 |
| e20 |  |  | 1.256 | .877 | 1.606 | .002 |

##### Squared Multiple Correlations: (east - Unconstrained)

| Parameter | | | Estimate | Lower | Upper | P |
| --- | --- | --- | --- | --- | --- | --- |
| AI自我效能 |  |  | .363 | .225 | .508 | .002 |
| 防御性动机 |  |  | .278 | .155 | .457 | .002 |
| 使用行为 |  |  | .448 | .301 | .611 | .002 |
| AB5 |  |  | .617 | .495 | .735 | .002 |
| AB3 |  |  | .662 | .558 | .763 | .002 |
| AB2 |  |  | .488 | .330 | .646 | .002 |
| AB1 |  |  | .615 | .491 | .728 | .002 |
| SE5 |  |  | .655 | .541 | .761 | .002 |
| SE4 |  |  | .671 | .550 | .771 | .002 |
| SE3 |  |  | .547 | .427 | .673 | .002 |
| SE2 |  |  | .549 | .399 | .687 | .002 |
| SE1 |  |  | .450 | .319 | .592 | .002 |
| DM5 |  |  | .531 | .400 | .659 | .002 |
| DM3 |  |  | .521 | .382 | .658 | .002 |
| DM2 |  |  | .633 | .512 | .761 | .002 |
| DM1 |  |  | .525 | .376 | .664 | .002 |
| AA5 |  |  | .617 | .507 | .736 | .002 |
| AA4 |  |  | .589 | .478 | .700 | .002 |
| AA3 |  |  | .518 | .396 | .636 | .002 |
| AA2 |  |  | .575 | .461 | .685 | .002 |
| AA1 |  |  | .690 | .571 | .787 | .002 |

##### User-defined estimands: (east - Unconstrained)

| Parameter | | | Estimate | Lower | Upper | P |
| --- | --- | --- | --- | --- | --- | --- |
| diff1 |  |  | .214 | .005 | .409 | .038 |
| diff2 |  |  | .070 | -.204 | .329 | .586 |
| diff3 |  |  | .278 | .009 | .571 | .043 |
| diff4 |  |  | .043 | -.174 | .274 | .751 |
| diff5 |  |  | -.091 | -.356 | .212 | .541 |

##### Bias-corrected percentile method (east - Unconstrained)

##### 95% confidence intervals (bias-corrected percentile method)

##### Scalar Estimates (east - Unconstrained)

##### Regression Weights: (east - Unconstrained)

| Parameter | | | Estimate | Lower | Upper | P |
| --- | --- | --- | --- | --- | --- | --- |
| AI自我效能 | <--- | AI威胁感知 | .506 | .354 | .665 | .003 |
| 防御性动机 | <--- | AI威胁感知 | .362 | .143 | .599 | .006 |
| 防御性动机 | <--- | AI自我效能 | .201 | -.035 | .475 | .097 |
| 使用行为 | <--- | 防御性动机 | .335 | .153 | .538 | .003 |
| 使用行为 | <--- | AI自我效能 | .570 | .337 | .832 | .002 |
| AA1 | <--- | AI威胁感知 | 1.000 | 1.000 | 1.000 | ... |
| AA2 | <--- | AI威胁感知 | .906 | .776 | 1.057 | .002 |
| AA3 | <--- | AI威胁感知 | .880 | .743 | 1.019 | .003 |
| AA4 | <--- | AI威胁感知 | .917 | .778 | 1.062 | .003 |
| AA5 | <--- | AI威胁感知 | .912 | .765 | 1.068 | .003 |
| DM1 | <--- | 防御性动机 | 1.000 | 1.000 | 1.000 | ... |
| DM2 | <--- | 防御性动机 | 1.072 | .875 | 1.349 | .002 |
| DM3 | <--- | 防御性动机 | .981 | .774 | 1.227 | .003 |
| DM5 | <--- | 防御性动机 | .916 | .715 | 1.140 | .002 |
| SE1 | <--- | AI自我效能 | 1.000 | 1.000 | 1.000 | ... |
| SE2 | <--- | AI自我效能 | 1.160 | .948 | 1.504 | .001 |
| SE3 | <--- | AI自我效能 | 1.116 | .900 | 1.420 | .002 |
| SE4 | <--- | AI自我效能 | 1.240 | 1.006 | 1.568 | .001 |
| SE5 | <--- | AI自我效能 | 1.230 | .998 | 1.561 | .002 |
| AB1 | <--- | 使用行为 | 1.000 | 1.000 | 1.000 | ... |
| AB2 | <--- | 使用行为 | .853 | .680 | 1.022 | .003 |
| AB3 | <--- | 使用行为 | 1.009 | .885 | 1.183 | .002 |
| AB5 | <--- | 使用行为 | .963 | .809 | 1.133 | .002 |

##### Standardized Regression Weights: (east - Unconstrained)

| Parameter | | | Estimate | Lower | Upper | P |
| --- | --- | --- | --- | --- | --- | --- |
| AI自我效能 | <--- | AI威胁感知 | .603 | .469 | .709 | .003 |
| 防御性动机 | <--- | AI威胁感知 | .395 | .148 | .589 | .008 |
| 防御性动机 | <--- | AI自我效能 | .184 | -.035 | .419 | .106 |
| 使用行为 | <--- | 防御性动机 | .307 | .134 | .476 | .003 |
| 使用行为 | <--- | AI自我效能 | .479 | .286 | .628 | .002 |
| AA1 | <--- | AI威胁感知 | .831 | .757 | .888 | .002 |
| AA2 | <--- | AI威胁感知 | .758 | .674 | .825 | .003 |
| AA3 | <--- | AI威胁感知 | .720 | .628 | .796 | .003 |
| AA4 | <--- | AI威胁感知 | .767 | .691 | .837 | .002 |
| AA5 | <--- | AI威胁感知 | .785 | .707 | .852 | .003 |
| DM1 | <--- | 防御性动机 | .725 | .607 | .811 | .003 |
| DM2 | <--- | 防御性动机 | .796 | .713 | .869 | .003 |
| DM3 | <--- | 防御性动机 | .722 | .612 | .803 | .004 |
| DM5 | <--- | 防御性动机 | .728 | .632 | .811 | .002 |
| SE1 | <--- | AI自我效能 | .671 | .554 | .763 | .003 |
| SE2 | <--- | AI自我效能 | .741 | .634 | .835 | .002 |
| SE3 | <--- | AI自我效能 | .740 | .647 | .818 | .002 |
| SE4 | <--- | AI自我效能 | .819 | .737 | .875 | .003 |
| SE5 | <--- | AI自我效能 | .809 | .729 | .869 | .003 |
| AB1 | <--- | 使用行为 | .784 | .694 | .849 | .003 |
| AB2 | <--- | 使用行为 | .699 | .571 | .801 | .003 |
| AB3 | <--- | 使用行为 | .814 | .740 | .872 | .003 |
| AB5 | <--- | 使用行为 | .786 | .699 | .855 | .003 |

##### Variances: (east - Unconstrained)

| Parameter | | | Estimate | Lower | Upper | P |
| --- | --- | --- | --- | --- | --- | --- |
| AI威胁感知 |  |  | 2.188 | 1.695 | 2.722 | .001 |
| e21 |  |  | .982 | .624 | 1.523 | .001 |
| e23 |  |  | 1.328 | .855 | 2.021 | .001 |
| e22 |  |  | 1.206 | .831 | 1.669 | .001 |
| e1 |  |  | .982 | .675 | 1.347 | .002 |
| e2 |  |  | 1.325 | 1.033 | 1.661 | .001 |
| e3 |  |  | 1.575 | 1.242 | 2.027 | .001 |
| e4 |  |  | 1.285 | .981 | 1.642 | .001 |
| e5 |  |  | 1.129 | .875 | 1.433 | .001 |
| e6 |  |  | 1.662 | 1.223 | 2.162 | .001 |
| e8 |  |  | 1.224 | .844 | 1.611 | .001 |
| e9 |  |  | 1.624 | 1.254 | 2.167 | .001 |
| e10 |  |  | 1.363 | 1.036 | 1.767 | .001 |
| e11 |  |  | 1.888 | 1.483 | 2.373 | .001 |
| e12 |  |  | 1.706 | 1.199 | 2.309 | .001 |
| e13 |  |  | 1.592 | 1.252 | 2.000 | .001 |
| e14 |  |  | 1.165 | .846 | 1.554 | .001 |
| e15 |  |  | 1.228 | .885 | 1.659 | .001 |
| e16 |  |  | 1.366 | 1.000 | 1.826 | .001 |
| e17 |  |  | 1.665 | 1.231 | 2.196 | .001 |
| e18 |  |  | 1.135 | .841 | 1.496 | .001 |
| e20 |  |  | 1.256 | .914 | 1.632 | .001 |

##### Squared Multiple Correlations: (east - Unconstrained)

| Parameter | | | Estimate | Lower | Upper | P |
| --- | --- | --- | --- | --- | --- | --- |
| AI自我效能 |  |  | .363 | .220 | .503 | .003 |
| 防御性动机 |  |  | .278 | .113 | .424 | .009 |
| 使用行为 |  |  | .448 | .290 | .593 | .003 |
| AB5 |  |  | .617 | .489 | .731 | .003 |
| AB3 |  |  | .662 | .548 | .760 | .003 |
| AB2 |  |  | .488 | .326 | .641 | .003 |
| AB1 |  |  | .615 | .481 | .721 | .003 |
| SE5 |  |  | .655 | .531 | .756 | .003 |
| SE4 |  |  | .671 | .543 | .765 | .003 |
| SE3 |  |  | .547 | .418 | .670 | .002 |
| SE2 |  |  | .549 | .402 | .696 | .002 |
| SE1 |  |  | .450 | .307 | .582 | .003 |
| DM5 |  |  | .531 | .399 | .658 | .002 |
| DM3 |  |  | .521 | .374 | .645 | .004 |
| DM2 |  |  | .633 | .508 | .755 | .003 |
| DM1 |  |  | .525 | .368 | .658 | .003 |
| AA5 |  |  | .617 | .500 | .726 | .003 |
| AA4 |  |  | .589 | .478 | .700 | .002 |
| AA3 |  |  | .518 | .394 | .634 | .003 |
| AA2 |  |  | .575 | .455 | .681 | .003 |
| AA1 |  |  | .690 | .573 | .788 | .002 |

##### User-defined estimands: (east - Unconstrained)

| Parameter | | | Estimate | Lower | Upper | P |
| --- | --- | --- | --- | --- | --- | --- |
| diff1 |  |  | .214 | .001 | .402 | .046 |
| diff2 |  |  | .070 | -.201 | .330 | .576 |
| diff3 |  |  | .278 | .036 | .599 | .030 |
| diff4 |  |  | .043 | -.168 | .287 | .651 |
| diff5 |  |  | -.091 | -.356 | .212 | .541 |

##### other (other - Unconstrained)

##### Estimates (other - Unconstrained)

##### Scalar Estimates (other - Unconstrained)

##### Maximum Likelihood Estimates

##### Regression Weights: (other - Unconstrained)

|  |  |  | Estimate | S.E. | C.R. | P | Label |
| --- | --- | --- | --- | --- | --- | --- | --- |
| AI自我效能 | <--- | AI威胁感知 | .292 | .060 | 4.845 | \*\*\* | H1 |
| 防御性动机 | <--- | AI威胁感知 | .405 | .076 | 5.334 | \*\*\* | H2 |
| 防御性动机 | <--- | AI自我效能 | -.016 | .094 | -.167 | .868 | H5 |
| 使用行为 | <--- | 防御性动机 | .433 | .084 | 5.122 | \*\*\* | H4 |
| 使用行为 | <--- | AI自我效能 | .387 | .094 | 4.121 | \*\*\* | H3 |
| AA1 | <--- | AI威胁感知 | 1.000 |  |  |  |  |
| AA2 | <--- | AI威胁感知 | .985 | .075 | 13.141 | \*\*\* | a1\_2 |
| AA3 | <--- | AI威胁感知 | .948 | .076 | 12.551 | \*\*\* | a2\_2 |
| AA4 | <--- | AI威胁感知 | .921 | .077 | 11.957 | \*\*\* | a3\_2 |
| AA5 | <--- | AI威胁感知 | .959 | .076 | 12.636 | \*\*\* | a4\_2 |
| DM1 | <--- | 防御性动机 | 1.000 |  |  |  |  |
| DM2 | <--- | 防御性动机 | .985 | .110 | 8.970 | \*\*\* | a5\_2 |
| DM3 | <--- | 防御性动机 | 1.102 | .113 | 9.789 | \*\*\* | a6\_2 |
| DM5 | <--- | 防御性动机 | 1.038 | .116 | 8.951 | \*\*\* | a7\_2 |
| SE1 | <--- | AI自我效能 | 1.000 |  |  |  |  |
| SE2 | <--- | AI自我效能 | 1.149 | .140 | 8.232 | \*\*\* | a8\_2 |
| SE3 | <--- | AI自我效能 | 1.080 | .134 | 8.050 | \*\*\* | a9\_2 |
| SE4 | <--- | AI自我效能 | 1.304 | .145 | 8.997 | \*\*\* | a10\_2 |
| SE5 | <--- | AI自我效能 | 1.212 | .136 | 8.905 | \*\*\* | a11\_2 |
| AB1 | <--- | 使用行为 | 1.000 |  |  |  |  |
| AB2 | <--- | 使用行为 | 1.084 | .103 | 10.555 | \*\*\* | a12\_2 |
| AB3 | <--- | 使用行为 | 1.122 | .102 | 10.970 | \*\*\* | a13\_2 |
| AB5 | <--- | 使用行为 | .967 | .102 | 9.481 | \*\*\* | a14\_2 |

##### Standardized Regression Weights: (other - Unconstrained)

|  |  |  | Estimate |
| --- | --- | --- | --- |
| AI自我效能 | <--- | AI威胁感知 | .397 |
| 防御性动机 | <--- | AI威胁感知 | .475 |
| 防御性动机 | <--- | AI自我效能 | -.014 |
| 使用行为 | <--- | 防御性动机 | .419 |
| 使用行为 | <--- | AI自我效能 | .324 |
| AA1 | <--- | AI威胁感知 | .810 |
| AA2 | <--- | AI威胁感知 | .800 |
| AA3 | <--- | AI威胁感知 | .771 |
| AA4 | <--- | AI威胁感知 | .742 |
| AA5 | <--- | AI威胁感知 | .775 |
| DM1 | <--- | 防御性动机 | .693 |
| DM2 | <--- | 防御性动机 | .697 |
| DM3 | <--- | 防御性动机 | .790 |
| DM5 | <--- | 防御性动机 | .695 |
| SE1 | <--- | AI自我效能 | .615 |
| SE2 | <--- | AI自我效能 | .690 |
| SE3 | <--- | AI自我效能 | .669 |
| SE4 | <--- | AI自我效能 | .791 |
| SE5 | <--- | AI自我效能 | .777 |
| AB1 | <--- | 使用行为 | .734 |
| AB2 | <--- | 使用行为 | .767 |
| AB3 | <--- | 使用行为 | .807 |
| AB5 | <--- | 使用行为 | .682 |

##### Variances: (other - Unconstrained)

|  |  |  | Estimate | S.E. | C.R. | P | Label |
| --- | --- | --- | --- | --- | --- | --- | --- |
| AI威胁感知 |  |  | 2.183 | .306 | 7.138 | \*\*\* | vvv1\_2 |
| e21 |  |  | .994 | .210 | 4.736 | \*\*\* | vv1\_2 |
| e23 |  |  | 1.232 | .232 | 5.311 | \*\*\* | vv3\_2 |
| e22 |  |  | 1.135 | .202 | 5.630 | \*\*\* | vv2\_2 |
| e1 |  |  | 1.144 | .140 | 8.157 | \*\*\* | v1\_2 |
| e2 |  |  | 1.193 | .143 | 8.337 | \*\*\* | v2\_2 |
| e3 |  |  | 1.341 | .153 | 8.756 | \*\*\* | v3\_2 |
| e4 |  |  | 1.518 | .167 | 9.083 | \*\*\* | v4\_2 |
| e5 |  |  | 1.334 | .153 | 8.702 | \*\*\* | v5\_2 |
| e6 |  |  | 1.715 | .199 | 8.613 | \*\*\* | v6\_2 |
| e8 |  |  | 1.622 | .190 | 8.556 | \*\*\* | v7\_2 |
| e9 |  |  | 1.154 | .167 | 6.933 | \*\*\* | v8\_2 |
| e10 |  |  | 1.819 | .212 | 8.579 | \*\*\* | v9\_2 |
| e11 |  |  | 1.937 | .203 | 9.554 | \*\*\* | v10\_2 |
| e12 |  |  | 1.717 | .191 | 8.980 | \*\*\* | v11\_2 |
| e13 |  |  | 1.702 | .186 | 9.172 | \*\*\* | v12\_2 |
| e14 |  |  | 1.200 | .160 | 7.496 | \*\*\* | v13\_2 |
| e15 |  |  | 1.139 | .146 | 7.780 | \*\*\* | v14\_2 |
| e16 |  |  | 1.446 | .172 | 8.424 | \*\*\* | v15\_2 |
| e17 |  |  | 1.388 | .176 | 7.896 | \*\*\* | v16\_2 |
| e18 |  |  | 1.139 | .162 | 7.049 | \*\*\* | v17\_2 |
| e20 |  |  | 1.811 | .201 | 9.018 | \*\*\* | v18\_2 |

##### Squared Multiple Correlations: (other - Unconstrained)

|  |  |  | Estimate |
| --- | --- | --- | --- |
| AI自我效能 |  |  | .158 |
| 防御性动机 |  |  | .221 |
| 使用行为 |  |  | .328 |
| AB5 |  |  | .466 |
| AB3 |  |  | .651 |
| AB2 |  |  | .588 |
| AB1 |  |  | .539 |
| SE5 |  |  | .604 |
| SE4 |  |  | .626 |
| SE3 |  |  | .447 |
| SE2 |  |  | .476 |
| SE1 |  |  | .379 |
| DM5 |  |  | .484 |
| DM3 |  |  | .624 |
| DM2 |  |  | .486 |
| DM1 |  |  | .480 |
| AA5 |  |  | .601 |
| AA4 |  |  | .550 |
| AA3 |  |  | .594 |
| AA2 |  |  | .640 |
| AA1 |  |  | .656 |

##### User-defined estimands: (other - Unconstrained)

|  |  |  |  |
| --- | --- | --- | --- |
| diff1 |  |  | .214 |
| diff2 |  |  | .070 |
| diff3 |  |  | .278 |
| diff4 |  |  | .043 |
| diff5 |  |  | -.091 |

##### Modification Indices (other - Unconstrained)

##### Covariances: (other - Unconstrained)

|  |  |  | M.I. | Par Change |
| --- | --- | --- | --- | --- |
| e20 | <--> | e23 | 4.382 | -.253 |
| e13 | <--> | AI威胁感知 | 4.103 | -.291 |
| e9 | <--> | e18 | 4.183 | .215 |
| e8 | <--> | e22 | 6.912 | .298 |
| e6 | <--> | e11 | 5.512 | -.323 |
| e5 | <--> | e14 | 12.260 | -.368 |
| e4 | <--> | e5 | 4.648 | .234 |
| e3 | <--> | e17 | 4.416 | .233 |
| e3 | <--> | e4 | 4.076 | -.220 |
| e2 | <--> | e14 | 6.158 | .251 |
| e1 | <--> | e3 | 4.890 | .216 |

##### Variances: (other - Unconstrained)

|  |  |  | M.I. | Par Change |
| --- | --- | --- | --- | --- |

##### Regression Weights: (other - Unconstrained)

|  |  |  | M.I. | Par Change |
| --- | --- | --- | --- | --- |
| AB5 | <--- | SE3 | 4.702 | .118 |
| AB2 | <--- | SE3 | 5.103 | -.113 |
| SE4 | <--- | AA2 | 4.787 | .100 |
| SE3 | <--- | AI威胁感知 | 4.103 | -.133 |
| SE3 | <--- | AA3 | 4.117 | -.103 |
| SE3 | <--- | AA2 | 6.708 | -.131 |
| SE1 | <--- | DM1 | 6.373 | -.134 |
| DM2 | <--- | 使用行为 | 4.622 | .165 |
| DM2 | <--- | AB2 | 5.531 | .118 |
| DM2 | <--- | AB1 | 6.301 | .130 |
| DM1 | <--- | SE1 | 4.911 | -.118 |
| AA5 | <--- | SE4 | 6.499 | -.119 |

##### Bootstrap (other - Unconstrained)

##### Bootstrap standard errors (other - Unconstrained)

##### Scalar Estimates (other - Unconstrained)

##### Regression Weights: (other - Unconstrained)

| Parameter | | | SE | SE-SE | Mean | Bias | SE-Bias |
| --- | --- | --- | --- | --- | --- | --- | --- |
| AI自我效能 | <--- | AI威胁感知 | .063 | .001 | .295 | .003 | .002 |
| 防御性动机 | <--- | AI威胁感知 | .075 | .002 | .406 | .002 | .002 |
| 防御性动机 | <--- | AI自我效能 | .099 | .002 | -.016 | .000 | .003 |
| 使用行为 | <--- | 防御性动机 | .083 | .002 | .439 | .006 | .003 |
| 使用行为 | <--- | AI自我效能 | .093 | .002 | .388 | .001 | .003 |
| AA1 | <--- | AI威胁感知 | .000 | .000 | 1.000 | .000 | .000 |
| AA2 | <--- | AI威胁感知 | .067 | .002 | .983 | -.001 | .002 |
| AA3 | <--- | AI威胁感知 | .061 | .001 | .949 | .001 | .002 |
| AA4 | <--- | AI威胁感知 | .076 | .002 | .921 | -.001 | .002 |
| AA5 | <--- | AI威胁感知 | .070 | .002 | .959 | .000 | .002 |
| DM1 | <--- | 防御性动机 | .000 | .000 | 1.000 | .000 | .000 |
| DM2 | <--- | 防御性动机 | .097 | .002 | .992 | .006 | .003 |
| DM3 | <--- | 防御性动机 | .098 | .002 | 1.112 | .010 | .003 |
| DM5 | <--- | 防御性动机 | .110 | .002 | 1.048 | .010 | .003 |
| SE1 | <--- | AI自我效能 | .000 | .000 | 1.000 | .000 | .000 |
| SE2 | <--- | AI自我效能 | .134 | .003 | 1.148 | -.001 | .004 |
| SE3 | <--- | AI自我效能 | .119 | .003 | 1.085 | .005 | .004 |
| SE4 | <--- | AI自我效能 | .137 | .003 | 1.308 | .004 | .004 |
| SE5 | <--- | AI自我效能 | .132 | .003 | 1.219 | .006 | .004 |
| AB1 | <--- | 使用行为 | .000 | .000 | 1.000 | .000 | .000 |
| AB2 | <--- | 使用行为 | .089 | .002 | 1.087 | .004 | .003 |
| AB3 | <--- | 使用行为 | .103 | .002 | 1.128 | .006 | .003 |
| AB5 | <--- | 使用行为 | .099 | .002 | .972 | .005 | .003 |

##### Standardized Regression Weights: (other - Unconstrained)

| Parameter | | | SE | SE-SE | Mean | Bias | SE-Bias |
| --- | --- | --- | --- | --- | --- | --- | --- |
| AI自我效能 | <--- | AI威胁感知 | .068 | .002 | .397 | .000 | .002 |
| 防御性动机 | <--- | AI威胁感知 | .074 | .002 | .479 | .004 | .002 |
| 防御性动机 | <--- | AI自我效能 | .084 | .002 | -.013 | .000 | .003 |
| 使用行为 | <--- | 防御性动机 | .067 | .001 | .420 | .001 | .002 |
| 使用行为 | <--- | AI自我效能 | .068 | .002 | .323 | -.001 | .002 |
| AA1 | <--- | AI威胁感知 | .031 | .001 | .811 | .000 | .001 |
| AA2 | <--- | AI威胁感知 | .030 | .001 | .798 | -.002 | .001 |
| AA3 | <--- | AI威胁感知 | .030 | .001 | .771 | .000 | .001 |
| AA4 | <--- | AI威胁感知 | .038 | .001 | .740 | -.001 | .001 |
| AA5 | <--- | AI威胁感知 | .036 | .001 | .775 | .000 | .001 |
| DM1 | <--- | 防御性动机 | .042 | .001 | .690 | -.003 | .001 |
| DM2 | <--- | 防御性动机 | .044 | .001 | .696 | -.001 | .001 |
| DM3 | <--- | 防御性动机 | .035 | .001 | .791 | .000 | .001 |
| DM5 | <--- | 防御性动机 | .045 | .001 | .695 | .000 | .001 |
| SE1 | <--- | AI自我效能 | .048 | .001 | .617 | .002 | .002 |
| SE2 | <--- | AI自我效能 | .045 | .001 | .688 | -.002 | .001 |
| SE3 | <--- | AI自我效能 | .043 | .001 | .670 | .002 | .001 |
| SE4 | <--- | AI自我效能 | .034 | .001 | .790 | -.001 | .001 |
| SE5 | <--- | AI自我效能 | .036 | .001 | .778 | .001 | .001 |
| AB1 | <--- | 使用行为 | .040 | .001 | .735 | .001 | .001 |
| AB2 | <--- | 使用行为 | .036 | .001 | .767 | .000 | .001 |
| AB3 | <--- | 使用行为 | .035 | .001 | .807 | .001 | .001 |
| AB5 | <--- | 使用行为 | .042 | .001 | .683 | .001 | .001 |

##### Variances: (other - Unconstrained)

| Parameter | | | SE | SE-SE | Mean | Bias | SE-Bias |
| --- | --- | --- | --- | --- | --- | --- | --- |
| AI威胁感知 |  |  | .264 | .006 | 2.191 | .008 | .008 |
| e21 |  |  | .185 | .004 | 1.000 | .005 | .006 |
| e23 |  |  | .197 | .004 | 1.199 | -.033 | .006 |
| e22 |  |  | .188 | .004 | 1.124 | -.011 | .006 |
| e1 |  |  | .141 | .003 | 1.131 | -.013 | .004 |
| e2 |  |  | .149 | .003 | 1.195 | .002 | .005 |
| e3 |  |  | .137 | .003 | 1.328 | -.014 | .004 |
| e4 |  |  | .176 | .004 | 1.508 | -.009 | .006 |
| e5 |  |  | .184 | .004 | 1.319 | -.015 | .006 |
| e6 |  |  | .189 | .004 | 1.712 | -.003 | .006 |
| e8 |  |  | .197 | .004 | 1.607 | -.016 | .006 |
| e9 |  |  | .148 | .003 | 1.136 | -.018 | .005 |
| e10 |  |  | .206 | .005 | 1.799 | -.020 | .007 |
| e11 |  |  | .176 | .004 | 1.913 | -.024 | .006 |
| e12 |  |  | .185 | .004 | 1.701 | -.016 | .006 |
| e13 |  |  | .181 | .004 | 1.677 | -.024 | .006 |
| e14 |  |  | .153 | .003 | 1.191 | -.009 | .005 |
| e15 |  |  | .153 | .003 | 1.123 | -.016 | .005 |
| e16 |  |  | .160 | .004 | 1.424 | -.022 | .005 |
| e17 |  |  | .175 | .004 | 1.378 | -.010 | .006 |
| e18 |  |  | .178 | .004 | 1.127 | -.012 | .006 |
| e20 |  |  | .183 | .004 | 1.792 | -.020 | .006 |

##### Squared Multiple Correlations: (other - Unconstrained)

| Parameter | | | SE | SE-SE | Mean | Bias | SE-Bias |
| --- | --- | --- | --- | --- | --- | --- | --- |
| AI自我效能 |  |  | .053 | .001 | .162 | .005 | .002 |
| 防御性动机 |  |  | .061 | .001 | .235 | .014 | .002 |
| 使用行为 |  |  | .063 | .001 | .336 | .008 | .002 |
| AB5 |  |  | .058 | .001 | .469 | .003 | .002 |
| AB3 |  |  | .056 | .001 | .653 | .002 | .002 |
| AB2 |  |  | .055 | .001 | .589 | .001 | .002 |
| AB1 |  |  | .059 | .001 | .542 | .003 | .002 |
| SE5 |  |  | .055 | .001 | .607 | .003 | .002 |
| SE4 |  |  | .053 | .001 | .626 | .000 | .002 |
| SE3 |  |  | .058 | .001 | .451 | .004 | .002 |
| SE2 |  |  | .062 | .001 | .475 | -.001 | .002 |
| SE1 |  |  | .058 | .001 | .383 | .005 | .002 |
| DM5 |  |  | .063 | .001 | .485 | .002 | .002 |
| DM3 |  |  | .056 | .001 | .626 | .002 | .002 |
| DM2 |  |  | .062 | .001 | .487 | .000 | .002 |
| DM1 |  |  | .058 | .001 | .477 | -.003 | .002 |
| AA5 |  |  | .056 | .001 | .602 | .002 | .002 |
| AA4 |  |  | .056 | .001 | .550 | .000 | .002 |
| AA3 |  |  | .046 | .001 | .596 | .001 | .001 |
| AA2 |  |  | .048 | .001 | .637 | -.003 | .002 |
| AA1 |  |  | .049 | .001 | .658 | .002 | .002 |

##### User-defined estimands: (other - Unconstrained)

| Parameter | | | SE | SE-SE | Mean | Bias | SE-Bias |
| --- | --- | --- | --- | --- | --- | --- | --- |
| diff1 |  |  | .105 | .002 | .217 | .003 | .003 |
| diff2 |  |  | .132 | .003 | .069 | -.001 | .004 |
| diff3 |  |  | .135 | .003 | .276 | -.002 | .004 |
| diff4 |  |  | .117 | .003 | .038 | -.005 | .004 |
| diff5 |  |  | .144 | .003 | -.087 | .004 | .005 |

##### Bootstrap Confidence (other - Unconstrained)

##### Percentile method (other - Unconstrained)

##### 95% confidence intervals (percentile method)

##### Scalar Estimates (other - Unconstrained)

##### Regression Weights: (other - Unconstrained)

| Parameter | | | Estimate | Lower | Upper | P |
| --- | --- | --- | --- | --- | --- | --- |
| AI自我效能 | <--- | AI威胁感知 | .292 | .173 | .422 | .002 |
| 防御性动机 | <--- | AI威胁感知 | .405 | .268 | .560 | .002 |
| 防御性动机 | <--- | AI自我效能 | -.016 | -.219 | .172 | .882 |
| 使用行为 | <--- | 防御性动机 | .433 | .287 | .610 | .002 |
| 使用行为 | <--- | AI自我效能 | .387 | .213 | .582 | .002 |
| AA1 | <--- | AI威胁感知 | 1.000 | 1.000 | 1.000 | ... |
| AA2 | <--- | AI威胁感知 | .985 | .858 | 1.118 | .002 |
| AA3 | <--- | AI威胁感知 | .948 | .830 | 1.083 | .002 |
| AA4 | <--- | AI威胁感知 | .921 | .794 | 1.091 | .002 |
| AA5 | <--- | AI威胁感知 | .959 | .828 | 1.113 | .002 |
| DM1 | <--- | 防御性动机 | 1.000 | 1.000 | 1.000 | ... |
| DM2 | <--- | 防御性动机 | .985 | .822 | 1.192 | .002 |
| DM3 | <--- | 防御性动机 | 1.102 | .938 | 1.307 | .002 |
| DM5 | <--- | 防御性动机 | 1.038 | .839 | 1.274 | .002 |
| SE1 | <--- | AI自我效能 | 1.000 | 1.000 | 1.000 | ... |
| SE2 | <--- | AI自我效能 | 1.149 | .908 | 1.447 | .002 |
| SE3 | <--- | AI自我效能 | 1.080 | .871 | 1.346 | .002 |
| SE4 | <--- | AI自我效能 | 1.304 | 1.088 | 1.625 | .002 |
| SE5 | <--- | AI自我效能 | 1.212 | .998 | 1.511 | .002 |
| AB1 | <--- | 使用行为 | 1.000 | 1.000 | 1.000 | ... |
| AB2 | <--- | 使用行为 | 1.084 | .922 | 1.278 | .002 |
| AB3 | <--- | 使用行为 | 1.122 | .954 | 1.367 | .002 |
| AB5 | <--- | 使用行为 | .967 | .801 | 1.177 | .002 |

##### Standardized Regression Weights: (other - Unconstrained)

| Parameter | | | Estimate | Lower | Upper | P |
| --- | --- | --- | --- | --- | --- | --- |
| AI自我效能 | <--- | AI威胁感知 | .397 | .256 | .521 | .002 |
| 防御性动机 | <--- | AI威胁感知 | .475 | .328 | .623 | .002 |
| 防御性动机 | <--- | AI自我效能 | -.014 | -.184 | .150 | .882 |
| 使用行为 | <--- | 防御性动机 | .419 | .292 | .548 | .002 |
| 使用行为 | <--- | AI自我效能 | .324 | .188 | .457 | .002 |
| AA1 | <--- | AI威胁感知 | .810 | .747 | .865 | .002 |
| AA2 | <--- | AI威胁感知 | .800 | .732 | .852 | .002 |
| AA3 | <--- | AI威胁感知 | .771 | .706 | .826 | .002 |
| AA4 | <--- | AI威胁感知 | .742 | .664 | .809 | .002 |
| AA5 | <--- | AI威胁感知 | .775 | .701 | .838 | .002 |
| DM1 | <--- | 防御性动机 | .693 | .606 | .768 | .002 |
| DM2 | <--- | 防御性动机 | .697 | .608 | .781 | .002 |
| DM3 | <--- | 防御性动机 | .790 | .715 | .858 | .002 |
| DM5 | <--- | 防御性动机 | .695 | .599 | .777 | .002 |
| SE1 | <--- | AI自我效能 | .615 | .515 | .707 | .002 |
| SE2 | <--- | AI自我效能 | .690 | .595 | .769 | .002 |
| SE3 | <--- | AI自我效能 | .669 | .578 | .751 | .002 |
| SE4 | <--- | AI自我效能 | .791 | .720 | .853 | .002 |
| SE5 | <--- | AI自我效能 | .777 | .701 | .844 | .002 |
| AB1 | <--- | 使用行为 | .734 | .653 | .807 | .002 |
| AB2 | <--- | 使用行为 | .767 | .696 | .833 | .002 |
| AB3 | <--- | 使用行为 | .807 | .732 | .872 | .002 |
| AB5 | <--- | 使用行为 | .682 | .599 | .761 | .002 |

##### Variances: (other - Unconstrained)

| Parameter | | | Estimate | Lower | Upper | P |
| --- | --- | --- | --- | --- | --- | --- |
| AI威胁感知 |  |  | 2.183 | 1.685 | 2.683 | .002 |
| e21 |  |  | .994 | .649 | 1.379 | .002 |
| e23 |  |  | 1.232 | .839 | 1.619 | .002 |
| e22 |  |  | 1.135 | .771 | 1.515 | .002 |
| e1 |  |  | 1.144 | .864 | 1.410 | .002 |
| e2 |  |  | 1.193 | .923 | 1.523 | .002 |
| e3 |  |  | 1.341 | 1.074 | 1.621 | .002 |
| e4 |  |  | 1.518 | 1.183 | 1.852 | .002 |
| e5 |  |  | 1.334 | .981 | 1.707 | .002 |
| e6 |  |  | 1.715 | 1.346 | 2.085 | .002 |
| e8 |  |  | 1.622 | 1.216 | 2.005 | .002 |
| e9 |  |  | 1.154 | .841 | 1.426 | .002 |
| e10 |  |  | 1.819 | 1.394 | 2.212 | .002 |
| e11 |  |  | 1.937 | 1.555 | 2.272 | .002 |
| e12 |  |  | 1.717 | 1.340 | 2.066 | .002 |
| e13 |  |  | 1.702 | 1.330 | 2.036 | .002 |
| e14 |  |  | 1.200 | .891 | 1.492 | .002 |
| e15 |  |  | 1.139 | .829 | 1.444 | .002 |
| e16 |  |  | 1.446 | 1.112 | 1.758 | .002 |
| e17 |  |  | 1.388 | 1.051 | 1.724 | .002 |
| e18 |  |  | 1.139 | .777 | 1.480 | .002 |
| e20 |  |  | 1.811 | 1.438 | 2.150 | .002 |

##### Squared Multiple Correlations: (other - Unconstrained)

| Parameter | | | Estimate | Lower | Upper | P |
| --- | --- | --- | --- | --- | --- | --- |
| AI自我效能 |  |  | .158 | .065 | .271 | .002 |
| 防御性动机 |  |  | .221 | .127 | .359 | .002 |
| 使用行为 |  |  | .328 | .221 | .464 | .002 |
| AB5 |  |  | .466 | .359 | .579 | .002 |
| AB3 |  |  | .651 | .536 | .761 | .002 |
| AB2 |  |  | .588 | .485 | .694 | .002 |
| AB1 |  |  | .539 | .426 | .652 | .002 |
| SE5 |  |  | .604 | .491 | .712 | .002 |
| SE4 |  |  | .626 | .519 | .728 | .002 |
| SE3 |  |  | .447 | .334 | .564 | .002 |
| SE2 |  |  | .476 | .354 | .591 | .002 |
| SE1 |  |  | .379 | .266 | .500 | .002 |
| DM5 |  |  | .484 | .358 | .604 | .002 |
| DM3 |  |  | .624 | .511 | .736 | .002 |
| DM2 |  |  | .486 | .370 | .610 | .002 |
| DM1 |  |  | .480 | .367 | .590 | .002 |
| AA5 |  |  | .601 | .492 | .703 | .002 |
| AA4 |  |  | .550 | .441 | .654 | .002 |
| AA3 |  |  | .594 | .499 | .682 | .002 |
| AA2 |  |  | .640 | .536 | .726 | .002 |
| AA1 |  |  | .656 | .557 | .749 | .002 |

##### User-defined estimands: (other - Unconstrained)

| Parameter | | | Estimate | Lower | Upper | P |
| --- | --- | --- | --- | --- | --- | --- |
| diff1 |  |  | .214 | .005 | .409 | .038 |
| diff2 |  |  | .070 | -.204 | .329 | .586 |
| diff3 |  |  | .278 | .009 | .571 | .043 |
| diff4 |  |  | .043 | -.174 | .274 | .751 |
| diff5 |  |  | -.091 | -.356 | .212 | .541 |

##### Bias-corrected percentile method (other - Unconstrained)

##### 95% confidence intervals (bias-corrected percentile method)

##### Scalar Estimates (other - Unconstrained)

##### Regression Weights: (other - Unconstrained)

| Parameter | | | Estimate | Lower | Upper | P |
| --- | --- | --- | --- | --- | --- | --- |
| AI自我效能 | <--- | AI威胁感知 | .292 | .173 | .421 | .002 |
| 防御性动机 | <--- | AI威胁感知 | .405 | .257 | .550 | .003 |
| 防御性动机 | <--- | AI自我效能 | -.016 | -.221 | .170 | .867 |
| 使用行为 | <--- | 防御性动机 | .433 | .286 | .606 | .002 |
| 使用行为 | <--- | AI自我效能 | .387 | .213 | .581 | .002 |
| AA1 | <--- | AI威胁感知 | 1.000 | 1.000 | 1.000 | ... |
| AA2 | <--- | AI威胁感知 | .985 | .865 | 1.140 | .001 |
| AA3 | <--- | AI威胁感知 | .948 | .832 | 1.083 | .002 |
| AA4 | <--- | AI威胁感知 | .921 | .804 | 1.102 | .001 |
| AA5 | <--- | AI威胁感知 | .959 | .840 | 1.134 | .001 |
| DM1 | <--- | 防御性动机 | 1.000 | 1.000 | 1.000 | ... |
| DM2 | <--- | 防御性动机 | .985 | .822 | 1.192 | .002 |
| DM3 | <--- | 防御性动机 | 1.102 | .928 | 1.289 | .003 |
| DM5 | <--- | 防御性动机 | 1.038 | .836 | 1.271 | .002 |
| SE1 | <--- | AI自我效能 | 1.000 | 1.000 | 1.000 | ... |
| SE2 | <--- | AI自我效能 | 1.149 | .922 | 1.467 | .001 |
| SE3 | <--- | AI自我效能 | 1.080 | .883 | 1.368 | .001 |
| SE4 | <--- | AI自我效能 | 1.304 | 1.106 | 1.664 | .001 |
| SE5 | <--- | AI自我效能 | 1.212 | 1.008 | 1.557 | .001 |
| AB1 | <--- | 使用行为 | 1.000 | 1.000 | 1.000 | ... |
| AB2 | <--- | 使用行为 | 1.084 | .929 | 1.281 | .002 |
| AB3 | <--- | 使用行为 | 1.122 | .954 | 1.367 | .002 |
| AB5 | <--- | 使用行为 | .967 | .800 | 1.176 | .002 |

##### Standardized Regression Weights: (other - Unconstrained)

| Parameter | | | Estimate | Lower | Upper | P |
| --- | --- | --- | --- | --- | --- | --- |
| AI自我效能 | <--- | AI威胁感知 | .397 | .254 | .520 | .002 |
| 防御性动机 | <--- | AI威胁感知 | .475 | .310 | .610 | .004 |
| 防御性动机 | <--- | AI自我效能 | -.014 | -.186 | .149 | .875 |
| 使用行为 | <--- | 防御性动机 | .419 | .278 | .543 | .003 |
| 使用行为 | <--- | AI自我效能 | .324 | .187 | .457 | .002 |
| AA1 | <--- | AI威胁感知 | .810 | .740 | .861 | .003 |
| AA2 | <--- | AI威胁感知 | .800 | .732 | .852 | .002 |
| AA3 | <--- | AI威胁感知 | .771 | .700 | .823 | .003 |
| AA4 | <--- | AI威胁感知 | .742 | .664 | .809 | .002 |
| AA5 | <--- | AI威胁感知 | .775 | .698 | .835 | .003 |
| DM1 | <--- | 防御性动机 | .693 | .613 | .775 | .001 |
| DM2 | <--- | 防御性动机 | .697 | .608 | .781 | .002 |
| DM3 | <--- | 防御性动机 | .790 | .710 | .855 | .003 |
| DM5 | <--- | 防御性动机 | .695 | .591 | .775 | .003 |
| SE1 | <--- | AI自我效能 | .615 | .508 | .699 | .003 |
| SE2 | <--- | AI自我效能 | .690 | .597 | .769 | .002 |
| SE3 | <--- | AI自我效能 | .669 | .571 | .746 | .003 |
| SE4 | <--- | AI自我效能 | .791 | .713 | .851 | .003 |
| SE5 | <--- | AI自我效能 | .777 | .695 | .836 | .003 |
| AB1 | <--- | 使用行为 | .734 | .640 | .802 | .003 |
| AB2 | <--- | 使用行为 | .767 | .695 | .833 | .002 |
| AB3 | <--- | 使用行为 | .807 | .728 | .869 | .003 |
| AB5 | <--- | 使用行为 | .682 | .590 | .756 | .003 |

##### Variances: (other - Unconstrained)

| Parameter | | | Estimate | Lower | Upper | P |
| --- | --- | --- | --- | --- | --- | --- |
| AI威胁感知 |  |  | 2.183 | 1.654 | 2.659 | .003 |
| e21 |  |  | .994 | .639 | 1.375 | .002 |
| e23 |  |  | 1.232 | .895 | 1.683 | .001 |
| e22 |  |  | 1.135 | .816 | 1.578 | .001 |
| e1 |  |  | 1.144 | .894 | 1.428 | .001 |
| e2 |  |  | 1.193 | .922 | 1.517 | .002 |
| e3 |  |  | 1.341 | 1.106 | 1.639 | .001 |
| e4 |  |  | 1.518 | 1.199 | 1.893 | .001 |
| e5 |  |  | 1.334 | 1.014 | 1.749 | .001 |
| e6 |  |  | 1.715 | 1.369 | 2.101 | .001 |
| e8 |  |  | 1.622 | 1.262 | 2.054 | .001 |
| e9 |  |  | 1.154 | .885 | 1.468 | .001 |
| e10 |  |  | 1.819 | 1.435 | 2.246 | .001 |
| e11 |  |  | 1.937 | 1.591 | 2.312 | .001 |
| e12 |  |  | 1.717 | 1.371 | 2.104 | .001 |
| e13 |  |  | 1.702 | 1.372 | 2.116 | .001 |
| e14 |  |  | 1.200 | .904 | 1.507 | .002 |
| e15 |  |  | 1.139 | .883 | 1.510 | .001 |
| e16 |  |  | 1.446 | 1.167 | 1.807 | .001 |
| e17 |  |  | 1.388 | 1.075 | 1.766 | .001 |
| e18 |  |  | 1.139 | .826 | 1.517 | .001 |
| e20 |  |  | 1.811 | 1.463 | 2.174 | .001 |

##### Squared Multiple Correlations: (other - Unconstrained)

| Parameter | | | Estimate | Lower | Upper | P |
| --- | --- | --- | --- | --- | --- | --- |
| AI自我效能 |  |  | .158 | .065 | .271 | .002 |
| 防御性动机 |  |  | .221 | .106 | .335 | .006 |
| 使用行为 |  |  | .328 | .209 | .449 | .004 |
| AB5 |  |  | .466 | .349 | .571 | .003 |
| AB3 |  |  | .651 | .530 | .755 | .003 |
| AB2 |  |  | .588 | .483 | .694 | .002 |
| AB1 |  |  | .539 | .410 | .643 | .003 |
| SE5 |  |  | .604 | .483 | .699 | .003 |
| SE4 |  |  | .626 | .509 | .724 | .003 |
| SE3 |  |  | .447 | .326 | .557 | .003 |
| SE2 |  |  | .476 | .357 | .592 | .002 |
| SE1 |  |  | .379 | .258 | .488 | .003 |
| DM5 |  |  | .484 | .350 | .601 | .003 |
| DM3 |  |  | .624 | .504 | .730 | .003 |
| DM2 |  |  | .486 | .369 | .609 | .002 |
| DM1 |  |  | .480 | .376 | .600 | .001 |
| AA5 |  |  | .601 | .487 | .697 | .003 |
| AA4 |  |  | .550 | .441 | .654 | .002 |
| AA3 |  |  | .594 | .490 | .678 | .003 |
| AA2 |  |  | .640 | .536 | .726 | .002 |
| AA1 |  |  | .656 | .547 | .742 | .003 |

##### User-defined estimands: (other - Unconstrained)

| Parameter | | | Estimate | Lower | Upper | P |
| --- | --- | --- | --- | --- | --- | --- |
| diff1 |  |  | .214 | .001 | .402 | .046 |
| diff2 |  |  | .070 | -.201 | .330 | .576 |
| diff3 |  |  | .278 | .036 | .599 | .030 |
| diff4 |  |  | .043 | -.168 | .287 | .651 |
| diff5 |  |  | -.091 | -.356 | .212 | .541 |

##### Minimization History (Unconstrained)

| Iteration |  | Negative eigenvalues | Condition # | Smallest eigenvalue | Diameter | F | NTries | Ratio |
| --- | --- | --- | --- | --- | --- | --- | --- | --- |
| 0 | e | 8 |  | -.589 | 9999.000 | 3457.179 | 0 | 9999.000 |
| 1 | e | 1 |  | -.007 | 3.513 | 784.158 | 20 | .457 |
| 2 | e | 0 | 68.457 |  | 1.773 | 374.742 | 5 | .814 |
| 3 | e | 0 | 28.868 |  | 1.022 | 297.026 | 2 | .000 |
| 4 | e | 0 | 24.272 |  | .670 | 261.026 | 1 | 1.126 |
| 5 | e | 0 | 31.079 |  | .269 | 258.073 | 1 | 1.092 |
| 6 | e | 0 | 31.887 |  | .064 | 257.968 | 1 | 1.043 |
| 7 | e | 0 | 32.795 |  | .006 | 257.968 | 1 | 1.006 |
| 8 | e | 0 | 32.792 |  | .000 | 257.968 | 1 | 1.000 |

##### Bootstrap (Unconstrained)

##### Summary of Bootstrap Iterations (Unconstrained)

##### (Unconstrained)

| Iterations | Method 0 | Method 1 | Method 2 |
| --- | --- | --- | --- |
| 1 | 0 | 0 | 0 |
| 2 | 0 | 0 | 0 |
| 3 | 0 | 0 | 0 |
| 4 | 0 | 0 | 0 |
| 5 | 0 | 0 | 0 |
| 6 | 0 | 13 | 0 |
| 7 | 0 | 119 | 0 |
| 8 | 0 | 248 | 0 |
| 9 | 0 | 270 | 0 |
| 10 | 0 | 160 | 0 |
| 11 | 0 | 96 | 0 |
| 12 | 0 | 51 | 0 |
| 13 | 0 | 20 | 0 |
| 14 | 0 | 13 | 0 |
| 15 | 0 | 9 | 0 |
| 16 | 0 | 1 | 0 |
| 17 | 0 | 0 | 0 |
| 18 | 0 | 0 | 0 |
| 19 | 0 | 0 | 0 |
| Total | 0 | 1000 | 0 |

0 bootstrap samples were unused because of a singular covariance matrix.

0 bootstrap samples were unused because a solution was not found.

1000 usable bootstrap samples were obtained.

##### Bootstrap Distributions (Unconstrained)

##### ML discrepancy (implied vs sample) (Unconstrained)

|  |  |  |
| --- | --- | --- |
|  |  | \|-------------------- |
|  | 418.663 | \|\* |
|  | 439.656 | \|\*\* |
|  | 460.648 | \|\*\*\*\*\* |
|  | 481.640 | \|\*\*\*\*\*\*\*\*\* |
|  | 502.632 | \|\*\*\*\*\*\*\*\*\*\*\*\* |
|  | 523.625 | \|\*\*\*\*\*\*\*\*\*\*\*\*\*\*\*\*\*\*\*\* |
|  | 544.617 | \|\*\*\*\*\*\*\*\*\*\*\*\*\*\*\*\*\*\* |
| N = 1000 | 565.609 | \|\*\*\*\*\*\*\*\*\*\*\*\*\*\*\*\*\* |
| Mean = 540.565 | 586.602 | \|\*\*\*\*\*\*\*\*\* |
| S. e. = 1.474 | 607.594 | \|\*\*\*\*\*\*\*\* |
|  | 628.586 | \|\*\*\* |
|  | 649.578 | \|\* |
|  | 670.571 | \|\*\* |
|  | 691.563 | \| |
|  | 712.555 | \|\* |
|  |  | \|-------------------- |

##### ML discrepancy (implied vs pop) (Unconstrained)

|  |  |  |
| --- | --- | --- |
|  |  | \|-------------------- |
|  | 321.054 | \|\* |
|  | 329.168 | \|\*\* |
|  | 337.282 | \|\*\*\*\* |
|  | 345.395 | \|\*\*\*\*\*\*\*\*\* |
|  | 353.509 | \|\*\*\*\*\*\*\*\*\*\*\*\*\*\* |
|  | 361.623 | \|\*\*\*\*\*\*\*\*\*\*\*\*\*\*\*\*\*\*\*\* |
|  | 369.736 | \|\*\*\*\*\*\*\*\*\*\*\*\*\*\*\* |
| N = 1000 | 377.850 | \|\*\*\*\*\*\*\*\*\*\*\*\*\*\* |
| Mean = 367.217 | 385.964 | \|\*\*\*\*\*\*\*\* |
| S. e. = .554 | 394.077 | \|\*\*\*\*\* |
|  | 402.191 | \|\*\*\*\* |
|  | 410.305 | \|\* |
|  | 418.418 | \|\* |
|  | 426.532 | \|\* |
|  | 434.646 | \|\* |
|  |  | \|-------------------- |

##### K-L overoptimism (unstabilized) (Unconstrained)

|  |  |  |
| --- | --- | --- |
|  |  | \|-------------------- |
|  | -129.799 | \|\* |
|  | -75.567 | \|\* |
|  | -21.336 | \|\*\*\* |
|  | 32.896 | \|\*\*\*\*\*\* |
|  | 87.128 | \|\*\*\*\*\*\*\*\*\*\*\* |
|  | 141.360 | \|\*\*\*\*\*\*\*\*\*\*\*\*\*\*\* |
|  | 195.591 | \|\*\*\*\*\*\*\*\*\*\*\*\*\*\*\*\*\*\* |
| N = 1000 | 249.823 | \|\*\*\*\*\*\*\*\*\*\*\*\*\*\*\*\*\*\*\* |
| Mean = 219.224 | 304.055 | \|\*\*\*\*\*\*\*\*\*\*\*\*\* |
| S. e. = 3.671 | 358.287 | \|\*\*\*\*\*\*\*\*\*\* |
|  | 412.518 | \|\*\*\*\*\* |
|  | 466.750 | \|\*\*\* |
|  | 520.982 | \|\* |
|  | 575.214 | \|\* |
|  | 629.445 | \|\* |
|  |  | \|-------------------- |

##### K-L overoptimism (stabilized) (Unconstrained)

|  |  |  |
| --- | --- | --- |
|  |  | \|-------------------- |
|  | 71.525 | \|\* |
|  | 94.124 | \|\* |
|  | 116.724 | \|\*\* |
|  | 139.323 | \|\*\*\* |
|  | 161.923 | \|\*\*\*\*\*\*\*\*\* |
|  | 184.522 | \|\*\*\*\*\*\*\*\*\*\*\*\*\*\*\* |
|  | 207.121 | \|\*\*\*\*\*\*\*\*\*\*\*\*\*\*\*\*\*\*\* |
| N = 1000 | 229.721 | \|\*\*\*\*\*\*\*\*\*\*\*\*\*\*\*\* |
| Mean = 213.933 | 252.320 | \|\*\*\*\*\*\*\*\*\*\*\*\*\* |
| S. e. = 1.327 | 274.920 | \|\*\*\*\*\* |
|  | 297.519 | \|\*\*\* |
|  | 320.119 | \|\*\* |
|  | 342.718 | \|\* |
|  | 365.317 | \|\* |
|  | 387.917 | \|\* |
|  |  | \|-------------------- |

##### Measurement weights (Measurement weights)

##### Notes for Model (Measurement weights)

##### Computation of degrees of freedom (Measurement weights)

|  |  |
| --- | --- |
| Number of distinct sample moments: | 342 |
| Number of distinct parameters to be estimated: | 68 |
| Degrees of freedom (342 - 68): | 274 |

##### Result (Measurement weights)

Minimum was achieved

Chi-square = 264.985

Degrees of freedom = 274

Probability level = .641

##### east (east - Measurement weights)

##### Estimates (east - Measurement weights)

##### Scalar Estimates (east - Measurement weights)

##### Maximum Likelihood Estimates

##### Regression Weights: (east - Measurement weights)

|  |  |  | Estimate | S.E. | C.R. | P | Label |
| --- | --- | --- | --- | --- | --- | --- | --- |
| AI自我效能 | <--- | AI威胁感知 | .518 | .076 | 6.808 | \*\*\* | H1a |
| 防御性动机 | <--- | AI威胁感知 | .367 | .104 | 3.517 | \*\*\* | H2a |
| 防御性动机 | <--- | AI自我效能 | .192 | .120 | 1.601 | .109 | H5a |
| 使用行为 | <--- | 防御性动机 | .326 | .095 | 3.430 | \*\*\* | H4a |
| 使用行为 | <--- | AI自我效能 | .547 | .105 | 5.185 | \*\*\* | H3a |
| AA1 | <--- | AI威胁感知 | 1.000 |  |  |  |  |
| AA2 | <--- | AI威胁感知 | .953 | .057 | 16.817 | \*\*\* | a1\_1 |
| AA3 | <--- | AI威胁感知 | .921 | .058 | 15.942 | \*\*\* | a2\_1 |
| AA4 | <--- | AI威胁感知 | .919 | .058 | 15.955 | \*\*\* | a3\_1 |
| AA5 | <--- | AI威胁感知 | .940 | .056 | 16.731 | \*\*\* | a4\_1 |
| DM1 | <--- | 防御性动机 | 1.000 |  |  |  |  |
| DM2 | <--- | 防御性动机 | 1.027 | .082 | 12.571 | \*\*\* | a5\_1 |
| DM3 | <--- | 防御性动机 | 1.049 | .082 | 12.771 | \*\*\* | a6\_1 |
| DM5 | <--- | 防御性动机 | .976 | .081 | 12.087 | \*\*\* | a7\_1 |
| SE1 | <--- | AI自我效能 | 1.000 |  |  |  |  |
| SE2 | <--- | AI自我效能 | 1.153 | .100 | 11.546 | \*\*\* | a8\_1 |
| SE3 | <--- | AI自我效能 | 1.098 | .096 | 11.415 | \*\*\* | a9\_1 |
| SE4 | <--- | AI自我效能 | 1.271 | .101 | 12.577 | \*\*\* | a10\_1 |
| SE5 | <--- | AI自我效能 | 1.220 | .098 | 12.454 | \*\*\* | a11\_1 |
| AB1 | <--- | 使用行为 | 1.000 |  |  |  |  |
| AB2 | <--- | 使用行为 | .980 | .071 | 13.769 | \*\*\* | a12\_1 |
| AB3 | <--- | 使用行为 | 1.068 | .071 | 14.967 | \*\*\* | a13\_1 |
| AB5 | <--- | 使用行为 | .970 | .071 | 13.673 | \*\*\* | a14\_1 |

##### Standardized Regression Weights: (east - Measurement weights)

|  |  |  | Estimate |
| --- | --- | --- | --- |
| AI自我效能 | <--- | AI威胁感知 | .603 |
| 防御性动机 | <--- | AI威胁感知 | .399 |
| 防御性动机 | <--- | AI自我效能 | .179 |
| 使用行为 | <--- | 防御性动机 | .308 |
| 使用行为 | <--- | AI自我效能 | .481 |
| AA1 | <--- | AI威胁感知 | .822 |
| AA2 | <--- | AI威胁感知 | .769 |
| AA3 | <--- | AI威胁感知 | .729 |
| AA4 | <--- | AI威胁感知 | .759 |
| AA5 | <--- | AI威胁感知 | .789 |
| DM1 | <--- | 防御性动机 | .717 |
| DM2 | <--- | 防御性动机 | .767 |
| DM3 | <--- | 防御性动机 | .743 |
| DM5 | <--- | 防御性动机 | .747 |
| SE1 | <--- | AI自我效能 | .670 |
| SE2 | <--- | AI自我效能 | .738 |
| SE3 | <--- | AI自我效能 | .732 |
| SE4 | <--- | AI自我效能 | .827 |
| SE5 | <--- | AI自我效能 | .806 |
| AB1 | <--- | 使用行为 | .765 |
| AB2 | <--- | 使用行为 | .736 |
| AB3 | <--- | 使用行为 | .817 |
| AB5 | <--- | 使用行为 | .769 |

##### Variances: (east - Measurement weights)

|  |  |  | Estimate | S.E. | C.R. | P | Label |
| --- | --- | --- | --- | --- | --- | --- | --- |
| AI威胁感知 |  |  | 2.087 | .302 | 6.905 | \*\*\* | vvv1\_1 |
| e21 |  |  | .978 | .189 | 5.183 | \*\*\* | vv1\_1 |
| e23 |  |  | 1.277 | .233 | 5.483 | \*\*\* | vv3\_1 |
| e22 |  |  | 1.092 | .198 | 5.517 | \*\*\* | vv2\_1 |
| e1 |  |  | 1.003 | .149 | 6.716 | \*\*\* | v1\_1 |
| e2 |  |  | 1.312 | .178 | 7.377 | \*\*\* | v2\_1 |
| e3 |  |  | 1.565 | .203 | 7.700 | \*\*\* | v3\_1 |
| e4 |  |  | 1.298 | .174 | 7.459 | \*\*\* | v4\_1 |
| e5 |  |  | 1.121 | .157 | 7.162 | \*\*\* | v5\_1 |
| e6 |  |  | 1.672 | .231 | 7.230 | \*\*\* | v6\_1 |
| e8 |  |  | 1.302 | .196 | 6.637 | \*\*\* | v7\_1 |
| e9 |  |  | 1.580 | .227 | 6.967 | \*\*\* | v8\_1 |
| e10 |  |  | 1.332 | .193 | 6.892 | \*\*\* | v9\_1 |
| e11 |  |  | 1.891 | .237 | 7.983 | \*\*\* | v10\_1 |
| e12 |  |  | 1.708 | .226 | 7.572 | \*\*\* | v11\_1 |
| e13 |  |  | 1.604 | .211 | 7.617 | \*\*\* | v12\_1 |
| e14 |  |  | 1.144 | .175 | 6.520 | \*\*\* | v13\_1 |
| e15 |  |  | 1.238 | .180 | 6.869 | \*\*\* | v14\_1 |
| e16 |  |  | 1.409 | .200 | 7.062 | \*\*\* | v15\_1 |
| e17 |  |  | 1.618 | .220 | 7.364 | \*\*\* | v16\_1 |
| e18 |  |  | 1.130 | .180 | 6.294 | \*\*\* | v17\_1 |
| e20 |  |  | 1.294 | .185 | 7.003 | \*\*\* | v18\_1 |

##### Squared Multiple Correlations: (east - Measurement weights)

|  |  |  | Estimate |
| --- | --- | --- | --- |
| AI自我效能 |  |  | .364 |
| 防御性动机 |  |  | .278 |
| 使用行为 |  |  | .450 |
| AB5 |  |  | .591 |
| AB3 |  |  | .667 |
| AB2 |  |  | .541 |
| AB1 |  |  | .585 |
| SE5 |  |  | .649 |
| SE4 |  |  | .685 |
| SE3 |  |  | .536 |
| SE2 |  |  | .545 |
| SE1 |  |  | .449 |
| DM5 |  |  | .558 |
| DM3 |  |  | .552 |
| DM2 |  |  | .589 |
| DM1 |  |  | .514 |
| AA5 |  |  | .622 |
| AA4 |  |  | .576 |
| AA3 |  |  | .531 |
| AA2 |  |  | .591 |
| AA1 |  |  | .675 |

##### User-defined estimands: (east - Measurement weights)

|  |  |  |  |
| --- | --- | --- | --- |
| diff1 |  |  | .231 |
| diff2 |  |  | .080 |
| diff3 |  |  | .260 |
| diff4 |  |  | .039 |
| diff5 |  |  | -.095 |

##### Modification Indices (east - Measurement weights)

##### Covariances: (east - Measurement weights)

|  |  |  | M.I. | Par Change |
| --- | --- | --- | --- | --- |
| e17 | <--> | e22 | 4.050 | -.266 |
| e13 | <--> | e16 | 7.311 | -.385 |
| e4 | <--> | e17 | 4.522 | .290 |
| e4 | <--> | e11 | 4.952 | .316 |
| e2 | <--> | e8 | 4.285 | -.267 |
| e1 | <--> | e15 | 5.137 | .257 |

##### Variances: (east - Measurement weights)

|  |  |  | M.I. | Par Change |
| --- | --- | --- | --- | --- |

##### Regression Weights: (east - Measurement weights)

|  |  |  | M.I. | Par Change |
| --- | --- | --- | --- | --- |
| SE2 | <--- | DM5 | 4.959 | -.144 |
| DM5 | <--- | AB5 | 5.212 | -.132 |
| DM5 | <--- | SE2 | 4.819 | -.117 |

##### Bootstrap (east - Measurement weights)

##### Bootstrap standard errors (east - Measurement weights)

##### Scalar Estimates (east - Measurement weights)

##### Regression Weights: (east - Measurement weights)

| Parameter | | | SE | SE-SE | Mean | Bias | SE-Bias |
| --- | --- | --- | --- | --- | --- | --- | --- |
| AI自我效能 | <--- | AI威胁感知 | .066 | .001 | .522 | .004 | .002 |
| 防御性动机 | <--- | AI威胁感知 | .113 | .003 | .369 | .001 | .004 |
| 防御性动机 | <--- | AI自我效能 | .124 | .003 | .198 | .006 | .004 |
| 使用行为 | <--- | 防御性动机 | .095 | .002 | .324 | -.002 | .003 |
| 使用行为 | <--- | AI自我效能 | .107 | .002 | .545 | -.001 | .003 |
| AA1 | <--- | AI威胁感知 | .000 | .000 | 1.000 | .000 | .000 |
| AA2 | <--- | AI威胁感知 | .048 | .001 | .952 | -.001 | .002 |
| AA3 | <--- | AI威胁感知 | .046 | .001 | .923 | .002 | .001 |
| AA4 | <--- | AI威胁感知 | .051 | .001 | .919 | .000 | .002 |
| AA5 | <--- | AI威胁感知 | .052 | .001 | .942 | .002 | .002 |
| DM1 | <--- | 防御性动机 | .000 | .000 | 1.000 | .000 | .000 |
| DM2 | <--- | 防御性动机 | .073 | .002 | 1.032 | .005 | .002 |
| DM3 | <--- | 防御性动机 | .075 | .002 | 1.056 | .007 | .002 |
| DM5 | <--- | 防御性动机 | .079 | .002 | .980 | .004 | .002 |
| SE1 | <--- | AI自我效能 | .000 | .000 | 1.000 | .000 | .000 |
| SE2 | <--- | AI自我效能 | .092 | .002 | 1.149 | -.005 | .003 |
| SE3 | <--- | AI自我效能 | .088 | .002 | 1.099 | .001 | .003 |
| SE4 | <--- | AI自我效能 | .096 | .002 | 1.267 | -.003 | .003 |
| SE5 | <--- | AI自我效能 | .094 | .002 | 1.220 | .000 | .003 |
| AB1 | <--- | 使用行为 | .000 | .000 | 1.000 | .000 | .000 |
| AB2 | <--- | 使用行为 | .060 | .001 | .982 | .002 | .002 |
| AB3 | <--- | 使用行为 | .063 | .001 | 1.071 | .002 | .002 |
| AB5 | <--- | 使用行为 | .065 | .001 | .972 | .002 | .002 |

##### Standardized Regression Weights: (east - Measurement weights)

| Parameter | | | SE | SE-SE | Mean | Bias | SE-Bias |
| --- | --- | --- | --- | --- | --- | --- | --- |
| AI自我效能 | <--- | AI威胁感知 | .061 | .001 | .604 | .001 | .002 |
| 防御性动机 | <--- | AI威胁感知 | .114 | .003 | .399 | .000 | .004 |
| 防御性动机 | <--- | AI自我效能 | .116 | .003 | .186 | .007 | .004 |
| 使用行为 | <--- | 防御性动机 | .088 | .002 | .306 | -.002 | .003 |
| 使用行为 | <--- | AI自我效能 | .082 | .002 | .480 | -.001 | .003 |
| AA1 | <--- | AI威胁感知 | .032 | .001 | .821 | -.001 | .001 |
| AA2 | <--- | AI威胁感知 | .030 | .001 | .768 | -.001 | .001 |
| AA3 | <--- | AI威胁感知 | .034 | .001 | .730 | .001 | .001 |
| AA4 | <--- | AI威胁感知 | .032 | .001 | .759 | .000 | .001 |
| AA5 | <--- | AI威胁感知 | .029 | .001 | .790 | .001 | .001 |
| DM1 | <--- | 防御性动机 | .041 | .001 | .717 | .000 | .001 |
| DM2 | <--- | 防御性动机 | .039 | .001 | .768 | .001 | .001 |
| DM3 | <--- | 防御性动机 | .036 | .001 | .745 | .002 | .001 |
| DM5 | <--- | 防御性动机 | .037 | .001 | .747 | .000 | .001 |
| SE1 | <--- | AI自我效能 | .042 | .001 | .672 | .002 | .001 |
| SE2 | <--- | AI自我效能 | .043 | .001 | .737 | -.001 | .001 |
| SE3 | <--- | AI自我效能 | .037 | .001 | .734 | .002 | .001 |
| SE4 | <--- | AI自我效能 | .028 | .001 | .827 | .000 | .001 |
| SE5 | <--- | AI自我效能 | .033 | .001 | .807 | .002 | .001 |
| AB1 | <--- | 使用行为 | .039 | .001 | .765 | .000 | .001 |
| AB2 | <--- | 使用行为 | .038 | .001 | .737 | .001 | .001 |
| AB3 | <--- | 使用行为 | .031 | .001 | .817 | .000 | .001 |
| AB5 | <--- | 使用行为 | .038 | .001 | .769 | .000 | .001 |

##### Variances: (east - Measurement weights)

| Parameter | | | SE | SE-SE | Mean | Bias | SE-Bias |
| --- | --- | --- | --- | --- | --- | --- | --- |
| AI威胁感知 |  |  | .229 | .005 | 2.085 | -.001 | .007 |
| e21 |  |  | .185 | .004 | .983 | .005 | .006 |
| e23 |  |  | .224 | .005 | 1.242 | -.035 | .007 |
| e22 |  |  | .186 | .004 | 1.074 | -.017 | .006 |
| e1 |  |  | .166 | .004 | 1.005 | .002 | .005 |
| e2 |  |  | .155 | .003 | 1.303 | -.008 | .005 |
| e3 |  |  | .189 | .004 | 1.549 | -.017 | .006 |
| e4 |  |  | .163 | .004 | 1.286 | -.012 | .005 |
| e5 |  |  | .134 | .003 | 1.103 | -.019 | .004 |
| e6 |  |  | .225 | .005 | 1.659 | -.013 | .007 |
| e8 |  |  | .186 | .004 | 1.288 | -.014 | .006 |
| e9 |  |  | .211 | .005 | 1.560 | -.020 | .007 |
| e10 |  |  | .171 | .004 | 1.321 | -.011 | .005 |
| e11 |  |  | .212 | .005 | 1.872 | -.019 | .007 |
| e12 |  |  | .269 | .006 | 1.700 | -.009 | .009 |
| e13 |  |  | .183 | .004 | 1.583 | -.021 | .006 |
| e14 |  |  | .172 | .004 | 1.135 | -.009 | .005 |
| e15 |  |  | .189 | .004 | 1.217 | -.021 | .006 |
| e16 |  |  | .196 | .004 | 1.392 | -.018 | .006 |
| e17 |  |  | .236 | .005 | 1.596 | -.022 | .007 |
| e18 |  |  | .163 | .004 | 1.116 | -.014 | .005 |
| e20 |  |  | .182 | .004 | 1.280 | -.014 | .006 |

##### Squared Multiple Correlations: (east - Measurement weights)

| Parameter | | | SE | SE-SE | Mean | Bias | SE-Bias |
| --- | --- | --- | --- | --- | --- | --- | --- |
| AI自我效能 |  |  | .072 | .002 | .368 | .004 | .002 |
| 防御性动机 |  |  | .079 | .002 | .298 | .020 | .002 |
| 使用行为 |  |  | .078 | .002 | .457 | .007 | .002 |
| AB5 |  |  | .058 | .001 | .593 | .001 | .002 |
| AB3 |  |  | .050 | .001 | .669 | .002 | .002 |
| AB2 |  |  | .056 | .001 | .545 | .004 | .002 |
| AB1 |  |  | .059 | .001 | .587 | .002 | .002 |
| SE5 |  |  | .052 | .001 | .653 | .004 | .002 |
| SE4 |  |  | .047 | .001 | .685 | .000 | .001 |
| SE3 |  |  | .054 | .001 | .540 | .004 | .002 |
| SE2 |  |  | .064 | .001 | .545 | .000 | .002 |
| SE1 |  |  | .056 | .001 | .453 | .005 | .002 |
| DM5 |  |  | .055 | .001 | .560 | .002 | .002 |
| DM3 |  |  | .053 | .001 | .556 | .004 | .002 |
| DM2 |  |  | .060 | .001 | .592 | .003 | .002 |
| DM1 |  |  | .059 | .001 | .516 | .002 | .002 |
| AA5 |  |  | .046 | .001 | .625 | .003 | .001 |
| AA4 |  |  | .049 | .001 | .577 | .001 | .002 |
| AA3 |  |  | .050 | .001 | .534 | .003 | .002 |
| AA2 |  |  | .046 | .001 | .591 | .000 | .001 |
| AA1 |  |  | .052 | .001 | .674 | -.001 | .002 |

##### User-defined estimands: (east - Measurement weights)

| Parameter | | | SE | SE-SE | Mean | Bias | SE-Bias |
| --- | --- | --- | --- | --- | --- | --- | --- |
| diff1 |  |  | .082 | .002 | .232 | .001 | .003 |
| diff2 |  |  | .126 | .003 | .079 | -.002 | .004 |
| diff3 |  |  | .127 | .003 | .256 | -.004 | .004 |
| diff4 |  |  | .112 | .003 | .034 | -.005 | .004 |
| diff5 |  |  | .139 | .003 | -.092 | .003 | .004 |

##### Bootstrap Confidence (east - Measurement weights)

##### Percentile method (east - Measurement weights)

##### 95% confidence intervals (percentile method)

##### Scalar Estimates (east - Measurement weights)

##### Regression Weights: (east - Measurement weights)

| Parameter | | | Estimate | Lower | Upper | P |
| --- | --- | --- | --- | --- | --- | --- |
| AI自我效能 | <--- | AI威胁感知 | .518 | .394 | .658 | .002 |
| 防御性动机 | <--- | AI威胁感知 | .367 | .147 | .585 | .006 |
| 防御性动机 | <--- | AI自我效能 | .192 | -.038 | .460 | .101 |
| 使用行为 | <--- | 防御性动机 | .326 | .139 | .514 | .002 |
| 使用行为 | <--- | AI自我效能 | .547 | .323 | .769 | .002 |
| AA1 | <--- | AI威胁感知 | 1.000 | 1.000 | 1.000 | ... |
| AA2 | <--- | AI威胁感知 | .953 | .863 | 1.048 | .002 |
| AA3 | <--- | AI威胁感知 | .921 | .835 | 1.017 | .002 |
| AA4 | <--- | AI威胁感知 | .919 | .829 | 1.026 | .002 |
| AA5 | <--- | AI威胁感知 | .940 | .842 | 1.052 | .002 |
| DM1 | <--- | 防御性动机 | 1.000 | 1.000 | 1.000 | ... |
| DM2 | <--- | 防御性动机 | 1.027 | .890 | 1.172 | .002 |
| DM3 | <--- | 防御性动机 | 1.049 | .912 | 1.209 | .002 |
| DM5 | <--- | 防御性动机 | .976 | .837 | 1.137 | .002 |
| SE1 | <--- | AI自我效能 | 1.000 | 1.000 | 1.000 | ... |
| SE2 | <--- | AI自我效能 | 1.153 | .974 | 1.342 | .002 |
| SE3 | <--- | AI自我效能 | 1.098 | .943 | 1.280 | .002 |
| SE4 | <--- | AI自我效能 | 1.271 | 1.101 | 1.474 | .002 |
| SE5 | <--- | AI自我效能 | 1.220 | 1.055 | 1.432 | .002 |
| AB1 | <--- | 使用行为 | 1.000 | 1.000 | 1.000 | ... |
| AB2 | <--- | 使用行为 | .980 | .867 | 1.109 | .002 |
| AB3 | <--- | 使用行为 | 1.068 | .956 | 1.202 | .002 |
| AB5 | <--- | 使用行为 | .970 | .854 | 1.117 | .002 |

##### Standardized Regression Weights: (east - Measurement weights)

| Parameter | | | Estimate | Lower | Upper | P |
| --- | --- | --- | --- | --- | --- | --- |
| AI自我效能 | <--- | AI威胁感知 | .603 | .475 | .714 | .002 |
| 防御性动机 | <--- | AI威胁感知 | .399 | .169 | .598 | .006 |
| 防御性动机 | <--- | AI自我效能 | .179 | -.030 | .420 | .101 |
| 使用行为 | <--- | 防御性动机 | .308 | .130 | .468 | .002 |
| 使用行为 | <--- | AI自我效能 | .481 | .294 | .633 | .002 |
| AA1 | <--- | AI威胁感知 | .822 | .755 | .879 | .002 |
| AA2 | <--- | AI威胁感知 | .769 | .708 | .824 | .002 |
| AA3 | <--- | AI威胁感知 | .729 | .656 | .795 | .002 |
| AA4 | <--- | AI威胁感知 | .759 | .692 | .819 | .002 |
| AA5 | <--- | AI威胁感知 | .789 | .732 | .847 | .002 |
| DM1 | <--- | 防御性动机 | .717 | .638 | .794 | .002 |
| DM2 | <--- | 防御性动机 | .767 | .696 | .845 | .002 |
| DM3 | <--- | 防御性动机 | .743 | .671 | .812 | .002 |
| DM5 | <--- | 防御性动机 | .747 | .670 | .816 | .002 |
| SE1 | <--- | AI自我效能 | .670 | .580 | .752 | .002 |
| SE2 | <--- | AI自我效能 | .738 | .646 | .821 | .002 |
| SE3 | <--- | AI自我效能 | .732 | .659 | .806 | .002 |
| SE4 | <--- | AI自我效能 | .827 | .768 | .878 | .002 |
| SE5 | <--- | AI自我效能 | .806 | .738 | .869 | .002 |
| AB1 | <--- | 使用行为 | .765 | .682 | .835 | .002 |
| AB2 | <--- | 使用行为 | .736 | .660 | .811 | .002 |
| AB3 | <--- | 使用行为 | .817 | .755 | .874 | .002 |
| AB5 | <--- | 使用行为 | .769 | .693 | .841 | .002 |

##### Variances: (east - Measurement weights)

| Parameter | | | Estimate | Lower | Upper | P |
| --- | --- | --- | --- | --- | --- | --- |
| AI威胁感知 |  |  | 2.087 | 1.635 | 2.551 | .002 |
| e21 |  |  | .978 | .668 | 1.381 | .002 |
| e23 |  |  | 1.277 | .828 | 1.730 | .002 |
| e22 |  |  | 1.092 | .709 | 1.446 | .002 |
| e1 |  |  | 1.003 | .693 | 1.333 | .002 |
| e2 |  |  | 1.312 | 1.010 | 1.615 | .002 |
| e3 |  |  | 1.565 | 1.211 | 1.957 | .002 |
| e4 |  |  | 1.298 | .973 | 1.605 | .002 |
| e5 |  |  | 1.121 | .844 | 1.359 | .002 |
| e6 |  |  | 1.672 | 1.250 | 2.105 | .002 |
| e8 |  |  | 1.302 | .888 | 1.637 | .002 |
| e9 |  |  | 1.580 | 1.177 | 2.007 | .002 |
| e10 |  |  | 1.332 | .983 | 1.677 | .002 |
| e11 |  |  | 1.891 | 1.477 | 2.316 | .002 |
| e12 |  |  | 1.708 | 1.184 | 2.251 | .002 |
| e13 |  |  | 1.604 | 1.232 | 1.952 | .002 |
| e14 |  |  | 1.144 | .816 | 1.493 | .002 |
| e15 |  |  | 1.238 | .867 | 1.603 | .002 |
| e16 |  |  | 1.409 | 1.018 | 1.797 | .002 |
| e17 |  |  | 1.618 | 1.148 | 2.090 | .002 |
| e18 |  |  | 1.130 | .803 | 1.439 | .002 |
| e20 |  |  | 1.294 | .930 | 1.640 | .002 |

##### Squared Multiple Correlations: (east - Measurement weights)

| Parameter | | | Estimate | Lower | Upper | P |
| --- | --- | --- | --- | --- | --- | --- |
| AI自我效能 |  |  | .364 | .226 | .509 | .002 |
| 防御性动机 |  |  | .278 | .154 | .451 | .002 |
| 使用行为 |  |  | .450 | .307 | .613 | .002 |
| AB5 |  |  | .591 | .480 | .707 | .002 |
| AB3 |  |  | .667 | .570 | .763 | .002 |
| AB2 |  |  | .541 | .435 | .657 | .002 |
| AB1 |  |  | .585 | .466 | .697 | .002 |
| SE5 |  |  | .649 | .545 | .754 | .002 |
| SE4 |  |  | .685 | .589 | .771 | .002 |
| SE3 |  |  | .536 | .434 | .649 | .002 |
| SE2 |  |  | .545 | .418 | .673 | .002 |
| SE1 |  |  | .449 | .337 | .565 | .002 |
| DM5 |  |  | .558 | .448 | .667 | .002 |
| DM3 |  |  | .552 | .450 | .659 | .002 |
| DM2 |  |  | .589 | .484 | .714 | .002 |
| DM1 |  |  | .514 | .407 | .630 | .002 |
| AA5 |  |  | .622 | .535 | .717 | .002 |
| AA4 |  |  | .576 | .478 | .671 | .002 |
| AA3 |  |  | .531 | .430 | .631 | .002 |
| AA2 |  |  | .591 | .501 | .679 | .002 |
| AA1 |  |  | .675 | .570 | .773 | .002 |

##### User-defined estimands: (east - Measurement weights)

| Parameter | | | Estimate | Lower | Upper | P |
| --- | --- | --- | --- | --- | --- | --- |
| diff1 |  |  | .231 | .065 | .378 | .005 |
| diff2 |  |  | .080 | -.175 | .316 | .534 |
| diff3 |  |  | .260 | .014 | .524 | .047 |
| diff4 |  |  | .039 | -.171 | .261 | .757 |
| diff5 |  |  | -.095 | -.349 | .192 | .517 |

##### Bias-corrected percentile method (east - Measurement weights)

##### 95% confidence intervals (bias-corrected percentile method)

##### Scalar Estimates (east - Measurement weights)

##### Regression Weights: (east - Measurement weights)

| Parameter | | | Estimate | Lower | Upper | P |
| --- | --- | --- | --- | --- | --- | --- |
| AI自我效能 | <--- | AI威胁感知 | .518 | .388 | .649 | .003 |
| 防御性动机 | <--- | AI威胁感知 | .367 | .136 | .583 | .007 |
| 防御性动机 | <--- | AI自我效能 | .192 | -.031 | .466 | .094 |
| 使用行为 | <--- | 防御性动机 | .326 | .150 | .526 | .002 |
| 使用行为 | <--- | AI自我效能 | .547 | .330 | .772 | .002 |
| AA1 | <--- | AI威胁感知 | 1.000 | 1.000 | 1.000 | ... |
| AA2 | <--- | AI威胁感知 | .953 | .865 | 1.053 | .002 |
| AA3 | <--- | AI威胁感知 | .921 | .829 | 1.014 | .003 |
| AA4 | <--- | AI威胁感知 | .919 | .829 | 1.028 | .002 |
| AA5 | <--- | AI威胁感知 | .940 | .842 | 1.051 | .002 |
| DM1 | <--- | 防御性动机 | 1.000 | 1.000 | 1.000 | ... |
| DM2 | <--- | 防御性动机 | 1.027 | .886 | 1.167 | .003 |
| DM3 | <--- | 防御性动机 | 1.049 | .904 | 1.200 | .003 |
| DM5 | <--- | 防御性动机 | .976 | .836 | 1.136 | .002 |
| SE1 | <--- | AI自我效能 | 1.000 | 1.000 | 1.000 | ... |
| SE2 | <--- | AI自我效能 | 1.153 | .990 | 1.357 | .001 |
| SE3 | <--- | AI自我效能 | 1.098 | .948 | 1.296 | .001 |
| SE4 | <--- | AI自我效能 | 1.271 | 1.113 | 1.484 | .001 |
| SE5 | <--- | AI自我效能 | 1.220 | 1.061 | 1.454 | .001 |
| AB1 | <--- | 使用行为 | 1.000 | 1.000 | 1.000 | ... |
| AB2 | <--- | 使用行为 | .980 | .873 | 1.117 | .001 |
| AB3 | <--- | 使用行为 | 1.068 | .957 | 1.205 | .002 |
| AB5 | <--- | 使用行为 | .970 | .857 | 1.127 | .001 |

##### Standardized Regression Weights: (east - Measurement weights)

| Parameter | | | Estimate | Lower | Upper | P |
| --- | --- | --- | --- | --- | --- | --- |
| AI自我效能 | <--- | AI威胁感知 | .603 | .468 | .710 | .003 |
| 防御性动机 | <--- | AI威胁感知 | .399 | .139 | .590 | .009 |
| 防御性动机 | <--- | AI自我效能 | .179 | -.033 | .420 | .102 |
| 使用行为 | <--- | 防御性动机 | .308 | .133 | .473 | .002 |
| 使用行为 | <--- | AI自我效能 | .481 | .291 | .628 | .002 |
| AA1 | <--- | AI威胁感知 | .822 | .753 | .876 | .002 |
| AA2 | <--- | AI威胁感知 | .769 | .706 | .821 | .002 |
| AA3 | <--- | AI威胁感知 | .729 | .648 | .791 | .003 |
| AA4 | <--- | AI威胁感知 | .759 | .694 | .821 | .002 |
| AA5 | <--- | AI威胁感知 | .789 | .725 | .841 | .003 |
| DM1 | <--- | 防御性动机 | .717 | .635 | .792 | .002 |
| DM2 | <--- | 防御性动机 | .767 | .692 | .843 | .003 |
| DM3 | <--- | 防御性动机 | .743 | .656 | .804 | .005 |
| DM5 | <--- | 防御性动机 | .747 | .666 | .813 | .003 |
| SE1 | <--- | AI自我效能 | .670 | .575 | .744 | .003 |
| SE2 | <--- | AI自我效能 | .738 | .645 | .820 | .002 |
| SE3 | <--- | AI自我效能 | .732 | .655 | .794 | .003 |
| SE4 | <--- | AI自我效能 | .827 | .758 | .875 | .003 |
| SE5 | <--- | AI自我效能 | .806 | .731 | .862 | .004 |
| AB1 | <--- | 使用行为 | .765 | .680 | .833 | .003 |
| AB2 | <--- | 使用行为 | .736 | .656 | .805 | .003 |
| AB3 | <--- | 使用行为 | .817 | .751 | .871 | .003 |
| AB5 | <--- | 使用行为 | .769 | .685 | .834 | .003 |

##### Variances: (east - Measurement weights)

| Parameter | | | Estimate | Lower | Upper | P |
| --- | --- | --- | --- | --- | --- | --- |
| AI威胁感知 |  |  | 2.087 | 1.642 | 2.557 | .002 |
| e21 |  |  | .978 | .678 | 1.411 | .002 |
| e23 |  |  | 1.277 | .913 | 1.839 | .000 |
| e22 |  |  | 1.092 | .768 | 1.517 | .001 |
| e1 |  |  | 1.003 | .696 | 1.343 | .002 |
| e2 |  |  | 1.312 | 1.026 | 1.639 | .001 |
| e3 |  |  | 1.565 | 1.238 | 1.999 | .001 |
| e4 |  |  | 1.298 | 1.008 | 1.630 | .001 |
| e5 |  |  | 1.121 | .887 | 1.413 | .001 |
| e6 |  |  | 1.672 | 1.270 | 2.132 | .001 |
| e8 |  |  | 1.302 | .917 | 1.658 | .001 |
| e9 |  |  | 1.580 | 1.215 | 2.093 | .001 |
| e10 |  |  | 1.332 | 1.024 | 1.719 | .001 |
| e11 |  |  | 1.891 | 1.510 | 2.360 | .001 |
| e12 |  |  | 1.708 | 1.210 | 2.270 | .001 |
| e13 |  |  | 1.604 | 1.285 | 1.986 | .001 |
| e14 |  |  | 1.144 | .845 | 1.532 | .001 |
| e15 |  |  | 1.238 | .911 | 1.640 | .001 |
| e16 |  |  | 1.409 | 1.050 | 1.845 | .001 |
| e17 |  |  | 1.618 | 1.196 | 2.128 | .001 |
| e18 |  |  | 1.130 | .826 | 1.457 | .001 |
| e20 |  |  | 1.294 | .965 | 1.684 | .001 |

##### Squared Multiple Correlations: (east - Measurement weights)

| Parameter | | | Estimate | Lower | Upper | P |
| --- | --- | --- | --- | --- | --- | --- |
| AI自我效能 |  |  | .364 | .219 | .504 | .003 |
| 防御性动机 |  |  | .278 | .113 | .424 | .009 |
| 使用行为 |  |  | .450 | .293 | .597 | .003 |
| AB5 |  |  | .591 | .470 | .695 | .003 |
| AB3 |  |  | .667 | .563 | .759 | .003 |
| AB2 |  |  | .541 | .430 | .648 | .003 |
| AB1 |  |  | .585 | .462 | .694 | .003 |
| SE5 |  |  | .649 | .534 | .743 | .004 |
| SE4 |  |  | .685 | .574 | .766 | .003 |
| SE3 |  |  | .536 | .429 | .630 | .003 |
| SE2 |  |  | .545 | .416 | .672 | .002 |
| SE1 |  |  | .449 | .331 | .554 | .003 |
| DM5 |  |  | .558 | .443 | .662 | .003 |
| DM3 |  |  | .552 | .431 | .646 | .005 |
| DM2 |  |  | .589 | .479 | .711 | .003 |
| DM1 |  |  | .514 | .403 | .627 | .002 |
| AA5 |  |  | .622 | .525 | .707 | .003 |
| AA4 |  |  | .576 | .482 | .673 | .002 |
| AA3 |  |  | .531 | .420 | .625 | .003 |
| AA2 |  |  | .591 | .499 | .674 | .002 |
| AA1 |  |  | .675 | .567 | .768 | .002 |

##### User-defined estimands: (east - Measurement weights)

| Parameter | | | Estimate | Lower | Upper | P |
| --- | --- | --- | --- | --- | --- | --- |
| diff1 |  |  | .231 | .062 | .376 | .006 |
| diff2 |  |  | .080 | -.180 | .314 | .554 |
| diff3 |  |  | .260 | .026 | .546 | .037 |
| diff4 |  |  | .039 | -.158 | .274 | .642 |
| diff5 |  |  | -.095 | -.343 | .198 | .543 |

##### other (other - Measurement weights)

##### Estimates (other - Measurement weights)

##### Scalar Estimates (other - Measurement weights)

##### Maximum Likelihood Estimates

##### Regression Weights: (other - Measurement weights)

|  |  |  | Estimate | S.E. | C.R. | P | Label |
| --- | --- | --- | --- | --- | --- | --- | --- |
| AI自我效能 | <--- | AI威胁感知 | .287 | .056 | 5.124 | \*\*\* | H1 |
| 防御性动机 | <--- | AI威胁感知 | .405 | .072 | 5.604 | \*\*\* | H2 |
| 防御性动机 | <--- | AI自我效能 | -.017 | .096 | -.178 | .859 | H5 |
| 使用行为 | <--- | 防御性动机 | .443 | .081 | 5.447 | \*\*\* | H4 |
| 使用行为 | <--- | AI自我效能 | .407 | .093 | 4.391 | \*\*\* | H3 |
| AA1 | <--- | AI威胁感知 | 1.000 |  |  |  |  |
| AA2 | <--- | AI威胁感知 | .953 | .057 | 16.817 | \*\*\* | a1\_1 |
| AA3 | <--- | AI威胁感知 | .921 | .058 | 15.942 | \*\*\* | a2\_1 |
| AA4 | <--- | AI威胁感知 | .919 | .058 | 15.955 | \*\*\* | a3\_1 |
| AA5 | <--- | AI威胁感知 | .940 | .056 | 16.731 | \*\*\* | a4\_1 |
| DM1 | <--- | 防御性动机 | 1.000 |  |  |  |  |
| DM2 | <--- | 防御性动机 | 1.027 | .082 | 12.571 | \*\*\* | a5\_1 |
| DM3 | <--- | 防御性动机 | 1.049 | .082 | 12.771 | \*\*\* | a6\_1 |
| DM5 | <--- | 防御性动机 | .976 | .081 | 12.087 | \*\*\* | a7\_1 |
| SE1 | <--- | AI自我效能 | 1.000 |  |  |  |  |
| SE2 | <--- | AI自我效能 | 1.153 | .100 | 11.546 | \*\*\* | a8\_1 |
| SE3 | <--- | AI自我效能 | 1.098 | .096 | 11.415 | \*\*\* | a9\_1 |
| SE4 | <--- | AI自我效能 | 1.271 | .101 | 12.577 | \*\*\* | a10\_1 |
| SE5 | <--- | AI自我效能 | 1.220 | .098 | 12.454 | \*\*\* | a11\_1 |
| AB1 | <--- | 使用行为 | 1.000 |  |  |  |  |
| AB2 | <--- | 使用行为 | .980 | .071 | 13.769 | \*\*\* | a12\_1 |
| AB3 | <--- | 使用行为 | 1.068 | .071 | 14.967 | \*\*\* | a13\_1 |
| AB5 | <--- | 使用行为 | .970 | .071 | 13.673 | \*\*\* | a14\_1 |

##### Standardized Regression Weights: (other - Measurement weights)

|  |  |  | Estimate |
| --- | --- | --- | --- |
| AI自我效能 | <--- | AI威胁感知 | .396 |
| 防御性动机 | <--- | AI威胁感知 | .476 |
| 防御性动机 | <--- | AI自我效能 | -.014 |
| 使用行为 | <--- | 防御性动机 | .420 |
| 使用行为 | <--- | AI自我效能 | .328 |
| AA1 | <--- | AI威胁感知 | .816 |
| AA2 | <--- | AI威胁感知 | .793 |
| AA3 | <--- | AI威胁感知 | .766 |
| AA4 | <--- | AI威胁感知 | .747 |
| AA5 | <--- | AI威胁感知 | .774 |
| DM1 | <--- | 防御性动机 | .701 |
| DM2 | <--- | 防御性动机 | .724 |
| DM3 | <--- | 防御性动机 | .775 |
| DM5 | <--- | 防御性动机 | .676 |
| SE1 | <--- | AI自我效能 | .617 |
| SE2 | <--- | AI自我效能 | .692 |
| SE3 | <--- | AI自我效能 | .677 |
| SE4 | <--- | AI自我效能 | .781 |
| SE5 | <--- | AI自我效能 | .781 |
| AB1 | <--- | 使用行为 | .751 |
| AB2 | <--- | 使用行为 | .740 |
| AB3 | <--- | 使用行为 | .802 |
| AB5 | <--- | 使用行为 | .701 |

##### Variances: (other - Measurement weights)

|  |  |  | Estimate | S.E. | C.R. | P | Label |
| --- | --- | --- | --- | --- | --- | --- | --- |
| AI威胁感知 |  |  | 2.256 | .285 | 7.930 | \*\*\* | vvv1\_2 |
| e21 |  |  | 1.001 | .171 | 5.843 | \*\*\* | vv1\_2 |
| e23 |  |  | 1.277 | .207 | 6.168 | \*\*\* | vv3\_2 |
| e22 |  |  | 1.220 | .190 | 6.437 | \*\*\* | vv2\_2 |
| e1 |  |  | 1.132 | .139 | 8.162 | \*\*\* | v1\_2 |
| e2 |  |  | 1.207 | .141 | 8.533 | \*\*\* | v2\_2 |
| e3 |  |  | 1.350 | .152 | 8.892 | \*\*\* | v3\_2 |
| e4 |  |  | 1.512 | .166 | 9.105 | \*\*\* | v4\_2 |
| e5 |  |  | 1.331 | .151 | 8.800 | \*\*\* | v5\_2 |
| e6 |  |  | 1.700 | .196 | 8.662 | \*\*\* | v6\_2 |
| e8 |  |  | 1.570 | .187 | 8.373 | \*\*\* | v7\_2 |
| e9 |  |  | 1.200 | .160 | 7.485 | \*\*\* | v8\_2 |
| e10 |  |  | 1.856 | .207 | 8.946 | \*\*\* | v9\_2 |
| e11 |  |  | 1.932 | .201 | 9.627 | \*\*\* | v10\_2 |
| e12 |  |  | 1.717 | .189 | 9.083 | \*\*\* | v11\_2 |
| e13 |  |  | 1.690 | .183 | 9.216 | \*\*\* | v12\_2 |
| e14 |  |  | 1.227 | .155 | 7.929 | \*\*\* | v13\_2 |
| e15 |  |  | 1.128 | .143 | 7.913 | \*\*\* | v14\_2 |
| e16 |  |  | 1.414 | .170 | 8.330 | \*\*\* | v15\_2 |
| e17 |  |  | 1.449 | .171 | 8.475 | \*\*\* | v16\_2 |
| e18 |  |  | 1.154 | .156 | 7.379 | \*\*\* | v17\_2 |
| e20 |  |  | 1.781 | .199 | 8.959 | \*\*\* | v18\_2 |

##### Squared Multiple Correlations: (other - Measurement weights)

|  |  |  | Estimate |
| --- | --- | --- | --- |
| AI自我效能 |  |  | .157 |
| 防御性动机 |  |  | .221 |
| 使用行为 |  |  | .332 |
| AB5 |  |  | .491 |
| AB3 |  |  | .644 |
| AB2 |  |  | .548 |
| AB1 |  |  | .564 |
| SE5 |  |  | .610 |
| SE4 |  |  | .610 |
| SE3 |  |  | .458 |
| SE2 |  |  | .479 |
| SE1 |  |  | .380 |
| DM5 |  |  | .457 |
| DM3 |  |  | .601 |
| DM2 |  |  | .524 |
| DM1 |  |  | .491 |
| AA5 |  |  | .600 |
| AA4 |  |  | .558 |
| AA3 |  |  | .587 |
| AA2 |  |  | .629 |
| AA1 |  |  | .666 |

##### User-defined estimands: (other - Measurement weights)

|  |  |  |  |
| --- | --- | --- | --- |
| diff1 |  |  | .231 |
| diff2 |  |  | .080 |
| diff3 |  |  | .260 |
| diff4 |  |  | .039 |
| diff5 |  |  | -.095 |

##### Modification Indices (other - Measurement weights)

##### Covariances: (other - Measurement weights)

|  |  |  | M.I. | Par Change |
| --- | --- | --- | --- | --- |
| e20 | <--> | e23 | 5.517 | -.290 |
| e13 | <--> | AI威胁感知 | 4.519 | -.310 |
| e9 | <--> | e18 | 4.392 | .221 |
| e8 | <--> | e22 | 6.381 | .297 |
| e6 | <--> | e11 | 5.402 | -.319 |
| e5 | <--> | e14 | 12.093 | -.366 |
| e4 | <--> | e5 | 4.448 | .229 |
| e3 | <--> | e17 | 4.171 | .227 |
| e3 | <--> | e4 | 4.025 | -.218 |
| e2 | <--> | e14 | 6.372 | .256 |
| e2 | <--> | e13 | 4.061 | -.225 |
| e1 | <--> | e3 | 4.834 | .215 |

##### Variances: (other - Measurement weights)

|  |  |  | M.I. | Par Change |
| --- | --- | --- | --- | --- |

##### Regression Weights: (other - Measurement weights)

|  |  |  | M.I. | Par Change |
| --- | --- | --- | --- | --- |
| AB5 | <--- | DM5 | 4.218 | -.106 |
| SE4 | <--- | AA2 | 5.655 | .110 |
| SE3 | <--- | AI威胁感知 | 4.519 | -.138 |
| SE3 | <--- | AA3 | 4.494 | -.108 |
| SE3 | <--- | AA2 | 7.298 | -.138 |
| SE1 | <--- | DM1 | 6.254 | -.132 |
| DM2 | <--- | AB2 | 4.214 | .105 |
| DM2 | <--- | AB1 | 4.554 | .109 |
| DM1 | <--- | SE1 | 4.990 | -.119 |
| AA5 | <--- | SE4 | 6.441 | -.119 |
| AA2 | <--- | SE4 | 4.588 | .097 |

##### Bootstrap (other - Measurement weights)

##### Bootstrap standard errors (other - Measurement weights)

##### Scalar Estimates (other - Measurement weights)

##### Regression Weights: (other - Measurement weights)

| Parameter | | | SE | SE-SE | Mean | Bias | SE-Bias |
| --- | --- | --- | --- | --- | --- | --- | --- |
| AI自我效能 | <--- | AI威胁感知 | .058 | .001 | .290 | .003 | .002 |
| 防御性动机 | <--- | AI威胁感知 | .072 | .002 | .408 | .002 | .002 |
| 防御性动机 | <--- | AI自我效能 | .099 | .002 | -.017 | .000 | .003 |
| 使用行为 | <--- | 防御性动机 | .078 | .002 | .450 | .006 | .002 |
| 使用行为 | <--- | AI自我效能 | .090 | .002 | .406 | .000 | .003 |
| AA1 | <--- | AI威胁感知 | .000 | .000 | 1.000 | .000 | .000 |
| AA2 | <--- | AI威胁感知 | .048 | .001 | .952 | -.001 | .002 |
| AA3 | <--- | AI威胁感知 | .046 | .001 | .923 | .002 | .001 |
| AA4 | <--- | AI威胁感知 | .051 | .001 | .919 | .000 | .002 |
| AA5 | <--- | AI威胁感知 | .052 | .001 | .942 | .002 | .002 |
| DM1 | <--- | 防御性动机 | .000 | .000 | 1.000 | .000 | .000 |
| DM2 | <--- | 防御性动机 | .073 | .002 | 1.032 | .005 | .002 |
| DM3 | <--- | 防御性动机 | .075 | .002 | 1.056 | .007 | .002 |
| DM5 | <--- | 防御性动机 | .079 | .002 | .980 | .004 | .002 |
| SE1 | <--- | AI自我效能 | .000 | .000 | 1.000 | .000 | .000 |
| SE2 | <--- | AI自我效能 | .092 | .002 | 1.149 | -.005 | .003 |
| SE3 | <--- | AI自我效能 | .088 | .002 | 1.099 | .001 | .003 |
| SE4 | <--- | AI自我效能 | .096 | .002 | 1.267 | -.003 | .003 |
| SE5 | <--- | AI自我效能 | .094 | .002 | 1.220 | .000 | .003 |
| AB1 | <--- | 使用行为 | .000 | .000 | 1.000 | .000 | .000 |
| AB2 | <--- | 使用行为 | .060 | .001 | .982 | .002 | .002 |
| AB3 | <--- | 使用行为 | .063 | .001 | 1.071 | .002 | .002 |
| AB5 | <--- | 使用行为 | .065 | .001 | .972 | .002 | .002 |

##### Standardized Regression Weights: (other - Measurement weights)

| Parameter | | | SE | SE-SE | Mean | Bias | SE-Bias |
| --- | --- | --- | --- | --- | --- | --- | --- |
| AI自我效能 | <--- | AI威胁感知 | .067 | .002 | .396 | .000 | .002 |
| 防御性动机 | <--- | AI威胁感知 | .075 | .002 | .480 | .004 | .002 |
| 防御性动机 | <--- | AI自我效能 | .084 | .002 | -.014 | .000 | .003 |
| 使用行为 | <--- | 防御性动机 | .066 | .001 | .422 | .002 | .002 |
| 使用行为 | <--- | AI自我效能 | .068 | .002 | .327 | -.001 | .002 |
| AA1 | <--- | AI威胁感知 | .026 | .001 | .816 | .000 | .001 |
| AA2 | <--- | AI威胁感知 | .028 | .001 | .791 | -.002 | .001 |
| AA3 | <--- | AI威胁感知 | .028 | .001 | .767 | .001 | .001 |
| AA4 | <--- | AI威胁感知 | .032 | .001 | .746 | -.001 | .001 |
| AA5 | <--- | AI威胁感知 | .033 | .001 | .775 | .001 | .001 |
| DM1 | <--- | 防御性动机 | .037 | .001 | .698 | -.003 | .001 |
| DM2 | <--- | 防御性动机 | .034 | .001 | .724 | .000 | .001 |
| DM3 | <--- | 防御性动机 | .032 | .001 | .776 | .001 | .001 |
| DM5 | <--- | 防御性动机 | .039 | .001 | .675 | -.001 | .001 |
| SE1 | <--- | AI自我效能 | .038 | .001 | .619 | .003 | .001 |
| SE2 | <--- | AI自我效能 | .036 | .001 | .691 | -.001 | .001 |
| SE3 | <--- | AI自我效能 | .037 | .001 | .679 | .002 | .001 |
| SE4 | <--- | AI自我效能 | .031 | .001 | .780 | -.001 | .001 |
| SE5 | <--- | AI自我效能 | .030 | .001 | .782 | .001 | .001 |
| AB1 | <--- | 使用行为 | .031 | .001 | .753 | .002 | .001 |
| AB2 | <--- | 使用行为 | .035 | .001 | .741 | .001 | .001 |
| AB3 | <--- | 使用行为 | .031 | .001 | .803 | .001 | .001 |
| AB5 | <--- | 使用行为 | .033 | .001 | .702 | .001 | .001 |

##### Variances: (other - Measurement weights)

| Parameter | | | SE | SE-SE | Mean | Bias | SE-Bias |
| --- | --- | --- | --- | --- | --- | --- | --- |
| AI威胁感知 |  |  | .233 | .005 | 2.257 | .001 | .007 |
| e21 |  |  | .149 | .003 | 1.005 | .005 | .005 |
| e23 |  |  | .184 | .004 | 1.245 | -.033 | .006 |
| e22 |  |  | .168 | .004 | 1.206 | -.014 | .005 |
| e1 |  |  | .134 | .003 | 1.122 | -.010 | .004 |
| e2 |  |  | .145 | .003 | 1.209 | .002 | .005 |
| e3 |  |  | .134 | .003 | 1.337 | -.012 | .004 |
| e4 |  |  | .169 | .004 | 1.505 | -.007 | .005 |
| e5 |  |  | .178 | .004 | 1.318 | -.013 | .006 |
| e6 |  |  | .184 | .004 | 1.699 | -.001 | .006 |
| e8 |  |  | .189 | .004 | 1.557 | -.012 | .006 |
| e9 |  |  | .136 | .003 | 1.184 | -.016 | .004 |
| e10 |  |  | .190 | .004 | 1.843 | -.013 | .006 |
| e11 |  |  | .168 | .004 | 1.912 | -.020 | .005 |
| e12 |  |  | .177 | .004 | 1.702 | -.015 | .006 |
| e13 |  |  | .176 | .004 | 1.668 | -.022 | .006 |
| e14 |  |  | .143 | .003 | 1.221 | -.006 | .005 |
| e15 |  |  | .143 | .003 | 1.116 | -.012 | .005 |
| e16 |  |  | .149 | .003 | 1.393 | -.021 | .005 |
| e17 |  |  | .168 | .004 | 1.439 | -.010 | .005 |
| e18 |  |  | .165 | .004 | 1.145 | -.009 | .005 |
| e20 |  |  | .175 | .004 | 1.766 | -.015 | .006 |

##### Squared Multiple Correlations: (other - Measurement weights)

| Parameter | | | SE | SE-SE | Mean | Bias | SE-Bias |
| --- | --- | --- | --- | --- | --- | --- | --- |
| AI自我效能 |  |  | .053 | .001 | .161 | .005 | .002 |
| 防御性动机 |  |  | .061 | .001 | .235 | .014 | .002 |
| 使用行为 |  |  | .063 | .001 | .341 | .009 | .002 |
| AB5 |  |  | .046 | .001 | .494 | .002 | .001 |
| AB3 |  |  | .049 | .001 | .646 | .002 | .002 |
| AB2 |  |  | .052 | .001 | .550 | .002 | .002 |
| AB1 |  |  | .047 | .001 | .567 | .004 | .001 |
| SE5 |  |  | .047 | .001 | .612 | .002 | .001 |
| SE4 |  |  | .048 | .001 | .609 | -.001 | .002 |
| SE3 |  |  | .050 | .001 | .463 | .004 | .002 |
| SE2 |  |  | .050 | .001 | .479 | .000 | .002 |
| SE1 |  |  | .047 | .001 | .385 | .005 | .001 |
| DM5 |  |  | .053 | .001 | .457 | .000 | .002 |
| DM3 |  |  | .050 | .001 | .603 | .002 | .002 |
| DM2 |  |  | .050 | .001 | .525 | .001 | .002 |
| DM1 |  |  | .052 | .001 | .489 | -.002 | .002 |
| AA5 |  |  | .050 | .001 | .602 | .002 | .002 |
| AA4 |  |  | .048 | .001 | .558 | .000 | .002 |
| AA3 |  |  | .043 | .001 | .588 | .002 | .001 |
| AA2 |  |  | .045 | .001 | .627 | -.002 | .001 |
| AA1 |  |  | .042 | .001 | .667 | .001 | .001 |

##### User-defined estimands: (other - Measurement weights)

| Parameter | | | SE | SE-SE | Mean | Bias | SE-Bias |
| --- | --- | --- | --- | --- | --- | --- | --- |
| diff1 |  |  | .082 | .002 | .232 | .001 | .003 |
| diff2 |  |  | .126 | .003 | .079 | -.002 | .004 |
| diff3 |  |  | .127 | .003 | .256 | -.004 | .004 |
| diff4 |  |  | .112 | .003 | .034 | -.005 | .004 |
| diff5 |  |  | .139 | .003 | -.092 | .003 | .004 |

##### Bootstrap Confidence (other - Measurement weights)

##### Percentile method (other - Measurement weights)

##### 95% confidence intervals (percentile method)

##### Scalar Estimates (other - Measurement weights)

##### Regression Weights: (other - Measurement weights)

| Parameter | | | Estimate | Lower | Upper | P |
| --- | --- | --- | --- | --- | --- | --- |
| AI自我效能 | <--- | AI威胁感知 | .287 | .178 | .402 | .002 |
| 防御性动机 | <--- | AI威胁感知 | .405 | .272 | .557 | .002 |
| 防御性动机 | <--- | AI自我效能 | -.017 | -.226 | .170 | .878 |
| 使用行为 | <--- | 防御性动机 | .443 | .302 | .610 | .002 |
| 使用行为 | <--- | AI自我效能 | .407 | .235 | .582 | .002 |
| AA1 | <--- | AI威胁感知 | 1.000 | 1.000 | 1.000 | ... |
| AA2 | <--- | AI威胁感知 | .953 | .863 | 1.048 | .002 |
| AA3 | <--- | AI威胁感知 | .921 | .835 | 1.017 | .002 |
| AA4 | <--- | AI威胁感知 | .919 | .829 | 1.026 | .002 |
| AA5 | <--- | AI威胁感知 | .940 | .842 | 1.052 | .002 |
| DM1 | <--- | 防御性动机 | 1.000 | 1.000 | 1.000 | ... |
| DM2 | <--- | 防御性动机 | 1.027 | .890 | 1.172 | .002 |
| DM3 | <--- | 防御性动机 | 1.049 | .912 | 1.209 | .002 |
| DM5 | <--- | 防御性动机 | .976 | .837 | 1.137 | .002 |
| SE1 | <--- | AI自我效能 | 1.000 | 1.000 | 1.000 | ... |
| SE2 | <--- | AI自我效能 | 1.153 | .974 | 1.342 | .002 |
| SE3 | <--- | AI自我效能 | 1.098 | .943 | 1.280 | .002 |
| SE4 | <--- | AI自我效能 | 1.271 | 1.101 | 1.474 | .002 |
| SE5 | <--- | AI自我效能 | 1.220 | 1.055 | 1.432 | .002 |
| AB1 | <--- | 使用行为 | 1.000 | 1.000 | 1.000 | ... |
| AB2 | <--- | 使用行为 | .980 | .867 | 1.109 | .002 |
| AB3 | <--- | 使用行为 | 1.068 | .956 | 1.202 | .002 |
| AB5 | <--- | 使用行为 | .970 | .854 | 1.117 | .002 |

##### Standardized Regression Weights: (other - Measurement weights)

| Parameter | | | Estimate | Lower | Upper | P |
| --- | --- | --- | --- | --- | --- | --- |
| AI自我效能 | <--- | AI威胁感知 | .396 | .255 | .521 | .002 |
| 防御性动机 | <--- | AI威胁感知 | .476 | .330 | .621 | .002 |
| 防御性动机 | <--- | AI自我效能 | -.014 | -.188 | .145 | .878 |
| 使用行为 | <--- | 防御性动机 | .420 | .297 | .551 | .002 |
| 使用行为 | <--- | AI自我效能 | .328 | .194 | .462 | .002 |
| AA1 | <--- | AI威胁感知 | .816 | .762 | .864 | .002 |
| AA2 | <--- | AI威胁感知 | .793 | .734 | .844 | .002 |
| AA3 | <--- | AI威胁感知 | .766 | .708 | .819 | .002 |
| AA4 | <--- | AI威胁感知 | .747 | .681 | .806 | .002 |
| AA5 | <--- | AI威胁感知 | .774 | .711 | .833 | .002 |
| DM1 | <--- | 防御性动机 | .701 | .625 | .769 | .002 |
| DM2 | <--- | 防御性动机 | .724 | .654 | .789 | .002 |
| DM3 | <--- | 防御性动机 | .775 | .708 | .838 | .002 |
| DM5 | <--- | 防御性动机 | .676 | .599 | .750 | .002 |
| SE1 | <--- | AI自我效能 | .617 | .543 | .693 | .002 |
| SE2 | <--- | AI自我效能 | .692 | .620 | .760 | .002 |
| SE3 | <--- | AI自我效能 | .677 | .607 | .745 | .002 |
| SE4 | <--- | AI自我效能 | .781 | .717 | .837 | .002 |
| SE5 | <--- | AI自我效能 | .781 | .720 | .837 | .002 |
| AB1 | <--- | 使用行为 | .751 | .691 | .812 | .002 |
| AB2 | <--- | 使用行为 | .740 | .671 | .806 | .002 |
| AB3 | <--- | 使用行为 | .802 | .739 | .860 | .002 |
| AB5 | <--- | 使用行为 | .701 | .639 | .764 | .002 |

##### Variances: (other - Measurement weights)

| Parameter | | | Estimate | Lower | Upper | P |
| --- | --- | --- | --- | --- | --- | --- |
| AI威胁感知 |  |  | 2.256 | 1.790 | 2.720 | .002 |
| e21 |  |  | 1.001 | .724 | 1.313 | .002 |
| e23 |  |  | 1.277 | .899 | 1.620 | .002 |
| e22 |  |  | 1.220 | .893 | 1.559 | .002 |
| e1 |  |  | 1.132 | .871 | 1.380 | .002 |
| e2 |  |  | 1.207 | .942 | 1.516 | .002 |
| e3 |  |  | 1.350 | 1.093 | 1.616 | .002 |
| e4 |  |  | 1.512 | 1.188 | 1.833 | .002 |
| e5 |  |  | 1.331 | .989 | 1.692 | .002 |
| e6 |  |  | 1.700 | 1.358 | 2.068 | .002 |
| e8 |  |  | 1.570 | 1.200 | 1.941 | .002 |
| e9 |  |  | 1.200 | .907 | 1.451 | .002 |
| e10 |  |  | 1.856 | 1.476 | 2.219 | .002 |
| e11 |  |  | 1.932 | 1.566 | 2.250 | .002 |
| e12 |  |  | 1.717 | 1.359 | 2.045 | .002 |
| e13 |  |  | 1.690 | 1.326 | 2.009 | .002 |
| e14 |  |  | 1.227 | .938 | 1.505 | .002 |
| e15 |  |  | 1.128 | .856 | 1.416 | .002 |
| e16 |  |  | 1.414 | 1.107 | 1.697 | .002 |
| e17 |  |  | 1.449 | 1.120 | 1.772 | .002 |
| e18 |  |  | 1.154 | .826 | 1.474 | .002 |
| e20 |  |  | 1.781 | 1.428 | 2.109 | .002 |

##### Squared Multiple Correlations: (other - Measurement weights)

| Parameter | | | Estimate | Lower | Upper | P |
| --- | --- | --- | --- | --- | --- | --- |
| AI自我效能 |  |  | .157 | .065 | .271 | .002 |
| 防御性动机 |  |  | .221 | .124 | .361 | .002 |
| 使用行为 |  |  | .332 | .225 | .466 | .002 |
| AB5 |  |  | .491 | .408 | .584 | .002 |
| AB3 |  |  | .644 | .546 | .740 | .002 |
| AB2 |  |  | .548 | .450 | .650 | .002 |
| AB1 |  |  | .564 | .477 | .659 | .002 |
| SE5 |  |  | .610 | .518 | .701 | .002 |
| SE4 |  |  | .610 | .514 | .701 | .002 |
| SE3 |  |  | .458 | .368 | .555 | .002 |
| SE2 |  |  | .479 | .384 | .578 | .002 |
| SE1 |  |  | .380 | .295 | .480 | .002 |
| DM5 |  |  | .457 | .359 | .563 | .002 |
| DM3 |  |  | .601 | .502 | .702 | .002 |
| DM2 |  |  | .524 | .428 | .623 | .002 |
| DM1 |  |  | .491 | .391 | .591 | .002 |
| AA5 |  |  | .600 | .506 | .694 | .002 |
| AA4 |  |  | .558 | .463 | .650 | .002 |
| AA3 |  |  | .587 | .502 | .671 | .002 |
| AA2 |  |  | .629 | .539 | .712 | .002 |
| AA1 |  |  | .666 | .581 | .747 | .002 |

##### User-defined estimands: (other - Measurement weights)

| Parameter | | | Estimate | Lower | Upper | P |
| --- | --- | --- | --- | --- | --- | --- |
| diff1 |  |  | .231 | .065 | .378 | .005 |
| diff2 |  |  | .080 | -.175 | .316 | .534 |
| diff3 |  |  | .260 | .014 | .524 | .047 |
| diff4 |  |  | .039 | -.171 | .261 | .757 |
| diff5 |  |  | -.095 | -.349 | .192 | .517 |

##### Bias-corrected percentile method (other - Measurement weights)

##### 95% confidence intervals (bias-corrected percentile method)

##### Scalar Estimates (other - Measurement weights)

##### Regression Weights: (other - Measurement weights)

| Parameter | | | Estimate | Lower | Upper | P |
| --- | --- | --- | --- | --- | --- | --- |
| AI自我效能 | <--- | AI威胁感知 | .287 | .173 | .399 | .002 |
| 防御性动机 | <--- | AI威胁感知 | .405 | .266 | .545 | .003 |
| 防御性动机 | <--- | AI自我效能 | -.017 | -.227 | .170 | .874 |
| 使用行为 | <--- | 防御性动机 | .443 | .294 | .596 | .003 |
| 使用行为 | <--- | AI自我效能 | .407 | .235 | .581 | .002 |
| AA1 | <--- | AI威胁感知 | 1.000 | 1.000 | 1.000 | ... |
| AA2 | <--- | AI威胁感知 | .953 | .865 | 1.053 | .002 |
| AA3 | <--- | AI威胁感知 | .921 | .829 | 1.014 | .003 |
| AA4 | <--- | AI威胁感知 | .919 | .829 | 1.028 | .002 |
| AA5 | <--- | AI威胁感知 | .940 | .842 | 1.051 | .002 |
| DM1 | <--- | 防御性动机 | 1.000 | 1.000 | 1.000 | ... |
| DM2 | <--- | 防御性动机 | 1.027 | .886 | 1.167 | .003 |
| DM3 | <--- | 防御性动机 | 1.049 | .904 | 1.200 | .003 |
| DM5 | <--- | 防御性动机 | .976 | .836 | 1.136 | .002 |
| SE1 | <--- | AI自我效能 | 1.000 | 1.000 | 1.000 | ... |
| SE2 | <--- | AI自我效能 | 1.153 | .990 | 1.357 | .001 |
| SE3 | <--- | AI自我效能 | 1.098 | .948 | 1.296 | .001 |
| SE4 | <--- | AI自我效能 | 1.271 | 1.113 | 1.484 | .001 |
| SE5 | <--- | AI自我效能 | 1.220 | 1.061 | 1.454 | .001 |
| AB1 | <--- | 使用行为 | 1.000 | 1.000 | 1.000 | ... |
| AB2 | <--- | 使用行为 | .980 | .873 | 1.117 | .001 |
| AB3 | <--- | 使用行为 | 1.068 | .957 | 1.205 | .002 |
| AB5 | <--- | 使用行为 | .970 | .857 | 1.127 | .001 |

##### Standardized Regression Weights: (other - Measurement weights)

| Parameter | | | Estimate | Lower | Upper | P |
| --- | --- | --- | --- | --- | --- | --- |
| AI自我效能 | <--- | AI威胁感知 | .396 | .254 | .520 | .002 |
| 防御性动机 | <--- | AI威胁感知 | .476 | .308 | .609 | .004 |
| 防御性动机 | <--- | AI自我效能 | -.014 | -.188 | .145 | .874 |
| 使用行为 | <--- | 防御性动机 | .420 | .278 | .542 | .003 |
| 使用行为 | <--- | AI自我效能 | .328 | .186 | .459 | .002 |
| AA1 | <--- | AI威胁感知 | .816 | .759 | .861 | .003 |
| AA2 | <--- | AI威胁感知 | .793 | .737 | .846 | .002 |
| AA3 | <--- | AI威胁感知 | .766 | .707 | .819 | .002 |
| AA4 | <--- | AI威胁感知 | .747 | .680 | .805 | .002 |
| AA5 | <--- | AI威胁感知 | .774 | .703 | .828 | .004 |
| DM1 | <--- | 防御性动机 | .701 | .630 | .774 | .001 |
| DM2 | <--- | 防御性动机 | .724 | .652 | .788 | .002 |
| DM3 | <--- | 防御性动机 | .775 | .703 | .834 | .003 |
| DM5 | <--- | 防御性动机 | .676 | .598 | .750 | .002 |
| SE1 | <--- | AI自我效能 | .617 | .538 | .687 | .004 |
| SE2 | <--- | AI自我效能 | .692 | .622 | .763 | .002 |
| SE3 | <--- | AI自我效能 | .677 | .601 | .740 | .004 |
| SE4 | <--- | AI自我效能 | .781 | .717 | .838 | .002 |
| SE5 | <--- | AI自我效能 | .781 | .716 | .833 | .003 |
| AB1 | <--- | 使用行为 | .751 | .680 | .804 | .004 |
| AB2 | <--- | 使用行为 | .740 | .669 | .805 | .002 |
| AB3 | <--- | 使用行为 | .802 | .735 | .857 | .003 |
| AB5 | <--- | 使用行为 | .701 | .634 | .762 | .003 |

##### Variances: (other - Measurement weights)

| Parameter | | | Estimate | Lower | Upper | P |
| --- | --- | --- | --- | --- | --- | --- |
| AI威胁感知 |  |  | 2.256 | 1.790 | 2.720 | .002 |
| e21 |  |  | 1.001 | .726 | 1.316 | .002 |
| e23 |  |  | 1.277 | .957 | 1.731 | .000 |
| e22 |  |  | 1.220 | .940 | 1.608 | .001 |
| e1 |  |  | 1.132 | .894 | 1.404 | .001 |
| e2 |  |  | 1.207 | .940 | 1.511 | .002 |
| e3 |  |  | 1.350 | 1.111 | 1.637 | .001 |
| e4 |  |  | 1.512 | 1.221 | 1.882 | .001 |
| e5 |  |  | 1.331 | 1.024 | 1.720 | .001 |
| e6 |  |  | 1.700 | 1.370 | 2.085 | .001 |
| e8 |  |  | 1.570 | 1.229 | 1.986 | .001 |
| e9 |  |  | 1.200 | .950 | 1.488 | .001 |
| e10 |  |  | 1.856 | 1.498 | 2.245 | .001 |
| e11 |  |  | 1.932 | 1.600 | 2.295 | .001 |
| e12 |  |  | 1.717 | 1.383 | 2.076 | .001 |
| e13 |  |  | 1.690 | 1.369 | 2.087 | .001 |
| e14 |  |  | 1.227 | .961 | 1.527 | .001 |
| e15 |  |  | 1.128 | .885 | 1.477 | .001 |
| e16 |  |  | 1.414 | 1.152 | 1.768 | .001 |
| e17 |  |  | 1.449 | 1.138 | 1.792 | .001 |
| e18 |  |  | 1.154 | .851 | 1.499 | .001 |
| e20 |  |  | 1.781 | 1.449 | 2.120 | .001 |

##### Squared Multiple Correlations: (other - Measurement weights)

| Parameter | | | Estimate | Lower | Upper | P |
| --- | --- | --- | --- | --- | --- | --- |
| AI自我效能 |  |  | .157 | .064 | .270 | .002 |
| 防御性动机 |  |  | .221 | .108 | .340 | .006 |
| 使用行为 |  |  | .332 | .208 | .453 | .005 |
| AB5 |  |  | .491 | .402 | .581 | .003 |
| AB3 |  |  | .644 | .540 | .735 | .003 |
| AB2 |  |  | .548 | .447 | .648 | .002 |
| AB1 |  |  | .564 | .462 | .647 | .004 |
| SE5 |  |  | .610 | .513 | .694 | .003 |
| SE4 |  |  | .610 | .514 | .701 | .002 |
| SE3 |  |  | .458 | .361 | .548 | .004 |
| SE2 |  |  | .479 | .387 | .582 | .002 |
| SE1 |  |  | .380 | .290 | .471 | .004 |
| DM5 |  |  | .457 | .358 | .562 | .002 |
| DM3 |  |  | .601 | .494 | .695 | .003 |
| DM2 |  |  | .524 | .425 | .620 | .002 |
| DM1 |  |  | .491 | .397 | .599 | .001 |
| AA5 |  |  | .600 | .494 | .686 | .004 |
| AA4 |  |  | .558 | .462 | .648 | .002 |
| AA3 |  |  | .587 | .500 | .670 | .002 |
| AA2 |  |  | .629 | .543 | .715 | .002 |
| AA1 |  |  | .666 | .576 | .742 | .003 |

##### User-defined estimands: (other - Measurement weights)

| Parameter | | | Estimate | Lower | Upper | P |
| --- | --- | --- | --- | --- | --- | --- |
| diff1 |  |  | .231 | .062 | .376 | .006 |
| diff2 |  |  | .080 | -.180 | .314 | .554 |
| diff3 |  |  | .260 | .026 | .546 | .037 |
| diff4 |  |  | .039 | -.158 | .274 | .642 |
| diff5 |  |  | -.095 | -.343 | .198 | .543 |

##### Minimization History (Measurement weights)

| Iteration |  | Negative eigenvalues | Condition # | Smallest eigenvalue | Diameter | F | NTries | Ratio |
| --- | --- | --- | --- | --- | --- | --- | --- | --- |
| 0 | e | 8 |  | -.925 | 9999.000 | 3457.824 | 0 | 9999.000 |
| 1 | e | 2 |  | -.028 | 3.295 | 736.563 | 19 | .340 |
| 2 | e | 0 | 62.687 |  | 1.870 | 356.471 | 6 | .683 |
| 3 | e | 0 | 84.983 |  | 1.348 | 345.762 | 1 | .125 |
| 4 | e | 0 | 34.805 |  | .466 | 277.317 | 1 | 1.141 |
| 5 | e | 0 | 27.468 |  | .176 | 267.384 | 1 | 1.208 |
| 6 | e | 0 | 24.661 |  | .169 | 265.216 | 1 | 1.161 |
| 7 | e | 0 | 25.447 |  | .047 | 264.989 | 1 | 1.082 |
| 8 | e | 0 | 25.424 |  | .008 | 264.985 | 1 | 1.013 |
| 9 | e | 0 | 25.424 |  | .000 | 264.985 | 1 | 1.000 |

##### Bootstrap (Measurement weights)

##### Summary of Bootstrap Iterations (Measurement weights)

##### (Measurement weights)

| Iterations | Method 0 | Method 1 | Method 2 |
| --- | --- | --- | --- |
| 1 | 0 | 0 | 0 |
| 2 | 0 | 0 | 0 |
| 3 | 0 | 0 | 0 |
| 4 | 0 | 0 | 0 |
| 5 | 0 | 1 | 0 |
| 6 | 0 | 102 | 0 |
| 7 | 0 | 398 | 0 |
| 8 | 0 | 290 | 0 |
| 9 | 0 | 151 | 0 |
| 10 | 0 | 41 | 0 |
| 11 | 0 | 11 | 0 |
| 12 | 0 | 4 | 0 |
| 13 | 0 | 1 | 0 |
| 14 | 0 | 0 | 0 |
| 15 | 0 | 1 | 0 |
| 16 | 0 | 0 | 0 |
| 17 | 0 | 0 | 0 |
| 18 | 0 | 0 | 0 |
| 19 | 0 | 0 | 0 |
| Total | 0 | 1000 | 0 |

0 bootstrap samples were unused because of a singular covariance matrix.

0 bootstrap samples were unused because a solution was not found.

1000 usable bootstrap samples were obtained.

##### Bootstrap Distributions (Measurement weights)

##### ML discrepancy (implied vs sample) (Measurement weights)

|  |  |  |
| --- | --- | --- |
|  |  | \|-------------------- |
|  | 433.116 | \|\* |
|  | 454.104 | \|\*\* |
|  | 475.092 | \|\*\*\*\*\* |
|  | 496.080 | \|\*\*\*\*\*\*\*\*\* |
|  | 517.069 | \|\*\*\*\*\*\*\*\*\*\*\*\* |
|  | 538.057 | \|\*\*\*\*\*\*\*\*\*\*\*\*\*\*\*\*\* |
|  | 559.045 | \|\*\*\*\*\*\*\*\*\*\*\*\*\*\*\*\*\*\*\* |
| N = 1000 | 580.033 | \|\*\*\*\*\*\*\*\*\*\*\*\*\*\*\*\* |
| Mean = 558.781 | 601.021 | \|\*\*\*\*\*\*\*\*\*\* |
| S. e. = 1.502 | 622.010 | \|\*\*\*\*\*\*\*\*\* |
|  | 642.998 | \|\*\*\*\* |
|  | 663.986 | \|\*\* |
|  | 684.974 | \|\*\* |
|  | 705.962 | \| |
|  | 726.951 | \|\* |
|  |  | \|-------------------- |

##### ML discrepancy (implied vs pop) (Measurement weights)

|  |  |  |
| --- | --- | --- |
|  |  | \|-------------------- |
|  | 318.082 | \|\* |
|  | 325.389 | \|\* |
|  | 332.696 | \|\*\*\*\* |
|  | 340.004 | \|\*\*\*\*\*\* |
|  | 347.311 | \|\*\*\*\*\*\*\*\*\*\*\*\*\*\*\*\* |
|  | 354.618 | \|\*\*\*\*\*\*\*\*\*\*\*\*\*\*\*\*\*\* |
|  | 361.926 | \|\*\*\*\*\*\*\*\*\*\*\*\*\*\*\*\*\*\*\* |
| N = 1000 | 369.233 | \|\*\*\*\*\*\*\*\*\*\*\*\*\*\*\*\* |
| Mean = 362.260 | 376.540 | \|\*\*\*\*\*\*\*\*\*\* |
| S. e. = .511 | 383.847 | \|\*\*\*\*\*\*\* |
|  | 391.155 | \|\*\*\*\* |
|  | 398.462 | \|\*\* |
|  | 405.769 | \|\* |
|  | 413.077 | \|\* |
|  | 420.384 | \|\* |
|  |  | \|-------------------- |

##### K-L overoptimism (unstabilized) (Measurement weights)

|  |  |  |
| --- | --- | --- |
|  |  | \|-------------------- |
|  | -155.218 | \|\* |
|  | -101.640 | \|\* |
|  | -48.062 | \|\*\*\* |
|  | 5.516 | \|\*\*\*\*\*\* |
|  | 59.095 | \|\*\*\*\*\*\*\*\*\* |
|  | 112.673 | \|\*\*\*\*\*\*\*\*\*\*\*\*\*\*\* |
|  | 166.251 | \|\*\*\*\*\*\*\*\*\*\*\*\*\*\*\*\*\*\* |
| N = 1000 | 219.829 | \|\*\*\*\*\*\*\*\*\*\*\*\*\*\*\*\*\*\*\*\* |
| Mean = 196.051 | 273.408 | \|\*\*\*\*\*\*\*\*\*\*\*\*\* |
| S. e. = 3.658 | 326.986 | \|\*\*\*\*\*\*\*\*\*\*\* |
|  | 380.564 | \|\*\*\*\*\*\* |
|  | 434.142 | \|\*\*\* |
|  | 487.720 | \|\* |
|  | 541.299 | \|\* |
|  | 594.877 | \|\* |
|  |  | \|-------------------- |

##### K-L overoptimism (stabilized) (Measurement weights)

|  |  |  |
| --- | --- | --- |
|  |  | \|-------------------- |
|  | 57.120 | \|\* |
|  | 78.324 | \|\* |
|  | 99.528 | \|\*\* |
|  | 120.733 | \|\*\*\*\* |
|  | 141.937 | \|\*\*\*\*\*\*\*\*\*\* |
|  | 163.141 | \|\*\*\*\*\*\*\*\*\*\*\*\*\*\* |
|  | 184.346 | \|\*\*\*\*\*\*\*\*\*\*\*\*\*\*\*\*\*\*\* |
| N = 1000 | 205.550 | \|\*\*\*\*\*\*\*\*\*\*\*\*\*\*\*\* |
| Mean = 190.760 | 226.754 | \|\*\*\*\*\*\*\*\*\*\*\*\*\* |
| S. e. = 1.283 | 247.959 | \|\*\*\*\*\* |
|  | 269.163 | \|\*\*\* |
|  | 290.367 | \|\*\* |
|  | 311.572 | \|\* |
|  | 332.776 | \|\* |
|  | 353.980 | \|\* |
|  |  | \|-------------------- |

##### Structural weights (Structural weights)

##### Notes for Model (Structural weights)

##### Computation of degrees of freedom (Structural weights)

|  |  |
| --- | --- |
| Number of distinct sample moments: | 342 |
| Number of distinct parameters to be estimated: | 63 |
| Degrees of freedom (342 - 63): | 279 |

##### Result (Structural weights)

Minimum was achieved

Chi-square = 276.671

Degrees of freedom = 279

Probability level = .528

##### east (east - Structural weights)

##### Estimates (east - Structural weights)

##### Scalar Estimates (east - Structural weights)

##### Maximum Likelihood Estimates

##### Regression Weights: (east - Structural weights)

|  |  |  | Estimate | S.E. | C.R. | P | Label |
| --- | --- | --- | --- | --- | --- | --- | --- |
| AI自我效能 | <--- | AI威胁感知 | .372 | .049 | 7.568 | \*\*\* | H1a |
| 防御性动机 | <--- | AI威胁感知 | .399 | .062 | 6.474 | \*\*\* | H2a |
| 防御性动机 | <--- | AI自我效能 | .074 | .074 | .999 | .318 | H5a |
| 使用行为 | <--- | 防御性动机 | .402 | .064 | 6.252 | \*\*\* | H4a |
| 使用行为 | <--- | AI自我效能 | .466 | .074 | 6.313 | \*\*\* | H3a |
| AA1 | <--- | AI威胁感知 | 1.000 |  |  |  |  |
| AA2 | <--- | AI威胁感知 | .953 | .057 | 16.829 | \*\*\* | a1\_1 |
| AA3 | <--- | AI威胁感知 | .921 | .058 | 15.919 | \*\*\* | a2\_1 |
| AA4 | <--- | AI威胁感知 | .919 | .058 | 15.976 | \*\*\* | a3\_1 |
| AA5 | <--- | AI威胁感知 | .939 | .056 | 16.729 | \*\*\* | a4\_1 |
| DM1 | <--- | 防御性动机 | 1.000 |  |  |  |  |
| DM2 | <--- | 防御性动机 | 1.026 | .082 | 12.532 | \*\*\* | a5\_1 |
| DM3 | <--- | 防御性动机 | 1.050 | .082 | 12.782 | \*\*\* | a6\_1 |
| DM5 | <--- | 防御性动机 | .978 | .081 | 12.070 | \*\*\* | a7\_1 |
| SE1 | <--- | AI自我效能 | 1.000 |  |  |  |  |
| SE2 | <--- | AI自我效能 | 1.163 | .101 | 11.499 | \*\*\* | a8\_1 |
| SE3 | <--- | AI自我效能 | 1.099 | .097 | 11.301 | \*\*\* | a9\_1 |
| SE4 | <--- | AI自我效能 | 1.281 | .103 | 12.496 | \*\*\* | a10\_1 |
| SE5 | <--- | AI自我效能 | 1.223 | .099 | 12.352 | \*\*\* | a11\_1 |
| AB1 | <--- | 使用行为 | 1.000 |  |  |  |  |
| AB2 | <--- | 使用行为 | .978 | .071 | 13.768 | \*\*\* | a12\_1 |
| AB3 | <--- | 使用行为 | 1.069 | .071 | 14.969 | \*\*\* | a13\_1 |
| AB5 | <--- | 使用行为 | .970 | .071 | 13.623 | \*\*\* | a14\_1 |

##### Standardized Regression Weights: (east - Structural weights)

|  |  |  | Estimate |
| --- | --- | --- | --- |
| AI自我效能 | <--- | AI威胁感知 | .470 |
| 防御性动机 | <--- | AI威胁感知 | .451 |
| 防御性动机 | <--- | AI自我效能 | .066 |
| 使用行为 | <--- | 防御性动机 | .385 |
| 使用行为 | <--- | AI自我效能 | .400 |
| AA1 | <--- | AI威胁感知 | .827 |
| AA2 | <--- | AI威胁感知 | .774 |
| AA3 | <--- | AI威胁感知 | .732 |
| AA4 | <--- | AI威胁感知 | .764 |
| AA5 | <--- | AI威胁感知 | .793 |
| DM1 | <--- | 防御性动机 | .707 |
| DM2 | <--- | 防御性动机 | .757 |
| DM3 | <--- | 防御性动机 | .733 |
| DM5 | <--- | 防御性动机 | .739 |
| SE1 | <--- | AI自我效能 | .643 |
| SE2 | <--- | AI自我效能 | .721 |
| SE3 | <--- | AI自我效能 | .708 |
| SE4 | <--- | AI自我效能 | .814 |
| SE5 | <--- | AI自我效能 | .785 |
| AB1 | <--- | 使用行为 | .751 |
| AB2 | <--- | 使用行为 | .720 |
| AB3 | <--- | 使用行为 | .806 |
| AB5 | <--- | 使用行为 | .754 |

##### Variances: (east - Structural weights)

|  |  |  | Estimate | S.E. | C.R. | P | Label |
| --- | --- | --- | --- | --- | --- | --- | --- |
| AI威胁感知 |  |  | 2.146 | .309 | 6.952 | \*\*\* | vvv1\_1 |
| e21 |  |  | 1.046 | .197 | 5.296 | \*\*\* | vv1\_1 |
| e23 |  |  | 1.279 | .232 | 5.504 | \*\*\* | vv3\_1 |
| e22 |  |  | 1.103 | .199 | 5.542 | \*\*\* | vv2\_1 |
| e1 |  |  | .988 | .149 | 6.631 | \*\*\* | v1\_1 |
| e2 |  |  | 1.307 | .178 | 7.334 | \*\*\* | v2\_1 |
| e3 |  |  | 1.572 | .205 | 7.679 | \*\*\* | v3\_1 |
| e4 |  |  | 1.292 | .174 | 7.418 | \*\*\* | v4\_1 |
| e5 |  |  | 1.115 | .157 | 7.116 | \*\*\* | v5\_1 |
| e6 |  |  | 1.677 | .231 | 7.250 | \*\*\* | v6\_1 |
| e8 |  |  | 1.311 | .196 | 6.676 | \*\*\* | v7\_1 |
| e9 |  |  | 1.585 | .227 | 6.991 | \*\*\* | v8\_1 |
| e10 |  |  | 1.330 | .193 | 6.895 | \*\*\* | v9\_1 |
| e11 |  |  | 1.904 | .239 | 7.979 | \*\*\* | v10\_1 |
| e12 |  |  | 1.676 | .223 | 7.509 | \*\*\* | v11\_1 |
| e13 |  |  | 1.610 | .212 | 7.603 | \*\*\* | v12\_1 |
| e14 |  |  | 1.123 | .175 | 6.408 | \*\*\* | v13\_1 |
| e15 |  |  | 1.252 | .183 | 6.859 | \*\*\* | v14\_1 |
| e16 |  |  | 1.406 | .199 | 7.054 | \*\*\* | v15\_1 |
| e17 |  |  | 1.618 | .220 | 7.367 | \*\*\* | v16\_1 |
| e18 |  |  | 1.123 | .179 | 6.272 | \*\*\* | v17\_1 |
| e20 |  |  | 1.302 | .186 | 7.009 | \*\*\* | v18\_1 |

##### Squared Multiple Correlations: (east - Structural weights)

|  |  |  | Estimate |
| --- | --- | --- | --- |
| AI自我效能 |  |  | .221 |
| 防御性动机 |  |  | .236 |
| 使用行为 |  |  | .394 |
| AB5 |  |  | .568 |
| AB3 |  |  | .649 |
| AB2 |  |  | .519 |
| AB1 |  |  | .564 |
| SE5 |  |  | .616 |
| SE4 |  |  | .662 |
| SE3 |  |  | .502 |
| SE2 |  |  | .520 |
| SE1 |  |  | .414 |
| DM5 |  |  | .547 |
| DM3 |  |  | .538 |
| DM2 |  |  | .574 |
| DM1 |  |  | .500 |
| AA5 |  |  | .629 |
| AA4 |  |  | .584 |
| AA3 |  |  | .536 |
| AA2 |  |  | .599 |
| AA1 |  |  | .685 |

##### User-defined estimands: (east - Structural weights)

|  |  |  |  |
| --- | --- | --- | --- |
| diff1 |  |  | .000 |
| diff2 |  |  | .027 |
| diff3 |  |  | .094 |
| diff4 |  |  | .030 |
| diff5 |  |  | -.298 |

##### Modification Indices (east - Structural weights)

##### Covariances: (east - Structural weights)

|  |  |  | M.I. | Par Change |
| --- | --- | --- | --- | --- |
| e21 | <--> | AI威胁感知 | 4.623 | .302 |
| e17 | <--> | e22 | 4.061 | -.268 |
| e13 | <--> | e16 | 7.127 | -.381 |
| e4 | <--> | e17 | 4.607 | .293 |
| e4 | <--> | e11 | 5.174 | .325 |
| e2 | <--> | e8 | 4.211 | -.265 |
| e1 | <--> | e15 | 5.510 | .267 |

##### Variances: (east - Structural weights)

|  |  |  | M.I. | Par Change |
| --- | --- | --- | --- | --- |

##### Regression Weights: (east - Structural weights)

|  |  |  | M.I. | Par Change |
| --- | --- | --- | --- | --- |
| AI自我效能 | <--- | AI威胁感知 | 4.623 | .141 |
| AB5 | <--- | SE3 | 4.129 | .114 |
| SE5 | <--- | AA1 | 5.051 | .128 |
| DM5 | <--- | AB5 | 5.454 | -.138 |
| DM5 | <--- | SE2 | 4.342 | -.115 |

##### Bootstrap (east - Structural weights)

##### Bootstrap standard errors (east - Structural weights)

##### Scalar Estimates (east - Structural weights)

##### Regression Weights: (east - Structural weights)

| Parameter | | | SE | SE-SE | Mean | Bias | SE-Bias |
| --- | --- | --- | --- | --- | --- | --- | --- |
| AI自我效能 | <--- | AI威胁感知 | .049 | .001 | .376 | .004 | .002 |
| 防御性动机 | <--- | AI威胁感知 | .063 | .001 | .402 | .003 | .002 |
| 防御性动机 | <--- | AI自我效能 | .080 | .002 | .076 | .002 | .003 |
| 使用行为 | <--- | 防御性动机 | .061 | .001 | .405 | .003 | .002 |
| 使用行为 | <--- | AI自我效能 | .074 | .002 | .464 | -.002 | .002 |
| AA1 | <--- | AI威胁感知 | .000 | .000 | 1.000 | .000 | .000 |
| AA2 | <--- | AI威胁感知 | .048 | .001 | .952 | -.001 | .002 |
| AA3 | <--- | AI威胁感知 | .046 | .001 | .922 | .002 | .001 |
| AA4 | <--- | AI威胁感知 | .051 | .001 | .919 | .000 | .002 |
| AA5 | <--- | AI威胁感知 | .052 | .001 | .941 | .002 | .002 |
| DM1 | <--- | 防御性动机 | .000 | .000 | 1.000 | .000 | .000 |
| DM2 | <--- | 防御性动机 | .073 | .002 | 1.031 | .005 | .002 |
| DM3 | <--- | 防御性动机 | .075 | .002 | 1.057 | .007 | .002 |
| DM5 | <--- | 防御性动机 | .078 | .002 | .983 | .004 | .002 |
| SE1 | <--- | AI自我效能 | .000 | .000 | 1.000 | .000 | .000 |
| SE2 | <--- | AI自我效能 | .093 | .002 | 1.158 | -.005 | .003 |
| SE3 | <--- | AI自我效能 | .088 | .002 | 1.101 | .002 | .003 |
| SE4 | <--- | AI自我效能 | .097 | .002 | 1.278 | -.004 | .003 |
| SE5 | <--- | AI自我效能 | .093 | .002 | 1.222 | -.001 | .003 |
| AB1 | <--- | 使用行为 | .000 | .000 | 1.000 | .000 | .000 |
| AB2 | <--- | 使用行为 | .059 | .001 | .981 | .002 | .002 |
| AB3 | <--- | 使用行为 | .063 | .001 | 1.071 | .003 | .002 |
| AB5 | <--- | 使用行为 | .065 | .001 | .972 | .002 | .002 |

##### Standardized Regression Weights: (east - Structural weights)

| Parameter | | | SE | SE-SE | Mean | Bias | SE-Bias |
| --- | --- | --- | --- | --- | --- | --- | --- |
| AI自我效能 | <--- | AI威胁感知 | .060 | .001 | .473 | .003 | .002 |
| 防御性动机 | <--- | AI威胁感知 | .066 | .001 | .455 | .004 | .002 |
| 防御性动机 | <--- | AI自我效能 | .073 | .002 | .070 | .004 | .002 |
| 使用行为 | <--- | 防御性动机 | .056 | .001 | .386 | .001 | .002 |
| 使用行为 | <--- | AI自我效能 | .060 | .001 | .399 | -.001 | .002 |
| AA1 | <--- | AI威胁感知 | .031 | .001 | .826 | -.001 | .001 |
| AA2 | <--- | AI威胁感知 | .029 | .001 | .773 | -.001 | .001 |
| AA3 | <--- | AI威胁感知 | .034 | .001 | .734 | .001 | .001 |
| AA4 | <--- | AI威胁感知 | .032 | .001 | .764 | .000 | .001 |
| AA5 | <--- | AI威胁感知 | .029 | .001 | .795 | .001 | .001 |
| DM1 | <--- | 防御性动机 | .042 | .001 | .706 | .000 | .001 |
| DM2 | <--- | 防御性动机 | .040 | .001 | .758 | .001 | .001 |
| DM3 | <--- | 防御性动机 | .037 | .001 | .735 | .002 | .001 |
| DM5 | <--- | 防御性动机 | .038 | .001 | .739 | .000 | .001 |
| SE1 | <--- | AI自我效能 | .042 | .001 | .646 | .003 | .001 |
| SE2 | <--- | AI自我效能 | .045 | .001 | .720 | -.001 | .001 |
| SE3 | <--- | AI自我效能 | .037 | .001 | .711 | .003 | .001 |
| SE4 | <--- | AI自我效能 | .030 | .001 | .814 | .000 | .001 |
| SE5 | <--- | AI自我效能 | .034 | .001 | .787 | .002 | .001 |
| AB1 | <--- | 使用行为 | .038 | .001 | .753 | .001 | .001 |
| AB2 | <--- | 使用行为 | .038 | .001 | .723 | .003 | .001 |
| AB3 | <--- | 使用行为 | .031 | .001 | .807 | .001 | .001 |
| AB5 | <--- | 使用行为 | .038 | .001 | .755 | .001 | .001 |

##### Variances: (east - Structural weights)

| Parameter | | | SE | SE-SE | Mean | Bias | SE-Bias |
| --- | --- | --- | --- | --- | --- | --- | --- |
| AI威胁感知 |  |  | .231 | .005 | 2.144 | -.001 | .007 |
| e21 |  |  | .192 | .004 | 1.055 | .009 | .006 |
| e23 |  |  | .226 | .005 | 1.260 | -.019 | .007 |
| e22 |  |  | .186 | .004 | 1.096 | -.007 | .006 |
| e1 |  |  | .165 | .004 | .990 | .002 | .005 |
| e2 |  |  | .156 | .003 | 1.299 | -.008 | .005 |
| e3 |  |  | .190 | .004 | 1.554 | -.017 | .006 |
| e4 |  |  | .163 | .004 | 1.280 | -.012 | .005 |
| e5 |  |  | .132 | .003 | 1.096 | -.019 | .004 |
| e6 |  |  | .224 | .005 | 1.665 | -.012 | .007 |
| e8 |  |  | .187 | .004 | 1.297 | -.014 | .006 |
| e9 |  |  | .211 | .005 | 1.567 | -.019 | .007 |
| e10 |  |  | .170 | .004 | 1.318 | -.012 | .005 |
| e11 |  |  | .212 | .005 | 1.884 | -.020 | .007 |
| e12 |  |  | .268 | .006 | 1.669 | -.007 | .008 |
| e13 |  |  | .184 | .004 | 1.588 | -.022 | .006 |
| e14 |  |  | .172 | .004 | 1.114 | -.009 | .005 |
| e15 |  |  | .188 | .004 | 1.231 | -.021 | .006 |
| e16 |  |  | .196 | .004 | 1.390 | -.017 | .006 |
| e17 |  |  | .235 | .005 | 1.596 | -.022 | .007 |
| e18 |  |  | .163 | .004 | 1.109 | -.014 | .005 |
| e20 |  |  | .183 | .004 | 1.287 | -.015 | .006 |

##### Squared Multiple Correlations: (east - Structural weights)

| Parameter | | | SE | SE-SE | Mean | Bias | SE-Bias |
| --- | --- | --- | --- | --- | --- | --- | --- |
| AI自我效能 |  |  | .057 | .001 | .227 | .006 | .002 |
| 防御性动机 |  |  | .055 | .001 | .249 | .013 | .002 |
| 使用行为 |  |  | .062 | .001 | .402 | .008 | .002 |
| AB5 |  |  | .058 | .001 | .572 | .003 | .002 |
| AB3 |  |  | .050 | .001 | .653 | .003 | .002 |
| AB2 |  |  | .054 | .001 | .524 | .005 | .002 |
| AB1 |  |  | .057 | .001 | .568 | .004 | .002 |
| SE5 |  |  | .053 | .001 | .620 | .004 | .002 |
| SE4 |  |  | .049 | .001 | .663 | .001 | .002 |
| SE3 |  |  | .052 | .001 | .507 | .005 | .002 |
| SE2 |  |  | .065 | .001 | .521 | .001 | .002 |
| SE1 |  |  | .054 | .001 | .419 | .006 | .002 |
| DM5 |  |  | .055 | .001 | .548 | .001 | .002 |
| DM3 |  |  | .054 | .001 | .542 | .004 | .002 |
| DM2 |  |  | .060 | .001 | .576 | .002 | .002 |
| DM1 |  |  | .060 | .001 | .501 | .001 | .002 |
| AA5 |  |  | .045 | .001 | .632 | .003 | .001 |
| AA4 |  |  | .049 | .001 | .585 | .001 | .002 |
| AA3 |  |  | .050 | .001 | .539 | .003 | .002 |
| AA2 |  |  | .045 | .001 | .598 | .000 | .001 |
| AA1 |  |  | .051 | .001 | .684 | -.001 | .002 |

##### User-defined estimands: (east - Structural weights)

| Parameter | | | SE | SE-SE | Mean | Bias | SE-Bias |
| --- | --- | --- | --- | --- | --- | --- | --- |
| diff1 |  |  | .000 | .000 | .000 | .000 | .000 |
| diff2 |  |  | .077 | .002 | .026 | -.001 | .002 |
| diff3 |  |  | .098 | .002 | .088 | -.006 | .003 |
| diff4 |  |  | .077 | .002 | .029 | -.001 | .002 |
| diff5 |  |  | .095 | .002 | -.300 | -.002 | .003 |

##### Bootstrap Confidence (east - Structural weights)

##### Percentile method (east - Structural weights)

##### 95% confidence intervals (percentile method)

##### Scalar Estimates (east - Structural weights)

##### Regression Weights: (east - Structural weights)

| Parameter | | | Estimate | Lower | Upper | P |
| --- | --- | --- | --- | --- | --- | --- |
| AI自我效能 | <--- | AI威胁感知 | .372 | .286 | .474 | .002 |
| 防御性动机 | <--- | AI威胁感知 | .399 | .284 | .531 | .002 |
| 防御性动机 | <--- | AI自我效能 | .074 | -.079 | .228 | .353 |
| 使用行为 | <--- | 防御性动机 | .402 | .291 | .525 | .002 |
| 使用行为 | <--- | AI自我效能 | .466 | .330 | .610 | .002 |
| AA1 | <--- | AI威胁感知 | 1.000 | 1.000 | 1.000 | ... |
| AA2 | <--- | AI威胁感知 | .953 | .864 | 1.047 | .002 |
| AA3 | <--- | AI威胁感知 | .921 | .834 | 1.016 | .002 |
| AA4 | <--- | AI威胁感知 | .919 | .826 | 1.027 | .002 |
| AA5 | <--- | AI威胁感知 | .939 | .841 | 1.053 | .002 |
| DM1 | <--- | 防御性动机 | 1.000 | 1.000 | 1.000 | ... |
| DM2 | <--- | 防御性动机 | 1.026 | .889 | 1.173 | .002 |
| DM3 | <--- | 防御性动机 | 1.050 | .915 | 1.211 | .002 |
| DM5 | <--- | 防御性动机 | .978 | .841 | 1.137 | .002 |
| SE1 | <--- | AI自我效能 | 1.000 | 1.000 | 1.000 | ... |
| SE2 | <--- | AI自我效能 | 1.163 | .981 | 1.356 | .002 |
| SE3 | <--- | AI自我效能 | 1.099 | .943 | 1.288 | .002 |
| SE4 | <--- | AI自我效能 | 1.281 | 1.110 | 1.482 | .002 |
| SE5 | <--- | AI自我效能 | 1.223 | 1.061 | 1.441 | .002 |
| AB1 | <--- | 使用行为 | 1.000 | 1.000 | 1.000 | ... |
| AB2 | <--- | 使用行为 | .978 | .866 | 1.108 | .002 |
| AB3 | <--- | 使用行为 | 1.069 | .959 | 1.203 | .002 |
| AB5 | <--- | 使用行为 | .970 | .855 | 1.117 | .002 |

##### Standardized Regression Weights: (east - Structural weights)

| Parameter | | | Estimate | Lower | Upper | P |
| --- | --- | --- | --- | --- | --- | --- |
| AI自我效能 | <--- | AI威胁感知 | .470 | .353 | .591 | .002 |
| 防御性动机 | <--- | AI威胁感知 | .451 | .335 | .596 | .002 |
| 防御性动机 | <--- | AI自我效能 | .066 | -.068 | .218 | .353 |
| 使用行为 | <--- | 防御性动机 | .385 | .277 | .491 | .002 |
| 使用行为 | <--- | AI自我效能 | .400 | .277 | .513 | .002 |
| AA1 | <--- | AI威胁感知 | .827 | .764 | .883 | .002 |
| AA2 | <--- | AI威胁感知 | .774 | .714 | .828 | .002 |
| AA3 | <--- | AI威胁感知 | .732 | .657 | .798 | .002 |
| AA4 | <--- | AI威胁感知 | .764 | .700 | .824 | .002 |
| AA5 | <--- | AI威胁感知 | .793 | .738 | .848 | .002 |
| DM1 | <--- | 防御性动机 | .707 | .621 | .784 | .002 |
| DM2 | <--- | 防御性动机 | .757 | .684 | .837 | .002 |
| DM3 | <--- | 防御性动机 | .733 | .657 | .806 | .002 |
| DM5 | <--- | 防御性动机 | .739 | .659 | .808 | .002 |
| SE1 | <--- | AI自我效能 | .643 | .556 | .730 | .002 |
| SE2 | <--- | AI自我效能 | .721 | .631 | .809 | .002 |
| SE3 | <--- | AI自我效能 | .708 | .639 | .781 | .002 |
| SE4 | <--- | AI自我效能 | .814 | .748 | .868 | .002 |
| SE5 | <--- | AI自我效能 | .785 | .715 | .850 | .002 |
| AB1 | <--- | 使用行为 | .751 | .673 | .822 | .002 |
| AB2 | <--- | 使用行为 | .720 | .649 | .798 | .002 |
| AB3 | <--- | 使用行为 | .806 | .744 | .867 | .002 |
| AB5 | <--- | 使用行为 | .754 | .679 | .829 | .002 |

##### Variances: (east - Structural weights)

| Parameter | | | Estimate | Lower | Upper | P |
| --- | --- | --- | --- | --- | --- | --- |
| AI威胁感知 |  |  | 2.146 | 1.698 | 2.605 | .002 |
| e21 |  |  | 1.046 | .728 | 1.469 | .002 |
| e23 |  |  | 1.279 | .848 | 1.741 | .002 |
| e22 |  |  | 1.103 | .739 | 1.458 | .002 |
| e1 |  |  | .988 | .672 | 1.316 | .002 |
| e2 |  |  | 1.307 | 1.009 | 1.618 | .002 |
| e3 |  |  | 1.572 | 1.214 | 1.963 | .002 |
| e4 |  |  | 1.292 | .965 | 1.602 | .002 |
| e5 |  |  | 1.115 | .840 | 1.352 | .002 |
| e6 |  |  | 1.677 | 1.246 | 2.104 | .002 |
| e8 |  |  | 1.311 | .907 | 1.664 | .002 |
| e9 |  |  | 1.585 | 1.187 | 2.011 | .002 |
| e10 |  |  | 1.330 | .981 | 1.672 | .002 |
| e11 |  |  | 1.904 | 1.490 | 2.336 | .002 |
| e12 |  |  | 1.676 | 1.156 | 2.211 | .002 |
| e13 |  |  | 1.610 | 1.225 | 1.956 | .002 |
| e14 |  |  | 1.123 | .798 | 1.472 | .002 |
| e15 |  |  | 1.252 | .883 | 1.618 | .002 |
| e16 |  |  | 1.406 | 1.021 | 1.798 | .002 |
| e17 |  |  | 1.618 | 1.150 | 2.089 | .002 |
| e18 |  |  | 1.123 | .798 | 1.434 | .002 |
| e20 |  |  | 1.302 | .938 | 1.653 | .002 |

##### Squared Multiple Correlations: (east - Structural weights)

| Parameter | | | Estimate | Lower | Upper | P |
| --- | --- | --- | --- | --- | --- | --- |
| AI自我效能 |  |  | .221 | .125 | .350 | .002 |
| 防御性动机 |  |  | .236 | .151 | .375 | .002 |
| 使用行为 |  |  | .394 | .289 | .530 | .002 |
| AB5 |  |  | .568 | .461 | .687 | .002 |
| AB3 |  |  | .649 | .554 | .752 | .002 |
| AB2 |  |  | .519 | .421 | .637 | .002 |
| AB1 |  |  | .564 | .453 | .676 | .002 |
| SE5 |  |  | .616 | .511 | .723 | .002 |
| SE4 |  |  | .662 | .560 | .754 | .002 |
| SE3 |  |  | .502 | .408 | .609 | .002 |
| SE2 |  |  | .520 | .398 | .654 | .002 |
| SE1 |  |  | .414 | .309 | .533 | .002 |
| DM5 |  |  | .547 | .434 | .653 | .002 |
| DM3 |  |  | .538 | .432 | .649 | .002 |
| DM2 |  |  | .574 | .469 | .701 | .002 |
| DM1 |  |  | .500 | .385 | .615 | .002 |
| AA5 |  |  | .629 | .545 | .720 | .002 |
| AA4 |  |  | .584 | .489 | .679 | .002 |
| AA3 |  |  | .536 | .432 | .637 | .002 |
| AA2 |  |  | .599 | .509 | .685 | .002 |
| AA1 |  |  | .685 | .584 | .780 | .002 |

##### User-defined estimands: (east - Structural weights)

| Parameter | | | Estimate | Lower | Upper | P |
| --- | --- | --- | --- | --- | --- | --- |
| diff1 |  |  | .000 | .000 | .000 | ... |
| diff2 |  |  | .027 | -.127 | .175 | .744 |
| diff3 |  |  | .094 | -.091 | .285 | .354 |
| diff4 |  |  | .030 | -.125 | .193 | .726 |
| diff5 |  |  | -.298 | -.489 | -.123 | .002 |

##### Bias-corrected percentile method (east - Structural weights)

##### 95% confidence intervals (bias-corrected percentile method)

##### Scalar Estimates (east - Structural weights)

##### Regression Weights: (east - Structural weights)

| Parameter | | | Estimate | Lower | Upper | P |
| --- | --- | --- | --- | --- | --- | --- |
| AI自我效能 | <--- | AI威胁感知 | .372 | .273 | .466 | .003 |
| 防御性动机 | <--- | AI威胁感知 | .399 | .275 | .522 | .003 |
| 防御性动机 | <--- | AI自我效能 | .074 | -.079 | .226 | .364 |
| 使用行为 | <--- | 防御性动机 | .402 | .284 | .523 | .002 |
| 使用行为 | <--- | AI自我效能 | .466 | .332 | .617 | .002 |
| AA1 | <--- | AI威胁感知 | 1.000 | 1.000 | 1.000 | ... |
| AA2 | <--- | AI威胁感知 | .953 | .867 | 1.056 | .001 |
| AA3 | <--- | AI威胁感知 | .921 | .829 | 1.013 | .002 |
| AA4 | <--- | AI威胁感知 | .919 | .833 | 1.030 | .002 |
| AA5 | <--- | AI威胁感知 | .939 | .839 | 1.048 | .002 |
| DM1 | <--- | 防御性动机 | 1.000 | 1.000 | 1.000 | ... |
| DM2 | <--- | 防御性动机 | 1.026 | .884 | 1.166 | .003 |
| DM3 | <--- | 防御性动机 | 1.050 | .903 | 1.205 | .003 |
| DM5 | <--- | 防御性动机 | .978 | .836 | 1.134 | .002 |
| SE1 | <--- | AI自我效能 | 1.000 | 1.000 | 1.000 | ... |
| SE2 | <--- | AI自我效能 | 1.163 | 1.005 | 1.366 | .001 |
| SE3 | <--- | AI自我效能 | 1.099 | .951 | 1.293 | .001 |
| SE4 | <--- | AI自我效能 | 1.281 | 1.120 | 1.499 | .001 |
| SE5 | <--- | AI自我效能 | 1.223 | 1.066 | 1.457 | .001 |
| AB1 | <--- | 使用行为 | 1.000 | 1.000 | 1.000 | ... |
| AB2 | <--- | 使用行为 | .978 | .870 | 1.116 | .002 |
| AB3 | <--- | 使用行为 | 1.069 | .956 | 1.202 | .002 |
| AB5 | <--- | 使用行为 | .970 | .858 | 1.128 | .001 |

##### Standardized Regression Weights: (east - Structural weights)

| Parameter | | | Estimate | Lower | Upper | P |
| --- | --- | --- | --- | --- | --- | --- |
| AI自我效能 | <--- | AI威胁感知 | .470 | .346 | .585 | .003 |
| 防御性动机 | <--- | AI威胁感知 | .451 | .318 | .583 | .003 |
| 防御性动机 | <--- | AI自我效能 | .066 | -.071 | .214 | .374 |
| 使用行为 | <--- | 防御性动机 | .385 | .269 | .482 | .003 |
| 使用行为 | <--- | AI自我效能 | .400 | .279 | .513 | .002 |
| AA1 | <--- | AI威胁感知 | .827 | .762 | .882 | .002 |
| AA2 | <--- | AI威胁感知 | .774 | .712 | .824 | .002 |
| AA3 | <--- | AI威胁感知 | .732 | .651 | .794 | .004 |
| AA4 | <--- | AI威胁感知 | .764 | .700 | .824 | .002 |
| AA5 | <--- | AI威胁感知 | .793 | .730 | .843 | .004 |
| DM1 | <--- | 防御性动机 | .707 | .625 | .790 | .002 |
| DM2 | <--- | 防御性动机 | .757 | .681 | .834 | .003 |
| DM3 | <--- | 防御性动机 | .733 | .649 | .799 | .004 |
| DM5 | <--- | 防御性动机 | .739 | .654 | .806 | .003 |
| SE1 | <--- | AI自我效能 | .643 | .549 | .718 | .003 |
| SE2 | <--- | AI自我效能 | .721 | .631 | .809 | .002 |
| SE3 | <--- | AI自我效能 | .708 | .631 | .772 | .004 |
| SE4 | <--- | AI自我效能 | .814 | .745 | .866 | .003 |
| SE5 | <--- | AI自我效能 | .785 | .710 | .844 | .004 |
| AB1 | <--- | 使用行为 | .751 | .668 | .819 | .003 |
| AB2 | <--- | 使用行为 | .720 | .641 | .791 | .003 |
| AB3 | <--- | 使用行为 | .806 | .739 | .864 | .003 |
| AB5 | <--- | 使用行为 | .754 | .673 | .825 | .003 |

##### Variances: (east - Structural weights)

| Parameter | | | Estimate | Lower | Upper | P |
| --- | --- | --- | --- | --- | --- | --- |
| AI威胁感知 |  |  | 2.146 | 1.707 | 2.610 | .002 |
| e21 |  |  | 1.046 | .729 | 1.473 | .002 |
| e23 |  |  | 1.279 | .912 | 1.855 | .001 |
| e22 |  |  | 1.103 | .746 | 1.472 | .002 |
| e1 |  |  | .988 | .683 | 1.325 | .002 |
| e2 |  |  | 1.307 | 1.025 | 1.631 | .001 |
| e3 |  |  | 1.572 | 1.246 | 2.008 | .001 |
| e4 |  |  | 1.292 | 1.005 | 1.631 | .001 |
| e5 |  |  | 1.115 | .887 | 1.399 | .001 |
| e6 |  |  | 1.677 | 1.283 | 2.177 | .001 |
| e8 |  |  | 1.311 | .922 | 1.682 | .001 |
| e9 |  |  | 1.585 | 1.234 | 2.107 | .001 |
| e10 |  |  | 1.330 | 1.024 | 1.705 | .001 |
| e11 |  |  | 1.904 | 1.521 | 2.364 | .001 |
| e12 |  |  | 1.676 | 1.179 | 2.217 | .002 |
| e13 |  |  | 1.610 | 1.292 | 1.990 | .001 |
| e14 |  |  | 1.123 | .824 | 1.500 | .001 |
| e15 |  |  | 1.252 | .940 | 1.654 | .001 |
| e16 |  |  | 1.406 | 1.048 | 1.834 | .001 |
| e17 |  |  | 1.618 | 1.202 | 2.127 | .001 |
| e18 |  |  | 1.123 | .813 | 1.446 | .001 |
| e20 |  |  | 1.302 | .971 | 1.694 | .001 |

##### Squared Multiple Correlations: (east - Structural weights)

| Parameter | | | Estimate | Lower | Upper | P |
| --- | --- | --- | --- | --- | --- | --- |
| AI自我效能 |  |  | .221 | .120 | .342 | .003 |
| 防御性动机 |  |  | .236 | .139 | .347 | .006 |
| 使用行为 |  |  | .394 | .275 | .516 | .004 |
| AB5 |  |  | .568 | .453 | .680 | .003 |
| AB3 |  |  | .649 | .546 | .746 | .003 |
| AB2 |  |  | .519 | .411 | .626 | .003 |
| AB1 |  |  | .564 | .446 | .671 | .003 |
| SE5 |  |  | .616 | .504 | .713 | .004 |
| SE4 |  |  | .662 | .555 | .750 | .003 |
| SE3 |  |  | .502 | .398 | .596 | .004 |
| SE2 |  |  | .520 | .398 | .654 | .002 |
| SE1 |  |  | .414 | .302 | .516 | .003 |
| DM5 |  |  | .547 | .428 | .649 | .003 |
| DM3 |  |  | .538 | .421 | .638 | .004 |
| DM2 |  |  | .574 | .463 | .696 | .003 |
| DM1 |  |  | .500 | .391 | .624 | .002 |
| AA5 |  |  | .629 | .534 | .711 | .004 |
| AA4 |  |  | .584 | .490 | .680 | .002 |
| AA3 |  |  | .536 | .424 | .630 | .004 |
| AA2 |  |  | .599 | .507 | .680 | .002 |
| AA1 |  |  | .685 | .581 | .777 | .002 |

##### User-defined estimands: (east - Structural weights)

| Parameter | | | Estimate | Lower | Upper | P |
| --- | --- | --- | --- | --- | --- | --- |
| diff1 |  |  | .000 | .000 | .000 | ... |
| diff2 |  |  | .027 | -.125 | .175 | .729 |
| diff3 |  |  | .094 | -.080 | .308 | .275 |
| diff4 |  |  | .030 | -.121 | .197 | .696 |
| diff5 |  |  | -.298 | -.489 | -.123 | .002 |

##### other (other - Structural weights)

##### Estimates (other - Structural weights)

##### Scalar Estimates (other - Structural weights)

##### Maximum Likelihood Estimates

##### Regression Weights: (other - Structural weights)

|  |  |  | Estimate | S.E. | C.R. | P | Label |
| --- | --- | --- | --- | --- | --- | --- | --- |
| AI自我效能 | <--- | AI威胁感知 | .372 | .049 | 7.568 | \*\*\* | H1a |
| 防御性动机 | <--- | AI威胁感知 | .399 | .062 | 6.474 | \*\*\* | H2a |
| 防御性动机 | <--- | AI自我效能 | .074 | .074 | .999 | .318 | H5a |
| 使用行为 | <--- | 防御性动机 | .402 | .064 | 6.252 | \*\*\* | H4a |
| 使用行为 | <--- | AI自我效能 | .466 | .074 | 6.313 | \*\*\* | H3a |
| AA1 | <--- | AI威胁感知 | 1.000 |  |  |  |  |
| AA2 | <--- | AI威胁感知 | .953 | .057 | 16.829 | \*\*\* | a1\_1 |
| AA3 | <--- | AI威胁感知 | .921 | .058 | 15.919 | \*\*\* | a2\_1 |
| AA4 | <--- | AI威胁感知 | .919 | .058 | 15.976 | \*\*\* | a3\_1 |
| AA5 | <--- | AI威胁感知 | .939 | .056 | 16.729 | \*\*\* | a4\_1 |
| DM1 | <--- | 防御性动机 | 1.000 |  |  |  |  |
| DM2 | <--- | 防御性动机 | 1.026 | .082 | 12.532 | \*\*\* | a5\_1 |
| DM3 | <--- | 防御性动机 | 1.050 | .082 | 12.782 | \*\*\* | a6\_1 |
| DM5 | <--- | 防御性动机 | .978 | .081 | 12.070 | \*\*\* | a7\_1 |
| SE1 | <--- | AI自我效能 | 1.000 |  |  |  |  |
| SE2 | <--- | AI自我效能 | 1.163 | .101 | 11.499 | \*\*\* | a8\_1 |
| SE3 | <--- | AI自我效能 | 1.099 | .097 | 11.301 | \*\*\* | a9\_1 |
| SE4 | <--- | AI自我效能 | 1.281 | .103 | 12.496 | \*\*\* | a10\_1 |
| SE5 | <--- | AI自我效能 | 1.223 | .099 | 12.352 | \*\*\* | a11\_1 |
| AB1 | <--- | 使用行为 | 1.000 |  |  |  |  |
| AB2 | <--- | 使用行为 | .978 | .071 | 13.768 | \*\*\* | a12\_1 |
| AB3 | <--- | 使用行为 | 1.069 | .071 | 14.969 | \*\*\* | a13\_1 |
| AB5 | <--- | 使用行为 | .970 | .071 | 13.623 | \*\*\* | a14\_1 |

##### Standardized Regression Weights: (other - Structural weights)

|  |  |  | Estimate |
| --- | --- | --- | --- |
| AI自我效能 | <--- | AI威胁感知 | .487 |
| 防御性动机 | <--- | AI威胁感知 | .455 |
| 防御性动机 | <--- | AI自我效能 | .064 |
| 使用行为 | <--- | 防御性动机 | .376 |
| 使用行为 | <--- | AI自我效能 | .380 |
| AA1 | <--- | AI威胁感知 | .813 |
| AA2 | <--- | AI威胁感知 | .791 |
| AA3 | <--- | AI威胁感知 | .762 |
| AA4 | <--- | AI威胁感知 | .744 |
| AA5 | <--- | AI威胁感知 | .770 |
| DM1 | <--- | 防御性动机 | .707 |
| DM2 | <--- | 防御性动机 | .730 |
| DM3 | <--- | 防御性动机 | .782 |
| DM5 | <--- | 防御性动机 | .684 |
| SE1 | <--- | AI自我效能 | .632 |
| SE2 | <--- | AI自我效能 | .710 |
| SE3 | <--- | AI自我效能 | .692 |
| SE4 | <--- | AI自我效能 | .798 |
| SE5 | <--- | AI自我效能 | .794 |
| AB1 | <--- | 使用行为 | .761 |
| AB2 | <--- | 使用行为 | .749 |
| AB3 | <--- | 使用行为 | .811 |
| AB5 | <--- | 使用行为 | .712 |

##### Variances: (other - Structural weights)

|  |  |  | Estimate | S.E. | C.R. | P | Label |
| --- | --- | --- | --- | --- | --- | --- | --- |
| AI威胁感知 |  |  | 2.216 | .279 | 7.933 | \*\*\* | vvv1\_2 |
| e21 |  |  | .987 | .171 | 5.780 | \*\*\* | vv1\_2 |
| e23 |  |  | 1.293 | .209 | 6.189 | \*\*\* | vv3\_2 |
| e22 |  |  | 1.226 | .190 | 6.464 | \*\*\* | vv2\_2 |
| e1 |  |  | 1.139 | .139 | 8.208 | \*\*\* | v1\_2 |
| e2 |  |  | 1.208 | .141 | 8.558 | \*\*\* | v2\_2 |
| e3 |  |  | 1.354 | .152 | 8.920 | \*\*\* | v3\_2 |
| e4 |  |  | 1.512 | .166 | 9.124 | \*\*\* | v4\_2 |
| e5 |  |  | 1.339 | .151 | 8.839 | \*\*\* | v5\_2 |
| e6 |  |  | 1.698 | .196 | 8.646 | \*\*\* | v6\_2 |
| e8 |  |  | 1.574 | .188 | 8.366 | \*\*\* | v7\_2 |
| e9 |  |  | 1.193 | .160 | 7.444 | \*\*\* | v8\_2 |
| e10 |  |  | 1.850 | .207 | 8.918 | \*\*\* | v9\_2 |
| e11 |  |  | 1.943 | .201 | 9.649 | \*\*\* | v10\_2 |
| e12 |  |  | 1.716 | .189 | 9.081 | \*\*\* | v11\_2 |
| e13 |  |  | 1.703 | .184 | 9.247 | \*\*\* | v12\_2 |
| e14 |  |  | 1.214 | .154 | 7.898 | \*\*\* | v13\_2 |
| e15 |  |  | 1.132 | .142 | 7.955 | \*\*\* | v14\_2 |
| e16 |  |  | 1.414 | .170 | 8.325 | \*\*\* | v15\_2 |
| e17 |  |  | 1.456 | .171 | 8.491 | \*\*\* | v16\_2 |
| e18 |  |  | 1.154 | .157 | 7.371 | \*\*\* | v17\_2 |
| e20 |  |  | 1.773 | .198 | 8.946 | \*\*\* | v18\_2 |

##### Squared Multiple Correlations: (other - Structural weights)

|  |  |  | Estimate |
| --- | --- | --- | --- |
| AI自我效能 |  |  | .237 |
| 防御性动机 |  |  | .240 |
| 使用行为 |  |  | .368 |
| AB5 |  |  | .507 |
| AB3 |  |  | .658 |
| AB2 |  |  | .561 |
| AB1 |  |  | .579 |
| SE5 |  |  | .631 |
| SE4 |  |  | .636 |
| SE3 |  |  | .478 |
| SE2 |  |  | .505 |
| SE1 |  |  | .400 |
| DM5 |  |  | .468 |
| DM3 |  |  | .611 |
| DM2 |  |  | .532 |
| DM1 |  |  | .500 |
| AA5 |  |  | .593 |
| AA4 |  |  | .553 |
| AA3 |  |  | .581 |
| AA2 |  |  | .625 |
| AA1 |  |  | .661 |

##### User-defined estimands: (other - Structural weights)

|  |  |  |  |
| --- | --- | --- | --- |
| diff1 |  |  | .000 |
| diff2 |  |  | .027 |
| diff3 |  |  | .094 |
| diff4 |  |  | .030 |
| diff5 |  |  | -.298 |

##### Modification Indices (other - Structural weights)

##### Covariances: (other - Structural weights)

|  |  |  | M.I. | Par Change |
| --- | --- | --- | --- | --- |
| e20 | <--> | e23 | 5.527 | -.291 |
| e13 | <--> | AI威胁感知 | 5.629 | -.345 |
| e9 | <--> | e18 | 4.515 | .224 |
| e8 | <--> | e22 | 6.821 | .309 |
| e6 | <--> | e11 | 5.449 | -.322 |
| e5 | <--> | e14 | 12.441 | -.370 |
| e4 | <--> | e5 | 4.608 | .233 |
| e3 | <--> | e17 | 4.137 | .226 |
| e2 | <--> | e14 | 6.192 | .251 |
| e2 | <--> | e13 | 4.275 | -.231 |
| e1 | <--> | e3 | 5.115 | .222 |

##### Variances: (other - Structural weights)

|  |  |  | M.I. | Par Change |
| --- | --- | --- | --- | --- |

##### Regression Weights: (other - Structural weights)

|  |  |  | M.I. | Par Change |
| --- | --- | --- | --- | --- |
| AB2 | <--- | SE3 | 4.030 | -.098 |
| SE4 | <--- | AA2 | 4.141 | .094 |
| SE3 | <--- | AI威胁感知 | 5.629 | -.155 |
| SE3 | <--- | AA3 | 5.384 | -.119 |
| SE3 | <--- | AA2 | 8.503 | -.150 |
| SE1 | <--- | DM1 | 7.046 | -.139 |
| DM2 | <--- | AB2 | 4.160 | .103 |
| DM2 | <--- | AB1 | 4.425 | .106 |
| DM1 | <--- | SE1 | 5.376 | -.122 |
| AA5 | <--- | SE4 | 6.367 | -.115 |

##### Bootstrap (other - Structural weights)

##### Bootstrap standard errors (other - Structural weights)

##### Scalar Estimates (other - Structural weights)

##### Regression Weights: (other - Structural weights)

| Parameter | | | SE | SE-SE | Mean | Bias | SE-Bias |
| --- | --- | --- | --- | --- | --- | --- | --- |
| AI自我效能 | <--- | AI威胁感知 | .049 | .001 | .376 | .004 | .002 |
| 防御性动机 | <--- | AI威胁感知 | .063 | .001 | .402 | .003 | .002 |
| 防御性动机 | <--- | AI自我效能 | .080 | .002 | .076 | .002 | .003 |
| 使用行为 | <--- | 防御性动机 | .061 | .001 | .405 | .003 | .002 |
| 使用行为 | <--- | AI自我效能 | .074 | .002 | .464 | -.002 | .002 |
| AA1 | <--- | AI威胁感知 | .000 | .000 | 1.000 | .000 | .000 |
| AA2 | <--- | AI威胁感知 | .048 | .001 | .952 | -.001 | .002 |
| AA3 | <--- | AI威胁感知 | .046 | .001 | .922 | .002 | .001 |
| AA4 | <--- | AI威胁感知 | .051 | .001 | .919 | .000 | .002 |
| AA5 | <--- | AI威胁感知 | .052 | .001 | .941 | .002 | .002 |
| DM1 | <--- | 防御性动机 | .000 | .000 | 1.000 | .000 | .000 |
| DM2 | <--- | 防御性动机 | .073 | .002 | 1.031 | .005 | .002 |
| DM3 | <--- | 防御性动机 | .075 | .002 | 1.057 | .007 | .002 |
| DM5 | <--- | 防御性动机 | .078 | .002 | .983 | .004 | .002 |
| SE1 | <--- | AI自我效能 | .000 | .000 | 1.000 | .000 | .000 |
| SE2 | <--- | AI自我效能 | .093 | .002 | 1.158 | -.005 | .003 |
| SE3 | <--- | AI自我效能 | .088 | .002 | 1.101 | .002 | .003 |
| SE4 | <--- | AI自我效能 | .097 | .002 | 1.278 | -.004 | .003 |
| SE5 | <--- | AI自我效能 | .093 | .002 | 1.222 | -.001 | .003 |
| AB1 | <--- | 使用行为 | .000 | .000 | 1.000 | .000 | .000 |
| AB2 | <--- | 使用行为 | .059 | .001 | .981 | .002 | .002 |
| AB3 | <--- | 使用行为 | .063 | .001 | 1.071 | .003 | .002 |
| AB5 | <--- | 使用行为 | .065 | .001 | .972 | .002 | .002 |

##### Standardized Regression Weights: (other - Structural weights)

| Parameter | | | SE | SE-SE | Mean | Bias | SE-Bias |
| --- | --- | --- | --- | --- | --- | --- | --- |
| AI自我效能 | <--- | AI威胁感知 | .045 | .001 | .488 | .001 | .001 |
| 防御性动机 | <--- | AI威胁感知 | .066 | .001 | .459 | .004 | .002 |
| 防御性动机 | <--- | AI自我效能 | .069 | .002 | .067 | .002 | .002 |
| 使用行为 | <--- | 防御性动机 | .055 | .001 | .377 | .001 | .002 |
| 使用行为 | <--- | AI自我效能 | .051 | .001 | .379 | -.002 | .002 |
| AA1 | <--- | AI威胁感知 | .026 | .001 | .813 | .000 | .001 |
| AA2 | <--- | AI威胁感知 | .029 | .001 | .789 | -.002 | .001 |
| AA3 | <--- | AI威胁感知 | .028 | .001 | .763 | .001 | .001 |
| AA4 | <--- | AI威胁感知 | .032 | .001 | .743 | -.001 | .001 |
| AA5 | <--- | AI威胁感知 | .033 | .001 | .771 | .001 | .001 |
| DM1 | <--- | 防御性动机 | .036 | .001 | .705 | -.002 | .001 |
| DM2 | <--- | 防御性动机 | .034 | .001 | .730 | .000 | .001 |
| DM3 | <--- | 防御性动机 | .031 | .001 | .783 | .001 | .001 |
| DM5 | <--- | 防御性动机 | .039 | .001 | .684 | .000 | .001 |
| SE1 | <--- | AI自我效能 | .038 | .001 | .635 | .003 | .001 |
| SE2 | <--- | AI自我效能 | .035 | .001 | .710 | -.001 | .001 |
| SE3 | <--- | AI自我效能 | .037 | .001 | .694 | .002 | .001 |
| SE4 | <--- | AI自我效能 | .029 | .001 | .797 | -.001 | .001 |
| SE5 | <--- | AI自我效能 | .029 | .001 | .795 | .001 | .001 |
| AB1 | <--- | 使用行为 | .030 | .001 | .762 | .001 | .001 |
| AB2 | <--- | 使用行为 | .034 | .001 | .749 | .001 | .001 |
| AB3 | <--- | 使用行为 | .029 | .001 | .812 | .001 | .001 |
| AB5 | <--- | 使用行为 | .031 | .001 | .713 | .001 | .001 |

##### Variances: (other - Structural weights)

| Parameter | | | SE | SE-SE | Mean | Bias | SE-Bias |
| --- | --- | --- | --- | --- | --- | --- | --- |
| AI威胁感知 |  |  | .232 | .005 | 2.218 | .001 | .007 |
| e21 |  |  | .150 | .003 | .994 | .007 | .005 |
| e23 |  |  | .187 | .004 | 1.268 | -.025 | .006 |
| e22 |  |  | .167 | .004 | 1.219 | -.007 | .005 |
| e1 |  |  | .133 | .003 | 1.129 | -.010 | .004 |
| e2 |  |  | .145 | .003 | 1.210 | .002 | .005 |
| e3 |  |  | .134 | .003 | 1.342 | -.012 | .004 |
| e4 |  |  | .169 | .004 | 1.505 | -.007 | .005 |
| e5 |  |  | .178 | .004 | 1.326 | -.013 | .006 |
| e6 |  |  | .183 | .004 | 1.698 | .000 | .006 |
| e8 |  |  | .189 | .004 | 1.562 | -.012 | .006 |
| e9 |  |  | .136 | .003 | 1.175 | -.018 | .004 |
| e10 |  |  | .191 | .004 | 1.837 | -.013 | .006 |
| e11 |  |  | .168 | .004 | 1.923 | -.020 | .005 |
| e12 |  |  | .178 | .004 | 1.702 | -.014 | .006 |
| e13 |  |  | .176 | .004 | 1.682 | -.022 | .006 |
| e14 |  |  | .143 | .003 | 1.208 | -.006 | .005 |
| e15 |  |  | .142 | .003 | 1.120 | -.012 | .004 |
| e16 |  |  | .148 | .003 | 1.394 | -.020 | .005 |
| e17 |  |  | .168 | .004 | 1.446 | -.010 | .005 |
| e18 |  |  | .165 | .004 | 1.145 | -.009 | .005 |
| e20 |  |  | .174 | .004 | 1.759 | -.015 | .005 |

##### Squared Multiple Correlations: (other - Structural weights)

| Parameter | | | SE | SE-SE | Mean | Bias | SE-Bias |
| --- | --- | --- | --- | --- | --- | --- | --- |
| AI自我效能 |  |  | .043 | .001 | .241 | .003 | .001 |
| 防御性动机 |  |  | .051 | .001 | .252 | .012 | .002 |
| 使用行为 |  |  | .052 | .001 | .373 | .005 | .002 |
| AB5 |  |  | .045 | .001 | .510 | .002 | .001 |
| AB3 |  |  | .047 | .001 | .660 | .002 | .001 |
| AB2 |  |  | .050 | .001 | .563 | .002 | .002 |
| AB1 |  |  | .046 | .001 | .582 | .003 | .001 |
| SE5 |  |  | .045 | .001 | .633 | .002 | .001 |
| SE4 |  |  | .045 | .001 | .636 | -.001 | .001 |
| SE3 |  |  | .051 | .001 | .483 | .004 | .002 |
| SE2 |  |  | .049 | .001 | .505 | .000 | .002 |
| SE1 |  |  | .048 | .001 | .404 | .005 | .002 |
| DM5 |  |  | .053 | .001 | .469 | .001 | .002 |
| DM3 |  |  | .049 | .001 | .614 | .003 | .002 |
| DM2 |  |  | .049 | .001 | .534 | .001 | .002 |
| DM1 |  |  | .051 | .001 | .499 | -.002 | .002 |
| AA5 |  |  | .051 | .001 | .596 | .002 | .002 |
| AA4 |  |  | .048 | .001 | .553 | .000 | .002 |
| AA3 |  |  | .043 | .001 | .583 | .002 | .001 |
| AA2 |  |  | .045 | .001 | .623 | -.002 | .001 |
| AA1 |  |  | .043 | .001 | .662 | .001 | .001 |

##### User-defined estimands: (other - Structural weights)

| Parameter | | | SE | SE-SE | Mean | Bias | SE-Bias |
| --- | --- | --- | --- | --- | --- | --- | --- |
| diff1 |  |  | .000 | .000 | .000 | .000 | .000 |
| diff2 |  |  | .077 | .002 | .026 | -.001 | .002 |
| diff3 |  |  | .098 | .002 | .088 | -.006 | .003 |
| diff4 |  |  | .077 | .002 | .029 | -.001 | .002 |
| diff5 |  |  | .095 | .002 | -.300 | -.002 | .003 |

##### Bootstrap Confidence (other - Structural weights)

##### Percentile method (other - Structural weights)

##### 95% confidence intervals (percentile method)

##### Scalar Estimates (other - Structural weights)

##### Regression Weights: (other - Structural weights)

| Parameter | | | Estimate | Lower | Upper | P |
| --- | --- | --- | --- | --- | --- | --- |
| AI自我效能 | <--- | AI威胁感知 | .372 | .286 | .474 | .002 |
| 防御性动机 | <--- | AI威胁感知 | .399 | .284 | .531 | .002 |
| 防御性动机 | <--- | AI自我效能 | .074 | -.079 | .228 | .353 |
| 使用行为 | <--- | 防御性动机 | .402 | .291 | .525 | .002 |
| 使用行为 | <--- | AI自我效能 | .466 | .330 | .610 | .002 |
| AA1 | <--- | AI威胁感知 | 1.000 | 1.000 | 1.000 | ... |
| AA2 | <--- | AI威胁感知 | .953 | .864 | 1.047 | .002 |
| AA3 | <--- | AI威胁感知 | .921 | .834 | 1.016 | .002 |
| AA4 | <--- | AI威胁感知 | .919 | .826 | 1.027 | .002 |
| AA5 | <--- | AI威胁感知 | .939 | .841 | 1.053 | .002 |
| DM1 | <--- | 防御性动机 | 1.000 | 1.000 | 1.000 | ... |
| DM2 | <--- | 防御性动机 | 1.026 | .889 | 1.173 | .002 |
| DM3 | <--- | 防御性动机 | 1.050 | .915 | 1.211 | .002 |
| DM5 | <--- | 防御性动机 | .978 | .841 | 1.137 | .002 |
| SE1 | <--- | AI自我效能 | 1.000 | 1.000 | 1.000 | ... |
| SE2 | <--- | AI自我效能 | 1.163 | .981 | 1.356 | .002 |
| SE3 | <--- | AI自我效能 | 1.099 | .943 | 1.288 | .002 |
| SE4 | <--- | AI自我效能 | 1.281 | 1.110 | 1.482 | .002 |
| SE5 | <--- | AI自我效能 | 1.223 | 1.061 | 1.441 | .002 |
| AB1 | <--- | 使用行为 | 1.000 | 1.000 | 1.000 | ... |
| AB2 | <--- | 使用行为 | .978 | .866 | 1.108 | .002 |
| AB3 | <--- | 使用行为 | 1.069 | .959 | 1.203 | .002 |
| AB5 | <--- | 使用行为 | .970 | .855 | 1.117 | .002 |

##### Standardized Regression Weights: (other - Structural weights)

| Parameter | | | Estimate | Lower | Upper | P |
| --- | --- | --- | --- | --- | --- | --- |
| AI自我效能 | <--- | AI威胁感知 | .487 | .398 | .575 | .002 |
| 防御性动机 | <--- | AI威胁感知 | .455 | .336 | .587 | .002 |
| 防御性动机 | <--- | AI自我效能 | .064 | -.067 | .197 | .353 |
| 使用行为 | <--- | 防御性动机 | .376 | .266 | .484 | .002 |
| 使用行为 | <--- | AI自我效能 | .380 | .275 | .475 | .002 |
| AA1 | <--- | AI威胁感知 | .813 | .758 | .862 | .002 |
| AA2 | <--- | AI威胁感知 | .791 | .730 | .841 | .002 |
| AA3 | <--- | AI威胁感知 | .762 | .705 | .816 | .002 |
| AA4 | <--- | AI威胁感知 | .744 | .677 | .803 | .002 |
| AA5 | <--- | AI威胁感知 | .770 | .705 | .831 | .002 |
| DM1 | <--- | 防御性动机 | .707 | .632 | .773 | .002 |
| DM2 | <--- | 防御性动机 | .730 | .660 | .796 | .002 |
| DM3 | <--- | 防御性动机 | .782 | .717 | .843 | .002 |
| DM5 | <--- | 防御性动机 | .684 | .607 | .759 | .002 |
| SE1 | <--- | AI自我效能 | .632 | .560 | .708 | .002 |
| SE2 | <--- | AI自我效能 | .710 | .642 | .773 | .002 |
| SE3 | <--- | AI自我效能 | .692 | .619 | .760 | .002 |
| SE4 | <--- | AI自我效能 | .798 | .737 | .850 | .002 |
| SE5 | <--- | AI自我效能 | .794 | .736 | .847 | .002 |
| AB1 | <--- | 使用行为 | .761 | .700 | .818 | .002 |
| AB2 | <--- | 使用行为 | .749 | .682 | .813 | .002 |
| AB3 | <--- | 使用行为 | .811 | .752 | .866 | .002 |
| AB5 | <--- | 使用行为 | .712 | .651 | .773 | .002 |

##### Variances: (other - Structural weights)

| Parameter | | | Estimate | Lower | Upper | P |
| --- | --- | --- | --- | --- | --- | --- |
| AI威胁感知 |  |  | 2.216 | 1.753 | 2.684 | .002 |
| e21 |  |  | .987 | .712 | 1.303 | .002 |
| e23 |  |  | 1.293 | .920 | 1.642 | .002 |
| e22 |  |  | 1.226 | .906 | 1.561 | .002 |
| e1 |  |  | 1.139 | .882 | 1.386 | .002 |
| e2 |  |  | 1.208 | .944 | 1.515 | .002 |
| e3 |  |  | 1.354 | 1.096 | 1.621 | .002 |
| e4 |  |  | 1.512 | 1.190 | 1.833 | .002 |
| e5 |  |  | 1.339 | .991 | 1.693 | .002 |
| e6 |  |  | 1.698 | 1.350 | 2.066 | .002 |
| e8 |  |  | 1.574 | 1.213 | 1.943 | .002 |
| e9 |  |  | 1.193 | .898 | 1.443 | .002 |
| e10 |  |  | 1.850 | 1.463 | 2.218 | .002 |
| e11 |  |  | 1.943 | 1.575 | 2.262 | .002 |
| e12 |  |  | 1.716 | 1.355 | 2.048 | .002 |
| e13 |  |  | 1.703 | 1.347 | 2.026 | .002 |
| e14 |  |  | 1.214 | .924 | 1.497 | .002 |
| e15 |  |  | 1.132 | .845 | 1.416 | .002 |
| e16 |  |  | 1.414 | 1.109 | 1.694 | .002 |
| e17 |  |  | 1.456 | 1.128 | 1.784 | .002 |
| e18 |  |  | 1.154 | .827 | 1.473 | .002 |
| e20 |  |  | 1.773 | 1.425 | 2.098 | .002 |

##### Squared Multiple Correlations: (other - Structural weights)

| Parameter | | | Estimate | Lower | Upper | P |
| --- | --- | --- | --- | --- | --- | --- |
| AI自我效能 |  |  | .237 | .158 | .331 | .002 |
| 防御性动机 |  |  | .240 | .159 | .358 | .002 |
| 使用行为 |  |  | .368 | .275 | .479 | .002 |
| AB5 |  |  | .507 | .424 | .598 | .002 |
| AB3 |  |  | .658 | .565 | .749 | .002 |
| AB2 |  |  | .561 | .466 | .661 | .002 |
| AB1 |  |  | .579 | .490 | .669 | .002 |
| SE5 |  |  | .631 | .542 | .717 | .002 |
| SE4 |  |  | .636 | .544 | .723 | .002 |
| SE3 |  |  | .478 | .384 | .578 | .002 |
| SE2 |  |  | .505 | .413 | .598 | .002 |
| SE1 |  |  | .400 | .314 | .501 | .002 |
| DM5 |  |  | .468 | .368 | .576 | .002 |
| DM3 |  |  | .611 | .515 | .711 | .002 |
| DM2 |  |  | .532 | .435 | .634 | .002 |
| DM1 |  |  | .500 | .400 | .598 | .002 |
| AA5 |  |  | .593 | .497 | .691 | .002 |
| AA4 |  |  | .553 | .458 | .645 | .002 |
| AA3 |  |  | .581 | .497 | .666 | .002 |
| AA2 |  |  | .625 | .533 | .707 | .002 |
| AA1 |  |  | .661 | .574 | .742 | .002 |

##### User-defined estimands: (other - Structural weights)

| Parameter | | | Estimate | Lower | Upper | P |
| --- | --- | --- | --- | --- | --- | --- |
| diff1 |  |  | .000 | .000 | .000 | ... |
| diff2 |  |  | .027 | -.127 | .175 | .744 |
| diff3 |  |  | .094 | -.091 | .285 | .354 |
| diff4 |  |  | .030 | -.125 | .193 | .726 |
| diff5 |  |  | -.298 | -.489 | -.123 | .002 |

##### Bias-corrected percentile method (other - Structural weights)

##### 95% confidence intervals (bias-corrected percentile method)

##### Scalar Estimates (other - Structural weights)

##### Regression Weights: (other - Structural weights)

| Parameter | | | Estimate | Lower | Upper | P |
| --- | --- | --- | --- | --- | --- | --- |
| AI自我效能 | <--- | AI威胁感知 | .372 | .273 | .466 | .003 |
| 防御性动机 | <--- | AI威胁感知 | .399 | .275 | .522 | .003 |
| 防御性动机 | <--- | AI自我效能 | .074 | -.079 | .226 | .364 |
| 使用行为 | <--- | 防御性动机 | .402 | .284 | .523 | .002 |
| 使用行为 | <--- | AI自我效能 | .466 | .332 | .617 | .002 |
| AA1 | <--- | AI威胁感知 | 1.000 | 1.000 | 1.000 | ... |
| AA2 | <--- | AI威胁感知 | .953 | .867 | 1.056 | .001 |
| AA3 | <--- | AI威胁感知 | .921 | .829 | 1.013 | .002 |
| AA4 | <--- | AI威胁感知 | .919 | .833 | 1.030 | .002 |
| AA5 | <--- | AI威胁感知 | .939 | .839 | 1.048 | .002 |
| DM1 | <--- | 防御性动机 | 1.000 | 1.000 | 1.000 | ... |
| DM2 | <--- | 防御性动机 | 1.026 | .884 | 1.166 | .003 |
| DM3 | <--- | 防御性动机 | 1.050 | .903 | 1.205 | .003 |
| DM5 | <--- | 防御性动机 | .978 | .836 | 1.134 | .002 |
| SE1 | <--- | AI自我效能 | 1.000 | 1.000 | 1.000 | ... |
| SE2 | <--- | AI自我效能 | 1.163 | 1.005 | 1.366 | .001 |
| SE3 | <--- | AI自我效能 | 1.099 | .951 | 1.293 | .001 |
| SE4 | <--- | AI自我效能 | 1.281 | 1.120 | 1.499 | .001 |
| SE5 | <--- | AI自我效能 | 1.223 | 1.066 | 1.457 | .001 |
| AB1 | <--- | 使用行为 | 1.000 | 1.000 | 1.000 | ... |
| AB2 | <--- | 使用行为 | .978 | .870 | 1.116 | .002 |
| AB3 | <--- | 使用行为 | 1.069 | .956 | 1.202 | .002 |
| AB5 | <--- | 使用行为 | .970 | .858 | 1.128 | .001 |

##### Standardized Regression Weights: (other - Structural weights)

| Parameter | | | Estimate | Lower | Upper | P |
| --- | --- | --- | --- | --- | --- | --- |
| AI自我效能 | <--- | AI威胁感知 | .487 | .393 | .569 | .003 |
| 防御性动机 | <--- | AI威胁感知 | .455 | .316 | .571 | .004 |
| 防御性动机 | <--- | AI自我效能 | .064 | -.072 | .195 | .380 |
| 使用行为 | <--- | 防御性动机 | .376 | .264 | .482 | .002 |
| 使用行为 | <--- | AI自我效能 | .380 | .274 | .475 | .002 |
| AA1 | <--- | AI威胁感知 | .813 | .754 | .859 | .003 |
| AA2 | <--- | AI威胁感知 | .791 | .732 | .845 | .002 |
| AA3 | <--- | AI威胁感知 | .762 | .704 | .815 | .002 |
| AA4 | <--- | AI威胁感知 | .744 | .675 | .803 | .002 |
| AA5 | <--- | AI威胁感知 | .770 | .699 | .826 | .003 |
| DM1 | <--- | 防御性动机 | .707 | .636 | .778 | .001 |
| DM2 | <--- | 防御性动机 | .730 | .658 | .794 | .002 |
| DM3 | <--- | 防御性动机 | .782 | .709 | .836 | .004 |
| DM5 | <--- | 防御性动机 | .684 | .604 | .753 | .003 |
| SE1 | <--- | AI自我效能 | .632 | .554 | .703 | .003 |
| SE2 | <--- | AI自我效能 | .710 | .643 | .774 | .002 |
| SE3 | <--- | AI自我效能 | .692 | .610 | .753 | .004 |
| SE4 | <--- | AI自我效能 | .798 | .740 | .851 | .002 |
| SE5 | <--- | AI自我效能 | .794 | .733 | .845 | .003 |
| AB1 | <--- | 使用行为 | .761 | .686 | .812 | .004 |
| AB2 | <--- | 使用行为 | .749 | .678 | .809 | .003 |
| AB3 | <--- | 使用行为 | .811 | .752 | .865 | .002 |
| AB5 | <--- | 使用行为 | .712 | .648 | .771 | .002 |

##### Variances: (other - Structural weights)

| Parameter | | | Estimate | Lower | Upper | P |
| --- | --- | --- | --- | --- | --- | --- |
| AI威胁感知 |  |  | 2.216 | 1.749 | 2.680 | .002 |
| e21 |  |  | .987 | .715 | 1.311 | .002 |
| e23 |  |  | 1.293 | .961 | 1.733 | .001 |
| e22 |  |  | 1.226 | .937 | 1.605 | .001 |
| e1 |  |  | 1.139 | .896 | 1.412 | .001 |
| e2 |  |  | 1.208 | .940 | 1.511 | .002 |
| e3 |  |  | 1.354 | 1.117 | 1.643 | .001 |
| e4 |  |  | 1.512 | 1.221 | 1.885 | .001 |
| e5 |  |  | 1.339 | 1.036 | 1.734 | .001 |
| e6 |  |  | 1.698 | 1.365 | 2.078 | .002 |
| e8 |  |  | 1.574 | 1.232 | 1.996 | .001 |
| e9 |  |  | 1.193 | .952 | 1.489 | .001 |
| e10 |  |  | 1.850 | 1.491 | 2.244 | .001 |
| e11 |  |  | 1.943 | 1.615 | 2.302 | .001 |
| e12 |  |  | 1.716 | 1.386 | 2.083 | .001 |
| e13 |  |  | 1.703 | 1.391 | 2.107 | .001 |
| e14 |  |  | 1.214 | .941 | 1.509 | .001 |
| e15 |  |  | 1.132 | .895 | 1.492 | .001 |
| e16 |  |  | 1.414 | 1.154 | 1.760 | .001 |
| e17 |  |  | 1.456 | 1.153 | 1.799 | .001 |
| e18 |  |  | 1.154 | .855 | 1.502 | .001 |
| e20 |  |  | 1.773 | 1.443 | 2.107 | .001 |

##### Squared Multiple Correlations: (other - Structural weights)

| Parameter | | | Estimate | Lower | Upper | P |
| --- | --- | --- | --- | --- | --- | --- |
| AI自我效能 |  |  | .237 | .154 | .324 | .003 |
| 防御性动机 |  |  | .240 | .142 | .338 | .006 |
| 使用行为 |  |  | .368 | .268 | .475 | .003 |
| AB5 |  |  | .507 | .420 | .595 | .002 |
| AB3 |  |  | .658 | .565 | .749 | .002 |
| AB2 |  |  | .561 | .460 | .655 | .003 |
| AB1 |  |  | .579 | .471 | .660 | .004 |
| SE5 |  |  | .631 | .538 | .714 | .003 |
| SE4 |  |  | .636 | .548 | .725 | .002 |
| SE3 |  |  | .478 | .372 | .567 | .004 |
| SE2 |  |  | .505 | .414 | .600 | .002 |
| SE1 |  |  | .400 | .307 | .494 | .003 |
| DM5 |  |  | .468 | .364 | .568 | .003 |
| DM3 |  |  | .611 | .503 | .699 | .004 |
| DM2 |  |  | .532 | .432 | .631 | .002 |
| DM1 |  |  | .500 | .404 | .605 | .001 |
| AA5 |  |  | .593 | .489 | .682 | .003 |
| AA4 |  |  | .553 | .456 | .644 | .002 |
| AA3 |  |  | .581 | .496 | .664 | .002 |
| AA2 |  |  | .625 | .536 | .715 | .002 |
| AA1 |  |  | .661 | .569 | .738 | .003 |

##### User-defined estimands: (other - Structural weights)

| Parameter | | | Estimate | Lower | Upper | P |
| --- | --- | --- | --- | --- | --- | --- |
| diff1 |  |  | .000 | .000 | .000 | ... |
| diff2 |  |  | .027 | -.125 | .175 | .729 |
| diff3 |  |  | .094 | -.080 | .308 | .275 |
| diff4 |  |  | .030 | -.121 | .197 | .696 |
| diff5 |  |  | -.298 | -.489 | -.123 | .002 |

##### Minimization History (Structural weights)

| Iteration |  | Negative eigenvalues | Condition # | Smallest eigenvalue | Diameter | F | NTries | Ratio |
| --- | --- | --- | --- | --- | --- | --- | --- | --- |
| 0 | e | 8 |  | -.966 | 9999.000 | 3457.735 | 0 | 9999.000 |
| 1 | e | 2 |  | -.040 | 3.387 | 760.896 | 19 | .313 |
| 2 | e | 0 | 40.032 |  | 1.841 | 353.517 | 6 | .712 |
| 3 | e | 0 | 24.540 |  | 1.179 | 302.810 | 1 | .702 |
| 4 | e | 0 | 23.356 |  | .266 | 278.300 | 1 | 1.104 |
| 5 | e | 0 | 25.630 |  | .104 | 276.740 | 1 | 1.111 |
| 6 | e | 0 | 26.186 |  | .026 | 276.671 | 1 | 1.042 |
| 7 | e | 0 | 26.180 |  | .002 | 276.671 | 1 | 1.003 |
| 8 | e | 0 | 26.180 |  | .000 | 276.671 | 1 | 1.000 |

##### Bootstrap (Structural weights)

##### Summary of Bootstrap Iterations (Structural weights)

##### (Structural weights)

| Iterations | Method 0 | Method 1 | Method 2 |
| --- | --- | --- | --- |
| 1 | 0 | 0 | 0 |
| 2 | 0 | 0 | 0 |
| 3 | 0 | 0 | 0 |
| 4 | 0 | 0 | 0 |
| 5 | 0 | 1 | 0 |
| 6 | 0 | 103 | 0 |
| 7 | 0 | 390 | 0 |
| 8 | 0 | 308 | 0 |
| 9 | 0 | 147 | 0 |
| 10 | 0 | 35 | 0 |
| 11 | 0 | 12 | 0 |
| 12 | 0 | 2 | 0 |
| 13 | 0 | 1 | 0 |
| 14 | 0 | 0 | 0 |
| 15 | 0 | 1 | 0 |
| 16 | 0 | 0 | 0 |
| 17 | 0 | 0 | 0 |
| 18 | 0 | 0 | 0 |
| 19 | 0 | 0 | 0 |
| Total | 0 | 1000 | 0 |

0 bootstrap samples were unused because of a singular covariance matrix.

0 bootstrap samples were unused because a solution was not found.

1000 usable bootstrap samples were obtained.

##### Bootstrap Distributions (Structural weights)

##### ML discrepancy (implied vs sample) (Structural weights)

|  |  |  |
| --- | --- | --- |
|  |  | \|-------------------- |
|  | 449.123 | \|\* |
|  | 470.793 | \|\*\* |
|  | 492.463 | \|\*\*\*\*\*\* |
|  | 514.133 | \|\*\*\*\*\*\*\*\* |
|  | 535.803 | \|\*\*\*\*\*\*\*\*\*\*\*\* |
|  | 557.472 | \|\*\*\*\*\*\*\*\*\*\*\*\*\*\*\*\*\*\*\* |
|  | 579.142 | \|\*\*\*\*\*\*\*\*\*\*\*\*\*\*\*\*\*\*\* |
| N = 1000 | 600.812 | \|\*\*\*\*\*\*\*\*\*\*\*\*\*\*\* |
| Mean = 575.390 | 622.482 | \|\*\*\*\*\*\*\*\*\*\* |
| S. e. = 1.523 | 644.152 | \|\*\*\*\*\*\*\* |
|  | 665.822 | \|\*\*\* |
|  | 687.491 | \|\*\* |
|  | 709.161 | \|\* |
|  | 730.831 | \|\* |
|  | 752.501 | \|\* |
|  |  | \|-------------------- |

##### ML discrepancy (implied vs pop) (Structural weights)

|  |  |  |
| --- | --- | --- |
|  |  | \|-------------------- |
|  | 327.488 | \|\* |
|  | 334.679 | \|\*\* |
|  | 341.871 | \|\*\*\*\* |
|  | 349.062 | \|\*\*\*\*\*\*\*\*\* |
|  | 356.253 | \|\*\*\*\*\*\*\*\*\*\*\*\*\*\*\*\*\* |
|  | 363.445 | \|\*\*\*\*\*\*\*\*\*\*\*\*\*\*\*\*\* |
|  | 370.636 | \|\*\*\*\*\*\*\*\*\*\*\*\*\*\*\*\*\*\*\*\* |
| N = 1000 | 377.827 | \|\*\*\*\*\*\*\*\*\*\*\*\*\*\*\* |
| Mean = 368.739 | 385.019 | \|\*\*\*\*\*\*\*\*\* |
| S. e. = .495 | 392.210 | \|\*\*\*\*\* |
|  | 399.401 | \|\*\*\*\* |
|  | 406.593 | \|\*\* |
|  | 413.784 | \|\* |
|  | 420.975 | \|\* |
|  | 428.167 | \|\* |
|  |  | \|-------------------- |

##### K-L overoptimism (unstabilized) (Structural weights)

|  |  |  |
| --- | --- | --- |
|  |  | \|-------------------- |
|  | -170.842 | \|\* |
|  | -116.269 | \|\* |
|  | -61.696 | \|\*\* |
|  | -7.124 | \|\*\*\*\*\* |
|  | 47.449 | \|\*\*\*\*\*\*\*\*\*\*\* |
|  | 102.022 | \|\*\*\*\*\*\*\*\*\*\*\*\*\*\*\* |
|  | 156.595 | \|\*\*\*\*\*\*\*\*\*\*\*\*\*\*\*\*\*\* |
| N = 1000 | 211.168 | \|\*\*\*\*\*\*\*\*\*\*\*\*\*\*\*\*\*\*\* |
| Mean = 185.921 | 265.740 | \|\*\*\*\*\*\*\*\*\*\*\*\*\*\* |
| S. e. = 3.643 | 320.313 | \|\*\*\*\*\*\*\*\*\*\*\* |
|  | 374.886 | \|\*\*\*\*\*\* |
|  | 429.459 | \|\*\* |
|  | 484.031 | \|\* |
|  | 538.604 | \|\* |
|  | 593.177 | \|\* |
|  |  | \|-------------------- |

##### K-L overoptimism (stabilized) (Structural weights)

|  |  |  |
| --- | --- | --- |
|  |  | \|-------------------- |
|  | 48.650 | \|\* |
|  | 69.075 | \|\* |
|  | 89.500 | \|\*\* |
|  | 109.926 | \|\*\*\*\*\* |
|  | 130.351 | \|\*\*\*\*\*\*\*\*\*\* |
|  | 150.776 | \|\*\*\*\*\*\*\*\*\*\*\*\*\*\*\*\* |
|  | 171.201 | \|\*\*\*\*\*\*\*\*\*\*\*\*\*\*\*\*\*\*\*\* |
| N = 1000 | 191.627 | \|\*\*\*\*\*\*\*\*\*\*\*\*\*\*\*\*\*\*\*\* |
| Mean = 180.630 | 212.052 | \|\*\*\*\*\*\*\*\*\*\*\*\*\*\*\*\* |
| S. e. = 1.271 | 232.477 | \|\*\*\*\*\*\*\*\* |
|  | 252.903 | \|\*\*\*\* |
|  | 273.328 | \|\*\* |
|  | 293.753 | \|\* |
|  | 314.178 | \| |
|  | 334.604 | \|\* |
|  |  | \|-------------------- |

##### Structural covariances (Structural covariances)

##### Notes for Model (Structural covariances)

##### Computation of degrees of freedom (Structural covariances)

|  |  |
| --- | --- |
| Number of distinct sample moments: | 342 |
| Number of distinct parameters to be estimated: | 62 |
| Degrees of freedom (342 - 62): | 280 |

##### Result (Structural covariances)

Minimum was achieved

Chi-square = 276.710

Degrees of freedom = 280

Probability level = .544

##### east (east - Structural covariances)

##### Estimates (east - Structural covariances)

##### Scalar Estimates (east - Structural covariances)

##### Maximum Likelihood Estimates

##### Regression Weights: (east - Structural covariances)

|  |  |  | Estimate | S.E. | C.R. | P | Label |
| --- | --- | --- | --- | --- | --- | --- | --- |
| AI自我效能 | <--- | AI威胁感知 | .372 | .049 | 7.571 | \*\*\* | H1a |
| 防御性动机 | <--- | AI威胁感知 | .399 | .062 | 6.475 | \*\*\* | H2a |
| 防御性动机 | <--- | AI自我效能 | .074 | .074 | .996 | .319 | H5a |
| 使用行为 | <--- | 防御性动机 | .402 | .064 | 6.252 | \*\*\* | H4a |
| 使用行为 | <--- | AI自我效能 | .466 | .074 | 6.314 | \*\*\* | H3a |
| AA1 | <--- | AI威胁感知 | 1.000 |  |  |  |  |
| AA2 | <--- | AI威胁感知 | .954 | .057 | 16.832 | \*\*\* | a1\_1 |
| AA3 | <--- | AI威胁感知 | .921 | .058 | 15.920 | \*\*\* | a2\_1 |
| AA4 | <--- | AI威胁感知 | .919 | .058 | 15.986 | \*\*\* | a3\_1 |
| AA5 | <--- | AI威胁感知 | .939 | .056 | 16.739 | \*\*\* | a4\_1 |
| DM1 | <--- | 防御性动机 | 1.000 |  |  |  |  |
| DM2 | <--- | 防御性动机 | 1.026 | .082 | 12.534 | \*\*\* | a5\_1 |
| DM3 | <--- | 防御性动机 | 1.050 | .082 | 12.780 | \*\*\* | a6\_1 |
| DM5 | <--- | 防御性动机 | .978 | .081 | 12.072 | \*\*\* | a7\_1 |
| SE1 | <--- | AI自我效能 | 1.000 |  |  |  |  |
| SE2 | <--- | AI自我效能 | 1.163 | .101 | 11.500 | \*\*\* | a8\_1 |
| SE3 | <--- | AI自我效能 | 1.099 | .097 | 11.302 | \*\*\* | a9\_1 |
| SE4 | <--- | AI自我效能 | 1.281 | .103 | 12.497 | \*\*\* | a10\_1 |
| SE5 | <--- | AI自我效能 | 1.223 | .099 | 12.352 | \*\*\* | a11\_1 |
| AB1 | <--- | 使用行为 | 1.000 |  |  |  |  |
| AB2 | <--- | 使用行为 | .978 | .071 | 13.768 | \*\*\* | a12\_1 |
| AB3 | <--- | 使用行为 | 1.069 | .071 | 14.969 | \*\*\* | a13\_1 |
| AB5 | <--- | 使用行为 | .970 | .071 | 13.625 | \*\*\* | a14\_1 |

##### Standardized Regression Weights: (east - Structural covariances)

|  |  |  | Estimate |
| --- | --- | --- | --- |
| AI自我效能 | <--- | AI威胁感知 | .474 |
| 防御性动机 | <--- | AI威胁感知 | .455 |
| 防御性动机 | <--- | AI自我效能 | .066 |
| 使用行为 | <--- | 防御性动机 | .386 |
| 使用行为 | <--- | AI自我效能 | .400 |
| AA1 | <--- | AI威胁感知 | .830 |
| AA2 | <--- | AI威胁感知 | .777 |
| AA3 | <--- | AI威胁感知 | .736 |
| AA4 | <--- | AI威胁感知 | .767 |
| AA5 | <--- | AI威胁感知 | .796 |
| DM1 | <--- | 防御性动机 | .708 |
| DM2 | <--- | 防御性动机 | .758 |
| DM3 | <--- | 防御性动机 | .734 |
| DM5 | <--- | 防御性动机 | .740 |
| SE1 | <--- | AI自我效能 | .644 |
| SE2 | <--- | AI自我效能 | .722 |
| SE3 | <--- | AI自我效能 | .709 |
| SE4 | <--- | AI自我效能 | .814 |
| SE5 | <--- | AI自我效能 | .785 |
| AB1 | <--- | 使用行为 | .752 |
| AB2 | <--- | 使用行为 | .721 |
| AB3 | <--- | 使用行为 | .806 |
| AB5 | <--- | 使用行为 | .754 |

##### Variances: (east - Structural covariances)

|  |  |  | Estimate | S.E. | C.R. | P | Label |
| --- | --- | --- | --- | --- | --- | --- | --- |
| AI威胁感知 |  |  | 2.187 | .232 | 9.413 | \*\*\* | vvv1\_1 |
| e21 |  |  | 1.045 | .197 | 5.295 | \*\*\* | vv1\_1 |
| e23 |  |  | 1.279 | .232 | 5.504 | \*\*\* | vv3\_1 |
| e22 |  |  | 1.103 | .199 | 5.542 | \*\*\* | vv2\_1 |
| e1 |  |  | .987 | .149 | 6.627 | \*\*\* | v1\_1 |
| e2 |  |  | 1.307 | .178 | 7.332 | \*\*\* | v2\_1 |
| e3 |  |  | 1.572 | .205 | 7.677 | \*\*\* | v3\_1 |
| e4 |  |  | 1.291 | .174 | 7.416 | \*\*\* | v4\_1 |
| e5 |  |  | 1.115 | .157 | 7.113 | \*\*\* | v5\_1 |
| e6 |  |  | 1.677 | .231 | 7.250 | \*\*\* | v6\_1 |
| e8 |  |  | 1.310 | .196 | 6.676 | \*\*\* | v7\_1 |
| e9 |  |  | 1.585 | .227 | 6.991 | \*\*\* | v8\_1 |
| e10 |  |  | 1.330 | .193 | 6.895 | \*\*\* | v9\_1 |
| e11 |  |  | 1.904 | .239 | 7.979 | \*\*\* | v10\_1 |
| e12 |  |  | 1.676 | .223 | 7.509 | \*\*\* | v11\_1 |
| e13 |  |  | 1.610 | .212 | 7.603 | \*\*\* | v12\_1 |
| e14 |  |  | 1.123 | .175 | 6.408 | \*\*\* | v13\_1 |
| e15 |  |  | 1.252 | .183 | 6.859 | \*\*\* | v14\_1 |
| e16 |  |  | 1.406 | .199 | 7.054 | \*\*\* | v15\_1 |
| e17 |  |  | 1.618 | .220 | 7.367 | \*\*\* | v16\_1 |
| e18 |  |  | 1.123 | .179 | 6.272 | \*\*\* | v17\_1 |
| e20 |  |  | 1.302 | .186 | 7.009 | \*\*\* | v18\_1 |

##### Squared Multiple Correlations: (east - Structural covariances)

|  |  |  | Estimate |
| --- | --- | --- | --- |
| AI自我效能 |  |  | .225 |
| 防御性动机 |  |  | .240 |
| 使用行为 |  |  | .396 |
| AB5 |  |  | .569 |
| AB3 |  |  | .650 |
| AB2 |  |  | .519 |
| AB1 |  |  | .565 |
| SE5 |  |  | .617 |
| SE4 |  |  | .663 |
| SE3 |  |  | .503 |
| SE2 |  |  | .521 |
| SE1 |  |  | .415 |
| DM5 |  |  | .548 |
| DM3 |  |  | .539 |
| DM2 |  |  | .575 |
| DM1 |  |  | .501 |
| AA5 |  |  | .634 |
| AA4 |  |  | .589 |
| AA3 |  |  | .541 |
| AA2 |  |  | .603 |
| AA1 |  |  | .689 |

##### User-defined estimands: (east - Structural covariances)

|  |  |  |  |
| --- | --- | --- | --- |
| diff1 |  |  | .000 |
| diff2 |  |  | .026 |
| diff3 |  |  | .094 |
| diff4 |  |  | .029 |
| diff5 |  |  | -.299 |

##### Modification Indices (east - Structural covariances)

##### Covariances: (east - Structural covariances)

|  |  |  | M.I. | Par Change |
| --- | --- | --- | --- | --- |
| e21 | <--> | AI威胁感知 | 4.479 | .299 |
| e17 | <--> | e22 | 4.061 | -.268 |
| e13 | <--> | e16 | 7.127 | -.381 |
| e4 | <--> | e17 | 4.612 | .293 |
| e4 | <--> | e11 | 5.168 | .324 |
| e2 | <--> | e8 | 4.225 | -.265 |
| e1 | <--> | e15 | 5.491 | .267 |

##### Variances: (east - Structural covariances)

|  |  |  | M.I. | Par Change |
| --- | --- | --- | --- | --- |

##### Regression Weights: (east - Structural covariances)

|  |  |  | M.I. | Par Change |
| --- | --- | --- | --- | --- |
| AI自我效能 | <--- | AI威胁感知 | 4.479 | .137 |
| AB5 | <--- | SE3 | 4.120 | .114 |
| SE5 | <--- | AA1 | 4.973 | .126 |
| DM5 | <--- | AB5 | 5.449 | -.138 |
| DM5 | <--- | SE2 | 4.337 | -.114 |

##### Bootstrap (east - Structural covariances)

##### Bootstrap standard errors (east - Structural covariances)

##### Scalar Estimates (east - Structural covariances)

##### Regression Weights: (east - Structural covariances)

| Parameter | | | SE | SE-SE | Mean | Bias | SE-Bias |
| --- | --- | --- | --- | --- | --- | --- | --- |
| AI自我效能 | <--- | AI威胁感知 | .049 | .001 | .376 | .004 | .002 |
| 防御性动机 | <--- | AI威胁感知 | .063 | .001 | .402 | .003 | .002 |
| 防御性动机 | <--- | AI自我效能 | .079 | .002 | .076 | .003 | .003 |
| 使用行为 | <--- | 防御性动机 | .061 | .001 | .405 | .003 | .002 |
| 使用行为 | <--- | AI自我效能 | .074 | .002 | .464 | -.002 | .002 |
| AA1 | <--- | AI威胁感知 | .000 | .000 | 1.000 | .000 | .000 |
| AA2 | <--- | AI威胁感知 | .048 | .001 | .952 | -.001 | .002 |
| AA3 | <--- | AI威胁感知 | .046 | .001 | .922 | .002 | .001 |
| AA4 | <--- | AI威胁感知 | .051 | .001 | .919 | .000 | .002 |
| AA5 | <--- | AI威胁感知 | .052 | .001 | .940 | .002 | .002 |
| DM1 | <--- | 防御性动机 | .000 | .000 | 1.000 | .000 | .000 |
| DM2 | <--- | 防御性动机 | .073 | .002 | 1.031 | .005 | .002 |
| DM3 | <--- | 防御性动机 | .075 | .002 | 1.057 | .007 | .002 |
| DM5 | <--- | 防御性动机 | .078 | .002 | .983 | .004 | .002 |
| SE1 | <--- | AI自我效能 | .000 | .000 | 1.000 | .000 | .000 |
| SE2 | <--- | AI自我效能 | .093 | .002 | 1.158 | -.005 | .003 |
| SE3 | <--- | AI自我效能 | .088 | .002 | 1.101 | .002 | .003 |
| SE4 | <--- | AI自我效能 | .097 | .002 | 1.278 | -.004 | .003 |
| SE5 | <--- | AI自我效能 | .093 | .002 | 1.222 | -.001 | .003 |
| AB1 | <--- | 使用行为 | .000 | .000 | 1.000 | .000 | .000 |
| AB2 | <--- | 使用行为 | .059 | .001 | .981 | .002 | .002 |
| AB3 | <--- | 使用行为 | .063 | .001 | 1.071 | .003 | .002 |
| AB5 | <--- | 使用行为 | .065 | .001 | .972 | .002 | .002 |

##### Standardized Regression Weights: (east - Structural covariances)

| Parameter | | | SE | SE-SE | Mean | Bias | SE-Bias |
| --- | --- | --- | --- | --- | --- | --- | --- |
| AI自我效能 | <--- | AI威胁感知 | .055 | .001 | .477 | .003 | .002 |
| 防御性动机 | <--- | AI威胁感知 | .064 | .001 | .459 | .004 | .002 |
| 防御性动机 | <--- | AI自我效能 | .073 | .002 | .070 | .004 | .002 |
| 使用行为 | <--- | 防御性动机 | .056 | .001 | .387 | .001 | .002 |
| 使用行为 | <--- | AI自我效能 | .060 | .001 | .399 | -.001 | .002 |
| AA1 | <--- | AI威胁感知 | .028 | .001 | .830 | .000 | .001 |
| AA2 | <--- | AI威胁感知 | .026 | .001 | .777 | .000 | .001 |
| AA3 | <--- | AI威胁感知 | .030 | .001 | .738 | .002 | .001 |
| AA4 | <--- | AI威胁感知 | .029 | .001 | .768 | .001 | .001 |
| AA5 | <--- | AI威胁感知 | .026 | .001 | .798 | .002 | .001 |
| DM1 | <--- | 防御性动机 | .042 | .001 | .707 | .000 | .001 |
| DM2 | <--- | 防御性动机 | .039 | .001 | .759 | .001 | .001 |
| DM3 | <--- | 防御性动机 | .037 | .001 | .736 | .002 | .001 |
| DM5 | <--- | 防御性动机 | .038 | .001 | .740 | .000 | .001 |
| SE1 | <--- | AI自我效能 | .042 | .001 | .647 | .003 | .001 |
| SE2 | <--- | AI自我效能 | .045 | .001 | .721 | -.001 | .001 |
| SE3 | <--- | AI自我效能 | .037 | .001 | .712 | .002 | .001 |
| SE4 | <--- | AI自我效能 | .030 | .001 | .814 | .000 | .001 |
| SE5 | <--- | AI自我效能 | .034 | .001 | .787 | .002 | .001 |
| AB1 | <--- | 使用行为 | .038 | .001 | .753 | .001 | .001 |
| AB2 | <--- | 使用行为 | .038 | .001 | .723 | .003 | .001 |
| AB3 | <--- | 使用行为 | .031 | .001 | .808 | .001 | .001 |
| AB5 | <--- | 使用行为 | .038 | .001 | .756 | .001 | .001 |

##### Variances: (east - Structural covariances)

| Parameter | | | SE | SE-SE | Mean | Bias | SE-Bias |
| --- | --- | --- | --- | --- | --- | --- | --- |
| AI威胁感知 |  |  | .189 | .004 | 2.188 | .001 | .006 |
| e21 |  |  | .191 | .004 | 1.054 | .009 | .006 |
| e23 |  |  | .226 | .005 | 1.259 | -.020 | .007 |
| e22 |  |  | .186 | .004 | 1.096 | -.007 | .006 |
| e1 |  |  | .164 | .004 | .989 | .002 | .005 |
| e2 |  |  | .156 | .003 | 1.299 | -.008 | .005 |
| e3 |  |  | .190 | .004 | 1.554 | -.017 | .006 |
| e4 |  |  | .163 | .004 | 1.279 | -.013 | .005 |
| e5 |  |  | .132 | .003 | 1.096 | -.019 | .004 |
| e6 |  |  | .224 | .005 | 1.665 | -.012 | .007 |
| e8 |  |  | .187 | .004 | 1.297 | -.014 | .006 |
| e9 |  |  | .211 | .005 | 1.567 | -.019 | .007 |
| e10 |  |  | .170 | .004 | 1.318 | -.012 | .005 |
| e11 |  |  | .212 | .005 | 1.884 | -.020 | .007 |
| e12 |  |  | .268 | .006 | 1.669 | -.007 | .008 |
| e13 |  |  | .184 | .004 | 1.588 | -.022 | .006 |
| e14 |  |  | .172 | .004 | 1.114 | -.009 | .005 |
| e15 |  |  | .188 | .004 | 1.231 | -.021 | .006 |
| e16 |  |  | .196 | .004 | 1.390 | -.017 | .006 |
| e17 |  |  | .235 | .005 | 1.596 | -.022 | .007 |
| e18 |  |  | .163 | .004 | 1.109 | -.014 | .005 |
| e20 |  |  | .183 | .004 | 1.287 | -.015 | .006 |

##### Squared Multiple Correlations: (east - Structural covariances)

| Parameter | | | SE | SE-SE | Mean | Bias | SE-Bias |
| --- | --- | --- | --- | --- | --- | --- | --- |
| AI自我效能 |  |  | .053 | .001 | .231 | .006 | .002 |
| 防御性动机 |  |  | .054 | .001 | .253 | .013 | .002 |
| 使用行为 |  |  | .061 | .001 | .403 | .007 | .002 |
| AB5 |  |  | .058 | .001 | .572 | .003 | .002 |
| AB3 |  |  | .050 | .001 | .653 | .003 | .002 |
| AB2 |  |  | .054 | .001 | .525 | .005 | .002 |
| AB1 |  |  | .057 | .001 | .569 | .004 | .002 |
| SE5 |  |  | .053 | .001 | .621 | .004 | .002 |
| SE4 |  |  | .049 | .001 | .664 | .001 | .002 |
| SE3 |  |  | .052 | .001 | .508 | .005 | .002 |
| SE2 |  |  | .065 | .001 | .522 | .001 | .002 |
| SE1 |  |  | .054 | .001 | .420 | .006 | .002 |
| DM5 |  |  | .055 | .001 | .549 | .001 | .002 |
| DM3 |  |  | .054 | .001 | .543 | .004 | .002 |
| DM2 |  |  | .060 | .001 | .577 | .002 | .002 |
| DM1 |  |  | .060 | .001 | .502 | .001 | .002 |
| AA5 |  |  | .041 | .001 | .638 | .004 | .001 |
| AA4 |  |  | .045 | .001 | .591 | .002 | .001 |
| AA3 |  |  | .043 | .001 | .545 | .004 | .001 |
| AA2 |  |  | .040 | .001 | .604 | .001 | .001 |
| AA1 |  |  | .046 | .001 | .689 | .000 | .001 |

##### User-defined estimands: (east - Structural covariances)

| Parameter | | | SE | SE-SE | Mean | Bias | SE-Bias |
| --- | --- | --- | --- | --- | --- | --- | --- |
| diff1 |  |  | .000 | .000 | .000 | .000 | .000 |
| diff2 |  |  | .077 | .002 | .025 | -.001 | .002 |
| diff3 |  |  | .098 | .002 | .088 | -.006 | .003 |
| diff4 |  |  | .077 | .002 | .029 | -.001 | .002 |
| diff5 |  |  | .094 | .002 | -.300 | -.001 | .003 |

##### Bootstrap Confidence (east - Structural covariances)

##### Percentile method (east - Structural covariances)

##### 95% confidence intervals (percentile method)

##### Scalar Estimates (east - Structural covariances)

##### Regression Weights: (east - Structural covariances)

| Parameter | | | Estimate | Lower | Upper | P |
| --- | --- | --- | --- | --- | --- | --- |
| AI自我效能 | <--- | AI威胁感知 | .372 | .286 | .472 | .002 |
| 防御性动机 | <--- | AI威胁感知 | .399 | .285 | .533 | .002 |
| 防御性动机 | <--- | AI自我效能 | .074 | -.078 | .228 | .355 |
| 使用行为 | <--- | 防御性动机 | .402 | .291 | .525 | .002 |
| 使用行为 | <--- | AI自我效能 | .466 | .330 | .610 | .002 |
| AA1 | <--- | AI威胁感知 | 1.000 | 1.000 | 1.000 | ... |
| AA2 | <--- | AI威胁感知 | .954 | .864 | 1.048 | .002 |
| AA3 | <--- | AI威胁感知 | .921 | .834 | 1.015 | .002 |
| AA4 | <--- | AI威胁感知 | .919 | .826 | 1.026 | .002 |
| AA5 | <--- | AI威胁感知 | .939 | .841 | 1.053 | .002 |
| DM1 | <--- | 防御性动机 | 1.000 | 1.000 | 1.000 | ... |
| DM2 | <--- | 防御性动机 | 1.026 | .889 | 1.173 | .002 |
| DM3 | <--- | 防御性动机 | 1.050 | .915 | 1.211 | .002 |
| DM5 | <--- | 防御性动机 | .978 | .841 | 1.137 | .002 |
| SE1 | <--- | AI自我效能 | 1.000 | 1.000 | 1.000 | ... |
| SE2 | <--- | AI自我效能 | 1.163 | .981 | 1.356 | .002 |
| SE3 | <--- | AI自我效能 | 1.099 | .943 | 1.288 | .002 |
| SE4 | <--- | AI自我效能 | 1.281 | 1.110 | 1.482 | .002 |
| SE5 | <--- | AI自我效能 | 1.223 | 1.061 | 1.441 | .002 |
| AB1 | <--- | 使用行为 | 1.000 | 1.000 | 1.000 | ... |
| AB2 | <--- | 使用行为 | .978 | .866 | 1.108 | .002 |
| AB3 | <--- | 使用行为 | 1.069 | .959 | 1.203 | .002 |
| AB5 | <--- | 使用行为 | .970 | .855 | 1.117 | .002 |

##### Standardized Regression Weights: (east - Structural covariances)

| Parameter | | | Estimate | Lower | Upper | P |
| --- | --- | --- | --- | --- | --- | --- |
| AI自我效能 | <--- | AI威胁感知 | .474 | .366 | .582 | .002 |
| 防御性动机 | <--- | AI威胁感知 | .455 | .334 | .590 | .002 |
| 防御性动机 | <--- | AI自我效能 | .066 | -.068 | .217 | .355 |
| 使用行为 | <--- | 防御性动机 | .386 | .279 | .490 | .002 |
| 使用行为 | <--- | AI自我效能 | .400 | .277 | .514 | .002 |
| AA1 | <--- | AI威胁感知 | .830 | .773 | .883 | .002 |
| AA2 | <--- | AI威胁感知 | .777 | .725 | .825 | .002 |
| AA3 | <--- | AI威胁感知 | .736 | .675 | .794 | .002 |
| AA4 | <--- | AI威胁感知 | .767 | .711 | .823 | .002 |
| AA5 | <--- | AI威胁感知 | .796 | .745 | .847 | .002 |
| DM1 | <--- | 防御性动机 | .708 | .623 | .786 | .002 |
| DM2 | <--- | 防御性动机 | .758 | .683 | .838 | .002 |
| DM3 | <--- | 防御性动机 | .734 | .657 | .806 | .002 |
| DM5 | <--- | 防御性动机 | .740 | .659 | .810 | .002 |
| SE1 | <--- | AI自我效能 | .644 | .557 | .728 | .002 |
| SE2 | <--- | AI自我效能 | .722 | .630 | .808 | .002 |
| SE3 | <--- | AI自我效能 | .709 | .639 | .782 | .002 |
| SE4 | <--- | AI自我效能 | .814 | .749 | .870 | .002 |
| SE5 | <--- | AI自我效能 | .785 | .716 | .851 | .002 |
| AB1 | <--- | 使用行为 | .752 | .672 | .822 | .002 |
| AB2 | <--- | 使用行为 | .721 | .649 | .798 | .002 |
| AB3 | <--- | 使用行为 | .806 | .746 | .867 | .002 |
| AB5 | <--- | 使用行为 | .754 | .679 | .829 | .002 |

##### Variances: (east - Structural covariances)

| Parameter | | | Estimate | Lower | Upper | P |
| --- | --- | --- | --- | --- | --- | --- |
| AI威胁感知 |  |  | 2.187 | 1.797 | 2.553 | .002 |
| e21 |  |  | 1.045 | .728 | 1.464 | .002 |
| e23 |  |  | 1.279 | .849 | 1.741 | .002 |
| e22 |  |  | 1.103 | .739 | 1.459 | .002 |
| e1 |  |  | .987 | .672 | 1.314 | .002 |
| e2 |  |  | 1.307 | 1.010 | 1.620 | .002 |
| e3 |  |  | 1.572 | 1.212 | 1.965 | .002 |
| e4 |  |  | 1.291 | .963 | 1.598 | .002 |
| e5 |  |  | 1.115 | .841 | 1.351 | .002 |
| e6 |  |  | 1.677 | 1.246 | 2.104 | .002 |
| e8 |  |  | 1.310 | .906 | 1.664 | .002 |
| e9 |  |  | 1.585 | 1.186 | 2.011 | .002 |
| e10 |  |  | 1.330 | .981 | 1.672 | .002 |
| e11 |  |  | 1.904 | 1.490 | 2.336 | .002 |
| e12 |  |  | 1.676 | 1.155 | 2.211 | .002 |
| e13 |  |  | 1.610 | 1.225 | 1.956 | .002 |
| e14 |  |  | 1.123 | .798 | 1.472 | .002 |
| e15 |  |  | 1.252 | .882 | 1.617 | .002 |
| e16 |  |  | 1.406 | 1.021 | 1.798 | .002 |
| e17 |  |  | 1.618 | 1.150 | 2.089 | .002 |
| e18 |  |  | 1.123 | .798 | 1.434 | .002 |
| e20 |  |  | 1.302 | .938 | 1.653 | .002 |

##### Squared Multiple Correlations: (east - Structural covariances)

| Parameter | | | Estimate | Lower | Upper | P |
| --- | --- | --- | --- | --- | --- | --- |
| AI自我效能 |  |  | .225 | .134 | .338 | .002 |
| 防御性动机 |  |  | .240 | .156 | .376 | .002 |
| 使用行为 |  |  | .396 | .287 | .532 | .002 |
| AB5 |  |  | .569 | .461 | .688 | .002 |
| AB3 |  |  | .650 | .556 | .752 | .002 |
| AB2 |  |  | .519 | .422 | .637 | .002 |
| AB1 |  |  | .565 | .451 | .676 | .002 |
| SE5 |  |  | .617 | .513 | .723 | .002 |
| SE4 |  |  | .663 | .561 | .756 | .002 |
| SE3 |  |  | .503 | .408 | .611 | .002 |
| SE2 |  |  | .521 | .397 | .652 | .002 |
| SE1 |  |  | .415 | .311 | .530 | .002 |
| DM5 |  |  | .548 | .435 | .656 | .002 |
| DM3 |  |  | .539 | .431 | .649 | .002 |
| DM2 |  |  | .575 | .466 | .703 | .002 |
| DM1 |  |  | .501 | .388 | .618 | .002 |
| AA5 |  |  | .634 | .555 | .718 | .002 |
| AA4 |  |  | .589 | .506 | .677 | .002 |
| AA3 |  |  | .541 | .456 | .631 | .002 |
| AA2 |  |  | .603 | .526 | .681 | .002 |
| AA1 |  |  | .689 | .597 | .779 | .002 |

##### User-defined estimands: (east - Structural covariances)

| Parameter | | | Estimate | Lower | Upper | P |
| --- | --- | --- | --- | --- | --- | --- |
| diff1 |  |  | .000 | .000 | .000 | ... |
| diff2 |  |  | .026 | -.127 | .175 | .745 |
| diff3 |  |  | .094 | -.092 | .286 | .357 |
| diff4 |  |  | .029 | -.125 | .193 | .728 |
| diff5 |  |  | -.299 | -.489 | -.122 | .002 |

##### Bias-corrected percentile method (east - Structural covariances)

##### 95% confidence intervals (bias-corrected percentile method)

##### Scalar Estimates (east - Structural covariances)

##### Regression Weights: (east - Structural covariances)

| Parameter | | | Estimate | Lower | Upper | P |
| --- | --- | --- | --- | --- | --- | --- |
| AI自我效能 | <--- | AI威胁感知 | .372 | .274 | .465 | .003 |
| 防御性动机 | <--- | AI威胁感知 | .399 | .273 | .520 | .003 |
| 防御性动机 | <--- | AI自我效能 | .074 | -.078 | .227 | .358 |
| 使用行为 | <--- | 防御性动机 | .402 | .284 | .523 | .002 |
| 使用行为 | <--- | AI自我效能 | .466 | .332 | .617 | .002 |
| AA1 | <--- | AI威胁感知 | 1.000 | 1.000 | 1.000 | ... |
| AA2 | <--- | AI威胁感知 | .954 | .866 | 1.055 | .001 |
| AA3 | <--- | AI威胁感知 | .921 | .830 | 1.014 | .002 |
| AA4 | <--- | AI威胁感知 | .919 | .831 | 1.029 | .002 |
| AA5 | <--- | AI威胁感知 | .939 | .840 | 1.050 | .002 |
| DM1 | <--- | 防御性动机 | 1.000 | 1.000 | 1.000 | ... |
| DM2 | <--- | 防御性动机 | 1.026 | .884 | 1.166 | .003 |
| DM3 | <--- | 防御性动机 | 1.050 | .903 | 1.205 | .003 |
| DM5 | <--- | 防御性动机 | .978 | .837 | 1.134 | .002 |
| SE1 | <--- | AI自我效能 | 1.000 | 1.000 | 1.000 | ... |
| SE2 | <--- | AI自我效能 | 1.163 | 1.006 | 1.366 | .001 |
| SE3 | <--- | AI自我效能 | 1.099 | .951 | 1.293 | .001 |
| SE4 | <--- | AI自我效能 | 1.281 | 1.120 | 1.501 | .001 |
| SE5 | <--- | AI自我效能 | 1.223 | 1.066 | 1.457 | .001 |
| AB1 | <--- | 使用行为 | 1.000 | 1.000 | 1.000 | ... |
| AB2 | <--- | 使用行为 | .978 | .870 | 1.116 | .002 |
| AB3 | <--- | 使用行为 | 1.069 | .956 | 1.202 | .002 |
| AB5 | <--- | 使用行为 | .970 | .858 | 1.128 | .001 |

##### Standardized Regression Weights: (east - Structural covariances)

| Parameter | | | Estimate | Lower | Upper | P |
| --- | --- | --- | --- | --- | --- | --- |
| AI自我效能 | <--- | AI威胁感知 | .474 | .359 | .578 | .003 |
| 防御性动机 | <--- | AI威胁感知 | .455 | .323 | .582 | .003 |
| 防御性动机 | <--- | AI自我效能 | .066 | -.071 | .214 | .379 |
| 使用行为 | <--- | 防御性动机 | .386 | .269 | .483 | .003 |
| 使用行为 | <--- | AI自我效能 | .400 | .278 | .514 | .002 |
| AA1 | <--- | AI威胁感知 | .830 | .773 | .882 | .002 |
| AA2 | <--- | AI威胁感知 | .777 | .720 | .824 | .003 |
| AA3 | <--- | AI威胁感知 | .736 | .670 | .787 | .004 |
| AA4 | <--- | AI威胁感知 | .767 | .711 | .823 | .002 |
| AA5 | <--- | AI威胁感知 | .796 | .739 | .841 | .004 |
| DM1 | <--- | 防御性动机 | .708 | .624 | .788 | .002 |
| DM2 | <--- | 防御性动机 | .758 | .681 | .836 | .002 |
| DM3 | <--- | 防御性动机 | .734 | .651 | .798 | .004 |
| DM5 | <--- | 防御性动机 | .740 | .656 | .807 | .003 |
| SE1 | <--- | AI自我效能 | .644 | .552 | .719 | .004 |
| SE2 | <--- | AI自我效能 | .722 | .632 | .808 | .002 |
| SE3 | <--- | AI自我效能 | .709 | .632 | .773 | .003 |
| SE4 | <--- | AI自我效能 | .814 | .744 | .867 | .003 |
| SE5 | <--- | AI自我效能 | .785 | .712 | .845 | .004 |
| AB1 | <--- | 使用行为 | .752 | .666 | .818 | .003 |
| AB2 | <--- | 使用行为 | .721 | .642 | .793 | .003 |
| AB3 | <--- | 使用行为 | .806 | .741 | .864 | .003 |
| AB5 | <--- | 使用行为 | .754 | .673 | .825 | .003 |

##### Variances: (east - Structural covariances)

| Parameter | | | Estimate | Lower | Upper | P |
| --- | --- | --- | --- | --- | --- | --- |
| AI威胁感知 |  |  | 2.187 | 1.781 | 2.538 | .003 |
| e21 |  |  | 1.045 | .729 | 1.476 | .002 |
| e23 |  |  | 1.279 | .914 | 1.856 | .001 |
| e22 |  |  | 1.103 | .746 | 1.472 | .002 |
| e1 |  |  | .987 | .684 | 1.326 | .002 |
| e2 |  |  | 1.307 | 1.024 | 1.626 | .001 |
| e3 |  |  | 1.572 | 1.244 | 2.008 | .001 |
| e4 |  |  | 1.291 | 1.004 | 1.630 | .001 |
| e5 |  |  | 1.115 | .888 | 1.399 | .001 |
| e6 |  |  | 1.677 | 1.283 | 2.176 | .001 |
| e8 |  |  | 1.310 | .922 | 1.681 | .001 |
| e9 |  |  | 1.585 | 1.233 | 2.106 | .001 |
| e10 |  |  | 1.330 | 1.025 | 1.705 | .001 |
| e11 |  |  | 1.904 | 1.522 | 2.367 | .001 |
| e12 |  |  | 1.676 | 1.179 | 2.218 | .002 |
| e13 |  |  | 1.610 | 1.292 | 1.990 | .001 |
| e14 |  |  | 1.123 | .825 | 1.501 | .001 |
| e15 |  |  | 1.252 | .939 | 1.653 | .001 |
| e16 |  |  | 1.406 | 1.048 | 1.834 | .001 |
| e17 |  |  | 1.618 | 1.202 | 2.127 | .001 |
| e18 |  |  | 1.123 | .813 | 1.446 | .001 |
| e20 |  |  | 1.302 | .971 | 1.693 | .001 |

##### Squared Multiple Correlations: (east - Structural covariances)

| Parameter | | | Estimate | Lower | Upper | P |
| --- | --- | --- | --- | --- | --- | --- |
| AI自我效能 |  |  | .225 | .129 | .334 | .003 |
| 防御性动机 |  |  | .240 | .145 | .341 | .007 |
| 使用行为 |  |  | .396 | .277 | .518 | .004 |
| AB5 |  |  | .569 | .453 | .681 | .003 |
| AB3 |  |  | .650 | .549 | .747 | .003 |
| AB2 |  |  | .519 | .412 | .630 | .003 |
| AB1 |  |  | .565 | .443 | .670 | .003 |
| SE5 |  |  | .617 | .507 | .715 | .004 |
| SE4 |  |  | .663 | .553 | .751 | .003 |
| SE3 |  |  | .503 | .400 | .597 | .003 |
| SE2 |  |  | .521 | .399 | .654 | .002 |
| SE1 |  |  | .415 | .305 | .516 | .004 |
| DM5 |  |  | .548 | .430 | .651 | .003 |
| DM3 |  |  | .539 | .423 | .637 | .004 |
| DM2 |  |  | .575 | .464 | .699 | .002 |
| DM1 |  |  | .501 | .389 | .620 | .002 |
| AA5 |  |  | .634 | .546 | .708 | .004 |
| AA4 |  |  | .589 | .506 | .677 | .002 |
| AA3 |  |  | .541 | .448 | .620 | .004 |
| AA2 |  |  | .603 | .518 | .679 | .003 |
| AA1 |  |  | .689 | .597 | .778 | .002 |

##### User-defined estimands: (east - Structural covariances)

| Parameter | | | Estimate | Lower | Upper | P |
| --- | --- | --- | --- | --- | --- | --- |
| diff1 |  |  | .000 | .000 | .000 | ... |
| diff2 |  |  | .026 | -.126 | .175 | .730 |
| diff3 |  |  | .094 | -.081 | .309 | .279 |
| diff4 |  |  | .029 | -.122 | .195 | .710 |
| diff5 |  |  | -.299 | -.489 | -.122 | .002 |

##### other (other - Structural covariances)

##### Estimates (other - Structural covariances)

##### Scalar Estimates (other - Structural covariances)

##### Maximum Likelihood Estimates

##### Regression Weights: (other - Structural covariances)

|  |  |  | Estimate | S.E. | C.R. | P | Label |
| --- | --- | --- | --- | --- | --- | --- | --- |
| AI自我效能 | <--- | AI威胁感知 | .372 | .049 | 7.571 | \*\*\* | H1a |
| 防御性动机 | <--- | AI威胁感知 | .399 | .062 | 6.475 | \*\*\* | H2a |
| 防御性动机 | <--- | AI自我效能 | .074 | .074 | .996 | .319 | H5a |
| 使用行为 | <--- | 防御性动机 | .402 | .064 | 6.252 | \*\*\* | H4a |
| 使用行为 | <--- | AI自我效能 | .466 | .074 | 6.314 | \*\*\* | H3a |
| AA1 | <--- | AI威胁感知 | 1.000 |  |  |  |  |
| AA2 | <--- | AI威胁感知 | .954 | .057 | 16.832 | \*\*\* | a1\_1 |
| AA3 | <--- | AI威胁感知 | .921 | .058 | 15.920 | \*\*\* | a2\_1 |
| AA4 | <--- | AI威胁感知 | .919 | .058 | 15.986 | \*\*\* | a3\_1 |
| AA5 | <--- | AI威胁感知 | .939 | .056 | 16.739 | \*\*\* | a4\_1 |
| DM1 | <--- | 防御性动机 | 1.000 |  |  |  |  |
| DM2 | <--- | 防御性动机 | 1.026 | .082 | 12.534 | \*\*\* | a5\_1 |
| DM3 | <--- | 防御性动机 | 1.050 | .082 | 12.780 | \*\*\* | a6\_1 |
| DM5 | <--- | 防御性动机 | .978 | .081 | 12.072 | \*\*\* | a7\_1 |
| SE1 | <--- | AI自我效能 | 1.000 |  |  |  |  |
| SE2 | <--- | AI自我效能 | 1.163 | .101 | 11.500 | \*\*\* | a8\_1 |
| SE3 | <--- | AI自我效能 | 1.099 | .097 | 11.302 | \*\*\* | a9\_1 |
| SE4 | <--- | AI自我效能 | 1.281 | .103 | 12.497 | \*\*\* | a10\_1 |
| SE5 | <--- | AI自我效能 | 1.223 | .099 | 12.352 | \*\*\* | a11\_1 |
| AB1 | <--- | 使用行为 | 1.000 |  |  |  |  |
| AB2 | <--- | 使用行为 | .978 | .071 | 13.768 | \*\*\* | a12\_1 |
| AB3 | <--- | 使用行为 | 1.069 | .071 | 14.969 | \*\*\* | a13\_1 |
| AB5 | <--- | 使用行为 | .970 | .071 | 13.625 | \*\*\* | a14\_1 |

##### Standardized Regression Weights: (other - Structural covariances)

|  |  |  | Estimate |
| --- | --- | --- | --- |
| AI自我效能 | <--- | AI威胁感知 | .485 |
| 防御性动机 | <--- | AI威胁感知 | .453 |
| 防御性动机 | <--- | AI自我效能 | .064 |
| 使用行为 | <--- | 防御性动机 | .376 |
| 使用行为 | <--- | AI自我效能 | .380 |
| AA1 | <--- | AI威胁感知 | .811 |
| AA2 | <--- | AI威胁感知 | .789 |
| AA3 | <--- | AI威胁感知 | .760 |
| AA4 | <--- | AI威胁感知 | .742 |
| AA5 | <--- | AI威胁感知 | .768 |
| DM1 | <--- | 防御性动机 | .707 |
| DM2 | <--- | 防御性动机 | .729 |
| DM3 | <--- | 防御性动机 | .781 |
| DM5 | <--- | 防御性动机 | .684 |
| SE1 | <--- | AI自我效能 | .632 |
| SE2 | <--- | AI自我效能 | .710 |
| SE3 | <--- | AI自我效能 | .691 |
| SE4 | <--- | AI自我效能 | .797 |
| SE5 | <--- | AI自我效能 | .794 |
| AB1 | <--- | 使用行为 | .760 |
| AB2 | <--- | 使用行为 | .748 |
| AB3 | <--- | 使用行为 | .811 |
| AB5 | <--- | 使用行为 | .712 |

##### Variances: (other - Structural covariances)

|  |  |  | Estimate | S.E. | C.R. | P | Label |
| --- | --- | --- | --- | --- | --- | --- | --- |
| AI威胁感知 |  |  | 2.187 | .232 | 9.413 | \*\*\* | vvv1\_1 |
| e21 |  |  | .987 | .171 | 5.780 | \*\*\* | vv1\_2 |
| e23 |  |  | 1.293 | .209 | 6.189 | \*\*\* | vv3\_2 |
| e22 |  |  | 1.226 | .190 | 6.464 | \*\*\* | vv2\_2 |
| e1 |  |  | 1.139 | .139 | 8.214 | \*\*\* | v1\_2 |
| e2 |  |  | 1.208 | .141 | 8.562 | \*\*\* | v2\_2 |
| e3 |  |  | 1.355 | .152 | 8.923 | \*\*\* | v3\_2 |
| e4 |  |  | 1.513 | .166 | 9.128 | \*\*\* | v4\_2 |
| e5 |  |  | 1.339 | .151 | 8.844 | \*\*\* | v5\_2 |
| e6 |  |  | 1.698 | .196 | 8.646 | \*\*\* | v6\_2 |
| e8 |  |  | 1.573 | .188 | 8.366 | \*\*\* | v7\_2 |
| e9 |  |  | 1.193 | .160 | 7.444 | \*\*\* | v8\_2 |
| e10 |  |  | 1.850 | .207 | 8.918 | \*\*\* | v9\_2 |
| e11 |  |  | 1.943 | .201 | 9.649 | \*\*\* | v10\_2 |
| e12 |  |  | 1.716 | .189 | 9.081 | \*\*\* | v11\_2 |
| e13 |  |  | 1.703 | .184 | 9.247 | \*\*\* | v12\_2 |
| e14 |  |  | 1.214 | .154 | 7.898 | \*\*\* | v13\_2 |
| e15 |  |  | 1.132 | .142 | 7.955 | \*\*\* | v14\_2 |
| e16 |  |  | 1.414 | .170 | 8.325 | \*\*\* | v15\_2 |
| e17 |  |  | 1.456 | .171 | 8.491 | \*\*\* | v16\_2 |
| e18 |  |  | 1.154 | .157 | 7.371 | \*\*\* | v17\_2 |
| e20 |  |  | 1.773 | .198 | 8.946 | \*\*\* | v18\_2 |

##### Squared Multiple Correlations: (other - Structural covariances)

|  |  |  | Estimate |
| --- | --- | --- | --- |
| AI自我效能 |  |  | .235 |
| 防御性动机 |  |  | .238 |
| 使用行为 |  |  | .367 |
| AB5 |  |  | .507 |
| AB3 |  |  | .657 |
| AB2 |  |  | .560 |
| AB1 |  |  | .578 |
| SE5 |  |  | .630 |
| SE4 |  |  | .636 |
| SE3 |  |  | .478 |
| SE2 |  |  | .504 |
| SE1 |  |  | .399 |
| DM5 |  |  | .467 |
| DM3 |  |  | .610 |
| DM2 |  |  | .532 |
| DM1 |  |  | .500 |
| AA5 |  |  | .590 |
| AA4 |  |  | .550 |
| AA3 |  |  | .578 |
| AA2 |  |  | .622 |
| AA1 |  |  | .657 |

##### User-defined estimands: (other - Structural covariances)

|  |  |  |  |
| --- | --- | --- | --- |
| diff1 |  |  | .000 |
| diff2 |  |  | .026 |
| diff3 |  |  | .094 |
| diff4 |  |  | .029 |
| diff5 |  |  | -.299 |

##### Modification Indices (other - Structural covariances)

##### Covariances: (other - Structural covariances)

|  |  |  | M.I. | Par Change |
| --- | --- | --- | --- | --- |
| e20 | <--> | e23 | 5.525 | -.291 |
| e13 | <--> | AI威胁感知 | 5.691 | -.344 |
| e9 | <--> | e18 | 4.515 | .224 |
| e8 | <--> | e22 | 6.820 | .309 |
| e6 | <--> | e11 | 5.450 | -.322 |
| e5 | <--> | e14 | 12.417 | -.370 |
| e4 | <--> | e5 | 4.626 | .234 |
| e3 | <--> | e17 | 4.133 | .226 |
| e2 | <--> | e14 | 6.195 | .251 |
| e2 | <--> | e13 | 4.291 | -.232 |
| e1 | <--> | e3 | 5.139 | .222 |

##### Variances: (other - Structural covariances)

|  |  |  | M.I. | Par Change |
| --- | --- | --- | --- | --- |

##### Regression Weights: (other - Structural covariances)

|  |  |  | M.I. | Par Change |
| --- | --- | --- | --- | --- |
| AB2 | <--- | SE3 | 4.035 | -.098 |
| SE4 | <--- | AA2 | 4.179 | .095 |
| SE3 | <--- | AI威胁感知 | 5.691 | -.157 |
| SE3 | <--- | AA3 | 5.421 | -.120 |
| SE3 | <--- | AA2 | 8.567 | -.151 |
| SE1 | <--- | DM1 | 7.054 | -.139 |
| DM2 | <--- | AB2 | 4.166 | .103 |
| DM2 | <--- | AB1 | 4.432 | .106 |
| DM1 | <--- | SE1 | 5.379 | -.122 |
| AA5 | <--- | SE4 | 6.321 | -.115 |

##### Bootstrap (other - Structural covariances)

##### Bootstrap standard errors (other - Structural covariances)

##### Scalar Estimates (other - Structural covariances)

##### Regression Weights: (other - Structural covariances)

| Parameter | | | SE | SE-SE | Mean | Bias | SE-Bias |
| --- | --- | --- | --- | --- | --- | --- | --- |
| AI自我效能 | <--- | AI威胁感知 | .049 | .001 | .376 | .004 | .002 |
| 防御性动机 | <--- | AI威胁感知 | .063 | .001 | .402 | .003 | .002 |
| 防御性动机 | <--- | AI自我效能 | .079 | .002 | .076 | .003 | .003 |
| 使用行为 | <--- | 防御性动机 | .061 | .001 | .405 | .003 | .002 |
| 使用行为 | <--- | AI自我效能 | .074 | .002 | .464 | -.002 | .002 |
| AA1 | <--- | AI威胁感知 | .000 | .000 | 1.000 | .000 | .000 |
| AA2 | <--- | AI威胁感知 | .048 | .001 | .952 | -.001 | .002 |
| AA3 | <--- | AI威胁感知 | .046 | .001 | .922 | .002 | .001 |
| AA4 | <--- | AI威胁感知 | .051 | .001 | .919 | .000 | .002 |
| AA5 | <--- | AI威胁感知 | .052 | .001 | .940 | .002 | .002 |
| DM1 | <--- | 防御性动机 | .000 | .000 | 1.000 | .000 | .000 |
| DM2 | <--- | 防御性动机 | .073 | .002 | 1.031 | .005 | .002 |
| DM3 | <--- | 防御性动机 | .075 | .002 | 1.057 | .007 | .002 |
| DM5 | <--- | 防御性动机 | .078 | .002 | .983 | .004 | .002 |
| SE1 | <--- | AI自我效能 | .000 | .000 | 1.000 | .000 | .000 |
| SE2 | <--- | AI自我效能 | .093 | .002 | 1.158 | -.005 | .003 |
| SE3 | <--- | AI自我效能 | .088 | .002 | 1.101 | .002 | .003 |
| SE4 | <--- | AI自我效能 | .097 | .002 | 1.278 | -.004 | .003 |
| SE5 | <--- | AI自我效能 | .093 | .002 | 1.222 | -.001 | .003 |
| AB1 | <--- | 使用行为 | .000 | .000 | 1.000 | .000 | .000 |
| AB2 | <--- | 使用行为 | .059 | .001 | .981 | .002 | .002 |
| AB3 | <--- | 使用行为 | .063 | .001 | 1.071 | .003 | .002 |
| AB5 | <--- | 使用行为 | .065 | .001 | .972 | .002 | .002 |

##### Standardized Regression Weights: (other - Structural covariances)

| Parameter | | | SE | SE-SE | Mean | Bias | SE-Bias |
| --- | --- | --- | --- | --- | --- | --- | --- |
| AI自我效能 | <--- | AI威胁感知 | .045 | .001 | .487 | .002 | .001 |
| 防御性动机 | <--- | AI威胁感知 | .065 | .001 | .458 | .004 | .002 |
| 防御性动机 | <--- | AI自我效能 | .069 | .002 | .066 | .002 | .002 |
| 使用行为 | <--- | 防御性动机 | .055 | .001 | .377 | .001 | .002 |
| 使用行为 | <--- | AI自我效能 | .051 | .001 | .379 | -.002 | .002 |
| AA1 | <--- | AI威胁感知 | .024 | .001 | .812 | .001 | .001 |
| AA2 | <--- | AI威胁感知 | .027 | .001 | .787 | -.001 | .001 |
| AA3 | <--- | AI威胁感知 | .026 | .001 | .761 | .001 | .001 |
| AA4 | <--- | AI威胁感知 | .030 | .001 | .741 | .000 | .001 |
| AA5 | <--- | AI威胁感知 | .031 | .001 | .769 | .001 | .001 |
| DM1 | <--- | 防御性动机 | .036 | .001 | .705 | -.002 | .001 |
| DM2 | <--- | 防御性动机 | .034 | .001 | .729 | .000 | .001 |
| DM3 | <--- | 防御性动机 | .031 | .001 | .783 | .001 | .001 |
| DM5 | <--- | 防御性动机 | .039 | .001 | .683 | -.001 | .001 |
| SE1 | <--- | AI自我效能 | .038 | .001 | .634 | .003 | .001 |
| SE2 | <--- | AI自我效能 | .035 | .001 | .709 | -.001 | .001 |
| SE3 | <--- | AI自我效能 | .037 | .001 | .694 | .002 | .001 |
| SE4 | <--- | AI自我效能 | .028 | .001 | .796 | -.001 | .001 |
| SE5 | <--- | AI自我效能 | .029 | .001 | .795 | .001 | .001 |
| AB1 | <--- | 使用行为 | .030 | .001 | .762 | .002 | .001 |
| AB2 | <--- | 使用行为 | .034 | .001 | .749 | .001 | .001 |
| AB3 | <--- | 使用行为 | .029 | .001 | .811 | .001 | .001 |
| AB5 | <--- | 使用行为 | .031 | .001 | .713 | .001 | .001 |

##### Variances: (other - Structural covariances)

| Parameter | | | SE | SE-SE | Mean | Bias | SE-Bias |
| --- | --- | --- | --- | --- | --- | --- | --- |
| AI威胁感知 |  |  | .189 | .004 | 2.188 | .001 | .006 |
| e21 |  |  | .150 | .003 | .994 | .007 | .005 |
| e23 |  |  | .187 | .004 | 1.267 | -.025 | .006 |
| e22 |  |  | .167 | .004 | 1.219 | -.007 | .005 |
| e1 |  |  | .133 | .003 | 1.129 | -.010 | .004 |
| e2 |  |  | .145 | .003 | 1.210 | .002 | .005 |
| e3 |  |  | .134 | .003 | 1.342 | -.013 | .004 |
| e4 |  |  | .169 | .004 | 1.505 | -.007 | .005 |
| e5 |  |  | .178 | .004 | 1.326 | -.013 | .006 |
| e6 |  |  | .183 | .004 | 1.698 | .000 | .006 |
| e8 |  |  | .189 | .004 | 1.562 | -.012 | .006 |
| e9 |  |  | .136 | .003 | 1.175 | -.018 | .004 |
| e10 |  |  | .191 | .004 | 1.837 | -.013 | .006 |
| e11 |  |  | .168 | .004 | 1.923 | -.020 | .005 |
| e12 |  |  | .178 | .004 | 1.702 | -.014 | .006 |
| e13 |  |  | .176 | .004 | 1.682 | -.022 | .006 |
| e14 |  |  | .143 | .003 | 1.208 | -.006 | .005 |
| e15 |  |  | .142 | .003 | 1.120 | -.012 | .004 |
| e16 |  |  | .148 | .003 | 1.394 | -.020 | .005 |
| e17 |  |  | .168 | .004 | 1.446 | -.010 | .005 |
| e18 |  |  | .165 | .004 | 1.145 | -.009 | .005 |
| e20 |  |  | .174 | .004 | 1.759 | -.015 | .005 |

##### Squared Multiple Correlations: (other - Structural covariances)

| Parameter | | | SE | SE-SE | Mean | Bias | SE-Bias |
| --- | --- | --- | --- | --- | --- | --- | --- |
| AI自我效能 |  |  | .044 | .001 | .239 | .004 | .001 |
| 防御性动机 |  |  | .049 | .001 | .250 | .012 | .002 |
| 使用行为 |  |  | .051 | .001 | .372 | .005 | .002 |
| AB5 |  |  | .045 | .001 | .509 | .002 | .001 |
| AB3 |  |  | .047 | .001 | .659 | .002 | .001 |
| AB2 |  |  | .050 | .001 | .562 | .002 | .002 |
| AB1 |  |  | .046 | .001 | .581 | .003 | .001 |
| SE5 |  |  | .045 | .001 | .632 | .002 | .001 |
| SE4 |  |  | .045 | .001 | .635 | .000 | .001 |
| SE3 |  |  | .051 | .001 | .482 | .005 | .002 |
| SE2 |  |  | .049 | .001 | .504 | .000 | .002 |
| SE1 |  |  | .048 | .001 | .404 | .005 | .002 |
| DM5 |  |  | .054 | .001 | .468 | .001 | .002 |
| DM3 |  |  | .049 | .001 | .613 | .003 | .002 |
| DM2 |  |  | .049 | .001 | .533 | .001 | .002 |
| DM1 |  |  | .051 | .001 | .498 | -.002 | .002 |
| AA5 |  |  | .048 | .001 | .593 | .003 | .002 |
| AA4 |  |  | .044 | .001 | .551 | .001 | .001 |
| AA3 |  |  | .039 | .001 | .580 | .002 | .001 |
| AA2 |  |  | .042 | .001 | .621 | -.002 | .001 |
| AA1 |  |  | .039 | .001 | .659 | .002 | .001 |

##### User-defined estimands: (other - Structural covariances)

| Parameter | | | SE | SE-SE | Mean | Bias | SE-Bias |
| --- | --- | --- | --- | --- | --- | --- | --- |
| diff1 |  |  | .000 | .000 | .000 | .000 | .000 |
| diff2 |  |  | .077 | .002 | .025 | -.001 | .002 |
| diff3 |  |  | .098 | .002 | .088 | -.006 | .003 |
| diff4 |  |  | .077 | .002 | .029 | -.001 | .002 |
| diff5 |  |  | .094 | .002 | -.300 | -.001 | .003 |

##### Bootstrap Confidence (other - Structural covariances)

##### Percentile method (other - Structural covariances)

##### 95% confidence intervals (percentile method)

##### Scalar Estimates (other - Structural covariances)

##### Regression Weights: (other - Structural covariances)

| Parameter | | | Estimate | Lower | Upper | P |
| --- | --- | --- | --- | --- | --- | --- |
| AI自我效能 | <--- | AI威胁感知 | .372 | .286 | .472 | .002 |
| 防御性动机 | <--- | AI威胁感知 | .399 | .285 | .533 | .002 |
| 防御性动机 | <--- | AI自我效能 | .074 | -.078 | .228 | .355 |
| 使用行为 | <--- | 防御性动机 | .402 | .291 | .525 | .002 |
| 使用行为 | <--- | AI自我效能 | .466 | .330 | .610 | .002 |
| AA1 | <--- | AI威胁感知 | 1.000 | 1.000 | 1.000 | ... |
| AA2 | <--- | AI威胁感知 | .954 | .864 | 1.048 | .002 |
| AA3 | <--- | AI威胁感知 | .921 | .834 | 1.015 | .002 |
| AA4 | <--- | AI威胁感知 | .919 | .826 | 1.026 | .002 |
| AA5 | <--- | AI威胁感知 | .939 | .841 | 1.053 | .002 |
| DM1 | <--- | 防御性动机 | 1.000 | 1.000 | 1.000 | ... |
| DM2 | <--- | 防御性动机 | 1.026 | .889 | 1.173 | .002 |
| DM3 | <--- | 防御性动机 | 1.050 | .915 | 1.211 | .002 |
| DM5 | <--- | 防御性动机 | .978 | .841 | 1.137 | .002 |
| SE1 | <--- | AI自我效能 | 1.000 | 1.000 | 1.000 | ... |
| SE2 | <--- | AI自我效能 | 1.163 | .981 | 1.356 | .002 |
| SE3 | <--- | AI自我效能 | 1.099 | .943 | 1.288 | .002 |
| SE4 | <--- | AI自我效能 | 1.281 | 1.110 | 1.482 | .002 |
| SE5 | <--- | AI自我效能 | 1.223 | 1.061 | 1.441 | .002 |
| AB1 | <--- | 使用行为 | 1.000 | 1.000 | 1.000 | ... |
| AB2 | <--- | 使用行为 | .978 | .866 | 1.108 | .002 |
| AB3 | <--- | 使用行为 | 1.069 | .959 | 1.203 | .002 |
| AB5 | <--- | 使用行为 | .970 | .855 | 1.117 | .002 |

##### Standardized Regression Weights: (other - Structural covariances)

| Parameter | | | Estimate | Lower | Upper | P |
| --- | --- | --- | --- | --- | --- | --- |
| AI自我效能 | <--- | AI威胁感知 | .485 | .392 | .576 | .002 |
| 防御性动机 | <--- | AI威胁感知 | .453 | .336 | .583 | .002 |
| 防御性动机 | <--- | AI自我效能 | .064 | -.067 | .197 | .355 |
| 使用行为 | <--- | 防御性动机 | .376 | .266 | .483 | .002 |
| 使用行为 | <--- | AI自我效能 | .380 | .274 | .475 | .002 |
| AA1 | <--- | AI威胁感知 | .811 | .764 | .857 | .002 |
| AA2 | <--- | AI威胁感知 | .789 | .733 | .836 | .002 |
| AA3 | <--- | AI威胁感知 | .760 | .705 | .811 | .002 |
| AA4 | <--- | AI威胁感知 | .742 | .679 | .800 | .002 |
| AA5 | <--- | AI威胁感知 | .768 | .706 | .823 | .002 |
| DM1 | <--- | 防御性动机 | .707 | .631 | .773 | .002 |
| DM2 | <--- | 防御性动机 | .729 | .658 | .795 | .002 |
| DM3 | <--- | 防御性动机 | .781 | .717 | .842 | .002 |
| DM5 | <--- | 防御性动机 | .684 | .606 | .757 | .002 |
| SE1 | <--- | AI自我效能 | .632 | .561 | .708 | .002 |
| SE2 | <--- | AI自我效能 | .710 | .640 | .773 | .002 |
| SE3 | <--- | AI自我效能 | .691 | .620 | .759 | .002 |
| SE4 | <--- | AI自我效能 | .797 | .737 | .850 | .002 |
| SE5 | <--- | AI自我效能 | .794 | .736 | .846 | .002 |
| AB1 | <--- | 使用行为 | .760 | .699 | .818 | .002 |
| AB2 | <--- | 使用行为 | .748 | .682 | .813 | .002 |
| AB3 | <--- | 使用行为 | .811 | .752 | .866 | .002 |
| AB5 | <--- | 使用行为 | .712 | .653 | .772 | .002 |

##### Variances: (other - Structural covariances)

| Parameter | | | Estimate | Lower | Upper | P |
| --- | --- | --- | --- | --- | --- | --- |
| AI威胁感知 |  |  | 2.187 | 1.797 | 2.553 | .002 |
| e21 |  |  | .987 | .712 | 1.303 | .002 |
| e23 |  |  | 1.293 | .921 | 1.642 | .002 |
| e22 |  |  | 1.226 | .906 | 1.561 | .002 |
| e1 |  |  | 1.139 | .881 | 1.386 | .002 |
| e2 |  |  | 1.208 | .948 | 1.515 | .002 |
| e3 |  |  | 1.355 | 1.098 | 1.621 | .002 |
| e4 |  |  | 1.513 | 1.192 | 1.833 | .002 |
| e5 |  |  | 1.339 | .992 | 1.695 | .002 |
| e6 |  |  | 1.698 | 1.349 | 2.066 | .002 |
| e8 |  |  | 1.573 | 1.213 | 1.943 | .002 |
| e9 |  |  | 1.193 | .898 | 1.442 | .002 |
| e10 |  |  | 1.850 | 1.463 | 2.218 | .002 |
| e11 |  |  | 1.943 | 1.575 | 2.262 | .002 |
| e12 |  |  | 1.716 | 1.355 | 2.048 | .002 |
| e13 |  |  | 1.703 | 1.347 | 2.027 | .002 |
| e14 |  |  | 1.214 | .924 | 1.496 | .002 |
| e15 |  |  | 1.132 | .845 | 1.416 | .002 |
| e16 |  |  | 1.414 | 1.109 | 1.694 | .002 |
| e17 |  |  | 1.456 | 1.128 | 1.784 | .002 |
| e18 |  |  | 1.154 | .827 | 1.474 | .002 |
| e20 |  |  | 1.773 | 1.425 | 2.098 | .002 |

##### Squared Multiple Correlations: (other - Structural covariances)

| Parameter | | | Estimate | Lower | Upper | P |
| --- | --- | --- | --- | --- | --- | --- |
| AI自我效能 |  |  | .235 | .154 | .332 | .002 |
| 防御性动机 |  |  | .238 | .159 | .354 | .002 |
| 使用行为 |  |  | .367 | .276 | .476 | .002 |
| AB5 |  |  | .507 | .426 | .596 | .002 |
| AB3 |  |  | .657 | .565 | .750 | .002 |
| AB2 |  |  | .560 | .466 | .660 | .002 |
| AB1 |  |  | .578 | .489 | .669 | .002 |
| SE5 |  |  | .630 | .541 | .716 | .002 |
| SE4 |  |  | .636 | .544 | .723 | .002 |
| SE3 |  |  | .478 | .384 | .577 | .002 |
| SE2 |  |  | .504 | .410 | .597 | .002 |
| SE1 |  |  | .399 | .314 | .502 | .002 |
| DM5 |  |  | .467 | .367 | .573 | .002 |
| DM3 |  |  | .610 | .514 | .708 | .002 |
| DM2 |  |  | .532 | .433 | .632 | .002 |
| DM1 |  |  | .500 | .398 | .597 | .002 |
| AA5 |  |  | .590 | .498 | .678 | .002 |
| AA4 |  |  | .550 | .461 | .641 | .002 |
| AA3 |  |  | .578 | .498 | .657 | .002 |
| AA2 |  |  | .622 | .538 | .699 | .002 |
| AA1 |  |  | .657 | .583 | .734 | .002 |

##### User-defined estimands: (other - Structural covariances)

| Parameter | | | Estimate | Lower | Upper | P |
| --- | --- | --- | --- | --- | --- | --- |
| diff1 |  |  | .000 | .000 | .000 | ... |
| diff2 |  |  | .026 | -.127 | .175 | .745 |
| diff3 |  |  | .094 | -.092 | .286 | .357 |
| diff4 |  |  | .029 | -.125 | .193 | .728 |
| diff5 |  |  | -.299 | -.489 | -.122 | .002 |

##### Bias-corrected percentile method (other - Structural covariances)

##### 95% confidence intervals (bias-corrected percentile method)

##### Scalar Estimates (other - Structural covariances)

##### Regression Weights: (other - Structural covariances)

| Parameter | | | Estimate | Lower | Upper | P |
| --- | --- | --- | --- | --- | --- | --- |
| AI自我效能 | <--- | AI威胁感知 | .372 | .274 | .465 | .003 |
| 防御性动机 | <--- | AI威胁感知 | .399 | .273 | .520 | .003 |
| 防御性动机 | <--- | AI自我效能 | .074 | -.078 | .227 | .358 |
| 使用行为 | <--- | 防御性动机 | .402 | .284 | .523 | .002 |
| 使用行为 | <--- | AI自我效能 | .466 | .332 | .617 | .002 |
| AA1 | <--- | AI威胁感知 | 1.000 | 1.000 | 1.000 | ... |
| AA2 | <--- | AI威胁感知 | .954 | .866 | 1.055 | .001 |
| AA3 | <--- | AI威胁感知 | .921 | .830 | 1.014 | .002 |
| AA4 | <--- | AI威胁感知 | .919 | .831 | 1.029 | .002 |
| AA5 | <--- | AI威胁感知 | .939 | .840 | 1.050 | .002 |
| DM1 | <--- | 防御性动机 | 1.000 | 1.000 | 1.000 | ... |
| DM2 | <--- | 防御性动机 | 1.026 | .884 | 1.166 | .003 |
| DM3 | <--- | 防御性动机 | 1.050 | .903 | 1.205 | .003 |
| DM5 | <--- | 防御性动机 | .978 | .837 | 1.134 | .002 |
| SE1 | <--- | AI自我效能 | 1.000 | 1.000 | 1.000 | ... |
| SE2 | <--- | AI自我效能 | 1.163 | 1.006 | 1.366 | .001 |
| SE3 | <--- | AI自我效能 | 1.099 | .951 | 1.293 | .001 |
| SE4 | <--- | AI自我效能 | 1.281 | 1.120 | 1.501 | .001 |
| SE5 | <--- | AI自我效能 | 1.223 | 1.066 | 1.457 | .001 |
| AB1 | <--- | 使用行为 | 1.000 | 1.000 | 1.000 | ... |
| AB2 | <--- | 使用行为 | .978 | .870 | 1.116 | .002 |
| AB3 | <--- | 使用行为 | 1.069 | .956 | 1.202 | .002 |
| AB5 | <--- | 使用行为 | .970 | .858 | 1.128 | .001 |

##### Standardized Regression Weights: (other - Structural covariances)

| Parameter | | | Estimate | Lower | Upper | P |
| --- | --- | --- | --- | --- | --- | --- |
| AI自我效能 | <--- | AI威胁感知 | .485 | .388 | .570 | .003 |
| 防御性动机 | <--- | AI威胁感知 | .453 | .323 | .570 | .003 |
| 防御性动机 | <--- | AI自我效能 | .064 | -.069 | .196 | .373 |
| 使用行为 | <--- | 防御性动机 | .376 | .264 | .482 | .002 |
| 使用行为 | <--- | AI自我效能 | .380 | .274 | .474 | .002 |
| AA1 | <--- | AI威胁感知 | .811 | .760 | .854 | .003 |
| AA2 | <--- | AI威胁感知 | .789 | .734 | .836 | .002 |
| AA3 | <--- | AI威胁感知 | .760 | .702 | .808 | .003 |
| AA4 | <--- | AI威胁感知 | .742 | .678 | .799 | .002 |
| AA5 | <--- | AI威胁感知 | .768 | .699 | .819 | .004 |
| DM1 | <--- | 防御性动机 | .707 | .635 | .778 | .001 |
| DM2 | <--- | 防御性动机 | .729 | .657 | .794 | .003 |
| DM3 | <--- | 防御性动机 | .781 | .710 | .837 | .004 |
| DM5 | <--- | 防御性动机 | .684 | .603 | .755 | .003 |
| SE1 | <--- | AI自我效能 | .632 | .553 | .702 | .003 |
| SE2 | <--- | AI自我效能 | .710 | .643 | .774 | .002 |
| SE3 | <--- | AI自我效能 | .691 | .608 | .752 | .005 |
| SE4 | <--- | AI自我效能 | .797 | .740 | .851 | .001 |
| SE5 | <--- | AI自我效能 | .794 | .733 | .843 | .003 |
| AB1 | <--- | 使用行为 | .760 | .685 | .812 | .004 |
| AB2 | <--- | 使用行为 | .748 | .680 | .809 | .003 |
| AB3 | <--- | 使用行为 | .811 | .751 | .865 | .002 |
| AB5 | <--- | 使用行为 | .712 | .647 | .770 | .003 |

##### Variances: (other - Structural covariances)

| Parameter | | | Estimate | Lower | Upper | P |
| --- | --- | --- | --- | --- | --- | --- |
| AI威胁感知 |  |  | 2.187 | 1.781 | 2.538 | .003 |
| e21 |  |  | .987 | .715 | 1.312 | .002 |
| e23 |  |  | 1.293 | .962 | 1.735 | .001 |
| e22 |  |  | 1.226 | .937 | 1.605 | .001 |
| e1 |  |  | 1.139 | .897 | 1.411 | .001 |
| e2 |  |  | 1.208 | .942 | 1.511 | .002 |
| e3 |  |  | 1.355 | 1.118 | 1.641 | .001 |
| e4 |  |  | 1.513 | 1.223 | 1.883 | .001 |
| e5 |  |  | 1.339 | 1.035 | 1.735 | .001 |
| e6 |  |  | 1.698 | 1.365 | 2.078 | .002 |
| e8 |  |  | 1.573 | 1.232 | 1.996 | .001 |
| e9 |  |  | 1.193 | .951 | 1.489 | .001 |
| e10 |  |  | 1.850 | 1.490 | 2.244 | .001 |
| e11 |  |  | 1.943 | 1.616 | 2.302 | .001 |
| e12 |  |  | 1.716 | 1.386 | 2.083 | .001 |
| e13 |  |  | 1.703 | 1.391 | 2.107 | .001 |
| e14 |  |  | 1.214 | .941 | 1.509 | .001 |
| e15 |  |  | 1.132 | .895 | 1.492 | .001 |
| e16 |  |  | 1.414 | 1.154 | 1.760 | .001 |
| e17 |  |  | 1.456 | 1.153 | 1.799 | .001 |
| e18 |  |  | 1.154 | .855 | 1.502 | .001 |
| e20 |  |  | 1.773 | 1.443 | 2.107 | .001 |

##### Squared Multiple Correlations: (other - Structural covariances)

| Parameter | | | Estimate | Lower | Upper | P |
| --- | --- | --- | --- | --- | --- | --- |
| AI自我效能 |  |  | .235 | .151 | .325 | .003 |
| 防御性动机 |  |  | .238 | .144 | .338 | .005 |
| 使用行为 |  |  | .367 | .270 | .470 | .003 |
| AB5 |  |  | .507 | .419 | .593 | .003 |
| AB3 |  |  | .657 | .563 | .748 | .002 |
| AB2 |  |  | .560 | .462 | .654 | .003 |
| AB1 |  |  | .578 | .470 | .660 | .004 |
| SE5 |  |  | .630 | .538 | .711 | .003 |
| SE4 |  |  | .636 | .548 | .725 | .001 |
| SE3 |  |  | .478 | .369 | .565 | .005 |
| SE2 |  |  | .504 | .413 | .600 | .002 |
| SE1 |  |  | .399 | .305 | .493 | .003 |
| DM5 |  |  | .467 | .364 | .570 | .003 |
| DM3 |  |  | .610 | .504 | .700 | .004 |
| DM2 |  |  | .532 | .431 | .630 | .003 |
| DM1 |  |  | .500 | .403 | .605 | .001 |
| AA5 |  |  | .590 | .488 | .671 | .004 |
| AA4 |  |  | .550 | .460 | .638 | .002 |
| AA3 |  |  | .578 | .492 | .652 | .003 |
| AA2 |  |  | .622 | .539 | .699 | .002 |
| AA1 |  |  | .657 | .578 | .730 | .003 |

##### User-defined estimands: (other - Structural covariances)

| Parameter | | | Estimate | Lower | Upper | P |
| --- | --- | --- | --- | --- | --- | --- |
| diff1 |  |  | .000 | .000 | .000 | ... |
| diff2 |  |  | .026 | -.126 | .175 | .730 |
| diff3 |  |  | .094 | -.081 | .309 | .279 |
| diff4 |  |  | .029 | -.122 | .195 | .710 |
| diff5 |  |  | -.299 | -.489 | -.122 | .002 |

##### Minimization History (Structural covariances)

| Iteration |  | Negative eigenvalues | Condition # | Smallest eigenvalue | Diameter | F | NTries | Ratio |
| --- | --- | --- | --- | --- | --- | --- | --- | --- |
| 0 | e | 9 |  | -.974 | 9999.000 | 3449.109 | 0 | 9999.000 |
| 1 | e | 2 |  | -.094 | 3.552 | 899.406 | 19 | .268 |
| 2 | e | 0 | 41.146 |  | 1.930 | 383.395 | 6 | .695 |
| 3 | e | 0 | 19.142 |  | .839 | 301.698 | 2 | .000 |
| 4 | e | 0 | 21.687 |  | .399 | 277.768 | 1 | 1.089 |
| 5 | e | 0 | 25.579 |  | .124 | 276.718 | 1 | 1.051 |
| 6 | e | 0 | 26.087 |  | .013 | 276.710 | 1 | 1.007 |
| 7 | e | 0 | 26.096 |  | .000 | 276.710 | 1 | 1.000 |

##### Bootstrap (Structural covariances)

##### Summary of Bootstrap Iterations (Structural covariances)

##### (Structural covariances)

| Iterations | Method 0 | Method 1 | Method 2 |
| --- | --- | --- | --- |
| 1 | 0 | 0 | 0 |
| 2 | 0 | 0 | 0 |
| 3 | 0 | 0 | 0 |
| 4 | 0 | 0 | 0 |
| 5 | 0 | 1 | 0 |
| 6 | 0 | 106 | 0 |
| 7 | 0 | 397 | 0 |
| 8 | 0 | 304 | 0 |
| 9 | 0 | 145 | 0 |
| 10 | 0 | 32 | 0 |
| 11 | 0 | 11 | 0 |
| 12 | 0 | 2 | 0 |
| 13 | 0 | 1 | 0 |
| 14 | 0 | 1 | 0 |
| 15 | 0 | 0 | 0 |
| 16 | 0 | 0 | 0 |
| 17 | 0 | 0 | 0 |
| 18 | 0 | 0 | 0 |
| 19 | 0 | 0 | 0 |
| Total | 0 | 1000 | 0 |

0 bootstrap samples were unused because of a singular covariance matrix.

0 bootstrap samples were unused because a solution was not found.

1000 usable bootstrap samples were obtained.

##### Bootstrap Distributions (Structural covariances)

##### ML discrepancy (implied vs sample) (Structural covariances)

|  |  |  |
| --- | --- | --- |
|  |  | \|-------------------- |
|  | 449.136 | \|\* |
|  | 470.812 | \|\*\* |
|  | 492.488 | \|\*\*\*\*\*\* |
|  | 514.165 | \|\*\*\*\*\*\*\*\* |
|  | 535.841 | \|\*\*\*\*\*\*\*\*\*\*\*\* |
|  | 557.517 | \|\*\*\*\*\*\*\*\*\*\*\*\*\*\*\*\*\*\*\* |
|  | 579.193 | \|\*\*\*\*\*\*\*\*\*\*\*\*\*\*\*\*\*\*\* |
| N = 1000 | 600.869 | \|\*\*\*\*\*\*\*\*\*\*\*\*\*\*\* |
| Mean = 576.010 | 622.546 | \|\*\*\*\*\*\*\*\*\*\* |
| S. e. = 1.524 | 644.222 | \|\*\*\*\*\*\*\*\* |
|  | 665.898 | \|\*\*\* |
|  | 687.574 | \|\*\* |
|  | 709.250 | \|\* |
|  | 730.927 | \|\* |
|  | 752.603 | \|\* |
|  |  | \|-------------------- |

##### ML discrepancy (implied vs pop) (Structural covariances)

|  |  |  |
| --- | --- | --- |
|  |  | \|-------------------- |
|  | 327.418 | \|\* |
|  | 334.527 | \|\*\* |
|  | 341.635 | \|\*\*\*\* |
|  | 348.744 | \|\*\*\*\*\*\*\*\*\* |
|  | 355.853 | \|\*\*\*\*\*\*\*\*\*\*\*\*\*\*\*\*\* |
|  | 362.962 | \|\*\*\*\*\*\*\*\*\*\*\*\*\*\*\*\*\*\* |
|  | 370.071 | \|\*\*\*\*\*\*\*\*\*\*\*\*\*\*\*\*\*\*\*\* |
| N = 1000 | 377.180 | \|\*\*\*\*\*\*\*\*\*\*\*\*\*\* |
| Mean = 368.182 | 384.289 | \|\*\*\*\*\*\*\*\*\*\* |
| S. e. = .491 | 391.398 | \|\*\*\*\*\* |
|  | 398.507 | \|\*\*\*\* |
|  | 405.616 | \|\*\* |
|  | 412.725 | \|\* |
|  | 419.834 | \|\* |
|  | 426.943 | \|\* |
|  |  | \|-------------------- |

##### K-L overoptimism (unstabilized) (Structural covariances)

|  |  |  |
| --- | --- | --- |
|  |  | \|-------------------- |
|  | -172.921 | \|\* |
|  | -118.244 | \|\* |
|  | -63.567 | \|\*\* |
|  | -8.890 | \|\*\*\*\*\* |
|  | 45.787 | \|\*\*\*\*\*\*\*\*\*\* |
|  | 100.464 | \|\*\*\*\*\*\*\*\*\*\*\*\*\*\*\* |
|  | 155.141 | \|\*\*\*\*\*\*\*\*\*\*\*\*\*\*\*\*\*\*\* |
| N = 1000 | 209.817 | \|\*\*\*\*\*\*\*\*\*\*\*\*\*\*\*\*\*\*\* |
| Mean = 184.745 | 264.494 | \|\*\*\*\*\*\*\*\*\*\*\*\*\*\* |
| S. e. = 3.640 | 319.171 | \|\*\*\*\*\*\*\*\*\*\*\* |
|  | 373.848 | \|\*\*\*\*\*\* |
|  | 428.525 | \|\*\* |
|  | 483.202 | \|\* |
|  | 537.879 | \|\* |
|  | 592.555 | \|\* |
|  |  | \|-------------------- |

##### K-L overoptimism (stabilized) (Structural covariances)

|  |  |  |
| --- | --- | --- |
|  |  | \|-------------------- |
|  | 47.266 | \|\* |
|  | 67.744 | \|\* |
|  | 88.222 | \|\* |
|  | 108.700 | \|\*\*\*\*\* |
|  | 129.178 | \|\*\*\*\*\*\*\*\*\* |
|  | 149.656 | \|\*\*\*\*\*\*\*\*\*\*\*\*\*\*\* |
|  | 170.134 | \|\*\*\*\*\*\*\*\*\*\*\*\*\*\*\*\*\*\*\* |
| N = 1000 | 190.612 | \|\*\*\*\*\*\*\*\*\*\*\*\*\*\*\*\*\*\*\* |
| Mean = 179.454 | 211.090 | \|\*\*\*\*\*\*\*\*\*\*\*\*\*\* |
| S. e. = 1.266 | 231.568 | \|\*\*\*\*\*\*\*\* |
|  | 252.047 | \|\*\*\*\* |
|  | 272.525 | \|\*\* |
|  | 293.003 | \|\* |
|  | 313.481 | \| |
|  | 333.959 | \|\* |
|  |  | \|-------------------- |

##### Structural residuals (Structural residuals)

##### Notes for Model (Structural residuals)

##### Computation of degrees of freedom (Structural residuals)

|  |  |
| --- | --- |
| Number of distinct sample moments: | 342 |
| Number of distinct parameters to be estimated: | 59 |
| Degrees of freedom (342 - 59): | 283 |

##### Result (Structural residuals)

Minimum was achieved

Chi-square = 277.072

Degrees of freedom = 283

Probability level = .588

##### east (east - Structural residuals)

##### Estimates (east - Structural residuals)

##### Scalar Estimates (east - Structural residuals)

##### Maximum Likelihood Estimates

##### Regression Weights: (east - Structural residuals)

|  |  |  | Estimate | S.E. | C.R. | P | Label |
| --- | --- | --- | --- | --- | --- | --- | --- |
| AI自我效能 | <--- | AI威胁感知 | .375 | .049 | 7.609 | \*\*\* | H1a |
| 防御性动机 | <--- | AI威胁感知 | .399 | .062 | 6.469 | \*\*\* | H2a |
| 防御性动机 | <--- | AI自我效能 | .072 | .074 | .979 | .328 | H5a |
| 使用行为 | <--- | 防御性动机 | .404 | .064 | 6.272 | \*\*\* | H4a |
| 使用行为 | <--- | AI自我效能 | .463 | .074 | 6.284 | \*\*\* | H3a |
| AA1 | <--- | AI威胁感知 | 1.000 |  |  |  |  |
| AA2 | <--- | AI威胁感知 | .954 | .057 | 16.831 | \*\*\* | a1\_1 |
| AA3 | <--- | AI威胁感知 | .921 | .058 | 15.921 | \*\*\* | a2\_1 |
| AA4 | <--- | AI威胁感知 | .919 | .058 | 15.985 | \*\*\* | a3\_1 |
| AA5 | <--- | AI威胁感知 | .939 | .056 | 16.739 | \*\*\* | a4\_1 |
| DM1 | <--- | 防御性动机 | 1.000 |  |  |  |  |
| DM2 | <--- | 防御性动机 | 1.027 | .082 | 12.538 | \*\*\* | a5\_1 |
| DM3 | <--- | 防御性动机 | 1.050 | .082 | 12.780 | \*\*\* | a6\_1 |
| DM5 | <--- | 防御性动机 | .979 | .081 | 12.078 | \*\*\* | a7\_1 |
| SE1 | <--- | AI自我效能 | 1.000 |  |  |  |  |
| SE2 | <--- | AI自我效能 | 1.162 | .101 | 11.519 | \*\*\* | a8\_1 |
| SE3 | <--- | AI自我效能 | 1.099 | .097 | 11.323 | \*\*\* | a9\_1 |
| SE4 | <--- | AI自我效能 | 1.281 | .102 | 12.520 | \*\*\* | a10\_1 |
| SE5 | <--- | AI自我效能 | 1.222 | .099 | 12.377 | \*\*\* | a11\_1 |
| AB1 | <--- | 使用行为 | 1.000 |  |  |  |  |
| AB2 | <--- | 使用行为 | .976 | .071 | 13.755 | \*\*\* | a12\_1 |
| AB3 | <--- | 使用行为 | 1.068 | .071 | 14.990 | \*\*\* | a13\_1 |
| AB5 | <--- | 使用行为 | .969 | .071 | 13.653 | \*\*\* | a14\_1 |

##### Standardized Regression Weights: (east - Structural residuals)

|  |  |  | Estimate |
| --- | --- | --- | --- |
| AI自我效能 | <--- | AI威胁感知 | .483 |
| 防御性动机 | <--- | AI威胁感知 | .454 |
| 防御性动机 | <--- | AI自我效能 | .064 |
| 使用行为 | <--- | 防御性动机 | .382 |
| 使用行为 | <--- | AI自我效能 | .386 |
| AA1 | <--- | AI威胁感知 | .830 |
| AA2 | <--- | AI威胁感知 | .777 |
| AA3 | <--- | AI威胁感知 | .736 |
| AA4 | <--- | AI威胁感知 | .767 |
| AA5 | <--- | AI威胁感知 | .796 |
| DM1 | <--- | 防御性动机 | .708 |
| DM2 | <--- | 防御性动机 | .759 |
| DM3 | <--- | 防御性动机 | .735 |
| DM5 | <--- | 防御性动机 | .741 |
| SE1 | <--- | AI自我效能 | .640 |
| SE2 | <--- | AI自我效能 | .717 |
| SE3 | <--- | AI自我效能 | .705 |
| SE4 | <--- | AI自我效能 | .811 |
| SE5 | <--- | AI自我效能 | .782 |
| AB1 | <--- | 使用行为 | .759 |
| AB2 | <--- | 使用行为 | .726 |
| AB3 | <--- | 使用行为 | .812 |
| AB5 | <--- | 使用行为 | .760 |

##### Variances: (east - Structural residuals)

|  |  |  | Estimate | S.E. | C.R. | P | Label |
| --- | --- | --- | --- | --- | --- | --- | --- |
| AI威胁感知 |  |  | 2.187 | .232 | 9.413 | \*\*\* | vvv1\_1 |
| e21 |  |  | 1.010 | .157 | 6.428 | \*\*\* | vv1\_1 |
| e23 |  |  | 1.287 | .182 | 7.083 | \*\*\* | vv3\_1 |
| e22 |  |  | 1.179 | .156 | 7.557 | \*\*\* | vv2\_1 |
| e1 |  |  | .988 | .149 | 6.631 | \*\*\* | v1\_1 |
| e2 |  |  | 1.307 | .178 | 7.334 | \*\*\* | v2\_1 |
| e3 |  |  | 1.571 | .205 | 7.677 | \*\*\* | v3\_1 |
| e4 |  |  | 1.292 | .174 | 7.417 | \*\*\* | v4\_1 |
| e5 |  |  | 1.115 | .157 | 7.116 | \*\*\* | v5\_1 |
| e6 |  |  | 1.677 | .231 | 7.251 | \*\*\* | v6\_1 |
| e8 |  |  | 1.310 | .196 | 6.675 | \*\*\* | v7\_1 |
| e9 |  |  | 1.585 | .227 | 6.995 | \*\*\* | v8\_1 |
| e10 |  |  | 1.327 | .193 | 6.892 | \*\*\* | v9\_1 |
| e11 |  |  | 1.904 | .239 | 7.982 | \*\*\* | v10\_1 |
| e12 |  |  | 1.679 | .223 | 7.518 | \*\*\* | v11\_1 |
| e13 |  |  | 1.611 | .212 | 7.608 | \*\*\* | v12\_1 |
| e14 |  |  | 1.125 | .175 | 6.425 | \*\*\* | v13\_1 |
| e15 |  |  | 1.254 | .182 | 6.874 | \*\*\* | v14\_1 |
| e16 |  |  | 1.398 | .199 | 7.025 | \*\*\* | v15\_1 |
| e17 |  |  | 1.623 | .220 | 7.363 | \*\*\* | v16\_1 |
| e18 |  |  | 1.114 | .179 | 6.232 | \*\*\* | v17\_1 |
| e20 |  |  | 1.299 | .186 | 6.991 | \*\*\* | v18\_1 |

##### Squared Multiple Correlations: (east - Structural residuals)

|  |  |  | Estimate |
| --- | --- | --- | --- |
| AI自我效能 |  |  | .233 |
| 防御性动机 |  |  | .238 |
| 使用行为 |  |  | .378 |
| AB5 |  |  | .578 |
| AB3 |  |  | .660 |
| AB2 |  |  | .527 |
| AB1 |  |  | .576 |
| SE5 |  |  | .611 |
| SE4 |  |  | .658 |
| SE3 |  |  | .497 |
| SE2 |  |  | .515 |
| SE1 |  |  | .409 |
| DM5 |  |  | .549 |
| DM3 |  |  | .540 |
| DM2 |  |  | .576 |
| DM1 |  |  | .502 |
| AA5 |  |  | .634 |
| AA4 |  |  | .589 |
| AA3 |  |  | .541 |
| AA2 |  |  | .603 |
| AA1 |  |  | .689 |

##### User-defined estimands: (east - Structural residuals)

|  |  |  |  |
| --- | --- | --- | --- |
| diff1 |  |  | .000 |
| diff2 |  |  | .024 |
| diff3 |  |  | .088 |
| diff4 |  |  | .029 |
| diff5 |  |  | -.303 |

##### Modification Indices (east - Structural residuals)

##### Covariances: (east - Structural residuals)

|  |  |  | M.I. | Par Change |
| --- | --- | --- | --- | --- |
| e21 | <--> | AI威胁感知 | 4.524 | .297 |
| e17 | <--> | e22 | 4.405 | -.287 |
| e13 | <--> | e16 | 7.081 | -.379 |
| e4 | <--> | e17 | 4.592 | .293 |
| e4 | <--> | e11 | 5.162 | .324 |
| e2 | <--> | e8 | 4.262 | -.267 |
| e1 | <--> | e15 | 5.423 | .265 |

##### Variances: (east - Structural residuals)

|  |  |  | M.I. | Par Change |
| --- | --- | --- | --- | --- |

##### Regression Weights: (east - Structural residuals)

|  |  |  | M.I. | Par Change |
| --- | --- | --- | --- | --- |
| AI自我效能 | <--- | AI威胁感知 | 4.524 | .136 |
| AB5 | <--- | SE3 | 4.248 | .117 |
| SE5 | <--- | AA1 | 4.975 | .126 |
| DM5 | <--- | AB5 | 5.240 | -.134 |
| DM5 | <--- | SE2 | 4.396 | -.116 |

##### Bootstrap (east - Structural residuals)

##### Bootstrap standard errors (east - Structural residuals)

##### Scalar Estimates (east - Structural residuals)

##### Regression Weights: (east - Structural residuals)

| Parameter | | | SE | SE-SE | Mean | Bias | SE-Bias |
| --- | --- | --- | --- | --- | --- | --- | --- |
| AI自我效能 | <--- | AI威胁感知 | .048 | .001 | .378 | .003 | .002 |
| 防御性动机 | <--- | AI威胁感知 | .063 | .001 | .402 | .002 | .002 |
| 防御性动机 | <--- | AI自我效能 | .078 | .002 | .074 | .002 | .002 |
| 使用行为 | <--- | 防御性动机 | .061 | .001 | .407 | .003 | .002 |
| 使用行为 | <--- | AI自我效能 | .073 | .002 | .461 | -.002 | .002 |
| AA1 | <--- | AI威胁感知 | .000 | .000 | 1.000 | .000 | .000 |
| AA2 | <--- | AI威胁感知 | .048 | .001 | .952 | -.001 | .002 |
| AA3 | <--- | AI威胁感知 | .046 | .001 | .923 | .002 | .001 |
| AA4 | <--- | AI威胁感知 | .051 | .001 | .919 | .000 | .002 |
| AA5 | <--- | AI威胁感知 | .052 | .001 | .940 | .002 | .002 |
| DM1 | <--- | 防御性动机 | .000 | .000 | 1.000 | .000 | .000 |
| DM2 | <--- | 防御性动机 | .073 | .002 | 1.032 | .005 | .002 |
| DM3 | <--- | 防御性动机 | .075 | .002 | 1.057 | .007 | .002 |
| DM5 | <--- | 防御性动机 | .078 | .002 | .983 | .004 | .002 |
| SE1 | <--- | AI自我效能 | .000 | .000 | 1.000 | .000 | .000 |
| SE2 | <--- | AI自我效能 | .093 | .002 | 1.158 | -.005 | .003 |
| SE3 | <--- | AI自我效能 | .088 | .002 | 1.101 | .002 | .003 |
| SE4 | <--- | AI自我效能 | .097 | .002 | 1.278 | -.003 | .003 |
| SE5 | <--- | AI自我效能 | .093 | .002 | 1.222 | .000 | .003 |
| AB1 | <--- | 使用行为 | .000 | .000 | 1.000 | .000 | .000 |
| AB2 | <--- | 使用行为 | .059 | .001 | .979 | .002 | .002 |
| AB3 | <--- | 使用行为 | .063 | .001 | 1.071 | .003 | .002 |
| AB5 | <--- | 使用行为 | .065 | .001 | .970 | .002 | .002 |

##### Standardized Regression Weights: (east - Structural residuals)

| Parameter | | | SE | SE-SE | Mean | Bias | SE-Bias |
| --- | --- | --- | --- | --- | --- | --- | --- |
| AI自我效能 | <--- | AI威胁感知 | .045 | .001 | .484 | .001 | .001 |
| 防御性动机 | <--- | AI威胁感知 | .062 | .001 | .457 | .003 | .002 |
| 防御性动机 | <--- | AI自我效能 | .069 | .002 | .066 | .002 | .002 |
| 使用行为 | <--- | 防御性动机 | .053 | .001 | .382 | .001 | .002 |
| 使用行为 | <--- | AI自我效能 | .052 | .001 | .384 | -.002 | .002 |
| AA1 | <--- | AI威胁感知 | .028 | .001 | .830 | .000 | .001 |
| AA2 | <--- | AI威胁感知 | .026 | .001 | .777 | .000 | .001 |
| AA3 | <--- | AI威胁感知 | .030 | .001 | .738 | .002 | .001 |
| AA4 | <--- | AI威胁感知 | .029 | .001 | .768 | .001 | .001 |
| AA5 | <--- | AI威胁感知 | .026 | .001 | .798 | .002 | .001 |
| DM1 | <--- | 防御性动机 | .037 | .001 | .708 | .000 | .001 |
| DM2 | <--- | 防御性动机 | .034 | .001 | .760 | .001 | .001 |
| DM3 | <--- | 防御性动机 | .033 | .001 | .737 | .002 | .001 |
| DM5 | <--- | 防御性动机 | .035 | .001 | .742 | .001 | .001 |
| SE1 | <--- | AI自我效能 | .039 | .001 | .643 | .003 | .001 |
| SE2 | <--- | AI自我效能 | .041 | .001 | .717 | .000 | .001 |
| SE3 | <--- | AI自我效能 | .035 | .001 | .708 | .003 | .001 |
| SE4 | <--- | AI自我效能 | .028 | .001 | .812 | .001 | .001 |
| SE5 | <--- | AI自我效能 | .031 | .001 | .784 | .002 | .001 |
| AB1 | <--- | 使用行为 | .034 | .001 | .760 | .002 | .001 |
| AB2 | <--- | 使用行为 | .036 | .001 | .729 | .003 | .001 |
| AB3 | <--- | 使用行为 | .027 | .001 | .814 | .002 | .001 |
| AB5 | <--- | 使用行为 | .034 | .001 | .762 | .001 | .001 |

##### Variances: (east - Structural residuals)

| Parameter | | | SE | SE-SE | Mean | Bias | SE-Bias |
| --- | --- | --- | --- | --- | --- | --- | --- |
| AI威胁感知 |  |  | .189 | .004 | 2.188 | .001 | .006 |
| e21 |  |  | .145 | .003 | 1.019 | .008 | .005 |
| e23 |  |  | .166 | .004 | 1.265 | -.022 | .005 |
| e22 |  |  | .141 | .003 | 1.173 | -.006 | .004 |
| e1 |  |  | .165 | .004 | .990 | .002 | .005 |
| e2 |  |  | .156 | .003 | 1.299 | -.008 | .005 |
| e3 |  |  | .190 | .004 | 1.554 | -.017 | .006 |
| e4 |  |  | .162 | .004 | 1.279 | -.013 | .005 |
| e5 |  |  | .132 | .003 | 1.096 | -.019 | .004 |
| e6 |  |  | .222 | .005 | 1.664 | -.012 | .007 |
| e8 |  |  | .184 | .004 | 1.296 | -.013 | .006 |
| e9 |  |  | .211 | .005 | 1.567 | -.018 | .007 |
| e10 |  |  | .170 | .004 | 1.314 | -.014 | .005 |
| e11 |  |  | .211 | .005 | 1.885 | -.019 | .007 |
| e12 |  |  | .266 | .006 | 1.671 | -.008 | .008 |
| e13 |  |  | .184 | .004 | 1.588 | -.022 | .006 |
| e14 |  |  | .171 | .004 | 1.115 | -.010 | .005 |
| e15 |  |  | .187 | .004 | 1.233 | -.021 | .006 |
| e16 |  |  | .193 | .004 | 1.380 | -.017 | .006 |
| e17 |  |  | .237 | .005 | 1.602 | -.021 | .007 |
| e18 |  |  | .160 | .004 | 1.098 | -.015 | .005 |
| e20 |  |  | .183 | .004 | 1.284 | -.015 | .006 |

##### Squared Multiple Correlations: (east - Structural residuals)

| Parameter | | | SE | SE-SE | Mean | Bias | SE-Bias |
| --- | --- | --- | --- | --- | --- | --- | --- |
| AI自我效能 |  |  | .044 | .001 | .236 | .003 | .001 |
| 防御性动机 |  |  | .048 | .001 | .249 | .010 | .002 |
| 使用行为 |  |  | .050 | .001 | .382 | .004 | .002 |
| AB5 |  |  | .052 | .001 | .581 | .003 | .002 |
| AB3 |  |  | .044 | .001 | .664 | .004 | .001 |
| AB2 |  |  | .052 | .001 | .532 | .005 | .002 |
| AB1 |  |  | .051 | .001 | .579 | .004 | .002 |
| SE5 |  |  | .049 | .001 | .616 | .005 | .002 |
| SE4 |  |  | .045 | .001 | .660 | .002 | .001 |
| SE3 |  |  | .049 | .001 | .502 | .005 | .002 |
| SE2 |  |  | .059 | .001 | .516 | .001 | .002 |
| SE1 |  |  | .050 | .001 | .415 | .006 | .002 |
| DM5 |  |  | .052 | .001 | .552 | .002 | .002 |
| DM3 |  |  | .049 | .001 | .544 | .004 | .002 |
| DM2 |  |  | .052 | .001 | .579 | .003 | .002 |
| DM1 |  |  | .053 | .001 | .503 | .001 | .002 |
| AA5 |  |  | .041 | .001 | .637 | .004 | .001 |
| AA4 |  |  | .045 | .001 | .591 | .002 | .001 |
| AA3 |  |  | .043 | .001 | .545 | .004 | .001 |
| AA2 |  |  | .040 | .001 | .604 | .000 | .001 |
| AA1 |  |  | .046 | .001 | .689 | .000 | .001 |

##### User-defined estimands: (east - Structural residuals)

| Parameter | | | SE | SE-SE | Mean | Bias | SE-Bias |
| --- | --- | --- | --- | --- | --- | --- | --- |
| diff1 |  |  | .000 | .000 | .000 | .000 | .000 |
| diff2 |  |  | .075 | .002 | .023 | -.001 | .002 |
| diff3 |  |  | .095 | .002 | .082 | -.006 | .003 |
| diff4 |  |  | .076 | .002 | .029 | .000 | .002 |
| diff5 |  |  | .092 | .002 | -.304 | -.001 | .003 |

##### Bootstrap Confidence (east - Structural residuals)

##### Percentile method (east - Structural residuals)

##### 95% confidence intervals (percentile method)

##### Scalar Estimates (east - Structural residuals)

##### Regression Weights: (east - Structural residuals)

| Parameter | | | Estimate | Lower | Upper | P |
| --- | --- | --- | --- | --- | --- | --- |
| AI自我效能 | <--- | AI威胁感知 | .375 | .289 | .473 | .002 |
| 防御性动机 | <--- | AI威胁感知 | .399 | .284 | .531 | .002 |
| 防御性动机 | <--- | AI自我效能 | .072 | -.078 | .219 | .375 |
| 使用行为 | <--- | 防御性动机 | .404 | .293 | .531 | .002 |
| 使用行为 | <--- | AI自我效能 | .463 | .325 | .607 | .002 |
| AA1 | <--- | AI威胁感知 | 1.000 | 1.000 | 1.000 | ... |
| AA2 | <--- | AI威胁感知 | .954 | .864 | 1.047 | .002 |
| AA3 | <--- | AI威胁感知 | .921 | .834 | 1.016 | .002 |
| AA4 | <--- | AI威胁感知 | .919 | .826 | 1.028 | .002 |
| AA5 | <--- | AI威胁感知 | .939 | .841 | 1.052 | .002 |
| DM1 | <--- | 防御性动机 | 1.000 | 1.000 | 1.000 | ... |
| DM2 | <--- | 防御性动机 | 1.027 | .890 | 1.173 | .002 |
| DM3 | <--- | 防御性动机 | 1.050 | .914 | 1.209 | .002 |
| DM5 | <--- | 防御性动机 | .979 | .841 | 1.140 | .002 |
| SE1 | <--- | AI自我效能 | 1.000 | 1.000 | 1.000 | ... |
| SE2 | <--- | AI自我效能 | 1.162 | .981 | 1.355 | .002 |
| SE3 | <--- | AI自我效能 | 1.099 | .945 | 1.288 | .002 |
| SE4 | <--- | AI自我效能 | 1.281 | 1.110 | 1.482 | .002 |
| SE5 | <--- | AI自我效能 | 1.222 | 1.060 | 1.443 | .002 |
| AB1 | <--- | 使用行为 | 1.000 | 1.000 | 1.000 | ... |
| AB2 | <--- | 使用行为 | .976 | .867 | 1.106 | .002 |
| AB3 | <--- | 使用行为 | 1.068 | .955 | 1.203 | .002 |
| AB5 | <--- | 使用行为 | .969 | .854 | 1.119 | .002 |

##### Standardized Regression Weights: (east - Structural residuals)

| Parameter | | | Estimate | Lower | Upper | P |
| --- | --- | --- | --- | --- | --- | --- |
| AI自我效能 | <--- | AI威胁感知 | .483 | .392 | .572 | .002 |
| 防御性动机 | <--- | AI威胁感知 | .454 | .333 | .578 | .002 |
| 防御性动机 | <--- | AI自我效能 | .064 | -.067 | .199 | .375 |
| 使用行为 | <--- | 防御性动机 | .382 | .278 | .478 | .002 |
| 使用行为 | <--- | AI自我效能 | .386 | .277 | .479 | .002 |
| AA1 | <--- | AI威胁感知 | .830 | .773 | .882 | .002 |
| AA2 | <--- | AI威胁感知 | .777 | .725 | .826 | .002 |
| AA3 | <--- | AI威胁感知 | .736 | .677 | .794 | .002 |
| AA4 | <--- | AI威胁感知 | .767 | .711 | .823 | .002 |
| AA5 | <--- | AI威胁感知 | .796 | .745 | .847 | .002 |
| DM1 | <--- | 防御性动机 | .708 | .636 | .777 | .002 |
| DM2 | <--- | 防御性动机 | .759 | .695 | .828 | .002 |
| DM3 | <--- | 防御性动机 | .735 | .666 | .801 | .002 |
| DM5 | <--- | 防御性动机 | .741 | .668 | .807 | .002 |
| SE1 | <--- | AI自我效能 | .640 | .563 | .718 | .002 |
| SE2 | <--- | AI自我效能 | .717 | .635 | .795 | .002 |
| SE3 | <--- | AI自我效能 | .705 | .634 | .775 | .002 |
| SE4 | <--- | AI自我效能 | .811 | .752 | .864 | .002 |
| SE5 | <--- | AI自我效能 | .782 | .720 | .841 | .002 |
| AB1 | <--- | 使用行为 | .759 | .690 | .823 | .002 |
| AB2 | <--- | 使用行为 | .726 | .660 | .798 | .002 |
| AB3 | <--- | 使用行为 | .812 | .761 | .867 | .002 |
| AB5 | <--- | 使用行为 | .760 | .696 | .827 | .002 |

##### Variances: (east - Structural residuals)

| Parameter | | | Estimate | Lower | Upper | P |
| --- | --- | --- | --- | --- | --- | --- |
| AI威胁感知 |  |  | 2.187 | 1.799 | 2.554 | .002 |
| e21 |  |  | 1.010 | .757 | 1.326 | .002 |
| e23 |  |  | 1.287 | .957 | 1.619 | .002 |
| e22 |  |  | 1.179 | .895 | 1.451 | .002 |
| e1 |  |  | .988 | .672 | 1.319 | .002 |
| e2 |  |  | 1.307 | 1.012 | 1.616 | .002 |
| e3 |  |  | 1.571 | 1.213 | 1.959 | .002 |
| e4 |  |  | 1.292 | .964 | 1.600 | .002 |
| e5 |  |  | 1.115 | .840 | 1.353 | .002 |
| e6 |  |  | 1.677 | 1.240 | 2.110 | .002 |
| e8 |  |  | 1.310 | .911 | 1.653 | .002 |
| e9 |  |  | 1.585 | 1.182 | 2.015 | .002 |
| e10 |  |  | 1.327 | .979 | 1.663 | .002 |
| e11 |  |  | 1.904 | 1.493 | 2.333 | .002 |
| e12 |  |  | 1.679 | 1.154 | 2.206 | .002 |
| e13 |  |  | 1.611 | 1.234 | 1.957 | .002 |
| e14 |  |  | 1.125 | .797 | 1.461 | .002 |
| e15 |  |  | 1.254 | .880 | 1.615 | .002 |
| e16 |  |  | 1.398 | 1.015 | 1.787 | .002 |
| e17 |  |  | 1.623 | 1.149 | 2.104 | .002 |
| e18 |  |  | 1.114 | .790 | 1.423 | .002 |
| e20 |  |  | 1.299 | .937 | 1.653 | .002 |

##### Squared Multiple Correlations: (east - Structural residuals)

| Parameter | | | Estimate | Lower | Upper | P |
| --- | --- | --- | --- | --- | --- | --- |
| AI自我效能 |  |  | .233 | .154 | .327 | .002 |
| 防御性动机 |  |  | .238 | .160 | .350 | .002 |
| 使用行为 |  |  | .378 | .288 | .480 | .002 |
| AB5 |  |  | .578 | .484 | .684 | .002 |
| AB3 |  |  | .660 | .579 | .752 | .002 |
| AB2 |  |  | .527 | .435 | .636 | .002 |
| AB1 |  |  | .576 | .476 | .678 | .002 |
| SE5 |  |  | .611 | .519 | .708 | .002 |
| SE4 |  |  | .658 | .565 | .746 | .002 |
| SE3 |  |  | .497 | .402 | .601 | .002 |
| SE2 |  |  | .515 | .404 | .633 | .002 |
| SE1 |  |  | .409 | .316 | .515 | .002 |
| DM5 |  |  | .549 | .446 | .651 | .002 |
| DM3 |  |  | .540 | .443 | .641 | .002 |
| DM2 |  |  | .576 | .483 | .685 | .002 |
| DM1 |  |  | .502 | .404 | .604 | .002 |
| AA5 |  |  | .634 | .555 | .718 | .002 |
| AA4 |  |  | .589 | .506 | .677 | .002 |
| AA3 |  |  | .541 | .458 | .630 | .002 |
| AA2 |  |  | .603 | .526 | .682 | .002 |
| AA1 |  |  | .689 | .597 | .779 | .002 |

##### User-defined estimands: (east - Structural residuals)

| Parameter | | | Estimate | Lower | Upper | P |
| --- | --- | --- | --- | --- | --- | --- |
| diff1 |  |  | .000 | .000 | .000 | ... |
| diff2 |  |  | .024 | -.128 | .166 | .756 |
| diff3 |  |  | .088 | -.090 | .279 | .379 |
| diff4 |  |  | .029 | -.123 | .190 | .751 |
| diff5 |  |  | -.303 | -.478 | -.131 | .002 |

##### Bias-corrected percentile method (east - Structural residuals)

##### 95% confidence intervals (bias-corrected percentile method)

##### Scalar Estimates (east - Structural residuals)

##### Regression Weights: (east - Structural residuals)

| Parameter | | | Estimate | Lower | Upper | P |
| --- | --- | --- | --- | --- | --- | --- |
| AI自我效能 | <--- | AI威胁感知 | .375 | .282 | .467 | .003 |
| 防御性动机 | <--- | AI威胁感知 | .399 | .273 | .523 | .003 |
| 防御性动机 | <--- | AI自我效能 | .072 | -.086 | .217 | .408 |
| 使用行为 | <--- | 防御性动机 | .404 | .290 | .525 | .003 |
| 使用行为 | <--- | AI自我效能 | .463 | .332 | .616 | .001 |
| AA1 | <--- | AI威胁感知 | 1.000 | 1.000 | 1.000 | ... |
| AA2 | <--- | AI威胁感知 | .954 | .866 | 1.055 | .001 |
| AA3 | <--- | AI威胁感知 | .921 | .832 | 1.014 | .002 |
| AA4 | <--- | AI威胁感知 | .919 | .831 | 1.029 | .002 |
| AA5 | <--- | AI威胁感知 | .939 | .840 | 1.050 | .002 |
| DM1 | <--- | 防御性动机 | 1.000 | 1.000 | 1.000 | ... |
| DM2 | <--- | 防御性动机 | 1.027 | .884 | 1.168 | .003 |
| DM3 | <--- | 防御性动机 | 1.050 | .905 | 1.205 | .003 |
| DM5 | <--- | 防御性动机 | .979 | .839 | 1.135 | .002 |
| SE1 | <--- | AI自我效能 | 1.000 | 1.000 | 1.000 | ... |
| SE2 | <--- | AI自我效能 | 1.162 | 1.004 | 1.368 | .001 |
| SE3 | <--- | AI自我效能 | 1.099 | .951 | 1.293 | .001 |
| SE4 | <--- | AI自我效能 | 1.281 | 1.120 | 1.496 | .001 |
| SE5 | <--- | AI自我效能 | 1.222 | 1.066 | 1.456 | .001 |
| AB1 | <--- | 使用行为 | 1.000 | 1.000 | 1.000 | ... |
| AB2 | <--- | 使用行为 | .976 | .869 | 1.111 | .002 |
| AB3 | <--- | 使用行为 | 1.068 | .955 | 1.203 | .002 |
| AB5 | <--- | 使用行为 | .969 | .857 | 1.128 | .001 |

##### Standardized Regression Weights: (east - Structural residuals)

| Parameter | | | Estimate | Lower | Upper | P |
| --- | --- | --- | --- | --- | --- | --- |
| AI自我效能 | <--- | AI威胁感知 | .483 | .390 | .567 | .003 |
| 防御性动机 | <--- | AI威胁感知 | .454 | .324 | .571 | .003 |
| 防御性动机 | <--- | AI自我效能 | .064 | -.072 | .196 | .411 |
| 使用行为 | <--- | 防御性动机 | .382 | .275 | .478 | .002 |
| 使用行为 | <--- | AI自我效能 | .386 | .278 | .480 | .002 |
| AA1 | <--- | AI威胁感知 | .830 | .772 | .881 | .002 |
| AA2 | <--- | AI威胁感知 | .777 | .720 | .824 | .003 |
| AA3 | <--- | AI威胁感知 | .736 | .670 | .788 | .004 |
| AA4 | <--- | AI威胁感知 | .767 | .710 | .822 | .002 |
| AA5 | <--- | AI威胁感知 | .796 | .739 | .841 | .004 |
| DM1 | <--- | 防御性动机 | .708 | .636 | .778 | .002 |
| DM2 | <--- | 防御性动机 | .759 | .692 | .825 | .003 |
| DM3 | <--- | 防御性动机 | .735 | .655 | .791 | .004 |
| DM5 | <--- | 防御性动机 | .741 | .662 | .803 | .003 |
| SE1 | <--- | AI自我效能 | .640 | .554 | .712 | .004 |
| SE2 | <--- | AI自我效能 | .717 | .636 | .796 | .002 |
| SE3 | <--- | AI自我效能 | .705 | .629 | .766 | .004 |
| SE4 | <--- | AI自我效能 | .811 | .749 | .861 | .003 |
| SE5 | <--- | AI自我效能 | .782 | .716 | .836 | .004 |
| AB1 | <--- | 使用行为 | .759 | .685 | .818 | .003 |
| AB2 | <--- | 使用行为 | .726 | .653 | .795 | .003 |
| AB3 | <--- | 使用行为 | .812 | .755 | .863 | .003 |
| AB5 | <--- | 使用行为 | .760 | .688 | .824 | .003 |

##### Variances: (east - Structural residuals)

| Parameter | | | Estimate | Lower | Upper | P |
| --- | --- | --- | --- | --- | --- | --- |
| AI威胁感知 |  |  | 2.187 | 1.781 | 2.537 | .003 |
| e21 |  |  | 1.010 | .757 | 1.326 | .002 |
| e23 |  |  | 1.287 | 1.002 | 1.671 | .000 |
| e22 |  |  | 1.179 | .909 | 1.459 | .001 |
| e1 |  |  | .988 | .688 | 1.325 | .002 |
| e2 |  |  | 1.307 | 1.024 | 1.627 | .001 |
| e3 |  |  | 1.571 | 1.246 | 2.007 | .001 |
| e4 |  |  | 1.292 | 1.005 | 1.632 | .001 |
| e5 |  |  | 1.115 | .890 | 1.404 | .001 |
| e6 |  |  | 1.677 | 1.289 | 2.172 | .001 |
| e8 |  |  | 1.310 | .946 | 1.674 | .001 |
| e9 |  |  | 1.585 | 1.229 | 2.098 | .001 |
| e10 |  |  | 1.327 | 1.027 | 1.711 | .001 |
| e11 |  |  | 1.904 | 1.520 | 2.359 | .001 |
| e12 |  |  | 1.679 | 1.188 | 2.214 | .001 |
| e13 |  |  | 1.611 | 1.294 | 1.987 | .001 |
| e14 |  |  | 1.125 | .831 | 1.497 | .001 |
| e15 |  |  | 1.254 | .935 | 1.652 | .001 |
| e16 |  |  | 1.398 | 1.040 | 1.819 | .001 |
| e17 |  |  | 1.623 | 1.222 | 2.166 | .001 |
| e18 |  |  | 1.114 | .819 | 1.460 | .001 |
| e20 |  |  | 1.299 | .977 | 1.691 | .001 |

##### Squared Multiple Correlations: (east - Structural residuals)

| Parameter | | | Estimate | Lower | Upper | P |
| --- | --- | --- | --- | --- | --- | --- |
| AI自我效能 |  |  | .233 | .152 | .322 | .003 |
| 防御性动机 |  |  | .238 | .146 | .332 | .005 |
| 使用行为 |  |  | .378 | .286 | .475 | .003 |
| AB5 |  |  | .578 | .473 | .679 | .003 |
| AB3 |  |  | .660 | .570 | .746 | .003 |
| AB2 |  |  | .527 | .426 | .632 | .003 |
| AB1 |  |  | .576 | .469 | .669 | .003 |
| SE5 |  |  | .611 | .513 | .699 | .004 |
| SE4 |  |  | .658 | .561 | .741 | .003 |
| SE3 |  |  | .497 | .396 | .587 | .004 |
| SE2 |  |  | .515 | .404 | .633 | .002 |
| SE1 |  |  | .409 | .307 | .506 | .004 |
| DM5 |  |  | .549 | .439 | .644 | .003 |
| DM3 |  |  | .540 | .429 | .626 | .004 |
| DM2 |  |  | .576 | .478 | .681 | .003 |
| DM1 |  |  | .502 | .405 | .605 | .002 |
| AA5 |  |  | .634 | .546 | .707 | .004 |
| AA4 |  |  | .589 | .504 | .676 | .002 |
| AA3 |  |  | .541 | .449 | .620 | .004 |
| AA2 |  |  | .603 | .519 | .679 | .003 |
| AA1 |  |  | .689 | .596 | .777 | .002 |

##### User-defined estimands: (east - Structural residuals)

| Parameter | | | Estimate | Lower | Upper | P |
| --- | --- | --- | --- | --- | --- | --- |
| diff1 |  |  | .000 | .000 | .000 | ... |
| diff2 |  |  | .024 | -.131 | .165 | .772 |
| diff3 |  |  | .088 | -.084 | .294 | .312 |
| diff4 |  |  | .029 | -.124 | .188 | .771 |
| diff5 |  |  | -.303 | -.480 | -.134 | .002 |

##### other (other - Structural residuals)

##### Estimates (other - Structural residuals)

##### Scalar Estimates (other - Structural residuals)

##### Maximum Likelihood Estimates

##### Regression Weights: (other - Structural residuals)

|  |  |  | Estimate | S.E. | C.R. | P | Label |
| --- | --- | --- | --- | --- | --- | --- | --- |
| AI自我效能 | <--- | AI威胁感知 | .375 | .049 | 7.609 | \*\*\* | H1a |
| 防御性动机 | <--- | AI威胁感知 | .399 | .062 | 6.469 | \*\*\* | H2a |
| 防御性动机 | <--- | AI自我效能 | .072 | .074 | .979 | .328 | H5a |
| 使用行为 | <--- | 防御性动机 | .404 | .064 | 6.272 | \*\*\* | H4a |
| 使用行为 | <--- | AI自我效能 | .463 | .074 | 6.284 | \*\*\* | H3a |
| AA1 | <--- | AI威胁感知 | 1.000 |  |  |  |  |
| AA2 | <--- | AI威胁感知 | .954 | .057 | 16.831 | \*\*\* | a1\_1 |
| AA3 | <--- | AI威胁感知 | .921 | .058 | 15.921 | \*\*\* | a2\_1 |
| AA4 | <--- | AI威胁感知 | .919 | .058 | 15.985 | \*\*\* | a3\_1 |
| AA5 | <--- | AI威胁感知 | .939 | .056 | 16.739 | \*\*\* | a4\_1 |
| DM1 | <--- | 防御性动机 | 1.000 |  |  |  |  |
| DM2 | <--- | 防御性动机 | 1.027 | .082 | 12.538 | \*\*\* | a5\_1 |
| DM3 | <--- | 防御性动机 | 1.050 | .082 | 12.780 | \*\*\* | a6\_1 |
| DM5 | <--- | 防御性动机 | .979 | .081 | 12.078 | \*\*\* | a7\_1 |
| SE1 | <--- | AI自我效能 | 1.000 |  |  |  |  |
| SE2 | <--- | AI自我效能 | 1.162 | .101 | 11.519 | \*\*\* | a8\_1 |
| SE3 | <--- | AI自我效能 | 1.099 | .097 | 11.323 | \*\*\* | a9\_1 |
| SE4 | <--- | AI自我效能 | 1.281 | .102 | 12.520 | \*\*\* | a10\_1 |
| SE5 | <--- | AI自我效能 | 1.222 | .099 | 12.377 | \*\*\* | a11\_1 |
| AB1 | <--- | 使用行为 | 1.000 |  |  |  |  |
| AB2 | <--- | 使用行为 | .976 | .071 | 13.755 | \*\*\* | a12\_1 |
| AB3 | <--- | 使用行为 | 1.068 | .071 | 14.990 | \*\*\* | a13\_1 |
| AB5 | <--- | 使用行为 | .969 | .071 | 13.653 | \*\*\* | a14\_1 |

##### Standardized Regression Weights: (other - Structural residuals)

|  |  |  | Estimate |
| --- | --- | --- | --- |
| AI自我效能 | <--- | AI威胁感知 | .483 |
| 防御性动机 | <--- | AI威胁感知 | .454 |
| 防御性动机 | <--- | AI自我效能 | .064 |
| 使用行为 | <--- | 防御性动机 | .382 |
| 使用行为 | <--- | AI自我效能 | .386 |
| AA1 | <--- | AI威胁感知 | .811 |
| AA2 | <--- | AI威胁感知 | .789 |
| AA3 | <--- | AI威胁感知 | .760 |
| AA4 | <--- | AI威胁感知 | .742 |
| AA5 | <--- | AI威胁感知 | .768 |
| DM1 | <--- | 防御性动机 | .706 |
| DM2 | <--- | 防御性动机 | .729 |
| DM3 | <--- | 防御性动机 | .780 |
| DM5 | <--- | 防御性动机 | .683 |
| SE1 | <--- | AI自我效能 | .636 |
| SE2 | <--- | AI自我效能 | .714 |
| SE3 | <--- | AI自我效能 | .695 |
| SE4 | <--- | AI自我效能 | .800 |
| SE5 | <--- | AI自我效能 | .797 |
| AB1 | <--- | 使用行为 | .757 |
| AB2 | <--- | 使用行为 | .743 |
| AB3 | <--- | 使用行为 | .807 |
| AB5 | <--- | 使用行为 | .707 |

##### Variances: (other - Structural residuals)

|  |  |  | Estimate | S.E. | C.R. | P | Label |
| --- | --- | --- | --- | --- | --- | --- | --- |
| AI威胁感知 |  |  | 2.187 | .232 | 9.413 | \*\*\* | vvv1\_1 |
| e21 |  |  | 1.010 | .157 | 6.428 | \*\*\* | vv1\_1 |
| e23 |  |  | 1.287 | .182 | 7.083 | \*\*\* | vv3\_1 |
| e22 |  |  | 1.179 | .156 | 7.557 | \*\*\* | vv2\_1 |
| e1 |  |  | 1.139 | .139 | 8.214 | \*\*\* | v1\_2 |
| e2 |  |  | 1.209 | .141 | 8.563 | \*\*\* | v2\_2 |
| e3 |  |  | 1.355 | .152 | 8.923 | \*\*\* | v3\_2 |
| e4 |  |  | 1.513 | .166 | 9.128 | \*\*\* | v4\_2 |
| e5 |  |  | 1.339 | .151 | 8.844 | \*\*\* | v5\_2 |
| e6 |  |  | 1.700 | .196 | 8.657 | \*\*\* | v6\_2 |
| e8 |  |  | 1.572 | .188 | 8.373 | \*\*\* | v7\_2 |
| e9 |  |  | 1.195 | .160 | 7.460 | \*\*\* | v8\_2 |
| e10 |  |  | 1.852 | .207 | 8.928 | \*\*\* | v9\_2 |
| e11 |  |  | 1.942 | .201 | 9.645 | \*\*\* | v10\_2 |
| e12 |  |  | 1.716 | .189 | 9.077 | \*\*\* | v11\_2 |
| e13 |  |  | 1.702 | .184 | 9.241 | \*\*\* | v12\_2 |
| e14 |  |  | 1.213 | .154 | 7.891 | \*\*\* | v13\_2 |
| e15 |  |  | 1.132 | .142 | 7.950 | \*\*\* | v14\_2 |
| e16 |  |  | 1.415 | .170 | 8.345 | \*\*\* | v15\_2 |
| e17 |  |  | 1.462 | .171 | 8.525 | \*\*\* | v16\_2 |
| e18 |  |  | 1.158 | .156 | 7.415 | \*\*\* | v17\_2 |
| e20 |  |  | 1.775 | .198 | 8.967 | \*\*\* | v18\_2 |

##### Squared Multiple Correlations: (other - Structural residuals)

|  |  |  | Estimate |
| --- | --- | --- | --- |
| AI自我效能 |  |  | .233 |
| 防御性动机 |  |  | .238 |
| 使用行为 |  |  | .378 |
| AB5 |  |  | .500 |
| AB3 |  |  | .651 |
| AB2 |  |  | .553 |
| AB1 |  |  | .573 |
| SE5 |  |  | .635 |
| SE4 |  |  | .641 |
| SE3 |  |  | .483 |
| SE2 |  |  | .509 |
| SE1 |  |  | .404 |
| DM5 |  |  | .466 |
| DM3 |  |  | .609 |
| DM2 |  |  | .531 |
| DM1 |  |  | .498 |
| AA5 |  |  | .590 |
| AA4 |  |  | .550 |
| AA3 |  |  | .578 |
| AA2 |  |  | .622 |
| AA1 |  |  | .658 |

##### User-defined estimands: (other - Structural residuals)

|  |  |  |  |
| --- | --- | --- | --- |
| diff1 |  |  | .000 |
| diff2 |  |  | .024 |
| diff3 |  |  | .088 |
| diff4 |  |  | .029 |
| diff5 |  |  | -.303 |

##### Modification Indices (other - Structural residuals)

##### Covariances: (other - Structural residuals)

|  |  |  | M.I. | Par Change |
| --- | --- | --- | --- | --- |
| e20 | <--> | e23 | 5.450 | -.289 |
| e13 | <--> | AI威胁感知 | 5.728 | -.345 |
| e9 | <--> | e18 | 4.418 | .222 |
| e8 | <--> | e22 | 6.796 | .304 |
| e6 | <--> | e11 | 5.437 | -.321 |
| e5 | <--> | e14 | 12.367 | -.369 |
| e4 | <--> | e5 | 4.622 | .234 |
| e3 | <--> | e17 | 4.109 | .226 |
| e2 | <--> | e14 | 6.220 | .252 |
| e2 | <--> | e13 | 4.290 | -.232 |
| e1 | <--> | e3 | 5.134 | .222 |

##### Variances: (other - Structural residuals)

|  |  |  | M.I. | Par Change |
| --- | --- | --- | --- | --- |

##### Regression Weights: (other - Structural residuals)

|  |  |  | M.I. | Par Change |
| --- | --- | --- | --- | --- |
| SE4 | <--- | AA2 | 4.170 | .095 |
| SE3 | <--- | AI威胁感知 | 5.728 | -.158 |
| SE3 | <--- | AA3 | 5.442 | -.120 |
| SE3 | <--- | AA2 | 8.601 | -.151 |
| SE1 | <--- | DM1 | 7.057 | -.139 |
| DM2 | <--- | AB2 | 4.104 | .103 |
| DM2 | <--- | AB1 | 4.371 | .106 |
| DM1 | <--- | SE1 | 5.332 | -.121 |
| AA5 | <--- | SE4 | 6.185 | -.113 |

##### Bootstrap (other - Structural residuals)

##### Bootstrap standard errors (other - Structural residuals)

##### Scalar Estimates (other - Structural residuals)

##### Regression Weights: (other - Structural residuals)

| Parameter | | | SE | SE-SE | Mean | Bias | SE-Bias |
| --- | --- | --- | --- | --- | --- | --- | --- |
| AI自我效能 | <--- | AI威胁感知 | .048 | .001 | .378 | .003 | .002 |
| 防御性动机 | <--- | AI威胁感知 | .063 | .001 | .402 | .002 | .002 |
| 防御性动机 | <--- | AI自我效能 | .078 | .002 | .074 | .002 | .002 |
| 使用行为 | <--- | 防御性动机 | .061 | .001 | .407 | .003 | .002 |
| 使用行为 | <--- | AI自我效能 | .073 | .002 | .461 | -.002 | .002 |
| AA1 | <--- | AI威胁感知 | .000 | .000 | 1.000 | .000 | .000 |
| AA2 | <--- | AI威胁感知 | .048 | .001 | .952 | -.001 | .002 |
| AA3 | <--- | AI威胁感知 | .046 | .001 | .923 | .002 | .001 |
| AA4 | <--- | AI威胁感知 | .051 | .001 | .919 | .000 | .002 |
| AA5 | <--- | AI威胁感知 | .052 | .001 | .940 | .002 | .002 |
| DM1 | <--- | 防御性动机 | .000 | .000 | 1.000 | .000 | .000 |
| DM2 | <--- | 防御性动机 | .073 | .002 | 1.032 | .005 | .002 |
| DM3 | <--- | 防御性动机 | .075 | .002 | 1.057 | .007 | .002 |
| DM5 | <--- | 防御性动机 | .078 | .002 | .983 | .004 | .002 |
| SE1 | <--- | AI自我效能 | .000 | .000 | 1.000 | .000 | .000 |
| SE2 | <--- | AI自我效能 | .093 | .002 | 1.158 | -.005 | .003 |
| SE3 | <--- | AI自我效能 | .088 | .002 | 1.101 | .002 | .003 |
| SE4 | <--- | AI自我效能 | .097 | .002 | 1.278 | -.003 | .003 |
| SE5 | <--- | AI自我效能 | .093 | .002 | 1.222 | .000 | .003 |
| AB1 | <--- | 使用行为 | .000 | .000 | 1.000 | .000 | .000 |
| AB2 | <--- | 使用行为 | .059 | .001 | .979 | .002 | .002 |
| AB3 | <--- | 使用行为 | .063 | .001 | 1.071 | .003 | .002 |
| AB5 | <--- | 使用行为 | .065 | .001 | .970 | .002 | .002 |

##### Standardized Regression Weights: (other - Structural residuals)

| Parameter | | | SE | SE-SE | Mean | Bias | SE-Bias |
| --- | --- | --- | --- | --- | --- | --- | --- |
| AI自我效能 | <--- | AI威胁感知 | .045 | .001 | .484 | .001 | .001 |
| 防御性动机 | <--- | AI威胁感知 | .062 | .001 | .457 | .003 | .002 |
| 防御性动机 | <--- | AI自我效能 | .069 | .002 | .066 | .002 | .002 |
| 使用行为 | <--- | 防御性动机 | .053 | .001 | .382 | .001 | .002 |
| 使用行为 | <--- | AI自我效能 | .052 | .001 | .384 | -.002 | .002 |
| AA1 | <--- | AI威胁感知 | .024 | .001 | .812 | .001 | .001 |
| AA2 | <--- | AI威胁感知 | .027 | .001 | .787 | -.001 | .001 |
| AA3 | <--- | AI威胁感知 | .026 | .001 | .761 | .001 | .001 |
| AA4 | <--- | AI威胁感知 | .030 | .001 | .741 | .000 | .001 |
| AA5 | <--- | AI威胁感知 | .031 | .001 | .769 | .001 | .001 |
| DM1 | <--- | 防御性动机 | .034 | .001 | .704 | -.002 | .001 |
| DM2 | <--- | 防御性动机 | .033 | .001 | .729 | .001 | .001 |
| DM3 | <--- | 防御性动机 | .029 | .001 | .782 | .002 | .001 |
| DM5 | <--- | 防御性动机 | .036 | .001 | .683 | .000 | .001 |
| SE1 | <--- | AI自我效能 | .036 | .001 | .639 | .003 | .001 |
| SE2 | <--- | AI自我效能 | .033 | .001 | .713 | .000 | .001 |
| SE3 | <--- | AI自我效能 | .033 | .001 | .698 | .003 | .001 |
| SE4 | <--- | AI自我效能 | .025 | .001 | .800 | .000 | .001 |
| SE5 | <--- | AI自我效能 | .027 | .001 | .798 | .001 | .001 |
| AB1 | <--- | 使用行为 | .029 | .001 | .758 | .002 | .001 |
| AB2 | <--- | 使用行为 | .031 | .001 | .744 | .001 | .001 |
| AB3 | <--- | 使用行为 | .028 | .001 | .808 | .001 | .001 |
| AB5 | <--- | 使用行为 | .031 | .001 | .708 | .001 | .001 |

##### Variances: (other - Structural residuals)

| Parameter | | | SE | SE-SE | Mean | Bias | SE-Bias |
| --- | --- | --- | --- | --- | --- | --- | --- |
| AI威胁感知 |  |  | .189 | .004 | 2.188 | .001 | .006 |
| e21 |  |  | .145 | .003 | 1.019 | .008 | .005 |
| e23 |  |  | .166 | .004 | 1.265 | -.022 | .005 |
| e22 |  |  | .141 | .003 | 1.173 | -.006 | .004 |
| e1 |  |  | .133 | .003 | 1.129 | -.010 | .004 |
| e2 |  |  | .145 | .003 | 1.211 | .002 | .005 |
| e3 |  |  | .134 | .003 | 1.342 | -.013 | .004 |
| e4 |  |  | .168 | .004 | 1.505 | -.007 | .005 |
| e5 |  |  | .178 | .004 | 1.326 | -.013 | .006 |
| e6 |  |  | .183 | .004 | 1.699 | -.001 | .006 |
| e8 |  |  | .189 | .004 | 1.560 | -.012 | .006 |
| e9 |  |  | .136 | .003 | 1.177 | -.018 | .004 |
| e10 |  |  | .189 | .004 | 1.838 | -.014 | .006 |
| e11 |  |  | .168 | .004 | 1.922 | -.020 | .005 |
| e12 |  |  | .178 | .004 | 1.701 | -.015 | .006 |
| e13 |  |  | .176 | .004 | 1.680 | -.022 | .006 |
| e14 |  |  | .142 | .003 | 1.207 | -.006 | .004 |
| e15 |  |  | .142 | .003 | 1.119 | -.012 | .004 |
| e16 |  |  | .147 | .003 | 1.395 | -.020 | .005 |
| e17 |  |  | .166 | .004 | 1.451 | -.010 | .005 |
| e18 |  |  | .165 | .004 | 1.148 | -.010 | .005 |
| e20 |  |  | .173 | .004 | 1.761 | -.015 | .005 |

##### Squared Multiple Correlations: (other - Structural residuals)

| Parameter | | | SE | SE-SE | Mean | Bias | SE-Bias |
| --- | --- | --- | --- | --- | --- | --- | --- |
| AI自我效能 |  |  | .044 | .001 | .236 | .003 | .001 |
| 防御性动机 |  |  | .048 | .001 | .249 | .010 | .002 |
| 使用行为 |  |  | .050 | .001 | .382 | .004 | .002 |
| AB5 |  |  | .043 | .001 | .503 | .002 | .001 |
| AB3 |  |  | .045 | .001 | .654 | .003 | .001 |
| AB2 |  |  | .046 | .001 | .555 | .002 | .001 |
| AB1 |  |  | .044 | .001 | .576 | .004 | .001 |
| SE5 |  |  | .042 | .001 | .638 | .003 | .001 |
| SE4 |  |  | .041 | .001 | .641 | .000 | .001 |
| SE3 |  |  | .045 | .001 | .488 | .005 | .001 |
| SE2 |  |  | .047 | .001 | .510 | .001 | .001 |
| SE1 |  |  | .046 | .001 | .409 | .005 | .001 |
| DM5 |  |  | .049 | .001 | .468 | .002 | .002 |
| DM3 |  |  | .046 | .001 | .613 | .004 | .001 |
| DM2 |  |  | .048 | .001 | .533 | .002 | .002 |
| DM1 |  |  | .048 | .001 | .497 | -.001 | .002 |
| AA5 |  |  | .048 | .001 | .593 | .003 | .002 |
| AA4 |  |  | .044 | .001 | .551 | .001 | .001 |
| AA3 |  |  | .039 | .001 | .580 | .002 | .001 |
| AA2 |  |  | .042 | .001 | .620 | -.002 | .001 |
| AA1 |  |  | .039 | .001 | .659 | .002 | .001 |

##### User-defined estimands: (other - Structural residuals)

| Parameter | | | SE | SE-SE | Mean | Bias | SE-Bias |
| --- | --- | --- | --- | --- | --- | --- | --- |
| diff1 |  |  | .000 | .000 | .000 | .000 | .000 |
| diff2 |  |  | .075 | .002 | .023 | -.001 | .002 |
| diff3 |  |  | .095 | .002 | .082 | -.006 | .003 |
| diff4 |  |  | .076 | .002 | .029 | .000 | .002 |
| diff5 |  |  | .092 | .002 | -.304 | -.001 | .003 |

##### Bootstrap Confidence (other - Structural residuals)

##### Percentile method (other - Structural residuals)

##### 95% confidence intervals (percentile method)

##### Scalar Estimates (other - Structural residuals)

##### Regression Weights: (other - Structural residuals)

| Parameter | | | Estimate | Lower | Upper | P |
| --- | --- | --- | --- | --- | --- | --- |
| AI自我效能 | <--- | AI威胁感知 | .375 | .289 | .473 | .002 |
| 防御性动机 | <--- | AI威胁感知 | .399 | .284 | .531 | .002 |
| 防御性动机 | <--- | AI自我效能 | .072 | -.078 | .219 | .375 |
| 使用行为 | <--- | 防御性动机 | .404 | .293 | .531 | .002 |
| 使用行为 | <--- | AI自我效能 | .463 | .325 | .607 | .002 |
| AA1 | <--- | AI威胁感知 | 1.000 | 1.000 | 1.000 | ... |
| AA2 | <--- | AI威胁感知 | .954 | .864 | 1.047 | .002 |
| AA3 | <--- | AI威胁感知 | .921 | .834 | 1.016 | .002 |
| AA4 | <--- | AI威胁感知 | .919 | .826 | 1.028 | .002 |
| AA5 | <--- | AI威胁感知 | .939 | .841 | 1.052 | .002 |
| DM1 | <--- | 防御性动机 | 1.000 | 1.000 | 1.000 | ... |
| DM2 | <--- | 防御性动机 | 1.027 | .890 | 1.173 | .002 |
| DM3 | <--- | 防御性动机 | 1.050 | .914 | 1.209 | .002 |
| DM5 | <--- | 防御性动机 | .979 | .841 | 1.140 | .002 |
| SE1 | <--- | AI自我效能 | 1.000 | 1.000 | 1.000 | ... |
| SE2 | <--- | AI自我效能 | 1.162 | .981 | 1.355 | .002 |
| SE3 | <--- | AI自我效能 | 1.099 | .945 | 1.288 | .002 |
| SE4 | <--- | AI自我效能 | 1.281 | 1.110 | 1.482 | .002 |
| SE5 | <--- | AI自我效能 | 1.222 | 1.060 | 1.443 | .002 |
| AB1 | <--- | 使用行为 | 1.000 | 1.000 | 1.000 | ... |
| AB2 | <--- | 使用行为 | .976 | .867 | 1.106 | .002 |
| AB3 | <--- | 使用行为 | 1.068 | .955 | 1.203 | .002 |
| AB5 | <--- | 使用行为 | .969 | .854 | 1.119 | .002 |

##### Standardized Regression Weights: (other - Structural residuals)

| Parameter | | | Estimate | Lower | Upper | P |
| --- | --- | --- | --- | --- | --- | --- |
| AI自我效能 | <--- | AI威胁感知 | .483 | .392 | .572 | .002 |
| 防御性动机 | <--- | AI威胁感知 | .454 | .333 | .578 | .002 |
| 防御性动机 | <--- | AI自我效能 | .064 | -.067 | .199 | .375 |
| 使用行为 | <--- | 防御性动机 | .382 | .278 | .478 | .002 |
| 使用行为 | <--- | AI自我效能 | .386 | .277 | .479 | .002 |
| AA1 | <--- | AI威胁感知 | .811 | .764 | .857 | .002 |
| AA2 | <--- | AI威胁感知 | .789 | .732 | .835 | .002 |
| AA3 | <--- | AI威胁感知 | .760 | .706 | .811 | .002 |
| AA4 | <--- | AI威胁感知 | .742 | .680 | .800 | .002 |
| AA5 | <--- | AI威胁感知 | .768 | .706 | .823 | .002 |
| DM1 | <--- | 防御性动机 | .706 | .636 | .768 | .002 |
| DM2 | <--- | 防御性动机 | .729 | .662 | .793 | .002 |
| DM3 | <--- | 防御性动机 | .780 | .724 | .840 | .002 |
| DM5 | <--- | 防御性动机 | .683 | .613 | .752 | .002 |
| SE1 | <--- | AI自我效能 | .636 | .566 | .708 | .002 |
| SE2 | <--- | AI自我效能 | .714 | .648 | .774 | .002 |
| SE3 | <--- | AI自我效能 | .695 | .630 | .759 | .002 |
| SE4 | <--- | AI自我效能 | .800 | .747 | .849 | .002 |
| SE5 | <--- | AI自我效能 | .797 | .741 | .847 | .002 |
| AB1 | <--- | 使用行为 | .757 | .696 | .813 | .002 |
| AB2 | <--- | 使用行为 | .743 | .680 | .805 | .002 |
| AB3 | <--- | 使用行为 | .807 | .749 | .862 | .002 |
| AB5 | <--- | 使用行为 | .707 | .649 | .767 | .002 |

##### Variances: (other - Structural residuals)

| Parameter | | | Estimate | Lower | Upper | P |
| --- | --- | --- | --- | --- | --- | --- |
| AI威胁感知 |  |  | 2.187 | 1.799 | 2.554 | .002 |
| e21 |  |  | 1.010 | .757 | 1.326 | .002 |
| e23 |  |  | 1.287 | .957 | 1.619 | .002 |
| e22 |  |  | 1.179 | .895 | 1.451 | .002 |
| e1 |  |  | 1.139 | .881 | 1.387 | .002 |
| e2 |  |  | 1.209 | .949 | 1.517 | .002 |
| e3 |  |  | 1.355 | 1.100 | 1.620 | .002 |
| e4 |  |  | 1.513 | 1.192 | 1.833 | .002 |
| e5 |  |  | 1.339 | .992 | 1.694 | .002 |
| e6 |  |  | 1.700 | 1.356 | 2.071 | .002 |
| e8 |  |  | 1.572 | 1.214 | 1.940 | .002 |
| e9 |  |  | 1.195 | .896 | 1.446 | .002 |
| e10 |  |  | 1.852 | 1.484 | 2.210 | .002 |
| e11 |  |  | 1.942 | 1.575 | 2.257 | .002 |
| e12 |  |  | 1.716 | 1.355 | 2.048 | .002 |
| e13 |  |  | 1.702 | 1.341 | 2.026 | .002 |
| e14 |  |  | 1.213 | .922 | 1.493 | .002 |
| e15 |  |  | 1.132 | .855 | 1.416 | .002 |
| e16 |  |  | 1.415 | 1.110 | 1.697 | .002 |
| e17 |  |  | 1.462 | 1.142 | 1.784 | .002 |
| e18 |  |  | 1.158 | .833 | 1.477 | .002 |
| e20 |  |  | 1.775 | 1.425 | 2.100 | .002 |

##### Squared Multiple Correlations: (other - Structural residuals)

| Parameter | | | Estimate | Lower | Upper | P |
| --- | --- | --- | --- | --- | --- | --- |
| AI自我效能 |  |  | .233 | .154 | .327 | .002 |
| 防御性动机 |  |  | .238 | .160 | .350 | .002 |
| 使用行为 |  |  | .378 | .288 | .480 | .002 |
| AB5 |  |  | .500 | .421 | .588 | .002 |
| AB3 |  |  | .651 | .560 | .742 | .002 |
| AB2 |  |  | .553 | .462 | .648 | .002 |
| AB1 |  |  | .573 | .485 | .662 | .002 |
| SE5 |  |  | .635 | .549 | .717 | .002 |
| SE4 |  |  | .641 | .558 | .721 | .002 |
| SE3 |  |  | .483 | .397 | .576 | .002 |
| SE2 |  |  | .509 | .419 | .598 | .002 |
| SE1 |  |  | .404 | .320 | .502 | .002 |
| DM5 |  |  | .466 | .376 | .565 | .002 |
| DM3 |  |  | .609 | .524 | .705 | .002 |
| DM2 |  |  | .531 | .439 | .629 | .002 |
| DM1 |  |  | .498 | .405 | .589 | .002 |
| AA5 |  |  | .590 | .499 | .677 | .002 |
| AA4 |  |  | .550 | .462 | .640 | .002 |
| AA3 |  |  | .578 | .498 | .657 | .002 |
| AA2 |  |  | .622 | .537 | .698 | .002 |
| AA1 |  |  | .658 | .583 | .734 | .002 |

##### User-defined estimands: (other - Structural residuals)

| Parameter | | | Estimate | Lower | Upper | P |
| --- | --- | --- | --- | --- | --- | --- |
| diff1 |  |  | .000 | .000 | .000 | ... |
| diff2 |  |  | .024 | -.128 | .166 | .756 |
| diff3 |  |  | .088 | -.090 | .279 | .379 |
| diff4 |  |  | .029 | -.123 | .190 | .751 |
| diff5 |  |  | -.303 | -.478 | -.131 | .002 |

##### Bias-corrected percentile method (other - Structural residuals)

##### 95% confidence intervals (bias-corrected percentile method)

##### Scalar Estimates (other - Structural residuals)

##### Regression Weights: (other - Structural residuals)

| Parameter | | | Estimate | Lower | Upper | P |
| --- | --- | --- | --- | --- | --- | --- |
| AI自我效能 | <--- | AI威胁感知 | .375 | .282 | .467 | .003 |
| 防御性动机 | <--- | AI威胁感知 | .399 | .273 | .523 | .003 |
| 防御性动机 | <--- | AI自我效能 | .072 | -.086 | .217 | .408 |
| 使用行为 | <--- | 防御性动机 | .404 | .290 | .525 | .003 |
| 使用行为 | <--- | AI自我效能 | .463 | .332 | .616 | .001 |
| AA1 | <--- | AI威胁感知 | 1.000 | 1.000 | 1.000 | ... |
| AA2 | <--- | AI威胁感知 | .954 | .866 | 1.055 | .001 |
| AA3 | <--- | AI威胁感知 | .921 | .832 | 1.014 | .002 |
| AA4 | <--- | AI威胁感知 | .919 | .831 | 1.029 | .002 |
| AA5 | <--- | AI威胁感知 | .939 | .840 | 1.050 | .002 |
| DM1 | <--- | 防御性动机 | 1.000 | 1.000 | 1.000 | ... |
| DM2 | <--- | 防御性动机 | 1.027 | .884 | 1.168 | .003 |
| DM3 | <--- | 防御性动机 | 1.050 | .905 | 1.205 | .003 |
| DM5 | <--- | 防御性动机 | .979 | .839 | 1.135 | .002 |
| SE1 | <--- | AI自我效能 | 1.000 | 1.000 | 1.000 | ... |
| SE2 | <--- | AI自我效能 | 1.162 | 1.004 | 1.368 | .001 |
| SE3 | <--- | AI自我效能 | 1.099 | .951 | 1.293 | .001 |
| SE4 | <--- | AI自我效能 | 1.281 | 1.120 | 1.496 | .001 |
| SE5 | <--- | AI自我效能 | 1.222 | 1.066 | 1.456 | .001 |
| AB1 | <--- | 使用行为 | 1.000 | 1.000 | 1.000 | ... |
| AB2 | <--- | 使用行为 | .976 | .869 | 1.111 | .002 |
| AB3 | <--- | 使用行为 | 1.068 | .955 | 1.203 | .002 |
| AB5 | <--- | 使用行为 | .969 | .857 | 1.128 | .001 |

##### Standardized Regression Weights: (other - Structural residuals)

| Parameter | | | Estimate | Lower | Upper | P |
| --- | --- | --- | --- | --- | --- | --- |
| AI自我效能 | <--- | AI威胁感知 | .483 | .390 | .567 | .003 |
| 防御性动机 | <--- | AI威胁感知 | .454 | .324 | .571 | .003 |
| 防御性动机 | <--- | AI自我效能 | .064 | -.072 | .196 | .411 |
| 使用行为 | <--- | 防御性动机 | .382 | .275 | .478 | .002 |
| 使用行为 | <--- | AI自我效能 | .386 | .278 | .480 | .002 |
| AA1 | <--- | AI威胁感知 | .811 | .760 | .854 | .003 |
| AA2 | <--- | AI威胁感知 | .789 | .734 | .836 | .002 |
| AA3 | <--- | AI威胁感知 | .760 | .703 | .808 | .003 |
| AA4 | <--- | AI威胁感知 | .742 | .678 | .799 | .002 |
| AA5 | <--- | AI威胁感知 | .768 | .699 | .819 | .004 |
| DM1 | <--- | 防御性动机 | .706 | .640 | .770 | .001 |
| DM2 | <--- | 防御性动机 | .729 | .661 | .789 | .003 |
| DM3 | <--- | 防御性动机 | .780 | .711 | .831 | .005 |
| DM5 | <--- | 防御性动机 | .683 | .612 | .751 | .002 |
| SE1 | <--- | AI自我效能 | .636 | .560 | .704 | .003 |
| SE2 | <--- | AI自我效能 | .714 | .651 | .775 | .002 |
| SE3 | <--- | AI自我效能 | .695 | .620 | .752 | .005 |
| SE4 | <--- | AI自我效能 | .800 | .747 | .850 | .002 |
| SE5 | <--- | AI自我效能 | .797 | .739 | .842 | .004 |
| AB1 | <--- | 使用行为 | .757 | .688 | .806 | .004 |
| AB2 | <--- | 使用行为 | .743 | .677 | .801 | .003 |
| AB3 | <--- | 使用行为 | .807 | .747 | .857 | .003 |
| AB5 | <--- | 使用行为 | .707 | .645 | .764 | .003 |

##### Variances: (other - Structural residuals)

| Parameter | | | Estimate | Lower | Upper | P |
| --- | --- | --- | --- | --- | --- | --- |
| AI威胁感知 |  |  | 2.187 | 1.781 | 2.537 | .003 |
| e21 |  |  | 1.010 | .757 | 1.326 | .002 |
| e23 |  |  | 1.287 | 1.002 | 1.671 | .000 |
| e22 |  |  | 1.179 | .909 | 1.459 | .001 |
| e1 |  |  | 1.139 | .898 | 1.409 | .001 |
| e2 |  |  | 1.209 | .943 | 1.511 | .002 |
| e3 |  |  | 1.355 | 1.117 | 1.639 | .001 |
| e4 |  |  | 1.513 | 1.224 | 1.880 | .001 |
| e5 |  |  | 1.339 | 1.031 | 1.733 | .001 |
| e6 |  |  | 1.700 | 1.375 | 2.089 | .001 |
| e8 |  |  | 1.572 | 1.235 | 1.994 | .001 |
| e9 |  |  | 1.195 | .958 | 1.487 | .001 |
| e10 |  |  | 1.852 | 1.501 | 2.241 | .001 |
| e11 |  |  | 1.942 | 1.615 | 2.302 | .001 |
| e12 |  |  | 1.716 | 1.387 | 2.082 | .001 |
| e13 |  |  | 1.702 | 1.387 | 2.106 | .001 |
| e14 |  |  | 1.213 | .939 | 1.506 | .001 |
| e15 |  |  | 1.132 | .895 | 1.496 | .001 |
| e16 |  |  | 1.415 | 1.152 | 1.754 | .001 |
| e17 |  |  | 1.462 | 1.166 | 1.808 | .001 |
| e18 |  |  | 1.158 | .866 | 1.511 | .001 |
| e20 |  |  | 1.775 | 1.444 | 2.107 | .001 |

##### Squared Multiple Correlations: (other - Structural residuals)

| Parameter | | | Estimate | Lower | Upper | P |
| --- | --- | --- | --- | --- | --- | --- |
| AI自我效能 |  |  | .233 | .152 | .322 | .003 |
| 防御性动机 |  |  | .238 | .146 | .332 | .005 |
| 使用行为 |  |  | .378 | .286 | .475 | .003 |
| AB5 |  |  | .500 | .415 | .584 | .003 |
| AB3 |  |  | .651 | .559 | .734 | .003 |
| AB2 |  |  | .553 | .459 | .642 | .003 |
| AB1 |  |  | .573 | .474 | .650 | .004 |
| SE5 |  |  | .635 | .546 | .709 | .004 |
| SE4 |  |  | .641 | .558 | .722 | .002 |
| SE3 |  |  | .483 | .385 | .565 | .005 |
| SE2 |  |  | .509 | .423 | .600 | .002 |
| SE1 |  |  | .404 | .314 | .495 | .003 |
| DM5 |  |  | .466 | .375 | .564 | .002 |
| DM3 |  |  | .609 | .505 | .691 | .005 |
| DM2 |  |  | .531 | .436 | .623 | .003 |
| DM1 |  |  | .498 | .410 | .593 | .001 |
| AA5 |  |  | .590 | .488 | .671 | .004 |
| AA4 |  |  | .550 | .460 | .638 | .002 |
| AA3 |  |  | .578 | .494 | .654 | .003 |
| AA2 |  |  | .622 | .539 | .699 | .002 |
| AA1 |  |  | .658 | .578 | .729 | .003 |

##### User-defined estimands: (other - Structural residuals)

| Parameter | | | Estimate | Lower | Upper | P |
| --- | --- | --- | --- | --- | --- | --- |
| diff1 |  |  | .000 | .000 | .000 | ... |
| diff2 |  |  | .024 | -.131 | .165 | .772 |
| diff3 |  |  | .088 | -.084 | .294 | .312 |
| diff4 |  |  | .029 | -.124 | .188 | .771 |
| diff5 |  |  | -.303 | -.480 | -.134 | .002 |

##### Minimization History (Structural residuals)

| Iteration |  | Negative eigenvalues | Condition # | Smallest eigenvalue | Diameter | F | NTries | Ratio |
| --- | --- | --- | --- | --- | --- | --- | --- | --- |
| 0 | e | 12 |  | -.912 | 9999.000 | 3476.393 | 0 | 9999.000 |
| 1 | e | 2 |  | -.008 | 2.313 | 964.054 | 18 | .478 |
| 2 | e | 0 | 20.138 |  | 1.907 | 393.913 | 6 | .832 |
| 3 | e | 0 | 19.681 |  | .980 | 298.420 | 1 | .902 |
| 4 | e | 0 | 32.833 |  | .342 | 277.901 | 1 | 1.084 |
| 5 | e | 0 | 42.098 |  | .093 | 277.079 | 1 | 1.045 |
| 6 | e | 0 | 44.210 |  | .014 | 277.072 | 1 | 1.011 |
| 7 | e | 0 | 44.197 |  | .000 | 277.072 | 1 | 1.000 |

##### Bootstrap (Structural residuals)

##### Summary of Bootstrap Iterations (Structural residuals)

##### (Structural residuals)

| Iterations | Method 0 | Method 1 | Method 2 |
| --- | --- | --- | --- |
| 1 | 0 | 0 | 0 |
| 2 | 0 | 0 | 0 |
| 3 | 0 | 0 | 0 |
| 4 | 0 | 0 | 0 |
| 5 | 0 | 4 | 0 |
| 6 | 0 | 234 | 0 |
| 7 | 0 | 428 | 0 |
| 8 | 0 | 229 | 0 |
| 9 | 0 | 85 | 0 |
| 10 | 0 | 14 | 0 |
| 11 | 0 | 5 | 0 |
| 12 | 0 | 0 | 0 |
| 13 | 0 | 1 | 0 |
| 14 | 0 | 0 | 0 |
| 15 | 0 | 0 | 0 |
| 16 | 0 | 0 | 0 |
| 17 | 0 | 0 | 0 |
| 18 | 0 | 0 | 0 |
| 19 | 0 | 0 | 0 |
| Total | 0 | 1000 | 0 |

0 bootstrap samples were unused because of a singular covariance matrix.

0 bootstrap samples were unused because a solution was not found.

1000 usable bootstrap samples were obtained.

##### Bootstrap Distributions (Structural residuals)

##### ML discrepancy (implied vs sample) (Structural residuals)

|  |  |  |
| --- | --- | --- |
|  |  | \|-------------------- |
|  | 454.123 | \|\* |
|  | 475.756 | \|\*\* |
|  | 497.389 | \|\*\*\*\*\*\* |
|  | 519.022 | \|\*\*\*\*\*\*\*\* |
|  | 540.655 | \|\*\*\*\*\*\*\*\*\*\*\*\*\* |
|  | 562.288 | \|\*\*\*\*\*\*\*\*\*\*\*\*\*\*\*\*\*\*\* |
|  | 583.921 | \|\*\*\*\*\*\*\*\*\*\*\*\*\*\*\*\*\*\*\* |
| N = 1000 | 605.554 | \|\*\*\*\*\*\*\*\*\*\*\*\*\*\*\* |
| Mean = 578.900 | 627.187 | \|\*\*\*\*\*\*\*\*\*\* |
| S. e. = 1.527 | 648.820 | \|\*\*\*\*\*\*\*\* |
|  | 670.453 | \|\*\*\* |
|  | 692.086 | \|\* |
|  | 713.719 | \|\* |
|  | 735.353 | \|\* |
|  | 756.986 | \|\* |
|  |  | \|-------------------- |

##### ML discrepancy (implied vs pop) (Structural residuals)

|  |  |  |
| --- | --- | --- |
|  |  | \|-------------------- |
|  | 327.272 | \|\* |
|  | 334.183 | \|\*\* |
|  | 341.093 | \|\*\*\*\*\* |
|  | 348.004 | \|\*\*\*\*\*\*\*\*\*\* |
|  | 354.915 | \|\*\*\*\*\*\*\*\*\*\*\*\*\*\*\*\*\*\* |
|  | 361.826 | \|\*\*\*\*\*\*\*\*\*\*\*\*\*\*\*\*\*\* |
|  | 368.737 | \|\*\*\*\*\*\*\*\*\*\*\*\*\*\*\*\*\*\*\* |
| N = 1000 | 375.648 | \|\*\*\*\*\*\*\*\*\*\*\*\*\* |
| Mean = 365.717 | 382.559 | \|\*\*\*\*\*\*\*\*\* |
| S. e. = .480 | 389.470 | \|\*\*\*\*\* |
|  | 396.380 | \|\*\*\* |
|  | 403.291 | \|\*\* |
|  | 410.202 | \|\* |
|  | 417.113 | \| |
|  | 424.024 | \|\* |
|  |  | \|-------------------- |

##### K-L overoptimism (unstabilized) (Structural residuals)

|  |  |  |
| --- | --- | --- |
|  |  | \|-------------------- |
|  | -176.913 | \|\* |
|  | -122.442 | \|\* |
|  | -67.970 | \|\*\* |
|  | -13.498 | \|\*\*\*\*\* |
|  | 40.973 | \|\*\*\*\*\*\*\*\*\*\* |
|  | 95.445 | \|\*\*\*\*\*\*\*\*\*\*\*\*\*\* |
|  | 149.917 | \|\*\*\*\*\*\*\*\*\*\*\*\*\*\*\*\*\*\*\* |
| N = 1000 | 204.388 | \|\*\*\*\*\*\*\*\*\*\*\*\*\*\*\*\*\*\*\* |
| Mean = 179.389 | 258.860 | \|\*\*\*\*\*\*\*\*\*\*\*\*\*\* |
| S. e. = 3.637 | 313.332 | \|\*\*\*\*\*\*\*\*\*\*\* |
|  | 367.803 | \|\*\*\*\*\*\*\* |
|  | 422.275 | \|\*\* |
|  | 476.747 | \|\* |
|  | 531.218 | \|\* |
|  | 585.690 | \|\* |
|  |  | \|-------------------- |

##### K-L overoptimism (stabilized) (Structural residuals)

|  |  |  |
| --- | --- | --- |
|  |  | \|-------------------- |
|  | 45.660 | \|\* |
|  | 66.152 | \|\* |
|  | 86.645 | \|\*\* |
|  | 107.138 | \|\*\*\*\*\* |
|  | 127.631 | \|\*\*\*\*\*\*\*\*\*\* |
|  | 148.124 | \|\*\*\*\*\*\*\*\*\*\*\*\*\* |
|  | 168.617 | \|\*\*\*\*\*\*\*\*\*\*\*\*\*\*\*\*\*\*\*\* |
| N = 1000 | 189.109 | \|\*\*\*\*\*\*\*\*\*\*\*\*\*\*\*\* |
| Mean = 174.098 | 209.602 | \|\*\*\*\*\*\*\*\*\*\*\*\* |
| S. e. = 1.256 | 230.095 | \|\*\*\*\*\* |
|  | 250.588 | \|\*\*\* |
|  | 271.081 | \|\*\* |
|  | 291.574 | \|\* |
|  | 312.066 | \| |
|  | 332.559 | \|\* |
|  |  | \|-------------------- |

##### Measurement residuals (Measurement residuals)

##### Notes for Model (Measurement residuals)

##### Computation of degrees of freedom (Measurement residuals)

|  |  |
| --- | --- |
| Number of distinct sample moments: | 342 |
| Number of distinct parameters to be estimated: | 41 |
| Degrees of freedom (342 - 41): | 301 |

##### Result (Measurement residuals)

Minimum was achieved

Chi-square = 290.627

Degrees of freedom = 301

Probability level = .656

##### east (east - Measurement residuals)

##### Estimates (east - Measurement residuals)

##### Scalar Estimates (east - Measurement residuals)

##### Maximum Likelihood Estimates

##### Regression Weights: (east - Measurement residuals)

|  |  |  | Estimate | S.E. | C.R. | P | Label |
| --- | --- | --- | --- | --- | --- | --- | --- |
| AI自我效能 | <--- | AI威胁感知 | .376 | .049 | 7.604 | \*\*\* | H1a |
| 防御性动机 | <--- | AI威胁感知 | .399 | .062 | 6.455 | \*\*\* | H2a |
| 防御性动机 | <--- | AI自我效能 | .074 | .074 | 1.001 | .317 | H5a |
| 使用行为 | <--- | 防御性动机 | .406 | .065 | 6.275 | \*\*\* | H4a |
| 使用行为 | <--- | AI自我效能 | .463 | .074 | 6.279 | \*\*\* | H3a |
| AA1 | <--- | AI威胁感知 | 1.000 |  |  |  |  |
| AA2 | <--- | AI威胁感知 | .951 | .057 | 16.731 | \*\*\* | a1\_1 |
| AA3 | <--- | AI威胁感知 | .919 | .058 | 15.827 | \*\*\* | a2\_1 |
| AA4 | <--- | AI威胁感知 | .920 | .058 | 15.915 | \*\*\* | a3\_1 |
| AA5 | <--- | AI威胁感知 | .944 | .056 | 16.744 | \*\*\* | a4\_1 |
| DM1 | <--- | 防御性动机 | 1.000 |  |  |  |  |
| DM2 | <--- | 防御性动机 | 1.022 | .082 | 12.447 | \*\*\* | a5\_1 |
| DM3 | <--- | 防御性动机 | 1.048 | .082 | 12.720 | \*\*\* | a6\_1 |
| DM5 | <--- | 防御性动机 | .983 | .082 | 11.999 | \*\*\* | a7\_1 |
| SE1 | <--- | AI自我效能 | 1.000 |  |  |  |  |
| SE2 | <--- | AI自我效能 | 1.162 | .101 | 11.518 | \*\*\* | a8\_1 |
| SE3 | <--- | AI自我效能 | 1.098 | .097 | 11.315 | \*\*\* | a9\_1 |
| SE4 | <--- | AI自我效能 | 1.282 | .102 | 12.519 | \*\*\* | a10\_1 |
| SE5 | <--- | AI自我效能 | 1.223 | .099 | 12.377 | \*\*\* | a11\_1 |
| AB1 | <--- | 使用行为 | 1.000 |  |  |  |  |
| AB2 | <--- | 使用行为 | .975 | .071 | 13.721 | \*\*\* | a12\_1 |
| AB3 | <--- | 使用行为 | 1.067 | .072 | 14.927 | \*\*\* | a13\_1 |
| AB5 | <--- | 使用行为 | .965 | .071 | 13.526 | \*\*\* | a14\_1 |

##### Standardized Regression Weights: (east - Measurement residuals)

|  |  |  | Estimate |
| --- | --- | --- | --- |
| AI自我效能 | <--- | AI威胁感知 | .483 |
| 防御性动机 | <--- | AI威胁感知 | .454 |
| 防御性动机 | <--- | AI自我效能 | .065 |
| 使用行为 | <--- | 防御性动机 | .383 |
| 使用行为 | <--- | AI自我效能 | .386 |
| AA1 | <--- | AI威胁感知 | .818 |
| AA2 | <--- | AI威胁感知 | .782 |
| AA3 | <--- | AI威胁感知 | .748 |
| AA4 | <--- | AI威胁感知 | .752 |
| AA5 | <--- | AI威胁感知 | .782 |
| DM1 | <--- | 防御性动机 | .706 |
| DM2 | <--- | 防御性动机 | .739 |
| DM3 | <--- | 防御性动机 | .761 |
| DM5 | <--- | 防御性动机 | .706 |
| SE1 | <--- | AI自我效能 | .637 |
| SE2 | <--- | AI自我效能 | .715 |
| SE3 | <--- | AI自我效能 | .699 |
| SE4 | <--- | AI自我效能 | .805 |
| SE5 | <--- | AI自我效能 | .791 |
| AB1 | <--- | 使用行为 | .757 |
| AB2 | <--- | 使用行为 | .736 |
| AB3 | <--- | 使用行为 | .808 |
| AB5 | <--- | 使用行为 | .726 |

##### Variances: (east - Measurement residuals)

|  |  |  | Estimate | S.E. | C.R. | P | Label |
| --- | --- | --- | --- | --- | --- | --- | --- |
| AI威胁感知 |  |  | 2.183 | .232 | 9.389 | \*\*\* | vvv1\_1 |
| e21 |  |  | 1.010 | .157 | 6.427 | \*\*\* | vv1\_1 |
| e23 |  |  | 1.283 | .182 | 7.060 | \*\*\* | vv3\_1 |
| e22 |  |  | 1.173 | .156 | 7.534 | \*\*\* | vv2\_1 |
| e1 |  |  | 1.080 | .104 | 10.369 | \*\*\* | v1\_1 |
| e2 |  |  | 1.258 | .113 | 11.156 | \*\*\* | v2\_1 |
| e3 |  |  | 1.448 | .124 | 11.681 | \*\*\* | v3\_1 |
| e4 |  |  | 1.423 | .122 | 11.636 | \*\*\* | v4\_1 |
| e5 |  |  | 1.235 | .111 | 11.148 | \*\*\* | v5\_1 |
| e6 |  |  | 1.693 | .153 | 11.036 | \*\*\* | v6\_1 |
| e8 |  |  | 1.468 | .141 | 10.435 | \*\*\* | v7\_1 |
| e9 |  |  | 1.350 | .136 | 9.938 | \*\*\* | v8\_1 |
| e10 |  |  | 1.642 | .149 | 11.043 | \*\*\* | v9\_1 |
| e11 |  |  | 1.926 | .155 | 12.425 | \*\*\* | v10\_1 |
| e12 |  |  | 1.700 | .146 | 11.638 | \*\*\* | v11\_1 |
| e13 |  |  | 1.666 | .141 | 11.842 | \*\*\* | v12\_1 |
| e14 |  |  | 1.177 | .119 | 9.910 | \*\*\* | v13\_1 |
| e15 |  |  | 1.181 | .115 | 10.275 | \*\*\* | v14\_1 |
| e16 |  |  | 1.410 | .132 | 10.656 | \*\*\* | v15\_1 |
| e17 |  |  | 1.523 | .138 | 11.052 | \*\*\* | v16\_1 |
| e18 |  |  | 1.146 | .122 | 9.360 | \*\*\* | v17\_1 |
| e20 |  |  | 1.585 | .141 | 11.227 | \*\*\* | v18\_1 |

##### Squared Multiple Correlations: (east - Measurement residuals)

|  |  |  | Estimate |
| --- | --- | --- | --- |
| AI自我效能 |  |  | .234 |
| 防御性动机 |  |  | .239 |
| 使用行为 |  |  | .380 |
| AB5 |  |  | .526 |
| AB3 |  |  | .653 |
| AB2 |  |  | .542 |
| AB1 |  |  | .573 |
| SE5 |  |  | .625 |
| SE4 |  |  | .648 |
| SE3 |  |  | .488 |
| SE2 |  |  | .511 |
| SE1 |  |  | .406 |
| DM5 |  |  | .498 |
| DM3 |  |  | .579 |
| DM2 |  |  | .546 |
| DM1 |  |  | .499 |
| AA5 |  |  | .612 |
| AA4 |  |  | .565 |
| AA3 |  |  | .560 |
| AA2 |  |  | .611 |
| AA1 |  |  | .669 |

##### User-defined estimands: (east - Measurement residuals)

|  |  |  |  |
| --- | --- | --- | --- |
| diff1 |  |  | .000 |
| diff2 |  |  | .024 |
| diff3 |  |  | .087 |
| diff4 |  |  | .030 |
| diff5 |  |  | -.302 |

##### Modification Indices (east - Measurement residuals)

##### Covariances: (east - Measurement residuals)

|  |  |  | M.I. | Par Change |
| --- | --- | --- | --- | --- |
| e21 | <--> | AI威胁感知 | 4.796 | .306 |
| e13 | <--> | e16 | 6.615 | -.375 |
| e4 | <--> | e17 | 4.218 | .286 |
| e4 | <--> | e11 | 4.671 | .324 |
| e2 | <--> | e8 | 4.141 | -.271 |
| e2 | <--> | e3 | 4.145 | -.256 |
| e1 | <--> | e15 | 5.183 | .263 |

##### Variances: (east - Measurement residuals)

|  |  |  | M.I. | Par Change |
| --- | --- | --- | --- | --- |

##### Regression Weights: (east - Measurement residuals)

|  |  |  | M.I. | Par Change |
| --- | --- | --- | --- | --- |
| AI自我效能 | <--- | AI威胁感知 | 4.796 | .140 |
| SE5 | <--- | AA1 | 5.114 | .123 |

##### Bootstrap (east - Measurement residuals)

##### Bootstrap standard errors (east - Measurement residuals)

##### Scalar Estimates (east - Measurement residuals)

##### Regression Weights: (east - Measurement residuals)

| Parameter | | | SE | SE-SE | Mean | Bias | SE-Bias |
| --- | --- | --- | --- | --- | --- | --- | --- |
| AI自我效能 | <--- | AI威胁感知 | .047 | .001 | .379 | .003 | .002 |
| 防御性动机 | <--- | AI威胁感知 | .063 | .001 | .401 | .002 | .002 |
| 防御性动机 | <--- | AI自我效能 | .077 | .002 | .076 | .002 | .002 |
| 使用行为 | <--- | 防御性动机 | .061 | .001 | .409 | .003 | .002 |
| 使用行为 | <--- | AI自我效能 | .073 | .002 | .461 | -.002 | .002 |
| AA1 | <--- | AI威胁感知 | .000 | .000 | 1.000 | .000 | .000 |
| AA2 | <--- | AI威胁感知 | .048 | .001 | .951 | -.001 | .002 |
| AA3 | <--- | AI威胁感知 | .046 | .001 | .921 | .002 | .001 |
| AA4 | <--- | AI威胁感知 | .052 | .001 | .920 | .000 | .002 |
| AA5 | <--- | AI威胁感知 | .052 | .001 | .945 | .001 | .002 |
| DM1 | <--- | 防御性动机 | .000 | .000 | 1.000 | .000 | .000 |
| DM2 | <--- | 防御性动机 | .073 | .002 | 1.028 | .006 | .002 |
| DM3 | <--- | 防御性动机 | .075 | .002 | 1.056 | .007 | .002 |
| DM5 | <--- | 防御性动机 | .078 | .002 | .988 | .005 | .002 |
| SE1 | <--- | AI自我效能 | .000 | .000 | 1.000 | .000 | .000 |
| SE2 | <--- | AI自我效能 | .092 | .002 | 1.157 | -.005 | .003 |
| SE3 | <--- | AI自我效能 | .088 | .002 | 1.100 | .002 | .003 |
| SE4 | <--- | AI自我效能 | .096 | .002 | 1.279 | -.002 | .003 |
| SE5 | <--- | AI自我效能 | .093 | .002 | 1.223 | .000 | .003 |
| AB1 | <--- | 使用行为 | .000 | .000 | 1.000 | .000 | .000 |
| AB2 | <--- | 使用行为 | .060 | .001 | .977 | .002 | .002 |
| AB3 | <--- | 使用行为 | .063 | .001 | 1.071 | .003 | .002 |
| AB5 | <--- | 使用行为 | .066 | .001 | .966 | .002 | .002 |

##### Standardized Regression Weights: (east - Measurement residuals)

| Parameter | | | SE | SE-SE | Mean | Bias | SE-Bias |
| --- | --- | --- | --- | --- | --- | --- | --- |
| AI自我效能 | <--- | AI威胁感知 | .045 | .001 | .484 | .001 | .001 |
| 防御性动机 | <--- | AI威胁感知 | .063 | .001 | .457 | .003 | .002 |
| 防御性动机 | <--- | AI自我效能 | .068 | .002 | .067 | .002 | .002 |
| 使用行为 | <--- | 防御性动机 | .053 | .001 | .384 | .001 | .002 |
| 使用行为 | <--- | AI自我效能 | .052 | .001 | .384 | -.002 | .002 |
| AA1 | <--- | AI威胁感知 | .023 | .001 | .817 | .000 | .001 |
| AA2 | <--- | AI威胁感知 | .023 | .001 | .780 | -.001 | .001 |
| AA3 | <--- | AI威胁感知 | .025 | .001 | .749 | .001 | .001 |
| AA4 | <--- | AI威胁感知 | .027 | .001 | .751 | -.001 | .001 |
| AA5 | <--- | AI威胁感知 | .026 | .001 | .783 | .001 | .001 |
| DM1 | <--- | 防御性动机 | .033 | .001 | .704 | -.002 | .001 |
| DM2 | <--- | 防御性动机 | .031 | .001 | .739 | .000 | .001 |
| DM3 | <--- | 防御性动机 | .029 | .001 | .762 | .001 | .001 |
| DM5 | <--- | 防御性动机 | .033 | .001 | .706 | .000 | .001 |
| SE1 | <--- | AI自我效能 | .035 | .001 | .639 | .002 | .001 |
| SE2 | <--- | AI自我效能 | .033 | .001 | .714 | -.002 | .001 |
| SE3 | <--- | AI自我效能 | .031 | .001 | .701 | .002 | .001 |
| SE4 | <--- | AI自我效能 | .024 | .001 | .804 | -.001 | .001 |
| SE5 | <--- | AI自我效能 | .025 | .001 | .792 | .001 | .001 |
| AB1 | <--- | 使用行为 | .028 | .001 | .758 | .001 | .001 |
| AB2 | <--- | 使用行为 | .030 | .001 | .737 | .001 | .001 |
| AB3 | <--- | 使用行为 | .024 | .001 | .809 | .001 | .001 |
| AB5 | <--- | 使用行为 | .030 | .001 | .726 | .000 | .001 |

##### Variances: (east - Measurement residuals)

| Parameter | | | SE | SE-SE | Mean | Bias | SE-Bias |
| --- | --- | --- | --- | --- | --- | --- | --- |
| AI威胁感知 |  |  | .189 | .004 | 2.179 | -.003 | .006 |
| e21 |  |  | .144 | .003 | 1.015 | .005 | .005 |
| e23 |  |  | .165 | .004 | 1.258 | -.025 | .005 |
| e22 |  |  | .139 | .003 | 1.165 | -.009 | .004 |
| e1 |  |  | .108 | .002 | 1.076 | -.004 | .003 |
| e2 |  |  | .108 | .002 | 1.255 | -.002 | .003 |
| e3 |  |  | .113 | .003 | 1.433 | -.015 | .004 |
| e4 |  |  | .123 | .003 | 1.414 | -.009 | .004 |
| e5 |  |  | .123 | .003 | 1.221 | -.014 | .004 |
| e6 |  |  | .151 | .003 | 1.689 | -.005 | .005 |
| e8 |  |  | .142 | .003 | 1.455 | -.013 | .004 |
| e9 |  |  | .125 | .003 | 1.333 | -.017 | .004 |
| e10 |  |  | .138 | .003 | 1.628 | -.014 | .004 |
| e11 |  |  | .134 | .003 | 1.907 | -.019 | .004 |
| e12 |  |  | .160 | .004 | 1.690 | -.010 | .005 |
| e13 |  |  | .134 | .003 | 1.644 | -.021 | .004 |
| e14 |  |  | .116 | .003 | 1.170 | -.007 | .004 |
| e15 |  |  | .120 | .003 | 1.165 | -.016 | .004 |
| e16 |  |  | .124 | .003 | 1.392 | -.018 | .004 |
| e17 |  |  | .143 | .003 | 1.509 | -.014 | .005 |
| e18 |  |  | .124 | .003 | 1.134 | -.012 | .004 |
| e20 |  |  | .137 | .003 | 1.571 | -.014 | .004 |

##### Squared Multiple Correlations: (east - Measurement residuals)

| Parameter | | | SE | SE-SE | Mean | Bias | SE-Bias |
| --- | --- | --- | --- | --- | --- | --- | --- |
| AI自我效能 |  |  | .043 | .001 | .236 | .003 | .001 |
| 防御性动机 |  |  | .048 | .001 | .249 | .010 | .002 |
| 使用行为 |  |  | .050 | .001 | .384 | .004 | .002 |
| AB5 |  |  | .043 | .001 | .528 | .001 | .001 |
| AB3 |  |  | .039 | .001 | .655 | .002 | .001 |
| AB2 |  |  | .044 | .001 | .544 | .002 | .001 |
| AB1 |  |  | .042 | .001 | .575 | .002 | .001 |
| SE5 |  |  | .040 | .001 | .628 | .002 | .001 |
| SE4 |  |  | .038 | .001 | .647 | .000 | .001 |
| SE3 |  |  | .043 | .001 | .492 | .004 | .001 |
| SE2 |  |  | .047 | .001 | .510 | -.001 | .001 |
| SE1 |  |  | .045 | .001 | .410 | .004 | .001 |
| DM5 |  |  | .047 | .001 | .499 | .001 | .001 |
| DM3 |  |  | .043 | .001 | .581 | .003 | .001 |
| DM2 |  |  | .045 | .001 | .547 | .001 | .001 |
| DM1 |  |  | .046 | .001 | .497 | -.002 | .001 |
| AA5 |  |  | .041 | .001 | .613 | .002 | .001 |
| AA4 |  |  | .040 | .001 | .565 | .000 | .001 |
| AA3 |  |  | .037 | .001 | .562 | .002 | .001 |
| AA2 |  |  | .036 | .001 | .610 | -.001 | .001 |
| AA1 |  |  | .037 | .001 | .669 | .000 | .001 |

##### User-defined estimands: (east - Measurement residuals)

| Parameter | | | SE | SE-SE | Mean | Bias | SE-Bias |
| --- | --- | --- | --- | --- | --- | --- | --- |
| diff1 |  |  | .000 | .000 | .000 | .000 | .000 |
| diff2 |  |  | .076 | .002 | .022 | -.001 | .002 |
| diff3 |  |  | .095 | .002 | .082 | -.005 | .003 |
| diff4 |  |  | .077 | .002 | .030 | .000 | .002 |
| diff5 |  |  | .092 | .002 | -.303 | -.002 | .003 |

##### Bootstrap Confidence (east - Measurement residuals)

##### Percentile method (east - Measurement residuals)

##### 95% confidence intervals (percentile method)

##### Scalar Estimates (east - Measurement residuals)

##### Regression Weights: (east - Measurement residuals)

| Parameter | | | Estimate | Lower | Upper | P |
| --- | --- | --- | --- | --- | --- | --- |
| AI自我效能 | <--- | AI威胁感知 | .376 | .288 | .473 | .002 |
| 防御性动机 | <--- | AI威胁感知 | .399 | .282 | .530 | .002 |
| 防御性动机 | <--- | AI自我效能 | .074 | -.077 | .222 | .343 |
| 使用行为 | <--- | 防御性动机 | .406 | .293 | .529 | .002 |
| 使用行为 | <--- | AI自我效能 | .463 | .326 | .607 | .002 |
| AA1 | <--- | AI威胁感知 | 1.000 | 1.000 | 1.000 | ... |
| AA2 | <--- | AI威胁感知 | .951 | .863 | 1.047 | .002 |
| AA3 | <--- | AI威胁感知 | .919 | .830 | 1.016 | .002 |
| AA4 | <--- | AI威胁感知 | .920 | .828 | 1.030 | .002 |
| AA5 | <--- | AI威胁感知 | .944 | .847 | 1.059 | .002 |
| DM1 | <--- | 防御性动机 | 1.000 | 1.000 | 1.000 | ... |
| DM2 | <--- | 防御性动机 | 1.022 | .887 | 1.172 | .002 |
| DM3 | <--- | 防御性动机 | 1.048 | .911 | 1.207 | .002 |
| DM5 | <--- | 防御性动机 | .983 | .846 | 1.142 | .002 |
| SE1 | <--- | AI自我效能 | 1.000 | 1.000 | 1.000 | ... |
| SE2 | <--- | AI自我效能 | 1.162 | .979 | 1.348 | .002 |
| SE3 | <--- | AI自我效能 | 1.098 | .946 | 1.282 | .002 |
| SE4 | <--- | AI自我效能 | 1.282 | 1.112 | 1.481 | .002 |
| SE5 | <--- | AI自我效能 | 1.223 | 1.058 | 1.446 | .002 |
| AB1 | <--- | 使用行为 | 1.000 | 1.000 | 1.000 | ... |
| AB2 | <--- | 使用行为 | .975 | .864 | 1.108 | .002 |
| AB3 | <--- | 使用行为 | 1.067 | .955 | 1.209 | .002 |
| AB5 | <--- | 使用行为 | .965 | .844 | 1.113 | .002 |

##### Standardized Regression Weights: (east - Measurement residuals)

| Parameter | | | Estimate | Lower | Upper | P |
| --- | --- | --- | --- | --- | --- | --- |
| AI自我效能 | <--- | AI威胁感知 | .483 | .392 | .571 | .002 |
| 防御性动机 | <--- | AI威胁感知 | .454 | .336 | .577 | .002 |
| 防御性动机 | <--- | AI自我效能 | .065 | -.067 | .197 | .343 |
| 使用行为 | <--- | 防御性动机 | .383 | .274 | .482 | .002 |
| 使用行为 | <--- | AI自我效能 | .386 | .277 | .482 | .002 |
| AA1 | <--- | AI威胁感知 | .818 | .772 | .859 | .002 |
| AA2 | <--- | AI威胁感知 | .782 | .732 | .821 | .002 |
| AA3 | <--- | AI威胁感知 | .748 | .697 | .795 | .002 |
| AA4 | <--- | AI威胁感知 | .752 | .695 | .804 | .002 |
| AA5 | <--- | AI威胁感知 | .782 | .730 | .828 | .002 |
| DM1 | <--- | 防御性动机 | .706 | .638 | .764 | .002 |
| DM2 | <--- | 防御性动机 | .739 | .677 | .799 | .002 |
| DM3 | <--- | 防御性动机 | .761 | .703 | .817 | .002 |
| DM5 | <--- | 防御性动机 | .706 | .638 | .766 | .002 |
| SE1 | <--- | AI自我效能 | .637 | .567 | .706 | .002 |
| SE2 | <--- | AI自我效能 | .715 | .646 | .776 | .002 |
| SE3 | <--- | AI自我效能 | .699 | .637 | .761 | .002 |
| SE4 | <--- | AI自我效能 | .805 | .752 | .850 | .002 |
| SE5 | <--- | AI自我效能 | .791 | .739 | .838 | .002 |
| AB1 | <--- | 使用行为 | .757 | .699 | .812 | .002 |
| AB2 | <--- | 使用行为 | .736 | .674 | .793 | .002 |
| AB3 | <--- | 使用行为 | .808 | .759 | .856 | .002 |
| AB5 | <--- | 使用行为 | .726 | .664 | .784 | .002 |

##### Variances: (east - Measurement residuals)

| Parameter | | | Estimate | Lower | Upper | P |
| --- | --- | --- | --- | --- | --- | --- |
| AI威胁感知 |  |  | 2.183 | 1.786 | 2.547 | .002 |
| e21 |  |  | 1.010 | .756 | 1.311 | .002 |
| e23 |  |  | 1.283 | .953 | 1.597 | .002 |
| e22 |  |  | 1.173 | .889 | 1.434 | .002 |
| e1 |  |  | 1.080 | .862 | 1.272 | .002 |
| e2 |  |  | 1.258 | 1.053 | 1.468 | .002 |
| e3 |  |  | 1.448 | 1.224 | 1.679 | .002 |
| e4 |  |  | 1.423 | 1.172 | 1.661 | .002 |
| e5 |  |  | 1.235 | .998 | 1.478 | .002 |
| e6 |  |  | 1.693 | 1.388 | 1.997 | .002 |
| e8 |  |  | 1.468 | 1.168 | 1.741 | .002 |
| e9 |  |  | 1.350 | 1.070 | 1.599 | .002 |
| e10 |  |  | 1.642 | 1.365 | 1.901 | .002 |
| e11 |  |  | 1.926 | 1.647 | 2.169 | .002 |
| e12 |  |  | 1.700 | 1.365 | 1.996 | .002 |
| e13 |  |  | 1.666 | 1.391 | 1.911 | .002 |
| e14 |  |  | 1.177 | .948 | 1.387 | .002 |
| e15 |  |  | 1.181 | .927 | 1.406 | .002 |
| e16 |  |  | 1.410 | 1.153 | 1.646 | .002 |
| e17 |  |  | 1.523 | 1.236 | 1.814 | .002 |
| e18 |  |  | 1.146 | .900 | 1.382 | .002 |
| e20 |  |  | 1.585 | 1.310 | 1.836 | .002 |

##### Squared Multiple Correlations: (east - Measurement residuals)

| Parameter | | | Estimate | Lower | Upper | P |
| --- | --- | --- | --- | --- | --- | --- |
| AI自我效能 |  |  | .234 | .153 | .326 | .002 |
| 防御性动机 |  |  | .239 | .158 | .349 | .002 |
| 使用行为 |  |  | .380 | .291 | .483 | .002 |
| AB5 |  |  | .526 | .441 | .615 | .002 |
| AB3 |  |  | .653 | .576 | .733 | .002 |
| AB2 |  |  | .542 | .454 | .629 | .002 |
| AB1 |  |  | .573 | .488 | .659 | .002 |
| SE5 |  |  | .625 | .546 | .703 | .002 |
| SE4 |  |  | .648 | .565 | .722 | .002 |
| SE3 |  |  | .488 | .406 | .579 | .002 |
| SE2 |  |  | .511 | .418 | .603 | .002 |
| SE1 |  |  | .406 | .322 | .499 | .002 |
| DM5 |  |  | .498 | .407 | .587 | .002 |
| DM3 |  |  | .579 | .494 | .668 | .002 |
| DM2 |  |  | .546 | .459 | .638 | .002 |
| DM1 |  |  | .499 | .407 | .583 | .002 |
| AA5 |  |  | .612 | .533 | .686 | .002 |
| AA4 |  |  | .565 | .483 | .647 | .002 |
| AA3 |  |  | .560 | .486 | .632 | .002 |
| AA2 |  |  | .611 | .536 | .674 | .002 |
| AA1 |  |  | .669 | .596 | .738 | .002 |

##### User-defined estimands: (east - Measurement residuals)

| Parameter | | | Estimate | Lower | Upper | P |
| --- | --- | --- | --- | --- | --- | --- |
| diff1 |  |  | .000 | .000 | .000 | ... |
| diff2 |  |  | .024 | -.126 | .166 | .767 |
| diff3 |  |  | .087 | -.088 | .272 | .373 |
| diff4 |  |  | .030 | -.121 | .190 | .735 |
| diff5 |  |  | -.302 | -.481 | -.130 | .002 |

##### Bias-corrected percentile method (east - Measurement residuals)

##### 95% confidence intervals (bias-corrected percentile method)

##### Scalar Estimates (east - Measurement residuals)

##### Regression Weights: (east - Measurement residuals)

| Parameter | | | Estimate | Lower | Upper | P |
| --- | --- | --- | --- | --- | --- | --- |
| AI自我效能 | <--- | AI威胁感知 | .376 | .282 | .465 | .003 |
| 防御性动机 | <--- | AI威胁感知 | .399 | .279 | .528 | .002 |
| 防御性动机 | <--- | AI自我效能 | .074 | -.079 | .214 | .377 |
| 使用行为 | <--- | 防御性动机 | .406 | .287 | .527 | .003 |
| 使用行为 | <--- | AI自我效能 | .463 | .327 | .608 | .002 |
| AA1 | <--- | AI威胁感知 | 1.000 | 1.000 | 1.000 | ... |
| AA2 | <--- | AI威胁感知 | .951 | .865 | 1.052 | .002 |
| AA3 | <--- | AI威胁感知 | .919 | .830 | 1.016 | .002 |
| AA4 | <--- | AI威胁感知 | .920 | .830 | 1.034 | .002 |
| AA5 | <--- | AI威胁感知 | .944 | .851 | 1.069 | .002 |
| DM1 | <--- | 防御性动机 | 1.000 | 1.000 | 1.000 | ... |
| DM2 | <--- | 防御性动机 | 1.022 | .876 | 1.158 | .003 |
| DM3 | <--- | 防御性动机 | 1.048 | .894 | 1.198 | .003 |
| DM5 | <--- | 防御性动机 | .983 | .841 | 1.138 | .002 |
| SE1 | <--- | AI自我效能 | 1.000 | 1.000 | 1.000 | ... |
| SE2 | <--- | AI自我效能 | 1.162 | 1.009 | 1.372 | .001 |
| SE3 | <--- | AI自我效能 | 1.098 | .948 | 1.287 | .002 |
| SE4 | <--- | AI自我效能 | 1.282 | 1.124 | 1.497 | .001 |
| SE5 | <--- | AI自我效能 | 1.223 | 1.064 | 1.454 | .001 |
| AB1 | <--- | 使用行为 | 1.000 | 1.000 | 1.000 | ... |
| AB2 | <--- | 使用行为 | .975 | .867 | 1.114 | .002 |
| AB3 | <--- | 使用行为 | 1.067 | .955 | 1.210 | .002 |
| AB5 | <--- | 使用行为 | .965 | .853 | 1.125 | .001 |

##### Standardized Regression Weights: (east - Measurement residuals)

| Parameter | | | Estimate | Lower | Upper | P |
| --- | --- | --- | --- | --- | --- | --- |
| AI自我效能 | <--- | AI威胁感知 | .483 | .388 | .566 | .003 |
| 防御性动机 | <--- | AI威胁感知 | .454 | .323 | .571 | .003 |
| 防御性动机 | <--- | AI自我效能 | .065 | -.070 | .195 | .366 |
| 使用行为 | <--- | 防御性动机 | .383 | .274 | .481 | .002 |
| 使用行为 | <--- | AI自我效能 | .386 | .277 | .482 | .002 |
| AA1 | <--- | AI威胁感知 | .818 | .772 | .858 | .002 |
| AA2 | <--- | AI威胁感知 | .782 | .731 | .820 | .003 |
| AA3 | <--- | AI威胁感知 | .748 | .694 | .794 | .003 |
| AA4 | <--- | AI威胁感知 | .752 | .694 | .804 | .002 |
| AA5 | <--- | AI威胁感知 | .782 | .722 | .825 | .004 |
| DM1 | <--- | 防御性动机 | .706 | .646 | .770 | .001 |
| DM2 | <--- | 防御性动机 | .739 | .673 | .795 | .003 |
| DM3 | <--- | 防御性动机 | .761 | .696 | .810 | .003 |
| DM5 | <--- | 防御性动机 | .706 | .636 | .765 | .002 |
| SE1 | <--- | AI自我效能 | .637 | .560 | .702 | .004 |
| SE2 | <--- | AI自我效能 | .715 | .647 | .778 | .002 |
| SE3 | <--- | AI自我效能 | .699 | .627 | .752 | .004 |
| SE4 | <--- | AI自我效能 | .805 | .752 | .850 | .002 |
| SE5 | <--- | AI自我效能 | .791 | .736 | .834 | .004 |
| AB1 | <--- | 使用行为 | .757 | .692 | .807 | .004 |
| AB2 | <--- | 使用行为 | .736 | .671 | .789 | .003 |
| AB3 | <--- | 使用行为 | .808 | .756 | .853 | .003 |
| AB5 | <--- | 使用行为 | .726 | .663 | .782 | .003 |

##### Variances: (east - Measurement residuals)

| Parameter | | | Estimate | Lower | Upper | P |
| --- | --- | --- | --- | --- | --- | --- |
| AI威胁感知 |  |  | 2.183 | 1.784 | 2.544 | .002 |
| e21 |  |  | 1.010 | .757 | 1.311 | .002 |
| e23 |  |  | 1.283 | 1.001 | 1.665 | .000 |
| e22 |  |  | 1.173 | .913 | 1.467 | .001 |
| e1 |  |  | 1.080 | .866 | 1.273 | .002 |
| e2 |  |  | 1.258 | 1.057 | 1.469 | .002 |
| e3 |  |  | 1.448 | 1.260 | 1.723 | .001 |
| e4 |  |  | 1.423 | 1.196 | 1.701 | .001 |
| e5 |  |  | 1.235 | 1.024 | 1.508 | .001 |
| e6 |  |  | 1.693 | 1.406 | 2.013 | .001 |
| e8 |  |  | 1.468 | 1.222 | 1.783 | .001 |
| e9 |  |  | 1.350 | 1.113 | 1.636 | .001 |
| e10 |  |  | 1.642 | 1.386 | 1.915 | .001 |
| e11 |  |  | 1.926 | 1.683 | 2.233 | .001 |
| e12 |  |  | 1.700 | 1.392 | 2.010 | .001 |
| e13 |  |  | 1.666 | 1.424 | 1.948 | .001 |
| e14 |  |  | 1.177 | .961 | 1.392 | .001 |
| e15 |  |  | 1.181 | .975 | 1.427 | .001 |
| e16 |  |  | 1.410 | 1.184 | 1.707 | .001 |
| e17 |  |  | 1.523 | 1.263 | 1.841 | .001 |
| e18 |  |  | 1.146 | .929 | 1.414 | .001 |
| e20 |  |  | 1.585 | 1.340 | 1.868 | .001 |

##### Squared Multiple Correlations: (east - Measurement residuals)

| Parameter | | | Estimate | Lower | Upper | P |
| --- | --- | --- | --- | --- | --- | --- |
| AI自我效能 |  |  | .234 | .151 | .320 | .003 |
| 防御性动机 |  |  | .239 | .146 | .332 | .005 |
| 使用行为 |  |  | .380 | .286 | .478 | .003 |
| AB5 |  |  | .526 | .439 | .611 | .003 |
| AB3 |  |  | .653 | .572 | .728 | .003 |
| AB2 |  |  | .542 | .451 | .622 | .003 |
| AB1 |  |  | .573 | .479 | .651 | .004 |
| SE5 |  |  | .625 | .541 | .696 | .004 |
| SE4 |  |  | .648 | .565 | .722 | .002 |
| SE3 |  |  | .488 | .393 | .566 | .004 |
| SE2 |  |  | .511 | .419 | .605 | .002 |
| SE1 |  |  | .406 | .313 | .493 | .004 |
| DM5 |  |  | .498 | .404 | .586 | .002 |
| DM3 |  |  | .579 | .485 | .657 | .003 |
| DM2 |  |  | .546 | .453 | .632 | .003 |
| DM1 |  |  | .499 | .417 | .593 | .001 |
| AA5 |  |  | .612 | .521 | .680 | .004 |
| AA4 |  |  | .565 | .482 | .646 | .002 |
| AA3 |  |  | .560 | .482 | .630 | .003 |
| AA2 |  |  | .611 | .535 | .673 | .003 |
| AA1 |  |  | .669 | .595 | .736 | .002 |

##### User-defined estimands: (east - Measurement residuals)

| Parameter | | | Estimate | Lower | Upper | P |
| --- | --- | --- | --- | --- | --- | --- |
| diff1 |  |  | .000 | .000 | .000 | ... |
| diff2 |  |  | .024 | -.129 | .164 | .797 |
| diff3 |  |  | .087 | -.082 | .285 | .321 |
| diff4 |  |  | .030 | -.122 | .187 | .754 |
| diff5 |  |  | -.302 | -.484 | -.132 | .002 |

##### other (other - Measurement residuals)

##### Estimates (other - Measurement residuals)

##### Scalar Estimates (other - Measurement residuals)

##### Maximum Likelihood Estimates

##### Regression Weights: (other - Measurement residuals)

|  |  |  | Estimate | S.E. | C.R. | P | Label |
| --- | --- | --- | --- | --- | --- | --- | --- |
| AI自我效能 | <--- | AI威胁感知 | .376 | .049 | 7.604 | \*\*\* | H1a |
| 防御性动机 | <--- | AI威胁感知 | .399 | .062 | 6.455 | \*\*\* | H2a |
| 防御性动机 | <--- | AI自我效能 | .074 | .074 | 1.001 | .317 | H5a |
| 使用行为 | <--- | 防御性动机 | .406 | .065 | 6.275 | \*\*\* | H4a |
| 使用行为 | <--- | AI自我效能 | .463 | .074 | 6.279 | \*\*\* | H3a |
| AA1 | <--- | AI威胁感知 | 1.000 |  |  |  |  |
| AA2 | <--- | AI威胁感知 | .951 | .057 | 16.731 | \*\*\* | a1\_1 |
| AA3 | <--- | AI威胁感知 | .919 | .058 | 15.827 | \*\*\* | a2\_1 |
| AA4 | <--- | AI威胁感知 | .920 | .058 | 15.915 | \*\*\* | a3\_1 |
| AA5 | <--- | AI威胁感知 | .944 | .056 | 16.744 | \*\*\* | a4\_1 |
| DM1 | <--- | 防御性动机 | 1.000 |  |  |  |  |
| DM2 | <--- | 防御性动机 | 1.022 | .082 | 12.447 | \*\*\* | a5\_1 |
| DM3 | <--- | 防御性动机 | 1.048 | .082 | 12.720 | \*\*\* | a6\_1 |
| DM5 | <--- | 防御性动机 | .983 | .082 | 11.999 | \*\*\* | a7\_1 |
| SE1 | <--- | AI自我效能 | 1.000 |  |  |  |  |
| SE2 | <--- | AI自我效能 | 1.162 | .101 | 11.518 | \*\*\* | a8\_1 |
| SE3 | <--- | AI自我效能 | 1.098 | .097 | 11.315 | \*\*\* | a9\_1 |
| SE4 | <--- | AI自我效能 | 1.282 | .102 | 12.519 | \*\*\* | a10\_1 |
| SE5 | <--- | AI自我效能 | 1.223 | .099 | 12.377 | \*\*\* | a11\_1 |
| AB1 | <--- | 使用行为 | 1.000 |  |  |  |  |
| AB2 | <--- | 使用行为 | .975 | .071 | 13.721 | \*\*\* | a12\_1 |
| AB3 | <--- | 使用行为 | 1.067 | .072 | 14.927 | \*\*\* | a13\_1 |
| AB5 | <--- | 使用行为 | .965 | .071 | 13.526 | \*\*\* | a14\_1 |

##### Standardized Regression Weights: (other - Measurement residuals)

|  |  |  | Estimate |
| --- | --- | --- | --- |
| AI自我效能 | <--- | AI威胁感知 | .483 |
| 防御性动机 | <--- | AI威胁感知 | .454 |
| 防御性动机 | <--- | AI自我效能 | .065 |
| 使用行为 | <--- | 防御性动机 | .383 |
| 使用行为 | <--- | AI自我效能 | .386 |
| AA1 | <--- | AI威胁感知 | .818 |
| AA2 | <--- | AI威胁感知 | .782 |
| AA3 | <--- | AI威胁感知 | .748 |
| AA4 | <--- | AI威胁感知 | .752 |
| AA5 | <--- | AI威胁感知 | .782 |
| DM1 | <--- | 防御性动机 | .706 |
| DM2 | <--- | 防御性动机 | .739 |
| DM3 | <--- | 防御性动机 | .761 |
| DM5 | <--- | 防御性动机 | .706 |
| SE1 | <--- | AI自我效能 | .637 |
| SE2 | <--- | AI自我效能 | .715 |
| SE3 | <--- | AI自我效能 | .699 |
| SE4 | <--- | AI自我效能 | .805 |
| SE5 | <--- | AI自我效能 | .791 |
| AB1 | <--- | 使用行为 | .757 |
| AB2 | <--- | 使用行为 | .736 |
| AB3 | <--- | 使用行为 | .808 |
| AB5 | <--- | 使用行为 | .726 |

##### Variances: (other - Measurement residuals)

|  |  |  | Estimate | S.E. | C.R. | P | Label |
| --- | --- | --- | --- | --- | --- | --- | --- |
| AI威胁感知 |  |  | 2.183 | .232 | 9.389 | \*\*\* | vvv1\_1 |
| e21 |  |  | 1.010 | .157 | 6.427 | \*\*\* | vv1\_1 |
| e23 |  |  | 1.283 | .182 | 7.060 | \*\*\* | vv3\_1 |
| e22 |  |  | 1.173 | .156 | 7.534 | \*\*\* | vv2\_1 |
| e1 |  |  | 1.080 | .104 | 10.369 | \*\*\* | v1\_1 |
| e2 |  |  | 1.258 | .113 | 11.156 | \*\*\* | v2\_1 |
| e3 |  |  | 1.448 | .124 | 11.681 | \*\*\* | v3\_1 |
| e4 |  |  | 1.423 | .122 | 11.636 | \*\*\* | v4\_1 |
| e5 |  |  | 1.235 | .111 | 11.148 | \*\*\* | v5\_1 |
| e6 |  |  | 1.693 | .153 | 11.036 | \*\*\* | v6\_1 |
| e8 |  |  | 1.468 | .141 | 10.435 | \*\*\* | v7\_1 |
| e9 |  |  | 1.350 | .136 | 9.938 | \*\*\* | v8\_1 |
| e10 |  |  | 1.642 | .149 | 11.043 | \*\*\* | v9\_1 |
| e11 |  |  | 1.926 | .155 | 12.425 | \*\*\* | v10\_1 |
| e12 |  |  | 1.700 | .146 | 11.638 | \*\*\* | v11\_1 |
| e13 |  |  | 1.666 | .141 | 11.842 | \*\*\* | v12\_1 |
| e14 |  |  | 1.177 | .119 | 9.910 | \*\*\* | v13\_1 |
| e15 |  |  | 1.181 | .115 | 10.275 | \*\*\* | v14\_1 |
| e16 |  |  | 1.410 | .132 | 10.656 | \*\*\* | v15\_1 |
| e17 |  |  | 1.523 | .138 | 11.052 | \*\*\* | v16\_1 |
| e18 |  |  | 1.146 | .122 | 9.360 | \*\*\* | v17\_1 |
| e20 |  |  | 1.585 | .141 | 11.227 | \*\*\* | v18\_1 |

##### Squared Multiple Correlations: (other - Measurement residuals)

|  |  |  | Estimate |
| --- | --- | --- | --- |
| AI自我效能 |  |  | .234 |
| 防御性动机 |  |  | .239 |
| 使用行为 |  |  | .380 |
| AB5 |  |  | .526 |
| AB3 |  |  | .653 |
| AB2 |  |  | .542 |
| AB1 |  |  | .573 |
| SE5 |  |  | .625 |
| SE4 |  |  | .648 |
| SE3 |  |  | .488 |
| SE2 |  |  | .511 |
| SE1 |  |  | .406 |
| DM5 |  |  | .498 |
| DM3 |  |  | .579 |
| DM2 |  |  | .546 |
| DM1 |  |  | .499 |
| AA5 |  |  | .612 |
| AA4 |  |  | .565 |
| AA3 |  |  | .560 |
| AA2 |  |  | .611 |
| AA1 |  |  | .669 |

##### User-defined estimands: (other - Measurement residuals)

|  |  |  |  |
| --- | --- | --- | --- |
| diff1 |  |  | .000 |
| diff2 |  |  | .024 |
| diff3 |  |  | .087 |
| diff4 |  |  | .030 |
| diff5 |  |  | -.302 |

##### Modification Indices (other - Measurement residuals)

##### Covariances: (other - Measurement residuals)

|  |  |  | M.I. | Par Change |
| --- | --- | --- | --- | --- |
| e20 | <--> | e23 | 5.847 | -.284 |
| e13 | <--> | AI威胁感知 | 5.656 | -.339 |
| e9 | <--> | e18 | 4.264 | .226 |
| e8 | <--> | e22 | 6.677 | .292 |
| e6 | <--> | e11 | 5.418 | -.319 |
| e5 | <--> | e14 | 13.600 | -.370 |
| e2 | <--> | e14 | 6.645 | .261 |
| e2 | <--> | e13 | 4.302 | -.233 |
| e1 | <--> | e4 | 5.285 | -.225 |
| e1 | <--> | e3 | 5.782 | .237 |

##### Variances: (other - Measurement residuals)

|  |  |  | M.I. | Par Change |
| --- | --- | --- | --- | --- |

##### Regression Weights: (other - Measurement residuals)

|  |  |  | M.I. | Par Change |
| --- | --- | --- | --- | --- |
| AB5 | <--- | DM5 | 4.618 | -.108 |
| SE4 | <--- | AA2 | 4.352 | .095 |
| SE3 | <--- | AI威胁感知 | 5.656 | -.155 |
| SE3 | <--- | AA3 | 5.358 | -.116 |
| SE3 | <--- | AA2 | 8.605 | -.149 |
| SE1 | <--- | DM1 | 7.161 | -.140 |
| DM2 | <--- | AB2 | 4.395 | .103 |
| DM2 | <--- | AB1 | 4.722 | .107 |
| DM1 | <--- | SE1 | 5.274 | -.120 |
| AA5 | <--- | SE4 | 6.780 | -.115 |
| AA2 | <--- | SE4 | 4.104 | .090 |

##### Bootstrap (other - Measurement residuals)

##### Bootstrap standard errors (other - Measurement residuals)

##### Scalar Estimates (other - Measurement residuals)

##### Regression Weights: (other - Measurement residuals)

| Parameter | | | SE | SE-SE | Mean | Bias | SE-Bias |
| --- | --- | --- | --- | --- | --- | --- | --- |
| AI自我效能 | <--- | AI威胁感知 | .047 | .001 | .379 | .003 | .002 |
| 防御性动机 | <--- | AI威胁感知 | .063 | .001 | .401 | .002 | .002 |
| 防御性动机 | <--- | AI自我效能 | .077 | .002 | .076 | .002 | .002 |
| 使用行为 | <--- | 防御性动机 | .061 | .001 | .409 | .003 | .002 |
| 使用行为 | <--- | AI自我效能 | .073 | .002 | .461 | -.002 | .002 |
| AA1 | <--- | AI威胁感知 | .000 | .000 | 1.000 | .000 | .000 |
| AA2 | <--- | AI威胁感知 | .048 | .001 | .951 | -.001 | .002 |
| AA3 | <--- | AI威胁感知 | .046 | .001 | .921 | .002 | .001 |
| AA4 | <--- | AI威胁感知 | .052 | .001 | .920 | .000 | .002 |
| AA5 | <--- | AI威胁感知 | .052 | .001 | .945 | .001 | .002 |
| DM1 | <--- | 防御性动机 | .000 | .000 | 1.000 | .000 | .000 |
| DM2 | <--- | 防御性动机 | .073 | .002 | 1.028 | .006 | .002 |
| DM3 | <--- | 防御性动机 | .075 | .002 | 1.056 | .007 | .002 |
| DM5 | <--- | 防御性动机 | .078 | .002 | .988 | .005 | .002 |
| SE1 | <--- | AI自我效能 | .000 | .000 | 1.000 | .000 | .000 |
| SE2 | <--- | AI自我效能 | .092 | .002 | 1.157 | -.005 | .003 |
| SE3 | <--- | AI自我效能 | .088 | .002 | 1.100 | .002 | .003 |
| SE4 | <--- | AI自我效能 | .096 | .002 | 1.279 | -.002 | .003 |
| SE5 | <--- | AI自我效能 | .093 | .002 | 1.223 | .000 | .003 |
| AB1 | <--- | 使用行为 | .000 | .000 | 1.000 | .000 | .000 |
| AB2 | <--- | 使用行为 | .060 | .001 | .977 | .002 | .002 |
| AB3 | <--- | 使用行为 | .063 | .001 | 1.071 | .003 | .002 |
| AB5 | <--- | 使用行为 | .066 | .001 | .966 | .002 | .002 |

##### Standardized Regression Weights: (other - Measurement residuals)

| Parameter | | | SE | SE-SE | Mean | Bias | SE-Bias |
| --- | --- | --- | --- | --- | --- | --- | --- |
| AI自我效能 | <--- | AI威胁感知 | .045 | .001 | .484 | .001 | .001 |
| 防御性动机 | <--- | AI威胁感知 | .063 | .001 | .457 | .003 | .002 |
| 防御性动机 | <--- | AI自我效能 | .068 | .002 | .067 | .002 | .002 |
| 使用行为 | <--- | 防御性动机 | .053 | .001 | .384 | .001 | .002 |
| 使用行为 | <--- | AI自我效能 | .052 | .001 | .384 | -.002 | .002 |
| AA1 | <--- | AI威胁感知 | .023 | .001 | .817 | .000 | .001 |
| AA2 | <--- | AI威胁感知 | .023 | .001 | .780 | -.001 | .001 |
| AA3 | <--- | AI威胁感知 | .025 | .001 | .749 | .001 | .001 |
| AA4 | <--- | AI威胁感知 | .027 | .001 | .751 | -.001 | .001 |
| AA5 | <--- | AI威胁感知 | .026 | .001 | .783 | .001 | .001 |
| DM1 | <--- | 防御性动机 | .033 | .001 | .704 | -.002 | .001 |
| DM2 | <--- | 防御性动机 | .031 | .001 | .739 | .000 | .001 |
| DM3 | <--- | 防御性动机 | .029 | .001 | .762 | .001 | .001 |
| DM5 | <--- | 防御性动机 | .033 | .001 | .706 | .000 | .001 |
| SE1 | <--- | AI自我效能 | .035 | .001 | .639 | .002 | .001 |
| SE2 | <--- | AI自我效能 | .033 | .001 | .714 | -.002 | .001 |
| SE3 | <--- | AI自我效能 | .031 | .001 | .701 | .002 | .001 |
| SE4 | <--- | AI自我效能 | .024 | .001 | .804 | -.001 | .001 |
| SE5 | <--- | AI自我效能 | .025 | .001 | .792 | .001 | .001 |
| AB1 | <--- | 使用行为 | .028 | .001 | .758 | .001 | .001 |
| AB2 | <--- | 使用行为 | .030 | .001 | .737 | .001 | .001 |
| AB3 | <--- | 使用行为 | .024 | .001 | .809 | .001 | .001 |
| AB5 | <--- | 使用行为 | .030 | .001 | .726 | .000 | .001 |

##### Variances: (other - Measurement residuals)

| Parameter | | | SE | SE-SE | Mean | Bias | SE-Bias |
| --- | --- | --- | --- | --- | --- | --- | --- |
| AI威胁感知 |  |  | .189 | .004 | 2.179 | -.003 | .006 |
| e21 |  |  | .144 | .003 | 1.015 | .005 | .005 |
| e23 |  |  | .165 | .004 | 1.258 | -.025 | .005 |
| e22 |  |  | .139 | .003 | 1.165 | -.009 | .004 |
| e1 |  |  | .108 | .002 | 1.076 | -.004 | .003 |
| e2 |  |  | .108 | .002 | 1.255 | -.002 | .003 |
| e3 |  |  | .113 | .003 | 1.433 | -.015 | .004 |
| e4 |  |  | .123 | .003 | 1.414 | -.009 | .004 |
| e5 |  |  | .123 | .003 | 1.221 | -.014 | .004 |
| e6 |  |  | .151 | .003 | 1.689 | -.005 | .005 |
| e8 |  |  | .142 | .003 | 1.455 | -.013 | .004 |
| e9 |  |  | .125 | .003 | 1.333 | -.017 | .004 |
| e10 |  |  | .138 | .003 | 1.628 | -.014 | .004 |
| e11 |  |  | .134 | .003 | 1.907 | -.019 | .004 |
| e12 |  |  | .160 | .004 | 1.690 | -.010 | .005 |
| e13 |  |  | .134 | .003 | 1.644 | -.021 | .004 |
| e14 |  |  | .116 | .003 | 1.170 | -.007 | .004 |
| e15 |  |  | .120 | .003 | 1.165 | -.016 | .004 |
| e16 |  |  | .124 | .003 | 1.392 | -.018 | .004 |
| e17 |  |  | .143 | .003 | 1.509 | -.014 | .005 |
| e18 |  |  | .124 | .003 | 1.134 | -.012 | .004 |
| e20 |  |  | .137 | .003 | 1.571 | -.014 | .004 |

##### Squared Multiple Correlations: (other - Measurement residuals)

| Parameter | | | SE | SE-SE | Mean | Bias | SE-Bias |
| --- | --- | --- | --- | --- | --- | --- | --- |
| AI自我效能 |  |  | .043 | .001 | .236 | .003 | .001 |
| 防御性动机 |  |  | .048 | .001 | .249 | .010 | .002 |
| 使用行为 |  |  | .050 | .001 | .384 | .004 | .002 |
| AB5 |  |  | .043 | .001 | .528 | .001 | .001 |
| AB3 |  |  | .039 | .001 | .655 | .002 | .001 |
| AB2 |  |  | .044 | .001 | .544 | .002 | .001 |
| AB1 |  |  | .042 | .001 | .575 | .002 | .001 |
| SE5 |  |  | .040 | .001 | .628 | .002 | .001 |
| SE4 |  |  | .038 | .001 | .647 | .000 | .001 |
| SE3 |  |  | .043 | .001 | .492 | .004 | .001 |
| SE2 |  |  | .047 | .001 | .510 | -.001 | .001 |
| SE1 |  |  | .045 | .001 | .410 | .004 | .001 |
| DM5 |  |  | .047 | .001 | .499 | .001 | .001 |
| DM3 |  |  | .043 | .001 | .581 | .003 | .001 |
| DM2 |  |  | .045 | .001 | .547 | .001 | .001 |
| DM1 |  |  | .046 | .001 | .497 | -.002 | .001 |
| AA5 |  |  | .041 | .001 | .613 | .002 | .001 |
| AA4 |  |  | .040 | .001 | .565 | .000 | .001 |
| AA3 |  |  | .037 | .001 | .562 | .002 | .001 |
| AA2 |  |  | .036 | .001 | .610 | -.001 | .001 |
| AA1 |  |  | .037 | .001 | .669 | .000 | .001 |

##### User-defined estimands: (other - Measurement residuals)

| Parameter | | | SE | SE-SE | Mean | Bias | SE-Bias |
| --- | --- | --- | --- | --- | --- | --- | --- |
| diff1 |  |  | .000 | .000 | .000 | .000 | .000 |
| diff2 |  |  | .076 | .002 | .022 | -.001 | .002 |
| diff3 |  |  | .095 | .002 | .082 | -.005 | .003 |
| diff4 |  |  | .077 | .002 | .030 | .000 | .002 |
| diff5 |  |  | .092 | .002 | -.303 | -.002 | .003 |

##### Bootstrap Confidence (other - Measurement residuals)

##### Percentile method (other - Measurement residuals)

##### 95% confidence intervals (percentile method)

##### Scalar Estimates (other - Measurement residuals)

##### Regression Weights: (other - Measurement residuals)

| Parameter | | | Estimate | Lower | Upper | P |
| --- | --- | --- | --- | --- | --- | --- |
| AI自我效能 | <--- | AI威胁感知 | .376 | .288 | .473 | .002 |
| 防御性动机 | <--- | AI威胁感知 | .399 | .282 | .530 | .002 |
| 防御性动机 | <--- | AI自我效能 | .074 | -.077 | .222 | .343 |
| 使用行为 | <--- | 防御性动机 | .406 | .293 | .529 | .002 |
| 使用行为 | <--- | AI自我效能 | .463 | .326 | .607 | .002 |
| AA1 | <--- | AI威胁感知 | 1.000 | 1.000 | 1.000 | ... |
| AA2 | <--- | AI威胁感知 | .951 | .863 | 1.047 | .002 |
| AA3 | <--- | AI威胁感知 | .919 | .830 | 1.016 | .002 |
| AA4 | <--- | AI威胁感知 | .920 | .828 | 1.030 | .002 |
| AA5 | <--- | AI威胁感知 | .944 | .847 | 1.059 | .002 |
| DM1 | <--- | 防御性动机 | 1.000 | 1.000 | 1.000 | ... |
| DM2 | <--- | 防御性动机 | 1.022 | .887 | 1.172 | .002 |
| DM3 | <--- | 防御性动机 | 1.048 | .911 | 1.207 | .002 |
| DM5 | <--- | 防御性动机 | .983 | .846 | 1.142 | .002 |
| SE1 | <--- | AI自我效能 | 1.000 | 1.000 | 1.000 | ... |
| SE2 | <--- | AI自我效能 | 1.162 | .979 | 1.348 | .002 |
| SE3 | <--- | AI自我效能 | 1.098 | .946 | 1.282 | .002 |
| SE4 | <--- | AI自我效能 | 1.282 | 1.112 | 1.481 | .002 |
| SE5 | <--- | AI自我效能 | 1.223 | 1.058 | 1.446 | .002 |
| AB1 | <--- | 使用行为 | 1.000 | 1.000 | 1.000 | ... |
| AB2 | <--- | 使用行为 | .975 | .864 | 1.108 | .002 |
| AB3 | <--- | 使用行为 | 1.067 | .955 | 1.209 | .002 |
| AB5 | <--- | 使用行为 | .965 | .844 | 1.113 | .002 |

##### Standardized Regression Weights: (other - Measurement residuals)

| Parameter | | | Estimate | Lower | Upper | P |
| --- | --- | --- | --- | --- | --- | --- |
| AI自我效能 | <--- | AI威胁感知 | .483 | .392 | .571 | .002 |
| 防御性动机 | <--- | AI威胁感知 | .454 | .336 | .577 | .002 |
| 防御性动机 | <--- | AI自我效能 | .065 | -.067 | .197 | .343 |
| 使用行为 | <--- | 防御性动机 | .383 | .274 | .482 | .002 |
| 使用行为 | <--- | AI自我效能 | .386 | .277 | .482 | .002 |
| AA1 | <--- | AI威胁感知 | .818 | .772 | .859 | .002 |
| AA2 | <--- | AI威胁感知 | .782 | .732 | .821 | .002 |
| AA3 | <--- | AI威胁感知 | .748 | .697 | .795 | .002 |
| AA4 | <--- | AI威胁感知 | .752 | .695 | .804 | .002 |
| AA5 | <--- | AI威胁感知 | .782 | .730 | .828 | .002 |
| DM1 | <--- | 防御性动机 | .706 | .638 | .764 | .002 |
| DM2 | <--- | 防御性动机 | .739 | .677 | .799 | .002 |
| DM3 | <--- | 防御性动机 | .761 | .703 | .817 | .002 |
| DM5 | <--- | 防御性动机 | .706 | .638 | .766 | .002 |
| SE1 | <--- | AI自我效能 | .637 | .567 | .706 | .002 |
| SE2 | <--- | AI自我效能 | .715 | .646 | .776 | .002 |
| SE3 | <--- | AI自我效能 | .699 | .637 | .761 | .002 |
| SE4 | <--- | AI自我效能 | .805 | .752 | .850 | .002 |
| SE5 | <--- | AI自我效能 | .791 | .739 | .838 | .002 |
| AB1 | <--- | 使用行为 | .757 | .699 | .812 | .002 |
| AB2 | <--- | 使用行为 | .736 | .674 | .793 | .002 |
| AB3 | <--- | 使用行为 | .808 | .759 | .856 | .002 |
| AB5 | <--- | 使用行为 | .726 | .664 | .784 | .002 |

##### Variances: (other - Measurement residuals)

| Parameter | | | Estimate | Lower | Upper | P |
| --- | --- | --- | --- | --- | --- | --- |
| AI威胁感知 |  |  | 2.183 | 1.786 | 2.547 | .002 |
| e21 |  |  | 1.010 | .756 | 1.311 | .002 |
| e23 |  |  | 1.283 | .953 | 1.597 | .002 |
| e22 |  |  | 1.173 | .889 | 1.434 | .002 |
| e1 |  |  | 1.080 | .862 | 1.272 | .002 |
| e2 |  |  | 1.258 | 1.053 | 1.468 | .002 |
| e3 |  |  | 1.448 | 1.224 | 1.679 | .002 |
| e4 |  |  | 1.423 | 1.172 | 1.661 | .002 |
| e5 |  |  | 1.235 | .998 | 1.478 | .002 |
| e6 |  |  | 1.693 | 1.388 | 1.997 | .002 |
| e8 |  |  | 1.468 | 1.168 | 1.741 | .002 |
| e9 |  |  | 1.350 | 1.070 | 1.599 | .002 |
| e10 |  |  | 1.642 | 1.365 | 1.901 | .002 |
| e11 |  |  | 1.926 | 1.647 | 2.169 | .002 |
| e12 |  |  | 1.700 | 1.365 | 1.996 | .002 |
| e13 |  |  | 1.666 | 1.391 | 1.911 | .002 |
| e14 |  |  | 1.177 | .948 | 1.387 | .002 |
| e15 |  |  | 1.181 | .927 | 1.406 | .002 |
| e16 |  |  | 1.410 | 1.153 | 1.646 | .002 |
| e17 |  |  | 1.523 | 1.236 | 1.814 | .002 |
| e18 |  |  | 1.146 | .900 | 1.382 | .002 |
| e20 |  |  | 1.585 | 1.310 | 1.836 | .002 |

##### Squared Multiple Correlations: (other - Measurement residuals)

| Parameter | | | Estimate | Lower | Upper | P |
| --- | --- | --- | --- | --- | --- | --- |
| AI自我效能 |  |  | .234 | .153 | .326 | .002 |
| 防御性动机 |  |  | .239 | .158 | .349 | .002 |
| 使用行为 |  |  | .380 | .291 | .483 | .002 |
| AB5 |  |  | .526 | .441 | .615 | .002 |
| AB3 |  |  | .653 | .576 | .733 | .002 |
| AB2 |  |  | .542 | .454 | .629 | .002 |
| AB1 |  |  | .573 | .488 | .659 | .002 |
| SE5 |  |  | .625 | .546 | .703 | .002 |
| SE4 |  |  | .648 | .565 | .722 | .002 |
| SE3 |  |  | .488 | .406 | .579 | .002 |
| SE2 |  |  | .511 | .418 | .603 | .002 |
| SE1 |  |  | .406 | .322 | .499 | .002 |
| DM5 |  |  | .498 | .407 | .587 | .002 |
| DM3 |  |  | .579 | .494 | .668 | .002 |
| DM2 |  |  | .546 | .459 | .638 | .002 |
| DM1 |  |  | .499 | .407 | .583 | .002 |
| AA5 |  |  | .612 | .533 | .686 | .002 |
| AA4 |  |  | .565 | .483 | .647 | .002 |
| AA3 |  |  | .560 | .486 | .632 | .002 |
| AA2 |  |  | .611 | .536 | .674 | .002 |
| AA1 |  |  | .669 | .596 | .738 | .002 |

##### User-defined estimands: (other - Measurement residuals)

| Parameter | | | Estimate | Lower | Upper | P |
| --- | --- | --- | --- | --- | --- | --- |
| diff1 |  |  | .000 | .000 | .000 | ... |
| diff2 |  |  | .024 | -.126 | .166 | .767 |
| diff3 |  |  | .087 | -.088 | .272 | .373 |
| diff4 |  |  | .030 | -.121 | .190 | .735 |
| diff5 |  |  | -.302 | -.481 | -.130 | .002 |

##### Bias-corrected percentile method (other - Measurement residuals)

##### 95% confidence intervals (bias-corrected percentile method)

##### Scalar Estimates (other - Measurement residuals)

##### Regression Weights: (other - Measurement residuals)

| Parameter | | | Estimate | Lower | Upper | P |
| --- | --- | --- | --- | --- | --- | --- |
| AI自我效能 | <--- | AI威胁感知 | .376 | .282 | .465 | .003 |
| 防御性动机 | <--- | AI威胁感知 | .399 | .279 | .528 | .002 |
| 防御性动机 | <--- | AI自我效能 | .074 | -.079 | .214 | .377 |
| 使用行为 | <--- | 防御性动机 | .406 | .287 | .527 | .003 |
| 使用行为 | <--- | AI自我效能 | .463 | .327 | .608 | .002 |
| AA1 | <--- | AI威胁感知 | 1.000 | 1.000 | 1.000 | ... |
| AA2 | <--- | AI威胁感知 | .951 | .865 | 1.052 | .002 |
| AA3 | <--- | AI威胁感知 | .919 | .830 | 1.016 | .002 |
| AA4 | <--- | AI威胁感知 | .920 | .830 | 1.034 | .002 |
| AA5 | <--- | AI威胁感知 | .944 | .851 | 1.069 | .002 |
| DM1 | <--- | 防御性动机 | 1.000 | 1.000 | 1.000 | ... |
| DM2 | <--- | 防御性动机 | 1.022 | .876 | 1.158 | .003 |
| DM3 | <--- | 防御性动机 | 1.048 | .894 | 1.198 | .003 |
| DM5 | <--- | 防御性动机 | .983 | .841 | 1.138 | .002 |
| SE1 | <--- | AI自我效能 | 1.000 | 1.000 | 1.000 | ... |
| SE2 | <--- | AI自我效能 | 1.162 | 1.009 | 1.372 | .001 |
| SE3 | <--- | AI自我效能 | 1.098 | .948 | 1.287 | .002 |
| SE4 | <--- | AI自我效能 | 1.282 | 1.124 | 1.497 | .001 |
| SE5 | <--- | AI自我效能 | 1.223 | 1.064 | 1.454 | .001 |
| AB1 | <--- | 使用行为 | 1.000 | 1.000 | 1.000 | ... |
| AB2 | <--- | 使用行为 | .975 | .867 | 1.114 | .002 |
| AB3 | <--- | 使用行为 | 1.067 | .955 | 1.210 | .002 |
| AB5 | <--- | 使用行为 | .965 | .853 | 1.125 | .001 |

##### Standardized Regression Weights: (other - Measurement residuals)

| Parameter | | | Estimate | Lower | Upper | P |
| --- | --- | --- | --- | --- | --- | --- |
| AI自我效能 | <--- | AI威胁感知 | .483 | .388 | .566 | .003 |
| 防御性动机 | <--- | AI威胁感知 | .454 | .323 | .571 | .003 |
| 防御性动机 | <--- | AI自我效能 | .065 | -.070 | .195 | .366 |
| 使用行为 | <--- | 防御性动机 | .383 | .274 | .481 | .002 |
| 使用行为 | <--- | AI自我效能 | .386 | .277 | .482 | .002 |
| AA1 | <--- | AI威胁感知 | .818 | .772 | .858 | .002 |
| AA2 | <--- | AI威胁感知 | .782 | .731 | .820 | .003 |
| AA3 | <--- | AI威胁感知 | .748 | .694 | .794 | .003 |
| AA4 | <--- | AI威胁感知 | .752 | .694 | .804 | .002 |
| AA5 | <--- | AI威胁感知 | .782 | .722 | .825 | .004 |
| DM1 | <--- | 防御性动机 | .706 | .646 | .770 | .001 |
| DM2 | <--- | 防御性动机 | .739 | .673 | .795 | .003 |
| DM3 | <--- | 防御性动机 | .761 | .696 | .810 | .003 |
| DM5 | <--- | 防御性动机 | .706 | .636 | .765 | .002 |
| SE1 | <--- | AI自我效能 | .637 | .560 | .702 | .004 |
| SE2 | <--- | AI自我效能 | .715 | .647 | .778 | .002 |
| SE3 | <--- | AI自我效能 | .699 | .627 | .752 | .004 |
| SE4 | <--- | AI自我效能 | .805 | .752 | .850 | .002 |
| SE5 | <--- | AI自我效能 | .791 | .736 | .834 | .004 |
| AB1 | <--- | 使用行为 | .757 | .692 | .807 | .004 |
| AB2 | <--- | 使用行为 | .736 | .671 | .789 | .003 |
| AB3 | <--- | 使用行为 | .808 | .756 | .853 | .003 |
| AB5 | <--- | 使用行为 | .726 | .663 | .782 | .003 |

##### Variances: (other - Measurement residuals)

| Parameter | | | Estimate | Lower | Upper | P |
| --- | --- | --- | --- | --- | --- | --- |
| AI威胁感知 |  |  | 2.183 | 1.784 | 2.544 | .002 |
| e21 |  |  | 1.010 | .757 | 1.311 | .002 |
| e23 |  |  | 1.283 | 1.001 | 1.665 | .000 |
| e22 |  |  | 1.173 | .913 | 1.467 | .001 |
| e1 |  |  | 1.080 | .866 | 1.273 | .002 |
| e2 |  |  | 1.258 | 1.057 | 1.469 | .002 |
| e3 |  |  | 1.448 | 1.260 | 1.723 | .001 |
| e4 |  |  | 1.423 | 1.196 | 1.701 | .001 |
| e5 |  |  | 1.235 | 1.024 | 1.508 | .001 |
| e6 |  |  | 1.693 | 1.406 | 2.013 | .001 |
| e8 |  |  | 1.468 | 1.222 | 1.783 | .001 |
| e9 |  |  | 1.350 | 1.113 | 1.636 | .001 |
| e10 |  |  | 1.642 | 1.386 | 1.915 | .001 |
| e11 |  |  | 1.926 | 1.683 | 2.233 | .001 |
| e12 |  |  | 1.700 | 1.392 | 2.010 | .001 |
| e13 |  |  | 1.666 | 1.424 | 1.948 | .001 |
| e14 |  |  | 1.177 | .961 | 1.392 | .001 |
| e15 |  |  | 1.181 | .975 | 1.427 | .001 |
| e16 |  |  | 1.410 | 1.184 | 1.707 | .001 |
| e17 |  |  | 1.523 | 1.263 | 1.841 | .001 |
| e18 |  |  | 1.146 | .929 | 1.414 | .001 |
| e20 |  |  | 1.585 | 1.340 | 1.868 | .001 |

##### Squared Multiple Correlations: (other - Measurement residuals)

| Parameter | | | Estimate | Lower | Upper | P |
| --- | --- | --- | --- | --- | --- | --- |
| AI自我效能 |  |  | .234 | .151 | .320 | .003 |
| 防御性动机 |  |  | .239 | .146 | .332 | .005 |
| 使用行为 |  |  | .380 | .286 | .478 | .003 |
| AB5 |  |  | .526 | .439 | .611 | .003 |
| AB3 |  |  | .653 | .572 | .728 | .003 |
| AB2 |  |  | .542 | .451 | .622 | .003 |
| AB1 |  |  | .573 | .479 | .651 | .004 |
| SE5 |  |  | .625 | .541 | .696 | .004 |
| SE4 |  |  | .648 | .565 | .722 | .002 |
| SE3 |  |  | .488 | .393 | .566 | .004 |
| SE2 |  |  | .511 | .419 | .605 | .002 |
| SE1 |  |  | .406 | .313 | .493 | .004 |
| DM5 |  |  | .498 | .404 | .586 | .002 |
| DM3 |  |  | .579 | .485 | .657 | .003 |
| DM2 |  |  | .546 | .453 | .632 | .003 |
| DM1 |  |  | .499 | .417 | .593 | .001 |
| AA5 |  |  | .612 | .521 | .680 | .004 |
| AA4 |  |  | .565 | .482 | .646 | .002 |
| AA3 |  |  | .560 | .482 | .630 | .003 |
| AA2 |  |  | .611 | .535 | .673 | .003 |
| AA1 |  |  | .669 | .595 | .736 | .002 |

##### User-defined estimands: (other - Measurement residuals)

| Parameter | | | Estimate | Lower | Upper | P |
| --- | --- | --- | --- | --- | --- | --- |
| diff1 |  |  | .000 | .000 | .000 | ... |
| diff2 |  |  | .024 | -.129 | .164 | .797 |
| diff3 |  |  | .087 | -.082 | .285 | .321 |
| diff4 |  |  | .030 | -.122 | .187 | .754 |
| diff5 |  |  | -.302 | -.484 | -.132 | .002 |

##### Minimization History (Measurement residuals)

| Iteration |  | Negative eigenvalues | Condition # | Smallest eigenvalue | Diameter | F | NTries | Ratio |
| --- | --- | --- | --- | --- | --- | --- | --- | --- |
| 0 | e | 8 |  | -.638 | 9999.000 | 3333.496 | 0 | 9999.000 |
| 1 | e\* | 3 |  | -.086 | 3.582 | 1257.627 | 20 | .338 |
| 2 | e | 1 |  | -.025 | 1.423 | 534.037 | 4 | .770 |
| 3 | e | 0 | 17.195 |  | .994 | 340.149 | 5 | .781 |
| 4 | e | 0 | 21.884 |  | .456 | 294.743 | 1 | 1.119 |
| 5 | e | 0 | 37.401 |  | .211 | 290.789 | 1 | 1.106 |
| 6 | e | 0 | 44.726 |  | .059 | 290.628 | 1 | 1.046 |
| 7 | e | 0 | 45.100 |  | .005 | 290.627 | 1 | 1.005 |
| 8 | e | 0 | 45.102 |  | .000 | 290.627 | 1 | 1.000 |

##### Bootstrap (Measurement residuals)

##### Summary of Bootstrap Iterations (Measurement residuals)

##### (Measurement residuals)

| Iterations | Method 0 | Method 1 | Method 2 |
| --- | --- | --- | --- |
| 1 | 0 | 0 | 0 |
| 2 | 0 | 0 | 0 |
| 3 | 0 | 0 | 0 |
| 4 | 0 | 1 | 0 |
| 5 | 0 | 148 | 0 |
| 6 | 0 | 402 | 0 |
| 7 | 0 | 296 | 0 |
| 8 | 0 | 117 | 0 |
| 9 | 0 | 27 | 0 |
| 10 | 0 | 6 | 0 |
| 11 | 0 | 3 | 0 |
| 12 | 0 | 0 | 0 |
| 13 | 0 | 0 | 0 |
| 14 | 0 | 0 | 0 |
| 15 | 0 | 0 | 0 |
| 16 | 0 | 0 | 0 |
| 17 | 0 | 0 | 0 |
| 18 | 0 | 0 | 0 |
| 19 | 0 | 0 | 0 |
| Total | 0 | 1000 | 0 |

0 bootstrap samples were unused because of a singular covariance matrix.

0 bootstrap samples were unused because a solution was not found.

1000 usable bootstrap samples were obtained.

##### Bootstrap Distributions (Measurement residuals)

##### ML discrepancy (implied vs sample) (Measurement residuals)

|  |  |  |
| --- | --- | --- |
|  |  | \|-------------------- |
|  | 481.776 | \|\* |
|  | 502.535 | \|\*\* |
|  | 523.294 | \|\*\*\*\*\* |
|  | 544.053 | \|\*\*\*\*\*\*\*\* |
|  | 564.812 | \|\*\*\*\*\*\*\*\*\*\* |
|  | 585.570 | \|\*\*\*\*\*\*\*\*\*\*\*\*\*\*\*\* |
|  | 606.329 | \|\*\*\*\*\*\*\*\*\*\*\*\*\*\*\*\*\*\*\* |
| N = 1000 | 627.088 | \|\*\*\*\*\*\*\*\*\*\*\*\*\*\*\*\* |
| Mean = 608.278 | 647.847 | \|\*\*\*\*\*\*\*\*\*\*\*\* |
| S. e. = 1.564 | 668.606 | \|\*\*\*\*\*\*\* |
|  | 689.364 | \|\*\*\*\*\* |
|  | 710.123 | \|\*\* |
|  | 730.882 | \|\* |
|  | 751.641 | \|\* |
|  | 772.400 | \|\* |
|  |  | \|-------------------- |

##### ML discrepancy (implied vs pop) (Measurement residuals)

|  |  |  |
| --- | --- | --- |
|  |  | \|-------------------- |
|  | 326.107 | \|\* |
|  | 333.331 | \|\*\* |
|  | 340.555 | \|\*\*\*\* |
|  | 347.779 | \|\*\*\*\*\*\*\*\*\*\*\*\*\* |
|  | 355.003 | \|\*\*\*\*\*\*\*\*\*\*\*\*\*\*\*\*\*\*\* |
|  | 362.227 | \|\*\*\*\*\*\*\*\*\*\*\*\*\*\*\*\*\*\* |
|  | 369.451 | \|\*\*\*\*\*\*\*\*\*\*\*\*\*\* |
| N = 1000 | 376.675 | \|\*\*\*\*\*\*\*\*\*\* |
| Mean = 361.882 | 383.899 | \|\*\*\*\* |
| S. e. = .419 | 391.124 | \|\*\* |
|  | 398.348 | \|\* |
|  | 405.572 | \|\* |
|  | 412.796 | \|\* |
|  | 420.020 | \|\* |
|  | 427.244 | \|\* |
|  |  | \|-------------------- |

##### K-L overoptimism (unstabilized) (Measurement residuals)

|  |  |  |
| --- | --- | --- |
|  |  | \|-------------------- |
|  | -188.489 | \|\* |
|  | -138.178 | \|\* |
|  | -87.868 | \|\*\*\* |
|  | -37.557 | \|\*\*\*\*\*\* |
|  | 12.754 | \|\*\*\*\*\*\*\*\*\*\* |
|  | 63.065 | \|\*\*\*\*\*\*\*\*\*\*\*\*\*\*\* |
|  | 113.375 | \|\*\*\*\*\*\*\*\*\*\*\*\*\*\*\*\*\*\*\* |
| N = 1000 | 163.686 | \|\*\*\*\*\*\*\*\*\*\*\*\*\*\*\*\*\*\*\*\* |
| Mean = 146.177 | 213.997 | \|\*\*\*\*\*\*\*\*\*\*\*\*\*\*\*\* |
| S. e. = 3.591 | 264.308 | \|\*\*\*\*\*\*\*\*\*\*\*\* |
|  | 314.618 | \|\*\*\*\*\*\*\*\* |
|  | 364.929 | \|\*\*\*\* |
|  | 415.240 | \|\* |
|  | 465.550 | \|\* |
|  | 515.861 | \|\* |
|  |  | \|-------------------- |

##### K-L overoptimism (stabilized) (Measurement residuals)

|  |  |  |
| --- | --- | --- |
|  |  | \|-------------------- |
|  | 26.103 | \|\* |
|  | 45.726 | \|\* |
|  | 65.350 | \|\*\*\* |
|  | 84.973 | \|\*\*\*\*\*\*\*\* |
|  | 104.596 | \|\*\*\*\*\*\*\*\*\*\*\*\*\*\* |
|  | 124.219 | \|\*\*\*\*\*\*\*\*\*\*\*\*\*\*\*\*\*\*\* |
|  | 143.842 | \|\*\*\*\*\*\*\*\*\*\*\*\*\*\*\*\*\*\*\*\* |
| N = 1000 | 163.465 | \|\*\*\*\*\*\*\*\*\*\*\*\*\*\*\*\*\*\*\*\* |
| Mean = 140.886 | 183.089 | \|\*\*\*\*\*\*\*\*\*\* |
| S. e. = 1.191 | 202.712 | \|\*\*\*\*\*\* |
|  | 222.335 | \|\*\* |
|  | 241.958 | \|\* |
|  | 261.581 | \|\* |
|  | 281.204 | \|\* |
|  | 300.828 | \|\* |
|  |  | \|-------------------- |

##### Model Fit Summary

##### CMIN

| Model | NPAR | CMIN | DF | P | CMIN/DF |
| --- | --- | --- | --- | --- | --- |
| Unconstrained | 82 | 257.968 | 260 | .524 | .992 |
| Measurement weights | 68 | 264.985 | 274 | .641 | .967 |
| Structural weights | 63 | 276.671 | 279 | .528 | .992 |
| Structural covariances | 62 | 276.710 | 280 | .544 | .988 |
| Structural residuals | 59 | 277.072 | 283 | .588 | .979 |
| Measurement residuals | 41 | 290.627 | 301 | .656 | .966 |
| Saturated model | 342 | .000 | 0 |  |  |
| Independence model | 36 | 3378.195 | 306 | .000 | 11.040 |

##### RMR, GFI

| Model | RMR | GFI | AGFI | PGFI |
| --- | --- | --- | --- | --- |
| Unconstrained | .142 | .931 | .909 | .708 |
| Measurement weights | .148 | .930 | .912 | .745 |
| Structural weights | .245 | .927 | .910 | .756 |
| Structural covariances | .239 | .927 | .910 | .759 |
| Structural residuals | .243 | .927 | .911 | .767 |
| Measurement residuals | .244 | .924 | .914 | .813 |
| Saturated model | .000 | 1.000 |  |  |
| Independence model | 1.097 | .331 | .252 | .296 |

##### Baseline Comparisons

| Model | NFI Delta1 | RFI rho1 | IFI Delta2 | TLI rho2 | CFI |
| --- | --- | --- | --- | --- | --- |
| Unconstrained | .924 | .910 | 1.001 | 1.001 | 1.000 |
| Measurement weights | .922 | .912 | 1.003 | 1.003 | 1.000 |
| Structural weights | .918 | .910 | 1.001 | 1.001 | 1.000 |
| Structural covariances | .918 | .910 | 1.001 | 1.001 | 1.000 |
| Structural residuals | .918 | .911 | 1.002 | 1.002 | 1.000 |
| Measurement residuals | .914 | .913 | 1.003 | 1.003 | 1.000 |
| Saturated model | 1.000 |  | 1.000 |  | 1.000 |
| Independence model | .000 | .000 | .000 | .000 | .000 |

##### Parsimony-Adjusted Measures

| Model | PRATIO | PNFI | PCFI |
| --- | --- | --- | --- |
| Unconstrained | .850 | .785 | .850 |
| Measurement weights | .895 | .825 | .895 |
| Structural weights | .912 | .837 | .912 |
| Structural covariances | .915 | .840 | .915 |
| Structural residuals | .925 | .849 | .925 |
| Measurement residuals | .984 | .899 | .984 |
| Saturated model | .000 | .000 | .000 |
| Independence model | 1.000 | .000 | .000 |

##### NCP

| Model | NCP | LO 90 | HI 90 |
| --- | --- | --- | --- |
| Unconstrained | .000 | .000 | 39.489 |
| Measurement weights | .000 | .000 | 32.538 |
| Structural weights | .000 | .000 | 40.507 |
| Structural covariances | .000 | .000 | 39.485 |
| Structural residuals | .000 | .000 | 36.697 |
| Measurement residuals | .000 | .000 | 32.906 |
| Saturated model | .000 | .000 | .000 |
| Independence model | 3072.195 | 2888.975 | 3262.759 |

##### FMIN

| Model | FMIN | F0 | LO 90 | HI 90 |
| --- | --- | --- | --- | --- |
| Unconstrained | .670 | .000 | .000 | .103 |
| Measurement weights | .688 | .000 | .000 | .085 |
| Structural weights | .719 | .000 | .000 | .105 |
| Structural covariances | .719 | .000 | .000 | .103 |
| Structural residuals | .720 | .000 | .000 | .095 |
| Measurement residuals | .755 | .000 | .000 | .085 |
| Saturated model | .000 | .000 | .000 | .000 |
| Independence model | 8.775 | 7.980 | 7.504 | 8.475 |

##### RMSEA

| Model | RMSEA | LO 90 | HI 90 | PCLOSE |
| --- | --- | --- | --- | --- |
| Unconstrained | .000 | .000 | .020 | 1.000 |
| Measurement weights | .000 | .000 | .018 | 1.000 |
| Structural weights | .000 | .000 | .019 | 1.000 |
| Structural covariances | .000 | .000 | .019 | 1.000 |
| Structural residuals | .000 | .000 | .018 | 1.000 |
| Measurement residuals | .000 | .000 | .017 | 1.000 |
| Independence model | .161 | .157 | .166 | .000 |

##### AIC

| Model | AIC | BCC | BIC | CAIC |
| --- | --- | --- | --- | --- |
| Unconstrained | 421.968 | 440.798 |  |  |
| Measurement weights | 400.985 | 416.601 |  |  |
| Structural weights | 402.671 | 417.138 |  |  |
| Structural covariances | 400.710 | 414.947 |  |  |
| Structural residuals | 395.072 | 408.621 |  |  |
| Measurement residuals | 372.627 | 382.042 |  |  |
| Saturated model | 684.000 | 762.537 |  |  |
| Independence model | 3450.195 | 3458.462 |  |  |

##### ECVI

| Model | ECVI | LO 90 | HI 90 | MECVI |
| --- | --- | --- | --- | --- |
| Unconstrained | 1.096 | 1.101 | 1.204 | 1.145 |
| Measurement weights | 1.042 | 1.065 | 1.149 | 1.082 |
| Structural weights | 1.046 | 1.052 | 1.157 | 1.083 |
| Structural covariances | 1.041 | 1.049 | 1.152 | 1.078 |
| Structural residuals | 1.026 | 1.042 | 1.137 | 1.061 |
| Measurement residuals | .968 | .995 | 1.080 | .992 |
| Saturated model | 1.777 | 1.777 | 1.777 | 1.981 |
| Independence model | 8.962 | 8.486 | 9.457 | 8.983 |

##### HOELTER

| Model | HOELTER .05 | HOELTER .01 |
| --- | --- | --- |
| Unconstrained | 447 | 473 |
| Measurement weights | 457 | 483 |
| Structural weights | 445 | 470 |
| Structural covariances | 447 | 472 |
| Structural residuals | 451 | 476 |
| Measurement residuals | 455 | 480 |
| Independence model | 41 | 43 |

##### Nested Model Comparisons

##### Assuming model Unconstrained to be correct:

|  |  |  |  |  |  |  |  |
| --- | --- | --- | --- | --- | --- | --- | --- |
| Model | DF | CMIN | P | NFI Delta-1 | IFI Delta-2 | RFI rho-1 | TLI rho2 |
| Measurement weights | 14 | 7.018 | .934 | .002 | .002 | -.002 | -.002 |
| Structural weights | 19 | 18.703 | .476 | .006 | .006 | .000 | .000 |
| Structural covariances | 20 | 18.742 | .539 | .006 | .006 | .000 | .000 |
| Structural residuals | 23 | 19.104 | .695 | .006 | .006 | -.001 | -.001 |
| Measurement residuals | 41 | 32.659 | .820 | .010 | .010 | -.002 | -.003 |

##### Assuming model Measurement weights to be correct:

|  |  |  |  |  |  |  |  |
| --- | --- | --- | --- | --- | --- | --- | --- |
| Model | DF | CMIN | P | NFI Delta-1 | IFI Delta-2 | RFI rho-1 | TLI rho2 |
| Structural weights | 5 | 11.685 | .039 | .003 | .004 | .002 | .002 |
| Structural covariances | 6 | 11.724 | .068 | .003 | .004 | .002 | .002 |
| Structural residuals | 9 | 12.087 | .208 | .004 | .004 | .001 | .001 |
| Measurement residuals | 27 | 25.642 | .539 | .008 | .008 | .000 | .000 |

##### Assuming model Structural weights to be correct:

|  |  |  |  |  |  |  |  |
| --- | --- | --- | --- | --- | --- | --- | --- |
| Model | DF | CMIN | P | NFI Delta-1 | IFI Delta-2 | RFI rho-1 | TLI rho2 |
| Structural covariances | 1 | .039 | .844 | .000 | .000 | .000 | .000 |
| Structural residuals | 4 | .401 | .982 | .000 | .000 | -.001 | -.001 |
| Measurement residuals | 22 | 13.956 | .903 | .004 | .005 | -.002 | -.003 |

##### Assuming model Structural covariances to be correct:

|  |  |  |  |  |  |  |  |
| --- | --- | --- | --- | --- | --- | --- | --- |
| Model | DF | CMIN | P | NFI Delta-1 | IFI Delta-2 | RFI rho-1 | TLI rho2 |
| Structural residuals | 3 | .363 | .948 | .000 | .000 | -.001 | -.001 |
| Measurement residuals | 21 | 13.917 | .873 | .004 | .004 | -.002 | -.002 |

##### Assuming model Structural residuals to be correct:

|  |  |  |  |  |  |  |  |
| --- | --- | --- | --- | --- | --- | --- | --- |
| Model | DF | CMIN | P | NFI Delta-1 | IFI Delta-2 | RFI rho-1 | TLI rho2 |
| Measurement residuals | 18 | 13.555 | .758 | .004 | .004 | -.001 | -.001 |

##### Execution time summary

|  |  |
| --- | --- |
| Minimization: | .016 |
| Miscellaneous: | 4.885 |
| Bootstrap: | .737 |
| Total: | 5.638 |
