# Supplementary material for: How AI threat perception relates to post-adoptive AI agent engagement: the dual paths of self-efficacy and defensive motivation
Source: Front Psychol. 2026 Oct 22;17:1887571. doi: 10.3389/fpsyg.2026.1887571 (PMC13735560; doi:10.3389/fpsyg.2026.1887571)
Supplement: Supplementary file 1 [file Data_Sheet_1.zip › log+Questionnaire/Manuscript with author detailsV9-Tracked.docx]

Type of the Paper (Article)

How AI Threat Perception Relates to Post-Adoptive AI Agent Engagement: The Dual Paths of Self-Efficacy and Defensive Motivation

Guilin Yang ^1^, Mingwei Feng ^1^ and Wanping Yang ^1,^*

^1^	School of Economics and Finance, Xi’an Jiaotong University, Xi’an, Shaanxi, China; yangguilin915@126.com

*****	Correspondence: yangwanping666@126.com

**Abstract**

As AI evolves into agent-based forms, AI threat perception has become a defining workplace concern. Informed by Cognitive Appraisal Theory and Protection Motivation Theory, this cross-sectional study investigates how AI threat perception relates to post-adoptive AI agent engagement via dual paths: AI self-efficacy and defensive motivation. Semi-structured interviews (N = 13) first grounded scale adaptation. Structural equation modeling of 387 knowledge workers demonstrates that AI threat perception is indirectly associated with post-adoptive engagement through both mediators, exhibiting an indirect-only pattern. Multi-group analysis further reveals demographic and contextual boundary conditions: higher education significantly amplifies defensive motivation (the link is stronger among postgraduates), while exploratory career-stage and regional comparisons indicate that the self-efficacy pathway is stronger among early-to-mid career professionals and workers in eastern regions. The study highlights the concurrent coexistence of capability beliefs and defensive intentions in post-adoptive use, while pointing to the need for longitudinal research to trace their temporal dynamics. These findings refine our understanding of technology threat perception and inform human-agent collaboration.

**Keywords:** AI threat perception; intelligent agents; self-efficacy; defensive motivation; post-adoptive AI agent engagement.

1. Introduction

In recent years, artificial intelligence has evolved from a mere auxiliary tool into "intelligent agents." These systems possess the ability to plan autonomously, invoke tools, and continuously optimize their performance. They can understand tasks, collaborate through reasoning, and refine their behaviors. Such systems show transformative potential across education, software development, management, and healthcare (Faisal, 2026; Yang et al., 2025). However, rapid technological penetration has not eliminated professional anxiety. Instead, it has triggered deep concerns in many knowledge-based roles. Workers fear job replacement, skill depreciation, and role marginalization (Hötte et al., 2023). Consequently, AI-induced threat perception has become a defining feature of workplace psychology in the digital age.

Traditional perspectives often view technology-related threat perception as a barrier to adoption. It is believed to weaken an individual's willingness to use new tools (Zhang & Tong, 2025). Yet, reality presents a more complex picture. Some individuals with high threat perception do not avoid AI. On the contrary, they actively learn, use it frequently, and even participate in customizing agent systems. This phenomenon challenges the linear assumption that threat perception inevitably inhibits behavior. According to Cognitive Appraisal Theory, evaluations of environmental stressors do not uniformly prompt withdrawal. Under specific conditions, stressors may evoke problem-focused coping or trigger compensatory competence development (Wang et al., 2022; Yang, 2026).

Although existing research has noted this paradox, the psychological mechanisms linking threat perceptions to sustained behavioral engagement remain under-explained. Specifically, there are gaps in the current literature. First, there is a lack of in-depth discussion on the mediating mechanisms of how "threat" transforms into proactive, task-directed motivation. Second, conventional technology adoption research predominantly focuses on initial acceptance or static usage intentions, offering limited insight into the post-adoptive phase where users must continuously adapt to evolving technologies. In the era of intelligent agents, this post-adoptive phase is characterized by sophisticated feature exploration, active workflow integration, and collaborative customization rather than routine, superficial querying (Oh & Lim, 2024).

Based on preliminary qualitative interviews, we identified two prominent psychological forces that accompany user engagement with AI agents. On the one hand, individuals may develop defensive motivation as a protective response to external workplace threats, such as the fear of falling behind or being replaced. On the other hand, users frequently rebuild confidence in their own capabilities through ongoing exploration, fostering AI self-efficacy. This leads to our core research questions: How is AI threat perception associated with Post-Adoptive AI Agent Engagement through the dual paths of self-efficacy and defensive motivation? What is the relationship between these capability-building and risk-avoidance mechanisms, and do individual characteristics, such as age and education, moderate these relationships?

To address this, the paper draws upon Cognitive Appraisal Theory and Protection Motivation Theory as overarching interpretive frameworks to construct a dual-path conceptual framework. Rather than directly capturing situational appraisal processes or formal protection-motivation components, our empirical framework focuses on downstream psychological orientations, wherein the self-efficacy path reflects a capability-building orientation, whereas the defensive motivation path captures a risk-mitigation orientation. Using structural equation modeling and multi-group analysis on a cross-sectional dataset of 387 knowledge workers, this study aims to examine the empirical associations between AI threat perception and Post-Adoptive AI Agent Engagement. The contributions are threefold. First, it empirically documents the simultaneous statistical mediation of self-efficacy and defensive motivation. Second, it identifies the boundary conditions of age, education, and region. Third, it extends the research context from basic AI tools to intelligent agents, providing nuanced empirical observations of post-adoptive engagement and sustained coproduction within ongoing human-agent interaction.

2. Literature Review and Research Framework

2.1. AI Threat Perception

The rapid development of artificial intelligence has changed work methods. It has also shaken basic expectations about job stability. Unlike traditional IT, AI systems can learn, reason, and make decisions. This significantly increases the likelihood of replacing human labor (Acemoglu & Restrepo, 2018). This technical feature has created a new type of AI threat perception. It refers to a persistent worry that AI might weaken professional value, reduce job necessity, or accelerate skill obsolescence (Li et al., 2025). This concept is not a fleeting discomfort with technology. Instead, it is a cognitive assessment that is long-term and structural.

Empirical studies show that the core source of AI threat perception lies in replaceability assessments. When individuals view their work as highly procedural, rule-based, or data-intensive, they fear replacement by AI (Vorobeva et al., 2022). Furthermore, organizational signals of AI deployment, such as automated processes or staff cuts, reinforce this perception (Kumar et al., 2023). AI threat perception also has significant sociocultural attributes. In collectivist cultures, employees may perceive AI threats more strongly. This is because technology is seen as a disruption to group harmony and stability (Mantello et al., 2021). This collective unease further amplifies individual psychological stress.

Early research often viewed technology-related threat perception as a negative outcome, leading to burnout or turnover intention. However, recent perspectives focus on its role as an antecedent. According to Cognitive Appraisal Theory, threat perception does not always lead to withdrawal. When individuals view a threat as a "challenge stressor," it can stimulate their coping potential (Nasaj et al., 2025). This parallels broader resilience perspectives indicating that when confronting external environmental volatility or technological disruptions, actors survive and build adaptive capacity primarily through proactive cognition and agile learning adaptation (Yang & Yang, 2026a). Therefore, AI threat perception may not lead to withdrawal but could serve as a starting point for adaptive behavior. The key question is which psychological mechanisms transform this threat into action. This leads to the discussion of two mediating paths below.

2.2. AI Self-Efficacy and Agent Adoption

When facing AI uncertainty, confidence in one's ability is a key resource for effective coping. In technology adoption, this is often called self-efficacy. It refers to an individual's judgment of their ability to master and use specific tools (Elavsky & McAuley, 2003). In the AI context, AI self-efficacy specifically refers to the confidence to learn, operate, or even customize AI agents.

Literature offers different views on how AI threat perception affects self-efficacy. Traditional views suggest that threats undermine confidence. However, the "challenge stressor" perspective argues that moderate threat perception can boost self-efficacy through a "coping assessment" mechanism. When individuals perceive an AI threat, they tend to assess their coping resources to eliminate discomfort. This assessment process can trigger a "fight" response. It prompts individuals to focus on improving their abilities, thereby cognitively reinforcing the belief that "I can do it" (Miraj et al., 2021).

Studies confirm that self-efficacy is a core predictor of deep technology use. In the diffusion of emerging technologies like cloud computing and big data, self-efficacy significantly and positively predicts usage intention and actual behavior (Yang & Lin, 2015). Its role is even more prominent in the AI field. AI tools often have "black box" characteristics, complexity, and rapid iteration. Users lacking basic confidence easily fall into a vicious cycle of avoidance and anxiety. Conversely, those who believe they can master AI are more willing to invest time in exploring advanced features. Examples include prompt engineering, workflow orchestration, or multi-agent collaboration (Han et al., 2025). This shift from passive user to active collaborator is a key marker of upgraded Post-Adoptive AI Agent Engagement.

Importantly, self-efficacy is not a static trait but can be dynamically improved through practice. Successful experiences from initial AI contact, such as completing a task, reinforce the belief that "I can do it." This promotes more complex application attempts (Bećirović et al., 2025). This dynamic improvement mechanism explains why some high-threat individuals show stronger AI integration behavior. Threat perception prompts them to take the first step, while initial success consolidates long-term use by boosting self-efficacy.

2.3. Defensive Motivation and Agent Adoption

Beyond capability beliefs, another important path stems from a rational response to external threats. Defensive motivation refers to the proactive intention to act to avoid potential losses, such as unemployment or marginalization. This concept is rooted in Protection Motivation Theory (Rogers, 1975). It emphasizes that when individuals perceive a threat as highly severe and susceptible, and believe countermeasures are effective, they initiate protective behavior. In the AI context, this motivation often appears as an urgency to learn. Conceptually, defensive motivation is distinct from both the upstream perception of environmental threat (which captures passive cognitive appraisal of external hazards) and generic technology usage intention (which lacks an explicit risk-mitigation purpose); instead, it operates as an active, problem-focused coping orientation aimed specifically at loss prevention.

Empirical studies find that job insecurity is a key driver of defensive technology learning. For instance, in knowledge-intensive industries like finance and law, practitioners worry about AI eroding professional barriers. Consequently, they significantly increase their learning investment in AI tools (Brynjolfsson, 2022). Although this behavior stems from external pressure, it may lead to substantial capability improvement. Research shows that highly educated individuals fear replacement more. This may be because they better understand the potential of technology and the risks of technological unemployment (Schwabe & Castellacci, 2020).

Furthermore, defensive motivation not only initiates behavior but also pushes usage toward higher levels. When individuals realize that basic operations are insufficient to resist replacement risks, they actively seek deep integration. This includes embedding AI into core workflows, participating in model fine-tuning, or developing specialized agents (Jöhnk et al., 2020; Golgeci et al., 2025). This leap from basic operation to active integration reflects the constructive potential of defensive motivation. It indicates that even if the initial motive is loss avoidance, the action process itself can generate new capabilities and value.

In summary, AI threat perception can influence post-adoptive AI agent engagement through two paths. One activates an internal sense of control by enhancing AI self-efficacy, enabling established users to tackle advanced features and complex prompt orchestration. The other triggers external adaptation strategies by stimulating defensive motivation, compelling experienced workers to embed agents deeply into core workflows to safeguard their professional relevance. Rather than reflecting redundant technological stress or baseline usage, defensive motivation serves as an indispensable protective coping mechanism that channels perceived external vulnerability into constructive, post-adoptive behavioral engagement.

2.4. Research Hypotheses

Based on the literature review, this paper proposes a dual-path model to explain how AI threat perception affects agent use behavior.

First, drawing on Cognitive Appraisal Theory, evaluations of environmental stressors shape individuals' coping orientations and resource development (Yu et al., 2023). Although AI threat perception stems from replacement and skill obsolescence risks, it also signals an urgent demand for task adaptation. In knowledge-intensive environments, perceived technological disruption can activate problem-focused coping efforts aimed at restoring personal control and task mastery (Zhang & Lu, 2009). Rather than solely inducing withdrawal, the perceived threat of obsolescence underscores the imperative of competence development, prompting knowledge workers to engage proactively with emerging AI tools and cultivate confidence in their operational capabilities. Consequently, higher levels of technological threat perception are expected to co-occur with strengthened capability beliefs, manifesting as enhanced AI self-efficacy. Thus, we propose the following hypothesis.

**H1**: AI threat perception is positively associated with AI self-efficacy.

Second, according to Defense Motivation Theory, when individuals face high levels of automation or external threats, such as full AI replacement, and perceive a threat to autonomy, defensive motivation arises. This external pressure, such as the threat of job marginalization, triggers defensive motivation. It prompts them to actively learn AI skills (Passalacqua et al., 2024). Thus, we propose the following hypothesis.

**H2**: AI threat perception is positively associated with defensive motivation.

Third, AI self-efficacy, as an internal capability belief, can significantly foster advanced post-adoptive behaviors. Once initial adoption has occurred, users who possess high self-efficacy exhibit greater cognitive resilience and task autonomy, motivating them to venture beyond superficial features into deep workflow embedding, iterative prompt refinement, and bespoke agent customization (Zhao et al., 2025). Thus, we propose the following hypothesis.

**H3**: AI self-efficacy is positively associated with post-adoptive AI agent engagement.

Fourth, although defensive motivation is rooted in loss avoidance, within the post-adoptive stage it operates as a continuous protective driver. When active users realize that superficial usage is insufficient to insulate them from technological obsolescence, defensive motivation prompts them to intensify their engagement, expand exploratory usage, and promote team-level tool integration to solidify their indispensable organizational standing (Yang et al., 2019; Ng et al., 2021). Thus, we propose the following hypothesis.

**H4**: Defensive motivation is positively associated with post-adoptive AI agent engagement.

Fifth, from an exploratory perspective, an open theoretical question concerns whether an intrinsic link exists between AI self-efficacy and defensive motivation. Individuals with high self-efficacy often have a clearer understanding of AI's capability boundaries. They can also more accurately judge which job content is being eroded. This cognitive advantage makes them more sensitive to the competitive landscape. The more they understand AI, the clearer the risk of "being left behind if not following up." In other words, capability beliefs not only drive action but may also indirectly heighten risk alertness. To explore this tentative association, we examine the following proposition.

**H5**: AI self-efficacy is positively associated with defensive motivation.

Sixth, grounded in career stage theory, individuals in the career establishment and advancement phase (spanning ages 25 to 45) face critical professional consolidation milestones alongside acute career transition pressures (Symanyuk et al., 2019). Compared to early-career entrants with minimal sunk capital or late-career cohorts approaching retirement, workers in this establishment stage confront pronounced promotional bottlenecks, family responsibilities, and rigid institutional tenure evaluations. Within knowledge-intensive sectors, career advancement anxiety during this pivotal window acts as a psychological catalyst: to maintain competitiveness and resolve obsolescence crises, these workers are under heightened pressure to convert technological threat perception into proactive capability building and AI self-efficacy. Given that the sample is naturally concentrated in core career stages, comparing this cohort against an aggregated baseline of outer age brackets reflects a practical trade-off to ensure statistical power in multi-group SEM; accordingly, this boundary examination is interpreted with appropriate caution as an exploratory analysis. Accordingly, we propose the hypothesis.

**H6**: Age moderates the association between AI threat perception and AI self-efficacy, such that the positive association is stronger among early-to-mid career workers (aged 25–45).

Seventh, based on Human Capital Theory, highly educated groups (e.g., graduate level and above) usually possess stronger information processing capabilities and technical understanding (Narine et al., 2023). This cognitive advantage allows them to perceive the real risks of technological unemployment more clearly. It also makes them more inclined to adopt instrumental coping strategies. They quickly calculate that "learning AI" is the optimal solution to avoid risks, thereby converting threat perception into defensive motivation more tightly. Accordingly, we propose the hypothesis.

**H7**: Education level moderates the association between AI threat perception and defensive motivation, such that the positive association is stronger among postgraduates.

Eighth, drawing on environmental and contextual perspectives, geographic regions differ noticeably in technology exposure and digital ecosystem vitality (Salazar-Concha et al., 2021). In developed eastern coastal areas, where intelligent tools are integrated earlier and more pervasively into daily business workflows, workers are constantly exposed to peer modeling and higher industry expectations regarding AI fluency. In this technology-dense environment, when knowledge workers perceive professional disruption from AI, they encounter stronger immediate incentives and richer opportunities to experiment directly with agent systems. Rather than responding passively, individuals in these regions are more likely to channel perceived technological disruption into hands-on mastery, thereby more vigorously translating AI threat perception into personal AI self-efficacy. Accordingly, we propose the hypothesis:

**H8**: Region moderates the association between AI threat perception and AI self-efficacy, such that the positive association is stronger in eastern (developed) regions.

Ninth, based on the above analysis, to fully reveal the mechanism of AI threat perception on agent use behavior, this study will examine the mediating role of AI self-efficacy and defensive motivation. It will also test the direct effect of AI threat perception on use behavior. Thus, we propose the following hypotheses.

**H9a**: AI self-efficacy mediates the positive association between AI threat perception and Post-Adoptive AI Agent Engagement.

**H9b**: Defensive motivation mediates the positive association between AI threat perception and Post-Adoptive AI Agent Engagement.

2.5. Research Framework

This study integrates conceptual insights from Cognitive Appraisal Theory and Protection Motivation Theory to construct a theoretical model with multiple core paths (see Figure 1). Unlike traditional Technology Acceptance Models that focus mainly on perceived usefulness and ease of use, this study focuses on how threat assessment connects with adaptive coping behavior through dual theoretical lenses. We emphasize that our structural model does not attempt to formally test the full structural architectures of Cognitive Appraisal Theory or Protection Motivation Theory, as situational challenge and threat appraisal states, response efficacy, and response costs were not directly measured. Instead, these frameworks serve as theoretical foundations explaining how technological stressors can simultaneously relate to capability-enhancing beliefs (AI self-efficacy) and proactive loss-prevention orientations (defensive motivation).

Figure 1. Research Framework.

Note. In the hypothesized structural model, AI threat perception is expected to be positively associated with AI self-efficacy (H1) and defensive motivation (H2). AI self-efficacy (H3) and defensive motivation (H4) are hypothesized to positively predict Post-Adoptive AI Agent Engagement. H5 examines the exploration path from AI self-efficacy to defensive motivation. H9a and H9b specify the dual mediating roles of AI self-efficacy and defensive motivation in connecting AI threat perception with Post-Adoptive AI Agent Engagement. H6, H7, and H8 specify the moderating roles of age, education level, and geographic region, respectively.

3. Research Methodology

3.1. Interview Exploration

To ensure the research questions are grounded in practical contexts, this study first employed qualitative interviews. The goal was to explore the true motivations behind user learning and agent usage. The research team recruited volunteers from agent learning and developer communities for semi-structured interviews. The respondents included five researchers, three corporate IT or digitalization technicians, two middle-level enterprise managers, two legal and consulting practitioners, and one freelancer. This totaled thirteen individuals, ensuring diversity in professional backgrounds. These personnel structures also reflect that the agent learning, and development group is mainly composed of knowledge-intensive populations.

Data collection strictly adhered to ethical standards throughout the process. Prior to data collection, the study protocol underwent institutional ethical review by the Institutional Review Board (IRB) of the School of Economics and Finance at Xi’an Jiaotong University and was determined to be exempt from full board oversight in accordance with national and institutional ethical guidelines for non-invasive, minimal-risk social science research. Verbally informed consent was obtained from all participants prior to each interview, with written consent documentation formally waived to protect participant anonymity. Researchers fully explained the study's objectives, the voluntary nature of participation, the right to withdraw at any time, and the measures taken to ensure confidentiality of data. Regarding data recording, several participants expressed privacy concerns and declined audio recording. To respect their preferences while ensuring textual rigor, two researchers attended each interview and independently recorded comprehensive, contemporaneous field notes. Immediately following each session, the two researchers cross-validated and reconciled their notes to resolve any discrepancies, producing a consolidated, cross-checked interview summary for subsequent coding.

The interview outlines focused on three core questions. These were the reasons for initial contact with agents, the most profound feelings during use, and the factors that prompted deep usage or even development of agents. Each interview lasted approximately ten to twenty minutes.

3.2. Coding Analysis and Core Theme Extraction

The research team used the three-level coding process of grounded theory to analyze the interview texts and field notes. In the open coding stage, two researchers independently coded the materials, and 86 initial concepts were extracted. Examples included mentor requirements, simple operation, fear of falling behind, worry about elimination, sense of control, and ease of learning. To evaluate inter-coder reliability, the categorizations made by both coders across preliminary categories were statistically compared. The two coders achieved an observed raw agreement of 72.1% (62 out of 86 concepts). Accounting for the expected agreement by chance across the six categories (18.4%), the resulting Cohen's kappa was 0.66, indicating substantial inter-coder consistency. All 24 conceptual discrepancies were resolved through consensus discussions with the corresponding author.

Thematic saturation was evaluated by examining code accumulation across sequential interviews. While new conceptual codes accumulated rapidly during the initial interviews, subsequent transcripts from the 11th interview onward repeatedly mapped onto existing categories without generating novel themes. The final two interviews confirmed that theoretical saturation had been achieved, as participants' reflections across varied occupational backgrounds were fully subsumed under the established coding categories without yielding new conceptual properties.

During the axial coding stage, the 86 initial concepts were systematically synthesized into six preliminary categories, which comprised external mandatory drive, social comparison pressure, efficacy experience and sense of control, practical value realization, emotional and cognitive transformation, and proactive integration behavior. Guided by the paradigm model of grounded theory, these categories were further linked through causal conditions, contextual phenomena, and intervening conditions, ultimately revealing that people usually start using agent tools driven by two psychological forces.

The first force stems from the progressive establishment of self-efficacy. When users try the tool for the first time, they find they can master it. They can complete tasks that were previously difficult and customize workflows to fit their needs. Consequently, they gradually build confidence in their AI usage capabilities. This "I can do it" belief prompts them to actively explore more features and try more complex application scenarios. They may even build personalized assistants, ultimately deeply integrating the tool into daily work. Several respondents expressed similar efficacy beliefs. Respondent 1 mentioned that as she gradually adjusted prompts, she found "I myself could fully use this tool very well." Respondents 3, 9, 10, and 11 all emphasized that learning agents is indeed relatively easy and operable. Respondent 6 pointed out that "operation is indeed very easy; even if someone knows nothing, just conversing is enough." Respondent 8 noted that "this thing is really easy to operate, and ordinary people can master it," while Respondent 11 stated that "as long as you are willing to practice, AI agents really have no threshold." Furthermore, Respondent 13 reflected that although she initially felt it was too difficult, having the tool generate draft designs based on prompts made her realize that "using it was actually so simple." The core feature of these statements lies in emphasizing subject capability belief, meaning "I can master it" or "learning is easy," rather than judging the value of the tool itself.

The second force comes from external pressure driven by defensive motivation. Some users started using AI due to organizational mandates, platform rules, or observing that colleagues and younger team members generally use it. They worried about falling behind or appearing out of touch. This fear of negative consequences prompted users to take the first step. Initially, it might be just for compliance. However, if they establish efficacy beliefs during use, they gradually shift from passive to active. Interview data showed Respondent 1 felt pushed by a mentor and noted that literature reviews can no longer rely solely on manual work. Respondent 2 feared being eliminated. Respondent 8 mentioned that corporate performance penalties and fear pushed them through the door. Respondent 9 noted the risk of budget cuts if the department failed to submit AI efficiency cases. Respondent 10 did not want to be seen as an "old fossil." Respondent 11 mentioned a mandatory requirement during the probation period. The core feature of these statements lies in emphasizing external threat perception, including elimination, performance downgrades, budget cuts, and social comparison pressure.

Further analysis found that these two forces often coexist in individuals. Many respondents stated they faced the risk of elimination if they did not use AI. Yet, they also mentioned discovering they could master the tool after actual trials. Respondent 8 explicitly stated, "Fear pushed me in, but solving problems kept me." Respondent 9 said, "At first I thought it was a hassle, now I really can't live without it." Respondent 13 mentioned survival pressure forced them in, and not learning would lead to being surpassed by competitors. Respondent 10 shifted from not wanting to be an "old fossil" to saying, "I now even dare to say in meetings that I will let the tool run a prototype for this requirement first." This indicates that defensive motivation and self-efficacy are not opposed but are part of a co-acting process. In knowledge-intensive environments like universities, enterprises, and law firms, users often take the first step due to external pressure. They are willing to go deeper because of the personal experience that they could utilize the tool and the low learning threshold. It is this dual mechanism of external push and internal efficacy belief that prompts individuals to move from initial, tentative usage toward profound post-adoptive engagement, characterized by active feature customization and collaborative workflow integration. This trajectory reflects an ongoing post-adoptive transition integrating survival pressure, professional dignity, and practical wisdom.

Based on the coding analysis results, this study operationalized two core psychological constructs for subsequent questionnaire design. The first is defensive motivation, which mainly measures the driving force of use arising from external pressure, career threats, and social comparison. The second is self-efficacy, which mainly measures the user's confidence and sense of control over their AI usage ability. This includes dimensions such as confidence in understanding operational logic, learning ability, and sense of control. Measurement items for the two constructs were adapted from validated scales and contextualized using typical expressions from the interviews. This ensures the scales have good face validity and content validity.

3.3. Questionnaire Design and Variable Measurement

Based on the core psychological mechanisms revealed by interview exploration and coding analysis, this study translated qualitative findings into operational quantitative variables. A structured questionnaire was developed accordingly. Interview results indicated that individual adoption of AI agents is accompanied by two primary psychological forces. One is defensive motivation triggered by external pressure, such as fearing technological elimination or workplace marginalization. The other is AI self-efficacy formed from positive experiences, which is confidence in the ability to master and apply AI tools. To systematically test this dual-drive mechanism, this study constructed four core constructs. These are AI threat perception, AI self-efficacy, defensive motivation, and post-adoptive AI agent engagement.

All constructs were measured using multi-item scales with a 7-point Likert scoring method (1 = strongly disagree, 7 = strongly agree). Item design was strictly based on mature scales from existing empirical research, grounded in the interview context. This ensures theoretical rationality and content validity. Specifically:

The AI threat perception scale integrates research by Gerlich (2024) on job replacement concerns, Yang et al. (2024) on rapid skill depreciation, and Sharma et al. (2024) on workplace social comparison pressure. It contains 5 items. These capture multidimensional threat perception regarding job stability, skill depreciation risk, work replacement possibility, competitive disadvantage, and career prospect uncertainty. Specific items cover the threat to career stability from agent development, the impact of AI on the market value of existing skills, the risk of automation replacement, the competitive disadvantage of not mastering tools, and uncertainty in career prospects caused by AI progress. To ensure the content validity of this adapted composite scale, all pooled items were contextualized using typical expressions from the preliminary interviews and were thoroughly reviewed by the research team for semantic clarity and situational relevance prior to formal survey distribution.

The AI self-efficacy scale is based on the classic computer self-efficacy scale by Compeau and Higgins (1995). This scale is widely used in information systems and has good reliability and validity. Combined with Bandura et al.'s (1999) self-efficacy theory and referencing Sundaresan and Zhang (2021) on high-level AI application capabilities, 5 items were formed. These cover confidence in learning complex AI tools, ability to understand basic operational logic, ability to effectively apply AI to work, ability to solve AI problems through self-study, and confidence in participating in agent customization or development.

The measurement of defensive motivation draws on empirical findings by Chen et al. (2024) and Wang and Xiao (2025) regarding AI threat perception initiating protective learning behavior. It also introduces Ryan and Deci's (2000) extrinsic motivation theory and Oliver's (1991) institutional pressure perspective. Five items were constructed. These reflect behavioral intentions to passively engage in AI learning to prevent unemployment or marginalization, cope with uncertainty, respond to external pressure, avoid organizational value decline, and meet basic workplace survival requirements(the full item wordings, grounded theoretical sources, and contextualization quotes from preliminary interviews are detailed in Table S1 in the Supplementary Material).

The post-adoptive AI agent engagement scale integrates usage frequency and workflow integration indicators proposed by Venkatesh et al. (2012). It also includes recommendation behavior from Murray's (2009) innovation diffusion theory. Combined with Dong et al.'s (2024) definition of knowledge worker participation in AI customization, 5 items were formed. These cover daily usage frequency, new platform or feature exploration, participation in customization or development, deep integration into core workflows, and recommendations to colleagues. These items cover not only basic use but emphasize deep integration, feature exploration, and co-creation behavior. This aligns highly with the actual practices of user groups in the interviews.

In addition to the core variables, the questionnaire collected demographic information. This included occupation, age, education, income, and gender. Occupation classification was optimized during the pre-test phase based on interview sample characteristics. Researchers were highlighted as an independent option to ensure a clear logic matching the research focus.

3.4. Data Collection and Analysis Strategy

This study distributed questionnaires via the Questionnaire Star platform. A combination of convenience sampling and snowball sampling was used to recruit participants. Crucially, because this investigation examines post-adoptive engagement rather than initial adoption decisions, sampling criteria strictly targeted active users who had incorporated AI agents into their weekly routines for at least six months (with a minimum threshold of ten minutes per week). To ensure data quality, users with average weekly usage under ten minutes were excluded. Invalid questionnaires with completion times under two minutes or identical scores for multiple items were also removed. Finally, 387 valid questionnaires were collected. The demographic characteristics of the sample are detailed in Table 1.

Table 1 shows that the sample presents significant knowledge-intensive and high-income characteristics. In terms of occupation distribution, digitalization technicians (34.63%) and researchers (31.01%) constituted the main body. Combined, they accounted for over 60%. Enterprise managers (20.93%) and legal/consulting professionals (6.98%) also held a certain proportion. This indicates respondents were mostly highly skilled professional groups. Regarding education, the sample had a high overall level. Those with bachelor's degrees or above accounted for 93.28%. There were nearly half of those with graduate degrees or above (48.58%). The age structure was mainly young and middle-aged adults aged 26 to 45 (72.87% combined). The 26 to 35 age group had the highest proportion (40.05%). This reflects that the core user group of AI agents is concentrated among professionals in the golden period of career development. Additionally, the proportion of male respondents (65.63%) was significantly higher than females. Regarding income level, nearly 84% (83.98%) of respondents had a monthly income over 10,000 yuan. This shows the group has a strong economic foundation. Geographically, the sample covered eastern, central, and western regions. The eastern region was slightly higher (40.57%), but the central and western regions combined were close to 60%. The distribution was relatively balanced. Overall, the sample has distinct industry representation and demographic characteristics. It is suitable for deeply exploring the post-adoptive AI agent engagement, attitudes, and associative mechanisms of knowledge workers regarding AI agents.

The study protocol underwent formal ethical review prior to data collection by the Institutional Review Board (IRB) of the School of Economics and Finance at Xi’an Jiaotong University and was determined to be exempt from full board oversight. Exemption was granted in accordance with national and institutional ethical guidelines for minimal-risk social science research. The research utilized fully anonymous, voluntary online questionnaires and non-invasive qualitative interviews, collecting no sensitive personal data or directly identifiable private information (such as names, employee IDs, or contact details) and posing minimal risk to participants. In accordance with institutional policy, exempt minimal-risk social science studies are not assigned a separate permit number, and the official determination of exemption serves as institutional clearance.

The data analysis for this study proceeded in the following four steps:

(1) Normality test and common method bias test. The data collected were tested to see if they met the multivariate normality assumption, which serves as a prerequisite for structural equation modeling. Simultaneously, methods like Harman's single factor test and the unmeasured latent methods factor (ULMC) approach were used to check for common method bias.

(2) Confirmatory factor analysis. The reliability and validity of the scales used in this study were tested, including internal consistency, convergent validity and discriminant validity.

(3) Path and mediation mechanism test. Structural equation modeling in AMOS 26.0 was employed to test the hypothesized structural associations and statistical significance between constructs. The bootstrap method (5,000 resamples) was used to test the indirect mediation effects of AI self-efficacy and defensive motivation.

(4) Multi-group analysis. Measurement invariance tests were first conducted using age, education level, and geographic region as grouping variables to establish metric invariance, satisfying the prerequisite for cross-group comparison. On this basis, path differences for H6, H7, and H8 were further tested across different age, education, and region groups using the critical ratio of differences (z-test) for parameter comparison alongside nested chi-square difference tests in SEM.

Table 1. Sample Descriptive Statistics

| **Variable** | **Category** | **Frequency (N)** | **Percentage(%)** |
| --- | --- | --- | --- |
| Age | Under 18 years | 0 | 0.00% |
|  | 18-25 years old | 72 | 18.60% |
|  | 26-35 years old | 155 | 40.05% |
|  | 36-45 years old | 127 | 32.82% |
|  | 46-55 years old | 27 | 6.98% |
|  | 56 years old and above | 6 | 1.55% |
| Gender | Male | 254 | 65.63% |
|  | Female | 133 | 34.37% |
| Education | Junior college and below | 26 | 6.72% |
|  | Bachelor’s degree | 173 | 44.70% |
|  | Postgraduate degree and above | 188 | 48.58% |
| Occupation | Researchers / R&D Personnel | 120 | 31.01% |
|  | Educators / Teaching Professionals | 6 | 1.55% |
|  | Digitalization & Tech Professionals | 134 | 34.63% |
|  | Corporate Managers / Administrators | 81 | 20.93% |
|  | Professionals (Legal, Consulting, etc.) | 27 | 6.98% |
|  | Others | 19 | 4.91% |
| Income(RMB) | < 5,000 | 4 | 1.03% |
|  | 5,000 – 9,999 | 58 | 14.99% |
|  | 10,000 – 19,999 | 176 | 45.48% |
|  | 20,000 – 34,999 | 89 | 23.00% |
|  | >=35000 | 60 | 15.50% |
| Region | Eastern China | 157 | 40.57% |
|  | Central China | 120 | 31.01% |
|  | Western China | 110 | 28.42% |

4. Results

4.1. Multivariate Normality and Common Method Bias Test

Before conducting structural equation modeling analysis, this study first tested whether the data met the multivariate normality assumption. The distribution characteristics of all 20 items were evaluated. Mahalanobis distance was calculated to examine multivariate outliers. The maximum value was 39.45, which is lower than the critical value of 45.31 for the χ² distribution at 18 degrees of freedom and a significance level of α = 0.001 (Stevens, 1984). This indicates no multivariate outliers in the data, satisfying the multivariate normality assumption.

Common method bias may exist in structural equation modeling analysis, potentially causing spurious correlations between variables. To test its impact, this paper used Harman's single-factor method and the unmeasured latent method factor (ULMC) method. In Harman's single-factor test, an unrotated exploratory factor analysis was performed on all items. The results showed that the first factor had an eigenvalue of 7.14 and explained 35.68% of the variance, which is far below the 50% threshold. Additionally, the ULMC test results showed that the method factor explained only 0.92% of the total variance. The minimum ratio of variance explained by substantive constructs to the method factor was 14.06 (standard > 3) (Liang et al., 2007). The variance of most items was driven mainly by substantive constructs. Although these statistical diagnostic tests suggest that common method variance is not the dominant driver of observed relationships, common method bias cannot be completely ruled out given the cross-sectional, single-source self-report design. Consequently, method variance may still partially contribute to the strength of the observed associations.

4.2. Reliability and Validity Test

Exploratory factor analysis(EFA) was first conducted for initial item screening across all 20 preliminary items (using principal component analysis with varimax rotation). After factor rotation, the four constructs aligned with the expected distribution. Specifically, one item for defensive motivation (DM4) and one item for Post-Adoptive AI Agent Engagement (AB4 in the initial pool) exhibited severe cross-loadings with other factors exceeding the 0.50 threshold (cross-loadings reached 0.567 and 0.557, respectively; see Table S2 in the Supplementary Material and raw output file 001exploratory factor analysis.htm). These two problematic items were consequently removed to ensure conceptual distinctiveness and indicator purity. Finally, 18 measurement items were retained for subsequent confirmatory factor analysis (CFA) and structural path estimation.

Based on the exploratory factor analysis, this study performed confirmatory factor analysis on each construct to test structural validity. For the AI threat perception dimension, the single-factor model fit was χ²(5) = 11.651, χ²/df = 2.330, p = 0.040, GFI = 0.988, AGFI = 0.964, CFI = 0.993, TLI = 0.986, RMSEA = 0.059. Although the RMSEA was slightly higher than the ideal standard of 0.05, it remained within the acceptable range of 0.08. Furthermore, incremental fit indices like CFI and TLI exceeded the strict standard of 0.95, indicating a good model fit.

The single-factor model fits for the other three constructs (AI self-efficacy, defensive motivation, and Post-Adoptive AI Agent Engagement) were also satisfactory, with all indicators reaching excellent levels (χ²/df < 2, GFI and AGFI > 0.90, RMSEA < 0.05). As reported in Table 2, standardized factor loadings across all retained items ranged from 0.636 to 0.818 (p < 0.001). Although item AA5 had a standardized loading of 0.636, it was retained because it exceeded the established 0.60 threshold for exploratory context adaptations and demonstrated strong conceptual relevance. Scale reliability and convergent validity were firmly supported, with Cronbach’s α ranging from 0.819 to 0.883, composite reliability (CR) from 0.830 to 0.869 (surpassing the 0.70 benchmark), and average variance extracted (AVE) from 0.549 to 0.572 (surpassing the 0.50 threshold). In summary, the measurement model has good structural validity, allowing for subsequent hypothesis testing.

Based on the convergent validity test, this study used the Fornell-Larcker criterion to further assess discriminant validity. The results showed that the square roots of AVE for each construct ranged from 0.741 to 0.756, which were all greater than the correlation coefficients between that construct and others (0.285 to 0.498). This meets the criteria for discriminant validity. Furthermore, the heterotrait-monotrait ratio of correlations (HTMT) was evaluated across all retained constructs to confirm discriminant distinctiveness. As reported in Table S3 in the Supplementary Material, all empirical HTMT values ranged from 0.276 to 0.504, remaining substantially below the conservative cutoff threshold of 0.85 (with the HTMT ratio between defensive motivation and AI threat perception being 0.488, and between defensive motivation and adoption engagement being 0.492). Combined with the confirmatory factor analysis results, the measurement model has good reliability, convergent validity, and discriminant validity, confirming that structural path analysis could proceed.

Table 2. Reliability and Validity Test

| **Dimension** | **Item** | **Unstd.** | **S.E.** | **Z** | **P** | **std.** | **Cronbach‘s α** | **CR** | **AVE** |
| --- | --- | --- | --- | --- | --- | --- | --- | --- | --- |
| AA | AA1 | 1 |  |  |  | 0.818 | 0.883 | 0.869 | 0.572 |
|  | AA2 | 0.952 | 0.057 | 16.762 | *** | 0.782 |  |  |  |
|  | AA3 | 0.921 | 0.058 | 15.935 | *** | 0.752 |  |  |  |
|  | AA4 | 0.945 | 0.056 | 16.766 | *** | 0.782 |  |  |  |
|  | AA5 | 1.000 |  |  |  | 0.636 |  |  |  |
| SE | SE1 | 1.165 | 0.101 | 11.512 | *** | 0.716 | 0.850 | 0.860 | 0.553 |
|  | SE2 | 1.099 | 0.097 | 11.277 | *** | 0.696 |  |  |  |
|  | SE3 | 1.284 | 0.103 | 12.504 | *** | 0.805 |  |  |  |
|  | SE4 | 1.222 | 0.099 | 12.338 | *** | 0.788 |  |  |  |
|  | SE5 | 1.000 |  |  |  | 0.705 |  |  |  |
| DM | DM1 | 1.026 | 0.082 | 12.47 | *** | 0.739 | 0.819 | 0.830 | 0.549 |
|  | DM2 | 1.051 | 0.083 | 12.738 | *** | 0.761 |  |  |  |
|  | DM3 | 0.984 | 0.082 | 12.001 | *** | 0.705 |  |  |  |
|  | DM4 | 1.000 |  |  |  | 0.758 |  |  |  |
| AB | AB1 | 0.975 | 0.071 | 13.751 | *** | 0.737 | 0.842 | 0.841 | 0.569 |
|  | AB2 | 1.064 | 0.071 | 14.925 | *** | 0.806 |  |  |  |
|  | AB3 | 0.964 | 0.071 | 13.543 | *** | 0.725 |  |  |  |
|  | AB4 | 0.920 | 0.058 | 15.833 | *** | 0.748 |  |  |  |

Note. Unstd. = unstandardized path coefficient; std. = standardized factor loading; *** indicates p < 0.01; ** indicates p < 0.05; * indicates p < 0.10. AA = AI threat perception. SE = self-efficacy. DM = defensive motivation. AB = Post-Adoptive AI Agent Engagement.

To verify construct distinctiveness and rule out potential indicator redundancy among the study variables, alternative CFA models were compared against the baseline measurement structure (see Table S4 in Supplementary Material: Questionnaire and Measurement Details). The proposed four-factor baseline model (χ² = 133.951, df = 129, CFI = 0.998, TLI = 0.998, RMSEA = 0.010) demonstrated a substantially better fit than alternative three-factor models combining defensive motivation with AI threat perception (Δχ² = 388.623, Δdf = 3) or with adoption engagement (Δχ² = 354.821, Δdf = 3), as well as the single-factor model (χ² = 1468.920, df = 135, CFI = 0.567, RMSEA = 0.160; Δχ² = 1334.969, Δdf = 6). These nested comparisons confirm the psychometric independence and robust construct validity across all focal variables.

4.3. Path and Mediation Mechanism Test

A structural equation model was constructed in AMOS 26.0 to test the hypothesized direct and indirect relationships among AI threat perception, AI self-efficacy, defensive motivation, and post-adoptive AI agent engagement. The structural model fit indices indicated an excellent fit to the data: χ²(129) = 133.951, χ²/df = 1.038, p = 0.365, GFI = 0.963, AGFI = 0.952, CFI = 0.998, TLI = 0.998, and RMSEA = 0.010. The parameter estimates for the direct structural paths are summarized in Table 3.

As presented in Table 3, H1 to H4 were fully supported by the empirical data (p < 0.001). AI threat perception was positively associated with AI self-efficacy (β = 0.486, p < 0.001) and defensive motivation (β = 0.460, p < 0.001). In turn, both AI self-efficacy (β = 0.411, p < 0.001) and defensive motivation (β = 0.409, p < 0.001) exerted significant positive impacts on post-adoptive AI agent engagement. However, H5 was not supported (β = 0.061, p = 0.350), indicating that the direct linkage between AI self-efficacy and defensive motivation was non-significant. Furthermore, the direct path from AI threat perception to post-adoptive AI agent engagement was non-significant (β = -0.059, p = 0.383).

Table 3. Path Test Results

| **Hypothesis** | **Path** | **Unstd.** | **S.E.** | **C.R.** | **p** | **Std. (β)** | **Result** |
| --- | --- | --- | --- | --- | --- | --- | --- |
| H1 | AA → SE | 0.377 | 0.049 | 7.636 | *** | 0.486 | Supported |
| H2 | AA → DM | 0.404 | 0.062 | 6.505 | *** | 0.460 | Supported |
| H3 | SE → AB | 0.493 | 0.082 | 6.003 | *** | 0.411 | Supported |
| H4 | DM → AB | 0.434 | 0.073 | 5.949 | *** | 0.409 | Supported |
| H5 | SE → DM | 0.069 | 0.074 | 0.934 | 0.35 | 0.061 | Not supported |
| Direct | AA → AB | -0.055 | 0.063 | -0.872 | 0.383 | -0.059 | Non-significant |

Note. Unstd. = unstandardized path coefficient. std. = standardized factor loading. *** indicates p < 0.01. ** indicates p < 0.05. * Indicates p < 0.10. AA = AI threat perception. SE = self-efficacy. DM = defensive motivation. AB = Post-Adoptive AI Agent Engagement.

To rigorously address the mediation mechanisms and distinguish between individual mediating pathways, a bootstrapping procedure with 5,000 resamples was performed in AMOS 26.0 using user-defined estimands. Both percentile and bias-corrected 95% confidence intervals were generated to test the specific indirect associations of self-efficacy (H9a), defensive motivation (H9b), and the potential serial mediation path. The detailed indirect effect decomposition is presented in Table 4.

As shown in Table 4, the specific indirect path linking AI threat perception to post-adoptive AI agent engagement via AI self-efficacy was statistically significant (Estimate = 0.186, S.E. = 0.037, bias-corrected 95% CI [0.122, 0.271], p < 0.001), providing empirical support for H9a. Similarly, the specific indirect path through defensive motivation was also statistically significant (Estimate = 0.176, S.E. = 0.039, bias-corrected 95% CI [0.109, 0.261], p < 0.001), lending support to H9b. Because neither 95% confidence interval enclosed zero, both variables exhibited viable mediating roles within the tested structural model.

In contrast, the serial mediation indirect pathway (AA → SE → DM → AB) was non-significant (Estimate = 0.011, S.E. = 0.012, bias-corrected 95% CI [-0.013, 0.037], p = 0.318), consistent with the non-significant direct link between SE and DM (H5). Furthermore, the direct association between AI threat perception and post-adoptive AI agent engagement remained non-significant in the presence of the mediators (Estimate = -0.055, S.E. = 0.065, bias-corrected 95% CI [-0.183, 0.076], p = 0.419).

Within the evaluated cross-sectional model, these empirical patterns are consistent with a statistical indirect-only framework, suggesting that the positive relationship between threat perception and employee engagement is largely transmitted through two concurrent psychological pathways: agency-driven self-efficacy and proactive loss-prevention motivation. Nonetheless, these findings should be interpreted with caution given the cross-sectional survey design. While the empirical estimates substantiate H9a and H9b statistically, they do not preclude alternative longitudinal dynamics, direct baseline effects, or other unmeasured contextual boundaries across diverse organizational settings.

Table 4. Indirect Effect Decomposition and Bootstrap Analysis

| **Path** | **Est.** | **S.E.** | **95% BC CI** | **p** | **Result** |
| --- | --- | --- | --- | --- | --- |
| AA → SE → AB | 0.186 | 0.037 | [0.122, 0.271] | 0.000 | H9a Supported |
| AA → DM → AB | 0.176 | 0.039 | [0.109, 0.261] | 0.000 | H9b Supported |
| AA → SE → DM → AB | 0.011 | 0.012 | [-0.013, 0.037] | 0.318 | Not supported |
| AA → AB (Direct) | -0.055 | 0.065 | [-0.183, 0.076] | 0.419 | Non-significant |

Note. N = 387; 5,000 bootstrap resamples. Est. = unstandardized point estimate; S.E. = bootstrap standard error; 95% BC CI = bias-corrected 95% confidence interval [lower bound, upper bound]. Corresponding 95% percentile confidence intervals are [0.120, 0.265] for SE indirect, [0.108, 0.259] for DM indirect, [-0.014, 0.036] for serial indirect, and [-0.187, 0.072] for direct effect. AA = AI Threat Perception; SE = AI Self-Efficacy; DM = Defensive Motivation; AB = Post-Adoptive AI Agent Engagement.

4.4. Multi-Group Analysis

Before conducting structural pathway comparisons, a measurement invariance (MI) test was performed across the focal moderating variables (age, education, and region) to assess the measurement properties functioned equivalently across demographic cohorts. Based on the evaluation framework proposed by Cheung and Rensvold (2002) and Chen (2007), configural invariance (an unconstrained baseline model) and metric invariance (equal factor loadings across groups) were evaluated sequentially. As presented in Table 5, the baseline configural models across all three cohorts exhibited satisfactory fit (all χ²/df ≤ 1.112, CFI ≥ 0.991). When factor loadings were constrained to be equal across groups, nested model comparisons indicated that the chi-square differences were non-significant for age (Δχ² = 10.743, Δdf = 14, p = 0.706), education (Δχ² = 9.875, Δdf = 14, p = 0.771), and region (Δχ² = 7.018, Δdf = 14, p = 0.934). In addition, changes in comparative fit indices remained minimal (all ΔCFI ≤ 0.001), lending empirical support to metric invariance across subgroups. Satisfying this prerequisite helps mitigate concerns that observed group differences stem from measurement discrepancies. Multi-group structural equation modeling was subsequently executed in AMOS 26.0, using nested chi-square difference tests and critical ratios of differences (z-scores) to evaluate structural variations across cohorts.

Table 5. Multi-Group Measurement Invariance Tests

| **Variable** | **Model Level** | **χ² (CMIN)** | **df** | **χ²/df** | **CFI** | **Comparison** | **Δχ²** | **Δdf** | **p** |
| --- | --- | --- | --- | --- | --- | --- | --- | --- | --- |
| Age | M1:Unconstrained | 289.057 | 260 | 1.112 | 0.991 | — | — | — | — |
|  | M2:Measurement weights | 299.800 | 274 | 1.094 | 0.992 | M2 vs. M1 | 10.743 | 14 | 0.706 |
| Education | M1:Unconstrained | 261.172 | 260 | 1.005 | 1.000 | — | — | — | — |
|  | M2:Measurement weights | 271.048 | 274 | 0.989 | 1.000 | M2 vs. M1 | 9.875 | 14 | 0.771 |
| Region | M1:Unconstrained | 257.968 | 260 | 0.992 | 1.000 | — | — | — | — |
|  | M2:Measurement weights | 264.985 | 274 | 0.967 | 1.000 | M2 vs. M1 | 7.018 | 14 | 0.934 |

Note. N = 387. Unconstrained = baseline configural invariance model with all parameters freely estimated across cohorts; Measurement weights = metric invariance model with factor loadings constrained to be equal across groups. CMIN = chi-square value; df = degrees of freedom; Δχ² = chi-square difference between nested models; Δdf = degrees of freedom difference; p = significance level of the nested chi-square difference test. Metric invariance is empirically confirmed when Δχ² is non-significant (p > 0.05) and ΔCFI ≤ 0.010. Reported CFI values of 1.000 occur when the model chi-square value is smaller than its corresponding degrees of freedom (χ²/df < 1.000), where AMOS automatically caps the comparative fit index at its theoretical upper limit.

First, regarding group differences in age, we conducted an exploratory multi-group analysis to examine structural variations across career stages. The sample was divided into an early-to-mid career group (aged 25 to 45, N=282) and a comparison group (under 25 and over 45 years, N = 105). This grouping is theoretically informed by career stage theory, which identifies the period spanning the mid-twenties to mid-forties as the career establishment and advancement phase. While classical career lifecycle models typically demarcate this establishment window from ages 25 to 44, the present study slightly extends the upper boundary to age 45 to align directly with institutional realities in Chinese knowledge-intensive sectors. Specifically, age 45 serves as the formal institutional eligibility ceiling for key national youth talent programs, tenure evaluations, and senior professional title reviews. Knowledge workers within this window experience both acute upward mobility pressures and established technical expertise, motivating them to actively cultivate AI self-efficacy in the face of perceived technological threats. Furthermore, from a methodological perspective, because 72.87% of the active users clustered within the 25–45 age bracket, disaggregating the remaining 105 participants into separate early-career (<25) and late-career (>45) cohorts would produce subgroup cell sizes insufficient to satisfy the statistical power requirements for multi-group SEM in AMOS (typically requiring over 100 cases per group for stable parameter estimation). Combining them into an overarching baseline group preserves model convergence while testing meaningful career-stage distinctions, though we acknowledge that pooling these outer cohorts introduces theoretical heterogeneity, warranting an exploratory interpretation of this empirical pattern.

The analysis results are shown in Table 6. Based on the nested chi-square difference test, the H1 path showed significant group differences (Δχ²(1)=9.696, p = 0.002). The positive association between AI threat perception and AI self-efficacy was significantly stronger in the early-to-mid career group (β = 0.580, p<0.001) than in the comparison group (β = 0.230, p = 0.049). This means career age stage has a significant moderating effect on the relationship between AI threat perception and AI self-efficacy, providing preliminary empirical support for H6 within this exploratory analysis. Group differences for paths H2 to H4 were not significant (p > 0.05).

Table 6. Age Moderation Test

| **Hypothesis** | **Path** | **Early-to-mid** | | **Others** | | **Difference** | | **Supported** |
| --- | --- | --- | --- | --- | --- | --- | --- | --- |
|  |  | **β** | **t-value** | **β** | **t-value** | **Δχ²(1)** | **P-value** |  |
| H1 | AA→SE | 0.580*** | 7.821 | 0.230** | 1.967 | 9.696 | 0.002 | YES |
| H2 | AA→DM | 0.445*** | 5.085 | 0.389*** | 3.250 | 0.000 | 0.997 | NO |
| H3 | SE→AB | 0.446*** | 6.017 | 0.249** | 2.182 | 0.852 | 0.356 | NO |
| H4 | DM→AB | 0.341*** | 4.764 | 0.448*** | 3.706 | 0.012 | 0.914 | NO |

Note. AA = AI threat perception. SE = self-efficacy. DM = defensive motivation. AB = Post-Adoptive AI Agent Engagement. β = coefficient. *** indicates p < 0.01; ** indicates p < 0.05; * indicates p < 0.10. Others refer to the comparison group (under 25 and over 45 years). Group difference tests are based on nested chi-square difference tests (Δχ², with Δdf = 1) comparing the model with an individual structural path constrained to be equal across groups against the unconstrained baseline model in AMOS 26.0. The age moderation is evaluated as an exploratory analysis.

Second, group differences in education. The sample was divided into a postgraduate group (master’s and Ph.D. degrees, N = 188) and an undergraduate and below comparison group (N = 199). This operationalization is informed by human capital theory, as workers with advanced degrees possess higher domain-specific knowledge investments and cognitive professional identity. Generative AI agents directly target higher-order analytical and synthesization tasks, making postgraduates particularly vulnerable to perceived status and skill depreciation, which intensifies their protective responses under technological threats.

The analysis results are shown in Table 7. The H2 path showed significant group differences (p = 0.039). The positive association between AI threat perception and defensive motivation was significantly stronger in the postgraduate group (β = 0.581, p < 0.001) than in the comparison group (β = 0.323, p < 0.001). This means education level has a significant moderating effect on the relationship between AI threat perception and defensive motivation, supporting H7. Group differences for paths H1, H3, and H4 were not statistically significant (p > 0.05).

Table 7. Education Level Moderation Effect Test

| **Hypothesis** | **Path** | **Postgraduate** | | **Others** | | **Difference** | | **Supported** |
| --- | --- | --- | --- | --- | --- | --- | --- | --- |
|  |  | **β** | **t-value** | **β** | **t-value** | **Δχ²(1)** | **P-value** |  |
| H1 | AA→SE | 0.508*** | 5.278 | 0.463*** | 5.444 | 0.000 | 0.992 | NO |
| H2 | AA→DM | 0.581*** | 5.594 | 0.323*** | 3.361 | 4.257 | 0.039 | YES |
| H3 | SE→AB | 0.389*** | 4.445 | 0.320*** | 4.465 | 1.085 | 0.298 | NO |
| H4 | DM→AB | 0.436*** | 5.111 | 0.390*** | 3.705 | 2.165 | 0.141 | NO |

Note. AA = AI threat perception. SE = self-efficacy. DM = defensive motivation. AB = Post-Adoptive AI Agent Engagement. β = coefficient. *** indicates p < 0.01; ** indicates p < 0.05; * indicates p < 0.10. Others refer to the comparison group (undergraduate and below). Group difference tests are based on nested chi-square difference tests (Δχ², with Δdf = 1) comparing the model with an individual structural path constrained to be equal across groups against the unconstrained baseline model in AMOS 26.0.

Third, group differences in region. The sample was divided into an eastern group(N = 157) and a central-western group(N = 230), capturing regional disparities in digital economic maturity and technological infrastructure. As summarized in Table 8, the H1 path showed significant group differences (p = 0.034). The positive association between AI threat perception and AI self-efficacy was stronger in the eastern group (β = 0.603, p < 0.001) than in the central-western group (β = 0.397, p < 0.001). This means region has a significant moderating effect on the relationship between AI threat perception and self-efficacy, supporting H8. Group differences for paths H2 to H4 were not statistically significant (p > 0.05).

Table 8. Region Moderation Effect Test

| **Hypothesis** | **Path** | **Eastern** | | **Central-Western** | | **Difference** | | **Supported** |
| --- | --- | --- | --- | --- | --- | --- | --- | --- |
|  |  | **β** | **t-value** | **β** | **t-value** | **Δχ²(1)** | **P-value** |  |
| H1 | AA→SE | 0.603*** | 6.068 | 0.397*** | 4.845 | 4.477 | 0.034 | YES |
| H2 | AA→DM | 0.395*** | 3.398 | 0.475*** | 5.334 | 0.104 | 0.747 | NO |
| H3 | SE→AB | 0.479*** | 4.799 | 0.324*** | 4.121 | 1.497 | 0.221 | NO |
| H4 | DM→AB | 0.307*** | 3.328 | 0.419*** | 5.122 | 0.544 | 0.461 | NO |

Note. AA = AI threat perception. SE = self-efficacy. DM = defensive motivation. AB = Post-Adoptive AI Agent Engagement. β = coefficient. *** indicates p < 0.01; ** indicates p < 0.05; * indicates p < 0.10. Group difference tests are based on nested chi-square difference tests (Δχ², with Δdf = 1) comparing the model with an individual structural path constrained to be equal across groups against the unconstrained baseline model in AMOS 26.0.

5. Discussion

5.1. Main Findings

This study used structural equation modeling and multi-group analysis in AMOS 26.0. We systematically examined the associative mechanisms linking AI threat perception to user adoption of AI agents. We also looked at its boundary conditions. The main findings are as follows.

First, AI threat perception is indirectly associated with Post-Adoptive AI Agent Engagement through dual paths of cognitive appraisal. This expands the perspective of traditional technostress research. Empirical results show that AI threat perception significantly and positively predicts AI self-efficacy and defensive motivation. These in turn are positively associated with Post-Adoptive AI Agent Engagement. This finding aligns conceptually with the broad logic of Cognitive Appraisal Theory. When knowledge workers face AI threats, their psychological responses are not unidimensional. While primary appraisal states were not directly measured in our survey, the observed empirical patterns are consistent with two distinct, concurrent coping orientations. On one hand, if they appraise it as a "challenge," they believe they can cope and benefit. This stimulates positive coping resources. It prompts individuals to invest cognitive resources to improve capabilities, meaning it enhances AI self-efficacy. On the other hand, if they appraise it as a "hindrance" or a "threat" to their status, they anticipate potential losses. This triggers protective motivation, or defensive motivation, to avoid risks or consolidate existing advantages. These two appraisals operate as distinct cognitive evaluations that jointly foster adaptive post-adoptive AI agent engagement. Unlike some previous studies suggesting that technology threat perception triggers avoidance, our investigation of seasoned knowledge workers demonstrates that threat perception functions as an ongoing impetus for post-adoptive deepening. Because these professionals have already crossed the threshold of initial adoption, technological threats do not induce functional abandonment; rather, they catalyze proactive capability upgrading and defensive workflow integration to maintain structural indispensability.

Second, individual characteristics have differentiated moderating effects. This reveals the boundary conditions of the cognitive appraisal process. Multi-group analysis shows that age, education, and region have significant moderating effects on specific paths. Specifically, the early-to-mid career group (25–45 years) and users in eastern regions exhibited stronger coefficients in the path from threat perception to AI self-efficacy, while the postgraduate group demonstrated a stronger link from threat perception to defensive motivation. While these regional differences are tentatively interpreted through the lens of environmental competition, we acknowledge that our survey did not directly quantify provincial digital economy indices or localized technology penetration. Hence, this regional divergence should be viewed as a preliminary macro-level pattern reflecting broader socioeconomic disparities rather than a definitive test of micro-level environmental pressure. Highly educated groups have stronger domain investments and cognitive capabilities. They can identify technological status depreciation more keenly. This generates stronger defensive learning motivation. In contrast, gender, occupation, and income did not show significant moderating effects. This indicates that the core cognitive appraisal mechanism is largely stable across groups in the face of this emerging technology. These heterogeneous boundary conditions resonate with configurational and contextual governance frameworks, which emphasize that individual and organizational adaptation under external constraints is rarely driven by isolated factors, but rather hinges on the alignment between specific resource endowments, institutional pressures, and contextual environments (Yang & Yang, 2026b).

Third, there is no significant association between AI self-efficacy and defensive motivation (H5 was not supported), reflecting the multidimensional and concurrent nature of cognitive appraisal in this cross-sectional model. While qualitative interview narratives (e.g., Respondent 8) described a dynamic, sequential progression where initial threat-driven defensive pressure preceded and later stimulated efficacy experiences, the quantitative structural model captures an empirical cross-section of active users who have already established stable usage patterns. In this established phase, capability beliefs and defensive intentions operate as two concurrent, non-competing psychological pathways within this cross-sectional observation. Future longitudinal research is needed to trace this temporal transition from early defensive compliance to mature capability consolidation.

Fourth, the direct association between AI threat perception and Post-Adoptive AI Agent Engagement is not significant in the presence of the mediators. This indicates that cognitive appraisal serves as a primary transmission conduit linking threat perception to behavioral responses in this sample. This finding aligns with the core view of Cognitive Appraisal Theory. Stressors do not automatically trigger behavioral outcomes. Instead, they depend on the individual's subjective assessment of their meaning. When threat perception is appraised as a "challenge," it is channeled into enhanced AI self-efficacy; When appraised as a "threat," it is channeled into defensive motivation. Within the current structural model, the empirical relationship is predominantly accounted for by these dual mediating mechanisms, resulting in an indirect-only statistical pattern. Rather than suggesting that technological threats exert no direct influence, this non-significant direct path may reflect competing psychological responses, including avoidance tendencies counterbalancing proactive learning, or the presence of unmeasured boundary conditions. Future research should incorporate affective indicators, such as technostress and emotional exhaustion, to further disentangle the direct and indirect dynamics of threat perception.

5.2. Theoretical Contributions

First, this study introduces Cognitive Appraisal Theory into the field of AI agent adoption, revealing a dual-path associative mechanism linking AI threat perception to behavior. Existing research mostly views technology-related threat perception as an inhibitor of technology acceptance. This study demonstrates that within the AI agent context, technological threat perception is positively associated with both capability-building (AI self-efficacy) and risk-mitigating (defensive motivation) pathways, both of which concurrently accompany Post-Adoptive AI Agent Engagement. By identifying how perceived technological disruption co-occurs with proactive adaptation rather than mere withdrawal, this finding enriches the application boundary of stress and coping frameworks in emerging human-AI collaboration.

Second, through multi-group analysis, this study identifies the group heterogeneity of the cognitive appraisal process, deepening the understanding of differential responses to technology threat perception. The moderating effects of career stage, education, and region indicate that cognitive appraisal is not a uniform psychological process. Instead, it is a contextualized mechanism embedded in specific career life cycles, human capital accumulation, and regional digital development. This not only explains why different groups react differently to AI threat perception but also provides empirical evidence for future research to segment user groups.

Third, this study shifts the research focus from general AI tools to AI agents as an emerging technology form. It reveals the psychological characteristics of knowledge workers in agent adoption, where "capability-driven" and "defense-driven" motivations coexist. This provides a grounded micro-psychological foundation for understanding user agency in human-agent collaboration.

5.3. Practical Implications

First, in the post-implementation phase, organizations should recognize that employee threat perception does not vanish after initial onboarding; rather, it persists as a salient psychological factor influencing deep feature utilization. Instead of suppressing technostress, management should channel this cognitive alertness into constructive pathways. By offering advanced prompt workshops and co-development platforms, organizations can help experienced workers translate technological anxiety into AI self-efficacy, catalyzing higher-order post-adoptive behaviors such as custom workflow automation and cross-functional agent deployment.

Second, organizations should adopt differentiated intervention strategies for different groups. For employees in the career establishment stage and those in developed regions, the linkage from threat to efficacy is more pronounced. Organizations should accelerate the conversion of threat awareness into capability confidence by establishing clear development paths and providing immediate skill feedback. For highly educated employees, the association between threat perception and defensive motivation is stronger. Organizations should emphasize the collaborative positioning of AI agents, mitigating perceptions of immediate occupational displacement and guiding workers to transform defensive anxiety into constructive learning motivation.

Third, create a supportive organizational atmosphere that ensures employees can obtain sufficient psychological and resource support when they feel threatened, encouraging proactive engagement with AI agents rather than avoidance behaviors.

6. Conclusions

6.1. Research Conclusions

Based on Cognitive Appraisal Theory, this study constructed a theoretical model examining the structural relationships between AI threat perception and Post-Adoptive AI Agent Engagement. We conducted empirical tests using structural equation modeling and multi-group analysis. The main conclusions are as follows.

First, AI threat perception is indirectly associated with Post-Adoptive AI Agent Engagement through a dual-path cognitive appraisal structure, exhibiting an indirect-only statistical mediation pattern within the cross-sectional model. AI threat perception can be appraised by knowledge workers as a "challenge" and a "threat." The former stimulates self-efficacy, whereas the latter stimulates defensive motivation. Both paths are positively associated with Post-Adoptive AI Agent Engagement. The direct association is not significant, indicating that cognitive appraisal serves as a primary statistical conduit linking threat perception to behavioral responses in this sample.

Second, capability-building and risk-mitigating orientations exhibit concurrent statistical mediation within the evaluated cross-sectional model. AI Self-efficacy and defensive motivation each demonstrate statistically viable specific indirect linkages between AI threat perception and post-adoptive AI agent engagement, with comparable effect sizes. This suggests that once initial adoption is established, competence growth and defensive vigilance can statistically coexist as co-occurring psychological correlates accompanying sustained engagement. However, because cross-sectional data cannot verify temporal independence, these empirical findings should not be interpreted as proving that the two mechanisms are psychologically isolated or mutually exclusive; rather, the potential dynamic or sequential interplay between defense and efficacy remains an open question for longitudinal investigation.

Third, boundary conditions exist across demographic and geographic cohorts. Specifically, the capability pathway linking AI threat perception to AI self-efficacy proved stronger among early-to-mid career professionals and participants in eastern regions, whereas the defensive motivation pathway was more pronounced among postgraduates. For the regional difference, this pattern suggests that employees in technologically advanced coastal environments may experience more direct competitive pressure, prompting them to translate threat perception into active practice and self-efficacy more readily. Nonetheless, because regional divisions capture broad geographic contexts rather than direct, micro-level measurements of local AI penetration or individual environmental stressors, this observed regional divergence should be interpreted cautiously as a contextual pattern warranting more granular multi-level verification.

6.2. Research Limitations and Future Outlook

This study has several limitations that should be acknowledged.

First, the cross-sectional design does not allow for definitive causal inferences, as threat perception, self-efficacy, and defensive motivation may exhibit reciprocal and dynamic relationships over time. Additionally, because all focal variables were gathered simultaneously via single-source, self-reported surveys without procedural separation (such as time-lagged sampling), common method variance cannot be completely ruled out despite statistical diagnostic tests (Harman's single factor and ULMC). Future research should employ multi-wave longitudinal sampling or objective behavioral telemetry to eliminate single-source dependency.

Second, regarding theoretical operationalization, primary cognitive appraisals (i.e., distinct challenge versus threat appraisal states) were inferred through downstream self-efficacy and defensive motivation rather than operationalized as explicit, independent measurement constructs. Future research should incorporate direct situational appraisal scales to more rigorously test the cognitive appraisal process.

Third, regarding measurement validation, the composite scale for AI threat perception was adapted from literature and contextualized via author review and qualitative interviews, without an external expert panel evaluation or pilot testing. Moreover, conducting both exploratory factor analysis (EFA) and confirmatory factor analysis (CFA) on the same sample (N = 387) carries risks of capitalization on chance, warranting independent sample cross-validation in future studies.

Fourth, the non-probability sampling approach (convenience and snowball recruitment via a single online platform) yielded a self-selected, knowledge-intensive sample skewed toward male (65.63%), high-income (83.98% earning over 10,000 RMB/month), and highly educated (93.28% holding a bachelor’s degree or above) professionals. This elite profile introduces demographic range restriction that specifically constrains the moderation findings. For education, because only 6.72% of respondents had an education level of junior college or below, the analysis effectively compared postgraduates against bachelor's degree holders rather than capturing broad educational disparities. Furthermore, regarding demographic and geographic moderation, both age and region were operationalized into aggregated cohorts (e.g., pooling respondents outside the 25 to 45 age range to satisfy multi-group SEM cell-size requirements and combining Central and Western regions into a single inland comparison group). While theoretically informed by institutional promotion thresholds and the macro-level economic divide between coastal digital vanguards and inland areas, this aggregation inevitably discards continuous variance and obscures intra-regional disparities within central and western provinces. Future research should deploy stratified probability sampling across broader demographics and measure chronological age continuously to test non-linear career-stage dynamics through moderated polynomial regression.

Fifth, potential cultural boundary conditions were not accounted for, even though systematic differences in technological threat interpretation may exist between collectivist and individualist cultural contexts.

Based on these limitations, future research could focus on the following directions. First, conduct longitudinal tracking or experimental studies to explore how users' cognitive appraisal processes evolve over time. Second, incorporate direct measures of appraisal states and cross-validate the adapted scales using independent holdout samples. Third, expand cross-group comparative studies to non-knowledge and older cohorts. Fourth, advance cross-cultural comparative studies to test the cultural generalizability of the conclusions. Fifth, enrich the system of outcome variables by incorporating emotional indicators (e.g., technostress or AI excitement) to unpack the underlying reasons for the non-significant direct association.

**Author Contributions:** Conceptualization, G.Y. and W.Y.; methodology, G.Y.; formal analysis, G.Y. and M.F.; investigation, G.Y. and M.F.; data curation, G.Y. and M.F.; writing—original draft preparation, G.Y.; writing—review and editing, M.F. and W.Y.; supervision, W.Y.; funding acquisition, W.Y. All authors have read and agreed to the published version of the manuscript.

**Funding:** This research was funded by the Shaanxi Social Science Fund Project, grant number 2025D001.

**Institutional Review Board Statement:** The research protocol underwent ethical review by the Institutional Review Board (IRB) of the School of Economics and Finance at Xi’an Jiaotong University and was determined to be exempt from full board oversight prior to data collection. Exemption was granted in accordance with institutional guidelines and national ethical frameworks for low-risk social science investigations, as the study utilized fully anonymous, minimal-risk surveys and non-invasive qualitative interviews without collecting personally identifiable information. In accordance with institutional policy, exempt minimal-risk social science projects are not assigned a formal permit number, and the institutional determination of exemption serves as official ethical clearance.

**Informed Consent Statement:** Written informed consent for participation was not required for this study, as explicitly authorized in the ethical approval document. An informed consent statement outlining the study's purpose, anonymity, and voluntary nature was presented on the first page of the questionnaire, and the submission of the completed questionnaire was considered as implied consent.

**Data Availability Statement:** Data is available from the corresponding author on reasonable request.

**Conflicts of Interest:** The authors declare no conflicts of interest.

References

1. Acemoglu, D., & Restrepo, P. (2018). Artificial intelligence, automation and work (NBER Working Paper No. 24196). National Bureau of Economic Research. [https://doi.org/10.3386/w24196](https://doi.org/10.3386/w24196)
2. Bandura, A., Freeman, W. H., & Lightsey, R. (1999). Self-efficacy: The exercise of control. *Journal of Cognitive Psychotherapy*, 13(2), 158–166. [https://doi.org/10.1891/0889-8391.13.2.158](https://doi.org/10.1891/0889-8391.13.2.158)
3. Bećirović, S., Polz, E., & Tinkel, I. (2025). Exploring students’ AI literacy and its effects on their AI output quality, self-efficacy, and academic performance. *Smart Learning Environments*, 12(1), Article 45. [https://doi.org/10.1186/s40561-025-00384-3](https://doi.org/10.1186/s40561-025-00384-3)
4. Brynjolfsson, E. (2022). The Turing trap: The promise & peril of human-like artificial intelligence. *Daedalus*, 151(2), 272–287. [https://doi.org/10.1162/daed_a_01915](https://doi.org/10.1162/daed_a_01915)
5. Chen, C., Hu, W., & Wei, X. (2024). From anxiety to action: Exploring the impact of artificial intelligence anxiety and artificial intelligence self-efficacy on motivated learning of undergraduate students. *Interactive Learning Environments*, 33(4), 3162–3177. [https://doi.org/10.1080/10494820.2024.2440877](https://doi.org/10.1080/10494820.2024.2440877)
6. Chen, F. F. (2007). Sensitivity of goodness of fit indexes to lack of measurement invariance. *Structural Equation Modeling: A Multidisciplinary Journal*, 14(3), 464–504. [https://doi.org/10.1080/10705510701301834](https://doi.org/10.1080/10705510701301834)
7. Cheung, G. W., & Rensvold, R. B. (2002). Evaluating goodness-of-fit indexes for testing measurement invariance. *Structural Equation Modeling: A Multidisciplinary Journal*, 9(2), 233–255. [https://doi.org/10.1207/s15328007sem0902_5](https://doi.org/10.1207/s15328007sem0902_5)
8. Compeau, D. R., & Higgins, C. A. (1995). Computer self-efficacy: Development of a measure and initial test. *MIS Quarterly*, 19(2), 189–211. [https://doi.org/10.2307/249688](https://doi.org/10.2307/249688)
9. Dong, X., Tian, Y., He, M., & Wang, T. (2024). When knowledge workers meet AI? The double-edged sword effects of AI adoption on innovative work behavior. *Journal of Knowledge Management*, 29(1), 113–147. <https://doi.org/10.1108/JKM-02-2024-0222>
10. Elavsky, S., & McAuley, E. (2003). Does the Physical Self-Efficacy Scale measure self-efficacy or self-esteem? *Medicine & Science in Sports & Exercise*, 35(Suppl 1), S138. [https://doi.org/10.1097/00005768-200305001-00763](https://doi.org/10.1097/00005768-200305001-00763)
11. Faisal, E. (2026). Complementary AI in higher education: Behavioral, cognitive, and ethical implications of ChatGPT and DeepSeek. *Frontiers in Psychology*, 17, Article 1699114. [https://doi.org/10.3389/fpsyg.2026.1699114](https://doi.org/10.3389/fpsyg.2026.1699114)
12. Gerlich, M. (2024). *Public anxieties about AI: Implications for corporate strategy and societal impact*. SSRN. [https://doi.org/10.2139/ssrn.5010191](https://doi.org/10.2139/ssrn.5010191)
13. Golgeci, I., Ritala, P., Arslan, A., McKenna, B., & Ali, I. (2025). Confronting and alleviating AI resistance in the workplace: An integrative review and a process framework. *Human Resource Management Review*, 35(2), Article 101075. [https://doi.org/10.1016/j.hrmr.2024.101075](https://doi.org/10.1016/j.hrmr.2024.101075)
14. Han, Z., Song, G., Zhang, Y., & Li, B. (2025). Trust the machine or trust yourself: How AI usage reshapes employee self-efficacy and willingness to take risks. *Behavioral Sciences*, 15(8), Article 1046. [https://doi.org/10.3390/bs15081046](https://doi.org/10.3390/bs15081046)
15. Hötte, K., Somers, M., & Theodorakopoulos, A. (2023). Technology and jobs: A systematic literature review. *Technological Forecasting and Social Change*, 194, Article 122750. [https://doi.org/10.1016/j.techfore.2023.122750](https://doi.org/10.1016/j.techfore.2023.122750)
16. Jöhnk, J., Weißert, M., & Wyrtki, K. (2020). Ready or not, AI comes—An interview study of organizational AI readiness factors. *Business & Information Systems Engineering*, 63(1), 5–20. [https://doi.org/10.1007/s12599-020-00676-7](https://doi.org/10.1007/s12599-020-00676-7)
17. Kumar, A., Krishnamoorthy, B., & Bhattacharyya, S. S. (2023). Machine learning and artificial intelligence-induced technostress in organizations: A study on automation-augmentation paradox with socio-technical systems as coping mechanisms. *International Journal of Organizational Analysis*, 32(4), 681–701. [https://doi.org/10.1108/IJOA-01-2023-3581](https://doi.org/10.1108/IJOA-01-2023-3581)
18. Li, R., Ouyang, S., & Lin, J. (2025). Mediating effect of AI attitudes and AI literacy on the relationship between career self-efficacy and job-seeking anxiety. *BMC Psychology*, 13(1), Article 123. [https://doi.org/10.1186/s40359-025-02757-2](https://doi.org/10.1186/s40359-025-02757-2)
19. Liang, H., Saraf, N., Hu, Q., & Xue, Y. (2007). Assimilation of enterprise systems: The effect of institutional pressures and the mediating role of top management. *MIS Quarterly*, 31(1), 59–87. [https://doi.org/10.2307/25148781](https://www.google.com/search?q=https://doi.org/10.2307/25148781)
20. Mantello, P., Ho, M.-T., Nguyen, M.-H., & Vuong, Q.-H. (2021). Bosses without a heart: Socio-demographic and cross-cultural determinants of attitude toward emotional AI in the workplace. *AI & SOCIETY*, 38(1), 97–119. [https://doi.org/10.1007/s00146-021-01290-1](https://doi.org/10.1007/s00146-021-01290-1)
21. Miraj, M., Chuntian, L., Mohd Said, R., Osei-Bonsu, R., & Rehman, R. ur. (2021). How information-seeking behavior, essential technologies, and resilience enhance the academic performance of students. *Frontiers in Psychology*, 12, Article 651550. [https://doi.org/10.3389/fpsyg.2021.651550](https://doi.org/10.3389/fpsyg.2021.651550)
22. Mishra, R., Singh, R. K., & Gunasekaran, A. (2023). Digitalization of supply chains in Industry 4.0 environment of manufacturing organizations: Conceptualization, scale development & validation. *Production Planning & Control*, 35(11), 1278–1297. [https://doi.org/10.1080/09537287.2023.2172622](https://doi.org/10.1080/09537287.2023.2172622)
23. Murray, C. E. (2009). Diffusion of innovation theory: A bridge for the research–practice gap in counseling. *Journal of Counseling & Development*, 87(1), 108–116. [https://doi.org/10.1002/j.1556-6678.2009.tb00556.x](https://doi.org/10.1002/j.1556-6678.2009.tb00556.x)
24. Narine, D., Yamashita, T., Chidebe, R. C. W., Cummins, P. A., Kramer, J. W., & Karam, R. (2023). Associations between education, information-processing skills, and job automation risks in the United States. *Journal of Adult and Continuing Education*, Advance online publication. [https://doi.org/10.1177/14779714231213004](https://doi.org/10.1177/14779714231213004)
25. Nasaj, M., Badi, S., Murtagh, N., & Ding, L. (2025). Collective AI anxiety and team innovative behaviour: The role of coping strategies. *Construction Innovation*, Advance online publication. [https://doi.org/10.1108/CI-08-2024-0222](https://doi.org/10.1108/CI-08-2024-0222)
26. Ng, K. C., Zhang, X., Thong, J. Y. L., & Tam, K. Y. (2021). Protecting against threats to information security: An attitudinal ambivalence perspective. *Journal of Management Information Systems*, 38(3), 732–764. [https://doi.org/10.1080/07421222.2021.1962601](https://doi.org/10.1080/07421222.2021.1962601)
27. Oh, J., & Lim, S. (2024). *How the anthropomorphism of an artificial intelligence agent affects the correction of misinformation: A focus on authenticity and behavioral-response system level*. SSRN. [https://doi.org/10.2139/ssrn.4809351](https://doi.org/10.2139/ssrn.4809351)
28. Oliver, C. (1991). Strategic responses to institutional processes. *Academy of Management Review*, 16(1), 145–179. [https://doi.org/10.2307/258610](https://doi.org/10.2307/258610)
29. Passalacqua, M., Pellerin, R., Yahia, E., Magnani, F., Rosin, F., Joblot, L., & Léger, P.-M. (2024). Practice with less AI makes perfect: Partially automated AI during training leads to better worker motivation, engagement, and skill acquisition. *International Journal of Human–Computer Interaction*, 41(4), 2268–2288. [https://doi.org/10.1080/10447318.2024.2319914](https://doi.org/10.1080/10447318.2024.2319914)
30. Rogers, R. W. (1975). A protection motivation theory of fear appeals and attitude change. *The Journal of Psychology*, 91(1), 93–114. [https://doi.org/10.1080/00223980.1975.9915803](https://doi.org/10.1080/00223980.1975.9915803)
31. Ryan, R. M., & Deci, E. L. (2000). Intrinsic and extrinsic motivations: Classic definitions and new directions. *Contemporary Educational Psychology*, 25(1), 54–67. [https://doi.org/10.1006/ceps.1999.1020](https://doi.org/10.1006/ceps.1999.1020)
32. Salazar-Concha, C., Ficapal-Cusí, P., Boada-Grau, J., & Camacho, L. J. (2021). Analyzing the evolution of technostress: A science mapping approach. *Heliyon*, 7(4), Article e06726. [https://doi.org/10.1016/j.heliyon.2021.e06726](https://doi.org/10.1016/j.heliyon.2021.e06726)
33. Schwabe, H., & Castellacci, F. (2020). Automation, workers’ skills and job satisfaction. *PLOS ONE*, 15(11), Article e0242929. [https://doi.org/10.1371/journal.pone.0242929](https://doi.org/10.1371/journal.pone.0242929)
34. Sharma, K., Davidson, B. G. J., George, J. P., & Muttungal, P. V. (2024). Breeding distrust during artificial intelligence (AI) era: How technological advancements, job insecurity and job stress fuel organizational cynicism? *Evidence-Based HRM: A Global Forum for Empirical Scholarship*, 13(4), 763–779. [https://doi.org/10.1108/EBHRM-05-2024-0159](https://doi.org/10.1108/EBHRM-05-2024-0159)
35. Stevens, J. P. (1984). Outliers and influential data points in regression analysis. *Psychological Bulletin*, 95(2), 334–344. [https://doi.org/10.1037/0033-2909.95.2.334](https://doi.org/10.1037/0033-2909.95.2.334)
36. Sundaresan, S., & Zhang, Z. (2021). AI-enabled knowledge sharing and learning: Redesigning roles and processes. *International Journal of Organizational Analysis*, 30(4), 983–999. [https://doi.org/10.1108/IJOA-12-2020-2558](https://doi.org/10.1108/IJOA-12-2020-2558)
37. Symanyuk, E., Borisov, G., Berdnikova, D., Tomberg, O., & Ryabukhina, A. (2019). Profession loss crisis at an old age: Specific features, factors, and mechanisms of coping. *Behavioral Sciences*, 9(12), Article 152. [https://doi.org/10.3390/bs9120152](https://doi.org/10.3390/bs9120152)
38. Venkatesh, V., Thong, J. Y. L., & Xu, X. (2012). Consumer acceptance and use of information technology: Extending the unified theory of acceptance and use of technology. *MIS Quarterly*, 36(1), 157–178. [https://doi.org/10.2307/41410412](https://doi.org/10.2307/41410412)
39. Vorobeva, D., El Fassi, Y., Costa Pinto, D., Hildebrand, D., Herter, M. M., & Mattila, A. S. (2022). Thinking skills don’t protect service workers from replacement by artificial intelligence. *Journal of Service Research*, 25(4), 601–613. [https://doi.org/10.1177/10946705221104312](https://doi.org/10.1177/10946705221104312)
40. Wang, C., & Xiao, A. (2025). Anxiety induced by artificial intelligence (AI) painting: An investigation based on the fear acquisition theory. *Psychological Trauma: Theory, Research, Practice, and Policy*, 17(Suppl 1), S214–S224. [https://doi.org/10.1037/tra0001862](https://doi.org/10.1037/tra0001862)
41. Wang, Y.-M., Wei, C.-L., Lin, H.-H., Wang, S.-C., & Wang, Y.-S. (2022). What drives students’ AI learning behavior: A perspective of AI anxiety. *Interactive Learning Environments*, Advance online publication, 1–17. [https://doi.org/10.1080/10494820.2022.2153147](https://doi.org/10.1080/10494820.2022.2153147)
42. Yang, G. (2026). *Unified adaptive agency theory: How the coupling of structural and processual subsystems drives organizational agency*. Research Square, Advance online publication. [https://doi.org/10.21203/rs.3.rs-9884228/v1](https://doi.org/10.21203/rs.3.rs-9884228/v1)
43. Yang, G., & Yang, W. (2026a). Macro-financial volatility exposure and corporate resilience: The dual contingencies of resource-capability alignment and cognition-learning adaptation. *Systems*, 14(9), Article 1121. [https://doi.org/10.3390/systems14091121](https://doi.org/10.3390/systems14091121)
44. Yang, G., & Yang, W. (2026b). Governing state-owned enterprises under constraint: A configurational approach to performance in the Global South. *International Journal of Public Administration*, 1–14. [https://doi.org/10.1080/01900692.2026.2687848](https://doi.org/10.1080/01900692.2026.2687848)
45. Yang, G., Yang, G., & Yang, W. (2025). The impact of AI on enterprise energy management: From the perspective of carbon emissions. *Science and Technology for Energy Transition*, 80, Article 8. [https://doi.org/10.2516/stet/2024096](https://doi.org/10.2516/stet/2024096)
46. Yang, H.-L., & Lin, S.-L. (2015). User continuance intention to use cloud storage service. *Computers in Human Behavior*, 52, 219–232. [https://doi.org/10.1016/j.chb.2015.05.057](https://doi.org/10.1016/j.chb.2015.05.057)
47. Yang, J., Amrollahi, A., & Marrone, M. (2024). Harnessing the potential of artificial intelligence: Affordances, constraints, and strategic implications for professional services. *The Journal of Strategic Information Systems*, 33(4), Article 101864. [https://doi.org/10.1016/j.jsis.2024.101864](https://doi.org/10.1016/j.jsis.2024.101864)
48. Yang, Q., Paul, K., & Pourtois, G. (2019). Defensive motivation increases conflict adaptation through local changes in cognitive control: Evidence from ERPs and mid-frontal theta. *Biological Psychology*, 148, Article 107738. [https://doi.org/10.1016/j.biopsycho.2019.107738](https://doi.org/10.1016/j.biopsycho.2019.107738)
49. Yu, L. R., Li, Y., & Fan, F. (2023). Employees’ appraisals and trust of artificial intelligences’ transparency and opacity. *Behavioral Sciences*, 13(4), Article 344. [https://doi.org/10.3390/bs13040344](https://doi.org/10.3390/bs13040344)
50. Zhang, T., & Tong, Q. (2025). The technostress of ChatGPT usage: How do perceived AI characteristics affect user discontinuous usage through AI anxiety and user negative attitudes? *International Journal of Human–Computer Interaction*, 41(16), 9918–9929. [https://doi.org/10.1080/10447318.2024.2429889](https://doi.org/10.1080/10447318.2024.2429889)
51. Zhang, Y. L., & Lu, C. Q. (2009). The relationship between challenge stressors-hindrance stressors and employees' work-related attitudes and behaviors: The moderating effects of general self-efficacy. *Acta Psychologica Sinica*, 41(6), 501–509. [https://journal.psych.ac.cn/xlxb/CN/Y2009/V41/I06/501](https://journal.psych.ac.cn/xlxb/CN/Y2009/V41/I06/501)
52. Zhao, Z., An, Q., & Liu, J. (2025). Exploring AI tool adoption in higher education: Evidence from a PLS-SEM model integrating multimodal literacy, self-efficacy, and university support. *Frontiers in Psychology*, 16, Article 1619391. [https://doi.org/10.3389/fpsyg.2025.1619391](https://doi.org/10.3389/fpsyg.2025.1619391)
