# Supplementary material for: How AI threat perception relates to post-adoptive AI agent engagement: the dual paths of self-efficacy and defensive motivation
Source: Front Psychol. 2026 Oct 22;17:1887571. doi: 10.3389/fpsyg.2026.1887571 (PMC13735560; doi:10.3389/fpsyg.2026.1887571)
Supplement: Supplementary file 1 [file Data_Sheet_1.zip › log+Questionnaire/Questionnaire and Measurement Details.docx]

# Table S1. Measurement Constructs, English Item Wordings, Theoretical Sources, and Qualitative Interview Grounding

| Construct / Item Code | English Survey Item Wording | Grounded Theoretical Source | Qualitative Grounding from Preliminary Interviews |
| --- | --- | --- | --- |
| AI Threat Perception (AA) | | | |
| AA1 | The rapid development of AI agents poses a serious threat to my future career stability. | Gerlich (2024) | Reflects structural replacement anxieties: "If this becomes the norm, won't people like us who rely on meticulous craft be eliminated soon?" (Participant 2). |
| AA2 | I perceive that AI technology is rapidly eroding the market value of my existing professional skills. | Yang et al. (2024) | Captures skill depreciation: "Originally, the barrier to design was high... now someone without experience can enter... you either adapt or get surpassed." (Participant 13). |
| AA3 | I believe that the tasks involved in my current work face a high risk of automated replacement by AI. | Gerlich (2024) | Aligns with job displacement risks: "The CTO announced that engineers who fail to use intelligent tools for efficiency will receive a failing performance grade C." (Participant 8). |
| AA4 | If I fail to master AI tools in a timely manner, I worry that I will be at a competitive disadvantage in the workplace. | Sharma et al. (2024) | Grounded in workplace social comparison: "During meetings, younger colleagues naturally discussed AI agents... I sat there understanding nothing... that look of surprise stayed with me." (Participant 10). |
| AA5 | Technological progress in AI makes me worry that my future career prospects are becoming uncertain. | Gerlich (2024); Yang et al. (2024) | Captures macro-level professional precariousness: "Peer firms have already made AI-driven solutions standard. I felt enormous pressure not updating my knowledge." (Participant 12). |
| AI Self-Efficacy (SE) | | | |
| SE1 | I believe I have the ability to learn and use complex AI agent tools. | Compeau & Higgins (1995); Bandura et al. (1999) | Captures foundational confidence: "As long as you are willing to practice, AI agents really have no threshold... mastery is easy." (Participant 11). |
| SE2 | Even without a technical background, I can understand the fundamental operational logic of AI agents. | Compeau & Higgins (1995) | Grounded in natural language ease: "Operation is very easy; even if someone knows nothing about coding, just conversing with it is enough." (Participant 6). |
| SE3 | I have the ability to effectively apply AI agents to my practical work tasks. | Sundaresan & Zhang (2021) | Reflects practical mastery: "I gradually adjusted prompts and found that I myself could fully use this tool very well." (Participant 1). |
| SE4 | When encountering AI-related problems, I can find solutions through autonomous self-study. | Compeau & Higgins (1995) | Captures self-directed problem-solving: "I followed online video tutorials and experimented with small tools... it proved very manageable." (Participant 9). |
| SE5 | I have the confidence to participate in customizing or developing basic functions of AI agents. | Sundaresan & Zhang (2021) | Grounded in agent customization: "I built a dedicated agent myself, uploading classic papers as a domain knowledge base." (Participant 3). |
| Defensive Motivation (DM) | | | |
| DM1 | I learn and use AI agents primarily to prevent future unemployment or occupational marginalization. | Chen et al. (2024); Ryan & Deci (2000) | Reflects loss-avoidance coping: "Fear pushed me through the door... not for promotion, but simply to feel psychologically secure." (Participant 8). |
| DM2 | Using AI is a necessary means for me to cope with occupational uncertainties brought by technological changes. | Wang & Xiao (2025) | Captures risk-hedging behavior: "Survival pressure forced me to start; if you don't learn, you will soon be left behind by competitors." (Participant 13). |
| DM3 | My engagement with AI is driven more by external workplace pressure than by personal interest. | Oliver (1991); Ryan & Deci (2000) | Reflects institutional and managerial mandates: "The CEO required every department to submit AI efficiency cases, otherwise budgets might be cut." (Participant 9). |
| DM4 (Dropped) | If I do not master AI, my value within the organization will significantly decline. | Oliver (1991) | Dropped during EFA screening due to conceptual blurring and cross-loading (>0.50) on AI Threat Perception. (Note: Reflected in Participant 10: "I don't want to be regarded as an old fossil"). |
| DM5 (CFA DM4) | I invest time in learning AI because it has become a baseline requirement for professional survival in the workplace. | Chen et al. (2024); Oliver (1991) | Reflects baseline workplace survival: "Our firm made using AI tools mandatory during the probation period... you either accept the change or get eliminated." (Participant 11). |
| Post-Adoptive AI Agent Engagement (AB) | | | |
| AB1 | I frequently use AI agents to assist with my daily professional work. | Venkatesh et al. (2012) | Captures daily usage routines: "I now use it on a daily basis; I really cannot live without it for routine duties." (Participant 2). |
| AB2 | I proactively explore new AI agent platforms or advanced features. | Venkatesh et al. (2012) | Reflects continuous feature exploration: "I am constantly experimenting to discover new features and see if multiple capabilities can be chained together." (Participant 7). |
| AB3 | Beyond using ready-made tools, I participate in customizing or developing my own AI agents. | Dong et al. (2024) | Captures user co-creation and agent orchestration: "To achieve better performance, I learned how to build and customize agents, even building an automated ops bot." (Participants 6, 10). |
| AB4 | I have deeply integrated AI agents into my core professional workflows. | Venkatesh et al. (2012) | Assesses structural workflow embedding: "I let it run the initial review of all contract drafts... it has become an indispensable part of my standard workflow." (Participant 11). |
| AB5 (Dropped) | I actively recommend the AI agents that I use to my colleagues. | Murray (2009) | Dropped during initial EFA screening due to high cross-loadings (>0.50) across multiple factors. (Note: Reflected in Participant 3: "A student recommended an agent to me"). |

*Note. All theoretical sources correspond strictly to the operational definitions established in Section 3.3 of the main text. Direct quotes represent translated contemporaneous interview statements collected during preliminary qualitative exploration. As described in Section 4.2 of the manuscript, two items (DM4 and AB5) were dropped during initial exploratory factor analysis (EFA) due to cross-loadings exceeding 0.50, yielding the final 18-item measurement model for subsequent confirmatory factor analysis (CFA) and structural modeling. Construct AB is labeled* ***"****Post-Adoptive AI Agent Engagement****"*** *to accurately capture deep and continuous usage behaviors among active users.*

# Table S2. Rotated Component Matrix from Initial Exploratory Factor Analysis (EFA)

| Item Code | Factor 1 (AA) | Factor 2 (SE) | Factor 3 (DM) | Factor 4 (AB) | Item Screening Decision & Rationale |
| --- | --- | --- | --- | --- | --- |
| AA1 | .817 |  |  |  | Retained |
| AA2 | .786 |  |  |  | Retained |
| AA3 | .780 |  |  |  | Retained |
| AA4 | .746 |  |  |  | Retained |
| AA5 | .796 |  |  |  | Retained |
| SE1 |  | .699 |  |  | Retained |
| SE2 |  | .758 |  |  | Retained |
| SE3 |  | .769 |  |  | Retained |
| SE4 |  | .782 |  |  | Retained |
| SE5 |  | .762 |  |  | Retained |
| DM1 |  |  | .772 |  | Retained |
| DM2 |  |  | .736 |  | Retained |
| DM3 |  |  | .748 |  | Retained |
| DM4 | .567 |  | .490 |  | Excluded (Cross-loading on Factor 1 > 0.50) |
| DM5 |  |  | .795 |  | Retained (Renumbered as DM4 in CFA) |
| AB1 |  |  |  | .789 | Retained |
| AB2 |  |  |  | .765 | Retained |
| AB3 |  |  |  | .801 | Retained |
| AB4 |  |  | .557 | .519 | Excluded (Cross-loading on Factor 3 > 0.50) |
| AB5 |  |  |  | .756 | Retained (Renumbered as AB4 in CFA) |

*Note. Extraction Method: Principal Component Analysis. Rotation Method: Varimax with Kaiser Normalization. Rotation converged in 6 iterations. Factor loadings below 0.30 are suppressed for clarity. As pre-specified in the methodology, items exhibiting severe cross-loadings greater than 0.50 (DM4 and AB4 in the initial pool) were excluded from subsequent confirmatory factor analysis (CFA) and structural modeling. The full raw SPSS EFA output file is provided as Supplementary File 001exploratory factor analysis.htm.*

# Table S3. Heterotrait-Monotrait Ratio of Correlations (HTMT) Analysis

| Construct | 1. AA | 2. SE | 3. DM | 4. AB |
| --- | --- | --- | --- | --- |
| 1. AI Threat Perception (AA) | — |  |  |  |
| 2. AI Self-Efficacy (SE) | 0.480 | — |  |  |
| 3. Defensive Motivation (DM) | 0.488 | 0.276 | — |  |
| 4. Post-Adoptive AI Agent Engagement (AB) | 0.346 | 0.504 | 0.492 | — |

# Table S4. Alternative CFA Model Comparisons

| Model | Chi-square | df | Chi-square/df | Delta df | GFI | AGFI | CFI | TLI | RMSEA |
| --- | --- | --- | --- | --- | --- | --- | --- | --- | --- |
| Four-factor (Baseline) | 133.951 | 129 | 1.038 | — | 0.963 | 0.952 | 0.998 | 0.998 | 0.010 |
| Three-factor (DM + AA) | 522.574 | 132 | 3.959 | 388.623 (3) | 0.829 | 0.778 | 0.873 | 0.853 | 0.088 |
| Three-factor (DM + AB) | 488.772 | 132 | 3.703 | 354.821 (3) | 0.831 | 0.781 | 0.884 | 0.866 | 0.084 |
| Single-factor (All items) | 1468.920 | 135 | 10.881 | 1334.969 (6) | 0.594 | 0.486 | 0.567 | 0.510 | 0.160 |

*Note. N = 387. AA = AI Threat Perception; SE = AI Self-Efficacy; DM = Defensive Motivation; AB = Post-Adoptive AI Agent Engagement. The baseline model consists of the 18 retained items. Delta Chi-square tests reflect comparisons against the baseline four-factor model. Critical values for Delta df = 3 and Delta df = 6 at p < 0.001 are 16.27 and 22.46, respectively.*
